# Supplementary material for: The burden of skin disease and eye disease due to onchocerciasis in countries formerly under the African Programme for Onchocerciasis Control mandate for 1990, 2020, and 2030
Source: PLoS Negl Trop Dis. 2021 Jul 26;15(7):e0009604. doi: 10.1371/journal.pntd.0009604 (PMC8312930; doi:10.1371/journal.pntd.0009604)
Supplement: S1 Text — (PDF) [file pntd.0009604.s001.pdf]

S1 Text: Detailed description of methods and results of sensitivity analyses

This document is a supplement to the following manuscript:

The burden of skin disease and eye disease due to onchocerciasis in Africa for 1990, 2020, and 2030

Authors:

Natalie V.S. Vinkeles Melchers[1]*, Wilma A. Stolk[1], Welmoed van Loon[1,2], Belén Pedrique[3], Roel Bakker[1], Michele E. Murdoch[4], Sake J. de Vlas[1], Luc E. Coffeng[1]*

Author affiliation:

1. Department of Public Health, Erasmus MC, University Medical Center Rotterdam, P.O. Box 2040, 3000 CA Rotterdam, The Netherlands.
2. Institute of Tropical Medicine and International Health, Charité-Universitätsmedizin, Berlin, Germany.
3. Drugs for Neglected Diseases *initiative* (DND*i*), Geneva, Switzerland.
4. Department of Dermatology, West Herts Hospitals NHS Trust, Watford General Hospital, Watford, Hertfordshire, UK.

*l.coffeng@erasmusmc.nl(LEC); *n.vinkelesmelchers@erasmusmc.nl(NVSVM)

Table of Contents

1. Definitions 3
2. Data 4
2.1 Pre-control infection levels 4
2.2 Population size 5
2.3 History of control 5
3. Mathematical modelling 8
3.1 Model background 8
3.2 Generic disease module 8
3.3 Simulations per APOC project 9
4. Disease burden calculation 13
4.1 Years Lived with Disability 13
4.2 Years of Life Lost 17
5. Sensitivity analysis 19
References 24

1. Definitions

Table A. List of terminology and definitions used in the manuscript.

Terminology	Definition
**Mf-positive person**	Someone who is positive for *Onchocerca volvulus* microfilariae (mf) in a single skin snip test.
**APOC project**	A geographical implementation unit for community-directed treatment with ivermectin (CDTi) under the formerly African Programme for Onchocerciasis Control (APOC). Each APOC project has its own organisational structure responsible for implementing the recommended CTDi strategy [1]; a list of all APOC projects is available in Supplement S3.
**Onchocercal eye disease (OED)**	Functional visual impairment or blindness. Following the WHO criteria, we define visual impairment as visual acuity in the range from worse than 6/18 to 6/60 and equal to or better than 3/60 in the better eye, which covers both moderate and severe visual impairment. According to the same guidelines, blindness was defined as visual acuity of less than 3/60 or a restriction of visual field to less than 10° in the better eye [2].
**Onchocercal skin disease (OSD)**	We considered six different subtypes of onchocercal skin disease (OSD), namely severe itch, reactive skin disease, palpable nodules, hanging groin, atrophy (<50 years of age), mild and severe depigmentation. We applied a publicly available clinical classification and grading system [3] to define the conditions.
**P5-project**	A combination of all areas in a country that are considered to be hypoendemic for onchocerciasis (based on REMO surveys showing nodule prevalence levels between 5% and 20%), that were not part of previously defined APOC projects, and that are likely to require treatment or other interventions for the purpose of onchocerciasis elimination [4].
**P20-project**	A combination of all areas in a country that are considered to be hyper- and mesoendemic for onchocerciasis (based on REMO surveys showing nodule prevalence levels of ≥20%), that were not part of previously defined APOC projects, and that are targeted for MDA for the purpose of onchocerciasis elimination [4].
**Pre-control onchocerciasis endemicity levels**	Onchocerciasis endemicity levels to which simulations were aggregated in our analysis are based on cut-offs in pre-control microfilariae prevalences (all ages) as defined by Prost *et al.* [5]: hypoendemic (<35%), mesoendemic (≥35% - <60%), hyperendemic (≥60%-<75%), and very hyperendemic (≥75%).

2. Data

2.1 Pre-control infection levels

We used a previously published raster map of the pre-control prevalence of onchocercal palpable nodules across 19 APOC countries (Angola, Burundi, Cameroon, Central African Republic, Chad, Congo, Democratic Republic of Congo, Equatorial Guinea, Ethiopia, Gabon, Kenya, Malawi, Mozambique, Nigeria, Rwanda, South Sudan, Sudan, Tanzania, Uganda), based on Rapid Epidemiological Mapping of Onchocerciasis (REMO) [6,7]. REMO surveys were carried out by APOC in a spatial sample of >14,000 villages, examining 30-50 adults for infection with onchocerciasis by assessing the presence of sub-cutaneous onchocercal nodules. Two APOC countries were found negative for *Onchocerca volvulus* infections (i.e. Kenya, Rwanda) [6] and were omitted from our analysis. We obtained the pixel-level infection data, and stratified each APOC project over six endemicity categories (**Table B**) by mean microfilariae (mf) prevalence to capture non-linearities between prevalence of infection and morbidity. We report results stratified by four endemicity categories, such as defined by Prost *et al.* (1979) [5] (**Table A**).

Table B. The estimated population at risk of onchocerciasis infection living in countries formerly under the APOC-mandate stratified over six endemicity categories. Population at risk for the year 1995 is based on a geostatistical analysis of REMO data combined with census data on the size of the population at risk in each geographical implementation unit. We then corrected the population densities of 1995 to 1990 (see section 2.2). Population numbers for the year 2030 are based on the assumption that populations grow by 2.75% each year and that the population does not move significantly between the different endemicity levels. Onchocerciasis endemicity levels are based on previous classification by Prost *et al*. [5] (**Table A**).

Onchocerciasis endemicity levels	Endemicity category for which analysis were performed	Cut-offs for prevalence of skin microfilariae in the general population (all ages)	Population at risk (millions)	
			1990	2030
Hypoendemic	1	≥ 1% - <35%	49.3	145.8
Mesoendemic	2	≥ 35% - <47%	13.3	39.2
	3	≥ 47% - <60%	8.7	26.5
Hyperendemic	4	≥ 60% - <66%	2.9	8.7
	5	≥ 66% - <75%	3.1	9.3
Very hyperendemic	6	≥ 75% - 100%	1.6	4.7
	**Total**		**78.9**	**234.4**

2.2 Population size

The population size per APOC project for 1995 was previously collected through a census conducted by community drug distributors for estimating the amount of ivermectin required in mass treatments. For projects where such information was not available, we used the population sizes as previously estimated and published by others [4,8]. We were interested in the population size for 1990 as some APOC projects already initiated treatment between 1990 and 1995. To correct the population size from 1995 to 1990, we used data from the United Nations Population Division [9]. On the basis of these data, we assumed an annual population growth of 2.75%. The pre-control population size for the year 1990 per APOC project was calculated by multiplying (1/1.0275)^5 with the available population size in 1995 (reduction in population size between 1995 and 1990). We estimated the population density for 2020 and 2030 by multiplying the pre-control country-specific population sizes by an assumed annual population growth factor of 1.0275^t, where t is the time in years.

2.3 History of control

We used a previously published APOC treatment database which contains information on pre-control endemicity, MDA start year, MDA frequency per year, and treatment coverage per APOC project up to November 2013 [8]. We adopted a recently updated version of the MDA data which includes some additional projects that were previously categorised as not requiring MDA, but do require MDA after all as described elsewhere [4]. For APOC projects where MDA was expected to start between 2013 and 2017, we consulted the ESPEN portal and corrected MDA start year where possible [10]. We also considered ivermectin distribution for lymphatic filariasis (LF) in areas that were endemic for both LF and onchocerciasis. For areas that initiated MDA before 2017 according to ESPEN, we assumed that MDA will continue until at least 2030 at the same coverage and frequency as previously reported [4,8]. For mesoendemic and hyperendemic areas that were not yet under MDA by 2017, according to ESPEN (in 2018), we assumed MDA initiation by 2019, irrespective of any feasibility concerns (e.g. post-conflict, *Loa loa*).

The WHO recommended in 2020 to suspend all epidemiological surveys and MDA activities for Neglected Tropical Diseases tackled by preventive chemotherapy and transmission control (PCT) due to severe acute respiratory syndrome coronavirus 2 disease 2019 (COVID-19) pandemic [11]. This has led to severe disruptions of MDA programmes throughout all onchocerciasis-endemic countries. To account for the COVID-19 pandemic in our analysis, we have set the therapeutic

coverage of MDA in the year 2020 to zero for all APOC projects; in case bi-annual MDA was provided both rounds were assumed to be missed.

We further assumed that untreated hypoendemic areas suspected of loiasis co-endemicity ("P5 areas", **Table A**) will remain untreated until at least 2025. This is in accordance with the *Mectizan Expert Committee/Technical Consultative Committee* (MEC/TCC) guidelines that recommended modified individual treatment rather than MDA in areas hypoendemic for onchocerciasis with known or suspected loiasis [12]. Hypoendemic areas that are non-endemic for loiasis were assumed to start MDA by 2023 with population coverage levels as reported by Kim *et al.* [8]. The underlying assumption is that *L. loa*-free hypoendemic areas will start additional mapping of onchocerciasis-endemic areas earlier than loiasis-endemic areas and will be able to start MDA more rapidly due to absence of implementation obstacles related to loiasis.

For APOC projects with a history of civil war (e.g. South Sudan from 1982 to 2005 and from 2013 to 2018; CAR from 2004 to 2007 and from 2012 to present), the treatment history was defined as follows. We assume that the impact of civil war on treatments before 2013 has been accounted for in the CDTI database through 2013 [13]. For MDA coverage of South Sudan and CAR since 2013, we consulted the ESPEN portal [10]. When the MDA coverage was unknown or not reported (mostly between 2013 and 2016), we assumed no MDA was provided. The mean coverage per region (i.e. APOC project, first administrative level) was calculated by averaging reported coverages over implementation units (IU, second administrative level of the countries) in the region, discarding reported coverages of >100%. [14]

The treatment history in Liberia, which was not included in the CDTi database by Kim *et al.* [8] and experienced civil war between 1989 to 2003, was characterised as follows. The ESPEN portal only started recording MDA coverages in Liberia since 2015. ESPEN did report cumulative MDA rounds in Liberia ranging between three to 11 rounds by 2013 (meaning that MDA likely started between 2002 and 2010 in most IUs) [10]. Due to civil war, we assume that country-wide MDA programmes started in 2004 with an average therapeutic coverage of 60%, which increased to 70% between 2007 and 2009 and to 82% since 2010. These assumptions on therapeutic coverage over time are based on data from the Ministry of Health in Liberia [15]. Liberia was severely affected by an outbreak of Ebola Virus Disease (EVD) between 2014 and 2015 [16]. ESPEN reports a country-wide therapeutic coverage of 49% in 2015, so we adapted this coverage also to 2014 to account for the EVD outbreak.

We applied the reported MDA epidemiological coverages between 2015 and 2019, as reported by ESPEN. MDA coverage of ivermectin distribution was averaged, as described above. We assumed that all NTD programmes were suspended in 2020 due to the COVID-19 pandemic, including all MDA activities. We further assume that the MDA coverage for 2021 to 2030 remains on average stable similar to the reported coverage of 2019. REMO surveys estimated that the population at risk for onchocerciasis in 1999 was 1.1 million [15], and this number was used to estimate the population at risk in 1995 using the UN population division growth factor [9].

In addition, in the current study we also consider that vector control was systematically implemented on the island of Bioko (Equatorial Guinea) [17,18]. In Uganda, an onchocerciasis elimination policy was launched in 2007 with the objective to eliminate onchocerciasis through bi-annual treatment with ivermectin using MDA and/or vector control/elimination [19,20]Most endemic districts in Uganda were henceforth treated bi-annually with ivermectin MDA since 2007. For APOC-projects Phases I to IV and P5Uganda, we assumed bi-annual MDA since 2007. As there were some remaining endemic foci that started MDA bi-annually at a later timepoint, we assumed that bi-annual MDA started in 2012 for Phase V. In addition, Uganda applied focal vector control over its various APOC projects (i.e. temephos river larvaciding, ground larvaciding, and trapping crabs)[20–22]. As result, vector control was applied in various endemic foci in Uganda through monthly or two-monthly cycles during different time periods for different durations (many foci applied vector control for a combined total duration of two or three years). Several vector control efforts were already initiated in 1994/1995. We account for local vector control in Uganda by simulating three continuous years of vector control during the initial years of vector control between 1994 and 1997. In many foci in Uganda, transmission of onchocerciasis has been interrupted and in some foci the disease has been eliminated [20–25].

A similar MDA database was also used to evaluate the impact of MDA on the number of onchocerciasis-loiasis co-infections in sub-Saharan Africa [4], but we made some further improvements accounting for security issues due to civil war, COVID-19, bi-annual MDA, and vector control. However, the MDA start year for several APOC projects used in this study deviates from the earlier published database [13]. See **S4 Text** for the APOC treatment database, used to simulate the impact of MDA and vector control on the prevalence of *O. volvulus* infection and onchocercal morbidity within each APOC project.

3. Mathematical modelling

3.1 Model background

We used the mathematical model ONCHOSIM to predict trends over time in the prevalence of infection and a wide range of clinical manifestations due to onchocerciasis. ONCHOSIM is a well-established mathematical model for simulating transmission and control of onchocerciasis in a dynamic population [26]. A detailed formal description of ONCHOSIM has been published elsewhere [27]. As previously, individual host participation in preventive chemotherapy was assumed to be a mix of random and systematic participation (some people are more inclined to participate than others). Ivermectin is assumed to clear 100% of the mf from the host, and to permanently reduce the capacity of female worms to produce mf by, on average, 34.9% [25]. We further assumed that treatment temporarily stops mf production by adult female worms altogether; mf production then gradually recovers over a period of 11 months on average. As in previous modelling exercises [26,28,29], we assumed no effect of ivermectin on prepatent worms.

3.2 Generic disease module

A new model extension for simulating morbidity due to onchocerciasis in ONCHOSIM (WORMSIM version 2.76) was recently developed and quantified, and is described elsewhere [27]. In short, the disease module allows simultaneous simulation of multiple subtypes of clinical manifestations (i.e. severe itch, reactive skin disease, palpable nodules, hanging groin, atrophy (<50 years of age), mild and severe depigmentation, and vision loss). The module allows conditions to be reversible or irreversible, and/or to be part of a continuum of manifestations (i.e. depigmentation: mild and severe depigmentation; vision loss: visual impairment and blindness), taking account of excess mortality due to blindness.

Within the new morbidity module, clinical manifestations due to *O. volvulus* infection are assumed to be caused by the death of mf triggering inflammatory reactions (e.g. by the release of *Wolbachia*) (skin and eye manifestations), or presence of adult female worms (palpable nodules). For each individual, the morbidity module keeps track of a "tissue damage" counter, which increases as a function of mf death or presence of adult female worms. The process of damage accrual is modelled in terms of three parameters per clinical manifestation: a damage threshold for the minimum amount of tissue damage associated with presence of the symptom, a regression rate for healing of tissue damage, and the level of inter-individual variation in susceptibility to tissue damage development. Together, these three parameters determine the shape of the community-level association between

prevalence of infection and a specific condition or continuum of clinical manifestations, as well as age patterns in morbidity at given levels of infection in the community. More information about the biological assumptions and implementation of these disease parameters in the model can be found elsewhere [27].

The pre-control association between the age-patterns in prevalence of each subtype of onchocercal skin disease (OSD) was quantified using data from a large multi-country dataset originating from forest areas [27]. Palpable nodules, severe itch and reactive skin disease (RSD) were considered to be acute, reversible clinical manifestations where regression of symptoms can occur after ivermectin treatment. The speed of reversion was calibrated using longitudinal trends in the prevalence of infection and morbidity after initiation of MDA [30,31]. The pre-control prevalence of onchocercal eye disease (OED) was quantified using various datasets on the community-level prevalence of infection and morbidity from forest and savanna areas [32–39]. Both stages of vision loss (visual impairment + blindness) were considered to be irreversible. More details on parameter quantifications for morbidity can be found elsewhere [27].

3.3 Simulations per APOC project

We divided each APOC project into six endemicity categories (**Tables A** and **B**) and for each category calculated the mean mf prevalence across pixels [6]. Next, in ONCHOSIM we calibrated the parameter for relative biting rate (rbr) to reproduce the mean pre-control mf prevalence in each project and endemicity category. As in previous ONCHOSIM modelling studies [8,26,29,40], exposure heterogeneity was set at k = 3.5. The rbr parameter was calibrated by simulating pre-control mf prevalences (all ages) for a grid of rbr values with steps of 0.01 (**Fig A**). Then, for each project and endemicity category, we mapped the mean pixel-level mf prevalence to a value for rbr, using linear interpolation. The corresponding rbr values were used in further simulations for the effect of MDA on infection and morbidity.

S3 Text provides an overview of the history of control and assumptions about future MDA used in the simulations. We assumed that vector control reduced vector abundance by 97.5%. Due to the absence of pre-control pixel-level data from the island of Bioko [6], based on literature we assumed that the overall crude pre-control mf prevalence on the island was 75.2% [41]. To reproduce this very high mean pre-control mf prevalence, we assumed that the island population was distributed

over the two highest endemicity categories (with average mf prevalence of 68.5% and 79.1%, respectively) at a ratio of 25:43 such that average prevalence over the whole population was 75.2%.

Fig A. Association between relative biting rate (rbr, x-axis) and the pre-control mf prevalence (all ages, y-axis) as predicted by ONCHOSIM. The level of exposure heterogeneity was set at k = 3.5.

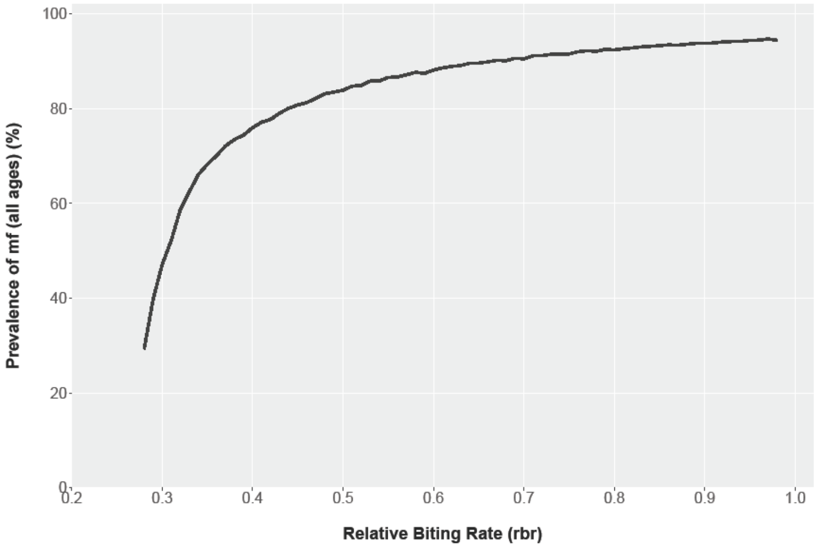

As ONCHOSIM cannot simulate stable infection with a community mf prevalence of below ~30% (these simulations result in spontaneous fade-out of transmission), we calculated the manifestations of disease prevalence in hypoendemic areas (endemicity level 1) as a ratio of the model-predicted morbidity prevalence in mesoendemic areas (endemicity level 2) (**Table B**), as previously done [40]. To assess which ratio in morbidity prevalence between mesoendemic versus hypoendemic areas would correspond to what is reported in the field, we performed a meta-analysis of published data on the prevalence of various subtypes of skin manifestations in hypoendemic versus mesoendemic areas [42,43]. For this purpose, we classified the different subtypes of skin manifestations in three groups corresponding to similar levels of acuteness and reversibility: (1) acute, reversible conditions (i.e. RSD and severe itch); (2) palpable nodules (i.e. slowly reversible); and (3) chronic, irreversible conditions (i.e. hanging groin, atrophy, depigmentation). In the meta-analysis, we estimated the relative difference in prevalence of aforementioned three groups of skin manifestations (i.e. using a log link function), allowing for random effects to capture heterogeneity between the two studies as well as between individual types of skin manifestations within each of the three groups. The results of the meta-analysis are visualised in **Fig B**. Trends in mf prevalence in hypoendemic areas were

derived by scaling trends for mesoendemic areas by the same amount as acute, reversible conditions, as these two metrics follow each other closely over time [27].

For six APOC projects (i.e. NY Cuanza Norte in Angola, Jigawa and Zamfara in Nigeria, Sudan and Sudan Abu Hamed in Sudan, Phase 2 in Uganda), the total population of the APOC project lived in hypoendemic areas and thus would result in spontaneous fading out of transmission in the model. To correct for this, we manually set an mf prevalence in endemicity level 2 (mesoendemic areas) corresponding to the average mf prevalence of all endemicity level 2 APOC projects combined (here: a mean mf prevalence of 39.4%). In this way, ONCHOSIM would be able to make predictions about the infection and morbidity prevalence within these APOC projects for mesoendemic areas (level 2), and then we could apply the ratio as provided in **Fig B** to estimate the prevalence of infection and morbidity in hypoendemic areas as opposed to mesoendemic areas. The model-predicted prevalence in mesoendemic areas (level 2) for these six APOC projects was further disregarded.

Fig B. Results of the meta-analysis of published data [42,43] on the prevalence of OSD in hypoendemic versus mesoendemic areas. The risk ratio (RR) represents the factor that should be multiplied by the morbidity prevalence in mesoendemic areas for each corresponding clinical manifestation to calculate the morbidity prevalence in hypoendemic areas.

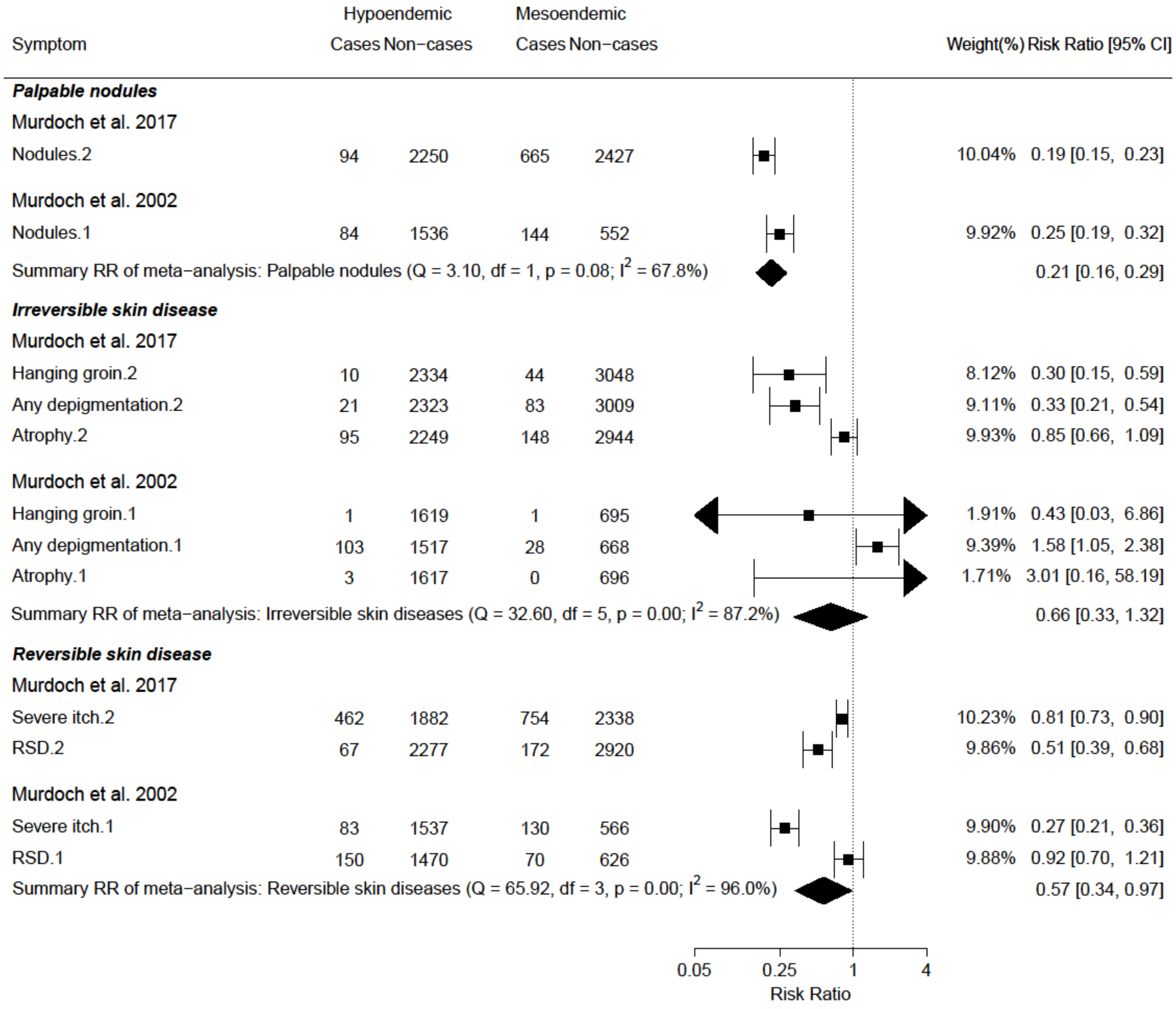

Note: There was some deviation in the clinical definition of severe itch between the Murchoch *et al* 2017 and 2002 studies. In Murdoch *et al* 2017, itching was defined as any itch with clinically normal skin (i.e. no evidence of onchocercal skin disease, nor any other itchy skin disease) [42]. In Murdoch *et al* 2002, itching was defined as troublesome itching with insomnia to try to exclude itching from other causes than onchocerciasis [43].

4. Disease burden calculation

In this section, we describe how the disease burden of onchocerciasis was calculated in terms of disability-adjusted life years (DALYs), which are defined as the sum of Years Lived with Disability (YLDs) and Years of Life Lost (YLLs) [44,45]. DALYs measure the time lost due to the effects of a condition in terms of (1) the time spent disabled by a condition (YLDs), weighted by the severity of the condition; and (2) time lost due to premature mortality by the condition (YLLs). One DALY represents one year of healthy life lost [44,45].

4.1 Years Lived with Disability

YLDs were calculated by multiplying the model-predicted number of prevalent cases with symptoms with a weighted severity level of the condition. These so-called disability weights have been developed for a very wide range of conditions as part of the Global Burden of Disease (GBD) studies [46,47]. The disability weights are estimated on the scoring of the severity of a health state by a participant assessed through face-to-face, telephone and online surveys from nine countries across all continents worldwide[44,48]. Although these is some variability in the scoring of disability weights for vision loss and blindness between respondents due to various factors [49], we have used the disability weights for visual impairment and blindness as most recently published by the GBD [44]. As these conditions were designed to be generic and not necessarily specific to any particular disease, we mapped each of the symptoms considered in this study to a condition associated with a disability weight in the GBD project. For OED, we adopted disability weights for functional vision loss directly from GBD (**Table C**). For OSD, we used a set of disability weights that were previously compiled by the consulting experts on onchocerciasis for the GBD 2010 study (which included co-authors Murdoch, Stolk, and Coffeng). This previously developed scheme considered three general severity levels of OSD (*OSD level 1*: slight, visible physical deformity that is sometimes sore or itchy, *OSD level 2*: visible physical deformity that is sore and itchy, *OSD level 3*: obvious physical deformity that is very painful and itchy), each of which could be associated with itch or not. Where previously, each of aforementioned severity levels encompassed a suit of OSD types, for our current analysis, we mapped each specific subtype of OSD to one of the severity levels. The disability weights and lay descriptions for each of the OSD types are shown in **Table C**.

Table C. Disability weights and lay descriptions for each clinical manifestation attributable to onchocerciasis. Disability weights are based on a previously published multi-country study by Salomon *et al.* [44].

Clinical manifestations	Assigned disability weight (95%CI)	Lay description in GBD for the assignment of disability weight
**Onchocercal skin disease (OSD)**		
Severe itch	0.188	*Disfigurement with itch or pain: level 2*. Person has a visible physical deformity that is sore and itchy. Other people stare and comment, which causes the person to worry. The person has trouble sleeping and concentrating.
Reactive skin disease (RSD)	0.048	*Disfigurement: levels 1 and 2*. See footnote.*
Palpable nodules	0.011	*Disfigurement: level 1*. Person has a slight, visible physical deformity that others notice, which causes some worry and discomfort.
Depigmentation		
Mild (partial)	0.011	*Disfigurement: level 1*. Person has a slight, visible physical deformity that others notice, which causes some worry and discomfort.
Severe (complete)	0.067	*Disfigurement: level 2*. Person has a visible physical deformity that causes others to stare and comment. As a result, the person is worried and has trouble sleeping and concentrating.
Atrophy	0.011	*Disfigurement: level 1*. See above
Hanging groin	0.405	*Disfigurement: level 3*. Person has an obvious physical deformity that makes others uncomfortable, which causes the person to avoid social contact, feel worried, sleep poorly, and think about suicide.
**Onchocercal eye disease (OED)**		
Visual impairment	0.031	*Distance vision: moderate impairment*. Person has vision problems that make it difficult to recognize faces or objects across a room.
Blindness	0.187	*Distance vision: blindness*. Person is completely blind, which causes great difficulty in some daily activities, worry and anxiety, and great difficulty going outside the home without assistance.

* RSD was defined as the presence of acute papular onchodermatitis (APOD), chronic papular onchodermatitis (CPOD), and/or lichenified onchodermatitis (LOD). To account for the resulting mix of severities of reactive skin conditions, we calculated an average disability weight, weighted by the prevalence of the three conditions in the multi-country dataset (APOD: 7.44%, CPOD: 12.89%, LOD: 1.23%) [43]. The three disability weights used for APOC, CPOD, and LOD were based on previous discussion with experts (dr. Michele Murdoch, professor Roderick Hay) for estimates by the Global Burden of Disease. APOD was assigned the disability weight for *disfigurement: level 1* and CPOD and LOD were assigned the disability weight for *disfigurement: level 2*.

Concurrence correction of disability weights

To correct YLD estimates for potential concurrence of multiple symptoms in the same individual (which can hypothetically lead to the sum of disability weights for concurring symptoms to exceed 1.0), we apply a multiplicative correction [50] to avoid overestimation of YLDs due to onchocerciasis [51]. Previous work has shown that there was considerable overlap of onchocercal morbidity within

individuals from a hyperendemic rainforest area of Cameroon, i.e. predominantly the occurrence of RSD and severe itch [50]. Different subtypes of OSD may lead to similar expressions of disability, e.g. stigmatisation (such as for depigmentation, hanging groin, atrophy, RSD) or low self-esteem due to skin disfigurement (e.g. RSD, open wounds due to scratching as a result of severe itching) [52]. It has therefore already been suggested by others that any overlap between clinical manifestations due to the same disease should be taken into account in burden estimates [50,53–55]. We choose to add up DALY estimates for OED and OSD without concurrence correction based on the notion that the two groups of symptoms involve completely different mechanisms of imposing a burden on an individual [50]. Any resulting potential overestimation of YLD will be limited because OED is relative rare compared to OSD (i.e. the concurrence correction would only affect a small number of people).

As such, we used a multiplicative approach in the estimation of disability weights to correct for non-random overlap of subtypes of OSD in the calculation of disease burden due to onchocerciasis. To do this, we first simulated all possible combinations of subtypes of OSD using ONCHOSIM, after which we obtained the prevalence of condition A **and / or** B (A|B), A|C, B|C, and A|B|C (here an example of three conditions that potentially concur). We then calculated the number of cases by multiplying the predicted prevalence with the population at risk. We could then calculate the number of cases with condition A **and** B (A⋔B), A⋔C, B⋔C, and A⋔B⋔C (see **Fig C**). For example, condition A⋔B was calculated by:

$$\text{Condition A}\pitchfork\text{B} = \left(\text{condition A(unc)} + \text{condition B(unc)}\right) - \text{condition A|B}$$

Where:
Condition A⋔B = Persons with both conditions A and condition B
Condition A*(unc)* = Persons with at least condition A, and potentially also condition B and/or C.
Condition B*(unc)* = Persons with at least condition B, and potentially also condition A and/or C.
Condition A|B = Persons with condition A and/or B.

In order to calculate the disability weight for each of the combinations of subtypes of OSD possible, we used the disability weights as assigned to each clinical manifestation (**Table C**) and applied the multiplicative model [53,54], using the following formula (here example of two concurrent conditions):

$$\mathrm{DwA{\cap}B} = 1 - (1 - \mathrm{DwA}) * (1 - DwB)$$

Where:

$Dw_{A\cap B}$ = The disability weight of the combined presence of conditions A and B ($A\cap B$).

Dw_A = The disability weight of condition A as provided in **Table C**.

Dw_B = The disability weight of condition B as provided in **Table C**.

The burden was then calculated by multiplying the corrected disability weight for each possible co-morbidity with the number of cases of the respective concurring conditions (here an example of two concurrent conditions):

$$\mathrm{Burden}(A\cap B) = Dw_{A\cap B} * \text{ Cases condition } A\cap B$$

Where:

Burden ($A\cap B$) = the total burden of condition A and condition B, for which it is as yet unknown which proportion of the burden is attributable to condition A and which proportion is attributable to condition B.

As we were interested in the burden of each unique clinical manifestation separately, we calculated the relative proportion of the burden attributable to each condition (here example: $DALY_A$ and $DALY_B$). This was done using the following equation (here an example of two concurrent conditions):

Relative proportion of YLD_A out of the $YLD_{A\cap B}$ = $YLD_{A\cap B} * \frac{\mathrm{DwA}}{\mathrm{DwA + DwB}}$

and

Relative proportion of YLD_B out of the $YLD_{A\cap B}$ = $YLD_{A\cap B} * \frac{\mathrm{DwB}}{\mathrm{DwA + DwB}}$

The total DALYs for each clinical manifestation corrected for co-morbidity was then calculated by summing for each possible co-morbidity the relative proportion of DALYs due to that subtype of OSD. Based on a theoretical maximum correction approach in case of maximum non-random concurrence of the six skin manifestations (i.e. severe itch, RSD, depigmentation, atrophy, hanging groin, palpable nodules), we calculated a reduction of 6.7% in total YLDs for OSD as compared to the uncorrected YLDs. To avoid the more than ten-fold increase in number of simulations required to perform the concurrence for each project and endemicity category (i.e. to simulate all symptom combinations $A\cap B\cap$ ...), and yet be conservative, we simply applied the aforementioned maximum concurrence correction for OSD of 6.7% to all projects and endemicity categories.

Fig C. Venn diagram representing how the total burden of multiple clinical manifestations is calculated, correcting for concurrency of symptoms using a multiplicative model. Here we used an example of three clinical manifestations only. Here, the total number of YLD would be 57.3 when not accounting for concurrence (i.e. calculating the burden for each symptom separately and then summing up), and is 55.9 (-2.3%) when correcting for concurrence with the multiplicative model.

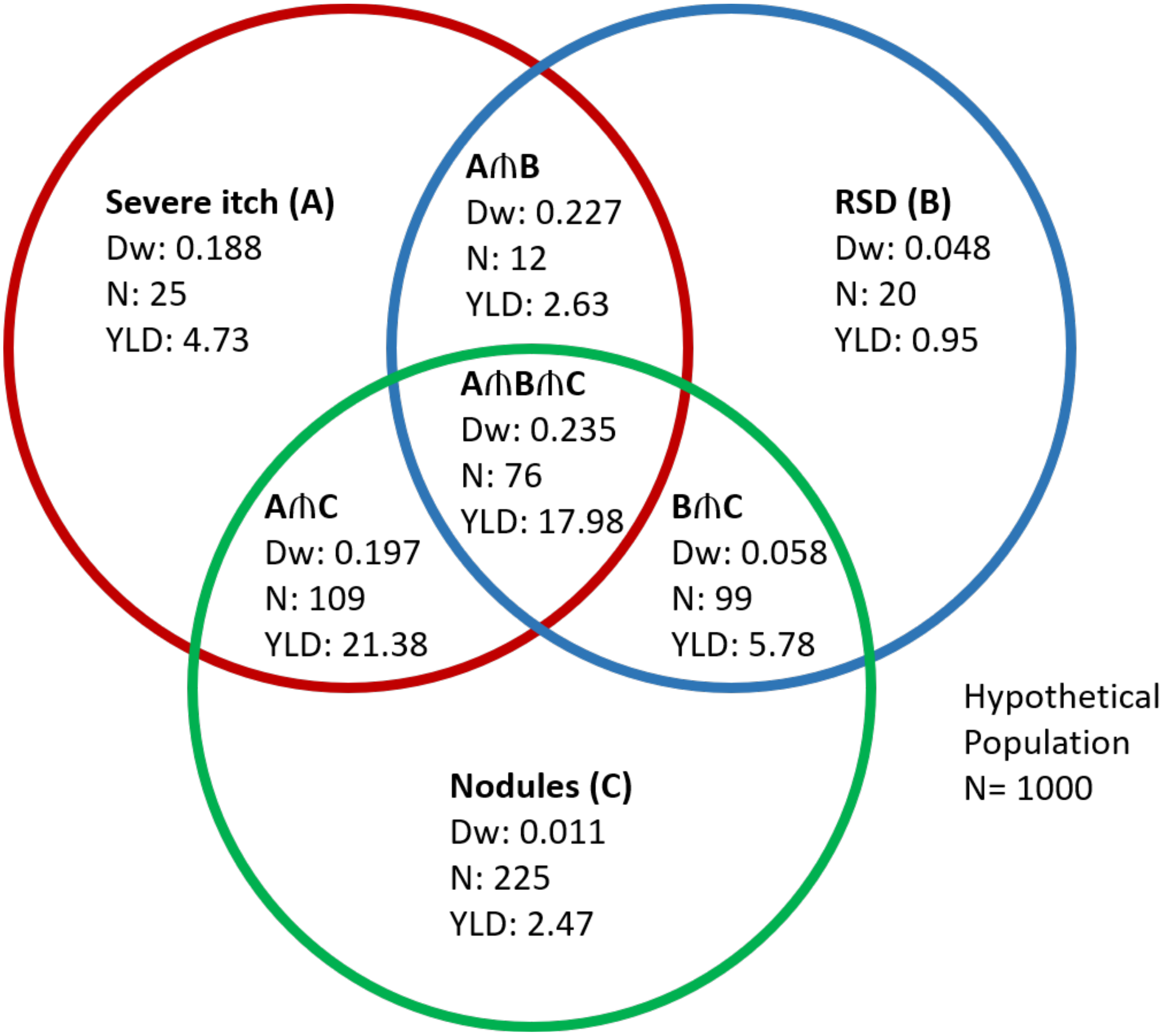

4.2 Years of Life Lost

The YLL measure captures the years of life lost due to premature mortality compared to the remaining life expectancy based on a counterfactual life table for a person of the same age but without the condition [56]. Of all the clinical conditions considered in this analysis, blindness is the only condition that has been associated with excess mortality [57,58]. We therefore calculated the YLLs for blindness only, stratifying by bioclimate (savanna vs forest areas) and the six endemicity levels.

Because ONCHOSIM itself does not provide output on life years lost or moment of death due to excess mortality, we estimated YLLs from ONCHOSIM output on prevalence of conditions using Sullivan life table methodology.

The counterfactual life table for individuals unaffected by onchocercal blindness was derived from the age distribution in sub-Saharan Africa as estimated by the UN Population Division for the year 2000 [59]. This life table was also used to simulate human demography in ONCHOSIM (**Fig D**). For a life table of individuals who are affected by onchocercal blindness, we calibrated age-specific excess mortality rates such that the life table reproduced the reduction in life-expectancy for blind individuals as simulated in ONCHOSIM (i.e. a reduction of 50%). As the average age of onset of onchocercal blindness depends on the endemicity (more highly endemic means younger average age of onset) and pathogenicity of the parasite strain (forest vs. savanna bioclimate), the number of YLLs per prevalent case of blindness was calculated by age, endemicity category, and bioclimate. **Table D** provides a summary of the total number of YLLs per endemicity category and bioclimate.

Fig D. Human cumulative survival probability (y-axis) in ONCHOSIM by age (x-axis).

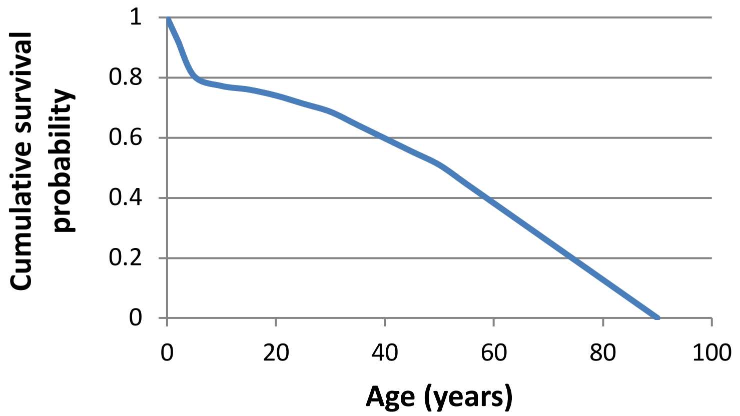

Table D. Estimated absolute number of pre-control Years of Life Lost (YLL) due to blindness, stratified by onchocerciasis endemicity levels and bioclimate.

Onchocerciasis endemicity levels	Cut-offs for prevalence of skin microfilariae in the general population (all ages)	Population size (millions)	YLL per prevalent case of blindness*	
			Forest	Savanna
Hypoendemic	≥ 1% - <35%	49.3	4.327	6.631
Mesoendemic	≥ 35% - <47%	13.3	5.632	6.870
	≥ 47% - <60%	8.7	6.173	7.298
Hyperendemic	≥ 60% - <66%	2.9	6.265	7.766
	≥ 66% - <75%	3.1	6.721	8.243
Very hyperendemic	≥ 75% - 100%	1.6	7.287	8.774

* As ONCHOSIM does not produce output on the number of new cases but only prevalent cases of blindness, years of life lost was calculated and expressed per prevalent case. These estimates are should be considered approximate as they are based on Sullivan life table analysis comparing two cohorts of blind and non-blind people in an equilibrium situation.

5. Sensitivity analysis

We performed univariate sensitivity analyses to assess the impact of biological and programmatic assumptions used in our analysis on the estimated number of cases with skin mf and onchocercal morbidity by 2030. An overview of the assumptions (and alternatives) assessed in these sensitivity analyses is presented in **Table E**.

Fig E-G present the estimated number of cases with mf-infection and morbidity by 2030 taking account of alternative assumptions as compared to baseline assumptions. The predicted number of cases with infection (mf-positivity and palpable nodules) by 2030 (**Fig E-F**) will increase considerably if MDA coverage is systematically being over-reported. We predict between 2,534 thousand and 6,375 thousand more mf-infected cases by 2030 in case of a 10% point or 20% point over-reporting of MDA, respectively, over the full duration of the MDA provided, and between 706.8 thousand and 1,894 thousand more cases respectively with palpable nodules. MDA over-reporting impacts the estimated number of cases with OED by 2030 to a lesser extent, as the development of clinical eye damage is much more progressive, yet we still predict between 41.2 thousand and 88.5 thousand more OED cases by 2030.

Table E. Biological and programmatic parameters assessed in the sensitivity analysis.

Parameter	Value in main analysis	Values in sensitivity analysis	
Fraction of the population permanently excluded from MDA	0	1%	5%
Reduction in remaining life expectancy at onset of blindness	50%	40%	60%
Regression rate of eye damage	0	0.01*	-
Scaling factor for morbidity prevalence in hypoendemic areas relative to mesoendemic areas#			
Mf infection (all ages)	0.57	0.33	
Worm infection	0.57	0.33	
Acute conditions (RSD and severe itch)	0.57	0.33	
Palpable nodules	0.21	0.33	
Chronic conditions (HG, atrophy, DPM, vision loss)	0.66	0.33	
Over-reporting of MDA coverage	None	10 percentage points	20 percentage points
Start of MDA in hypoendemic areas without MDA up to 2017	*L. loa*-endemic areas: roll-out in 2025;<br>*L. loa* non-endemic areas: roll-out in 2023.	*L. loa*-endemic areas: roll-out in 2024;<br>*L. loa* non-endemic areas: roll-out in 2022	*L. loa*-endemic areas: roll-out in 2026;<br>*L. loa* non-endemic areas: roll-out in 2024
Impact on MDA due to COVID-19 pandemic	No MDA provided in 2020 (one or two missed rounds)	MDA provided as usual	

* In the sensitivity analysis we assume that blindness is irreversible, but as long as an individual is not (yet) blind, visual impairment is reversible. The number of 0.01 is the fraction of "eye damage" that an individual recovers from each month, such that in absence of new damage, the damage declines exponentially as a function of the regression rate ρ (i.e. a reduction in total damage of $1-(1-\rho)^t$). This means that for $\rho = 0.01$, after one year 11% of damage would "heal", and after 5 years 45% of the damage would heal (N.B. in absence of new damage due to ongoing infection).

# Factors applied in the sensitivity analysis are derived from Coffeng *et al.* [40], who assumed that the prevalence of infection and morbidity in hypoendemic areas is 1/3 of that in mesoendemic areas.

On the other hand, fewer cases with infection (mf-positivity and palpable nodules) would be expected than currently estimated for 2030, if we assumed a different ratio in the prevalence of infection

between mesoendemic versus hypoendemic areas (**Fig E-F**): there may be up to 905.1 thousand and 252.4 thousand fewer mf-positive cases and cases with palpable nodules respectively by 2030. To a lesser extent, an earlier start of MDA in untreated areas would help to reduce the case estimates of mf-positivity and palpable nodules by 2030 with 797.4 thousand and 115.9 thousand fewer cases respectively than according to the baseline assumption.

Fig E. Results of the univariate sensitivity analysis with each of the alternative assumptions (coloured bars) for various parameters as compared to the baseline assumption (vertical black line) on the predicted number of cases with infection (mf-positivity, all ages) by the year 2030. Numbers of cases are presented in thousands.

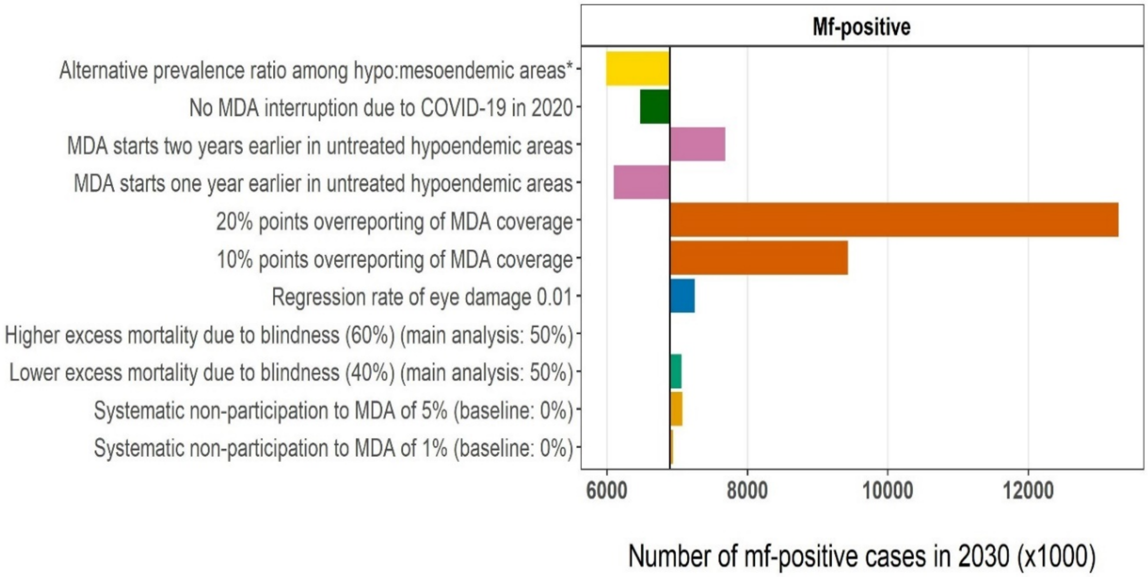

Caption: *Alternative ratio of morbidity prevalence in hypoendemic versus mesoendemic areas (see **Table E**).

The case estimates for OED by 2030 greatly depend on the assumptions applied to the possibility of regression of eye damage once symptoms have developed (**Fig G**). If we assume a 1% rate of damage regression that allows for healing of eye symptoms with a constant fraction of damage that is healed with every monthly time step, then we predict 358.4 thousand fewer cases with visual impairment or blindness. If we assume higher (60%) excess mortality due to blindness, we predict 26.1 thousand fewer OED cases. As many pre-control hypoendemic areas are in the savanna bioclimate, alternative assumptions on the prevalence ratio among hypoendemic versus mesoendemic areas impact the OED case numbers considerably (191.4 thousand fewer cases).

Fig F. Results of the univariate sensitivity analysis with each of the alternative assumptions (coloured bars) for various parameters as compared to the baseline assumption (vertical black line) for the predicted number of cases with palpable nodules by the year 2030. Numbers of cases are presented in thousands.

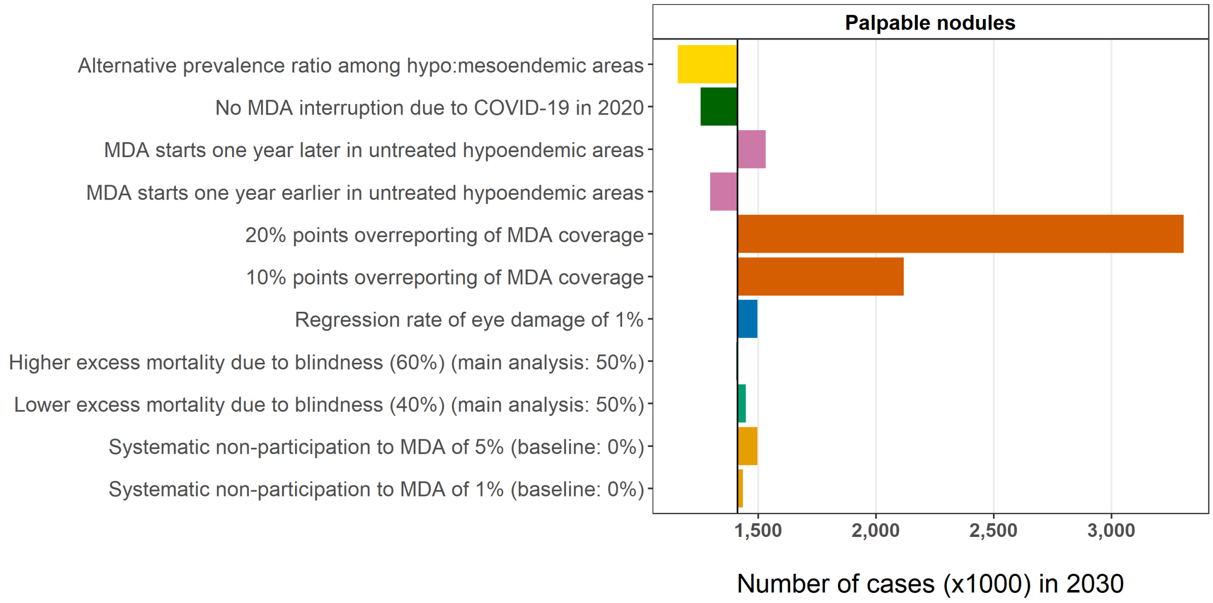

Fig G. Results of the univariate sensitivity analysis with each of the alternative assumptions (coloured bars) for various parameters as compared to the baseline assumption (vertical black line) for the predicted number of cases with onchocercal eye disease (OED) for forest and savanna areas by the year 2030. Numbers of cases are presented in thousands.

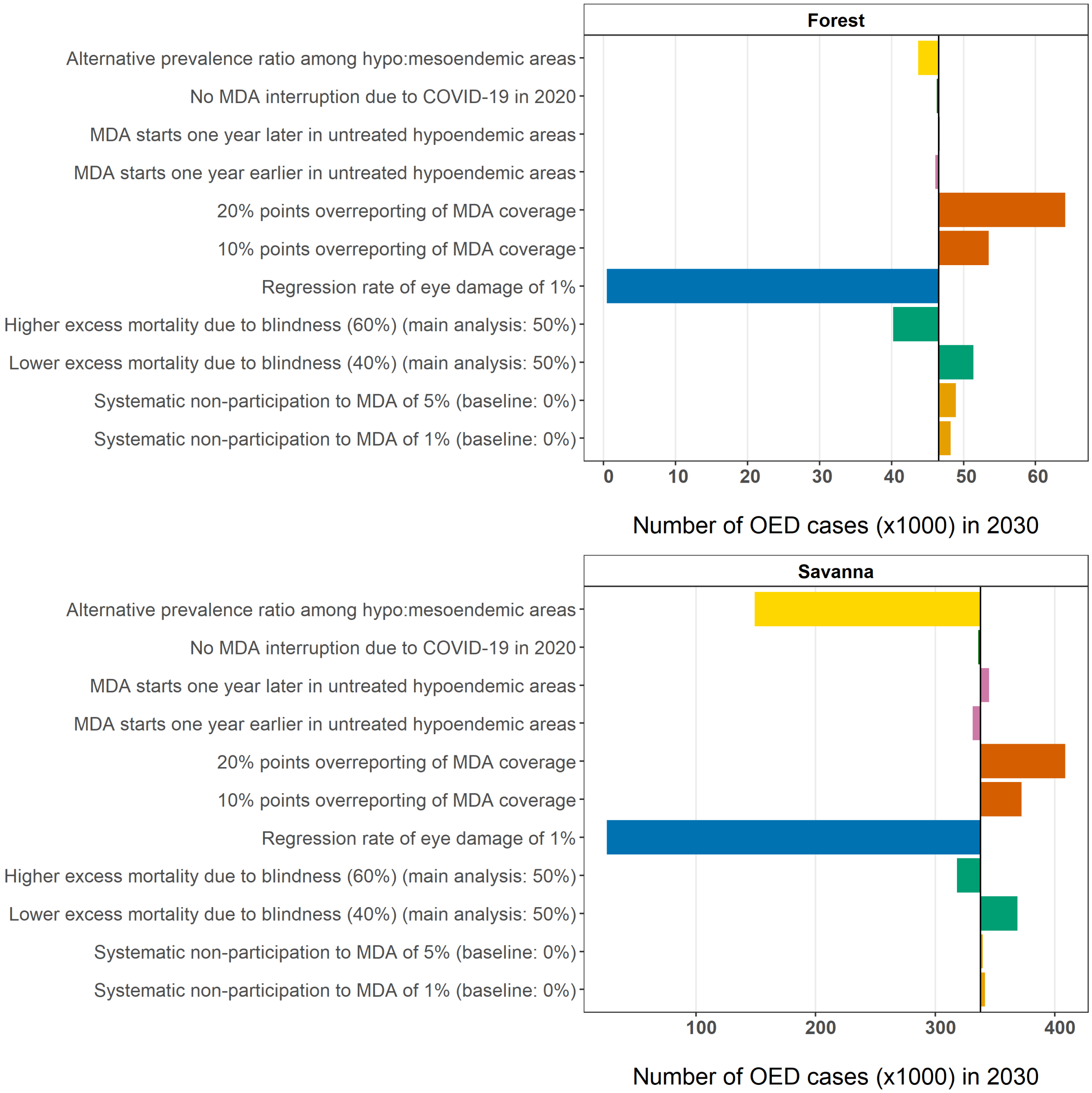

References

1. APOC. Guidelines for development of national plan and project proposal for sustainable community-directed treatment with ivermectin. African Programme for Onchocerciasis Control (APOC). 1999;APOC DOC.3.

2. WHO Monitoring Committee for the Elimination of Avoidable Blindness. Report of the first meeting. Geneva, 17–18 January 2006. Geneva, World Health Organization, 2006 (WHO/PBL/06.100).

3. Murdoch ME, Hay RJ, Mackenzie CD, Williams JF, Ghalib HW, Cousens S, et al. A clinical classification and grading system of the cutaneous changes in onchocerciasis. Br J Dermatol. 1993;129: 260–9.

4. Vinkeles Melchers NVS, Coffeng LE, Boussinesq M, Pedrique B, Pion SDS, Tekle AH, et al. Projected number of people with onchocerciasis-loiasis co-infection in Africa, 1995 to 2025. Clin Infect Dis. 2019;ciz647: 1–9.

5. Prost A, Hervouet J, Thylefors B. The degrees of endemicity of onchocerciasis. Bull World Health Organ. 1979;57: 655–662.

6. Zouré H, Noma M, Tekle AH, Amazigo UV, Diggle PJ, Giorgi E, et al. The geographic distribution of onchocerciasis in the 20 participating countries of the African Programme for Onchocerciasis Control: (2) pre-control endemicity levels and estimated number infected. Parasit vectors. 2014;7.

7. Noma M, Nwoke BEB, Nutall I, Tambala PA, Enyong P, Namsenmo A, et al. Rapid epidemiological mapping of onchocerciasis (REMO): its application by the African Programme for Onchocerciasis Control (APOC). Trop Med Parasitol. 2002;96 Suppl 1: S29-39.

8. Kim YE, Remme JHF, Steinmann P, Stolk WA, Roungou J-B, Tediosi F. Control, elimination, and eradication of river blindness: scenarios, timelines, and ivermectin treatment needs in Africa. PLoS Negl Trop Dis. 2015;9: e0003664.

9. United Nations, Department of Economic and Social Affairs PD (2020). Population growth rates. World Population Prospects: The 2020 Revision Available at: http://esa.un.org/wpp/Excel-Data/population.htm. (Accessed on: 10 March 2021).

10. ESPEN Portal. Onchocerciasis maps. Expanded Special Project for Elimination of Neglected Tropical Diseases (ESPEN). World Health Organization Regional Office for Africa. Available at: https://espen.afro.who.int/ (Assessed on: 11 May 2021).

11. World Health Organization. COVID-19: WHO Issues Interim Guidance for Implementation of NTD Programmes. Available at: https://www.who.int/neglected_diseases/news/COVID19-WHO-interim-guidance-implementation-NTD-programmes/en/ (Accessed on: 17 May 2021).

12. Mectizan-Taskforce. Recommendations for the treatment of Onchocerciasis with Mectizan® in areas co-endemic for Onchocerciasis and Loiasis. The Mectizan® Expert Committee/Technical Consultative Committee. 2004.

13. Kim YE, Remme JHF, Steinmann P, Stolk WA, Roungou JB, Tediosi F. Control, elimination, and eradication of river blindness: scenarios, timelines, and ivermectin treatment needs in Africa. PLoS Negl Trop Dis. 2015;9.

14. WHO-African Programme for Onchocerciasis Control (APOC). South Sudan Onchocerciasis Task Force (SSOTF). Annual NOTF secretariat 2014 technical report submitted to Technical Consultative Committee (TCC). 2015.

15. The Republic of Liberia, Ministry of Health. Master Plan for Neglected Tropical Diseases: 2016-2020. Available at: https://espen.afro.who.int/system/files/content/resources/LIBERIA_NTD_Master_Plan_2016_2020.pdf. (Accessed on: 15 June 2021).

16. Bogus J, Gankpala L, Fischer K, Krentel A, Weil GJ, Fischer PU, et al. Community attitudes toward mass drug administration for control and elimination of neglected tropical diseases after the 2014 outbreak of ebola virus disease in Lofa County, Liberia. Am. J. Trop. Med. Hyg. 2016;94: 497–503.

17. Herrador Z, Garcia B, Ncogo P, Perteguer MJ, Rubio JM, Rivas E, et al. Interruption of onchocerciasis transmission in Bioko Island: Accelerating the movement from control to elimination in Equatorial Guinea. PLoS Negl Trop Dis. 2018;12: e0006471.

18. Moya L, Herrador Z, Ta-Tang TH, Rubio JM, Perteguer MJ, Hernandez-González A, et al. Evidence for Suppression of Onchocerciasis Transmission in Bioko Island, Equatorial Guinea. PLoS Negl Trop Dis. 2016;10: e0004829.

19. National Onchocerciasis Control Program. Ministry of Health. Republic of Uganda. Available at: https://www.health.go.ug/programs/national-onchocerciasis-control-program/. (Accessed on: 11 May 2021).

20. Katabarwa MN, Lakwo T, Habomugisha P, Unnasch TR, Garms R, Hudson-Davis L, et al. After 70 years of fighting an age-old scourge, onchocerciasis in Uganda, the end is in sight. International Health. Oxford University Press; 2018. pp. i79–i88.

21. Lakwo TL, Garms R, Rubaale T, Katabarwa M, Walsh F, Habomugisha P, et al. The disappearance of onchocerciasis from the Itwara focus, western Uganda after elimination of the vector *Simulium neavei* and 19 years of annual ivermectin treatments. Acta Trop. 2013;126: 218–21.

22. Katabarwa M, Lakwo T, Habomugisha P, Agunyo S, Byamukama E, Oguttu D, et al. Transmission of *Onchocerca volvulus* by *Simulium neavei* in Mount Elgon focus of eastern Uganda has been interrupted. Am J Trop Med Hyg. 2014;90: 1159–1166.

23. Katabarwa MN, Walsh F, Habomugisha P, Lakwo TL, Agunyo S, Oguttu DW, et al. Transmission of onchocerciasis in Wadelai focus of northwestern Uganda has been interrupted and the disease eliminated. J Parasitol Res. 2012.

24. Katabarwa MN, Katamanywa J, Lakwo T, Habomugisha P, Byamukama E, Oguttu D, et al. The imaramagambo onchocerciasis focus in southwestern Uganda: Interruption of transmission after disappearance of the vector *simulium neavei* and its associated freshwater crabs. Am J Trop Med Hyg. 2016;95: 417–425.

25. Katabarwa MN, Habomugisha P, Khainza A, Oguttu DW, Byamukama E, Katamanywa J, et al. Historical elimination of onchocerciasis from victoria nile focus in Central Uganda verified using WHO criteria. Am J Trop Med Hyg. 2020;102: 1411–1416.

26. Plaisier A, van Oortmarssen G, Habbema J, Remme J, Alley E. ONCHOSIM: a model and computer simulation program for the transmission and control of onchocerciasis. Comput Methods Programs Biomed. 1990;31: 43–56.

27. Vinkeles Melchers NVS, Stolk WA, Murdoch ME, Pedrique B, Kloek M, Bakker R, et al. How does onchocerciasis-related skin and eye disease in Africa depend on cumulative exposure to infection and mass treatment? PLoS Negl Trop Dis. 2021;15: e0009489.

28. Coffeng LE, Stolk WA, Hoerauf A, Habbema D, Bakker R, Hopkins AD, et al. Elimination of African onchocerciasis: modeling the impact of increasing the frequency of ivermectin mass treatment. PloS one 2014;9: e115886.

29. Stolk WA, Walker M, Coffeng LE, Basáñez M-G, de Vlas SJ. Required duration of mass ivermectin treatment for onchocerciasis elimination in Africa: a comparative modelling analysis. Parasit Vectors 2015;8.

30. Ozoh GA, Murdoch ME, Bissek A-C, Hagan M, Ogbuagu K, Shamad M, et al. The African Programme for Onchocerciasis Control: impact on onchocercal skin disease. Trop Med Int Health 2011;16: 875–83.

31. Brieger WR, Awedoba AK, Eneanya CI, Hagan M, Ogbuagu KF, Okello DO, et al. The effects of ivermectin on onchocercal skin disease and severe itching: results of a multicentre trial. Trop Med Int Health 1998;3: 951–61.

32. Remme JHF. The Global Burden of Onchocerciasis in 1990. Geneva: World Health Organization. 2004.

33. Remme J, Dadzie KY, Rolland A, Thylefors B. Ocular onchocerciasis and intensity of infection in the community. I. West African savanna. Trop Med Parasitol. 1989;40: 340–7.

34. Resnikoff S, Pascolini D, Etya'ale D, Kocur I, Pararajasegaram R, Pokharel GP, et al. Global data on visual impairment in the year 2002. Bull World Health Organ. 2004;82: 844–51.

35. Kayembe DL, Kasonga DL, Kayembe PK, Mwanza J-CK, Boussinesq M. Profile of eye lesions and vision loss: a cross-sectional study in Lusambo, a forest-savanna area hyperendemic for onchocerciasis in the Democratic Republic of Congo. Trop Med Int Health. 2003;8: 83–9.

36. Brown R, Shannon R. Prevalence, intensity and ocular manifestations of *Onchocerca volvulus* infection in Dimbelenge, Zaire. Ann Soc Belg Med Trop. 1989;69: 137–42.

37. Henry MC, Maertens K. The onchocerciasis focus at Kinsuka/Kinshasa (Republic of Zaire) in 1985. II. Parasitological and clinical aspects. Ann Trop Med Parasitol. 1990;84: 493–502.

38. Whitworth JA, Gilbert CE, Mabey DM, Maude GH, Morgan D, Taylor DW. Effects of repeated doses of ivermectin on ocular onchocerciasis: community-based trial in Sierra Leone. Lancet 1991;338: 1100–3.

39. Whitworth JA, Gilbert CE, Mabey DM, Morgan D, Foster A. Visual loss in an onchocerciasis endemic community in Sierra Leone. Br J Ophthalmolx. 1993;77: 30–2.

40. Coffeng LE, Stolk WA, Zouré HGM, Veerman JL, Agblewonu KB, Murdoch ME, et al. African Programme for Onchocerciasis Control 1995-2015: model-estimated health impact and cost. PLoS Negl Trop Dis. 2013;7: e2032.

41. Mas J, Sima A, Untoria D, Post R, Limiñana C, Ncogo P, et al. Onchocerciasis and its control in Equatorial Guinea: 1909-1996. Parasitol. Res. 1996;56: 147–155.

42. Murdoch ME, Murdoch IE, Evans J, Yahaya H, Njepuome N, Cousens S, et al. Pre-control relationship of onchocercal skin disease with onchocercal infection in Guinea Savanna, Northern Nigeria. PLoS Negl Trop Dis. 2017;11: e0005489.

43. Murdoch ME, Asuzu MC, Hagan M, Makunde WH, Ngoumou P, Ogbuagu KF, et al. Onchocerciasis: the clinical and epidemiological burden of skin disease in Africa. Ann Trop Med Parasitol. 2002;96: 283–96.

44. Salomon JA, Haagsma JA, Davis A, de Noordhout CM, Polinder S, Havelaar AH, et al. Disability weights for the Global Burden of Disease 2013 study. Lancet Glob Health. 2015;3: e712–e723.

45. World Bank. World Development Report 1993 : Investing in Health. New York: Oxford University Press. License: CC BY 3.0 IGO. Available at: https://openknowledge.worldbank.org/handle/10986/5976. (Assessed on: 10 March 2020).

46. Vos T, Abajobir AA, Abate KH, Abbafati C, Abbas KM, Abd-Allah F, et al. Global, regional, and national incidence, prevalence, and years lived with disability for 328 diseases and injuries for 195 countries, 1990–2016: a systematic analysis for the Global Burden of Disease Study 2016. Lancet 2017;390: 1211–1259.

47. Murray CJ. Quantifying the burden of disease: the technical basis for disability-adjusted life years. Bull World Health Organ.1994;72: 429–45.

48. Salomon JA, Vos T, Hogan DR, Gagnon M, Naghavi M, Mokdad A, et al. Common values in assessing health outcomes from disease and injury: disability weights measurement study for the Global Burden of Disease Study 2010. Lancet. 2012;380: 2129–43.

49. Braithwaite T, Taylor H, Bourne R, Keeffe J, Pesudovs K. Does blindness count? Disability weights for vision loss. Clinical and Experimental Ophthalmology. Blackwell Publishing; 2017. pp. 217–220.

50. Coffeng LE, Fobi G, Ozoh G, Bissek AC, Nlatté BO, Enyong P, et al. Concurrence of dermatological and ophthalmological morbidity in onchocerciasis. Trans R Soc Trop Med Hyg. 2012;106: 243–51.

51. Murray CJ, Lopez AD. Progress and directions in refining the global burden of disease approach: a response to Williams. Health Econ. 2000;9: 69–82.

52. Alonso L, Murdoch M, Jofre-Bonet M. Psycho-social and economical evaluation of onchocerciasis: a literature review. Soc Med. 2009;4: 8–31.

53. Mathers CD, Iburg KM, Begg S. Adjusting for dependent comorbidity in the calculation of healthy life expectancy. Popul Health Metr. 2006;4:4.

54. van Baal PH, Hoeymans N, Hoogenveen RT, de Wit GA, Westert GP. Disability weights for comorbidity and their influence on Health-adjusted Life Expectancy. Popul Health Metr. 2006;4: 1.

55. Flanagan W, McIntosh CN, Le Petit C, Berthelot J-M. Deriving utility scores for co-morbid conditions: a test of the multiplicative model for combining individual condition scores. Popul Health Metr. 2006;4: 13.

56. Haidong, Abajobir AA, Abate KH, Abbafati C, Abbas KM, Abd-Allah F, et al. GBD 2016 Mortality Collaborators. Global, regional, and national under-5 mortality, adult mortality, age-specific mortality, and life expectancy, 1970-2016: a systematic analysis for the Global Burden of Disease Study 2016. Lancet. 2017;390: 1084–1150.

57. Prost A, Vaugelade J. Excess mortality among blind persons in the West African savannah zone. Bull World Health Organ. 1981;59: 773–6.

58. Pion SDS, Kamgno J, Demanga-Ngangue M, Boussinesq M. Excess mortality associated with blindness in the onchocerciasis focus of the Mbam Valley, Cameroon. Ann Trop Med Parasitol. 2002;96: 181–9.

59. UN (2013) World Population Prospects. 2012 population. Available at: http://esa.un.org/wpp/Excel- Data/population.htm. (Accessed on: 29 March 2021).
