# Supplementary material for: The burden of skin disease and eye disease due to onchocerciasis in countries formerly under the African Programme for Onchocerciasis Control mandate for 1990, 2020, and 2030
Source: PLoS Negl Trop Dis. 2021 Jul 26;15(7):e0009604. doi: 10.1371/journal.pntd.0009604 (PMC8312930; doi:10.1371/journal.pntd.0009604)
Supplement: S2 Text — (PDF) [file pntd.0009604.s002.pdf]

The burden of skin disease and eye disease due to onchocerciasis in Africa for 1990, 2020, and 2030

S2 Text:
Treatment history and assumptions per APOC project.

Natalie V.S. Vinkeles Melchers[1], Wilma A. Stolk[1], Welmoed van Loon[1,2], Belén Pedrique[3], Roel Bakker[1], Michele E. Murdoch[4], Sake J. de Vlas[1], Luc E. Coffeng[1]

1. Department of Public Health, Erasmus MC, University Medical Center Rotterdam, Rotterdam, The Netherlands.
2. Institute of Tropical Medicine and International Health, Charité - Universitätsmedizin Berlin, corporate member of Freie Universität Berlin, Humboldt-Universität zu Berlin, and Berlin Institute of Health, Berlin, Germany.
3. Drugs for Neglected Diseases initiative (DND*i*), Geneva, Switzerland
4. Department of Dermatology, Watford General Hospital, Watford, Hertfordshire, UK

* Correspondence to:

Luc E. Coffeng, Department of Public Health, Erasmus MC, University Medical Center Rotterdam, P.O. box 2040, 3000 CA Rotterdam, The Netherlands; l.coffeng@erasmusmc.nl

Alternative:
Natalie VS Vinkeles Melchers, Department of Public Health, Erasmus MC, University Medical Center Rotterdam, P.O. box 2040, 3000 CA Rotterdam, The Netherlands; n.vinkelesmelchers@erasmusmc.nl, Natalie.melchers@gmail.com; +31 614217276

Table F: Summary table of pre-control onchocerciasis endemicity, population at risk for 1990 and 2030, MDA treatment history by country and APOC-project. Abbreviation: PTS = Post-treatment surveillance.

			Pop. at risk (x1000)		MDA †					
Country	Project	Endem. levels**	1990	2030	start	cov. at start (%)	mean cov. (%)	No. rounds 1990-2030	Vector control	Remarks
Angola	Bengo	Meso	13	39	2010	75.0	70.8	20	No	
Angola	Benguela	Hypo	25	75	2012	62.7	59.4	18	No	
Angola	Cuanza Norte	Meso	13	38	2011	74.9	71.2	19	No	
Angola	Huila	Meso	119	352	2007	22.0	59.2	21	No	
Angola	Kuando Kubango	Meso	205	607	2007	65.0	68.9	22	No	
Angola	Lunda Norte	Hyper	153	453	2005	6.0	59.8	25	No	
Angola	Lunda sul	Meso	129	382	2005	15.0	60.4	25	No	
Angola	Moxico 1	Hyper	135	399	2007	22.0	58.8	23	No	
Angola	Namibe	Meso	18	53	2019	67.0	61.4	11	No	
Angola	NY Benguela	Hyper	57	168	2019	67.0	61.4	11	No	
Angola	NY Cuanza Norte	Meso	9	27	2019	67.0	61.4	11	No	
Angola	NY Huila	Meso	11	34	2019	67.0	61.4	11	No	
Angola	NY Lunda Norte	Meso	33	99	2019	67.0	61.4	11	No	
Angola	NY Moxico 1	Meso	172	510	2019	67.0	61.4	11	No	
Angola	P5Angola*	Hypo	131	388	2025	65.0	65.0	6	No	*Loa loa* coendemicity
Angola	Uige	Hyper	95	280	2019	67.0	61.4	11	No	
Angola	Zaire	Meso	7	21	2019	67.0	61.4	11	No	
Burundi	Bururi	Meso	220	652	2006	49.0	69.7	24	No	
Burundi	Cibitoke-Bubanza	Meso	526	1,557	2005	23.0	72.4	25	No	
Burundi	P5Burundi*	Hypo	415	1,228	2025	65.0	65.0	6	No	*Loa loa* coendemicity
Burundi	Rutana	Hypo	171	508	2006	51.0	71.1	24	No	
Cameroon	Adamaoua 1	Hyper	287	850	2004	47.0	67.9	26	No	
Cameroon	Adamaoua 2	Hyper	261	772	1999	10.0	65.8	31	No	
Cameroon	Centre 1	Hyper	265	785	2000	32.0	69.0	30	No	
Cameroon	Centre 2	Hyper	62	185	2000	13.0	64.1	29	No	
Cameroon	Centre 3	Hyper	201	596	1999	43.0	67.4	31	No	
Cameroon	East	Hyper	74	218	2005	33.0	74.7	25	No	
Cameroon	Far North	Meso	174	515	2005	57.0	74.5	25	No	
Cameroon	Littoral 1	Hyper	174	515	2006	31.0	63.1	24	No	
Cameroon	Littoral 2	Hyper	93	275	1999	38.0	67.2	31	No	

(continued)

Country	Project	Endem. levels**	Pop. at risk (x1000) 1990	2030	MDA † start	cov. at start (%)	mean cov. (%)	No. rounds 1990-2030	Vector control	Remarks
Cameroon	Northern	Hyper	390	1,155	1999	21.0	69.5	31	No	
Cameroon	Northwest	Hyper	507	1,501	2004	58.0	72.5	26	No	
Cameroon	P20Cameroon*	Hyper	93	276	2016	78.3	73.0	14	No	
Cameroon	P5Cameroon*	Hypo	929	2,751	2025	65.0	65.0	6	No	*Loa loa* coendemicity
Cameroon	South	Hyper	185	548	2005	32.0	70.7	25	No	
Cameroon	South West 1	Hyper	243	719	1999	68.0	68.8	31	No	
Cameroon	South West 2	Hyper	165	489	2001	37.0	74.6	29	No	
Cameroon	Western	Hyper	1,016	3,007	2001	19.0	72.9	29	No	
CAR	CAR region 3	Hyper	265	784	1999	73.0	62.5	28	No	Civil war between 2012-present
CAR	CAR region 4	Hyper	276	817	1999	73.0	61.5	28	No	Civil war between 2012-present
CAR	CAR region 5	Hyper	255	756	1999	73.0	58.9	26	No	Civil war between 2012-present
CAR	CAR region 6	Hyper	393	1,163	1999	73.0	60.3	27	No	Civil war between 2012-present
CAR	P20CAR*	Hyper	39	115	2019	80.4	73.7	11	No	Civil war between 2012-present
CAR	P5CAR*	Hypo	88	260	2025	65.0	65.0	6	No	Civil war between 2012-present; *Loa loa* coendemicity
Chad	Chad	Hyper	1,068	3,160	1998	40.0	72.2	32	No	
Chad	P5Chad*	Hypo	133	393	2025	65.0	65.0	6	No	*Loa loa* coendemicity
Chad	P5Chad propext*	Hypo	76	226	2025	65.0	65.0	6	No	*Loa loa* coendemicity
Congo	Congo 1	Hyper	487	1,441	2001	36.0	70.4	29	No	
Congo	P20Congo*	Hyper	21	63	2015	81.1	76.0	15	No	
Congo	P5Congo*	Hypo	286	846	2025	65.0	65.0	6	No	*Loa loa* coendemicity
DRC	Bandundu	Hyper	3	10	2003	12.0	70.7	26	No	
DRC	Bas-Congo Kinshasa	Hyper	803	2,378	2004	60.0	61.9	25	No	
DRC	Butembo-Beni	Hyper	499	1,478	2008	9.0	61.2	22	No	
DRC	Equateur-Kiri	Hyper	664	1,964	2006	19.0	69.9	24	No	
DRC	Ituri-Nord	Hyper	671	1,987	2008	28.0	75.3	22	No	
DRC	Ituri-Sud	Hyper	614	1,816	2012	71.9	68.1	18	No	
DRC	Kasai	Hyper	5,756	17,038	2001	8.0	61.9	29	No	
DRC	Kasongo	Hyper	721	2,134	2007	9.0	63.6	23	No	
DRC	Katanga-Nord	Hyper	335	992	2006	32.0	71.7	24	No	
DRC	Katanga-Sud	Hyper	371	1,098	2006	32.0	68.1	24	No	

(continued)

Country	Project	Endem. levels**	Pop. at risk (x1000) 1990	2030	MDA † start	cov. at start (%)	mean cov. (%)	No. rounds 1990-2030	Vector control	Remarks
DRC	Lualaba	Hyper	120	356	2006	57.0	74.3	24	No	
DRC	Lubutu	Hyper	179	529	2008	24.0	66.7	22	No	
DRC	Masisi-Walikale	Hyper	560	1,659	2009	31.0	67.7	21	No	
DRC	Mongala	Hyper	779	2,306	2006	8.0	68.8	24	No	
DRC	NY Katanga-Nord	Meso	243	720	2016	71.0	66.3	14	No	(Post) conflict
DRC	NY Lualaba	Hyper	526	1,557	2016	71.0	66.3	14	No	
DRC	NY Masisi-Walikale	Hyper	29	86	2016	71.9	67.1	14	No	(Post) conflict
DRC	NY Rutshuru-Ngoma	Meso	5	13	2013	71.9	67.9	17	No	
DRC	NY Sankuru	Hyper	251	743	2014	71.0	66.8	16	No	
DRC	NY Ueles	Hyper	85	253	2014	71.9	67.7	16	No	
DRC	P20DRC*	Hyper	1,259	3,726	2019	71.9	65.9	11	No	(Post) conflict
DRC	P5DRC*	Hypo	4,003	11,848	2025	65.0	65.0	6	No	(Post) conflict; *Loa loa* coendemicity
DRC	Rutshuru-Ngoma	Hyper	353	1,045	2006	40.0	65.2	23	No	
DRC	Sankuru	Hyper	571	1,690	2003	42.0	71.3	27	No	
DRC	Tshopo	Hyper	853	2,525	2003	17.0	54.0	25	No	
DRC	Tshuapa	Hyper	760	2,249	2006	4.0	57.2	24	No	
DRC	Ubangi-Nord	Hyper	428	1,267	2006	22.0	59.6	24	No	
DRC	Ubangi-Sud	Hyper	722	2,136	2006	10.0	60.8	24	No	
DRC	Ueles	Hyper	842	2,492	2002	20.0	65.5	27	No	
Eq. Guinea	Bioko	Hyper	59	176	1990	53.2	49.2	23	Yes	Vector control between 1999-2005; PTS since 2016 [1-3]‡
Eq. Guinea	P5EqGuinea*	Hypo	139	412	2025	65.0	65.0	6	No	*Loa loa* coendemicity
Ethiopia	Assosa	Meso	317	939	2019	79.0	72.4	22	No	Bi-annual MDA since 2019
Ethiopia	Bench-Maji	Hyper	420	1,242	2003	35.0	70.1	27	No	
Ethiopia	East Wellega	Meso	514	1,521	2004	39.0	72.5	25	No	
Ethiopia	Gambella	Hyper	61	182	2004	71.0	70.8	25	No	
Ethiopia	Horo Guduru	Meso	30	89	2019	79.0	72.4	11	No	
Ethiopia	Illubabor	Hyper	435	1,287	2004	77.0	76.9	26	No	
Ethiopia	Jimma	Meso	513	1,519	2004	73.0	78.7	26	No	
Ethiopia	Kaffa-Sheka	Hyper	729	2,157	2001	24.0	71.8	29	No	
Ethiopia	Kamashi	Hyper	271	802	2016	80.0	74.7	28	No	

(continued)

Country	Project	Endem. levels**	Pop. at risk (x1000) 1990	2030	MDA † start	cov. at start (%)	mean cov. (%)	No. rounds 1990-2030	Vector control	Remarks
Ethiopia	Metekel	Meso	93	275	2004	50.0	66.8	25	No	
Ethiopia	North Gondar	Meso	180	533	2003	37.0	68.3	27	No	
Ethiopia	NY East Wellega	Hyper	165	487	2016	80.0	74.7	14	No	
Ethiopia	NY West Wellega	Meso	167	495	2016	79.0	73.7	28	No	Bi-annual MDA since 2016
Ethiopia	P20Ethiopia*	Hyper	191	566	2019	80.0	73.3	11	No	
Ethiopia	P5Ethiopia*	Hypo	2,018	5,972	2023	65.0	65.0	8	No	
Ethiopia	West Shewa	Hypo	33	98	2023	65.0	65.0	8	No	
Ethiopia	West Wellega	Hyper	590	1,747	2004	20.0	72.7	25	No	
Gabon	P5Gabon*	Hypo	49	146	2025	65.0	65.0	6	No	*Loa loa* coendemicity
Liberia	All_Liberia	Meso	881	2,606	2004	60.0	66.3	26	No	Civil war between 1989-2003
Malawi	Malawi Extension	Meso	665	1,968	2000	4.0	69.4	30	No	
Malawi	Thyolo Mwanza	Meso	507	1,501	1997	13.0	69.1	33	No	
Mozamb.	P20Mozambique*	Meso	9	27	2019	70.0	64.2	11	No	
Mozamb.	P5Mozambique*	Hypo	27	80	2023	65.0	65.0	8	No	
Nigeria	Adamawa	Hyper	1,019	3,015	1999	47.0	72.9	31	No	
Nigeria	Akwa Ibom	Meso	17	52	2004	38.0	74.1	26	No	
Nigeria	Bauchi	Meso	1,044	3,089	1999	15.0	63.2	31	No	
Nigeria	Benue	Hyper	2,058	6,092	1999	11.0	62.9	31	No	
Nigeria	Borno	Meso	820	2,428	1999	48.0	72.1	31	No	
Nigeria	Cross River	Hyper	744	2,202	1998	52.0	75.1	32	No	
Nigeria	Edo Delta	Hyper	956	2,831	1999	74.0	75.6	31	No	
Nigeria	Ekiti	Meso	1,291	3,821	1999	36.0	68.7	31	No	
Nigeria	Enugu Anambra Ebony	Hyper	1,421	4,206	1999	63.0	76.6	31	No	
Nigeria	FCT	Meso	302	893	1998	44.0	71.0	32	No	
Nigeria	Gombe	Hyper	1,134	3,356	1999	12.0	68.3	31	No	
Nigeria	Imo Abia	Hyper	783	2,319	1999	69.0	65.5	31	No	
Nigeria	Jigawa	Hypo	212	629	1999	24.0	63.0	31	No	
Nigeria	Kaduna	Meso	1,650	4,884	1991	77.3	77.0	39	No	
Nigeria	Kano	Hyper	589	1,743	1999	45.0	76.0	31	No	
Nigeria	Kebbi	Hypo	124	366	1999	57.0	66.8	31	No	
Nigeria	Kogi	Hyper	1,063	3,146	1998	18.0	75.8	32	No	
Nigeria	Kwara	Hyper	898	2,659	1999	33.0	73.0	31	No	

(continued)

Country	Project	Endem. levels**	Pop. at risk (x1000) 1990	2030	MDA † start	cov. at start (%)	mean cov. (%)	No. rounds 1990-2030	Vector control	Remarks
Nigeria	Niger	Meso	1,579	4,675	1999	16.0	66.6	31	No	
Nigeria	Ogun	Meso	205	606	2002	57.0	77.2	28	No	
Nigeria	Ondo	Meso	814	2,409	1999	51.0	75.9	31	No	
Nigeria	Osun	Meso	960	2,842	1998	13.0	63.3	32	No	
Nigeria	Oyo	Meso	655	1,938	1999	31.0	65.4	31	No	
Nigeria	P20Nigeria*	Hyper	132	391	2019	79.7	73.0	11	No	
Nigeria	P5Nigeria*	Hypo	4,491	13,294	2025	65.0	65.0	6	No	*Loa loa* coendemicity
Nigeria	Plateau Nassarawa	Meso	911	2,695	1998	53.0	72.7	20	No	PTS since 2018 [10]‡
Nigeria	Plateau Nassarawa LF	Hyper	922	2,729	2000	81.5	81.5	18	No	PTS since 2018 [10]‡
Nigeria	Taraba	Hyper	991	2,932	1998	45.0	69.1	32	No	
Nigeria	Yobe	Meso	366	1,085	1999	51.0	71.4	31	No	
Nigeria	Zamfara	Hypo	170	503	1999	60.0	73.8	31	No	
S. Sudan	East Bahr El Ghazal	Hyper	326	966	2005	32.0	51.3	21	No	Civil war between 2013-2019
S. Sudan	East Equatoria	Hyper	580	1,716	2006	17.0	41.3	17	No	Civil war between 2013-2019
S. Sudan	P20SouthSudan*	Hyper	16	48	2020	<0.05	54.5	10	No	Civil war between 2013-2019
S. Sudan	P5SouthSudan*	Hypo	450	1,331	2025	65.0	65.0	6	No	*Loa loa* coendemicity ?
S. Sudan	Upper Nile	Meso	304	899	2006	12.0	39.5	17	No	Civil war between 2013-2019
S. Sudan	West Bahr El Ghazal	Hyper	1,758	5,205	2006	3.0	44.3	24	No	
S. Sudan	West Equatoria	Hyper	415	1,228	2005	36.0	62.8	25	No	
Sudan	P5Sudan*	Hypo	127	375	2025	65.0	65.0	6	No	*Loa loa* coendemicity
Sudan	Sudan	Hypo	120	354	2008	81.5	78.0	22	No	
Sudan	Sudan Abu Hamed	Hypo	120	354	2008	81.5	78.0	22	No	
Tanzania	Kilosa	Meso	287	849	2002	62.0	74.6	28	No	
Tanzania	Mahenge	Hyper	287	849	1998	27.0	68.4	32	No	
Tanzania	Morogoro	Meso	205	608	2004	58.0	73.1	25	No	
Tanzania	P5Tanzania*	Hypo	520	1,540	2023	65.0	65.0	8	No	
Tanzania	Ruvuma	Hyper	227	671	1999	35.0	68.2	31	No	
Tanzania	Tanga	Meso	178	526	2001	46.0	73.1	29	No	
Tanzania	Tukuyu	Meso	65	193	2001	65.0	75.0	29	No	
Tanzania	Tunduru	Hyper	71	209	2005	65.0	77.3	25	No	
Uganda	P5Uganda*	Hypo	131	387	1997	75.0	72.8	66	No	Bi-annual MDA since 2007

(continued)

Country	Project	Endem. levels**	Pop. at risk (x1000) 1990	2030	MDA † start	cov. at start (%)	mean cov. (%)	No. rounds 1990-2030	Vector control	Remarks
Uganda	Phase 1¶	Hyper	205	607	1999	52.0	73.2	62	Yes	Bi-annual MDA since 2007; Three years of focal vector control; Interruption suspected, or transmission halted in many foci; Onchocerciasis eliminated in other foci since 2017
Uganda	Phase 2¶	Hypo	399	1,181	1999	50.0	72.4	36	Yes	Bi-annual MDA since 2007; Three years of focal vector control; Onchocerciasis eliminated since 2017
Uganda	Phase 3¶	Hyper	736	2,178	1999	37.0	63.8	62	Yes	Bi-annual MDA since 2007; Three years of focal vector control.
Uganda	Phase 4¶	Hyper	416	1,233	1999	72.0	77.2	36	Yes	Bi-annual MDA since 2007; Three years of focal vector control; Onchocerciasis eliminated since 2017
Uganda	Phase 5¶	Hyper	268	794	2012	75.0	71.1	36	Yes	Bi-annual MDA since 2012

Note 1: Population in thousands.

Note 2: We assumed that all APOC-projects would continue MDA until at least the target year of onchocerciasis elimination (2030) except for Bioko (Eq. Guinea) [1-3]‡, several APOC projects in Uganda [4-9]‡, and Plateau and Nassarawa in Nigeria [10]‡.

* P5 and P20 APOC-projects are a compilation of multiple areas within a country with a prevalence of palpable nodule of $\geq 5\%- < 20\%$ and $\geq 20\%$ respectively.

** Pre-control endemicity levels in terms of mf prevalence in general population (all ages) according to Prost et al. [11]‡ with following adapted cut-offs: hypoendemic ($\geq 1\%- < 35\%$), mesoendemic ($\geq 35\%- < 60\%$), hyperendemic ($\geq 60\%- < 75\%$), very hyperendemic ($\geq 75\%$).

¶ Uganda applied focal vector control over its various APOC projects (i.e. temephos river larvaciding, ground larvaciding, trapping crabs, "slash-and-clear" of vegetation) [4,8,12]‡. As result, vector control was applied in various endemic foci in Uganda through monthly or two-monthly cycles during different time periods for different durations (many foci applied vector control for a combined total duration of two or three years). Several vector control efforts were already initiated in 1994/1995. We account for local vector control in Uganda by simulating three continuous years of vector control (1994-1997), in line with the implementation of the first vector control interventions in Uganda. In many foci in Uganda, transmission of onchocerciasis has been interrupted and in some foci the disease has been eliminated [4-9]‡.

† We make a distinction between the MDA coverage at start year of treatment and the mean therapeutic coverage over the total duration of MDA provided (2030 end year of simulations). The mean MDA coverage is for most treated APOC projects higher than the MDA coverage at time of initiation of mass chemotherapy due to scaling-up and initial implementation challenges. Treatment coverage is expressed as the number of people treated among the total at-risk population.

‡ References:
1. ESPEN Portal. Onchocerciasis maps. Expand Spec Proj Elimin Neglected Trop Dis (ESPEN) World Health Organization Regional Office for Africa. Available at: http://espen.afro.who.int/diseases/onchocerciasis. Accessed on: 10 May 2021.
2. Herrador Z, Garcia B, Ncogo P, Perteguer MJ, Rubio JM, Rivas E, et al. Interruption of onchocerciasis transmission in Bioko Island: Accelerating the movement from control to elimination in Equatorial Guinea. Wanji S. PLoS Negl Trop Dis. Public Library of Science; 2018;12: e0006471.
3. Traoré S, Wilson MD, Sima A, Barro T, Diallo A, Ak? A, et al. The elimination of the onchocerciasis vector from the island of Bioko as a result of larviciding by the WHO African Programme for Onchocerciasis Control. Acta Trop. 2009;111: 211-218.
4. Katabarwa MN, Lakwo T, Habomugisha P, Unnasch TR, Garms R, et al. (2018) After 70 years of fighting an age-old scourge, onchocerciasis in Uganda, the end is in sight. Int Health10: i79–i88.
5. Katabarwa MN, Lakwo T, Habomugisha P, Agunyo S, Byamukama E, et al. (2014) Transmission of Onchocerca volvulus by Simulium neavei in Mount Elgon focus of eastern Uganda has been interrupted. Am J Trop Med Hyg 90: 1159–1166.
6. Lakwo TL, Garms R, Rubaale T, Katabarwa M, Walsh F, et al. (2013) The disappearance of onchocerciasis from the Itwara focus, western Uganda after elimination of the vector Simulium neavei and 19 years of annual ivermectin treatments. Acta Trop 126: 218–221.
7. Katabarwa MN, Walsh F, Habomugisha P, Lakwo TL, Agunyo S, et al. (2012) Transmission of onchocerciasis in Wadelai focus of northwestern Uganda has been interrupted and the disease eliminated. J Parasitol Res 2012.
8. Katabarwa MN, Katamanywa J, Lakwo T, Habomugisha P, Byamukama E, et al. (2016) The imaramagambo onchocerciasis focus in southwestern Uganda: Interruption of transmission after disappearance of the vector simulium neavei and its associated freshwater crabs. Am J Trop Med Hyg 95: 417–425.
9. Katabarwa MN, Habomugisha P, Khainza A, Oguttu DW, Byamukama E, et al. (2020) Historical elimination of onchocerciasis from victoria nile focus in Central Uganda verified using WHO criteria. Am J Trop Med Hyg 102: 1411–1416.
10. Richards FO, Eigege A, Umaru J, Kahansim B, Adelamo S, et al. (2020) The interruption of transmission of human onchocerciasis by an annual mass drug administration program in Plateau and Nasarawa States, Nigeria. Am J Trop Med Hyg 102: 582–592.
11. Prost A, Hervouet JP, Thylefors B. The degrees of endemicity of onchocerciasis. Bull World Health Organ. 1979; 57: 655-662.
12. Jacob BG, Loum D, Lakwo TL, Katholi CR, Habomugisha P, Byamukama E, et al. (2018) Community-directed vector control to supplement mass drug distribution for onchocerciasis elimination in the Madi mid-North focus of Northern Uganda. PLoS Negl Trop Dis 12(8): e0006702.
