# Supplementary material for: The burden of skin disease and eye disease due to onchocerciasis in countries formerly under the African Programme for Onchocerciasis Control mandate for 1990, 2020, and 2030
Source: PLoS Negl Trop Dis. 2021 Jul 26;15(7):e0009604. doi: 10.1371/journal.pntd.0009604 (PMC8312930; doi:10.1371/journal.pntd.0009604)
Supplement: S3 Text — (PDF) [file pntd.0009604.s003.pdf]

The burden of skin disease and eye disease due to onchocerciasis in Africa for 1990, 2020, and 2030

S3 Text:
Additional tables with case numbers by age, sex, endemicity level, or APOC-project

Natalie V.S. Vinkeles Melchers[1], Wilma A. Stolk[1], Welmoed van Loon[1,2], Belén Pedrique[3], Roel Bakker[1], Michele E. Murdoch[4], Sake J. de Vlas[1], Luc E. Coffeng[1]

1. Department of Public Health, Erasmus MC, University Medical Center Rotterdam, Rotterdam, The Netherlands.
2. Institute of Tropical Medicine and International Health, Charité - Universitätsmedizin Berlin, corporate member of Freie Universität Berlin, Humboldt-Universität zu Berlin, and Berlin Institute of Health, Berlin, Germany.
3. Drugs for Neglected Diseases initiative (DND*i*), Geneva, Switzerland
4. Department of Dermatology, Watford General Hospital, Watford, Hertfordshire, UK

* Correspondence to:

Luc E. Coffeng, Department of Public Health, Erasmus MC, University Medical Center Rotterdam, P.O. box 2040, 3000 CA Rotterdam, The Netherlands; l.coffeng@erasmusmc.nl

Alternative:
Natalie VS Vinkeles Melchers, Department of Public Health, Erasmus MC, University Medical Center Rotterdam, P.O. box 2040, 3000 CA Rotterdam, The Netherlands; n.vinkelesmelchers@erasmusmc.nl, Natalie.melchers@gmail.com; +31 614217276

Table G: Population at risk, number of cases, and DALYs lost due to onchocercal eye disease (OED) stratified by bioclime for 1990, 2020, and 2030. Absolute numbers (N and DALYs) are presented in thousands.

	1990			2020			2030		
	N	%	DALYs	N	%	DALYs	N	%	DALYs
**Forest areas**									
Total populaton at risk	54,399	100	_	122,757	100	_	161,014	100	_
Visual impairment	48	0.1	1	48	<0.05	1	30	<0.05	1
Blindness	62	0.1	430	39	<0.05	268	17	<0.05	115
Eye disease (OED)	109	0.2	432	87	0.1	270	47	<0.05	116
**Savanna areas**									
Total populaton at risk	25,369	100	_	57,247	100	_	75,088	100	_
Visual impairment	374	1.5	12	378	0.7	12	286	0.4	9
Blindness	133	0.5	966	87	0.2	635	52	0.1	376
Eye disease (OED)	506	2.0	978	465	0.8	647	338	0.4	385
Total									
Total populaton at risk	79,768	100	_	180,004	100	_	236,102	100	_
Visual impairment	421	0.5	13	426	0.2	13	316	0.1	10
Blindness	194	0.2	1,397	126	0.1	903	69	<0.05	491
Eye disease (OED)	616	0.8	1,410	552	0.3	916	384	0.2	501

Table H: Population at risk and number of cases stratified by age (males and females combined) for 1990. Absolute numbers (N and DALYs) are presented in thousands.

Age category:	0-1		2-4		5-9		10-19		20-29		30-49		50+		Total	
	N	%	N	%	N	%	N	%	N	%	N	%	N	%	N	%
Total populaton at risk	6,314	100	7,999	100	11,068	100	17,857	100	13,210	100	15,720	100	7,599	100	79,768	100
**Infection:**																
Mf-positive	<0.5	<0.05	42	0.5	883	8.0	5,663	31.7	6,722	50.9	8,546	54.4	4,107	54.0	25,964	32.5
Mf-positive (age 5+)	<0.5	<0.05	<0.5	<0.05	883	8.0	5,663	31.7	6,722	50.9	8,546	54.4	4,107	54.0	25,922	32.5
Worm infected	7	0.1	345	4.3	2,247	20.3	8,067	45.2	7,842	59.4	9,668	61.5	4,650	61.2	32,825	41.2
**Clinical manifestations:**																
Palpable nodules	<0.5	<0.05	5	0.1	144	1.3	1,354	7.6	2,053	15.5	2,756	17.5	1,304	17.2	7,617	9.5
Reversible skin disease:																
Severe itch	<0.5	<0.05	<0.5	<0.05	13	0.1	411	2.3	1,021	7.7	1,778	11.3	891	11.7	4,115	5.2
Reactive skin disease	<0.5	<0.05	<0.5	<0.05	11	0.1	343	1.9	817	6.2	1,300	8.3	618	8.1	3,089	3.9
**Reversible skin disease**	**<0.5**	**<0.05**	**<0.5**	**<0.05**	**23**	**0.2**	**755**	**4.2**	**1,839**	**13.9**	**3,078**	**19.6**	**1,510**	**19.9**	**7,205**	**9.0**
Irreversible skin disease:																
Mild depigmentation	<0.5	<0.05	<0.5	<0.05	<0.5	<0.05	30	0.2	145	1.1	433	2.8	327	4.3	935	1.2
Severe depigmentation	<0.5	<0.05	<0.5	<0.05	<0.5	<0.05	13	0.1	104	0.8	462	2.9	479	6.3	1,057	1.3
Atrophy	<0.5	<0.05	<0.5	<0.05	<0.5	<0.05	1	<0.05	15	0.1	<0.5	<0.05	<0.5	<0.05	16	<0.05
Hanging groin	<0.5	<0.05	<0.5	<0.05	<0.5	<0.05	<0.5	<0.05	<0.5	<0.05	9	0.1	23	0.3	33	<0.05
**Irreversible skin disease**	**<0.5**	**<0.05**	**<0.5**	**<0.05**	**<0.5**	**<0.05**	**44**	**0.2**	**264**	**2.0**	**904**	**5.7**	**829**	**10.9**	**2,041**	**2.6**
**Total skin disease (OSD)**	**<0.5**	**<0.05**	**5**	**0.1**	**167**	**1.5**	**2,152**	**12.1**	**4,156**	**31.5**	**6,738**	**42.9**	**3,643**	**47.9**	**16,862**	**21.1**
Eye disease (OED):																
Visual impairment	<0.5	<0.05	<0.5	<0.05	<0.5	<0.05	3	<0.05	32	0.2	183	1.2	203	2.7	421	0.5
Blindness	<0.5	<0.05	<0.5	<0.05	<0.5	<0.05	<0.5	<0.05	10	0.1	87	0.6	97	1.3	194	0.2
**Eye disease (OED)**	**<0.5**	**<0.05**	**<0.5**	**<0.05**	**<0.5**	**<0.05**	**3**	**<0.05**	**42**	**0.3**	**270**	**1.7**	**300**	**3.9**	**616**	**0.8**
**Total all manifestations**	**<0.5**	**<0.05**	**5**	**0.1**	**167**	**1.5**	**2,155**	**12.1**	**4,199**	**31.8**	**7,008**	**44.6**	**3,943**	**51.9**	**17,478**	**21.9**

Note: Total skin disease (OSD) is defined as the sum of the number of cases with palpable nodules as well as reversible and irreversible skin disease.

Table I: Population at risk and number of cases in **males** stratified by age for 1990. Absolute numbers (N and DALYs) are presented in thousands.

Age category:	0-1		2-4		5-9		10-19		20-29		30-49		50+		Total	
	N	%	N	%	N	%	N	%	N	%	N	%	N	%	N	%
Total populaton at risk	3,156	100	3,997	100	5,539	100	8,936	100	6,615	100	7,890	100	3,805	100	39,940	100
**Infection:**																
Mf-positive	<0.5	<0.05	27	0.7	536	9.7	3,216	36.0	3,648	55.1	4,602	58.3	2,203	57.9	14,231	35.6
Mf-positive (age 5+)	<0.5	<0.05	<0.5	<0.05	536	9.7	3,216	36.0	3,648	55.1	4,602	58.3	2,203	57.9	14,204	35.6
Worm infected	4	0.1	199	5.0	1,266	22.9	4,359	48.8	4,121	62.3	5,060	64.1	2,425	63.7	17,434	43.7
**Clinical manifestations:**																
Palpable nodules	<0.5	<0.05	3	0.1	90	1.6	808	9.0	1,189	18.0	1,585	20.1	746	19.6	4,423	11.1
Reversible skin disease:																
Severe itch	<0.5	<0.05	<0.5	<0.05	8	0.1	254	2.8	605	9.1	1,035	13.1	516	13.6	2,418	6.1
Reactive skin disease	<0.5	<0.05	<0.5	<0.05	7	0.1	214	2.4	493	7.4	775	9.8	367	9.6	1,856	4.6
**Reversible skin disease**	**<0.5**	**<0.05**	**<0.5**	**<0.05**	**15**	**0.3**	**468**	**5.2**	**1,097**	**16.6**	**1,810**	**22.9**	**883**	**23.2**	**4,274**	**10.7**
Irreversible skin disease:																
Mild depigmentation	<0.5	<0.05	<0.5	<0.05	<0.5	<0.05	19	0.2	89	1.3	252	3.2	183	4.8	544	1.4
Severe depigmentation	<0.5	<0.05	<0.5	<0.05	<0.5	<0.05	9	0.1	67	1.0	286	3.6	288	7.6	650	1.6
Atrophy	<0.5	<0.05	<0.5	<0.05	<0.5	<0.05	1	<0.05	10	0.2	<0.5	<0.05	<0.5	<0.05	11	<0.05
Hanging groin	<0.5	<0.05	<0.5	<0.05	<0.5	<0.05	<0.5	<0.05	<0.5	<0.05	7	0.1	16	0.4	23	0.1
**Irreversible skin disease**	**<0.5**	**<0.05**	**<0.5**	**<0.05**	**<0.5**	**<0.05**	**29**	**0.3**	**167**	**2.5**	**545**	**6.9**	**488**	**12.8**	**1,228**	**3.1**
**Total skin disease (OSD)**	**<0.5**	**<0.05**	**3**	**0.1**	**105**	**1.9**	**1,305**	**14.6**	**2,454**	**37.1**	**3,941**	**49.9**	**2,117**	**55.6**	**9,925**	**24.9**
Eye disease (OED):																
Visual impairment	<0.5	<0.05	<0.5	<0.05	<0.5	<0.05	2	<0.05	22	0.3	117	1.5	123	3.2	264	0.7
Blindness	<0.5	<0.05	<0.5	<0.05	<0.5	<0.05	<0.5	<0.05	7	0.1	60	0.8	63	1.7	131	0.3
**Eye disease (OED)**	**<0.5**	**<0.05**	**<0.5**	**<0.05**	**<0.5**	**<0.05**	**2**	**<0.05**	**29**	**0.4**	**177**	**2.2**	**186**	**4.9**	**395**	**1.0**
**Total all manifestations**	**<0.5**	**<0.05**	**3**	**0.1**	**105**	**1.9**	**1,308**	**14.6**	**2,483**	**37.5**	**4,118**	**52.2**	**2,303**	**60.5**	**10,320**	**25.8**

Note: Total skin disease (OSD) is defined as the sum of the number of cases with palpable nodules as well as reversible and irreversible skin disease.

Table J: Population at risk and number of cases in **females** stratified by age for 1990. Absolute numbers (N and DALYs) are presented in thousands.

Age category:	0-1		2-4		5-9		10-19		20-29		30-49		50+		Total	
	N	%	N	%	N	%	N	%	N	%	N	%	N	%	N	%
Total populaton at risk	3,157	100	4,002	100	5,529	100	8,921	100	6,594	100	7,831	100	3,795	100	39,828	100
**Infection:**																
Mf-positive	<0.5	<0.05	15	0.4	347	6.3	2,447	27.4	3,074	46.6	3,945	50.4	1,904	50.2	11,732	29.5
Mf-positive (age 5+)	<0.5	<0.05	<0.5	<0.05	347	6.3	2,447	27.4	3,074	46.6	3,945	50.4	1,904	50.2	11,717	29.4
Worm infected	3	0.1	146	3.6	980	17.7	3,708	41.6	3,721	56.4	4,608	58.8	2,225	58.6	15,391	38.6
**Clinical manifestations:**																
Palpable nodules	<0.5	<0.05	2	0.1	54	1.0	545	6.1	864	13.1	1,171	15.0	558	14.7	3,194	8.0
Reversible skin disease:																
Severe itch	<0.5	<0.05	<0.5	<0.05	4	0.1	158	1.8	417	6.3	743	9.5	375	9.9	1,697	4.3
Reactive skin disease	<0.5	<0.05	<0.5	<0.05	4	0.1	129	1.4	324	4.9	525	6.7	252	6.6	1,234	3.1
**Reversible skin disease**	**<0.5**	**<0.05**	**<0.5**	**<0.05**	**8**	**0.1**	**287**	**3.2**	**741**	**11.2**	**1,268**	**16.2**	**627**	**16.5**	**2,931**	**7.4**
Irreversible skin disease:																
Mild depigmentation	<0.5	<0.05	<0.5	<0.05	<0.5	<0.05	10	0.1	56	0.8	181	2.3	144	3.8	391	1.0
Severe depigmentation	<0.5	<0.05	<0.5	<0.05	<0.5	<0.05	4	<0.05	37	0.6	176	2.2	190	5.0	407	1.0
Atrophy	<0.5	<0.05	<0.5	<0.05	<0.5	<0.05	<0.5	<0.05	5	0.1	<0.5	<0.05	<0.5	<0.05	5	<0.05
Hanging groin	<0.5	<0.05	<0.5	<0.05	<0.5	<0.05	<0.5	<0.05	<0.5	<0.05	2	<0.05	7	0.2	10	<0.05
**Irreversible skin disease**	**<0.5**	**<0.05**	**<0.5**	**<0.05**	**<0.5**	**<0.05**	**15**	**0.2**	**97**	**1.5**	**359**	**4.6**	**341**	**9.0**	**813**	**2.0**
**Total skin disease (OSD)**	**<0.5**	**<0.05**	**2**	**0.1**	**62**	**1.1**	**847**	**9.5**	**1,703**	**25.8**	**2,797**	**35.7**	**1,527**	**40.2**	**6,937**	**17.4**
Eye disease (OED):																
Visual impairment	<0.5	<0.05	<0.5	<0.05	<0.5	<0.05	1	<0.05	10	0.2	66	0.8	81	2.1	157	0.4
Blindness	<0.5	<0.05	<0.5	<0.05	<0.5	<0.05	<0.5	<0.05	3	<0.05	27	0.3	33	0.9	63	0.2
**Eye disease (OED)**	**<0.5**	**<0.05**	**<0.5**	**<0.05**	**<0.5**	**<0.05**	**1**	**<0.05**	**13**	**0.2**	**93**	**1.2**	**114**	**3.0**	**221**	**0.6**
**Total all manifestations**	**<0.5**	**<0.05**	**2**	**0.1**	**62**	**1.1**	**848**	**9.5**	**1,716**	**26.0**	**2,890**	**36.9**	**1,641**	**43.2**	**7,158**	**18.0**

Note: Total skin disease (OSD) is defined as the sum of the number of cases with palpable nodules as well as reversible and irreversible skin disease.

Table K: Population at risk and number of cases stratified by age (males and females combined) for 2020. Absolute numbers (N and DALYs) are presented in thousands.

Age category:	0-1		2-4		5-9		10-19		20-29		30-49		50+		Total	
	N	%	N	%	N	%	N	%	N	%	N	%	N	%	N	%
Total populaton at risk	14,214	100	18,055	100	24,943	100	40,253	100	29,820	100	35,498	100	17,221	100	180,004	100
**Infection:**																
Mf-positive	<0.5	<0.05	8	<0.05	268	1.1	2,570	6.4	3,755	12.6	5,057	14.2	2,433	14.1	14,092	7.8
Mf-positive (age 5+)	<0.5	<0.05	<0.5	<0.05	268	1.1	2,570	6.4	3,755	12.6	5,057	14.2	2,433	14.1	14,084	7.8
Worm infected	2	<0.05	138	0.8	1,119	4.5	5,619	14.0	6,792	22.8	9,040	25.5	4,363	25.3	27,073	15.0
**Clinical manifestations:**																
Palpable nodules	<0.5	<0.05	1	<0.05	36	0.1	517	1.3	1,025	3.4	1,517	4.3	714	4.1	3,810	2.1
Reversible skin disease:																
Severe itch	<0.5	<0.05	<0.5	<0.05	2	<0.05	96	0.2	345	1.2	787	2.2	433	2.5	1,663	0.9
Reactive skin disease	<0.5	<0.05	<0.5	<0.05	2	<0.05	67	0.2	217	0.7	374	1.1	186	1.1	845	0.5
**Reversible skin disease**	**<0.5**	**<0.05**	**<0.5**	**<0.05**	**3**	**<0.05**	**163**	**0.4**	**561**	**1.9**	**1,161**	**3.3**	**619**	**3.6**	**2,508**	**1.4**
Irreversible skin disease:																
Mild depigmentation	<0.5	<0.05	<0.5	<0.05	<0.5	<0.05	6	<0.05	62	0.2	461	1.3	588	3.4	1,117	0.6
Severe depigmentation	<0.5	<0.05	<0.5	<0.05	<0.5	<0.05	2	<0.05	32	0.1	404	1.1	744	4.3	1,181	0.7
Atrophy	<0.5	<0.05	<0.5	<0.05	<0.5	<0.05	<0.5	<0.05	3	<0.05	<0.5	<0.05	<0.5	<0.05	3	<0.05
Hanging groin	<0.5	<0.05	<0.5	<0.05	<0.5	<0.05	<0.5	<0.05	<0.5	<0.05	3	<0.05	23	0.1	27	<0.05
**Irreversible skin disease**	**<0.5**	**<0.05**	**<0.5**	**<0.05**	**<0.5**	**<0.05**	**8**	**<0.05**	**97**	**0.3**	**869**	**2.4**	**1,355**	**7.9**	**2,328**	**1.3**
**Total skin disease (OSD)**	**<0.5**	**<0.05**	**1**	**<0.05**	**40**	**0.2**	**688**	**1.7**	**1,683**	**5.6**	**3,547**	**10.0**	**2,687**	**15.6**	**8,647**	**4.8**
Eye disease (OED):																
Visual impairment	<0.5	<0.05	<0.5	<0.05	<0.5	<0.05	1	<0.05	10	<0.05	122	0.3	292	1.7	426	0.2
Blindness	<0.5	<0.05	<0.5	<0.05	<0.5	<0.05	<0.5	<0.05	3	<0.05	40	0.1	83	0.5	126	0.1
**Eye disease (OED)**	**<0.5**	**<0.05**	**<0.5**	**<0.05**	**<0.5**	**<0.05**	**1**	**<0.05**	**13**	**<0.05**	**162**	**0.5**	**376**	**2.2**	**552**	**0.3**
**Total all manifestations**	**<0.5**	**<0.05**	**1**	**<0.05**	**40**	**0.2**	**688**	**1.7**	**1,696**	**5.7**	**3,710**	**10.5**	**3,063**	**17.8**	**9,198**	**5.1**

Note: Total skin disease (OSD) is defined as the sum of the number of cases with palpable nodules as well as reversible and irreversible skin disease.

Table L: Population at risk and number of cases in **males** stratified by age for 2020. Absolute numbers (N and DALYs) are presented in thousands.

Age category:	0-1		2-4		5-9		10-19		20-29		30-49		50+		Total	
	N	%	N	%	N	%	N	%	N	%	N	%	N	%	N	%
Total populaton at risk	7,108	100	9,024	100	12,471	100	20,159	100	14,926	100	17,837	100	8,635	100	90,161	100
**Infection:**																
Mf-positive	<0.5	<0.05	5	0.1	170	1.4	1,517	7.5	2,118	14.2	2,839	15.9	1,356	15.7	8,006	8.9
Mf-positive (age 5+)	<0.5	<0.05	<0.5	<0.05	170	1.4	1,517	7.5	2,118	14.2	2,839	15.9	1,356	15.7	8,000	8.9
Worm infected	1	<0.05	80	0.9	644	5.2	3,120	15.5	3,691	24.7	4,909	27.5	2,357	27.3	14,802	16.4
**Clinical manifestations:**																
Palpable nodules	<0.5	<0.05	1	<0.05	23	0.2	318	1.6	617	4.1	905	5.1	424	4.9	2,288	2.5
Reversible skin disease:																
Severe itch	<0.5	<0.05	<0.5	<0.05	1	<0.05	62	0.3	212	1.4	477	2.7	261	3.0	1,013	1.1
Reactive skin disease	<0.5	<0.05	<0.5	<0.05	1	<0.05	43	0.2	136	0.9	231	1.3	116	1.3	527	0.6
**Reversible skin disease**	**<0.5**	**<0.05**	**<0.5**	**<0.05**	**2**	**<0.05**	**106**	**0.5**	**348**	**2.3**	**708**	**4.0**	**376**	**4.4**	**1,540**	**1.7**
Irreversible skin disease:																
Mild depigmentation	<0.5	<0.05	<0.5	<0.05	<0.5	<0.05	4	<0.05	39	0.3	278	1.6	336	3.9	656	0.7
Severe depigmentation	<0.5	<0.05	<0.5	<0.05	<0.5	<0.05	1	<0.05	22	0.1	258	1.4	450	5.2	731	0.8
Atrophy	<0.5	<0.05	<0.5	<0.05	<0.5	<0.05	<0.5	<0.05	2	<0.05	<0.5	<0.05	<0.5	<0.05	2	<0.05
Hanging groin	<0.5	<0.05	<0.5	<0.05	<0.5	<0.05	<0.5	<0.05	<0.5	<0.05	3	<0.05	17	0.2	19	<0.05
**Irreversible skin disease**	**<0.5**	**<0.05**	**<0.5**	**<0.05**	**<0.5**	**<0.05**	**5**	**<0.05**	**63**	**0.4**	**538**	**3.0**	**802**	**9.3**	**1,409**	**1.6**
**Total skin disease (OSD)**	**<0.5**	**<0.05**	**1**	**<0.05**	**25**	**0.2**	**429**	**2.1**	**1,028**	**6.9**	**2,151**	**12.1**	**1,602**	**18.6**	**5,236**	**5.8**
Eye disease (OED):																
Visual impairment	<0.5	<0.05	<0.5	<0.05	<0.5	<0.05	<0.5	<0.05	7	<0.05	82	0.5	181	2.1	271	0.3
Blindness	<0.5	<0.05	<0.5	<0.05	<0.5	<0.05	<0.5	<0.05	2	<0.05	29	0.2	56	0.6	87	0.1
**Eye disease (OED)**	**<0.5**	**<0.05**	**<0.5**	**<0.05**	**<0.5**	**<0.05**	**1**	**<0.05**	**9**	**0.1**	**110**	**0.6**	**238**	**2.8**	**358**	**0.4**
**Total all manifestations**	**<0.5**	**<0.05**	**1**	**<0.05**	**25**	**0.2**	**430**	**2.1**	**1,037**	**6.9**	**2,261**	**12.7**	**1,840**	**21.3**	**5,594**	**6.2**

Note: Total skin disease (OSD) is defined as the sum of the number of cases with palpable nodules as well as reversible and irreversible skin disease.

Table M: Population at risk and number of cases in **females** stratified by age for 2020. Absolute numbers (N and DALYs) are presented in thousands.

Age category:	0-1		2-4		5-9		10-19		20-29		30-49		50+		Total	
	N	%	N	%	N	%	N	%	N	%	N	%	N	%	N	%
Total populaton at risk	7,105	100	9,031	100	12,471	100	20,094	100	14,895	100	17,660	100	8,587	100	89,843	100
**Infection:**																
Mf-positive	<0.5	<0.05	3	<0.05	98	0.8	1,053	5.2	1,637	11.0	2,218	12.6	1,077	12.5	6,086	6.8
Mf-positive (age 5+)	<0.5	<0.05	<0.5	<0.05	98	0.8	1,053	5.2	1,637	11.0	2,218	12.6	1,077	12.5	6,083	6.8
Worm infected	1	<0.05	58	0.6	475	3.8	2,499	12.4	3,101	20.8	4,130	23.4	2,006	23.4	12,270	13.7
**Clinical manifestations:**																
Palpable nodules	<0.5	<0.05	<0.5	<0.05	13	0.1	199	1.0	408	2.7	612	3.5	290	3.4	1,522	1.7
Reversible skin disease:																
Severe itch	<0.5	<0.05	<0.5	<0.05	1	<0.05	34	0.2	133	0.9	310	1.8	172	2.0	650	0.7
Reactive skin disease	<0.5	<0.05	<0.5	<0.05	1	<0.05	24	0.1	80	0.5	143	0.8	71	0.8	318	0.4
**Reversible skin disease**	**<0.5**	**<0.05**	**<0.5**	**<0.05**	**1**	**<0.05**	**57**	**0.3**	**213**	**1.4**	**453**	**2.6**	**243**	**2.8**	**968**	**1.1**
Irreversible skin disease:																
Mild depigmentation	<0.5	<0.05	<0.5	<0.05	<0.5	<0.05	2	<0.05	23	0.2	184	1.0	252	2.9	461	0.5
Severe depigmentation	<0.5	<0.05	<0.5	<0.05	<0.5	<0.05	1	<0.05	10	0.1	147	0.8	293	3.4	450	0.5
Atrophy	<0.5	<0.05	<0.5	<0.05	<0.5	<0.05	<0.5	<0.05	1	<0.05	<0.5	<0.05	<0.5	<0.05	1	<0.05
Hanging groin	<0.5	<0.05	<0.5	<0.05	<0.5	<0.05	<0.5	<0.05	<0.5	<0.05	1	<0.05	7	0.1	8	<0.05
**Irreversible skin disease**	**<0.5**	**<0.05**	**<0.5**	**<0.05**	**<0.5**	**<0.05**	**3**	**<0.05**	**34**	**0.2**	**331**	**1.9**	**552**	**6.4**	**920**	**1.0**
**Total skin disease (OSD)**	**<0.5**	**<0.05**	**<0.5**	**<0.05**	**15**	**0.1**	**259**	**1.3**	**655**	**4.4**	**1,396**	**7.9**	**1,085**	**12.6**	**3,410**	**3.8**
Eye disease (OED):																
Visual impairment	<0.5	<0.05	<0.5	<0.05	<0.5	<0.05	<0.5	<0.05	3	<0.05	41	0.2	111	1.3	155	0.2
Blindness	<0.5	<0.05	<0.5	<0.05	<0.5	<0.05	<0.5	<0.05	1	<0.05	11	0.1	27	0.3	39	<0.05
**Eye disease (OED)**	**<0.5**	**<0.05**	**<0.5**	**<0.05**	**<0.5**	**<0.05**	**<0.5**	**<0.05**	**4**	**<0.05**	**52**	**0.3**	**138**	**1.6**	**194**	**0.2**
**Total all manifestations**	**<0.5**	**<0.05**	**<0.5**	**<0.05**	**15**	**0.1**	**259**	**1.3**	**659**	**4.4**	**1,448**	**8.2**	**1,223**	**14.2**	**3,604**	**4.0**

Note: Total skin disease (OSD) is defined as the sum of the number of cases with palpable nodules as well as reversible and irreversible skin disease.

Table N: Population at risk and number of cases stratified by age (males and females combined) for 2030. Absolute numbers (N and DALYs) are presented in thousands.

Age category:	0-1		2-4		5-9		10-19		20-29		30-49		50+		Total	
	N	%	N	%	N	%	N	%	N	%	N	%	N	%	N	%
Total populaton at risk	18,628	100	23,651	100	32,719	100	52,772	100	39,090	100	46,560	100	22,683	100	236,102	100
**Infection:**																
Mf-positive	<0.5	<0.05	1	<0.05	56	0.2	1,015	1.9	1,859	4.8	2,624	5.6	1,257	5.5	6,813	2.9
Mf-positive (age 5+)	<0.5	<0.05	<0.5	<0.05	56	0.2	1,015	1.9	1,859	4.8	2,624	5.6	1,257	5.5	6,812	2.9
Worm infected	1	<0.05	43	0.2	471	1.4	3,076	5.8	4,012	10.3	5,375	11.5	2,598	11.5	15,577	6.6
**Clinical manifestations:**																
Palpable nodules	<0.5	<0.05	<0.5	<0.05	8	<0.05	161	0.3	377	1.0	588	1.3	279	1.2	1,412	0.6
Reversible skin disease:																
Severe itch	<0.5	<0.05	<0.5	<0.05	<0.5	<0.05	14	<0.05	89	0.2	277	0.6	162	0.7	542	0.2
Reactive skin disease	<0.5	<0.05	<0.5	<0.05	<0.5	<0.05	7	<0.05	28	0.1	62	0.1	31	0.1	129	0.1
**Reversible skin disease**	**<0.5**	**<0.05**	**<0.5**	**<0.05**	**<0.5**	**<0.05**	**21**	**<0.05**	**117**	**0.3**	**339**	**0.7**	**193**	**0.9**	**670**	**0.3**
Irreversible skin disease:																
Mild depigmentation	<0.5	<0.05	<0.5	<0.05	<0.5	<0.05	1	<0.05	20	0.1	266	0.6	591	2.6	879	0.4
Severe depigmentation	<0.5	<0.05	<0.5	<0.05	<0.5	<0.05	<0.5	<0.05	8	<0.05	185	0.4	669	2.9	863	0.4
Atrophy	<0.5	<0.05	<0.5	<0.05	<0.5	<0.05	<0.5	<0.05	<0.5	<0.05	<0.5	<0.05	<0.5	<0.05	<0.5	<0.05
Hanging groin	<0.5	<0.05	<0.5	<0.05	<0.5	<0.05	<0.5	<0.05	<0.5	<0.05	1	<0.05	14	0.1	15	<0.05
**Irreversible skin disease**	**<0.5**	**<0.05**	**<0.5**	**<0.05**	**<0.5**	**<0.05**	**1**	**<0.05**	**29**	**0.1**	**452**	**1.0**	**1,274**	**5.6**	**1,756**	**0.7**
**Total skin disease (OSD)**	**<0.5**	**<0.05**	**<0.5**	**<0.05**	**8**	**<0.05**	**183**	**0.3**	**523**	**1.3**	**1,380**	**3.0**	**1,745**	**7.7**	**3,839**	**1.6**
Eye disease (OED):																
Visual impairment	<0.5	<0.05	<0.5	<0.05	<0.5	<0.05	<0.5	<0.05	4	<0.05	67	0.1	245	1.1	316	0.1
Blindness	<0.5	<0.05	<0.5	<0.05	<0.5	<0.05	<0.5	<0.05	<0.5	<0.05	16	<0.05	52	0.2	69	<0.05
**Eye disease (OED)**	**<0.5**	**<0.05**	**<0.5**	**<0.05**	**<0.5**	**<0.05**	**<0.5**	**<0.05**	**5**	**<0.05**	**83**	**0.2**	**297**	**1.3**	**384**	**0.2**
**Total all manifestations**	**<0.5**	**<0.05**	**<0.5**	**<0.05**	**8**	**<0.05**	**183**	**0.3**	**527**	**1.3**	**1,463**	**3.1**	**2,042**	**9.0**	**4,223**	**1.8**

Note: Total skin disease (OSD) is defined as the sum of the number of cases with palpable nodules as well as reversible and irreversible skin disease.

Table O: Population at risk and number of cases in **males** stratified by age for 2030. Absolute numbers (N and DALYs) are presented in thousands.

Age category:	0-1		2-4		5-9		10-19		20-29		30-49		50+		Total	
	N	%	N	%	N	%	N	%	N	%	N	%	N	%	N	%
Total populaton at risk	9,330	100	11,846	100	16,356	100	26,393	100	19,606	100	23,369	100	11,393	100	118,294	100
**Infection:**																
Mf-positive	<0.5	<0.05	1	<0.05	37	0.2	621	2.4	1,098	5.6	1,517	6.5	732	6.4	4,006	3.4
Mf-positive (age 5+)	<0.5	<0.05	<0.5	<0.05	37	0.2	621	2.4	1,098	5.6	1,517	6.5	732	6.4	4,005	3.4
Worm infected	1	<0.05	26	0.2	271	1.7	1,736	6.6	2,217	11.3	2,940	12.6	1,427	12.5	8,618	7.3
**Clinical manifestations:**																
Palpable nodules	<0.5	<0.05	<0.5	<0.05	5	<0.05	101	0.4	233	1.2	361	1.5	172	1.5	871	0.7
Reversible skin disease:																
Severe itch	<0.5	<0.05	<0.5	<0.05	<0.5	<0.05	9	<0.05	58	0.3	176	0.8	100	0.9	344	0.3
Reactive skin disease	<0.5	<0.05	<0.5	<0.05	<0.5	<0.05	5	<0.05	19	0.1	41	0.2	21	0.2	86	0.1
**Reversible skin disease**	**<0.5**	**<0.05**	**<0.5**	**<0.05**	**<0.5**	**<0.05**	**14**	**0.1**	**77**	**0.4**	**217**	**0.9**	**121**	**1.1**	**429**	**0.4**
Irreversible skin disease:																
Mild depigmentation	<0.5	<0.05	<0.5	<0.05	<0.5	<0.05	1	<0.05	13	0.1	162	0.7	342	3.0	518	0.4
Severe depigmentation	<0.5	<0.05	<0.5	<0.05	<0.5	<0.05	<0.5	<0.05	6	<0.05	120	0.5	412	3.6	537	0.5
Atrophy	<0.5	<0.05	<0.5	<0.05	<0.5	<0.05	<0.5	<0.05	<0.5	<0.05	<0.5	<0.05	<0.5	<0.05	<0.5	<0.05
Hanging groin	<0.5	<0.05	<0.5	<0.05	<0.5	<0.05	<0.5	<0.05	<0.5	<0.05	1	<0.05	10	0.1	11	<0.05
**Irreversible skin disease**	**<0.5**	**<0.05**	**<0.5**	**<0.05**	**<0.5**	**<0.05**	**1**	**<0.05**	**19**	**0.1**	**283**	**1.2**	**764**	**6.7**	**1,067**	**0.9**
**Total skin disease (OSD)**	**<0.5**	**<0.05**	**<0.5**	**<0.05**	**5**	**<0.05**	**116**	**0.4**	**329**	**1.7**	**861**	**3.7**	**1,057**	**9.3**	**2,367**	**2.0**
Eye disease (OED):																
Visual impairment	<0.5	<0.05	<0.5	<0.05	<0.5	<0.05	<0.5	<0.05	3	<0.05	44	0.2	155	1.4	201	0.2
Blindness	<0.5	<0.05	<0.5	<0.05	<0.5	<0.05	<0.5	<0.05	<0.5	<0.05	12	<0.05	36	0.3	48	<0.05
**Eye disease (OED)**	**<0.5**	**<0.05**	**<0.5**	**<0.05**	**<0.5**	**<0.05**	**<0.5**	**<0.05**	**3**	**<0.05**	**55**	**0.2**	**191**	**1.7**	**249**	**0.2**
**Total all manifestations**	**<0.5**	**<0.05**	**<0.5**	**<0.05**	**5**	**<0.05**	**116**	**0.4**	**332**	**1.7**	**916**	**3.9**	**1,248**	**11.0**	**2,617**	**2.2**

Note: Total skin disease (OSD) is defined as the sum of the number of cases with palpable nodules as well as reversible and irreversible skin disease.

Table P: Population at risk and number of cases in **females** stratified by age for 2030. Absolute numbers (N and DALYs) are presented in thousands.

Age category:	0-1		2-4		5-9		10-19		20-29		30-49		50+		Total	
	N	%	N	%	N	%	N	%	N	%	N	%	N	%	N	%
Total populaton at risk	9,298	100	11,805	100	16,363	100	26,378	100	19,484	100	23,191	100	11,290	100	117,808	100
**Infection:**																
Mf-positive	<0.5	<0.05	<0.5	<0.05	20	0.1	394	1.5	761	3.9	1,106	4.8	525	4.7	2,807	2.4
Mf-positive (age 5+)	<0.5	<0.05	<0.5	<0.05	20	0.1	394	1.5	761	3.9	1,106	4.8	525	4.7	2,807	2.4
Worm infected	<0.5	<0.05	17	0.1	200	1.2	1,341	5.1	1,795	9.2	2,435	10.5	1,171	10.4	6,958	5.9
**Clinical manifestations:**																
Palpable nodules	<0.5	<0.05	<0.5	<0.05	3	<0.05	60	0.2	144	0.7	227	1.0	107	0.9	541	0.5
Reversible skin disease:																
Severe itch	<0.5	<0.05	<0.5	<0.05	<0.5	<0.05	5	<0.05	31	0.2	101	0.4	62	0.5	198	0.2
Reactive skin disease	<0.5	<0.05	<0.5	<0.05	<0.5	<0.05	2	<0.05	9	<0.05	22	0.1	10	0.1	43	<0.05
**Reversible skin disease**	**<0.5**	**<0.05**	**<0.5**	**<0.05**	**<0.5**	**<0.05**	**7**	**<0.05**	**40**	**0.2**	**122**	**0.5**	**72**	**0.6**	**241**	**0.2**
Irreversible skin disease:																
Mild depigmentation	<0.5	<0.05	<0.5	<0.05	<0.5	<0.05	<0.5	<0.05	7	<0.05	104	0.4	249	2.2	360	0.3
Severe depigmentation	<0.5	<0.05	<0.5	<0.05	<0.5	<0.05	<0.5	<0.05	3	<0.05	65	0.3	257	2.3	325	0.3
Atrophy	<0.5	<0.05	<0.5	<0.05	<0.5	<0.05	<0.5	<0.05	<0.5	<0.05	<0.5	<0.05	<0.5	<0.05	<0.5	<0.05
Hanging groin	<0.5	<0.05	<0.5	<0.05	<0.5	<0.05	<0.5	<0.05	<0.5	<0.05	<0.5	<0.05	4	<0.05	4	<0.05
**Irreversible skin disease**	**<0.5**	**<0.05**	**<0.5**	**<0.05**	**<0.5**	**<0.05**	**<0.5**	**<0.05**	**10**	**0.1**	**170**	**0.7**	**510**	**4.5**	**689**	**0.6**
**Total skin disease (OSD)**	**<0.5**	**<0.05**	**<0.5**	**<0.05**	**3**	**<0.05**	**67**	**0.3**	**194**	**1.0**	**519**	**2.2**	**688**	**6.1**	**1,472**	**1.2**
Eye disease (OED):																
Visual impairment	<0.5	<0.05	<0.5	<0.05	<0.5	<0.05	<0.5	<0.05	1	<0.05	23	0.1	90	0.8	114	0.1
Blindness	<0.5	<0.05	<0.5	<0.05	<0.5	<0.05	<0.5	<0.05	<0.5	<0.05	5	<0.05	16	0.1	21	<0.05
**Eye disease (OED)**	**<0.5**	**<0.05**	**<0.5**	**<0.05**	**<0.5**	**<0.05**	**<0.5**	**<0.05**	**1**	**<0.05**	**27**	**0.1**	**106**	**0.9**	**135**	**0.1**
**Total all manifestations**	**<0.5**	**<0.05**	**<0.5**	**<0.05**	**3**	**<0.05**	**67**	**0.3**	**195**	**1.0**	**547**	**2.4**	**794**	**7.0**	**1,607**	**1.4**

Note: Total skin disease (OSD) is defined as the sum of the number of cases with palpable nodules as well as reversible and irreversible skin disease.

Table Q: Population at risk, number of cases with infection and clinical manifestations, and DALYs lost due to onchocerciasis stratified by endemicity level for 1990. Absolute numbers (N and DALYs) are presented in thousands.

	Hypoendemic			Mesoendemic			Hyperendemic			Very hyperendemic			Total		
	N	%	DALYs	N	%	DALYs	N	%	DALYs	N	%	DALYs	N	%	DALYs
Total populaton at risk	49,274	100	_	22,799	100	_	6,099	100	_	1,595	100	_	79,768	100	_
**Infection:**															
Mf-positive	10,633	21.6	_	10,096	44.3	_	4,041	66.3	_	1,193	74.8	_	25,964	32.5	_
Mf-positive (age 5+)	10,627	21.6	_	10,087	44.2	_	4,026	66.0	_	1,182	74.1	_	25,922	32.5	_
Worm infected	14,299	29.0	_	12,790	56.1	_	4,463	73.2	_	1,273	79.8	_	32,825	41.2	_
**Clinical manifestations:**															
Palpable nodules	1,117	2.3	12	3,377	14.8	36	2,274	37.3	24	848	53.2	9	7,617	9.5	81
Reversible skin disease:															
Severe itch	1,313	2.7	238	1,451	6.4	263	973	16.0	177	378	23.7	69	4,115	5.2	747
Reactive skin disease	874	1.8	40	1,025	4.5	47	836	13.7	39	355	22.3	16	3,089	3.9	143
**Reversible skin disease**	**2,186**	**4.4**	**279**	**2,477**	**10.9**	**311**	**1,808**	**29.7**	**215**	**733**	**46.0**	**85**	**7,205**	**9.0**	**890**
Irreversible skin disease:															
Mild depigmentation	358	0.7	4	328	1.4	3	186	3.1	2	63	4.0	1	935	1.2	10
Severe depigmentation	301	0.6	19	321	1.4	21	298	4.9	19	137	8.6	9	1,057	1.3	68
Atrophy	1	<0.05	<0.5	2	<0.05	<0.5	7	0.1	<0.5	7	0.4	<0.5	16	<0.05	<0.5
Hanging groin	1	<0.05	<0.5	2	<0.05	1	14	0.2	5	16	1.0	6	33	<0.05	13
**Irreversible skin disease**	**661**	**1.3**	**24**	**652**	**2.9**	**25**	**505**	**8.3**	**27**	**223**	**14.0**	**16**	**2,041**	**2.6**	**91**
**Total skin disease (OSD)**	**3,965**	**8.0**	**314**	**6,506**	**28.5**	**372**	**4,587**	**75.2**	**266**	**1,804**	**113.1**	**110**	**16,862**	**21.1**	**1,062**
Eye disease (OED):															
Visual impairment	232	0.5	7	113	0.5	4	55	0.9	2	20	1.3	1	421	0.5	13
Blindness	69	0.1	465	42	0.2	303	51	0.8	377	33	2.0	252	194	0.2	1,397
**Eye disease (OED)**	**301**	**0.6**	**472**	**156**	**0.7**	**307**	**106**	**1.7**	**378**	**53**	**3.3**	**252**	**616**	**0.8**	**1,410**
**Total all manifestations**	**4,266**	**8.7**	**786**	**6,661**	**29.2**	**678**	**4,693**	**76.9**	**645**	**1,857**	**116.4**	**362**	**17,478**	**21.9**	**2,472**

Note: Total skin disease (OSD) is defined as the sum of the number of cases with palpable nodules as well as reversible and irreversible skin disease.

Table R: Population at risk, number of cases with infection and clinical manifestations, and DALYs lost due to onchocerciasis stratified by endemicity level for 2020. Absolute numbers (N and DALYs) are presented in thousands.

	Hypoendemic			Mesoendemic			Hyperendemic			Very hyperendemic			Total		
	N	%	DALYs	N	%	DALYs	N	%	DALYs	N	%	DALYs	N	%	DALYs
Total populaton at risk	111,193	100	_	51,449	100	_	13,763	100	_	3,599	100	_	180,004	100	_
**Infection:**															
Mf-positive	6,821	6.1	_	3,732	7.3	_	2,345	17.0	_	1,194	33.2	_	14,092	7.8	_
Mf-positive (age 5+)	6,818	6.1	_	3,730	7.2	_	2,343	17.0	_	1,193	33.2	_	14,084	7.8	_
Worm infected	11,670	10.5	_	8,588	16.7	_	4,855	35.3	_	1,959	54.4	_	27,073	15.0	_
**Clinical manifestations:**															
Palpable nodules	725	0.7	8	1,278	2.5	14	1,138	8.3	12	669	18.6	7	3,810	2.1	40
Reversible skin disease:															
Severe itch	791	0.7	144	436	0.8	79	301	2.2	55	135	3.7	24	1,663	0.9	302
Reactive skin disease	481	0.4	22	213	0.4	10	114	0.8	5	38	1.0	2	845	0.5	39
**Reversible skin disease**	**1,272**	**1.1**	**166**	**649**	**1.3**	**89**	**414**	**3.0**	**60**	**172**	**4.8**	**26**	**2,508**	**1.4**	**341**
Irreversible skin disease:															
Mild depigmentation	438	0.4	5	371	0.7	4	227	1.6	2	81	2.3	1	1,117	0.6	12
Severe depigmentation	350	0.3	23	339	0.7	22	335	2.4	22	157	4.4	10	1,181	0.7	76
Atrophy	<0.5	<0.05	<0.5	<0.5	<0.05	<0.5	1	<0.05	<0.5	1	<0.05	<0.5	3	<0.05	<0.5
Hanging groin	1	<0.05	<0.5	1	<0.05	1	11	0.1	4	13	0.4	5	27	<0.05	10
**Irreversible skin disease**	**789**	**0.7**	**28**	**712**	**1.4**	**26**	**574**	**4.2**	**29**	**253**	**7.0**	**16**	**2,328**	**1.3**	**99**
**Total skin disease (OSD)**	**2,787**	**2.5**	**201**	**2,639**	**5.1**	**129**	**2,126**	**15.5**	**100**	**1,094**	**30.4**	**50**	**8,647**	**4.8**	**480**
Eye disease (OED):															
Visual impairment	244	0.2	8	103	0.2	3	55	0.4	2	23	0.6	1	426	0.2	13
Blindness	51	<0.05	344	22	<0.05	159	30	0.2	222	23	0.6	177	126	0.1	903
**Eye disease (OED)**	**295**	**0.3**	**352**	**125**	**0.2**	**162**	**85**	**0.6**	**224**	**45**	**1.3**	**178**	**552**	**0.3**	**916**
**Total all manifestations**	**3,083**	**2.8**	**553**	**2,764**	**5.4**	**291**	**2,212**	**16.1**	**324**	**1,139**	**31.7**	**228**	**9,198**	**5.1**	**1,397**

Note: Total skin disease (OSD) is defined as the sum of the number of cases with palpable nodules as well as reversible and irreversible skin disease.

Table S: Population at risk, number of cases with infection and clinical manifestations, and DALYs lost due to onchocerciasis by country for 1990. Absolute numbers (N and DALYs) are presented in thousands.

		Mf-positive		Palpable nodules			Reversible skin disease			Irreverisble skin disease			Eye disease (OED)			Total all manifestations		
Country	Pop. at risk	N	%	N	%	DALYs	N	%	DALYs	N	%	DALYs	N	%	DALYs	N	%	DALYs
Angola	1,326	333	25.1	55	4.2	1	72	5.4	9	21	1.6	1	<0.5	<0.05	1	148	11.2	11
Burundi	1,333	359	26.9	68	5.1	1	78	5.9	10	23	1.7	1	<0.5	<0.05	1	169	12.7	12
Cameroon	5,121	2,241	43.8	934	18.2	10	787	15.4	95	220	4.3	11	54	1.0	161	1,995	39.0	277
CAR	1,316	549	41.7	195	14.8	2	160	12.1	20	43	3.2	2	55	4.1	140	452	34.4	164
Chad	1,277	379	29.7	85	6.7	1	83	6.5	10	23	1.8	1	28	2.2	53	219	17.1	65
Congo	794	220	27.8	46	5.8	<0.5	51	6.5	6	15	1.8	1	<0.5	<0.05	1	112	14.1	9
DRC	23,006	9,075	39.4	3,366	14.6	36	2,963	12.9	361	843	3.7	42	70	0.3	287	7,242	31.5	726
Eq. Guinea	199	76	38.2	35	17.7	<0.5	35	17.7	4	11	5.6	1	2	1.1	11	83	42.0	16
Ethiopia	6,727	2,206	32.8	581	8.6	6	542	8.1	68	152	2.3	6	4	0.1	12	1,279	19.0	92
Gabon	49	11	21.6	1	2.5	<0.5	2	4.3	<0.5	1	1.3	<0.5	<0.5	<0.05	<0.5	4	8.1	<0.5
Liberia	881	343	39.0	100	11.3	1	72	8.2	9	19	2.1	1	<0.5	<0.05	<0.5	191	21.7	11
Malawi	1,172	273	23.3	41	3.5	<0.5	54	4.6	7	16	1.4	1	<0.5	<0.05	<0.5	111	9.5	8
Mozambique	36	7	20.0	1	2.0	<0.5	1	3.9	<0.5	<0.5	1.2	<0.5	<0.5	<0.05	<0.5	3	7.2	<0.5
Nigeria	28,322	7,352	26.0	1,430	5.0	15	1,638	5.8	207	468	1.7	17	296	1.0	505	3,832	13.5	744
South Sudan	3,849	1,122	29.1	270	7.0	3	262	6.8	33	73	1.9	3	93	2.4	202	698	18.1	241
Sudan	366	79	21.6	9	2.4	<0.5	16	4.3	2	5	1.3	<0.5	6	1.6	9	35	9.6	12
Tanzania	1,839	631	34.3	189	10.3	2	180	9.8	22	51	2.8	2	3	0.2	11	423	23.0	38
Uganda	2,155	708	32.9	209	9.7	2	207	9.6	25	60	2.8	3	4	0.2	15	480	22.3	45
**Total**	**79,768**	**25,964**	**32.5**	**7,617**	**9.5**	**81**	**7,205**	**9.0**	**890**	**2,041**	**2.6**	**91**	**616**	**0.8**	**1,410**	**17,478**	**21.9**	**2,472**

Table T: Population at risk, number of cases with infection and clinical manifestations, and DALYs lost due to onchocerciasis by country for 2020. Absolute numbers (N and DALYs) are presented in thousands.

		Mf-positive		Palpable nodules			Reversible skin disease			Irreverisble skin disease			Eye disease (OED)			Total all manifestations		
Country	Pop. at risk	N	%	N	%	DALYs	N	%	DALYs	N	%	DALYs	N	%	DALYs	N	%	DALYs
Angola	2,992	309	10.3	50	1.7	1	51	1.7	7	32	1.1	1	<0.5	<0.05	1	134	4.5	9
Burundi	3,007	220	7.3	33	1.1	<0.5	44	1.5	6	30	1.0	1	<0.5	<0.05	1	108	3.6	8
Cameroon	11,555	829	7.2	208	1.8	2	135	1.2	18	222	1.9	10	37	0.3	67	601	5.2	98
CAR	2,970	126	4.3	31	1.0	<0.5	21	0.7	3	34	1.1	1	33	1.1	48	119	4.0	52
Chad	2,882	109	3.8	21	0.7	<0.5	22	0.8	3	20	0.7	1	21	0.7	27	84	2.9	31
Congo	1,791	186	10.4	40	2.2	<0.5	43	2.4	5	21	1.2	1	<0.5	<0.05	1	104	5.8	8
DRC	51,916	7,182	13.8	2,464	4.7	26	1,244	2.4	174	1,121	2.2	52	64	0.1	215	4,893	9.4	467
Eq. Guinea	448	<0.5	<0.05	<0.5	<0.05	<0.5	<0.5	<0.05	<0.5	5	1.1	<0.5	<0.5	0.1	1	5	1.2	1
Ethiopia	15,180	1,614	10.6	381	2.5	4	338	2.2	45	207	1.4	8	4	<0.05	9	930	6.1	66
Gabon	112	22	20.0	3	2.3	<0.5	4	3.8	1	1	1.2	<0.5	<0.5	<0.05	<0.5	8	7.3	1
Liberia	1,987	5	0.3	2	0.1	<0.5	1	<0.05	<0.5	13	0.7	<0.5	<0.5	<0.05	<0.5	15	0.8	1
Malawi	2,645	1	<0.05	<0.5	<0.05	<0.5	<0.5	<0.05	<0.5	12	0.5	<0.5	<0.5	<0.05	<0.5	13	0.5	1
Mozambique	82	14	17.2	1	1.7	<0.5	3	3.1	<0.5	1	1.0	<0.5	<0.5	<0.05	<0.5	5	5.9	<0.5
Nigeria	63,911	2,084	3.3	278	0.4	3	390	0.6	51	408	0.6	15	261	0.4	316	1,338	2.1	385
South Sudan	8,686	979	11.3	212	2.4	2	128	1.5	18	98	1.1	4	117	1.3	211	555	6.4	235
Sudan	826	64	7.8	8	1.0	<0.5	13	1.6	2	7	0.8	<0.5	8	1.0	11	36	4.4	13
Tanzania	4,151	338	8.1	68	1.6	1	69	1.7	9	55	1.3	2	2	<0.05	4	194	4.7	16
Uganda	4,863	7	0.2	10	0.2	<0.5	3	0.1	<0.5	42	0.9	2	2	<0.05	4	56	1.1	6
**Total**	**180,004**	**14,092**	**7.8**	**3,810**	**2.1**	**40**	**2,508**	**1.4**	**341**	**2,328**	**1.3**	**99**	**552**	**0.3**	**916**	**9,198**	**5.1**	**1,397**

Table U: Population at risk, number of cases with infection and clinical manifestations, and DALYs lost due to onchocerciasis by country and APOC-project for 1990. Absolute numbers (N and DALYs) are presented in thousands.

			Mf-positive		Palpable nodules			Reversible skin disease			Irreverisble skin disease			Eye disease (OED)			Total all manifestations		
Country	Project	Pop. at risk	N	%	N	%	DALYs	N	%	DALYs	N	%	DALYs	N	%	DALYs	N	%	DALYs
Angola	Bengo	13	3	23.9	<0.5	3.5	<0.5	1	4.9	<0.5	<0.5	1.4	<0.5	<0.5	<0.05	<0.5	1	9.8	<0.5
Angola	Benguela	25	6	23.6	1	3.6	<0.5	1	4.8	<0.5	<0.5	1.4	<0.5	<0.5	<0.05	<0.5	2	9.8	<0.5
Angola	Cuanza Norte	13	3	22.6	<0.5	3.1	<0.5	1	4.5	<0.5	<0.5	1.3	<0.5	<0.5	<0.05	<0.5	1	8.9	<0.5
Angola	Huila	119	39	33.2	11	8.8	<0.5	10	8.4	1	3	2.4	<0.5	<0.5	0.1	<0.5	23	19.7	2
Angola	Kuando Kubango	205	47	22.7	6	3.0	<0.5	9	4.6	1	3	1.3	<0.5	<0.5	<0.05	<0.5	18	9.0	1
Angola	Lunda Norte	153	42	27.4	8	5.2	<0.5	9	5.9	1	3	1.7	<0.5	<0.5	<0.05	<0.5	20	12.8	1
Angola	Lunda sul	129	37	28.5	7	5.5	<0.5	8	6.5	1	2	1.8	<0.5	<0.5	<0.05	<0.5	18	13.8	1
Angola	Moxico 1	135	31	23.0	4	3.1	<0.5	6	4.7	1	2	1.4	<0.5	<0.5	<0.05	<0.5	13	9.3	1
Angola	Namibe	18	5	26.1	1	4.3	<0.5	1	5.7	<0.5	<0.5	1.7	<0.5	<0.5	<0.05	<0.5	2	11.7	<0.5
Angola	NY Benguela	57	15	27.2	3	5.1	<0.5	3	6.1	<0.5	1	1.8	<0.5	<0.5	<0.05	<0.5	7	13.0	1
Angola	NY Cuanza Norte	9	2	22.6	<0.5	2.4	<0.5	<0.5	4.8	<0.5	<0.5	1.5	<0.5	<0.5	<0.05	<0.5	1	8.7	<0.5
Angola	NY Huila	11	3	26.5	1	4.5	<0.5	1	6.0	<0.5	<0.5	1.8	<0.5	<0.5	<0.05	<0.5	1	12.4	<0.5
Angola	NY Lunda Norte	33	9	25.8	1	4.1	<0.5	2	5.6	<0.5	1	1.7	<0.5	<0.5	<0.05	<0.5	4	11.3	<0.5
Angola	NY Moxico 1	172	34	19.9	4	2.1	<0.5	6	3.8	1	2	1.2	<0.5	<0.5	<0.05	<0.5	12	7.0	1
Angola	P5Angola*#	131	29	22.4	3	2.6	<0.5	6	4.6	1	2	1.4	<0.5	<0.5	<0.05	<0.5	11	8.6	1
Angola	Uige	95	26	27.3	5	5.2	<0.5	6	5.9	1	2	1.7	<0.5	<0.5	<0.05	<0.5	12	12.7	1
Angola	Zaire	7	1	20.3	<0.5	2.1	<0.5	<0.5	4.0	<0.5	<0.5	1.3	<0.5	<0.5	<0.05	<0.5	1	7.4	<0.5
Burundi	Bururi	220	53	23.9	8	3.5	<0.5	11	4.8	1	3	1.4	<0.5	<0.5	<0.05	<0.5	22	9.8	2
Burundi	Cibitoke-Bubanza	526	173	32.9	43	8.1	<0.5	40	7.7	5	11	2.1	<0.5	<0.5	<0.05	<0.5	94	17.9	6
Burundi	P5Burundi*#	415	98	23.6	14	3.3	<0.5	20	4.9	3	6	1.5	<0.5	<0.5	<0.05	<0.5	40	9.7	3
Burundi	Rutana	171	35	20.4	4	2.1	<0.5	7	3.9	1	2	1.3	<0.5	<0.5	<0.05	<0.5	13	7.3	1
Cameroon	Adamaoua 1	287	88	30.6	22	7.8	<0.5	22	7.5	3	6	2.1	<0.5	8	2.7	17	58	20.1	20
Cameroon	Adamaoua 2	261	86	32.9	23	8.8	<0.5	21	8.0	3	6	2.1	<0.5	7	2.8	15	57	21.8	18
Cameroon	Centre 1	265	136	51.2	61	23.0	1	49	18.4	6	13	5.1	1	1	0.4	4	124	46.8	11
Cameroon	Centre 2	62	27	43.6	11	17.2	<0.5	9	14.4	1	3	4.1	<0.5	<0.5	0.3	1	22	36.0	2
Cameroon	Centre 3	201	80	39.9	27	13.4	<0.5	23	11.4	3	6	3.1	<0.5	<0.5	0.1	1	56	28.0	4
Cameroon	East	74	29	39.7	11	14.7	<0.5	10	13.0	1	3	3.6	<0.5	<0.5	0.3	1	23	31.6	2
Cameroon	Far North	174	33	18.8	3	1.8	<0.5	6	3.5	1	2	1.0	<0.5	2	1.2	3	13	7.6	4

(continued)

			Mf-positive		Palpable nodules			Reversible skin disease			Irreverisble skin disease			Eye disease (OED)			Total all manifestations		
Country	Project	Pop. at risk	N	%	N	%	DALYs	N	%	DALYs	N	%	DALYs	N	%	DALYs	N	%	DALYs
Cameroon	Littoral 1	174	92	52.6	42	24.0	<0.5	33	19.2	4	9	5.3	<0.5	1	0.4	3	85	48.9	8
Cameroon	Littoral 2	93	56	60.4	31	33.3	<0.5	25	27.3	3	7	7.9	<0.5	1	1.0	4	65	69.5	8
Cameroon	Northern	390	162	41.4	61	15.5	1	50	12.8	6	13	3.4	1	19	4.8	54	142	36.5	61
Cameroon	Northwest	507	223	44.1	78	15.5	1	62	12.2	8	17	3.3	1	1	0.1	2	157	31.1	12
Cameroon	P20Cameroon[*]	93	40	43.3	14	15.2	<0.5	11	12.0	1	3	3.2	<0.5	<0.5	0.1	<0.5	29	30.6	2
Cameroon	P5Cameroon[*#]	929	224	24.1	34	3.6	<0.5	48	5.2	6	14	1.5	1	<0.5	<0.05	1	97	10.4	8
Cameroon	South	185	60	32.4	15	8.1	<0.5	14	7.4	2	4	2.0	<0.5	<0.5	<0.05	<0.5	33	17.6	2
Cameroon	South West 1	243	143	59.0	72	29.6	1	56	23.2	7	16	6.5	1	1	0.5	5	145	59.8	13
Cameroon	South West 2	165	88	53.2	39	23.4	<0.5	30	18.2	4	8	5.0	<0.5	1	0.3	2	78	46.9	6
Cameroon	Western	1,016	673	66.3	391	38.5	4	318	31.3	38	90	8.9	5	11	1.1	48	811	79.9	95
CAR	CAR region 3	265	147	55.6	66	24.8	1	50	18.9	6	13	4.9	1	19	7.0	53	147	55.6	61
CAR	CAR region 4	276	102	36.8	31	11.4	<0.5	27	9.8	3	7	2.6	<0.5	10	3.5	22	75	27.3	26
CAR	CAR region 5	255	80	31.4	19	7.6	<0.5	19	7.3	2	5	2.1	<0.5	6	2.5	12	50	19.4	15
CAR	CAR region 6	393	181	46.0	69	17.7	1	55	13.9	7	14	3.7	1	20	5.1	52	158	40.3	61
CAR	P20CAR[*]	39	16	41.3	5	13.5	<0.5	4	10.9	1	1	2.9	<0.5	<0.5	0.1	<0.5	11	27.4	1
CAR	P5CAR[*#]	88	23	26.3	4	4.8	<0.5	5	5.9	1	2	1.7	<0.5	<0.5	<0.05	<0.5	11	12.5	1
Chad	Chad	1,068	326	30.5	75	7.1	1	72	6.7	9	20	1.8	1	25	2.3	47	192	17.9	57
Chad	P5Chad[*#]	133	36	27.3	8	5.8	<0.5	7	5.6	1	2	1.5	<0.5	3	1.9	4	20	14.8	6
Chad	P5Chad propext[*#]	76	17	22.3	2	3.1	<0.5	3	4.3	<0.5	1	1.2	<0.5	1	1.5	2	8	10.0	2
Congo	Congo 1	487	133	27.3	27	5.5	<0.5	29	6.0	4	8	1.7	<0.5	<0.5	<0.05	1	65	13.3	5
Congo	P20Congo[*]	21	12	56.7	5	24.7	<0.5	4	18.7	<0.5	1	5.0	<0.5	<0.5	0.3	<0.5	10	48.6	1
Congo	P5Congo[*#]	286	75	26.4	14	4.8	<0.5	18	6.3	2	5	1.8	<0.5	<0.5	0.1	<0.5	37	13.0	3
DRC	Bandundu	3	1	27.1	<0.5	4.9	<0.5	<0.5	6.3	<0.5	<0.5	1.9	<0.5	<0.5	<0.05	<0.5	<0.5	13.1	<0.5
DRC	Bas-Congo Kinshasa	803	298	37.0	89	11.1	1	77	9.6	10	21	2.6	1	1	0.1	2	188	23.3	13
DRC	Butembo-Beni	499	215	43.1	76	15.1	1	60	12.0	7	16	3.2	1	1	0.1	2	152	30.4	11
DRC	Equateur-Kiri	664	272	40.9	89	13.4	1	72	10.9	9	19	2.9	1	1	0.1	2	181	27.3	13
DRC	Ituri-Nord	671	423	63.0	246	36.7	3	205	30.5	24	59	8.8	4	9	1.3	38	519	77.3	68
DRC	Ituri-Sud	614	354	57.7	192	31.2	2	159	25.9	19	46	7.4	3	6	0.9	24	402	65.5	48
DRC	Kasai	5,756	2,210	38.4	801	13.9	9	722	12.5	88	207	3.6	10	17	0.3	72	1,748	30.4	179
DRC	Kasongo	721	228	31.6	59	8.2	1	59	8.1	7	17	2.3	1	1	0.1	2	135	18.7	11

(continued)

Country	Project	Pop. at risk	Mf-positive N	Mf-positive %	Palpable nodules N	Palpable nodules %	Palpable nodules DALYs	Reversible skin disease N	Reversible skin disease %	Reversible skin disease DALYs	Irreverisble skin disease N	Irreverisble skin disease %	Irreverisble skin disease DALYs	Eye disease (OED) N	Eye disease (OED) %	Eye disease (OED) DALYs	Total all manifestations N	Total all manifestations %	Total all manifestations DALYs
DRC	Katanga-Nord	335	126	37.6	45	13.4	<0.5	41	12.1	5	11	3.4	1	1	0.3	3	98	29.1	9
DRC	Katanga-Sud	371	141	38.0	50	13.6	1	45	12.2	6	13	3.5	1	1	0.3	4	110	29.6	11
DRC	Lualaba	120	48	39.7	16	13.2	<0.5	13	11.1	2	4	3.0	<0.5	<0.5	0.1	1	33	27.5	2
DRC	Lubutu	179	101	56.7	45	25.2	<0.5	34	19.2	4	9	5.2	<0.5	1	0.3	2	89	49.9	7
DRC	Masisi-Walikale	560	290	51.7	128	22.8	1	101	18.1	12	28	5.0	1	2	0.4	10	260	46.3	25
DRC	Mongala	779	286	36.7	86	11.1	1	74	9.4	9	21	2.6	1	1	0.1	3	181	23.3	14
DRC	NY Katanga-Nord	243	60	24.5	9	3.8	<0.5	13	5.4	2	4	1.6	<0.5	<0.5	<0.05	<0.5	26	10.9	2
DRC	NY Lualaba	526	146	27.7	27	5.1	<0.5	33	6.3	4	10	1.8	<0.5	<0.5	<0.05	<0.5	70	13.3	5
DRC	NY Masisi-Walikale	29	11	37.2	3	10.4	<0.5	3	9.0	<0.5	1	2.4	<0.5	<0.5	<0.05	<0.5	6	21.9	<0.5
DRC	NY Rutshuru-Ngoma	5	2	50.8	1	18.8	<0.5	1	13.8	<0.5	<0.5	3.7	<0.5	<0.5	0.1	<0.5	2	36.5	<0.5
DRC	NY Sankuru	251	70	27.9	15	5.9	<0.5	18	7.1	2	5	2.0	<0.5	<0.5	0.1	1	38	15.1	3
DRC	NY Ueles	85	50	59.0	28	32.7	<0.5	23	27.0	3	7	7.8	<0.5	1	1.0	4	58	68.5	7
DRC	P20DRC*	1,259	592	47.0	238	18.9	3	190	15.1	23	52	4.1	2	4	0.3	14	484	38.4	42
DRC	P5DRC*#	4,003	975	24.4	148	3.7	2	209	5.2	27	62	1.5	2	1	<0.05	2	419	10.5	32
DRC	Rutshuru-Ngoma	353	138	39.2	40	11.2	<0.5	32	9.2	4	9	2.5	<0.5	<0.5	<0.05	<0.5	81	22.9	5
DRC	Sankuru	571	270	47.2	133	23.2	1	116	20.3	14	34	6.0	2	5	0.8	20	287	50.3	37
DRC	Tshopo	853	438	51.4	200	23.4	2	161	18.9	19	45	5.3	2	4	0.5	17	410	48.1	41
DRC	Tshuapa	760	381	50.2	165	21.7	2	133	17.5	16	37	4.9	2	3	0.4	11	337	44.4	30
DRC	Ubangi-Nord	428	207	48.3	94	21.9	1	78	18.1	9	22	5.1	1	2	0.5	10	196	45.7	21
DRC	Ubangi-Sud	722	231	32.0	56	7.7	1	54	7.5	7	15	2.1	1	<0.5	<0.05	1	125	17.3	9
DRC	Ueles	842	512	60.8	288	34.3	3	239	28.3	28	69	8.2	4	10	1.2	44	606	72.0	79
Eq. Guinea	Bioko	59	44	74.8	32	53.4	<0.5	28	47.8	3	9	15.3	1	2	3.6	11	71	120.1	15
Eq. Guinea	P5EqGuinea*#	139	31	22.6	3	2.4	<0.5	7	4.8	1	2	1.5	<0.5	<0.5	<0.05	<0.5	12	8.7	1
Ethiopia	Assosa	317	84	26.5	15	4.7	<0.5	18	5.7	2	5	1.6	<0.5	<0.5	<0.05	<0.5	38	12.1	3
Ethiopia	Bench-Maji	420	162	38.7	49	11.6	1	40	9.5	5	11	2.6	<0.5	<0.5	0.1	1	100	23.7	7
Ethiopia	East Wellega	514	175	34.1	46	9.0	<0.5	42	8.2	5	12	2.3	<0.5	<0.5	0.1	1	101	19.6	7
Ethiopia	Gambella	61	22	36.4	6	10.3	<0.5	6	9.1	1	2	2.5	<0.5	<0.5	0.1	<0.5	13	22.0	1

(continued)

			Mf-positive		Palpable nodules			Reversible skin disease			Irreverisble skin disease			Eye disease (OED)			Total all manifestations		
Country	Project	Pop. at risk	N	%	N	%	DALYs	N	%	DALYs	N	%	DALYs	N	%	DALYs	N	%	DALYs
Ethiopia	Horo Guduru	30	10	34.7	3	9.0	<0.5	2	7.8	<0.5	1	2.1	<0.5	<0.5	<0.05	<0.5	6	19.0	<0.5
Ethiopia	Illubabor	435	169	38.9	54	12.3	1	44	10.0	5	12	2.7	<0.5	<0.5	0.1	1	109	25.2	8
Ethiopia	Jimma	513	145	28.2	29	5.6	<0.5	31	6.1	4	9	1.8	<0.5	<0.5	<0.05	<0.5	69	13.5	5
Ethiopia	Kaffa-Sheka	729	326	44.8	113	15.5	1	91	12.4	11	24	3.4	1	1	0.1	2	229	31.5	16
Ethiopia	Kamashi	271	102	37.7	33	12.3	<0.5	28	10.2	3	8	2.8	<0.5	<0.5	0.1	1	69	25.5	5
Ethiopia	Metekel	93	28	30.1	6	6.6	<0.5	6	6.7	1	2	1.9	<0.5	<0.5	<0.05	<0.5	14	15.2	1
Ethiopia	North Gondar	180	51	28.5	11	5.9	<0.5	12	6.6	1	3	1.9	<0.5	<0.5	<0.05	<0.5	26	14.4	2
Ethiopia	NY East Wellega	165	63	38.5	20	12.2	<0.5	16	9.8	2	4	2.7	<0.5	<0.5	0.1	<0.5	41	24.8	3
Ethiopia	NY West Wellega	167	41	24.8	7	4.2	<0.5	8	5.0	1	2	1.5	<0.5	<0.5	<0.05	<0.5	18	10.7	1
Ethiopia	P20Ethiopia*	191	81	42.1	27	14.2	<0.5	21	11.2	3	6	3.0	<0.5	<0.5	0.1	1	55	28.5	4
Ethiopia	P5Ethiopia*	2,018	494	24.5	75	3.7	1	103	5.1	13	30	1.5	1	<0.5	<0.05	1	209	10.4	16
Ethiopia	West Shewa	33	7	22.1	1	2.7	<0.5	1	4.5	<0.5	<0.5	1.3	<0.5	<0.5	<0.05	<0.5	3	8.5	<0.5
Ethiopia	West Wellega	590	244	41.3	86	14.6	1	72	12.2	9	20	3.4	1	1	0.2	3	179	30.3	14
Gabon	P5Gabon*#	49	11	21.6	1	2.5	<0.5	2	4.3	<0.5	1	1.3	<0.5	<0.5	<0.05	<0.5	4	8.1	<0.5
Liberia	All_Liberia	881	343	39.0	100	11.3	1	72	8.2	9	19	2.1	1	<0.5	<0.05	<0.5	191	21.7	11
Malawi	Malawi Extension	665	136	20.4	15	2.2	<0.5	26	3.9	3	8	1.2	<0.5	<0.5	<0.05	<0.5	49	7.4	4
Malawi	Thyolo Mwanza	507	137	27.0	26	5.2	<0.5	28	5.5	4	8	1.6	<0.5	<0.5	<0.05	<0.5	62	12.3	4
Mozamb.	P20Mozambique*	9	2	20.4	<0.5	2.2	<0.5	<0.5	4.0	<0.5	<0.5	1.2	<0.5	<0.5	<0.05	<0.5	1	7.4	<0.5
Mozamb.	P5Mozambique*	27	5	19.9	1	2.0	<0.5	1	3.9	<0.5	<0.5	1.2	<0.5	<0.5	<0.05	<0.5	2	7.1	<0.5
Nigeria	Adamawa	1,019	241	23.7	34	3.4	<0.5	49	4.9	6	14	1.4	1	17	1.7	27	115	11.3	35
Nigeria	Akwa Ibom	17	4	21.6	<0.5	2.5	<0.5	1	4.3	<0.5	<0.5	1.3	<0.5	<0.5	<0.05	<0.5	1	8.1	<0.5
Nigeria	Bauchi	1,044	225	21.5	26	2.5	<0.5	45	4.3	6	14	1.3	<0.5	16	1.5	26	101	9.6	33
Nigeria	Benue	2,058	783	38.0	239	11.6	3	203	9.9	25	56	2.7	2	2	0.1	5	500	24.3	36
Nigeria	Borno	820	160	19.5	18	2.2	<0.5	29	3.5	4	9	1.1	<0.5	10	1.3	16	66	8.1	20
Nigeria	Cross River	744	222	29.8	55	7.4	1	55	7.4	7	16	2.1	1	1	0.1	2	127	17.1	10
Nigeria	Edo Delta	956	266	27.8	52	5.4	1	57	5.9	7	16	1.7	1	<0.5	<0.05	<0.5	125	13.1	9
Nigeria	Ekiti	1,291	265	20.5	28	2.2	<0.5	52	4.0	7	16	1.2	1	<0.5	<0.05	<0.5	95	7.4	8
Nigeria	Enugu Anambra Ebony	1,421	691	48.6	273	19.2	3	211	14.9	26	57	4.0	3	3	0.2	10	545	38.3	42

(continued)

Country	Project	Pop. at risk	Mf-positive N	Mf-positive %	Palpable nodules N	Palpable nodules %	Palpable nodules DALYs	Reversible skin disease N	Reversible skin disease %	Reversible skin disease DALYs	Irreverisble skin disease N	Irreverisble skin disease %	Irreverisble skin disease DALYs	Eye disease (OED) N	Eye disease (OED) %	Eye disease (OED) DALYs	Total all manifestations N	Total all manifestations %	Total all manifestations DALYs
Nigeria	FCT	302	67	22.3	8	2.6	<0.5	14	4.7	2	4	1.4	<0.5	<0.5	<0.05	<0.5	26	8.7	2
Nigeria	Gombe	1,134	261	23.1	35	3.1	<0.5	54	4.8	7	16	1.4	1	20	1.7	33	125	11.0	41
Nigeria	Imo Abia	783	226	28.8	49	6.3	1	52	6.7	7	15	1.9	1	<0.5	<0.05	1	117	14.9	8
Nigeria	Jigawa	212	45	21.1	5	2.2	<0.5	9	4.2	1	3	1.2	<0.5	3	1.5	5	19	9.1	6
Nigeria	Kaduna	1,650	393	23.8	57	3.4	1	84	5.1	11	24	1.5	1	29	1.8	50	194	11.7	62
Nigeria	Kano	589	121	20.5	13	2.2	<0.5	23	4.0	3	7	1.2	<0.5	8	1.4	13	52	8.8	17
Nigeria	Kebbi	124	24	19.3	2	1.9	<0.5	5	3.7	1	1	1.1	<0.5	2	1.3	3	10	8.0	3
Nigeria	Kogi	1,063	275	25.9	48	4.5	1	58	5.5	7	17	1.6	1	<0.5	<0.05	<0.5	124	11.6	9
Nigeria	Kwara	898	223	24.8	37	4.1	<0.5	45	5.0	6	13	1.4	<0.5	16	1.7	26	110	12.2	32
Nigeria	Niger	1,579	332	21.0	39	2.5	<0.5	65	4.1	8	19	1.2	1	23	1.4	35	145	9.2	44
Nigeria	Ogun	205	57	27.9	11	5.4	<0.5	12	5.9	2	3	1.7	<0.5	<0.5	<0.05	<0.5	27	13.0	2
Nigeria	Ondo	814	206	25.3	35	4.3	<0.5	42	5.2	5	12	1.5	<0.5	<0.5	<0.05	<0.5	90	11.0	7
Nigeria	Osun	960	211	22.0	29	3.0	<0.5	41	4.3	5	12	1.3	<0.5	<0.5	<0.05	<0.5	82	8.6	6
Nigeria	Oyo	655	149	22.8	23	3.5	<0.5	28	4.3	4	8	1.3	<0.5	10	1.5	15	69	10.5	19
Nigeria	P20Nigeria*	132	54	41.0	17	12.9	<0.5	14	10.3	2	4	2.7	<0.5	<0.5	0.1	<0.5	34	26.0	2
Nigeria	P5Nigeria*#	4,491	930	20.7	105	2.3	1	183	4.1	23	54	1.2	2	62	1.4	95	404	9.0	122
Nigeria	Plateau	911	227	24.9	35	3.8	<0.5	48	5.3	6	13	1.5	<0.5	17	1.9	28	113	12.4	35
Nigeria	Nassarawa Plateau	922	279	30.3	66	7.2	1	62	6.8	8	17	1.9	1	22	2.3	40	167	18.1	49
Nigeria	Nassarawa LF Taraba	991	310	31.2	80	8.0	1	77	7.8	10	22	2.2	1	28	2.8	62	206	20.8	73
Nigeria	Yobe	366	70	19.1	7	1.9	<0.5	13	3.6	2	4	1.0	<0.5	5	1.2	7	28	7.7	9
Nigeria	Zamfara	170	36	21.1	4	2.2	<0.5	7	4.2	1	2	1.2	<0.5	3	1.5	4	15	9.1	5
South Sudan	East Bahr El Ghazal	326	105	32.1	27	8.3	<0.5	24	7.2	3	6	2.0	<0.5	8	2.4	15	65	19.9	19
South Sudan	East Equatoria	580	180	31.0	44	7.6	<0.5	40	6.9	5	11	1.9	<0.5	14	2.4	26	109	18.8	32
South Sudan	P20SouthSudan*	16	6	37.7	2	10.8	<0.5	1	8.8	<0.5	<0.5	2.3	<0.5	<0.5	3.0	1	4	25.0	1
South Sudan	P5SouthSudan*#	450	91	20.2	10	2.2	<0.5	17	3.9	2	5	1.1	<0.5	6	1.4	9	39	8.7	11
South Sudan	Upper Nile	304	75	24.6	12	3.9	<0.5	16	5.1	2	5	1.5	<0.5	6	1.8	10	38	12.4	12
South Sudan	West Bahr El Ghazal	1,758	525	29.8	137	7.8	1	132	7.5	16	36	2.1	2	47	2.7	120	353	20.1	139

(continued)

Country	Project	Pop. at risk	Mf-positive		Palpable nodules			Reversible skin disease			Irreverisble skin disease			Eye disease (OED)			Total all manifestations		
			N	%	N	%	DALYs	N	%	DALYs	N	%	DALYs	N	%	DALYs	N	%	DALYs
South Sudan	West Equatoria	415	141	33.9	38	9.2	<0.5	33	7.8	4	9	2.2	<0.5	11	2.7	22	91	21.9	27
Sudan	P5Sudan*#	127	29	22.6	4	2.9	<0.5	6	4.7	1	2	1.4	<0.5	2	1.7	3	13	10.6	4
Sudan	Sudan	120	25	21.1	3	2.2	<0.5	5	4.2	1	1	1.2	<0.5	2	1.5	3	11	9.1	4
Sudan	Sudan Abu Hamed	120	25	21.1	3	2.2	<0.5	5	4.2	1	1	1.2	<0.5	2	1.5	3	11	9.1	4
Tanzania	Kilosa	287	80	27.8	14	4.8	<0.5	19	6.5	2	5	1.9	<0.5	<0.5	<0.05	<0.5	38	13.2	3
Tanzania	Mahenge	287	127	44.2	48	16.8	1	39	13.7	5	11	3.8	1	1	0.3	3	99	34.6	9
Tanzania	Morogoro	205	58	28.1	11	5.6	<0.5	13	6.2	2	4	1.8	<0.5	<0.5	<0.05	<0.5	28	13.5	2
Tanzania	P5Tanzania*	520	135	25.9	23	4.4	<0.5	30	5.7	4	9	1.7	<0.5	<0.5	<0.05	<0.5	61	11.8	5
Tanzania	Ruvuma	227	127	56.2	64	28.3	1	52	23.0	6	15	6.5	1	2	0.7	7	133	58.5	15
Tanzania	Tanga	178	47	26.3	8	4.5	<0.5	10	5.7	1	3	1.6	<0.5	<0.5	<0.05	<0.5	21	11.8	2
Tanzania	Tukuyu	65	25	38.9	8	13.0	<0.5	7	11.0	1	2	3.0	<0.5	<0.5	0.1	<0.5	18	27.2	1
Tanzania	Tunduru	71	33	46.7	13	18.0	<0.5	10	14.3	1	3	3.9	<0.5	<0.5	0.2	1	26	36.4	2
Uganda	P5Uganda*	131	31	23.4	4	3.2	<0.5	6	4.9	1	2	1.5	<0.5	<0.5	<0.05	<0.5	13	9.6	1
Uganda	Phase 1	205	58	28.3	12	5.6	<0.5	13	6.5	2	4	1.9	<0.5	<0.5	<0.05	<0.5	29	14.0	2
Uganda	Phase 2	399	90	22.6	10	2.4	<0.5	19	4.8	2	6	1.5	<0.5	<0.5	<0.05	<0.5	35	8.7	3
Uganda	Phase 3	736	336	45.7	145	19.7	2	123	16.7	15	35	4.7	2	3	0.5	14	306	41.6	32
Uganda	Phase 4	416	114	27.4	21	5.1	<0.5	26	6.2	3	7	1.8	<0.5	<0.5	<0.05	<0.5	55	13.1	4
Uganda	Phase 5	268	79	29.6	18	6.8	<0.5	20	7.3	2	6	2.1	<0.5	<0.5	0.1	<0.5	44	16.2	3
**Total**	**Total**	**79,768**	**25,964**	**32.5**	**7,617**	**9.5**	**81**	**7,205**	**9.0**	**890**	**2,041**	**2.6**	**91**	**616**	**0.8**	**1,410**	**17,478**	**21.9**	**2,472**

* P5 and P20 APOC-projects are a compilation of multiple areas within a country with a prevalence of palpable nodule of $\geq 5\% - < 20\%$ and $\geq 20\%$ respectively.

Onchocerciasis hypoendemic areas with confirmed *L. loa* co-endemicity

Table V: Population at risk, number of cases with infection and clinical manifestations, and DALYs lost due to onchocerciasis by country and APOC-project for 2020. Absolute numbers (N and DALYs) are presented in thousands.

			Mf-positive		Palpable nodules			Reversible skin disease			Irreveristble skin disease			Eye disease (OED)			Total all manifestations		
Country	Project	Pop. at risk	N	%	N	%	DALYs	N	%	DALYs	N	%	DALYs	N	%	DALYs	N	%	DALYs
Angola	Bengo	30	1	3.4	<0.5	0.6	<0.5	<0.5	0.3	<0.5	<0.5	0.9	<0.5	<0.5	<0.05	<0.5	1	1.8	<0.5
Angola	Benguela	57	4	7.6	1	1.3	<0.5	<0.5	0.7	<0.5	1	0.9	<0.5	<0.5	<0.05	<0.5	2	2.9	<0.5
Angola	Cuanza Norte	29	1	4.0	<0.5	0.7	<0.5	<0.5	0.3	<0.5	<0.5	0.8	<0.5	<0.5	<0.05	<0.5	1	1.8	<0.5
Angola	Huila	268	19	7.1	6	2.1	<0.5	2	0.7	<0.5	4	1.4	<0.5	<0.5	<0.05	<0.5	11	4.2	1
Angola	Kuando Kubango	463	6	1.3	1	0.2	<0.5	1	0.1	<0.5	3	0.7	<0.5	<0.5	<0.05	<0.5	5	1.0	<0.5
Angola	Lunda Norte	346	9	2.6	2	0.5	<0.5	1	0.2	<0.5	3	0.9	<0.5	<0.5	<0.05	<0.5	6	1.7	<0.5
Angola	Lunda sul	291	8	2.6	1	0.5	<0.5	1	0.2	<0.5	3	1.0	<0.5	<0.5	<0.05	<0.5	5	1.7	<0.5
Angola	Moxico 1	304	11	3.7	2	0.5	<0.5	1	0.3	<0.5	2	0.8	<0.5	<0.5	<0.05	<0.5	5	1.7	<0.5
Angola	Namibe	40	10	23.9	2	4.2	<0.5	2	4.5	<0.5	1	1.6	<0.5	<0.5	<0.05	<0.5	4	10.2	<0.5
Angola	NY Benguela	128	32	24.8	6	4.9	<0.5	6	4.7	1	2	1.6	<0.5	<0.5	<0.05	<0.5	14	11.2	1
Angola	NY Cuanza Norte	21	4	20.4	<0.5	2.3	<0.5	1	3.7	<0.5	<0.5	1.4	<0.5	<0.5	<0.05	<0.5	2	7.4	<0.5
Angola	NY Huila	26	6	24.4	1	4.3	<0.5	1	4.7	<0.5	<0.5	1.7	<0.5	<0.5	<0.05	<0.5	3	10.7	<0.5
Angola	NY Lunda Norte	75	18	23.8	3	4.0	<0.5	3	4.5	<0.5	1	1.5	<0.5	<0.5	<0.05	<0.5	8	10.1	1
Angola	NY Moxico 1	388	62	16.1	7	1.7	<0.5	10	2.6	1	4	1.0	<0.5	<0.5	<0.05	<0.5	21	5.4	2
Angola	P5Angola*#	296	63	21.2	7	2.4	<0.5	13	4.3	2	4	1.3	<0.5	<0.5	<0.05	<0.5	24	8.0	2
Angola	Uige	214	52	24.4	10	4.8	<0.5	10	4.5	1	3	1.5	<0.5	<0.5	<0.05	<0.5	23	10.9	2
Angola	Zaire	16	3	16.7	<0.5	1.8	<0.5	<0.5	2.7	<0.5	<0.5	1.1	<0.5	<0.5	<0.05	<0.5	1	5.6	<0.5
Burundi	Bururi	497	4	0.9	1	0.2	<0.5	<0.5	0.1	<0.5	3	0.7	<0.5	<0.5	<0.05	<0.5	5	0.9	<0.5
Burundi	Cibitoke-Bubanza	1,187	11	0.9	4	0.3	<0.5	1	0.1	<0.5	12	1.0	<0.5	<0.5	<0.05	<0.5	17	1.4	1
Burundi	P5Burundi*#	936	203	21.7	28	3.0	<0.5	42	4.5	5	13	1.3	<0.5	<0.5	<0.05	<0.5	83	8.9	7
Burundi	Rutana	387	2	0.5	<0.5	0.1	<0.5	<0.5	0.1	<0.5	2	0.5	<0.5	<0.5	<0.05	<0.5	3	0.7	<0.5
Cameroon	Adamaoua 1	648	9	1.4	3	0.5	<0.5	1	0.1	<0.5	6	0.9	<0.5	7	1.1	10	17	2.6	10
Cameroon	Adamaoua 2	589	2	0.4	1	0.1	<0.5	<0.5	<0.05	<0.5	5	0.8	<0.5	5	0.9	6	11	1.8	6
Cameroon	Centre 1	598	14	2.4	5	0.9	<0.5	1	0.2	<0.5	12	2.0	1	1	0.1	1	19	3.1	2
Cameroon	Centre 2	141	4	3.2	2	1.2	<0.5	<0.5	0.3	<0.5	2	1.7	<0.5	<0.5	0.1	<0.5	5	3.3	1
Cameroon	Centre 3	455	3	0.6	1	0.2	<0.5	<0.5	0.1	<0.5	5	1.1	<0.5	<0.5	<0.05	<0.5	7	1.5	<0.5
Cameroon	East	166	6	3.4	3	1.6	<0.5	1	0.4	<0.5	3	1.8	<0.5	<0.5	0.1	<0.5	6	3.9	1
Cameroon	Far North	393	1	0.2	<0.5	<0.05	<0.5	<0.5	<0.05	<0.5	2	0.4	<0.5	2	0.4	1	3	0.9	2

(continued)

Country	Project	Pop. at risk	Mf-positive		Palpable nodules			Reversible skin disease			Irreverisble skin disease			Eye disease (OED)			Total all manifestations		
			N	%	N	%	DALYs	N	%	DALYs	N	%	DALYs	N	%	DALYs	N	%	DALYs
Cameroon	Littoral 1	393	49	12.5	18	4.7	<0.5	5	1.3	1	11	2.8	<0.5	1	0.1	2	35	8.9	3
Cameroon	Littoral 2	209	17	7.9	7	3.2	<0.5	1	0.5	<0.5	6	3.0	<0.5	<0.5	0.2	2	14	6.9	2
Cameroon	Northern	881	9	1.0	4	0.4	<0.5	1	0.1	<0.5	10	1.2	<0.5	13	1.4	22	27	3.1	23
Cameroon	Northwest	1,144	18	1.6	7	0.6	<0.5	2	0.1	<0.5	17	1.4	1	<0.5	<0.05	1	26	2.3	2
Cameroon	P20Cameroon*	211	64	30.5	25	11.8	<0.5	10	4.5	1	6	2.7	<0.5	<0.5	0.1	1	40	19.1	3
Cameroon	P5Cameroon*#	2,097	482	23.0	72	3.4	1	102	4.9	13	31	1.5	1	<0.5	<0.05	1	205	9.8	16
Cameroon	South	418	4	1.0	1	0.3	<0.5	<0.5	0.1	<0.5	4	0.9	<0.5	<0.5	<0.05	<0.5	6	1.4	<0.5
Cameroon	South West 1	548	16	3.0	7	1.2	<0.5	1	0.2	<0.5	13	2.4	1	1	0.1	1	22	3.9	2
Cameroon	South West 2	373	4	1.0	2	0.5	<0.5	<0.5	0.1	<0.5	7	2.0	<0.5	<0.5	0.1	1	10	2.6	1
Cameroon	Western	2,292	127	5.5	50	2.2	1	10	0.4	2	82	3.6	4	6	0.3	17	148	6.5	24
CAR	CAR region 3	598	17	2.9	5	0.8	<0.5	1	0.1	<0.5	9	1.6	<0.5	12	2.0	19	26	4.4	20
CAR	CAR region 4	623	5	0.8	1	0.2	<0.5	<0.5	<0.05	<0.5	5	0.8	<0.5	6	0.9	7	12	2.0	7
CAR	CAR region 5	576	2	0.3	<0.5	0.1	<0.5	<0.5	<0.05	<0.5	3	0.6	<0.5	4	0.7	4	8	1.3	4
CAR	CAR region 6	887	18	2.0	4	0.5	<0.5	1	0.1	<0.5	10	1.2	<0.5	12	1.3	17	27	3.1	18
CAR	P20CAR*	87	34	39.2	12	13.4	<0.5	8	8.9	1	2	2.8	<0.5	<0.5	0.1	<0.5	22	25.3	1
CAR	P5CAR*#	198	50	25.3	9	4.6	<0.5	11	5.6	1	3	1.6	<0.5	<0.5	<0.05	<0.5	24	11.9	2
Chad	Chad	2,409	1	<0.05	<0.5	<0.05	<0.5	<0.5	<0.05	<0.5	14	0.6	<0.5	14	0.6	14	28	1.2	15
Chad	P5Chad*#	300	74	24.7	16	5.3	<0.5	15	5.1	2	4	1.4	<0.5	5	1.7	9	40	13.5	12
Chad	P5Chad propext*#	172	34	20.0	5	2.7	<0.5	7	3.8	1	2	1.1	<0.5	2	1.3	4	15	8.9	5
Congo	Congo 1	1,099	3	0.3	1	0.1	<0.5	<0.5	<0.05	<0.5	7	0.7	<0.5	<0.5	<0.05	<0.5	9	0.8	1
Congo	P20Congo*	48	18	37.4	8	17.3	<0.5	3	5.5	<0.5	2	4.0	<0.5	<0.5	0.2	<0.5	13	26.9	1
Congo	P5Congo*#	645	166	25.7	31	4.7	<0.5	40	6.1	5	12	1.8	<0.5	<0.5	<0.05	1	82	12.7	6
DRC	Bandundu	8	<0.5	0.5	<0.5	0.2	<0.5	<0.5	0.1	<0.5	<0.5	0.8	<0.5	<0.5	<0.05	<0.5	<0.5	1.0	<0.5
DRC	Bas-Congo Kinshasa	1,813	57	3.1	17	0.9	<0.5	5	0.3	1	23	1.2	1	<0.5	<0.05	1	44	2.4	3
DRC	Butembo-Beni	1,127	127	11.3	45	4.0	<0.5	13	1.2	2	22	2.0	1	1	0.1	2	81	7.2	5
DRC	Equateur-Kiri	1,497	52	3.5	21	1.4	<0.5	6	0.4	1	23	1.5	1	1	<0.05	1	50	3.4	3
DRC	Ituri-Nord	1,515	306	20.2	172	11.4	2	39	2.6	7	77	5.1	4	8	0.5	28	297	19.6	41
DRC	Ituri-Sud	1,385	462	33.4	255	18.4	3	78	5.7	12	71	5.2	4	7	0.5	26	412	29.7	45
DRC	Kasai	12,990	608	4.7	260	2.0	3	55	0.4	9	212	1.6	10	12	0.1	38	538	4.1	60
DRC	Kasongo	1,627	81	5.0	25	1.6	<0.5	8	0.5	1	21	1.3	1	1	<0.05	2	55	3.4	4

(continued)

Country	Project	Pop. at risk	Mf-positive		Palpable nodules			Reversible skin disease			Irreverisble skin disease			Eye disease (OED)			Total all manifestations		
			N	%	N	%	DALYs	N	%	DALYs	N	%	DALYs	N	%	DALYs	N	%	DALYs
DRC	Katanga-Nord	756	37	4.9	17	2.2	<0.5	4	0.5	1	13	1.8	1	1	0.1	2	35	4.6	4
DRC	Katanga-Sud	837	52	6.2	23	2.7	<0.5	6	0.7	1	16	1.9	1	1	0.1	3	45	5.3	5
DRC	Lualaba	272	7	2.5	3	1.0	<0.5	1	0.3	<0.5	4	1.5	<0.5	<0.5	<0.05	<0.5	8	2.8	1
DRC	Lubutu	403	60	14.8	24	6.1	<0.5	6	1.6	1	12	3.1	1	1	0.1	1	44	10.8	3
DRC	Masisi-Walikale	1,265	200	15.8	91	7.2	1	24	1.9	4	39	3.0	2	2	0.2	8	156	12.3	15
DRC	Mongala	1,758	59	3.4	23	1.3	<0.5	6	0.3	1	24	1.3	1	1	<0.05	2	54	3.0	4
DRC	NY Katanga-Nord	549	91	16.5	16	2.9	<0.5	11	2.0	2	7	1.3	<0.5	<0.5	<0.05	<0.5	34	6.2	2
DRC	NY Lualaba	1,187	229	19.3	46	3.9	<0.5	28	2.4	4	18	1.5	1	<0.5	<0.05	<0.5	93	7.8	6
DRC	NY Masisi-Walikale	66	17	26.5	5	8.0	<0.5	2	3.5	<0.5	1	2.0	<0.5	<0.5	<0.05	<0.5	9	13.6	1
DRC	NY Rutshuru-Ngoma	10	2	24.3	1	9.4	<0.5	<0.5	2.7	<0.5	<0.5	2.6	<0.5	<0.5	0.1	<0.5	2	14.8	<0.5
DRC	NY Sankuru	566	83	14.6	21	3.6	<0.5	10	1.7	2	9	1.5	<0.5	<0.5	0.1	1	39	6.9	3
DRC	NY Ueles	193	81	42.1	47	24.3	<0.5	17	9.0	3	12	6.0	1	1	0.7	5	77	39.9	9
DRC	P20DRC*	2,841	1,268	44.6	532	18.7	6	352	12.4	45	114	4.0	5	8	0.3	28	1,005	35.4	84
DRC	P5DRC*#	9,033	2,113	23.4	321	3.6	3	448	5.0	57	134	1.5	5	2	<0.05	4	905	10.0	70
DRC	Rutshuru-Ngoma	796	28	3.5	9	1.1	<0.5	3	0.3	<0.5	10	1.3	<0.5	<0.5	<0.05	<0.5	21	2.7	1
DRC	Sankuru	1,289	98	7.6	48	3.7	1	9	0.7	2	35	2.7	2	3	0.2	11	96	7.4	15
DRC	Tshopo	1,925	296	15.4	124	6.4	1	33	1.7	5	56	2.9	3	4	0.2	12	217	11.3	21
DRC	Tshuapa	1,715	264	15.4	105	6.1	1	30	1.8	5	49	2.9	2	3	0.2	9	187	10.9	17
DRC	Ubangi-Nord	966	157	16.2	71	7.4	1	19	2.0	3	28	2.9	1	2	0.2	8	121	12.5	13
DRC	Ubangi-Sud	1,629	80	4.9	21	1.3	<0.5	7	0.4	1	19	1.2	1	<0.5	<0.05	1	47	2.9	3
DRC	Ueles	1,900	268	14.1	122	6.4	1	23	1.2	4	72	3.8	4	6	0.3	22	222	11.7	31
Eq. Guinea	Bioko	134	<0.5	<0.05	<0.5	<0.05	<0.5	<0.5	<0.05	<0.5	4	2.9	<0.5	<0.5	0.3	1	4	3.2	1
Eq. Guinea	P5EqGuinea*#	314	<0.5	<0.05	<0.5	<0.05	<0.5	<0.5	<0.05	<0.5	1	0.4	<0.5	<0.5	<0.05	<0.5	1	0.4	<0.5
Ethiopia	Assosa	716	123	17.2	32	4.5	<0.5	30	4.2	4	11	1.5	<0.5	<0.5	<0.05	<0.5	74	10.3	5
Ethiopia	Bench-Maji	947	6	0.6	2	0.2	<0.5	1	0.1	<0.5	10	1.1	<0.5	<0.5	<0.05	<0.5	13	1.4	1
Ethiopia	East Wellega	1,160	9	0.8	4	0.3	<0.5	1	0.1	<0.5	12	1.0	<0.5	<0.5	<0.05	<0.5	17	1.5	1
Ethiopia	Gambella	139	1	0.9	<0.5	0.3	<0.5	<0.5	0.1	<0.5	2	1.1	<0.5	<0.5	<0.05	<0.5	2	1.5	<0.5

(continued)

Country	Project	Pop. at risk	Mf-positive		Palpable nodules			Reversible skin disease			Irreverisble skin disease			Eye disease (OED)			Total all manifestations		
			N	%	N	%	DALYs	N	%	DALYs	N	%	DALYs	N	%	DALYs	N	%	DALYs
Ethiopia	Horo Guduru	68	22	32.0	6	8.7	<0.5	4	6.0	1	1	2.0	<0.5	<0.5	<0.05	<0.5	11	16.8	1
Ethiopia	Illubabor	981	6	0.6	3	0.3	<0.5	1	0.1	<0.5	11	1.1	<0.5	<0.5	<0.05	<0.5	15	1.5	1
Ethiopia	Jimma	1,158	1	0.1	1	0.1	<0.5	<0.5	<0.05	<0.5	8	0.7	<0.5	<0.5	<0.05	<0.5	9	0.8	<0.5
Ethiopia	Kaffa-Sheka	1,645	7	0.4	3	0.2	<0.5	1	<0.05	<0.5	21	1.3	1	<0.5	<0.05	1	25	1.5	2
Ethiopia	Kamashi	612	55	9.0	52	8.4	1	20	3.2	3	14	2.2	1	1	0.1	2	85	13.9	6
Ethiopia	Metekel	209	2	0.8	<0.5	0.2	<0.5	<0.5	0.1	<0.5	2	0.8	<0.5	<0.5	<0.05	<0.5	2	1.1	<0.5
Ethiopia	North Gondar	406	2	0.5	1	0.2	<0.5	<0.5	0.1	<0.5	3	0.8	<0.5	<0.5	<0.05	<0.5	4	1.0	<0.5
Ethiopia	NY East Wellega	371	96	25.8	34	9.2	<0.5	13	3.4	2	8	2.2	<0.5	<0.5	0.1	1	55	14.9	3
Ethiopia	NY West Wellega	377	18	4.8	9	2.5	<0.5	5	1.3	1	4	1.1	<0.5	<0.5	<0.05	<0.5	18	4.9	1
Ethiopia	P20Ethiopia*	431	168	39.0	60	14.0	1	38	8.8	5	12	2.8	1	<0.5	0.1	1	111	25.7	8
Ethiopia	P5Ethiopia*	4,553	1,059	23.3	161	3.5	2	219	4.8	28	66	1.5	2	1	<0.05	2	448	9.8	34
Ethiopia	West Shewa	75	16	21.0	2	2.6	<0.5	3	4.2	<0.5	1	1.3	<0.5	<0.5	<0.05	<0.5	6	8.1	<0.5
Ethiopia	West Wellega	1,332	22	1.7	10	0.7	<0.5	3	0.2	<0.5	21	1.6	1	1	<0.05	1	34	2.5	3
Gabon	P5Gabon*#	112	22	20.0	3	2.3	<0.5	4	3.8	1	1	1.2	<0.5	<0.5	<0.05	<0.5	8	7.3	1
Liberia	All_Liberia	1,987	5	0.3	2	0.1	<0.5	1	<0.05	<0.5	13	0.7	<0.5	<0.5	<0.05	<0.5	15	0.8	1
Malawi	Malawi Extension	1,501	1	0.1	<0.5	<0.05	<0.5	<0.5	<0.05	<0.5	7	0.5	<0.5	<0.5	<0.05	<0.5	7	0.5	<0.5
Malawi	Thyolo Mwanza	1,145	<0.5	<0.05	<0.5	<0.05	<0.5	<0.5	<0.05	<0.5	6	0.5	<0.5	<0.5	<0.05	<0.5	6	0.5	<0.5
Mozamb.	P20Mozambique*	21	4	16.8	<0.5	1.9	<0.5	1	2.8	<0.5	<0.5	1.0	<0.5	<0.5	<0.05	<0.5	1	5.7	<0.5
Mozamb.	P5Mozambique*	61	11	17.3	1	1.7	<0.5	2	3.3	<0.5	1	1.0	<0.5	<0.5	<0.05	<0.5	4	6.0	<0.5
Nigeria	Adamawa	2,298	<0.5	<0.05	<0.5	<0.05	<0.5	<0.5	<0.05	<0.5	11	0.5	<0.5	10	0.4	8	21	0.9	9
Nigeria	Akwa Ibom	39	<0.5	0.2	<0.5	0.1	<0.5	<0.5	<0.05	<0.5	<0.5	0.6	<0.5	<0.5	<0.05	<0.5	<0.5	0.6	<0.5
Nigeria	Bauchi	2,355	5	0.2	1	<0.05	<0.5	1	<0.05	<0.5	11	0.5	<0.5	12	0.5	11	25	1.1	12
Nigeria	Benue	4,644	41	0.9	16	0.3	<0.5	4	0.1	1	51	1.1	2	1	<0.05	2	72	1.5	5
Nigeria	Borno	1,851	<0.5	<0.05	<0.5	<0.05	<0.5	<0.5	<0.05	<0.5	5	0.3	<0.5	6	0.3	4	11	0.6	4
Nigeria	Cross River	1,679	1	<0.05	<0.5	<0.05	<0.5	<0.5	<0.05	<0.5	10	0.6	<0.5	<0.5	<0.05	<0.5	11	0.7	1
Nigeria	Edo Delta	2,158	<0.5	<0.05	<0.5	<0.05	<0.5	<0.5	<0.05	<0.5	11	0.5	<0.5	<0.5	<0.05	<0.5	11	0.5	<0.5
Nigeria	Ekiti	2,913	1	<0.05	<0.5	<0.05	<0.5	<0.5	<0.05	<0.5	11	0.4	<0.5	<0.5	<0.05	<0.5	11	0.4	<0.5
Nigeria	Enugu Anambra Ebony	3,207	5	0.2	3	0.1	<0.5	<0.5	<0.05	<0.5	40	1.2	2	1	<0.05	2	44	1.4	4

(continued)

Country	Project	Pop. at risk	Mf-positive N	Mf-positive %	Palpable nodules N	Palpable nodules %	Palpable nodules DALYs	Reversible skin disease N	Reversible skin disease %	Reversible skin disease DALYs	Irreverisble skin disease N	Irreverisble skin disease %	Irreverisble skin disease DALYs	Eye disease (OED) N	Eye disease (OED) %	Eye disease (OED) DALYs	Total all manifestations N	Total all manifestations %	Total all manifestations DALYs
Nigeria	FCT	681	<0.5	<0.05	<0.5	<0.05	<0.5	<0.5	<0.05	<0.5	3	0.4	<0.5	<0.5	<0.05	<0.5	3	0.5	<0.5
Nigeria	Gombe	2,559	2	0.1	1	<0.05	<0.5	<0.5	<0.05	<0.5	12	0.5	<0.5	13	0.5	10	26	1.0	11
Nigeria	Imo Abia	1,768	5	0.3	2	0.1	<0.5	1	<0.05	<0.5	11	0.6	<0.5	<0.5	<0.05	<0.5	13	0.7	1
Nigeria	Jigawa	479	<0.5	0.1	<0.5	<0.05	<0.5	<0.5	<0.05	<0.5	2	0.4	<0.5	2	0.4	2	4	0.9	2
Nigeria	Kaduna	3,723	<0.5	<0.05	<0.5	<0.05	<0.5	<0.5	<0.05	<0.5	9	0.2	<0.5	9	0.2	5	17	0.5	6
Nigeria	Kano	1,329	<0.5	<0.05	<0.5	<0.05	<0.5	<0.5	<0.05	<0.5	4	0.3	<0.5	5	0.3	4	9	0.7	4
Nigeria	Kebbi	279	<0.5	<0.05	<0.5	<0.05	<0.5	<0.5	<0.05	<0.5	1	0.3	<0.5	1	0.3	1	2	0.7	1
Nigeria	Kogi	2,399	<0.5	<0.05	<0.5	<0.05	<0.5	<0.5	<0.05	<0.5	10	0.4	<0.5	<0.5	<0.05	<0.5	11	0.4	<0.5
Nigeria	Kwara	2,027	<0.5	<0.05	<0.5	<0.05	<0.5	<0.5	<0.05	<0.5	9	0.4	<0.5	9	0.5	9	19	0.9	9
Nigeria	Niger	3,564	2	0.1	1	<0.05	<0.5	<0.5	<0.05	<0.5	15	0.4	1	15	0.4	11	30	0.8	12
Nigeria	Ogun	462	<0.5	<0.05	<0.5	<0.05	<0.5	<0.5	<0.05	<0.5	3	0.6	<0.5	<0.5	<0.05	<0.5	3	0.6	<0.5
Nigeria	Ondo	1,837	<0.5	<0.05	<0.5	<0.05	<0.5	<0.5	<0.05	<0.5	8	0.4	<0.5	<0.5	<0.05	<0.5	8	0.4	<0.5
Nigeria	Osun	2,167	2	0.1	1	<0.05	<0.5	<0.5	<0.05	<0.5	9	0.4	<0.5	<0.5	<0.05	<0.5	10	0.5	<0.5
Nigeria	Oyo	1,478	2	0.1	1	<0.05	<0.5	<0.5	<0.05	<0.5	5	0.3	<0.5	6	0.4	5	11	0.8	5
Nigeria	P20Nigeria*	298	115	38.5	38	12.8	<0.5	24	8.1	3	8	2.6	<0.5	<0.5	0.1	<0.5	70	23.6	4
Nigeria	P5Nigeria*#	10,135	1,892	18.7	210	2.1	2	358	3.5	46	109	1.1	4	131	1.3	197	808	8.0	249
Nigeria	Plateau Nassarawa	2,055	<0.5	<0.05	<0.5	<0.05	<0.5	<0.5	<0.05	<0.5	9	0.4	<0.5	9	0.4	7	18	0.9	7
Nigeria	Plateau Nassarawa LF	2,080	<0.5	<0.05	<0.5	<0.05	<0.5	<0.5	<0.05	<0.5	12	0.6	<0.5	12	0.6	12	24	1.2	13
Nigeria	Taraba	2,236	9	0.4	4	0.2	<0.5	1	<0.05	<0.5	15	0.7	1	17	0.8	21	37	1.6	22
Nigeria	Yobe	827	<0.5	<0.05	<0.5	<0.05	<0.5	<0.5	<0.05	<0.5	2	0.3	<0.5	2	0.3	2	5	0.6	2
Nigeria	Zamfara	384	<0.5	<0.05	<0.5	<0.05	<0.5	<0.5	<0.05	<0.5	1	0.4	<0.5	1	0.4	1	3	0.7	1
South Sudan	East Bahr El Ghazal	736	46	6.2	10	1.3	<0.5	4	0.5	1	7	0.9	<0.5	8	1.1	11	29	3.9	12
South Sudan	East Equatoria	1,309	143	10.9	24	1.8	<0.5	15	1.2	2	14	1.1	<0.5	16	1.2	23	69	5.2	26
South Sudan	P20SouthSudan*	37	13	36.5	4	10.6	<0.5	3	8.5	<0.5	1	2.2	<0.5	1	2.9	2	9	24.2	3
South Sudan	P5SouthSudan*#	1,015	183	18.0	20	1.9	<0.5	34	3.4	4	11	1.1	<0.5	12	1.2	19	77	7.6	24
South Sudan	Upper Nile	686	60	8.7	7	1.0	<0.5	6	0.9	1	6	0.8	<0.5	6	0.9	8	25	3.7	9
South Sudan	West Bahr El Ghazal	3,968	509	12.8	141	3.5	1	63	1.6	9	51	1.3	2	63	1.6	133	317	8.0	145

(continued)

Country	Project	Pop. at risk	Mf-positive		Palpable nodules			Reversible skin disease			Irreverisble skin disease			Eye disease (OED)			Total all manifestations		
			N	%	N	%	DALYs	N	%	DALYs	N	%	DALYs	N	%	DALYs	N	%	DALYs
South Sudan	West Equatoria	936	26	2.7	7	0.8	<0.5	2	0.2	<0.5	10	1.0	<0.5	11	1.2	14	30	3.2	15
Sudan	P5Sudan*#	286	61	21.2	8	2.7	<0.5	13	4.4	2	4	1.3	<0.5	5	1.6	8	28	10.0	9
Sudan	Sudan	270	2	0.7	<0.5	0.1	<0.5	<0.5	0.1	<0.5	2	0.6	<0.5	2	0.6	2	4	1.4	2
Sudan	Sudan Abu Hamed	270	2	0.7	<0.5	0.1	<0.5	<0.5	0.1	<0.5	2	0.6	<0.5	2	0.6	2	4	1.4	2
Tanzania	Kilosa	647	1	0.1	<0.5	<0.05	<0.5	<0.5	<0.05	<0.5	5	0.7	<0.5	<0.5	<0.05	<0.5	5	0.8	<0.5
Tanzania	Mahenge	647	8	1.3	3	0.5	<0.5	1	0.1	<0.5	8	1.3	<0.5	<0.5	0.1	1	13	1.9	1
Tanzania	Morogoro	463	2	0.3	1	0.1	<0.5	<0.5	<0.05	<0.5	3	0.7	<0.5	<0.5	<0.05	<0.5	4	0.9	<0.5
Tanzania	P5Tanzania*	1,174	296	25.2	50	4.3	1	66	5.6	8	19	1.7	1	<0.5	<0.05	1	136	11.6	10
Tanzania	Ruvuma	511	28	5.4	12	2.4	<0.5	2	0.3	<0.5	13	2.5	1	1	0.2	3	27	5.3	4
Tanzania	Tanga	401	<0.5	0.1	<0.5	<0.05	<0.5	<0.5	<0.05	<0.5	2	0.6	<0.5	<0.5	<0.05	<0.5	2	0.6	<0.5
Tanzania	Tukuyu	147	<0.5	0.3	<0.5	0.1	<0.5	<0.5	<0.05	<0.5	2	1.1	<0.5	<0.5	<0.05	<0.5	2	1.2	<0.5
Tanzania	Tunduru	159	3	1.9	1	0.9	<0.5	<0.5	0.2	<0.5	3	1.7	<0.5	<0.5	0.1	<0.5	5	2.9	<0.5
Uganda	P5Uganda*	295	<0.5	<0.05	<0.5	<0.05	<0.5	<0.5	<0.05	<0.5	1	0.4	<0.5	<0.5	<0.05	<0.5	1	0.4	<0.5
Uganda	Phase 1	463	<0.5	<0.05	<0.5	<0.05	<0.5	<0.5	<0.05	<0.5	2	0.5	<0.5	<0.5	<0.05	<0.5	2	0.5	<0.5
Uganda	Phase 2	900	<0.5	<0.05	<0.5	<0.05	<0.5	<0.5	<0.05	<0.5	3	0.4	<0.5	<0.5	<0.05	<0.5	3	0.4	<0.5
Uganda	Phase 3	1,660	3	0.2	4	0.2	<0.5	1	0.1	<0.5	25	1.5	1	1	0.1	3	31	1.9	5
Uganda	Phase 4	940	<0.5	<0.05	<0.5	<0.05	<0.5	<0.5	<0.05	<0.5	4	0.5	<0.5	<0.5	<0.05	<0.5	4	0.5	<0.5
Uganda	Phase 5	605	5	0.8	6	1.0	<0.5	2	0.3	<0.5	5	0.9	<0.5	<0.5	<0.05	<0.5	14	2.2	1
**Total**	**Total**	**180,004**	**14,092**	**7.8**	**3,810**	**2.1**	**40**	**2,508**	**1.4**	**341**	**2,328**	**1.3**	**99**	**552**	**0.3**	**916**	**9,198**	**5.1**	**1,397**

* P5 and P20 APOC-projects are a compilation of multiple areas within a country with a prevalence of palpable nodule of $\geq 5\%- < 20\%$ and $\geq 20\%$ respectively.

Onchocerciasis hypoendemic areas with confirmed *L. loa* co-endemicity

Table W: Population at risk, number of cases with infection and clinical manifestations, and DALYs lost due to onchocerciasis by country and APOC-project for 2030. Absolute numbers (N and DALYs) are presented in thousands.

			Mf-positive		Palpable nodules			Reversible skin disease			Irreverisble skin disease			Eye disease (OED)			Total all manifestations		
Country	Project	Pop. at risk	N	%	N	%	DALYs	N	%	DALYs	N	%	DALYs	N	%	DALYs	N	%	DALYs
Angola	Bengo	39	<0.5	<0.05	<0.5	<0.05	<0.5	<0.5	<0.05	<0.5	<0.5	0.5	<0.5	<0.5	<0.05	<0.5	<0.5	0.5	<0.5
Angola	Benguela	75	<0.5	0.3	<0.5	0.1	<0.5	<0.5	<0.05	<0.5	<0.5	0.5	<0.5	<0.5	<0.05	<0.5	<0.5	0.6	<0.5
Angola	Cuanza Norte	38	<0.5	<0.05	<0.5	<0.05	<0.5	<0.5	<0.05	<0.5	<0.5	0.4	<0.5	<0.5	<0.05	<0.5	<0.5	0.4	<0.5
Angola	Huila	352	1	0.3	<0.5	0.1	<0.5	<0.5	<0.05	<0.5	3	0.8	<0.5	<0.5	<0.05	<0.5	3	0.9	<0.5
Angola	Kuando Kubango	607	<0.5	<0.05	<0.5	<0.05	<0.5	<0.5	<0.05	<0.5	2	0.3	<0.5	<0.5	<0.05	<0.5	2	0.3	<0.5
Angola	Lunda Norte	453	<0.5	<0.05	<0.5	<0.05	<0.5	<0.5	<0.05	<0.5	2	0.5	<0.5	<0.5	<0.05	<0.5	2	0.5	<0.5
Angola	Lunda sul	382	<0.5	<0.05	<0.5	<0.05	<0.5	<0.5	<0.05	<0.5	2	0.5	<0.5	<0.5	<0.05	<0.5	2	0.5	<0.5
Angola	Moxico 1	399	<0.5	<0.05	<0.5	<0.05	<0.5	<0.5	<0.05	<0.5	2	0.4	<0.5	<0.5	<0.05	<0.5	2	0.4	<0.5
Angola	Namibe	53	3	5.3	<0.5	0.9	<0.5	<0.5	0.4	<0.5	1	1.0	<0.5	<0.5	<0.05	<0.5	1	2.3	<0.5
Angola	NY Benguela	168	9	5.6	2	1.1	<0.5	1	0.5	<0.5	2	1.0	<0.5	<0.5	<0.05	<0.5	4	2.6	<0.5
Angola	NY Cuanza Norte	27	1	4.3	<0.5	0.5	<0.5	<0.5	0.4	<0.5	<0.5	0.9	<0.5	<0.5	<0.05	<0.5	<0.5	1.7	<0.5
Angola	NY Huila	34	2	5.6	<0.5	1.0	<0.5	<0.5	0.5	<0.5	<0.5	1.0	<0.5	<0.5	<0.05	<0.5	1	2.5	<0.5
Angola	NY Lunda Norte	99	5	5.4	1	0.9	<0.5	<0.5	0.4	<0.5	1	1.0	<0.5	<0.5	<0.05	<0.5	2	2.3	<0.5
Angola	NY Moxico 1	510	14	2.8	2	0.3	<0.5	1	0.2	<0.5	3	0.6	<0.5	<0.5	<0.05	<0.5	6	1.2	<0.5
Angola	P5Angola[*#]	388	50	12.8	6	1.6	<0.5	5	1.4	1	4	1.0	<0.5	<0.5	<0.05	<0.5	16	4.0	1
Angola	Uige	280	15	5.2	3	1.0	<0.5	1	0.4	<0.5	3	0.9	<0.5	<0.5	<0.05	<0.5	7	2.4	<0.5
Angola	Zaire	21	1	2.9	<0.5	0.3	<0.5	<0.5	0.2	<0.5	<0.5	0.6	<0.5	<0.5	<0.05	<0.5	<0.5	1.2	<0.5
Burundi	Bururi	652	<0.5	<0.05	<0.5	<0.05	<0.5	<0.5	<0.05	<0.5	2	0.3	<0.5	<0.5	<0.05	<0.5	2	0.4	<0.5
Burundi	Cibitoke-Bubanza	1,557	<0.5	<0.05	<0.5	<0.05	<0.5	<0.5	<0.05	<0.5	8	0.5	<0.5	<0.5	<0.05	<0.5	8	0.5	<0.5
Burundi	P5Burundi[*#]	1,228	163	13.3	25	2.0	<0.5	17	1.4	3	13	1.0	<0.5	<0.5	<0.05	<0.5	55	4.5	4
Burundi	Rutana	508	<0.5	<0.05	<0.5	<0.05	<0.5	<0.5	<0.05	<0.5	1	0.3	<0.5	<0.5	<0.05	<0.5	1	0.3	<0.5
Cameroon	Adamaoua 1	850	<0.5	<0.05	<0.5	<0.05	<0.5	<0.5	<0.05	<0.5	4	0.5	<0.5	4	0.5	4	8	1.0	4
Cameroon	Adamaoua 2	772	<0.5	<0.05	<0.5	<0.05	<0.5	<0.5	<0.05	<0.5	3	0.4	<0.5	3	0.4	2	6	0.8	3
Cameroon	Centre 1	785	1	0.1	<0.5	<0.05	<0.5	<0.5	<0.05	<0.5	8	1.0	<0.5	<0.5	<0.05	<0.5	8	1.1	1
Cameroon	Centre 2	185	1	0.3	<0.5	0.1	<0.5	<0.5	<0.05	<0.5	2	0.9	<0.5	<0.5	<0.05	<0.5	2	1.0	<0.5
Cameroon	Centre 3	596	<0.5	<0.05	<0.5	<0.05	<0.5	<0.5	<0.05	<0.5	3	0.6	<0.5	<0.5	<0.05	<0.5	3	0.6	<0.5
Cameroon	East	218	<0.5	0.2	<0.5	0.1	<0.5	<0.5	<0.05	<0.5	2	0.9	<0.5	<0.5	<0.05	<0.5	2	1.1	<0.5
Cameroon	Far North	515	<0.5	<0.05	<0.5	<0.05	<0.5	<0.5	<0.05	<0.5	1	0.2	<0.5	1	0.2	1	2	0.4	1

(continued)

Country	Project	Pop. at risk	Mf-positive		Palpable nodules			Reversible skin disease			Irreverisble skin disease			Eye disease (OED)			Total all manifestations		
			N	%	N	%	DALYs	N	%	DALYs	N	%	DALYs	N	%	DALYs	N	%	DALYs
Cameroon	Littoral 1	515	8	1.5	2	0.4	<0.5	<0.5	0.1	<0.5	8	1.5	<0.5	<0.5	0.1	1	11	2.1	1
Cameroon	Littoral 2	275	3	1.0	1	0.3	<0.5	<0.5	<0.05	<0.5	4	1.6	<0.5	<0.5	0.1	1	6	2.0	1
Cameroon	Northern	1,155	<0.5	<0.05	<0.5	<0.05	<0.5	<0.5	<0.05	<0.5	7	0.6	<0.5	8	0.7	10	15	1.3	10
Cameroon	Northwest	1,501	1	0.1	<0.5	<0.05	<0.5	<0.5	<0.05	<0.5	11	0.7	<0.5	<0.5	<0.05	<0.5	12	0.8	1
Cameroon	P20Cameroon*	276	8	2.9	3	1.1	<0.5	1	0.3	<0.5	4	1.5	<0.5	<0.5	<0.05	<0.5	8	2.9	1
Cameroon	P5Cameroon*#	2,751	395	14.4	64	2.3	1	45	1.6	7	31	1.1	1	1	<0.05	1	141	5.1	10
Cameroon	South	548	<0.5	<0.05	<0.5	<0.05	<0.5	<0.5	<0.05	<0.5	2	0.5	<0.5	<0.5	<0.05	<0.5	3	0.5	<0.5
Cameroon	South West 1	719	<0.5	<0.05	<0.5	<0.05	<0.5	<0.5	<0.05	<0.5	9	1.2	<0.5	<0.5	<0.05	<0.5	9	1.3	1
Cameroon	South West 2	489	<0.5	<0.05	<0.5	<0.05	<0.5	<0.5	<0.05	<0.5	5	1.0	<0.5	<0.5	<0.05	<0.5	5	1.0	<0.5
Cameroon	Western	3,007	9	0.3	3	0.1	<0.5	<0.5	<0.05	<0.5	57	1.9	3	3	0.1	7	63	2.1	9
CAR	CAR region 3	784	3	0.4	1	0.1	<0.5	<0.5	<0.05	<0.5	6	0.8	<0.5	7	0.9	8	14	1.8	9
CAR	CAR region 4	817	1	0.1	<0.5	<0.05	<0.5	<0.5	<0.05	<0.5	3	0.4	<0.5	3	0.4	3	7	0.8	3
CAR	CAR region 5	756	<0.5	<0.05	<0.5	<0.05	<0.5	<0.5	<0.05	<0.5	2	0.3	<0.5	2	0.3	1	4	0.6	1
CAR	CAR region 6	1,163	3	0.2	1	0.1	<0.5	<0.5	<0.05	<0.5	7	0.6	<0.5	7	0.6	7	15	1.2	7
CAR	P20CAR*	115	14	11.9	4	3.8	<0.5	1	1.1	<0.5	2	1.8	<0.5	<0.5	<0.05	<0.5	8	6.8	<0.5
CAR	P5CAR*#	260	42	16.1	8	3.2	<0.5	5	1.9	1	3	1.3	<0.5	<0.5	<0.05	<0.5	17	6.5	1
Chad	Chad	3,160	<0.5	<0.05	<0.5	<0.05	<0.5	<0.5	<0.05	<0.5	8	0.3	<0.5	8	0.3	6	16	0.5	6
Chad	P5Chad*#	393	59	15.0	14	3.6	<0.5	7	1.7	1	4	1.1	<0.5	5	1.3	8	30	7.7	10
Chad	P5Chad propext*#	226	27	11.7	4	1.8	<0.5	3	1.2	<0.5	2	0.9	<0.5	2	1.0	3	11	4.9	4
Congo	Congo 1	1,441	<0.5	<0.05	<0.5	<0.05	<0.5	<0.5	<0.05	<0.5	5	0.3	<0.5	<0.5	<0.05	<0.5	5	0.3	<0.5
Congo	P20Congo*	63	2	2.7	1	1.1	<0.5	<0.5	0.2	<0.5	1	2.3	<0.5	<0.5	0.1	<0.5	2	3.6	<0.5
Congo	P5Congo*#	846	142	16.8	29	3.4	<0.5	18	2.1	3	12	1.5	<0.5	<0.5	<0.05	1	59	7.0	4
DRC	Bandundu	10	<0.5	<0.05	<0.5	<0.05	<0.5	<0.5	<0.05	<0.5	<0.5	0.4	<0.5	<0.5	<0.05	<0.5	<0.5	0.4	<0.5
DRC	Bas-Congo Kinshasa	2,378	1	0.1	1	<0.05	<0.5	<0.5	<0.05	<0.5	15	0.6	1	<0.5	<0.05	<0.5	16	0.7	1
DRC	Butembo-Beni	1,478	11	0.7	3	0.2	<0.5	1	<0.05	<0.5	15	1.0	1	<0.5	<0.05	1	20	1.4	1
DRC	Equateur-Kiri	1,964	2	0.1	1	<0.05	<0.5	<0.5	<0.05	<0.5	16	0.8	1	<0.5	<0.05	<0.5	17	0.9	1
DRC	Ituri-Nord	1,987	57	2.9	20	1.0	<0.5	2	0.1	<0.5	57	2.9	3	4	0.2	11	83	4.2	15
DRC	Ituri-Sud	1,816	177	9.7	66	3.6	1	11	0.6	2	55	3.0	3	4	0.2	12	136	7.5	17
DRC	Kasai	17,038	138	0.8	46	0.3	<0.5	4	<0.05	1	146	0.9	7	6	<0.05	16	202	1.2	23
DRC	Kasongo	2,134	8	0.4	3	0.1	<0.5	<0.5	<0.05	<0.5	15	0.7	1	<0.5	<0.05	1	18	0.9	1

(continued)

Country	Project	Pop. at risk	Mf-positive		Palpable nodules			Reversible skin disease			Irreverisble skin disease			Eye disease (OED)			Total all manifestations		
			N	%	N	%	DALYs	N	%	DALYs	N	%	DALYs	N	%	DALYs	N	%	DALYs
DRC	Katanga-Nord	992	3	0.3	1	0.1	<0.5	<0.5	<0.05	<0.5	9	0.9	<0.5	<0.5	<0.05	1	11	1.1	1
DRC	Katanga-Sud	1,098	9	0.8	3	0.3	<0.5	<0.5	<0.05	<0.5	11	1.0	<0.5	<0.5	<0.05	1	15	1.3	2
DRC	Lualaba	356	<0.5	<0.05	<0.5	<0.05	<0.5	<0.5	<0.05	<0.5	3	0.8	<0.5	<0.5	<0.05	<0.5	3	0.8	<0.5
DRC	Lubutu	529	5	0.9	2	0.3	<0.5	<0.5	0.1	<0.5	9	1.7	<0.5	<0.5	<0.05	<0.5	11	2.1	1
DRC	Masisi-Walikale	1,659	40	2.4	14	0.9	<0.5	2	0.1	<0.5	28	1.7	1	1	0.1	3	46	2.8	5
DRC	Mongala	2,306	2	0.1	1	<0.05	<0.5	<0.5	<0.05	<0.5	16	0.7	1	<0.5	<0.05	1	18	0.8	1
DRC	NY Katanga-Nord	720	10	1.4	2	0.3	<0.5	1	0.1	<0.5	5	0.7	<0.5	<0.5	<0.05	<0.5	8	1.2	<0.5
DRC	NY Lualaba	1,557	27	1.7	5	0.3	<0.5	2	0.1	<0.5	13	0.9	<0.5	<0.5	<0.05	<0.5	21	1.3	1
DRC	NY Masisi-Walikale	86	2	2.5	1	0.7	<0.5	<0.5	0.2	<0.5	1	1.2	<0.5	<0.5	<0.05	<0.5	2	2.1	<0.5
DRC	NY Rutshuru-Ngoma	13	<0.5	1.3	<0.5	0.4	<0.5	<0.5	0.1	<0.5	<0.5	1.5	<0.5	<0.5	<0.05	<0.5	<0.5	2.1	<0.5
DRC	NY Sankuru	743	10	1.3	3	0.4	<0.5	1	0.1	<0.5	6	0.9	<0.5	<0.5	<0.05	<0.5	10	1.4	1
DRC	NY Ueles	253	36	14.4	15	5.9	<0.5	3	1.1	<0.5	9	3.6	<0.5	1	0.3	2	27	10.9	3
DRC	P20DRC*	3,726	518	13.9	219	5.9	2	57	1.5	9	94	2.5	4	5	0.1	15	375	10.1	31
DRC	P5DRC*#	11,848	1,732	14.6	287	2.4	3	196	1.7	30	143	1.2	5	2	<0.05	2	629	5.3	40
DRC	Rutshuru-Ngoma	1,045	<0.5	<0.05	<0.5	<0.05	<0.5	<0.5	<0.05	<0.5	7	0.6	<0.5	<0.5	<0.05	<0.5	7	0.7	<0.5
DRC	Sankuru	1,690	13	0.8	5	0.3	<0.5	<0.5	<0.05	<0.5	25	1.5	1	2	0.1	4	32	1.9	6
DRC	Tshopo	2,525	92	3.6	31	1.2	<0.5	5	0.2	1	40	1.6	2	2	0.1	5	78	3.1	8
DRC	Tshuapa	2,249	67	3.0	22	1.0	<0.5	4	0.2	1	35	1.6	2	1	0.1	3	62	2.8	6
DRC	Ubangi-Nord	1,267	56	4.4	19	1.5	<0.5	3	0.2	<0.5	21	1.6	1	1	0.1	3	44	3.5	5
DRC	Ubangi-Sud	2,136	2	0.1	1	<0.05	<0.5	<0.5	<0.05	<0.5	13	0.6	<0.5	<0.5	<0.05	<0.5	14	0.7	1
DRC	Ueles	2,492	76	3.0	24	1.0	<0.5	2	0.1	<0.5	51	2.1	3	3	0.1	9	81	3.2	12
Eq. Guinea	Bioko	176	<0.5	<0.05	<0.5	<0.05	<0.5	<0.5	<0.05	<0.5	2	1.4	<0.5	<0.5	0.1	<0.5	3	1.4	<0.5
Eq. Guinea	P5EqGuinea*#	412	<0.5	<0.05	<0.5	<0.05	<0.5	<0.5	<0.05	<0.5	1	0.2	<0.5	<0.5	<0.05	<0.5	1	0.2	<0.5
Ethiopia	Assosa	939	1	0.1	3	0.3	<0.5	1	0.1	<0.5	8	0.9	<0.5	<0.5	<0.05	<0.5	13	1.3	1
Ethiopia	Bench-Maji	1,242	<0.5	<0.05	<0.5	<0.05	<0.5	<0.5	<0.05	<0.5	7	0.5	<0.5	<0.5	<0.05	<0.5	7	0.5	<0.5
Ethiopia	East Wellega	1,521	<0.5	<0.05	<0.5	<0.05	<0.5	<0.5	<0.05	<0.5	8	0.5	<0.5	<0.5	<0.05	<0.5	8	0.5	<0.5
Ethiopia	Gambella	182	<0.5	<0.05	<0.5	<0.05	<0.5	<0.5	<0.05	<0.5	1	0.5	<0.5	<0.5	<0.05	<0.5	1	0.5	<0.5

(continued)

Country	Project	Pop. at risk	Mf-positive		Palpable nodules			Reversible skin disease			Irreverisble skin disease			Eye disease (OED)			Total all manifestations		
			N	%	N	%	DALYs	N	%	DALYs	N	%	DALYs	N	%	DALYs	N	%	DALYs
Ethiopia	Horo Guduru	89	4	4.4	1	1.4	<0.5	<0.5	0.3	<0.5	1	1.2	<0.5	<0.5	<0.05	<0.5	3	3.0	<0.5
Ethiopia	Illubabor	1,287	<0.5	<0.05	<0.5	<0.05	<0.5	<0.5	<0.05	<0.5	7	0.6	<0.5	<0.5	<0.05	<0.5	7	0.6	<0.5
Ethiopia	Jimma	1,519	<0.5	<0.05	<0.5	<0.05	<0.5	<0.5	<0.05	<0.5	5	0.3	<0.5	<0.5	<0.05	<0.5	5	0.3	<0.5
Ethiopia	Kaffa-Sheka	2,157	<0.5	<0.05	<0.5	<0.05	<0.5	<0.5	<0.05	<0.5	14	0.6	<0.5	<0.5	<0.05	<0.5	14	0.6	1
Ethiopia	Kamashi	802	<0.5	<0.05	1	0.1	<0.5	1	0.1	<0.5	10	1.3	<0.5	<0.5	<0.05	1	12	1.5	1
Ethiopia	Metekel	275	<0.5	<0.05	<0.5	<0.05	<0.5	<0.5	<0.05	<0.5	1	0.4	<0.5	<0.5	<0.05	<0.5	1	0.4	<0.5
Ethiopia	North Gondar	533	<0.5	<0.05	<0.5	<0.05	<0.5	<0.5	<0.05	<0.5	2	0.4	<0.5	<0.5	<0.05	<0.5	2	0.4	<0.5
Ethiopia	NY East Wellega	487	8	1.7	3	0.7	<0.5	1	0.2	<0.5	6	1.2	<0.5	<0.5	<0.05	<0.5	10	2.1	1
Ethiopia	NY West Wellega	495	<0.5	<0.05	<0.5	<0.05	<0.5	<0.5	<0.05	<0.5	3	0.6	<0.5	<0.5	<0.05	<0.5	3	0.7	<0.5
Ethiopia	P20Ethiopia*	566	39	6.9	16	2.9	<0.5	4	0.7	1	10	1.7	<0.5	<0.5	<0.05	1	30	5.3	2
Ethiopia	P5Ethiopia*	5,972	619	10.4	100	1.7	1	56	0.9	9	64	1.1	2	1	<0.05	1	221	3.7	14
Ethiopia	West Shewa	98	9	9.0	1	1.2	<0.5	1	0.8	<0.5	1	0.9	<0.5	<0.5	<0.05	<0.5	3	2.9	<0.5
Ethiopia	West Wellega	1,747	<0.5	<0.05	<0.5	<0.05	<0.5	<0.5	<0.05	<0.5	14	0.8	1	<0.5	<0.05	<0.5	15	0.9	1
Gabon	P5Gabon*#	146	17	11.9	2	1.5	<0.5	2	1.2	<0.5	1	0.9	<0.5	<0.5	<0.05	<0.5	5	3.6	<0.5
Liberia	All_Liberia	2,606	<0.5	<0.05	<0.5	<0.05	<0.5	<0.5	<0.05	<0.5	8	0.3	<0.5	<0.5	<0.05	<0.5	8	0.3	<0.5
Malawi	Malawi Extension	1,968	<0.5	<0.05	<0.5	<0.05	<0.5	<0.5	<0.05	<0.5	4	0.2	<0.5	<0.5	<0.05	<0.5	4	0.2	<0.5
Malawi	Thyolo Mwanza	1,501	<0.5	<0.05	<0.5	<0.05	<0.5	<0.5	<0.05	<0.5	3	0.2	<0.5	<0.5	<0.05	<0.5	3	0.2	<0.5
Mozamb.	P20Mozambique*	27	1	2.7	<0.5	0.3	<0.5	<0.5	0.2	<0.5	<0.5	0.6	<0.5	<0.5	<0.05	<0.5	<0.5	1.2	<0.5
Mozamb.	P5Mozambique*	80	5	6.8	1	0.8	<0.5	<0.5	0.6	<0.5	1	0.7	<0.5	<0.5	<0.05	<0.5	2	2.1	<0.5
Nigeria	Adamawa	3,015	<0.5	<0.05	<0.5	<0.05	<0.5	<0.5	<0.05	<0.5	6	0.2	<0.5	5	0.2	4	12	0.4	4
Nigeria	Akwa Ibom	52	<0.5	<0.05	<0.5	<0.05	<0.5	<0.5	<0.05	<0.5	<0.5	0.3	<0.5	<0.5	<0.05	<0.5	<0.5	0.3	<0.5
Nigeria	Bauchi	3,089	<0.5	<0.05	<0.5	<0.05	<0.5	<0.5	<0.05	<0.5	7	0.2	<0.5	7	0.2	5	14	0.5	5
Nigeria	Benue	6,092	1	<0.05	<0.5	<0.05	<0.5	<0.5	<0.05	<0.5	34	0.6	1	<0.5	<0.05	1	34	0.6	2
Nigeria	Borno	2,428	<0.5	<0.05	<0.5	<0.05	<0.5	<0.5	<0.05	<0.5	3	0.1	<0.5	3	0.1	1	6	0.3	1
Nigeria	Cross River	2,202	<0.5	<0.05	<0.5	<0.05	<0.5	<0.5	<0.05	<0.5	6	0.3	<0.5	<0.5	<0.05	<0.5	7	0.3	<0.5
Nigeria	Edo Delta	2,831	<0.5	<0.05	<0.5	<0.05	<0.5	<0.5	<0.05	<0.5	6	0.2	<0.5	<0.5	<0.05	<0.5	6	0.2	<0.5
Nigeria	Ekiti	3,821	<0.5	<0.05	<0.5	<0.05	<0.5	<0.5	<0.05	<0.5	7	0.2	<0.5	<0.5	<0.05	<0.5	7	0.2	<0.5
Nigeria	Enugu Anambra Ebony	4,206	<0.5	<0.05	<0.5	<0.05	<0.5	<0.5	<0.05	<0.5	25	0.6	1	<0.5	<0.05	1	26	0.6	2

(continued)

			Mf-positive		Palpable nodules			Reversible skin disease			Irreverisble skin disease			Eye disease (OED)			Total all manifestations		
Country	Project	Pop. at risk	N	%	N	%	DALYs	N	%	DALYs	N	%	DALYs	N	%	DALYs	N	%	DALYs
Nigeria	FCT	893	<0.5	<0.05	<0.5	<0.05	<0.5	<0.5	<0.05	<0.5	2	0.2	<0.5	<0.5	<0.05	<0.5	2	0.2	<0.5
Nigeria	Gombe	3,356	<0.5	<0.05	<0.5	<0.05	<0.5	<0.5	<0.05	<0.5	8	0.2	<0.5	7	0.2	3	15	0.4	4
Nigeria	Imo Abia	2,319	<0.5	<0.05	<0.5	<0.05	<0.5	<0.5	<0.05	<0.5	6	0.3	<0.5	<0.5	<0.05	<0.5	6	0.3	<0.5
Nigeria	Jigawa	629	<0.5	<0.05	<0.5	<0.05	<0.5	<0.5	<0.05	<0.5	1	0.2	<0.5	1	0.2	1	2	0.4	1
Nigeria	Kaduna	4,884	<0.5	<0.05	<0.5	<0.05	<0.5	<0.5	<0.05	<0.5	4	0.1	<0.5	4	0.1	1	8	0.2	1
Nigeria	Kano	1,743	<0.5	<0.05	<0.5	<0.05	<0.5	<0.5	<0.05	<0.5	3	0.2	<0.5	2	0.1	1	5	0.3	1
Nigeria	Kebbi	366	<0.5	<0.05	<0.5	<0.05	<0.5	<0.5	<0.05	<0.5	1	0.2	<0.5	1	0.1	<0.5	1	0.3	<0.5
Nigeria	Kogi	3,146	<0.5	<0.05	<0.5	<0.05	<0.5	<0.5	<0.05	<0.5	6	0.2	<0.5	<0.5	<0.05	<0.5	6	0.2	<0.5
Nigeria	Kwara	2,659	<0.5	<0.05	<0.5	<0.05	<0.5	<0.5	<0.05	<0.5	5	0.2	<0.5	5	0.2	2	10	0.4	3
Nigeria	Niger	4,675	<0.5	<0.05	<0.5	<0.05	<0.5	<0.5	<0.05	<0.5	9	0.2	<0.5	8	0.2	4	17	0.4	5
Nigeria	Ogun	606	<0.5	<0.05	<0.5	<0.05	<0.5	<0.5	<0.05	<0.5	2	0.3	<0.5	<0.5	<0.05	<0.5	2	0.3	<0.5
Nigeria	Ondo	2,409	<0.5	<0.05	<0.5	<0.05	<0.5	<0.5	<0.05	<0.5	5	0.2	<0.5	<0.5	<0.05	<0.5	5	0.2	<0.5
Nigeria	Osun	2,842	<0.5	<0.05	<0.5	<0.05	<0.5	<0.5	<0.05	<0.5	5	0.2	<0.5	<0.5	<0.05	<0.5	5	0.2	<0.5
Nigeria	Oyo	1,938	<0.5	<0.05	<0.5	<0.05	<0.5	<0.5	<0.05	<0.5	3	0.2	<0.5	3	0.2	2	6	0.3	2
Nigeria	P20Nigeria*	391	23	6.0	9	2.2	<0.5	2	0.5	<0.5	6	1.6	<0.5	<0.5	<0.05	<0.5	17	4.3	1
Nigeria	P5Nigeria*#	13,294	1,450	10.9	178	1.3	2	147	1.1	23	112	0.8	4	126	1.0	164	563	4.2	192
Nigeria	Plateau Nassarawa	2,695	<0.5	<0.05	<0.5	<0.05	<0.5	<0.5	<0.05	<0.5	6	0.2	<0.5	5	0.2	3	11	0.4	3
Nigeria	Plateau Nassarawa LF	2,729	<0.5	<0.05	<0.5	<0.05	<0.5	<0.5	<0.05	<0.5	7	0.2	<0.5	7	0.2	4	13	0.5	4
Nigeria	Taraba	2,932	1	<0.05	<0.5	<0.05	<0.5	<0.5	<0.05	<0.5	9	0.3	<0.5	10	0.3	9	20	0.7	9
Nigeria	Yobe	1,085	<0.5	<0.05	<0.5	<0.05	<0.5	<0.5	<0.05	<0.5	1	0.1	<0.5	1	0.1	1	3	0.2	1
Nigeria	Zamfara	503	<0.5	<0.05	<0.5	<0.05	<0.5	<0.5	<0.05	<0.5	1	0.2	<0.5	1	0.1	<0.5	2	0.3	<0.5
South Sudan	East Bahr El Ghazal	966	4	0.4	1	0.1	<0.5	<0.5	<0.05	<0.5	5	0.5	<0.5	5	0.5	5	11	1.2	5
South Sudan	East Equatoria	1,716	26	1.5	7	0.4	<0.5	1	0.1	<0.5	10	0.6	<0.5	11	0.6	11	29	1.7	11
South Sudan	P20SouthSudan*	48	7	13.6	2	3.9	<0.5	1	1.3	<0.5	1	1.5	<0.5	1	1.8	1	4	8.5	2
South Sudan	P5SouthSudan*#	1,331	141	10.6	17	1.3	<0.5	14	1.1	2	11	0.8	<0.5	12	0.9	16	54	4.0	19
South Sudan	Upper Nile	899	9	1.0	2	0.2	<0.5	1	0.1	<0.5	4	0.4	<0.5	4	0.5	4	10	1.2	4
South Sudan	West Bahr El Ghazal	5,205	137	2.6	53	1.0	1	11	0.2	2	38	0.7	1	44	0.8	75	145	2.8	79

(continued)

			Mf-positive		Palpable nodules			Reversible skin disease			Irreverisble skin disease			Eye disease (OED)			Total all manifestations		
Country	Project	Pop. at risk	N	%	N	%	DALYs	N	%	DALYs	N	%	DALYs	N	%	DALYs	N	%	DALYs
South Sudan	West Equatoria	1,228	1	<0.05	<0.5	<0.05	<0.5	<0.5	<0.05	<0.5	6	0.5	<0.5	7	0.6	7	14	1.1	7
Sudan	P5Sudan*#	375	48	12.9	7	1.8	<0.5	5	1.4	1	4	1.0	<0.5	4	1.2	6	20	5.3	7
Sudan	Sudan	354	<0.5	<0.05	<0.5	<0.05	<0.5	<0.5	<0.05	<0.5	1	0.3	<0.5	1	0.3	1	2	0.6	1
Sudan	Sudan Abu Hamed	354	<0.5	<0.05	<0.5	<0.05	<0.5	<0.5	<0.05	<0.5	1	0.3	<0.5	1	0.3	1	2	0.6	1
Tanzania	Kilosa	849	<0.5	<0.05	<0.5	<0.05	<0.5	<0.5	<0.05	<0.5	3	0.3	<0.5	<0.5	<0.05	<0.5	3	0.3	<0.5
Tanzania	Mahenge	849	1	0.1	<0.5	<0.05	<0.5	<0.5	<0.05	<0.5	5	0.6	<0.5	<0.5	<0.05	<0.5	6	0.7	1
Tanzania	Morogoro	608	<0.5	<0.05	<0.5	<0.05	<0.5	<0.5	<0.05	<0.5	2	0.4	<0.5	<0.5	<0.05	<0.5	2	0.4	<0.5
Tanzania	P5Tanzania*	1,540	182	11.8	33	2.2	<0.5	19	1.2	3	19	1.2	1	<0.5	<0.05	<0.5	71	4.6	4
Tanzania	Ruvuma	671	3	0.5	1	0.2	<0.5	<0.5	<0.05	<0.5	9	1.3	<0.5	<0.5	0.1	1	10	1.5	1
Tanzania	Tanga	526	<0.5	<0.05	<0.5	<0.05	<0.5	<0.5	<0.05	<0.5	1	0.3	<0.5	<0.5	<0.05	<0.5	1	0.3	<0.5
Tanzania	Tukuyu	193	<0.5	<0.05	<0.5	<0.05	<0.5	<0.5	<0.05	<0.5	1	0.5	<0.5	<0.5	<0.05	<0.5	1	0.5	<0.5
Tanzania	Tunduru	209	<0.5	0.1	<0.5	<0.05	<0.5	<0.5	<0.05	<0.5	2	0.9	<0.5	<0.5	<0.05	<0.5	2	1.0	<0.5
Uganda	P5Uganda*	387	<0.5	<0.05	<0.5	<0.05	<0.5	<0.5	<0.05	<0.5	1	0.2	<0.5	<0.5	<0.05	<0.5	1	0.2	<0.5
Uganda	Phase 1	607	<0.5	<0.05	<0.5	<0.05	<0.5	<0.5	<0.05	<0.5	1	0.2	<0.5	<0.5	<0.05	<0.5	1	0.2	<0.5
Uganda	Phase 2	1,181	<0.5	<0.05	<0.5	<0.05	<0.5	<0.5	<0.05	<0.5	2	0.2	<0.5	<0.5	<0.05	<0.5	2	0.2	<0.5
Uganda	Phase 3	2,178	<0.5	<0.05	<0.5	<0.05	<0.5	<0.5	<0.05	<0.5	16	0.7	1	1	<0.05	1	17	0.8	2
Uganda	Phase 4	1,233	<0.5	<0.05	<0.5	<0.05	<0.5	<0.5	<0.05	<0.5	3	0.2	<0.5	<0.5	<0.05	<0.5	3	0.2	<0.5
Uganda	Phase 5	794	<0.5	<0.05	<0.5	<0.05	<0.5	<0.5	<0.05	<0.5	4	0.5	<0.5	<0.5	<0.05	<0.5	4	0.5	<0.5
**Total**	**Total**	**236,102**	**6,813**	**2.9**	**1,412**	**0.6**	**15**	**670**	**0.3**	**104**	**1,756**	**0.7**	**71**	**384**	**0.2**	**501**	**4,223**	**1.8**	**691**

* P5 and P20 APOC-projects are a compilation of multiple areas within a country with a prevalence of palpable nodule of $\geq 5\% - < 20\%$ and $\geq 20\%$ respectively.

Onchocerciasis hypoendemic areas with confirmed *L. loa* co-endemicity
