# Supplementary material for: The burden of skin disease and eye disease due to onchocerciasis in countries formerly under the African Programme for Onchocerciasis Control mandate for 1990, 2020, and 2030
Source: PLoS Negl Trop Dis. 2021 Jul 26;15(7):e0009604. doi: 10.1371/journal.pntd.0009604 (PMC8312930; doi:10.1371/journal.pntd.0009604)
Supplement: S4 Text — (PDF) [file pntd.0009604.s004.pdf]

The burden of skin disease and eye disease due to onchocerciasis in Africa for 1990, 2020, and 2030

Text S4:
Detailed country-level results

Natalie V.S. Vinkeles Melchers[1], Wilma A. Stolk[1], Welmoed van Loon[1,2], Belén Pedrique[3], Roel Bakker[1], Michele E. Murdoch[4], Sake J. de Vlas[1], Luc E. Coffeng[1]

1. Department of Public Health, Erasmus MC, University Medical Center Rotterdam, Rotterdam, The Netherlands.
2. Institute of Tropical Medicine and International Health, Charité - Universitätsmedizin Berlin, corporate member of Freie Universität Berlin, Humboldt-Universität zu Berlin, and Berlin Institute of Health, Berlin, Germany.
3. Drugs for Neglected Diseases initiative (DND*i*), Geneva, Switzerland
4. Department of Dermatology, Watford General Hospital, Watford, Hertfordshire, UK

* Correspondence to:

Luc E. Coffeng, Department of Public Health, Erasmus MC, University Medical Center Rotterdam, P.O. box 2040, 3000 CA Rotterdam, The Netherlands; l.coffeng@erasmusmc.nl

Alternative:
Natalie VS Vinkeles Melchers, Department of Public Health, Erasmus MC, University Medical Center Rotterdam, P.O. box 2040, 3000 CA Rotterdam, The Netherlands; n.vinkelesmelchers@erasmusmc.nl, Natalie.melchers@gmail.com; +31 614217276

**Country:**	**Angola**
Projects:	Bengo, Benguela, Cuanza Norte, Huila, Kuando Kubango, Lunda Norte, Lunda sul, Moxico 1, Namibe, NY Benguela, NY Cuanza Norte, NY Huila, NY Lunda Norte, NY Moxico 1, P5Angola, Uige, Zaire
Bioclime:	Forest

	1990			2020			2030		
	N	%	DALYs	N	%	DALYs	N	%	DALYs
Total populaton at risk	1,326	100	_	2,992	100	_	3,925	100	_
**Infection:**									
Mf-positive	333	25.1	_	309	10.3	_	101	2.6	_
Mf-positive (age 5+)	332	25.1	_	309	10.3	_	101	2.6	_
Worm infected	442	33.3	_	589	19.7	_	285	7.3	_
**Clinical manifestations:**									
Palpable nodules	55	4.2	1	50	1.7	1	15	0.4	<0.5
Reversible skin disease:									
Severe itch	43	3.2	8	34	1.1	6	8	0.2	1
Reactive skin disease	29	2.2	1	18	0.6	1	2	<0.05	<0.5
**Reversible skin disease**	**72**	**5.4**	**9**	**51**	**1.7**	**7**	**10**	**0.2**	**2**
Irreversible skin disease:									
Mild depigmentation	11	0.8	<0.5	17	0.6	<0.5	14	0.4	<0.5
Severe depigmentation	10	0.7	1	14	0.5	1	11	0.3	1
Atrophy	<0.5	<0.05	<0.5	<0.5	<0.05	<0.5	<0.5	<0.05	<0.5
Hanging groin	<0.5	<0.05	<0.5	<0.5	<0.05	<0.5	<0.5	<0.05	<0.5
**Irreversible skin disease**	**21**	**1.6**	**1**	**32**	**1.1**	**1**	**25**	**0.6**	**1**
**Total skin disease (OSD)**	**148**	**11.2**	**10**	**133**	**4.5**	**9**	**49**	**1.3**	**3**
Eye disease (OED):									
Visual impairment	<0.5	<0.05	<0.5	<0.5	<0.05	<0.5	<0.5	<0.05	<0.5
Blindness	<0.5	<0.05	1	<0.5	<0.05	1	<0.5	<0.05	<0.5
**Eye disease (OED)**	**<0.5**	**<0.05**	**1**	**<0.5**	**<0.05**	**1**	**<0.5**	**<0.05**	**<0.5**
**Total all manifestations**	**148**	**11.2**	**11**	**134**	**4.5**	**9**	**50**	**1.3**	**3**

Note 1: Population at risk, number of cases (N) and DALYs are presented in thousands.

Note 2: Total skin disease is defined as the sum of the number of cases with palpable nodules, reversible and irreversible skin disease.

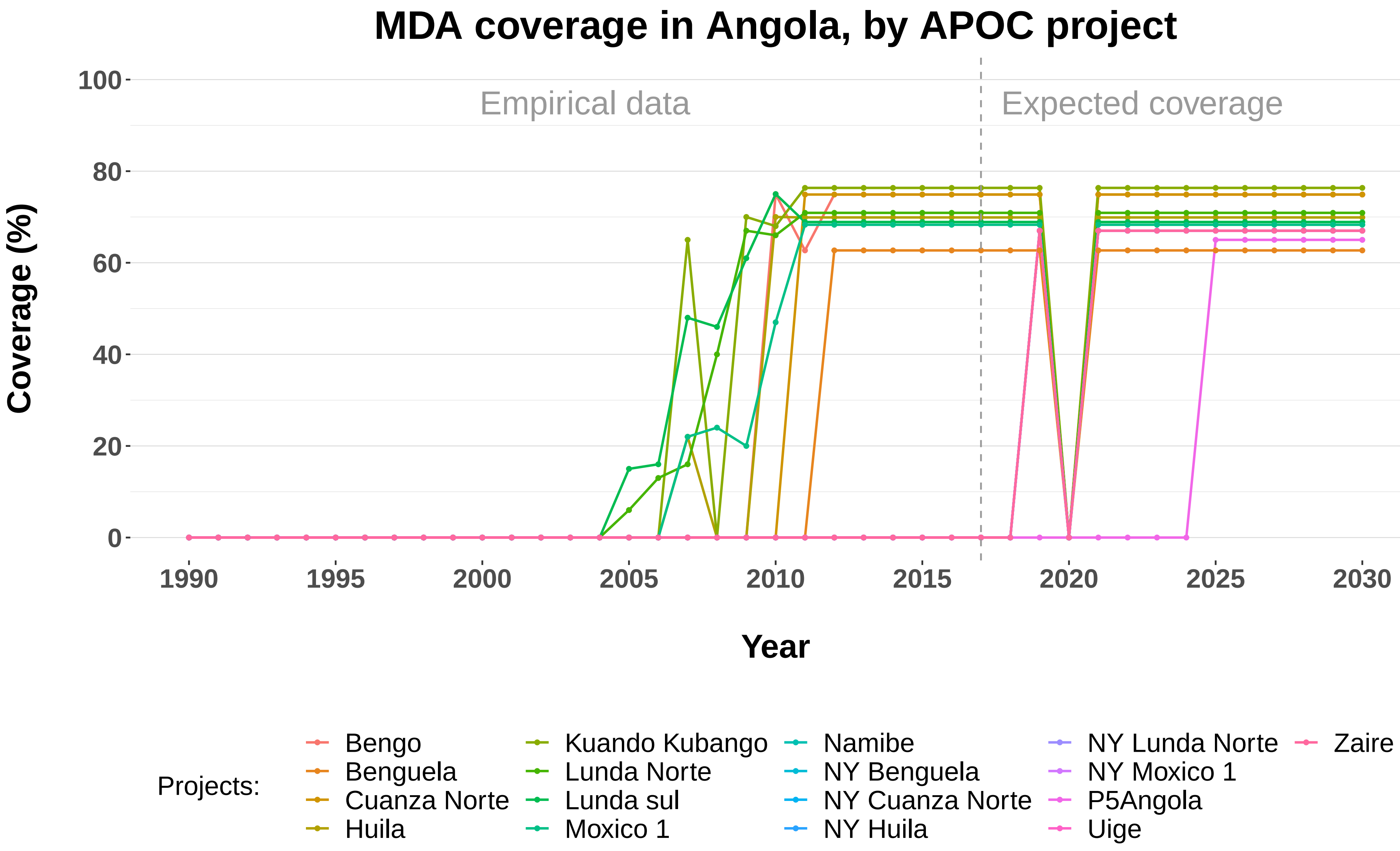

MDA coverage in Angola, by APOC project
Empirical data
Expected coverage
Coverage (%)
100
80
60
40
20
0
1990
1995
2000
2005
2010
2015
2020
2025
2030
Year
Projects:
Bengo
Benguela
Cuanza Norte
Huila
Kuando Kubango
Lunda Norte
Lunda sul
Moxico 1
Namibe
NY Benguela
NY Cuanza Norte
NY Huila
NY Lunda Norte
NY Moxico 1
P5Angola
Uige
Zaire

**Country:**	**Burundi**
Projects:	Bururi, Cibitoke-Bubanza, P5Burundi, Rutana
Bioclime:	Forest

	1990			2020			2030		
	N	%	DALYs	N	%	DALYs	N	%	DALYs
Total populaton at risk	1,333	100	_	3,007	100	_	3,944	100	_
**Infection:**									
Mf-positive	359	26.9	_	220	7.3	_	163	4.1	_
Mf-positive (age 5+)	358	26.9	_	220	7.3	_	163	4.1	_
Worm infected	472	35.4	_	432	14.4	_	300	7.6	_
**Clinical manifestations:**									
Palpable nodules	68	5.1	1	33	1.1	<0.5	25	0.6	<0.5
Reversible skin disease:									
Severe itch	46	3.5	8	27	0.9	5	14	0.4	3
Reactive skin disease	32	2.4	1	17	0.6	1	4	0.1	<0.5
**Reversible skin disease**	**78**	**5.9**	**10**	**44**	**1.5**	**6**	**17**	**0.4**	**3**
Irreversible skin disease:									
Mild depigmentation	12	0.9	<0.5	16	0.5	<0.5	13	0.3	<0.5
Severe depigmentation	11	0.8	1	14	0.5	1	11	0.3	1
Atrophy	<0.5	<0.05	<0.5	<0.5	<0.05	<0.5	<0.5	<0.05	<0.5
Hanging groin	<0.5	<0.05	<0.5	<0.5	<0.05	<0.5	<0.5	<0.05	<0.5
**Irreversible skin disease**	**23**	**1.7**	**1**	**30**	**1.0**	**1**	**24**	**0.6**	**1**
**Total skin disease (OSD)**	**168**	**12.6**	**11**	**107**	**3.6**	**7**	**67**	**1.7**	**4**
Eye disease (OED):									
Visual impairment	<0.5	<0.05	<0.5	<0.5	<0.05	<0.5	<0.5	<0.05	<0.5
Blindness	<0.5	<0.05	1	<0.5	<0.05	1	<0.5	<0.05	<0.5
**Eye disease (OED)**	**<0.5**	**<0.05**	**1**	**<0.5**	**<0.05**	**1**	**<0.5**	**<0.05**	**<0.5**
**Total all manifestations**	**169**	**12.7**	**12**	**108**	**3.6**	**8**	**67**	**1.7**	**4**

Note 1: Population at risk, number of cases (N) and DALYs are presented in thousands.

Note 2: Total skin disease is defined as the sum of the number of cases with palpable nodules, reversible and irreversible skin disease.

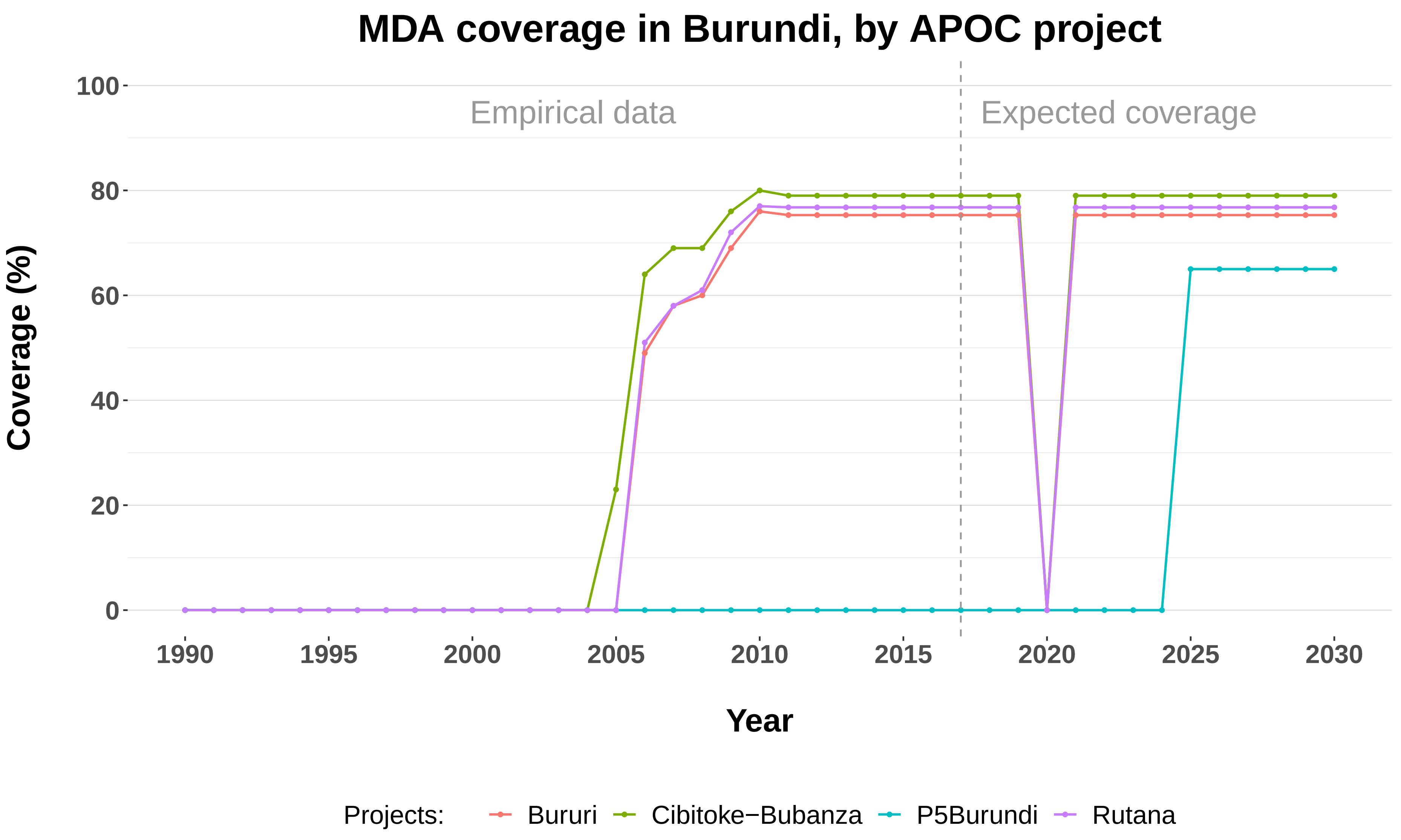
MDA coverage in Burundi, by APOC project
Empirical data
Expected coverage
Coverage (%)
100
80
60
40
20
0
1990
1995
2000
2005
2010
2015
2020
2025
2030
Year
Projects:
Bururi
Cibitoke−Bubanza
P5Burundi
Rutana

**Country:**	**Cameroon**
Projects:	Adamaoua 1, Adamaoua 2, Centre 1, Centre 2, Centre 3, East, Far North, Littoral 1, Littoral 2, Northern, Northwest, P20Cameroon, P5Cameroon, South, South West 1, South West 2, Western
Bioclime:	Savannah, Forest

	1990			2020			2030		
	N	%	DALYs	N	%	DALYs	N	%	DALYs
Total populaton at risk	5,121	100	_	11,555	100	_	15,156	100	_
**Infection:**									
Mf-positive	2,241	43.8	_	829	7.2	_	426	2.8	_
Mf-positive (age 5+)	2,235	43.6	_	829	7.2	_	426	2.8	_
Worm infected	2,671	52.2	_	1,969	17.0	_	945	6.2	_
**Clinical manifestations:**									
Palpable nodules	934	18.2	10	208	1.8	2	76	0.5	1
Reversible skin disease:									
Severe itch	435	8.5	79	90	0.8	16	36	0.2	7
Reactive skin disease	352	6.9	16	45	0.4	2	10	0.1	<0.5
**Reversible skin disease**	**787**	**15.4**	**95**	**135**	**1.2**	**18**	**46**	**0.3**	**7**
Irreversible skin disease:									
Mild depigmentation	90	1.8	1	99	0.9	1	75	0.5	1
Severe depigmentation	123	2.4	8	120	1.0	8	84	0.6	5
Atrophy	3	<0.05	<0.5	<0.5	<0.05	<0.5	<0.5	<0.05	<0.5
Hanging groin	5	0.1	2	3	<0.05	1	2	<0.05	1
**Irreversible skin disease**	**220**	**4.3**	**11**	**222**	**1.9**	**10**	**161**	**1.1**	**7**
**Total skin disease (OSD)**	**1,942**	**37.9**	**116**	**565**	**4.9**	**31**	**283**	**1.9**	**15**
Eye disease (OED):									
Visual impairment	32	0.6	1	28	0.2	1	18	0.1	1
Blindness	22	0.4	160	9	0.1	66	4	<0.05	27
**Eye disease (OED)**	**54**	**1.0**	**161**	**37**	**0.3**	**67**	**21**	**0.1**	**28**
**Total all manifestations**	**1,995**	**39.0**	**277**	**601**	**5.2**	**98**	**304**	**2.0**	**42**

Note 1: Population at risk, number of cases (N) and DALYs are presented in thousands.

Note 2: Total skin disease is defined as the sum of the number of cases with palpable nodules, reversible and irreversible skin disease.

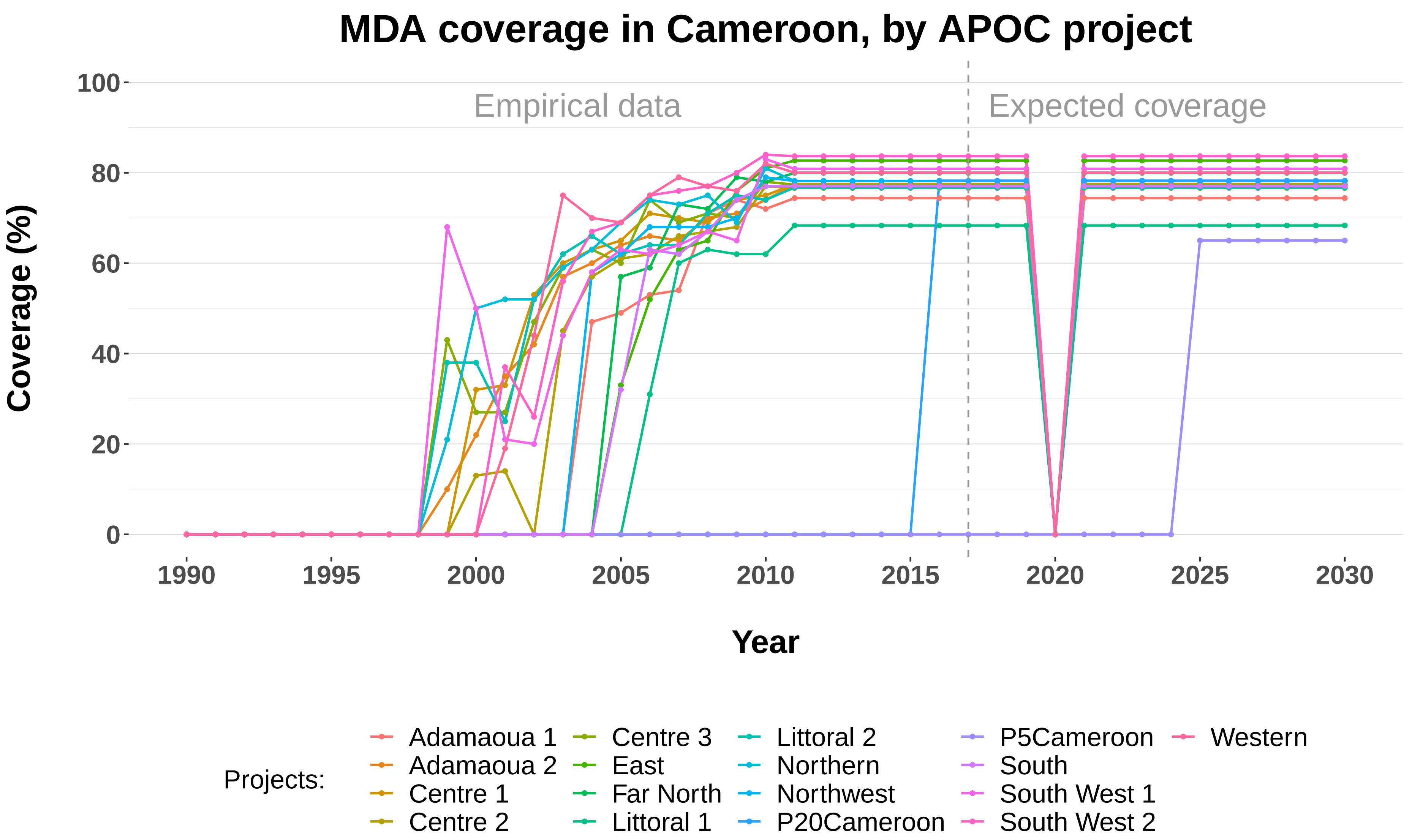
MDA coverage in Cameroon, by APOC project
Empirical data
Expected coverage
Coverage (%)
100
80
60
40
20
0
1990
1995
2000
2005
2010
2015
2020
2025
2030
Year
Projects:
Adamaoua 1
Adamaoua 2
Centre 1
Centre 2
Centre 3
East
Far North
Littoral 1
Littoral 2
Northern
Northwest
P20Cameroon
P5Cameroon
South
South West 1
South West 2
Western

**Country:**	**CAR**
Projects:	CAR region 3, CAR region 4, CAR region 5, CAR region 6, P20CAR, P5CAR
Bioclime:	Savannah, Forest

	1990			2020			2030		
	N	%	DALYs	N	%	DALYs	N	%	DALYs
Total populaton at risk	1,316	100	_	2,970	100	_	3,896	100	_
**Infection:**									
Mf-positive	549	41.7	_	126	4.3	_	62	1.6	_
Mf-positive (age 5+)	548	41.7	_	126	4.3	_	62	1.6	_
Worm infected	671	51.0	_	284	9.6	_	148	3.8	_
**Clinical manifestations:**									
Palpable nodules	195	14.8	2	31	1.0	<0.5	15	0.4	<0.5
Reversible skin disease:									
Severe itch	91	6.9	16	13	0.4	2	5	0.1	1
Reactive skin disease	69	5.2	3	8	0.3	<0.5	1	<0.05	<0.5
**Reversible skin disease**	**160**	**12.1**	**20**	**21**	**0.7**	**3**	**7**	**0.2**	**1**
Irreversible skin disease:									
Mild depigmentation	20	1.5	<0.5	17	0.6	<0.5	12	0.3	<0.5
Severe depigmentation	22	1.7	1	16	0.6	1	11	0.3	1
Atrophy	<0.5	<0.05	<0.5	<0.5	<0.05	<0.5	<0.5	<0.05	<0.5
Hanging groin	<0.5	<0.05	<0.5	<0.5	<0.05	<0.5	<0.5	<0.05	<0.5
**Irreversible skin disease**	**43**	**3.2**	**2**	**34**	**1.1**	**1**	**23**	**0.6**	**1**
**Total skin disease (OSD)**	**398**	**30.2**	**24**	**86**	**2.9**	**4**	**45**	**1.1**	**2**
Eye disease (OED):									
Visual impairment	37	2.8	1	27	0.9	1	17	0.4	1
Blindness	18	1.4	139	6	0.2	47	2	0.1	19
**Eye disease (OED)**	**55**	**4.1**	**140**	**33**	**1.1**	**48**	**20**	**0.5**	**20**
**Total all manifestations**	**452**	**34.4**	**164**	**119**	**4.0**	**52**	**64**	**1.7**	**22**

Note 1: Population at risk, number of cases (N) and DALYs are presented in thousands.

Note 2: Total skin disease is defined as the sum of the number of cases with palpable nodules, reversible and irreversible skin disease.

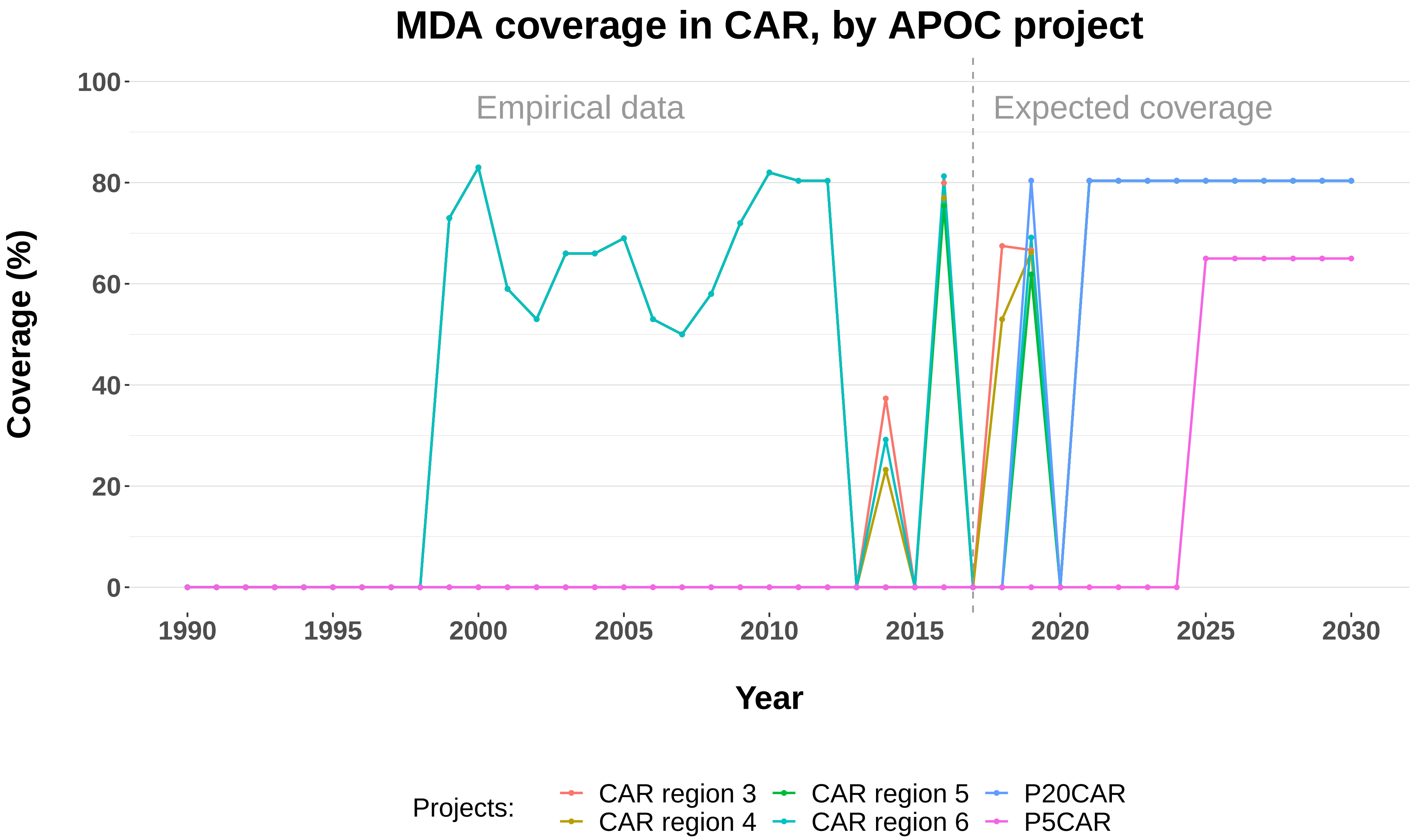
MDA coverage in CAR, by APOC project
Empirical data
Expected coverage
Coverage (%)
100
80
60
40
20
0
1990
1995
2000
2005
2010
2015
2020
2025
2030
Year
Projects:
CAR region 3
CAR region 4
CAR region 5
CAR region 6
P20CAR
P5CAR

**Country:**	**Chad**
Projects:	Chad, P5Chad, P5Chad propext
Bioclime:	Savannah

	1990			2020			2030		
	N	%	DALYs	N	%	DALYs	N	%	DALYs
Total populaton at risk	1,277	100	_	2,882	100	_	3,780	100	_
**Infection:**									
Mf-positive	379	29.7	_	109	3.8	_	85	2.3	_
Mf-positive (age 5+)	379	29.7	_	109	3.8	_	85	2.3	_
Worm infected	499	39.0	_	181	6.3	_	160	4.2	_
**Clinical manifestations:**									
Palpable nodules	85	6.7	1	21	0.7	<0.5	18	0.5	<0.5
Reversible skin disease:									
Severe itch	49	3.8	9	13	0.5	2	7	0.2	1
Reactive skin disease	34	2.6	2	9	0.3	<0.5	2	0.1	<0.5
**Reversible skin disease**	**83**	**6.5**	**10**	**22**	**0.8**	**3**	**9**	**0.2**	**1**
Irreversible skin disease:									
Mild depigmentation	12	0.9	<0.5	11	0.4	<0.5	9	0.2	<0.5
Severe depigmentation	11	0.8	1	9	0.3	1	6	0.2	<0.5
Atrophy	<0.5	<0.05	<0.5	<0.5	<0.05	<0.5	<0.5	<0.05	<0.5
Hanging groin	<0.5	<0.05	<0.5	<0.5	<0.05	<0.5	<0.5	<0.05	<0.5
**Irreversible skin disease**	**23**	**1.8**	**1**	**20**	**0.7**	**1**	**15**	**0.4**	**<0.5**
**Total skin disease (OSD)**	**191**	**14.9**	**12**	**63**	**2.2**	**4**	**42**	**1.1**	**2**
Eye disease (OED):									
Visual impairment	21	1.6	1	18	0.6	1	13	0.3	<0.5
Blindness	7	0.6	52	4	0.1	26	2	0.1	17
**Eye disease (OED)**	**28**	**2.2**	**53**	**21**	**0.7**	**27**	**15**	**0.4**	**17**
**Total all manifestations**	**219**	**17.1**	**65**	**84**	**2.9**	**31**	**58**	**1.5**	**19**

Note 1: Population at risk, number of cases (N) and DALYs are presented in thousands.

Note 2: Total skin disease is defined as the sum of the number of cases with palpable nodules, reversible and irreversible skin disease.

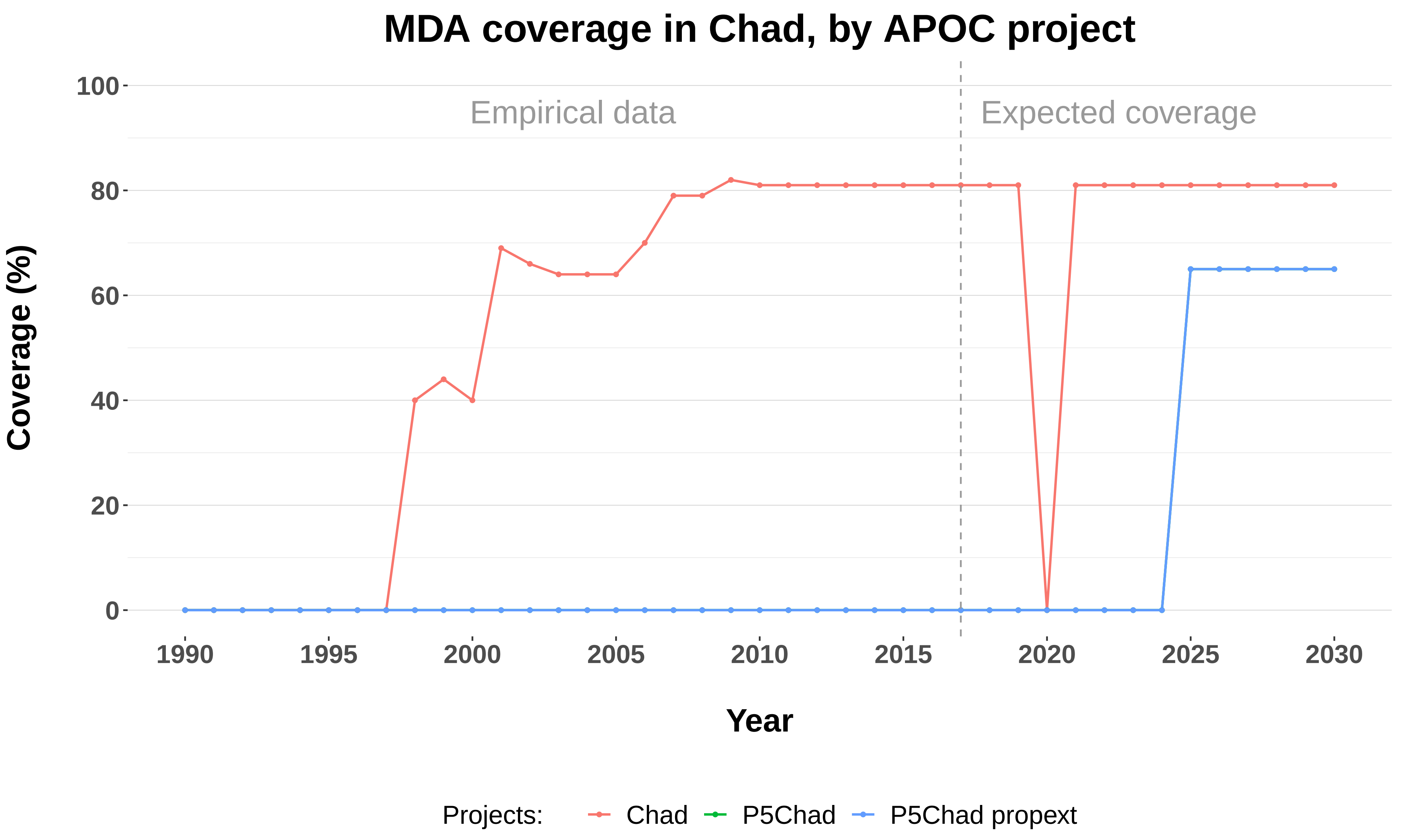
MDA coverage in Chad, by APOC project
Empirical data
Expected coverage
Coverage (%)
100
80
60
40
20
0
1990
1995
2000
2005
2010
2015
2020
2025
2030
Year
Projects:
Chad
P5Chad
P5Chad propext

**Country:**	**Congo**
Projects:	Congo 1, P20Congo, P5Congo
Bioclime:	Forest

	1990			2020			2030		
	N	%	DALYs	N	%	DALYs	N	%	DALYs
Total populaton at risk	794	100	_	1,791	100	_	2,350	100	_
**Infection:**									
Mf-positive	220	27.8	_	186	10.4	_	144	6.1	_
Mf-positive (age 5+)	220	27.7	_	186	10.4	_	144	6.1	_
Worm infected	287	36.1	_	281	15.7	_	248	10.6	_
**Clinical manifestations:**									
Palpable nodules	46	5.8	<0.5	40	2.2	<0.5	29	1.3	<0.5
Reversible skin disease:									
Severe itch	30	3.8	6	26	1.4	5	14	0.6	3
Reactive skin disease	21	2.7	1	17	0.9	1	4	0.2	<0.5
**Reversible skin disease**	**51**	**6.5**	**6**	**43**	**2.4**	**5**	**18**	**0.8**	**3**
Irreversible skin disease:									
Mild depigmentation	7	0.9	<0.5	11	0.6	<0.5	10	0.4	<0.5
Severe depigmentation	7	0.9	<0.5	10	0.6	1	9	0.4	1
Atrophy	<0.5	<0.05	<0.5	<0.5	<0.05	<0.5	<0.5	<0.05	<0.5
Hanging groin	<0.5	<0.05	<0.5	<0.5	<0.05	<0.5	<0.5	<0.05	<0.5
**Irreversible skin disease**	**15**	**1.8**	**1**	**21**	**1.2**	**1**	**18**	**0.8**	**1**
**Total skin disease (OSD)**	**112**	**14.1**	**8**	**104**	**5.8**	**7**	**66**	**2.8**	**4**
Eye disease (OED):									
Visual impairment	<0.5	<0.05	<0.5	<0.5	<0.05	<0.5	<0.5	<0.05	<0.5
Blindness	<0.5	<0.05	1	<0.5	<0.05	1	<0.5	<0.05	1
**Eye disease (OED)**	**<0.5**	**<0.05**	**1**	**<0.5**	**<0.05**	**1**	**<0.5**	**<0.05**	**1**
**Total all manifestations**	**112**	**14.1**	**9**	**104**	**5.8**	**8**	**66**	**2.8**	**5**

Note 1: Population at risk, number of cases (N) and DALYs are presented in thousands.

Note 2: Total skin disease is defined as the sum of the number of cases with palpable nodules, reversible and irreversible skin disease.

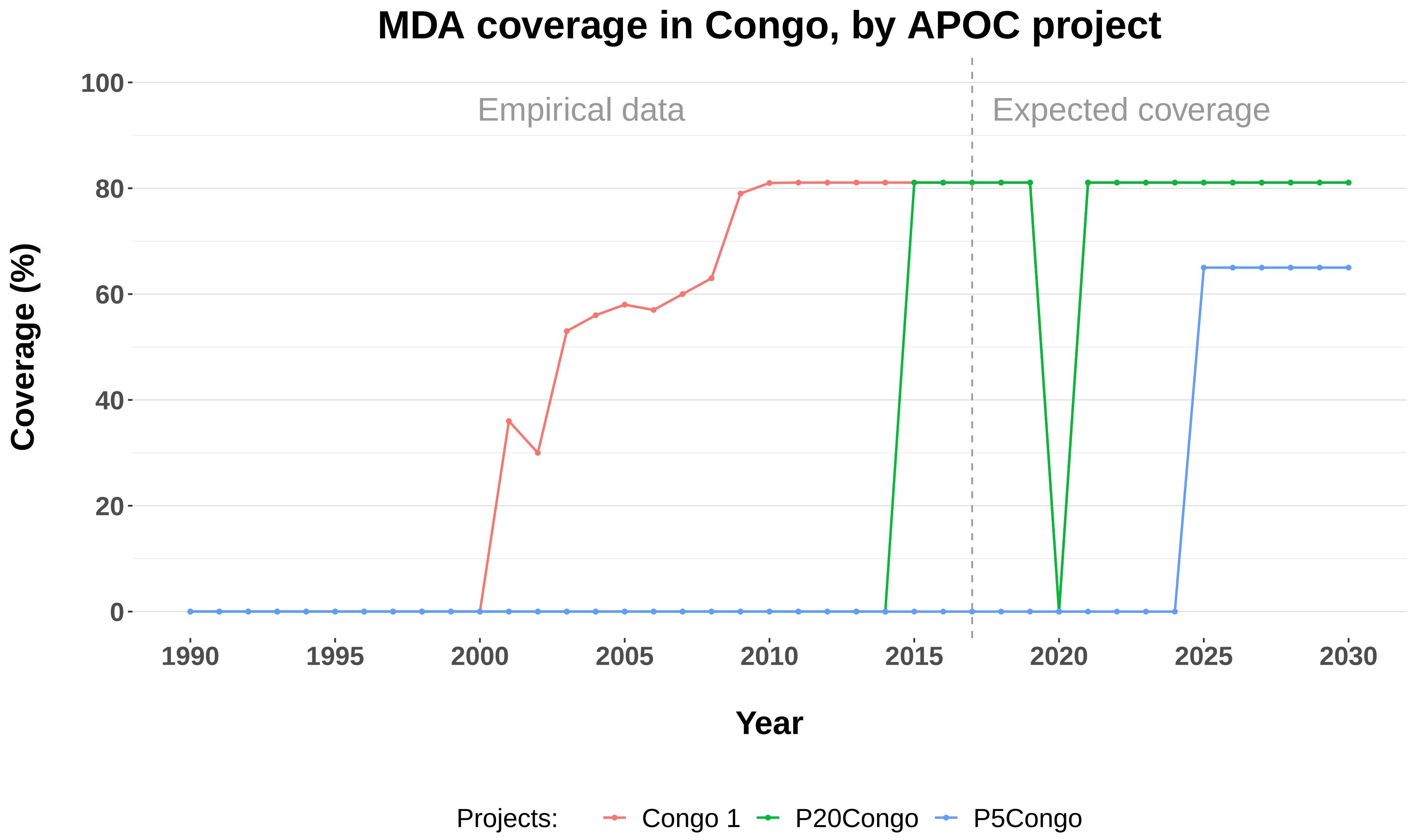

MDA coverage in Congo, by APOC project
Empirical data
Expected coverage
Coverage (%)
100
80
60
40
20
0
1990
1995
2000
2005
2010
2015
2020
2025
2030
Year
Projects:
Congo 1
P20Congo
P5Congo

**Country:**	**DRC**
Projects:	Bandundu, Bas-Congo Kinshasa, Butembo-Beni, Equateur-Kiri, Ituri-Nord, Ituri-Sud, Kasai, Kasongo, Katanga-Nord, Katanga-Sud, Lualaba, Lubutu, Masisi-Walikale, Mongala, NY Katanga-Nord, NY Lualaba, NY Masisi-Walikale, NY Rutshuru-Ngoma, NY Sankuru, NY Ueles, P20DRC, P5DRC, Rutshuru-Ngoma, Sankuru, Tshopo, Tshuapa, Ubangi-Nord, Ubangi-Sud, Ueles
Bioclime:	Forest

	1990			2020			2030		
	N	%	DALYs	N	%	DALYs	N	%	DALYs
Total populaton at risk	23,006	100	_	51,916	100	_	68,096	100	_
**Infection:**									
Mf-positive	9,075	39.4	_	7,182	13.8	_	3,094	4.5	_
Mf-positive (age 5+)	9,053	39.4	_	7,177	13.8	_	3,094	4.5	_
Worm infected	11,032	48.0	_	13,608	26.2	_	7,390	10.9	_
**Clinical manifestations:**									
Palpable nodules	3,366	14.6	36	2,464	4.7	26	795	1.2	8
Reversible skin disease:									
Severe itch	1,654	7.2	300	858	1.7	156	241	0.4	44
Reactive skin disease	1,308	5.7	61	386	0.7	18	55	0.1	3
**Reversible skin disease**	**2,963**	**12.9**	**361**	**1,244**	**2.4**	**174**	**296**	**0.4**	**46**
Irreversible skin disease:									
Mild depigmentation	351	1.5	4	496	1.0	5	399	0.6	4
Severe depigmentation	461	2.0	30	603	1.2	39	449	0.7	29
Atrophy	10	<0.05	<0.5	2	<0.05	<0.5	<0.5	<0.05	<0.5
Hanging groin	21	0.1	8	20	<0.05	8	12	<0.05	5
**Irreversible skin disease**	**843**	**3.7**	**42**	**1,121**	**2.2**	**52**	**860**	**1.3**	**38**
**Total skin disease (OSD)**	**7,172**	**31.2**	**439**	**4,829**	**9.3**	**252**	**1,951**	**2.9**	**92**
Eye disease (OED):									
Visual impairment	30	0.1	1	34	0.1	1	22	<0.05	1
Blindness	41	0.2	286	30	0.1	214	13	<0.05	93
**Eye disease (OED)**	**70**	**0.3**	**287**	**64**	**0.1**	**215**	**35**	**0.1**	**94**
**Total all manifestations**	**7,242**	**31.5**	**726**	**4,893**	**9.4**	**467**	**1,986**	**2.9**	**186**

Note 1: Population at risk, number of cases (N) and DALYs are presented in thousands.

Note 2: Total skin disease is defined as the sum of the number of cases with palpable nodules, reversible and irreversible skin disease.

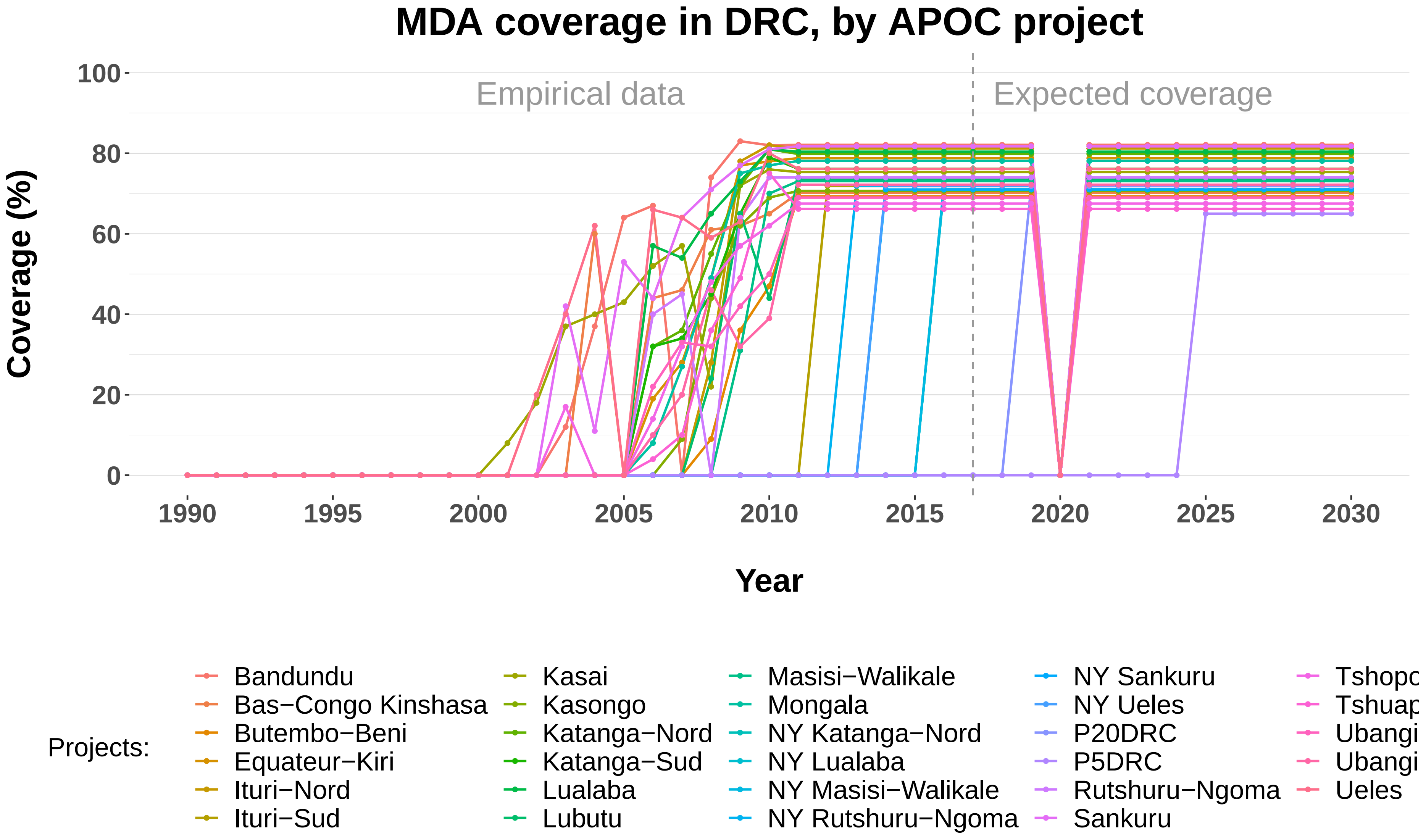

**Country:**	**Equatorial Guinea**
Projects:	Bioko, P5EqGuinea
Bioclime:	Forest

	1990			2020			2030		
	N	%	DALYs	N	%	DALYs	N	%	DALYs
Total populaton at risk	199	100	_	448	100	_	588	100	_
**Infection:**									
Mf-positive	76	38.2	_	<0.5	<0.05	_	<0.5	<0.05	_
Mf-positive (age 5+)	75	37.9	_	<0.5	<0.05	_	<0.5	<0.05	_
Worm infected	89	44.8	_	1	0.2	_	<0.5	<0.05	_
**Clinical manifestations:**									
Palpable nodules	35	17.7	<0.5	<0.5	<0.05	<0.5	<0.5	<0.05	<0.5
Reversible skin disease:									
Severe itch	19	9.3	3	<0.5	<0.05	<0.5	<0.5	<0.05	<0.5
Reactive skin disease	17	8.3	1	<0.5	<0.05	<0.5	<0.5	<0.05	<0.5
**Reversible skin disease**	**35**	**17.7**	**4**	**<0.5**	**<0.05**	**<0.5**	**<0.5**	**<0.05**	**<0.5**
Irreversible skin disease:									
Mild depigmentation	3	1.7	<0.5	2	0.4	<0.5	1	0.2	<0.5
Severe depigmentation	6	3.2	<0.5	3	0.6	<0.5	2	0.3	<0.5
Atrophy	<0.5	0.2	<0.5	<0.5	<0.05	<0.5	<0.5	<0.05	<0.5
Hanging groin	1	0.5	<0.5	<0.5	<0.05	<0.5	<0.5	<0.05	<0.5
**Irreversible skin disease**	**11**	**5.6**	**1**	**5**	**1.1**	**<0.5**	**3**	**0.5**	**<0.5**
**Total skin disease (OSD)**	**81**	**41.0**	**5**	**5**	**1.1**	**<0.5**	**3**	**0.5**	**<0.5**
Eye disease (OED):									
Visual impairment	1	0.3	<0.5	<0.5	0.1	<0.5	<0.5	<0.05	<0.5
Blindness	1	0.7	11	<0.5	<0.05	1	<0.5	<0.05	<0.5
**Eye disease (OED)**	**2**	**1.1**	**11**	**<0.5**	**0.1**	**1**	**<0.5**	**<0.05**	**<0.5**
**Total all manifestations**	**83**	**42.0**	**16**	**5**	**1.2**	**1**	**3**	**0.6**	**1**

Note 1: Population at risk, number of cases (N) and DALYs are presented in thousands.

Note 2: Total skin disease is defined as the sum of the number of cases with palpable nodules, reversible and irreversible skin disease.

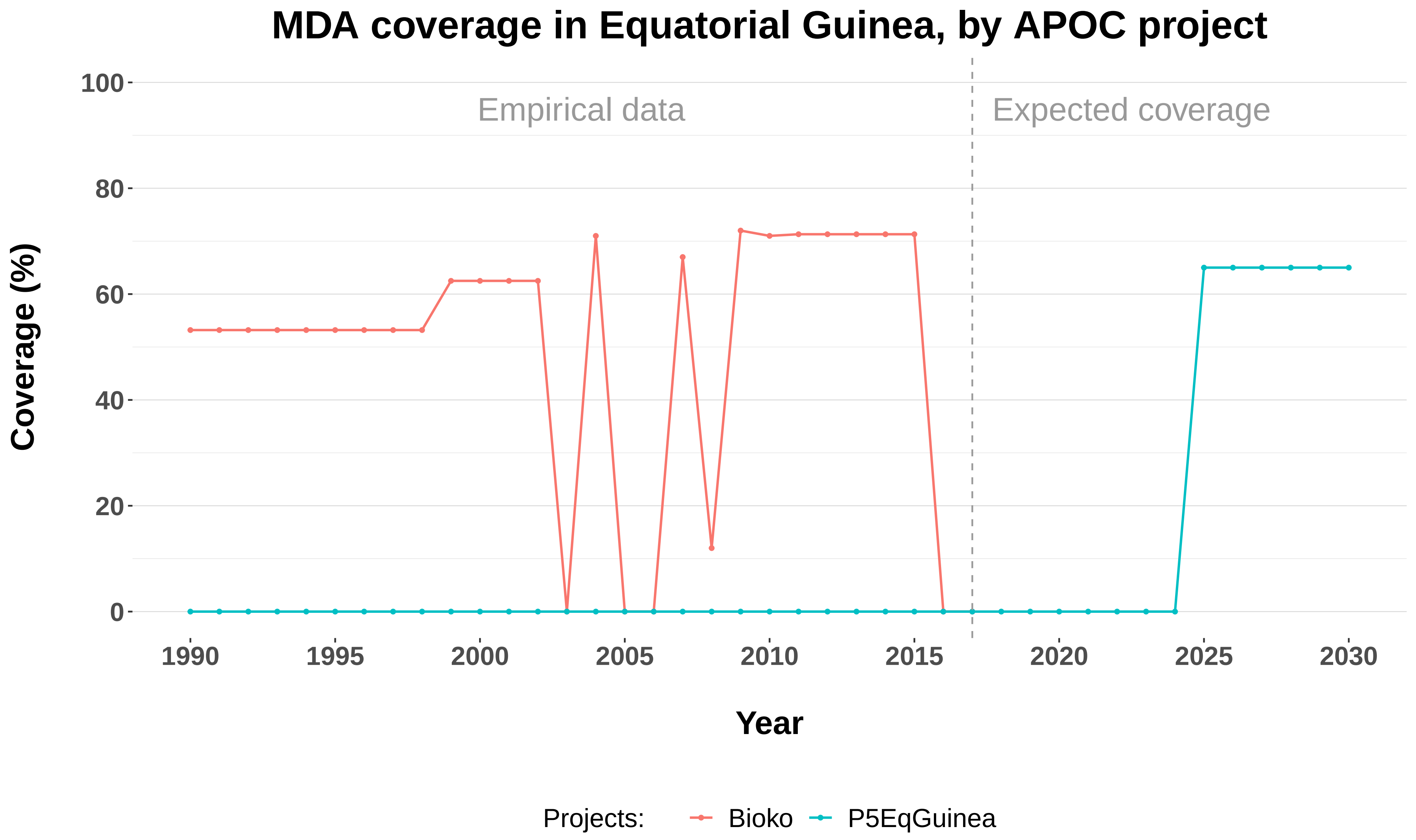

Note: We assume that most MDA interventions on the mainland of Equatorial Guinea would continue until at least 2030. We assume no ivermectic MDA for Bioko since 2018 as the island is currently under post-treatment surveillance [1-3]‡.

**Country:**	**Ethiopia**
Projects:	Assosa, Bench-Maji, East Wellega, Gambella, Horo Guduru, Illubabor, Jimma, Kaffa-Sheka, Kamashi, Metekel, North Gondar, NY East Wellega, NY West Wellega, P20Ethiopia, P5Ethiopia, West Shewa, West Wellega
Bioclime:	Forest

	1990			2020			2030		
	N	%	DALYs	N	%	DALYs	N	%	DALYs
Total populaton at risk	6,727	100	_	15,180	100	_	19,911	100	_
**Infection:**									
Mf-positive	2,206	32.8	_	1,614	10.6	_	681	3.4	_
Mf-positive (age 5+)	2,204	32.8	_	1,613	10.6	_	681	3.4	_
Worm infected	2,816	41.9	_	2,969	19.6	_	1,697	8.5	_
**Clinical manifestations:**									
Palpable nodules	581	8.6	6	381	2.5	4	126	0.6	1
Reversible skin disease:									
Severe itch	317	4.7	58	215	1.4	39	54	0.3	10
Reactive skin disease	225	3.3	10	123	0.8	6	10	<0.05	<0.5
**Reversible skin disease**	**542**	**8.1**	**68**	**338**	**2.2**	**45**	**64**	**0.3**	**10**
Irreversible skin disease:									
Mild depigmentation	75	1.1	1	108	0.7	1	88	0.4	1
Severe depigmentation	75	1.1	5	99	0.6	6	74	0.4	5
Atrophy	<0.5	<0.05	<0.5	<0.5	<0.05	<0.5	<0.5	<0.05	<0.5
Hanging groin	1	<0.05	<0.5	1	<0.05	<0.5	<0.5	<0.05	<0.5
**Irreversible skin disease**	**152**	**2.3**	**6**	**207**	**1.4**	**8**	**163**	**0.8**	**6**
**Total skin disease (OSD)**	**1,275**	**19.0**	**80**	**925**	**6.1**	**56**	**353**	**1.8**	**18**
Eye disease (OED):									
Visual impairment	2	<0.05	<0.5	3	<0.05	<0.5	1	<0.05	<0.5
Blindness	2	<0.05	12	2	<0.05	9	1	<0.05	4
**Eye disease (OED)**	**4**	**0.1**	**12**	**4**	**<0.05**	**9**	**2**	**<0.05**	**4**
**Total all manifestations**	**1,279**	**19.0**	**92**	**930**	**6.1**	**66**	**355**	**1.8**	**21**

Note 1: Population at risk, number of cases (N) and DALYs are presented in thousands.

Note 2: Total skin disease is defined as the sum of the number of cases with palpable nodules, reversible and irreversible skin disease.

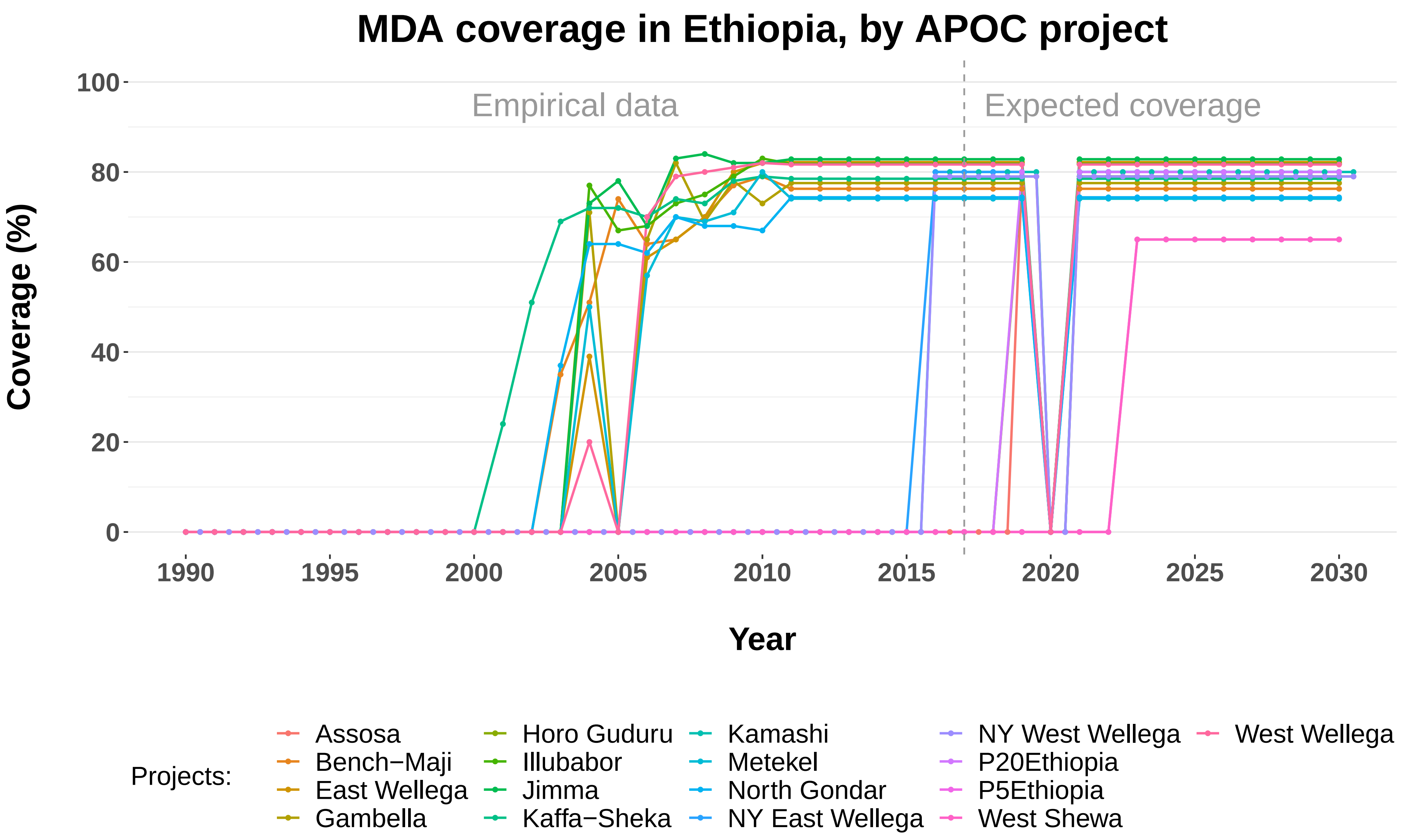
MDA coverage in Ethiopia, by APOC project
Empirical data
Expected coverage
Coverage (%)
100
80
60
40
20
0
1990
1995
2000
2005
2010
2015
2020
2025
2030
Year
Projects:
Assosa
Bench−Maji
East Wellega
Gambella
Horo Guduru
Illubabor
Jimma
Kaffa−Sheka
Kamashi
Metekel
North Gondar
NY East Wellega
NY West Wellega
P20Ethiopia
P5Ethiopia
West Shewa
West Wellega

**Country:**	**Gabon**
Projects:	P5Gabon
Bioclime:	Forest

	1990			2020			2030		
	N	%	DALYs	N	%	DALYs	N	%	DALYs
Total populaton at risk	49	100	_	112	100	_	146	100	_
**Infection:**									
Mf-positive	11	21.6	_	22	20.0	_	17	11.9	_
Mf-positive (age 5+)	11	21.6	_	22	20.0	_	17	11.9	_
Worm infected	15	29.3	_	31	27.6	_	32	22.1	_
**Clinical manifestations:**									
Palpable nodules	1	2.5	<0.5	3	2.3	<0.5	2	1.5	<0.5
Reversible skin disease:									
Severe itch	1	2.6	<0.5	3	2.3	<0.5	1	0.9	<0.5
Reactive skin disease	1	1.7	<0.5	2	1.5	<0.5	<0.5	0.3	<0.5
**Reversible skin disease**	**2**	**4.3**	**<0.5**	**4**	**3.8**	**1**	**2**	**1.2**	**<0.5**
Irreversible skin disease:									
Mild depigmentation	<0.5	0.7	<0.5	1	0.7	<0.5	1	0.5	<0.5
Severe depigmentation	<0.5	0.6	<0.5	1	0.5	<0.5	1	0.4	<0.5
Atrophy	<0.5	<0.05	<0.5	<0.5	<0.05	<0.5	<0.5	<0.05	<0.5
Hanging groin	<0.5	<0.05	<0.5	<0.5	<0.05	<0.5	<0.5	<0.05	<0.5
**Irreversible skin disease**	**1**	**1.3**	**<0.5**	**1**	**1.2**	**<0.5**	**1**	**0.9**	**<0.5**
**Total skin disease (OSD)**	**4**	**8.1**	**<0.5**	**8**	**7.3**	**1**	**5**	**3.6**	**<0.5**
Eye disease (OED):									
Visual impairment	<0.5	<0.05	<0.5	<0.5	<0.05	<0.5	<0.5	<0.05	<0.5
Blindness	<0.5	<0.05	<0.5	<0.5	<0.05	<0.5	<0.5	<0.05	<0.5
**Eye disease (OED)**	**<0.5**	**<0.05**	**<0.5**	**<0.5**	**<0.05**	**<0.5**	**<0.5**	**<0.05**	**<0.5**
**Total all manifestations**	**4**	**8.1**	**<0.5**	**8**	**7.3**	**1**	**5**	**3.6**	**<0.5**

Note 1: Population at risk, number of cases (N) and DALYs are presented in thousands.

Note 2: Total skin disease is defined as the sum of the number of cases with palpable nodules, reversible and irreversible skin disease.

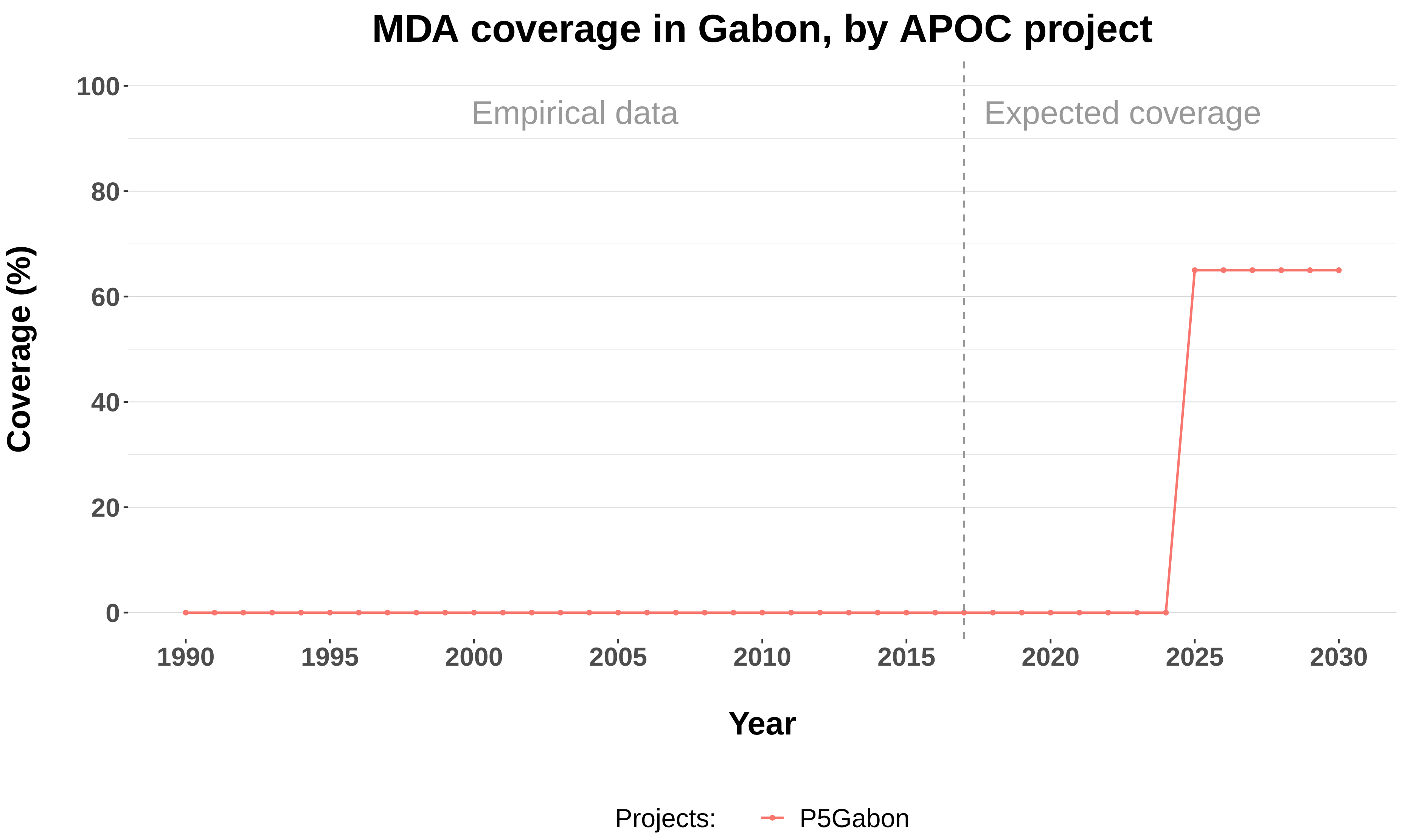

MDA coverage in Gabon, by APOC project
Empirical data
Expected coverage
Coverage (%)
100
80
60
40
20
0
1990
1995
2000
2005
2010
2015
2020
2025
2030
Year
Projects:
P5Gabon

**Country:**	**Liberia**
Projects:	All_Liberia
Bioclime:	Forest

	1990			2020			2030		
	N	%	DALYs	N	%	DALYs	N	%	DALYs
Total populaton at risk	881	100	_	1,987	100	_	2,606	100	_
**Infection:**									
Mf-positive	343	39.0	_	5	0.3	_	<0.5	<0.05	_
Mf-positive (age 5+)	343	38.9	_	5	0.3	_	<0.5	<0.05	_
Worm infected	455	51.7	_	54	2.7	_	2	0.1	_
**Clinical manifestations:**									
Palpable nodules	100	11.3	1	2	0.1	<0.5	<0.5	<0.05	<0.5
Reversible skin disease:									
Severe itch	43	4.9	8	<0.5	<0.05	<0.5	<0.5	<0.05	<0.5
Reactive skin disease	29	3.3	1	<0.5	<0.05	<0.5	<0.5	<0.05	<0.5
**Reversible skin disease**	**72**	**8.2**	**9**	**1**	**<0.05**	**<0.5**	**<0.5**	**<0.05**	**<0.5**
Irreversible skin disease:									
Mild depigmentation	10	1.1	<0.5	7	0.4	<0.5	5	0.2	<0.5
Severe depigmentation	9	1.0	1	6	0.3	<0.5	3	0.1	<0.5
Atrophy	<0.5	<0.05	<0.5	<0.5	<0.05	<0.5	<0.5	<0.05	<0.5
Hanging groin	<0.5	<0.05	<0.5	<0.5	<0.05	<0.5	<0.5	<0.05	<0.5
**Irreversible skin disease**	**19**	**2.1**	**1**	**13**	**0.7**	**<0.5**	**8**	**0.3**	**<0.5**
**Total skin disease (OSD)**	**191**	**21.7**	**11**	**15**	**0.8**	**1**	**8**	**0.3**	**<0.5**
Eye disease (OED):									
Visual impairment	<0.5	<0.05	<0.5	<0.5	<0.05	<0.5	<0.5	<0.05	<0.5
Blindness	<0.5	<0.05	<0.5	<0.5	<0.05	<0.5	<0.5	<0.05	<0.5
**Eye disease (OED)**	**<0.5**	**<0.05**	**<0.5**	**<0.5**	**<0.05**	**<0.5**	**<0.5**	**<0.05**	**<0.5**
**Total all manifestations**	**191**	**21.7**	**11**	**15**	**0.8**	**1**	**8**	**0.3**	**<0.5**

Note 1: Population at risk, number of cases (N) and DALYs are presented in thousands.

Note 2: Total skin disease is defined as the sum of the number of cases with palpable nodules, reversible and irreversible skin disease.

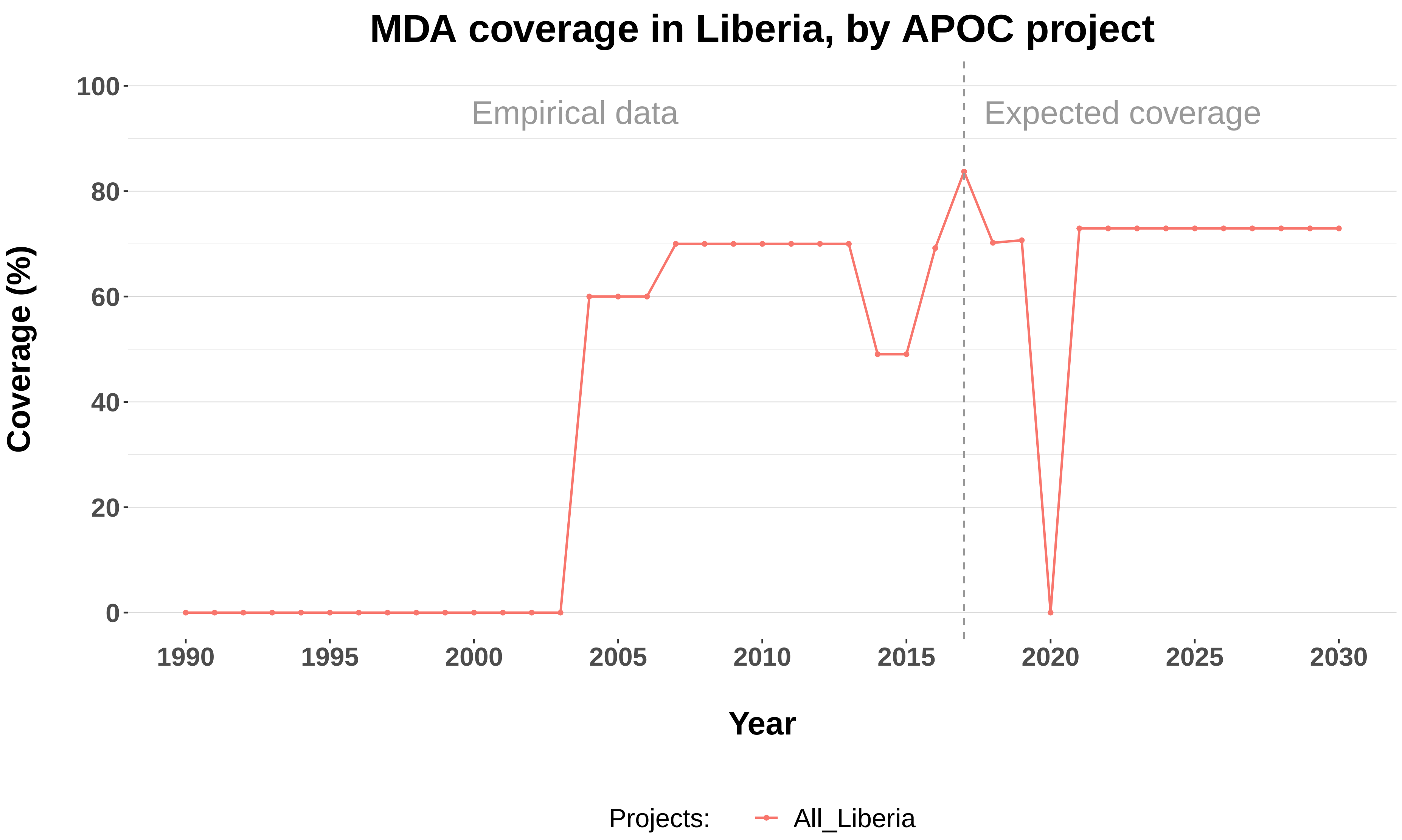
MDA coverage in Liberia, by APOC project
Empirical data
Expected coverage
Coverage (%)
0
20
40
60
80
100
1990
1995
2000
2005
2010
2015
2020
2025
2030
Year
Projects:
All_Liberia

**Country:**	**Malawi**
Projects:	Malawi Extension, Thyolo Mwanza
Bioclime:	Forest

	1990			2020			2030		
	N	%	DALYs	N	%	DALYs	N	%	DALYs
Total populaton at risk	1,172	100	_	2,645	100	_	3,469	100	_
**Infection:**									
Mf-positive	273	23.3	_	1	<0.05	_	<0.5	<0.05	_
Mf-positive (age 5+)	273	23.3	_	1	<0.05	_	<0.5	<0.05	_
Worm infected	374	31.9	_	41	1.5	_	<0.5	<0.05	_
**Clinical manifestations:**									
Palpable nodules	41	3.5	<0.5	<0.5	<0.05	<0.5	<0.5	<0.05	<0.5
Reversible skin disease:									
Severe itch	32	2.8	6	<0.5	<0.05	<0.5	<0.5	<0.05	<0.5
Reactive skin disease	22	1.8	1	<0.5	<0.05	<0.5	<0.5	<0.05	<0.5
**Reversible skin disease**	**54**	**4.6**	**7**	**<0.5**	**<0.05**	**<0.5**	**<0.5**	**<0.05**	**<0.5**
Irreversible skin disease:									
Mild depigmentation	9	0.7	<0.5	7	0.3	<0.5	5	0.1	<0.5
Severe depigmentation	8	0.6	<0.5	5	0.2	<0.5	3	0.1	<0.5
Atrophy	<0.5	<0.05	<0.5	<0.5	<0.05	<0.5	<0.5	<0.05	<0.5
Hanging groin	<0.5	<0.05	<0.5	<0.5	<0.05	<0.5	<0.5	<0.05	<0.5
**Irreversible skin disease**	**16**	**1.4**	**1**	**12**	**0.5**	**<0.5**	**8**	**0.2**	**<0.5**
**Total skin disease (OSD)**	**111**	**9.5**	**8**	**13**	**0.5**	**<0.5**	**8**	**0.2**	**<0.5**
Eye disease (OED):									
Visual impairment	<0.5	<0.05	<0.5	<0.5	<0.05	<0.5	<0.5	<0.05	<0.5
Blindness	<0.5	<0.05	<0.5	<0.5	<0.05	<0.5	<0.5	<0.05	<0.5
**Eye disease (OED)**	**<0.5**	**<0.05**	**<0.5**	**<0.5**	**<0.05**	**<0.5**	**<0.5**	**<0.05**	**<0.5**
**Total all manifestations**	**111**	**9.5**	**8**	**13**	**0.5**	**1**	**8**	**0.2**	**<0.5**

Note 1: Population at risk, number of cases (N) and DALYs are presented in thousands.

Note 2: Total skin disease is defined as the sum of the number of cases with palpable nodules, reversible and irreversible skin disease.

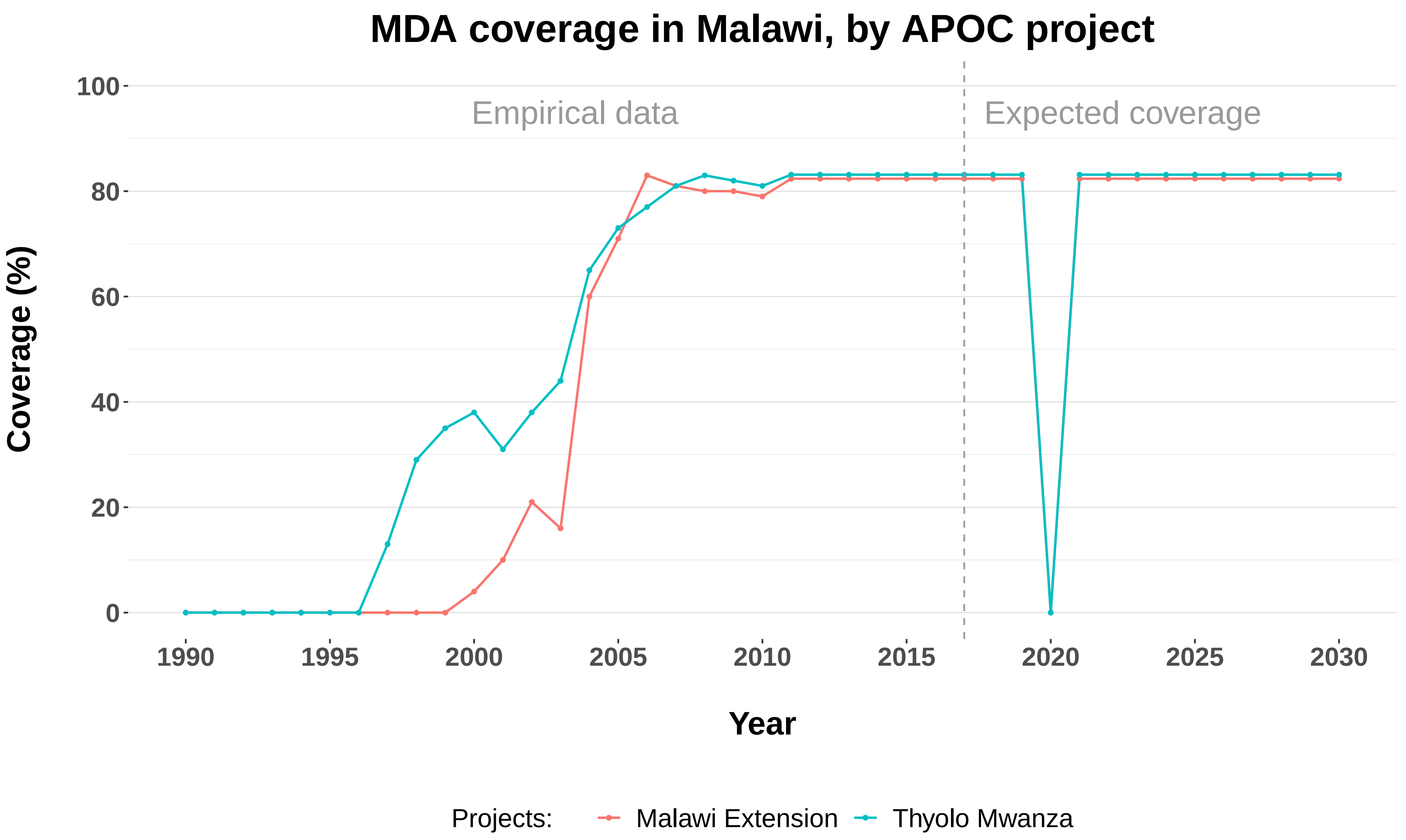
MDA coverage in Malawi, by APOC project
Empirical data
Expected coverage
Coverage (%)
100
80
60
40
20
0
1990
1995
2000
2005
2010
2015
2020
2025
2030
Year
Projects:
Malawi Extension
Thyolo Mwanza

**Country:**	**Mozambique**
Projects:	P20Mozambique, P5Mozambique
Bioclime:	Forest

	1990			2020			2030		
	N	%	DALYs	N	%	DALYs	N	%	DALYs
Total populaton at risk	36	100	_	82	100	_	107	100	_
**Infection:**									
Mf-positive	7	20.0	_	14	17.2	_	6	5.7	_
Mf-positive (age 5+)	7	20.0	_	14	17.2	_	6	5.7	_
Worm infected	10	27.6	_	20	24.9	_	16	15.0	_
**Clinical manifestations:**									
Palpable nodules	1	2.0	<0.5	1	1.7	<0.5	1	0.6	<0.5
Reversible skin disease:									
Severe itch	1	2.3	<0.5	2	1.9	<0.5	<0.5	0.4	<0.5
Reactive skin disease	1	1.6	<0.5	1	1.2	<0.5	<0.5	0.1	<0.5
**Reversible skin disease**	**1**	**3.9**	**<0.5**	**3**	**3.1**	**<0.5**	**1**	**0.5**	**<0.5**
Irreversible skin disease:									
Mild depigmentation	<0.5	0.7	<0.5	<0.5	0.6	<0.5	<0.5	0.4	<0.5
Severe depigmentation	<0.5	0.6	<0.5	<0.5	0.5	<0.5	<0.5	0.3	<0.5
Atrophy	<0.5	<0.05	<0.5	<0.5	<0.05	<0.5	<0.5	<0.05	<0.5
Hanging groin	<0.5	<0.05	<0.5	<0.5	<0.05	<0.5	<0.5	<0.05	<0.5
**Irreversible skin disease**	**<0.5**	**1.2**	**<0.5**	**1**	**1.0**	**<0.5**	**1**	**0.7**	**<0.5**
**Total skin disease (OSD)**	**3**	**7.1**	**<0.5**	**5**	**5.9**	**<0.5**	**2**	**1.8**	**<0.5**
Eye disease (OED):									
Visual impairment	<0.5	<0.05	<0.5	<0.5	<0.05	<0.5	<0.5	<0.05	<0.5
Blindness	<0.5	<0.05	<0.5	<0.5	<0.05	<0.5	<0.5	<0.05	<0.5
**Eye disease (OED)**	**<0.5**	**<0.05**	**<0.5**	**<0.5**	**<0.05**	**<0.5**	**<0.5**	**<0.05**	**<0.5**
**Total all manifestations**	**3**	**7.2**	**<0.5**	**5**	**5.9**	**<0.5**	**2**	**1.8**	**<0.5**

Note 1: Population at risk, number of cases (N) and DALYs are presented in thousands.

Note 2: Total skin disease is defined as the sum of the number of cases with palpable nodules, reversible and irreversible skin disease.

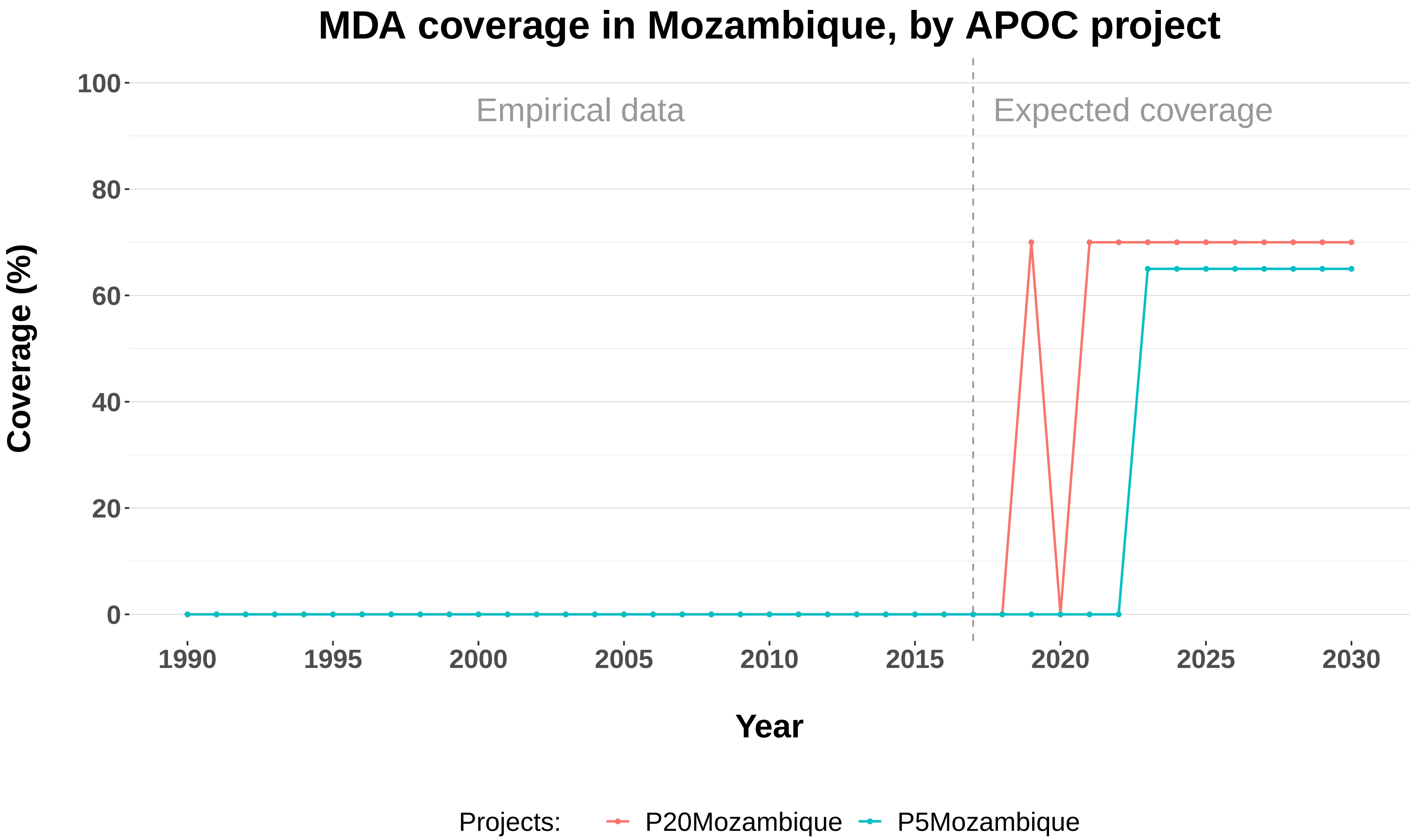
MDA coverage in Mozambique, by APOC project
Empirical data
Expected coverage
Coverage (%)
100
80
60
40
20
0
1990
1995
2000
2005
2010
2015
2020
2025
2030
Year
Projects:
P20Mozambique
P5Mozambique

**Country:**	**Nigeria**
Projects:	Adamawa, Akwa Ibom, Bauchi, Benue, Borno, Cross River, Edo Delta, Ekiti, Enugu Anambra Ebony, FCT, Gombe, Imo Abia, Jigawa, Kaduna, Kano, Kebbi, Kogi, Kwara, Niger, Ogun, Ondo, Osun, Oyo, P20Nigeria, P5Nigeria, Plateau Nassarawa, Plateau Nassarawa LF, Taraba, Yobe, Zamfara
Bioclime:	Savannah, Forest

	1990			2020			2030		
	N	%	DALYs	N	%	DALYs	N	%	DALYs
Total populaton at risk	28,322	100	_	63,911	100	_	83,829	100	_
**Infection:**									
Mf-positive	7,352	26.0	_	2,084	3.3	_	1,475	1.8	_
Mf-positive (age 5+)	7,346	25.9	_	2,083	3.3	_	1,475	1.8	_
Worm infected	9,741	34.4	_	3,854	6.0	_	2,911	3.5	_
**Clinical manifestations:**									
Palpable nodules	1,430	5.0	15	278	0.4	3	187	0.2	2
Reversible skin disease:									
Severe itch	970	3.4	176	241	0.4	44	119	0.1	22
Reactive skin disease	669	2.4	31	149	0.2	7	30	<0.05	1
**Reversible skin disease**	**1,638**	**5.8**	**207**	**390**	**0.6**	**51**	**149**	**0.2**	**23**
Irreversible skin disease:									
Mild depigmentation	245	0.9	3	223	0.3	2	168	0.2	2
Severe depigmentation	220	0.8	14	184	0.3	12	129	0.2	8
Atrophy	1	<0.05	<0.5	<0.5	<0.05	<0.5	<0.5	<0.05	<0.5
Hanging groin	2	<0.05	1	1	<0.05	<0.5	<0.5	<0.05	<0.5
**Irreversible skin disease**	**468**	**1.7**	**17**	**408**	**0.6**	**15**	**298**	**0.4**	**10**
**Total skin disease (OSD)**	**3,536**	**12.5**	**240**	**1,076**	**1.7**	**68**	**634**	**0.8**	**35**
Eye disease (OED):									
Visual impairment	224	0.8	7	217	0.3	7	169	0.2	5
Blindness	72	0.3	498	45	0.1	310	29	<0.05	202
**Eye disease (OED)**	**296**	**1.0**	**505**	**261**	**0.4**	**316**	**198**	**0.2**	**207**
**Total all manifestations**	**3,832**	**13.5**	**744**	**1,338**	**2.1**	**385**	**832**	**1.0**	**242**

Note 1: Population at risk, number of cases (N) and DALYs are presented in thousands.

Note 2: Total skin disease is defined as the sum of the number of cases with palpable nodules, reversible and irreversible skin disease.

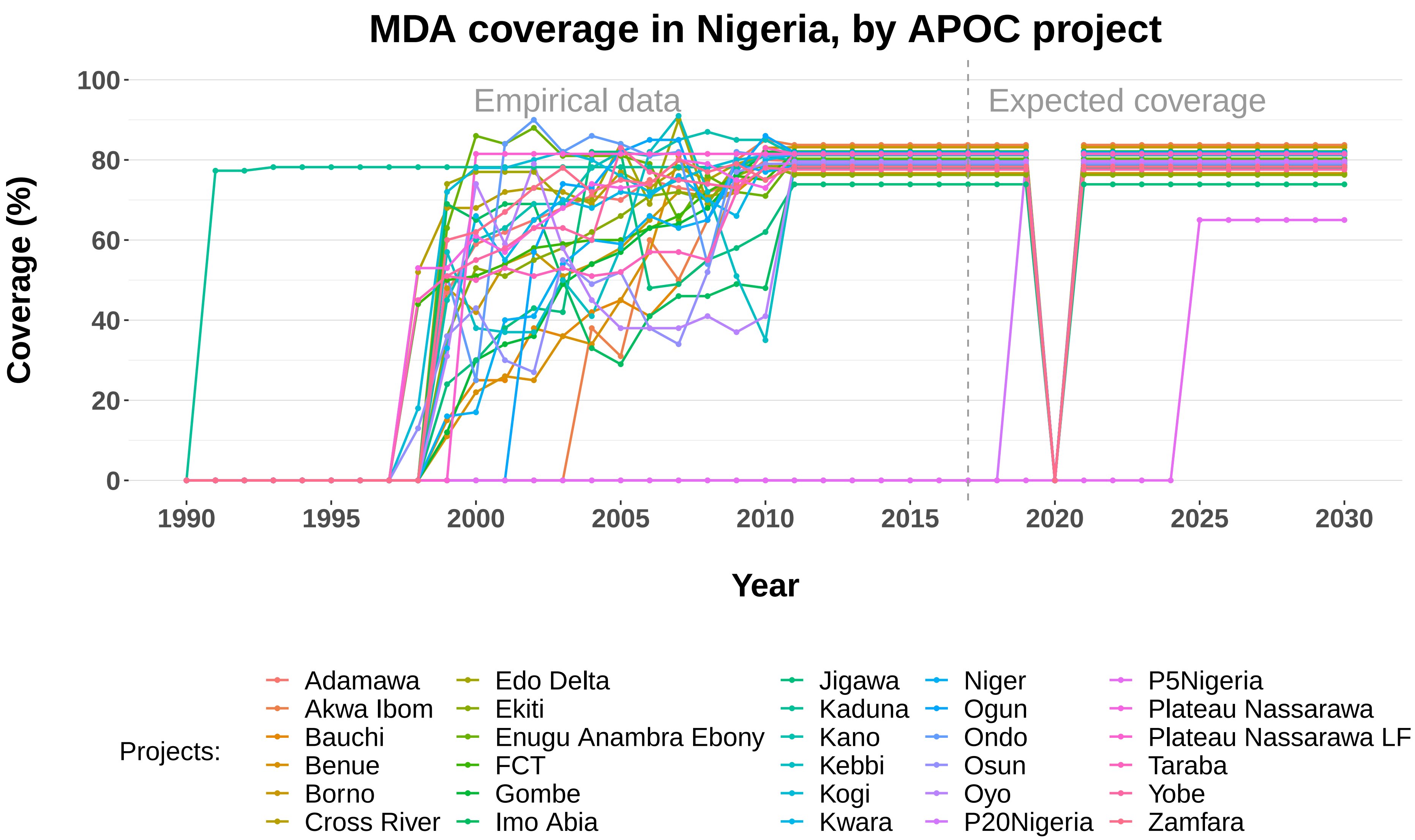

Note: We assume that most MDA interventions would continue until at least 2030 (even though some foci are considered for post-treatment surveillance). We assume no ivermectin MDA for Plateau and Nassarawa since 2018 as onchocerciasis transmission has been interrupted.[4][‡, page 354].

Country: **South Sudan**

Projects: East Bahr El Ghazal, East Equatoria, P20SouthSudan, P5SouthSudan, Upper Nile, West Bahr El Ghazal, West Equatoria

Bioclime: Savannah

	1990			2020			2030		
	N	%	DALYs	N	%	DALYs	N	%	DALYs
Total populaton at risk	3,849	100	_	8,686	100	_	11,393	100	_
**Infection:**									
Mf-positive	1,122	29.1	_	979	11.3	_	323	2.8	_
Mf-positive (age 5+)	1,120	29.1	_	978	11.3	_	323	2.8	_
Worm infected	1,478	38.4	_	1,897	21.8	_	943	8.3	_
**Clinical manifestations:**									
Palpable nodules	270	7.0	3	212	2.4	2	81	0.7	1
Reversible skin disease:									
Severe itch	154	4.0	28	88	1.0	16	22	0.2	4
Reactive skin disease	109	2.8	5	40	0.5	2	6	0.1	<0.5
**Reversible skin disease**	**262**	**6.8**	**33**	**128**	**1.5**	**18**	**28**	**0.2**	**4**
Irreversible skin disease:									
Mild depigmentation	37	1.0	<0.5	52	0.6	1	41	0.4	<0.5
Severe depigmentation	35	0.9	2	46	0.5	3	33	0.3	2
Atrophy	<0.5	<0.05	<0.5	<0.5	<0.05	<0.5	<0.5	<0.05	<0.5
Hanging groin	1	<0.05	<0.5	1	<0.05	<0.5	<0.5	<0.05	<0.5
**Irreversible skin disease**	**73**	**1.9**	**3**	**98**	**1.1**	**4**	**74**	**0.7**	**3**
**Total skin disease (OSD)**	**605**	**15.7**	**39**	**438**	**5.0**	**24**	**184**	**1.6**	**8**
Eye disease (OED):									
Visual impairment	66	1.7	2	90	1.0	3	68	0.6	2
Blindness	26	0.7	200	27	0.3	208	15	0.1	116
**Eye disease (OED)**	**93**	**2.4**	**202**	**117**	**1.3**	**211**	**83**	**0.7**	**118**
**Total all manifestations**	**698**	**18.1**	**241**	**555**	**6.4**	**235**	**267**	**2.3**	**126**

Note 1: Population at risk, number of cases (N) and DALYs are presented in thousands.

Note 2: Total skin disease is defined as the sum of the number of cases with palpable nodules, reversible and irreversible skin disease.

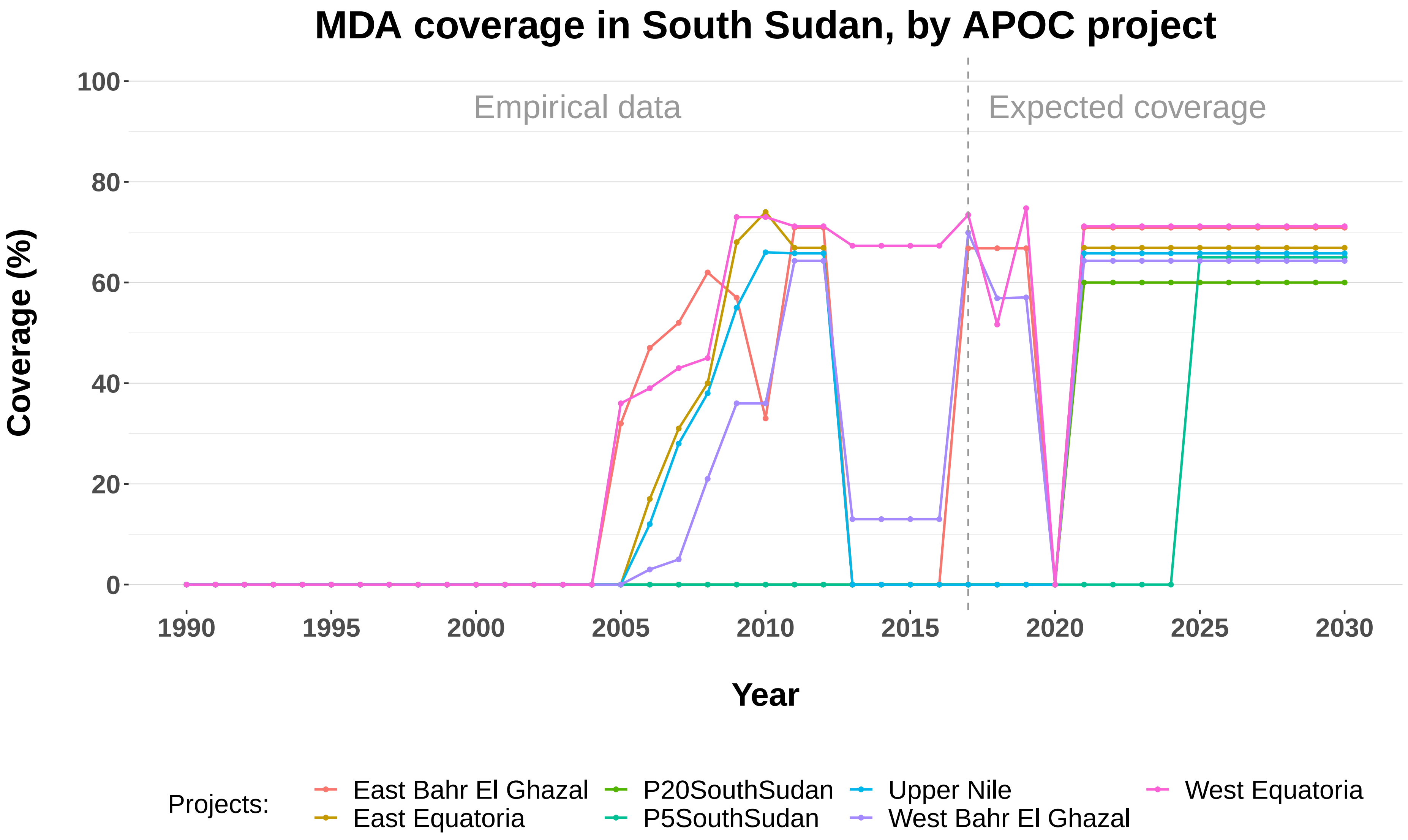

MDA coverage in South Sudan, by APOC project
Empirical data
Expected coverage
Coverage (%)
100
80
60
40
20
0
1990
1995
2000
2005
2010
2015
2020
2025
2030
Year
Projects:
East Bahr El Ghazal
East Equatoria
P20SouthSudan
P5SouthSudan
Upper Nile
West Bahr El Ghazal
West Equatoria

**Country:**	**Sudan**
Projects:	P5Sudan, Sudan, Sudan Abu Hamed
Bioclime:	Savannah

	1990			2020			2030		
	N	%	DALYs	N	%	DALYs	N	%	DALYs
Total populaton at risk	366	100	_	826	100	_	1,083	100	_
**Infection:**									
Mf-positive	79	21.6	_	64	7.8	_	48	4.5	_
Mf-positive (age 5+)	79	21.6	_	64	7.8	_	48	4.5	_
Worm infected	107	29.1	_	117	14.2	_	89	8.2	_
**Clinical manifestations:**									
Palpable nodules	9	2.4	<0.5	8	1.0	<0.5	7	0.6	<0.5
Reversible skin disease:									
Severe itch	10	2.6	2	8	1.0	1	4	0.4	1
Reactive skin disease	6	1.7	<0.5	5	0.6	<0.5	1	0.1	<0.5
**Reversible skin disease**	**16**	**4.3**	**2**	**13**	**1.6**	**2**	**5**	**0.5**	**1**
Irreversible skin disease:									
Mild depigmentation	3	0.7	<0.5	4	0.5	<0.5	3	0.3	<0.5
Severe depigmentation	2	0.6	<0.5	3	0.4	<0.5	2	0.2	<0.5
Atrophy	<0.5	<0.05	<0.5	<0.5	<0.05	<0.5	<0.5	<0.05	<0.5
Hanging groin	<0.5	<0.05	<0.5	<0.5	<0.05	<0.5	<0.5	<0.05	<0.5
**Irreversible skin disease**	**5**	**1.3**	**<0.5**	**7**	**0.8**	**<0.5**	**6**	**0.5**	**<0.5**
**Total skin disease (OSD)**	**29**	**8.0**	**2**	**28**	**3.4**	**2**	**18**	**1.6**	**1**
Eye disease (OED):									
Visual impairment	4	1.2	<0.5	6	0.8	<0.5	5	0.5	<0.5
Blindness	1	0.4	9	2	0.2	11	1	0.1	8
**Eye disease (OED)**	**6**	**1.6**	**9**	**8**	**1.0**	**11**	**7**	**0.6**	**8**
**Total all manifestations**	**35**	**9.6**	**12**	**36**	**4.4**	**13**	**24**	**2.2**	**9**

Note 1: Population at risk, number of cases (N) and DALYs are presented in thousands.

Note 2: Total skin disease is defined as the sum of the number of cases with palpable nodules, reversible and irreversible skin disease.

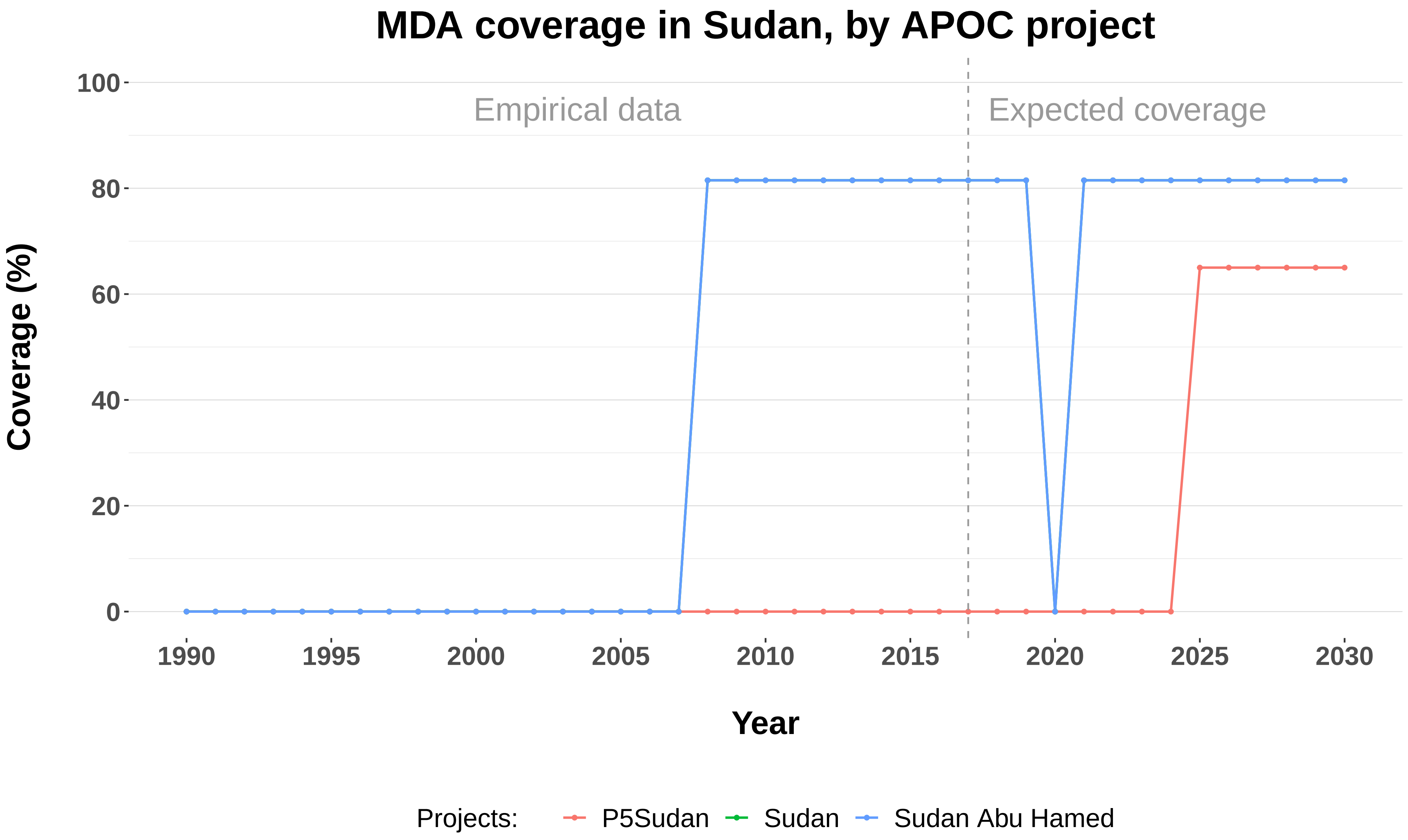
MDA coverage in Sudan, by APOC project
Empirical data
Expected coverage
Coverage (%)
100
80
60
40
20
0
1990
1995
2000
2005
2010
2015
2020
2025
2030
Year
Projects:
P5Sudan
Sudan
Sudan Abu Hamed

**Country:**	**Tanzania**
Projects:	Kilosa, Mahenge, Morogoro, P5Tanzania, Ruvuma, Tanga, Tukuyu, Tunduru
Bioclime:	Forest

	1990			2020			2030		
	N	%	DALYs	N	%	DALYs	N	%	DALYs
Total populaton at risk	1,839	100	_	4,151	100	_	5,444	100	_
**Infection:**									
Mf-positive	631	34.3	_	338	8.1	_	186	3.4	_
Mf-positive (age 5+)	630	34.3	_	338	8.1	_	186	3.4	_
Worm infected	787	42.8	_	594	14.3	_	403	7.4	_
**Clinical manifestations:**									
Palpable nodules	189	10.3	2	68	1.6	1	35	0.6	<0.5
Reversible skin disease:									
Severe itch	103	5.6	19	42	1.0	8	16	0.3	3
Reactive skin disease	77	4.2	4	27	0.6	1	3	0.1	<0.5
**Reversible skin disease**	**180**	**9.8**	**22**	**69**	**1.7**	**9**	**19**	**0.3**	**3**
Irreversible skin disease:									
Mild depigmentation	23	1.3	<0.5	27	0.6	<0.5	21	0.4	<0.5
Severe depigmentation	27	1.4	2	27	0.7	2	20	0.4	1
Atrophy	<0.5	<0.05	<0.5	<0.5	<0.05	<0.5	<0.5	<0.05	<0.5
Hanging groin	1	<0.05	<0.5	<0.5	<0.05	<0.5	<0.5	<0.05	<0.5
**Irreversible skin disease**	**51**	**2.8**	**2**	**55**	**1.3**	**2**	**42**	**0.8**	**2**
**Total skin disease (OSD)**	**420**	**22.8**	**26**	**192**	**4.6**	**12**	**96**	**1.8**	**5**
Eye disease (OED):									
Visual impairment	1	0.1	<0.5	1	<0.05	<0.5	1	<0.05	<0.5
Blindness	2	0.1	11	1	<0.05	4	<0.5	<0.05	2
**Eye disease (OED)**	**3**	**0.2**	**11**	**2**	**<0.05**	**4**	**1**	**<0.05**	**2**
**Total all manifestations**	**423**	**23.0**	**38**	**194**	**4.7**	**16**	**97**	**1.8**	**7**

Note 1: Population at risk, number of cases (N) and DALYs are presented in thousands.

Note 2: Total skin disease is defined as the sum of the number of cases with palpable nodules, reversible and irreversible skin disease.

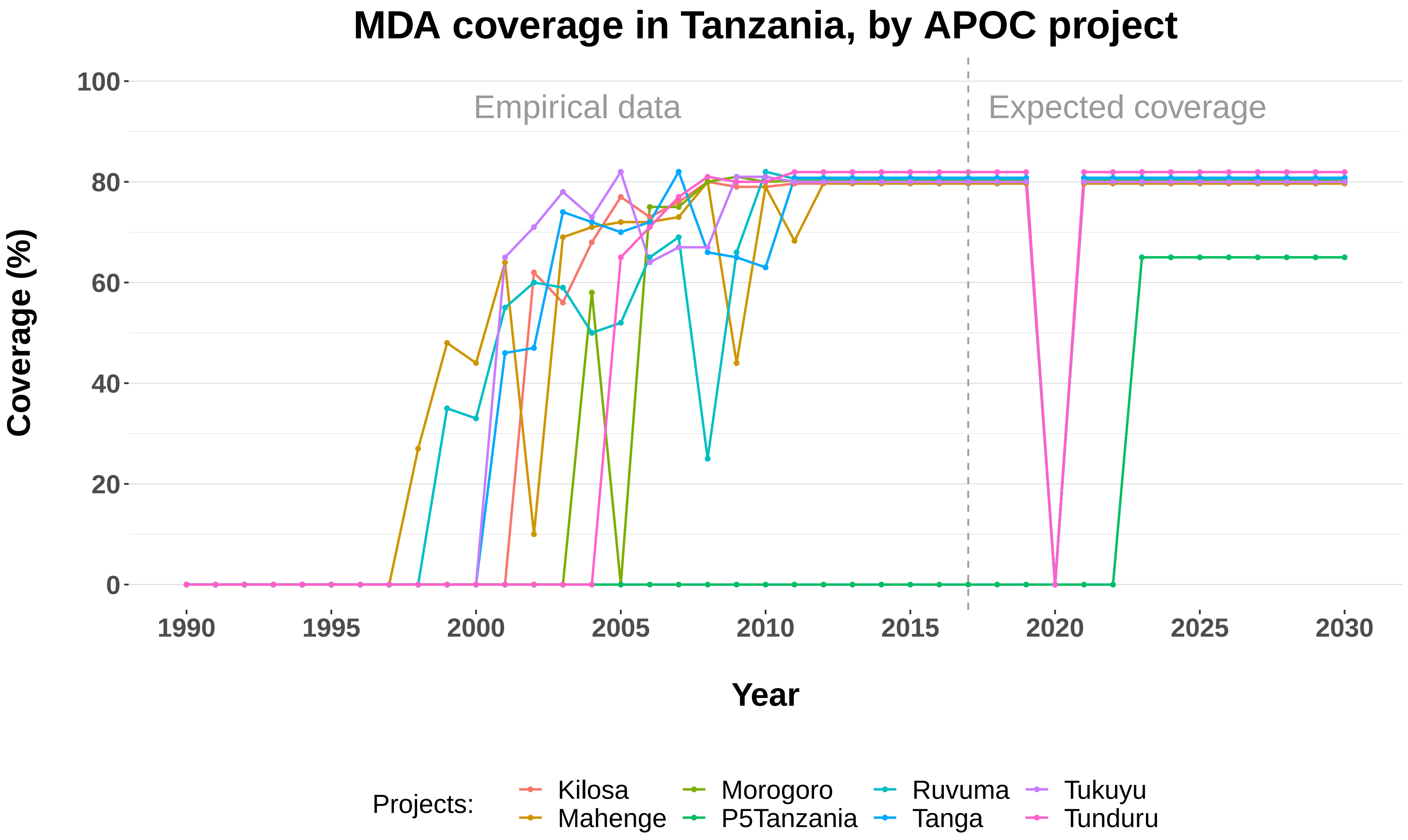

MDA coverage in Tanzania, by APOC project
Empirical data
Expected coverage
Coverage (%)
100
80
60
40
20
0
1990
1995
2000
2005
2010
2015
2020
2025
2030
Year
Projects:
Kilosa
Mahenge
Morogoro
P5Tanzania
Ruvuma
Tanga
Tukuyu
Tunduru

Country: **Uganda**

Projects: P5Uganda, Phase 1, Phase 2, Phase 3, Phase 4, Phase 5
Bioclime: Forest

	1990			2020			2030		
	N	%	DALYs	N	%	DALYs	N	%	DALYs
Total populaton at risk	2,155	100	_	4,863	100	_	6,379	100	_
**Infection:**									
Mf-positive	708	32.9	_	7	0.2	_	<0.5	<0.05	_
Mf-positive (age 5+)	707	32.8	_	7	0.2	_	<0.5	<0.05	_
Worm infected	881	40.9	_	150	3.1	_	4	0.1	_
**Clinical manifestations:**									
Palpable nodules	209	9.7	2	10	0.2	<0.5	<0.5	<0.05	<0.5
Reversible skin disease:									
Severe itch	117	5.5	21	3	0.1	<0.5	<0.5	<0.05	<0.5
Reactive skin disease	90	4.2	4	<0.5	<0.05	<0.5	<0.5	<0.05	<0.5
**Reversible skin disease**	**207**	**9.6**	**25**	**3**	**0.1**	**<0.5**	**<0.5**	**<0.05**	**<0.5**
Irreversible skin disease:									
Mild depigmentation	27	1.2	<0.5	20	0.4	<0.5	13	0.2	<0.5
Severe depigmentation	31	1.5	2	21	0.4	1	13	0.2	1
Atrophy	1	<0.05	<0.5	<0.5	<0.05	<0.5	<0.5	<0.05	<0.5
Hanging groin	1	0.1	<0.5	1	<0.05	<0.5	<0.5	<0.05	<0.5
**Irreversible skin disease**	**60**	**2.8**	**3**	**42**	**0.9**	**2**	**27**	**0.4**	**1**
**Total skin disease (OSD)**	**476**	**22.1**	**30**	**54**	**1.1**	**2**	**27**	**0.4**	**1**
Eye disease (OED):									
Visual impairment	2	0.1	<0.5	1	<0.05	<0.5	1	<0.05	<0.5
Blindness	2	0.1	15	1	<0.05	4	<0.5	<0.05	1
**Eye disease (OED)**	**4**	**0.2**	**15**	**2**	**<0.05**	**4**	**1**	**<0.05**	**1**
**Total all manifestations**	**480**	**22.3**	**45**	**56**	**1.1**	**6**	**27**	**0.4**	**2**

Note 1: Population at risk, number of cases (N) and DALYs are presented in thousands.

Note 2: Total skin disease is defined as the sum of the number of cases with palpable nodules, reversible and irreversible skin disease.

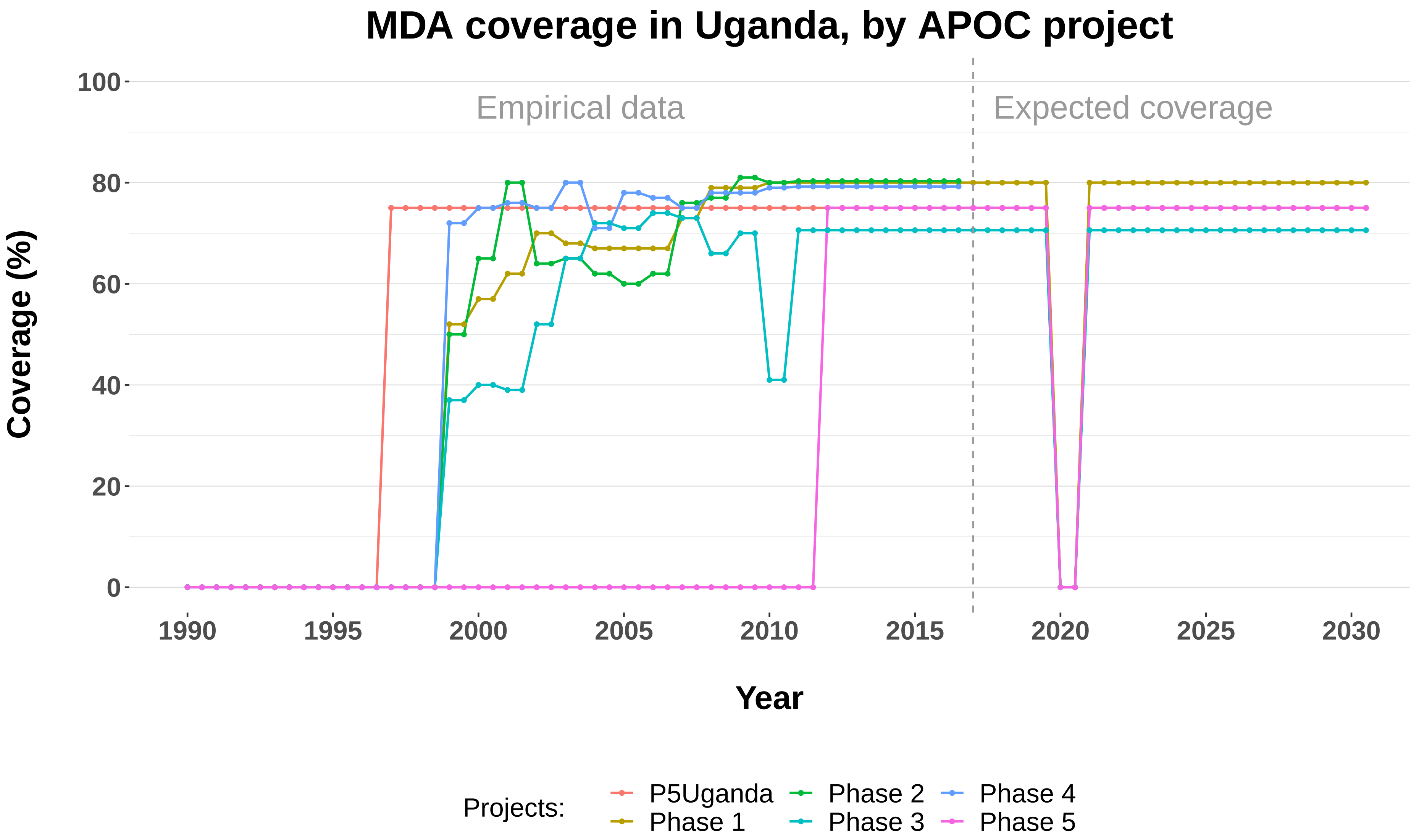

Note: We assume no ivermectin MDA for Phase 2 and Phase 4 as the foci in these APOC-projects are currently under post-treatment surveillance (PTS), or completed the PTS successfully [5-10][‡, page 354]. For the other APOC-projects in Uganda, we assume that most MDA interventions would continue until at least 2030 (even though some foci are also considered for PTS).

**Project, country:**	**Bengo, Angola**
Bioclime:	
Endemicity level(s):	Mesoendemic
MDA start year:	2010
Mean MDA coverage per year:	70.8

	1990			2020			2030		
	N	%	DALYs	N	%	DALYs	N	%	DALYs
Total populaton at risk	13	100	_	30	100	_	39	100	_
**Infection:**									
Mf-positive	3	23.9	_	1	3.4	_	<0.5	<0.05	_
Mf-positive (age 5+)	3	23.8	_	1	3.4	_	<0.5	<0.05	_
Worm infected	4	32.0	_	4	13.9	_	<0.5	1.0	_
**Clinical manifestations:**									
Palpable nodules	<0.5	3.5	<0.5	<0.5	0.6	<0.5	<0.5	<0.05	<0.5
Reversible skin disease:									
Severe itch	<0.5	2.9	<0.5	<0.5	0.2	<0.5	<0.5	<0.05	<0.5
Reactive skin disease	<0.5	1.9	<0.5	<0.5	<0.05	<0.5	<0.5	<0.05	<0.5
**Reversible skin disease**	**1**	**4.9**	**<0.5**	**<0.5**	**0.3**	**<0.5**	**<0.5**	**<0.05**	**<0.5**
Irreversible skin disease:									
Mild depigmentation	<0.5	0.8	<0.5	<0.5	0.5	<0.5	<0.5	0.3	<0.5
Severe depigmentation	<0.5	0.7	<0.5	<0.5	0.4	<0.5	<0.5	0.2	<0.5
Atrophy	<0.5	<0.05	<0.5	<0.5	<0.05	<0.5	<0.5	<0.05	<0.5
Hanging groin	<0.5	<0.05	<0.5	<0.5	<0.05	<0.5	<0.5	<0.05	<0.5
**Irreversible skin disease**	**<0.5**	**1.4**	**<0.5**	**<0.5**	**0.9**	**<0.5**	**<0.5**	**0.5**	**<0.5**
**Total skin disease (OSD)**	**1**	**9.8**	**<0.5**	**1**	**1.8**	**<0.5**	**<0.5**	**0.5**	**<0.5**
Eye disease (OED):									
Visual impairment	<0.5	<0.05	<0.5	<0.5	<0.05	<0.5	<0.5	<0.05	<0.5
Blindness	<0.5	<0.05	<0.5	<0.5	<0.05	<0.5	<0.5	<0.05	<0.5
**Eye disease (OED)**	**<0.5**	**<0.05**	**<0.5**	**<0.5**	**<0.05**	**<0.5**	**<0.5**	**<0.05**	**<0.5**
**Total all manifestations**	**1**	**9.8**	**<0.5**	**1**	**1.8**	**<0.5**	**<0.5**	**0.5**	**<0.5**

Note 1: Population at risk, number of cases (N) and DALYs are presented in thousands.

Note 2: Total skin disease is defined as the sum of the number of cases with palpable nodules, reversible and irreversible skin disease.

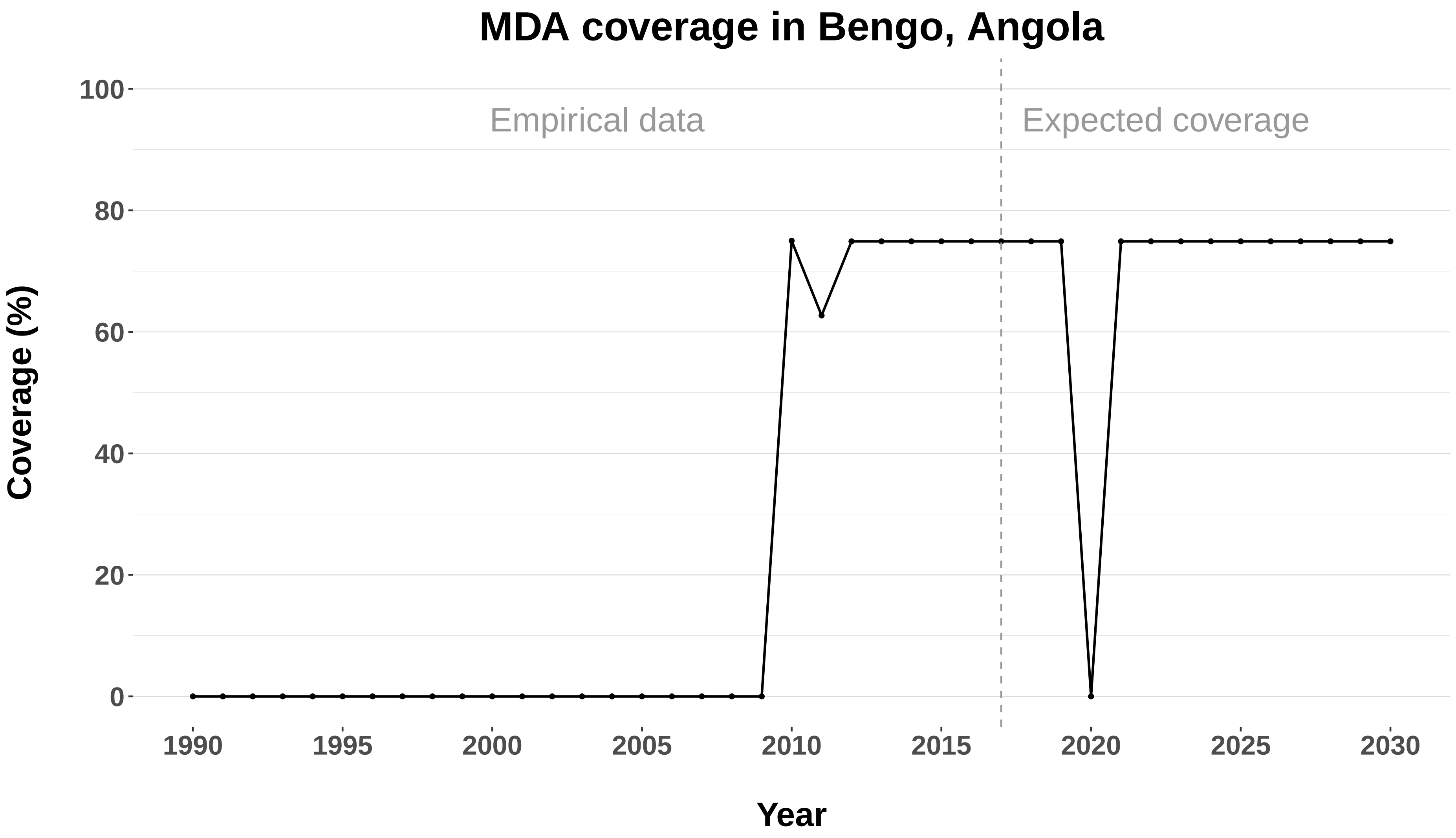
MDA coverage in Bengo, Angola
Empirical data
Expected coverage
Coverage (%)
100
80
60
40
20
0
1990
1995
2000
2005
2010
2015
2020
2025
2030
Year

**Project, country:**	**Benguela, Angola**
Bioclime:	
Endemicity level(s):	Hypoendemic
MDA start year:	2012
Mean MDA coverage per year:	59.4

	1990			2020			2030		
	N	%	DALYs	N	%	DALYs	N	%	DALYs
Total populaton at risk	25	100	_	57	100	_	75	100	_
**Infection:**									
Mf-positive	6	23.6	_	4	7.6	_	<0.5	0.3	_
Mf-positive (age 5+)	6	23.6	_	4	7.6	_	<0.5	0.3	_
Worm infected	8	32.2	_	11	19.2	_	2	3.3	_
**Clinical manifestations:**									
Palpable nodules	1	3.6	<0.5	1	1.3	<0.5	<0.5	0.1	<0.5
Reversible skin disease:									
Severe itch	1	2.8	<0.5	<0.5	0.6	<0.5	<0.5	<0.05	<0.5
Reactive skin disease	<0.5	1.9	<0.5	<0.5	0.1	<0.5	<0.5	<0.05	<0.5
**Reversible skin disease**	**1**	**4.8**	**<0.5**	**<0.5**	**0.7**	**<0.5**	**<0.5**	**<0.05**	**<0.5**
Irreversible skin disease:									
Mild depigmentation	<0.5	0.8	<0.5	<0.5	0.5	<0.5	<0.5	0.3	<0.5
Severe depigmentation	<0.5	0.6	<0.5	<0.5	0.4	<0.5	<0.5	0.2	<0.5
Atrophy	<0.5	<0.05	<0.5	<0.5	<0.05	<0.5	<0.5	<0.05	<0.5
Hanging groin	<0.5	<0.05	<0.5	<0.5	<0.05	<0.5	<0.5	<0.05	<0.5
**Irreversible skin disease**	**<0.5**	**1.4**	**<0.5**	**1**	**0.9**	**<0.5**	**<0.5**	**0.5**	**<0.5**
**Total skin disease (OSD)**	**2**	**9.7**	**<0.5**	**2**	**2.9**	**<0.5**	**<0.5**	**0.6**	**<0.5**
Eye disease (OED):									
Visual impairment	<0.5	<0.05	<0.5	<0.5	<0.05	<0.5	<0.5	<0.05	<0.5
Blindness	<0.5	<0.05	<0.5	<0.5	<0.05	<0.5	<0.5	<0.05	<0.5
**Eye disease (OED)**	**<0.5**	**<0.05**	**<0.5**	**<0.5**	**<0.05**	**<0.5**	**<0.5**	**<0.05**	**<0.5**
**Total all manifestations**	**2**	**9.8**	**<0.5**	**2**	**2.9**	**<0.5**	**<0.5**	**0.6**	**<0.5**

Note 1: Population at risk, number of cases (N) and DALYs are presented in thousands.

Note 2: Total skin disease is defined as the sum of the number of cases with palpable nodules, reversible and irreversible skin disease.

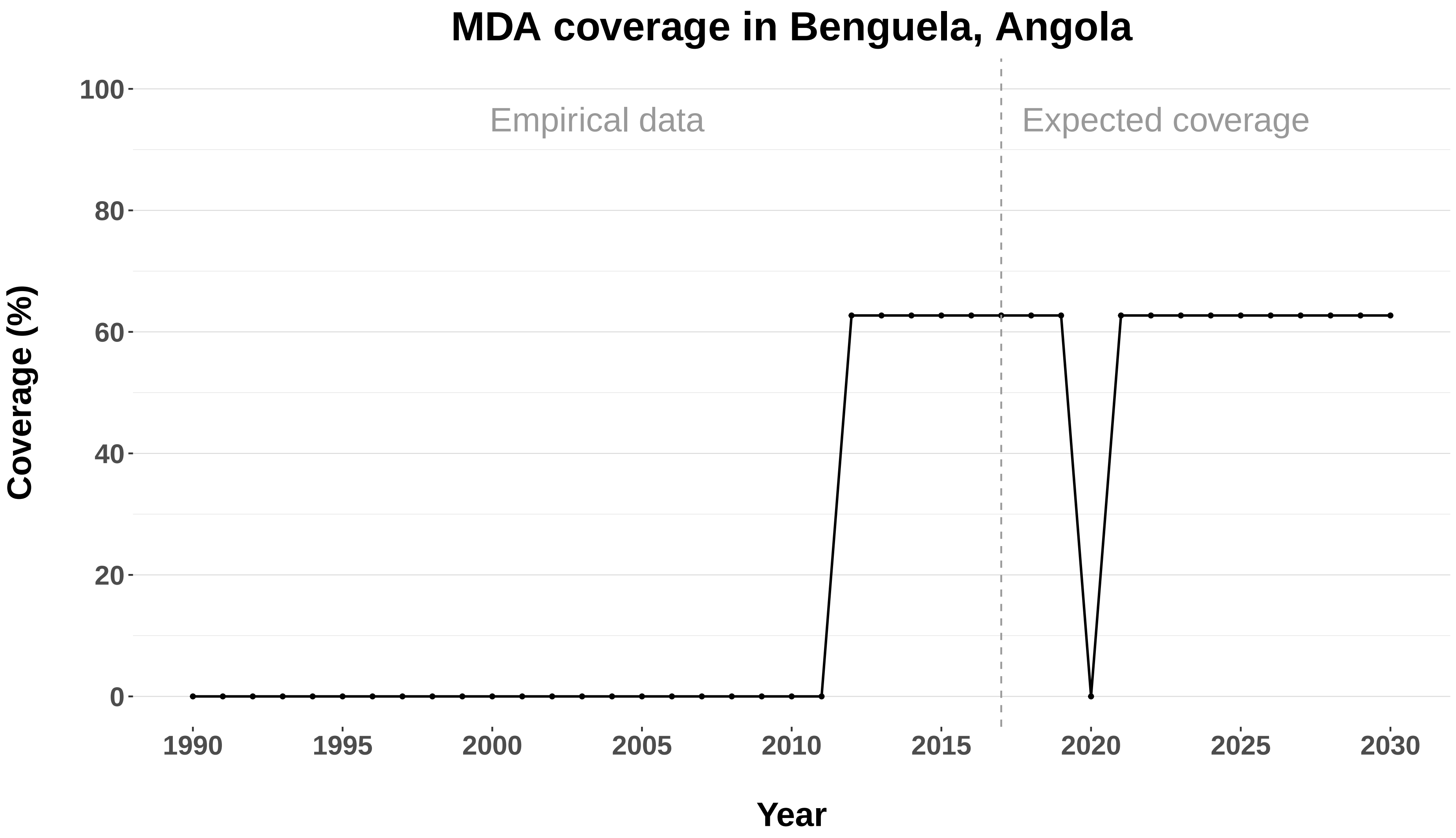
MDA coverage in Benguela, Angola
Empirical data
Expected coverage
Coverage (%)
100
80
60
40
20
0
1990
1995
2000
2005
2010
2015
2020
2025
2030
Year

**Project, country:**	**Cuanza Norte, Angola**
Bioclime:	
Endemicity level(s):	Mesoendemic
MDA start year:	2011
Mean MDA coverage per year:	71.2

	1990			2020			2030		
	N	%	DALYs	N	%	DALYs	N	%	DALYs
Total populaton at risk	13	100	_	29	100	_	38	100	_
**Infection:**									
Mf-positive	3	22.6	_	1	4.0	_	<0.5	<0.05	_
Mf-positive (age 5+)	3	22.6	_	1	4.0	_	<0.5	<0.05	_
Worm infected	4	31.0	_	4	14.6	_	<0.5	1.2	_
**Clinical manifestations:**									
Palpable nodules	<0.5	3.1	<0.5	<0.5	0.7	<0.5	<0.5	<0.05	<0.5
Reversible skin disease:									
Severe itch	<0.5	2.7	<0.5	<0.5	0.3	<0.5	<0.5	<0.05	<0.5
Reactive skin disease	<0.5	1.8	<0.5	<0.5	<0.05	<0.5	<0.5	<0.05	<0.5
**Reversible skin disease**	**1**	**4.5**	**<0.5**	**<0.5**	**0.3**	**<0.5**	**<0.5**	**<0.05**	**<0.5**
Irreversible skin disease:									
Mild depigmentation	<0.5	0.7	<0.5	<0.5	0.5	<0.5	<0.5	0.2	<0.5
Severe depigmentation	<0.5	0.6	<0.5	<0.5	0.3	<0.5	<0.5	0.2	<0.5
Atrophy	<0.5	<0.05	<0.5	<0.5	<0.05	<0.5	<0.5	<0.05	<0.5
Hanging groin	<0.5	<0.05	<0.5	<0.5	<0.05	<0.5	<0.5	<0.05	<0.5
**Irreversible skin disease**	**<0.5**	**1.3**	**<0.5**	**<0.5**	**0.8**	**<0.5**	**<0.5**	**0.4**	**<0.5**
**Total skin disease (OSD)**	**1**	**8.9**	**<0.5**	**1**	**1.8**	**<0.5**	**<0.5**	**0.4**	**<0.5**
Eye disease (OED):									
Visual impairment	<0.5	<0.05	<0.5	<0.5	<0.05	<0.5	<0.5	<0.05	<0.5
Blindness	<0.5	<0.05	<0.5	<0.5	<0.05	<0.5	<0.5	<0.05	<0.5
**Eye disease (OED)**	**<0.5**	**<0.05**	**<0.5**	**<0.5**	**<0.05**	**<0.5**	**<0.5**	**<0.05**	**<0.5**
**Total all manifestations**	**1**	**8.9**	**<0.5**	**1**	**1.8**	**<0.5**	**<0.5**	**0.4**	**<0.5**

Note 1: Population at risk, number of cases (N) and DALYs are presented in thousands.

Note 2: Total skin disease is defined as the sum of the number of cases with palpable nodules, reversible and irreversible skin disease.

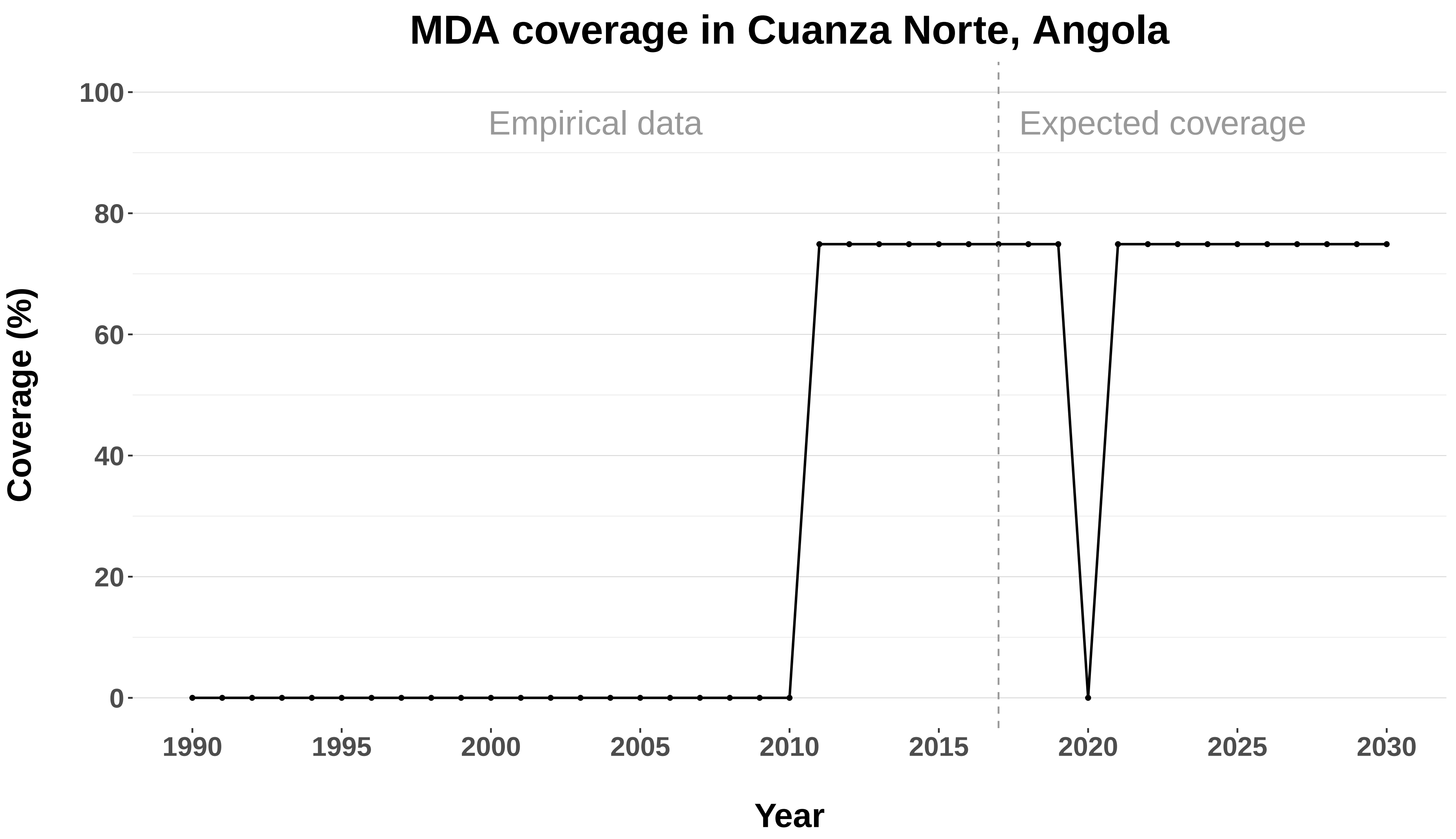
MDA coverage in Cuanza Norte, Angola
Empirical data
Expected coverage
Coverage (%)
0
20
40
60
80
100
1990
1995
2000
2005
2010
2015
2020
2025
2030
Year

**Project, country:**	**Huila, Angola**
Bioclime:	
Endemicity level(s):	Mesoendemic
MDA start year:	2007
Mean MDA coverage per year:	59.2

	1990			2020			2030		
	N	%	DALYs	N	%	DALYs	N	%	DALYs
Total populaton at risk	119	100	_	268	100	_	352	100	_
**Infection:**									
Mf-positive	39	33.2	_	19	7.1	_	1	0.3	_
Mf-positive (age 5+)	39	33.2	_	19	7.1	_	1	0.3	_
Worm infected	50	42.0	_	56	20.9	_	10	2.8	_
**Clinical manifestations:**									
Palpable nodules	11	8.8	<0.5	6	2.1	<0.5	<0.5	0.1	<0.5
Reversible skin disease:									
Severe itch	6	4.9	1	2	0.6	<0.5	<0.5	<0.05	<0.5
Reactive skin disease	4	3.5	<0.5	<0.5	0.1	<0.5	<0.5	<0.05	<0.5
**Reversible skin disease**	**10**	**8.4**	**1**	**2**	**0.7**	**<0.5**	**<0.5**	**<0.05**	**<0.5**
Irreversible skin disease:									
Mild depigmentation	1	1.2	<0.5	2	0.7	<0.5	1	0.4	<0.5
Severe depigmentation	1	1.2	<0.5	2	0.7	<0.5	1	0.4	<0.5
Atrophy	<0.5	<0.05	<0.5	<0.5	<0.05	<0.5	<0.5	<0.05	<0.5
Hanging groin	<0.5	<0.05	<0.5	<0.5	<0.05	<0.5	<0.5	<0.05	<0.5
**Irreversible skin disease**	**3**	**2.4**	**<0.5**	**4**	**1.4**	**<0.5**	**3**	**0.8**	**<0.5**
**Total skin disease (OSD)**	**23**	**19.7**	**1**	**11**	**4.1**	**<0.5**	**3**	**0.9**	**<0.5**
Eye disease (OED):									
Visual impairment	<0.5	<0.05	<0.5	<0.5	<0.05	<0.5	<0.5	<0.05	<0.5
Blindness	<0.5	<0.05	<0.5	<0.5	<0.05	<0.5	<0.5	<0.05	<0.5
**Eye disease (OED)**	**<0.5**	**0.1**	**<0.5**	**<0.5**	**<0.05**	**<0.5**	**<0.5**	**<0.05**	**<0.5**
**Total all manifestations**	**23**	**19.7**	**2**	**11**	**4.2**	**1**	**3**	**0.9**	**<0.5**

Note 1: Population at risk, number of cases (N) and DALYs are presented in thousands.

Note 2: Total skin disease is defined as the sum of the number of cases with palpable nodules, reversible and irreversible skin disease.

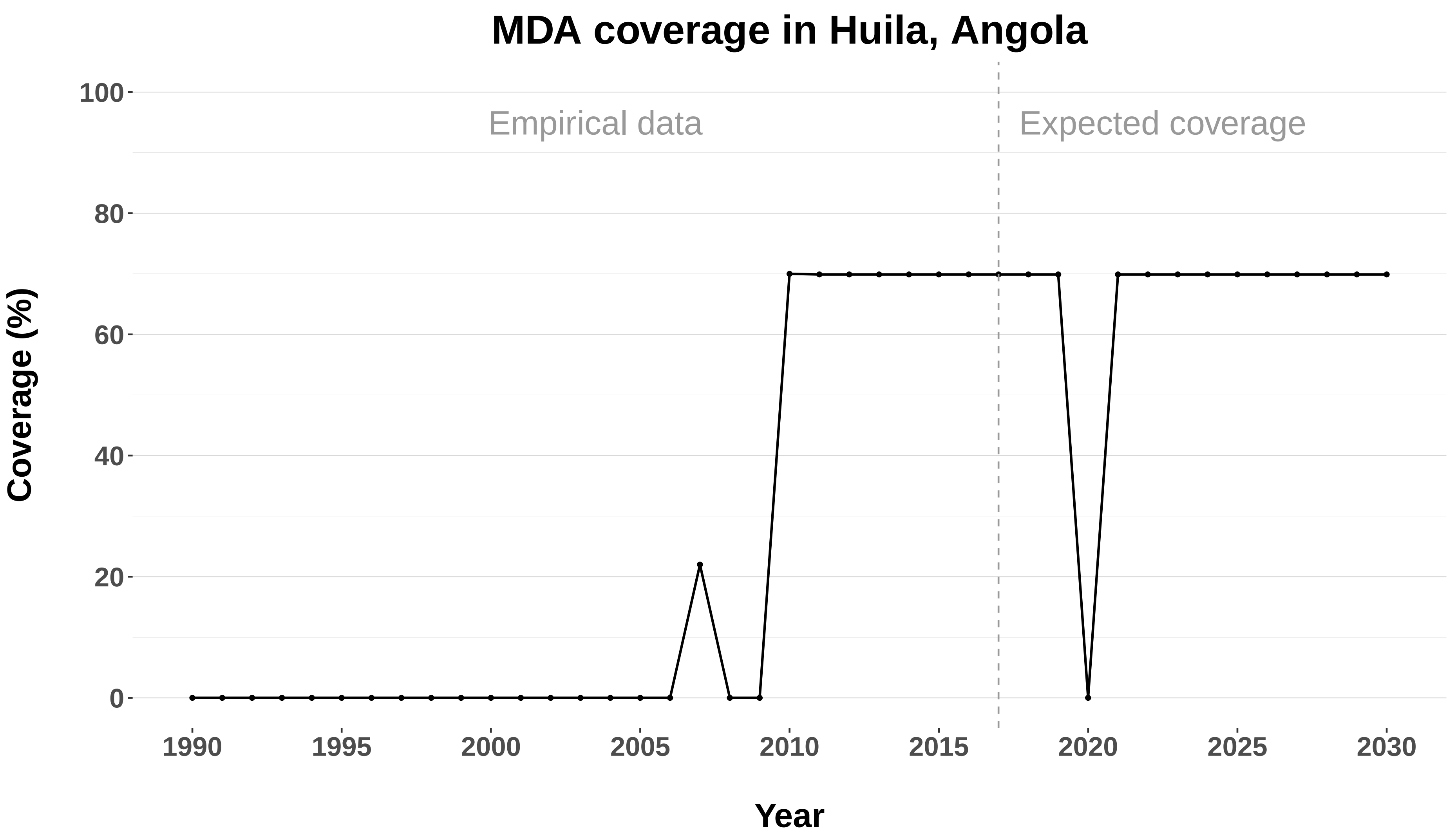
MDA coverage in Huila, Angola
Empirical data
Expected coverage
Coverage (%)
100
80
60
40
20
0
1990
1995
2000
2005
2010
2015
2020
2025
2030
Year

**Project, country:**	**Kuando Kubango, Angola**
Bioclime:	
Endemicity level(s):	Mesoendemic
MDA start year:	2007
Mean MDA coverage per year:	68.9

	1990			2020			2030		
	N	%	DALYs	N	%	DALYs	N	%	DALYs
Total populaton at risk	205	100	_	463	100	_	607	100	_
**Infection:**									
Mf-positive	47	22.7	_	6	1.3	_	<0.5	<0.05	_
Mf-positive (age 5+)	47	22.7	_	6	1.3	_	<0.5	<0.05	_
Worm infected	63	30.9	_	40	8.6	_	2	0.3	_
**Clinical manifestations:**									
Palpable nodules	6	3.0	<0.5	1	0.2	<0.5	<0.5	<0.05	<0.5
Reversible skin disease:									
Severe itch	6	2.7	1	1	0.1	<0.5	<0.5	<0.05	<0.5
Reactive skin disease	4	1.9	<0.5	<0.5	<0.05	<0.5	<0.5	<0.05	<0.5
**Reversible skin disease**	**9**	**4.6**	**1**	**1**	**0.1**	**<0.5**	**<0.5**	**<0.05**	**<0.5**
Irreversible skin disease:									
Mild depigmentation	1	0.7	<0.5	2	0.4	<0.5	1	0.2	<0.5
Severe depigmentation	1	0.6	<0.5	1	0.3	<0.5	1	0.1	<0.5
Atrophy	<0.5	<0.05	<0.5	<0.5	<0.05	<0.5	<0.5	<0.05	<0.5
Hanging groin	<0.5	<0.05	<0.5	<0.5	<0.05	<0.5	<0.5	<0.05	<0.5
**Irreversible skin disease**	**3**	**1.3**	**<0.5**	**3**	**0.7**	**<0.5**	**2**	**0.3**	**<0.5**
**Total skin disease (OSD)**	**18**	**9.0**	**1**	**5**	**1.0**	**<0.5**	**2**	**0.3**	**<0.5**
Eye disease (OED):									
Visual impairment	<0.5	<0.05	<0.5	<0.5	<0.05	<0.5	<0.5	<0.05	<0.5
Blindness	<0.5	<0.05	<0.5	<0.5	<0.05	<0.5	<0.5	<0.05	<0.5
**Eye disease (OED)**	**<0.5**	**<0.05**	**<0.5**	**<0.5**	**<0.05**	**<0.5**	**<0.5**	**<0.05**	**<0.5**
**Total all manifestations**	**18**	**9.0**	**1**	**5**	**1.0**	**<0.5**	**2**	**0.3**	**<0.5**

Note 1: Population at risk, number of cases (N) and DALYs are presented in thousands.

Note 2: Total skin disease is defined as the sum of the number of cases with palpable nodules, reversible and irreversible skin disease.

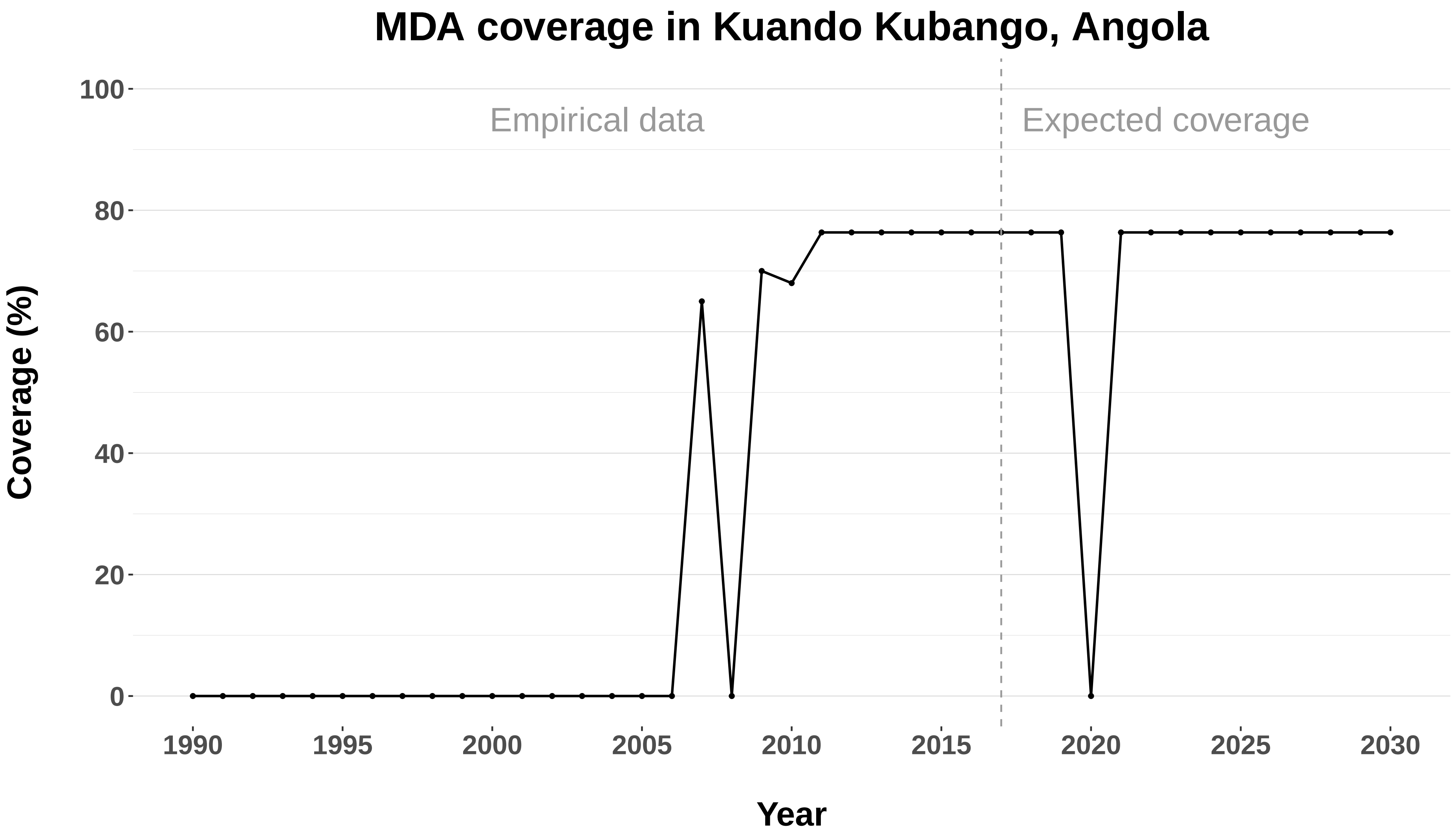

MDA coverage in Kuando Kubango, Angola
Empirical data
Expected coverage
Coverage (%)
100
80
60
40
20
0
1990
1995
2000
2005
2010
2015
2020
2025
2030
Year

**Project, country:**	**Lunda Norte, Angola**
Bioclime:	
Endemicity level(s):	Hyperendemic
MDA start year:	2005
Mean MDA coverage per year:	59.8

	1990			2020			2030		
	N	%	DALYs	N	%	DALYs	N	%	DALYs
Total populaton at risk	153	100	_	346	100	_	453	100	_
**Infection:**									
Mf-positive	42	27.4	_	9	2.6	_	<0.5	<0.05	_
Mf-positive (age 5+)	42	27.4	_	9	2.6	_	<0.5	<0.05	_
Worm infected	56	36.3	_	42	12.3	_	4	0.8	_
**Clinical manifestations:**									
Palpable nodules	8	5.2	<0.5	2	0.5	<0.5	<0.5	<0.05	<0.5
Reversible skin disease:									
Severe itch	5	3.6	1	1	0.2	<0.5	<0.5	<0.05	<0.5
Reactive skin disease	4	2.3	<0.5	<0.5	<0.05	<0.5	<0.5	<0.05	<0.5
**Reversible skin disease**	**9**	**5.9**	**1**	**1**	**0.2**	**<0.5**	**<0.5**	**<0.05**	**<0.5**
Irreversible skin disease:									
Mild depigmentation	1	0.9	<0.5	2	0.5	<0.5	1	0.3	<0.5
Severe depigmentation	1	0.8	<0.5	1	0.4	<0.5	1	0.2	<0.5
Atrophy	<0.5	<0.05	<0.5	<0.5	<0.05	<0.5	<0.5	<0.05	<0.5
Hanging groin	<0.5	<0.05	<0.5	<0.5	<0.05	<0.5	<0.5	<0.05	<0.5
**Irreversible skin disease**	**3**	**1.7**	**<0.5**	**3**	**0.9**	**<0.5**	**2**	**0.5**	**<0.5**
**Total skin disease (OSD)**	**20**	**12.8**	**1**	**6**	**1.7**	**<0.5**	**2**	**0.5**	**<0.5**
Eye disease (OED):									
Visual impairment	<0.5	<0.05	<0.5	<0.5	<0.05	<0.5	<0.5	<0.05	<0.5
Blindness	<0.5	<0.05	<0.5	<0.5	<0.05	<0.5	<0.5	<0.05	<0.5
**Eye disease (OED)**	**<0.5**	**<0.05**	**<0.5**	**<0.5**	**<0.05**	**<0.5**	**<0.5**	**<0.05**	**<0.5**
**Total all manifestations**	**20**	**12.8**	**1**	**6**	**1.7**	**<0.5**	**2**	**0.5**	**<0.5**

Note 1: Population at risk, number of cases (N) and DALYs are presented in thousands.

Note 2: Total skin disease is defined as the sum of the number of cases with palpable nodules, reversible and irreversible skin disease.

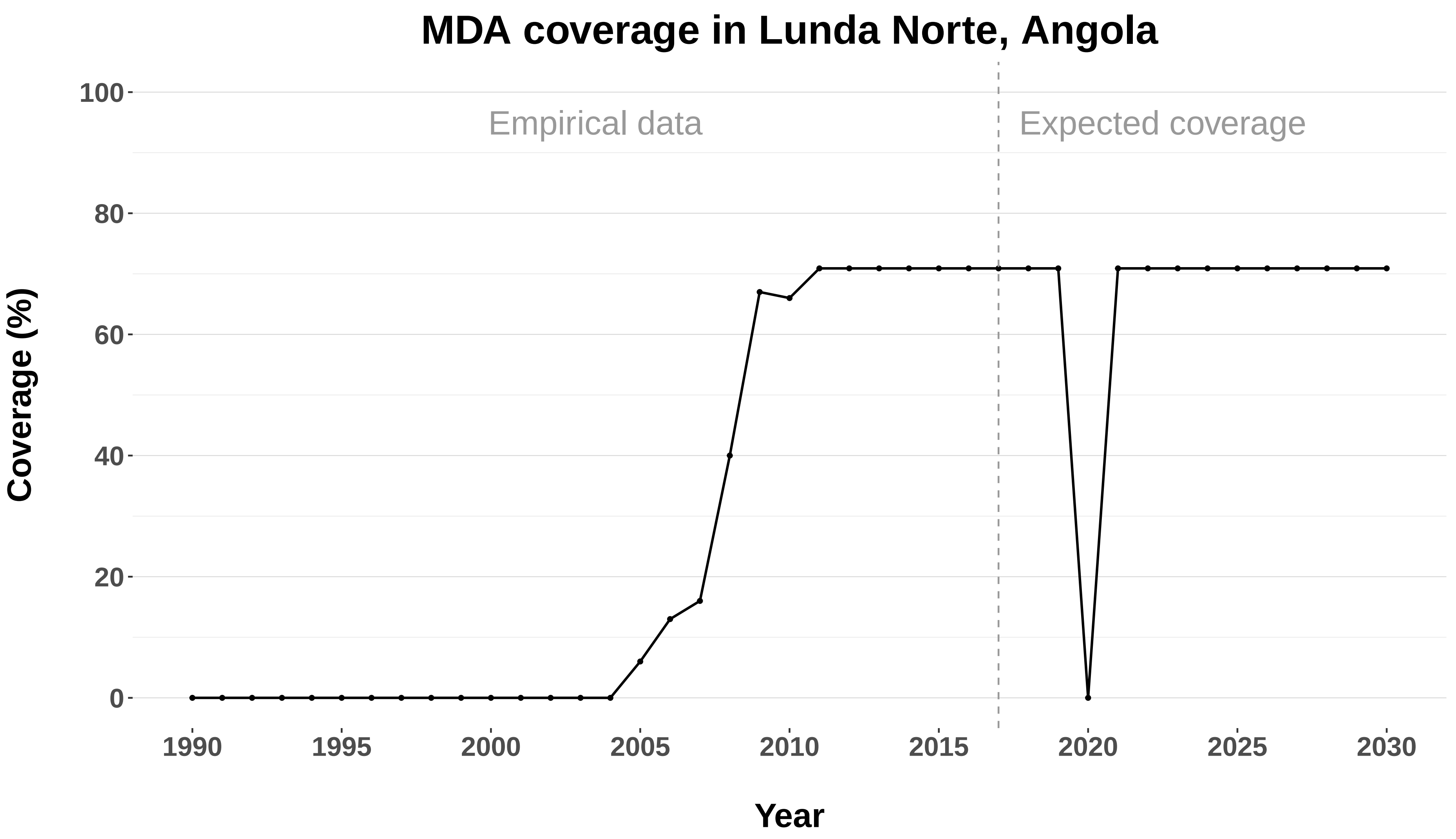
MDA coverage in Lunda Norte, Angola
Empirical data
Expected coverage
Coverage (%)
100
80
60
40
20
0
1990
1995
2000
2005
2010
2015
2020
2025
2030
Year

**Project, country:**	**Lunda sul, Angola**
Bioclime:	
Endemicity level(s):	Mesoendemic
MDA start year:	2005
Mean MDA coverage per year:	60.4

	1990			2020			2030		
	N	%	DALYs	N	%	DALYs	N	%	DALYs
Total populaton at risk	129	100	_	291	100	_	382	100	_
**Infection:**									
Mf-positive	37	28.5	_	8	2.6	_	<0.5	<0.05	_
Mf-positive (age 5+)	37	28.5	_	8	2.6	_	<0.5	<0.05	_
Worm infected	48	36.9	_	35	12.1	_	3	0.9	_
**Clinical manifestations:**									
Palpable nodules	7	5.5	<0.5	1	0.5	<0.5	<0.5	<0.05	<0.5
Reversible skin disease:									
Severe itch	5	3.9	1	1	0.2	<0.5	<0.5	<0.05	<0.5
Reactive skin disease	3	2.6	<0.5	<0.5	<0.05	<0.5	<0.5	<0.05	<0.5
**Reversible skin disease**	**8**	**6.5**	**1**	**1**	**0.2**	**<0.5**	**<0.5**	**<0.05**	**<0.5**
Irreversible skin disease:									
Mild depigmentation	1	1.0	<0.5	2	0.5	<0.5	1	0.3	<0.5
Severe depigmentation	1	0.9	<0.5	1	0.4	<0.5	1	0.2	<0.5
Atrophy	<0.5	<0.05	<0.5	<0.5	<0.05	<0.5	<0.5	<0.05	<0.5
Hanging groin	<0.5	<0.05	<0.5	<0.5	<0.05	<0.5	<0.5	<0.05	<0.5
**Irreversible skin disease**	**2**	**1.8**	**<0.5**	**3**	**1.0**	**<0.5**	**2**	**0.5**	**<0.5**
**Total skin disease (OSD)**	**18**	**13.8**	**1**	**5**	**1.7**	**<0.5**	**2**	**0.5**	**<0.5**
Eye disease (OED):									
Visual impairment	<0.5	<0.05	<0.5	<0.5	<0.05	<0.5	<0.5	<0.05	<0.5
Blindness	<0.5	<0.05	<0.5	<0.5	<0.05	<0.5	<0.5	<0.05	<0.5
**Eye disease (OED)**	**<0.5**	**<0.05**	**<0.5**	**<0.5**	**<0.05**	**<0.5**	**<0.5**	**<0.05**	**<0.5**
**Total all manifestations**	**18**	**13.8**	**1**	**5**	**1.7**	**<0.5**	**2**	**0.5**	**<0.5**

Note 1: Population at risk, number of cases (N) and DALYs are presented in thousands.

Note 2: Total skin disease is defined as the sum of the number of cases with palpable nodules, reversible and irreversible skin disease.

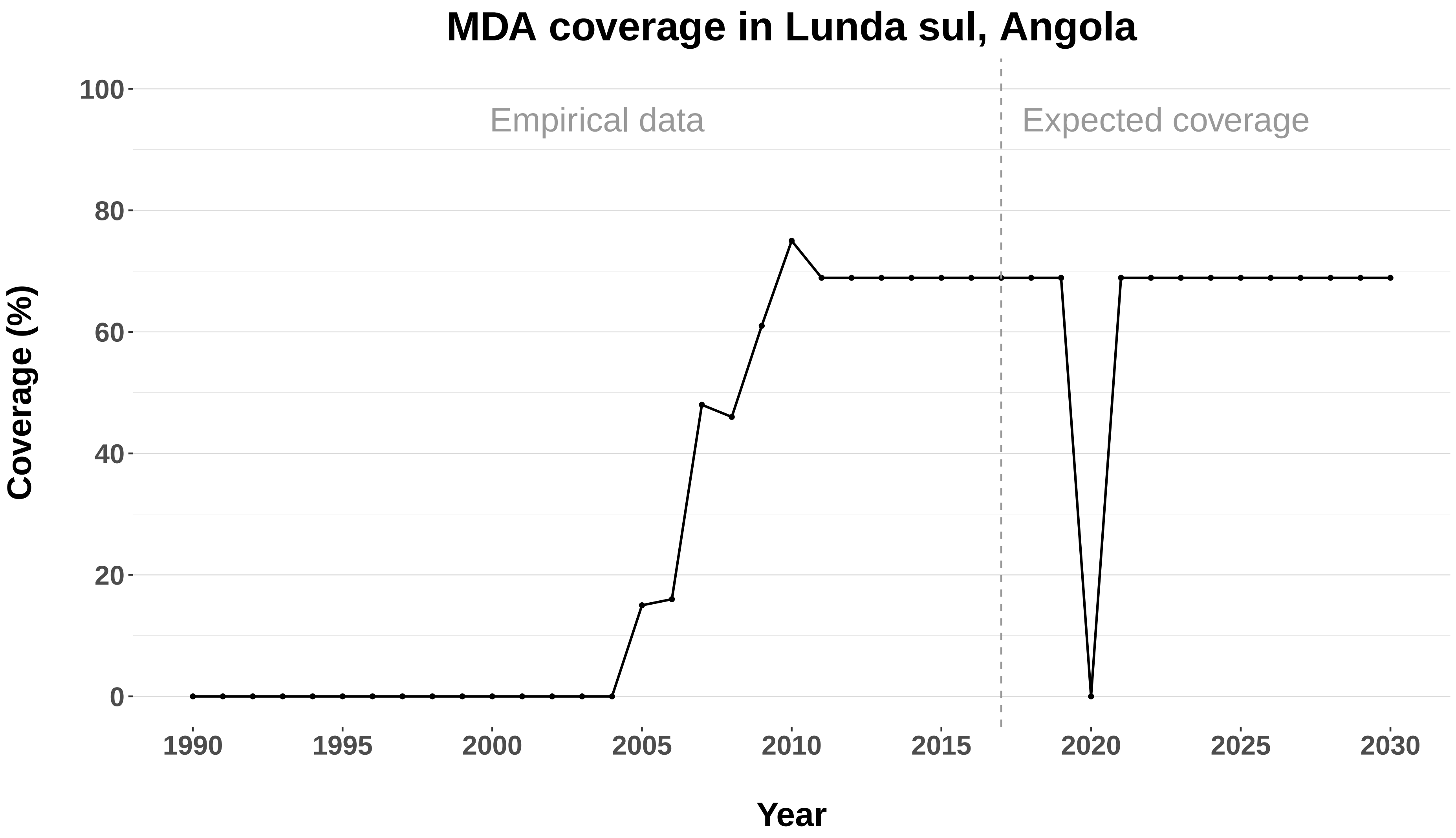
MDA coverage in Lunda sul, Angola
Empirical data
Expected coverage
Coverage (%)
100
80
60
40
20
0
1990
1995
2000
2005
2010
2015
2020
2025
2030
Year

**Project, country:**	**Moxico 1, Angola**
Bioclime:	
Endemicity level(s):	Hyperendemic
MDA start year:	2007
Mean MDA coverage per year:	58.8

	1990			2020			2030		
	N	%	DALYs	N	%	DALYs	N	%	DALYs
Total populaton at risk	135	100	_	304	100	_	399	100	_
**Infection:**									
Mf-positive	31	23.0	_	11	3.7	_	<0.5	<0.05	_
Mf-positive (age 5+)	31	23.0	_	11	3.7	_	<0.5	<0.05	_
Worm infected	42	30.9	_	41	13.5	_	5	1.3	_
**Clinical manifestations:**									
Palpable nodules	4	3.1	<0.5	2	0.5	<0.5	<0.5	<0.05	<0.5
Reversible skin disease:									
Severe itch	4	2.9	1	1	0.3	<0.5	<0.5	<0.05	<0.5
Reactive skin disease	3	1.9	<0.5	<0.5	<0.05	<0.5	<0.5	<0.05	<0.5
**Reversible skin disease**	**6**	**4.7**	**1**	**1**	**0.3**	**<0.5**	**<0.5**	**<0.05**	**<0.5**
Irreversible skin disease:									
Mild depigmentation	1	0.8	<0.5	1	0.4	<0.5	1	0.2	<0.5
Severe depigmentation	1	0.7	<0.5	1	0.4	<0.5	1	0.2	<0.5
Atrophy	<0.5	<0.05	<0.5	<0.5	<0.05	<0.5	<0.5	<0.05	<0.5
Hanging groin	<0.5	<0.05	<0.5	<0.5	<0.05	<0.5	<0.5	<0.05	<0.5
**Irreversible skin disease**	**2**	**1.4**	**<0.5**	**2**	**0.8**	**<0.5**	**2**	**0.4**	**<0.5**
**Total skin disease (OSD)**	**12**	**9.3**	**1**	**5**	**1.7**	**<0.5**	**2**	**0.4**	**<0.5**
Eye disease (OED):									
Visual impairment	<0.5	<0.05	<0.5	<0.5	<0.05	<0.5	<0.5	<0.05	<0.5
Blindness	<0.5	<0.05	<0.5	<0.5	<0.05	<0.5	<0.5	<0.05	<0.5
**Eye disease (OED)**	**<0.5**	**<0.05**	**<0.5**	**<0.5**	**<0.05**	**<0.5**	**<0.5**	**<0.05**	**<0.5**
**Total all manifestations**	**13**	**9.3**	**1**	**5**	**1.7**	**<0.5**	**2**	**0.4**	**<0.5**

Note 1: Population at risk, number of cases (N) and DALYs are presented in thousands.

Note 2: Total skin disease is defined as the sum of the number of cases with palpable nodules, reversible and irreversible skin disease.

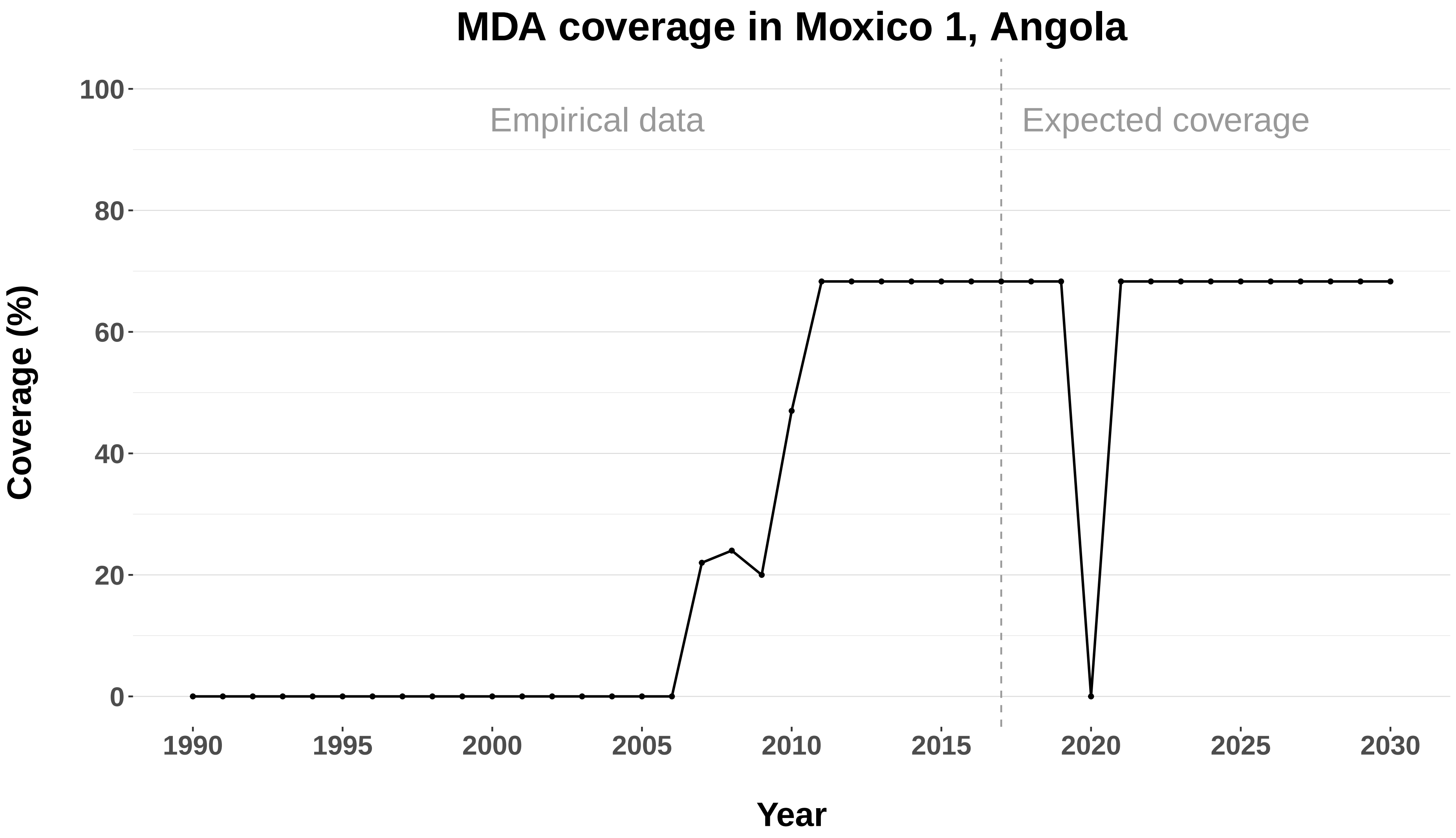
MDA coverage in Moxico 1, Angola
Empirical data
Expected coverage
100
80
60
40
20
0
Coverage (%)
1990
1995
2000
2005
2010
2015
2020
2025
2030
Year

**Project, country:**	**Namibe, Angola**
Bioclime:	
Endemicity level(s):	Mesoendemic
MDA start year:	2019
Mean MDA coverage per year:	61.4

	1990			2020			2030		
	N	%	DALYs	N	%	DALYs	N	%	DALYs
Total populaton at risk	18	100	_	40	100	_	53	100	_
**Infection:**									
Mf-positive	5	26.1	_	10	23.9	_	3	5.3	_
Mf-positive (age 5+)	5	26.1	_	10	23.9	_	3	5.3	_
Worm infected	6	34.2	_	13	33.3	_	9	16.5	_
**Clinical manifestations:**									
Palpable nodules	1	4.3	<0.5	2	4.2	<0.5	<0.5	0.9	<0.5
Reversible skin disease:									
Severe itch	1	3.4	<0.5	1	2.8	<0.5	<0.5	0.4	<0.5
Reactive skin disease	<0.5	2.3	<0.5	1	1.6	<0.5	<0.5	0.1	<0.5
**Reversible skin disease**	**1**	**5.7**	**<0.5**	**2**	**4.5**	**<0.5**	**<0.5**	**0.4**	**<0.5**
Irreversible skin disease:									
Mild depigmentation	<0.5	0.9	<0.5	<0.5	0.8	<0.5	<0.5	0.5	<0.5
Severe depigmentation	<0.5	0.8	<0.5	<0.5	0.7	<0.5	<0.5	0.4	<0.5
Atrophy	<0.5	<0.05	<0.5	<0.5	<0.05	<0.5	<0.5	<0.05	<0.5
Hanging groin	<0.5	<0.05	<0.5	<0.5	<0.05	<0.5	<0.5	<0.05	<0.5
**Irreversible skin disease**	**<0.5**	**1.7**	**<0.5**	**1**	**1.6**	**<0.5**	**1**	**1.0**	**<0.5**
**Total skin disease (OSD)**	**2**	**11.7**	**<0.5**	**4**	**10.2**	**<0.5**	**1**	**2.3**	**<0.5**
Eye disease (OED):									
Visual impairment	<0.5	<0.05	<0.5	<0.5	<0.05	<0.5	<0.5	<0.05	<0.5
Blindness	<0.5	<0.05	<0.5	<0.5	<0.05	<0.5	<0.5	<0.05	<0.5
**Eye disease (OED)**	**<0.5**	**<0.05**	**<0.5**	**<0.5**	**<0.05**	**<0.5**	**<0.5**	**<0.05**	**<0.5**
**Total all manifestations**	**2**	**11.7**	**<0.5**	**4**	**10.2**	**<0.5**	**1**	**2.3**	**<0.5**

Note 1: Population at risk, number of cases (N) and DALYs are presented in thousands.

Note 2: Total skin disease is defined as the sum of the number of cases with palpable nodules, reversible and irreversible skin disease.

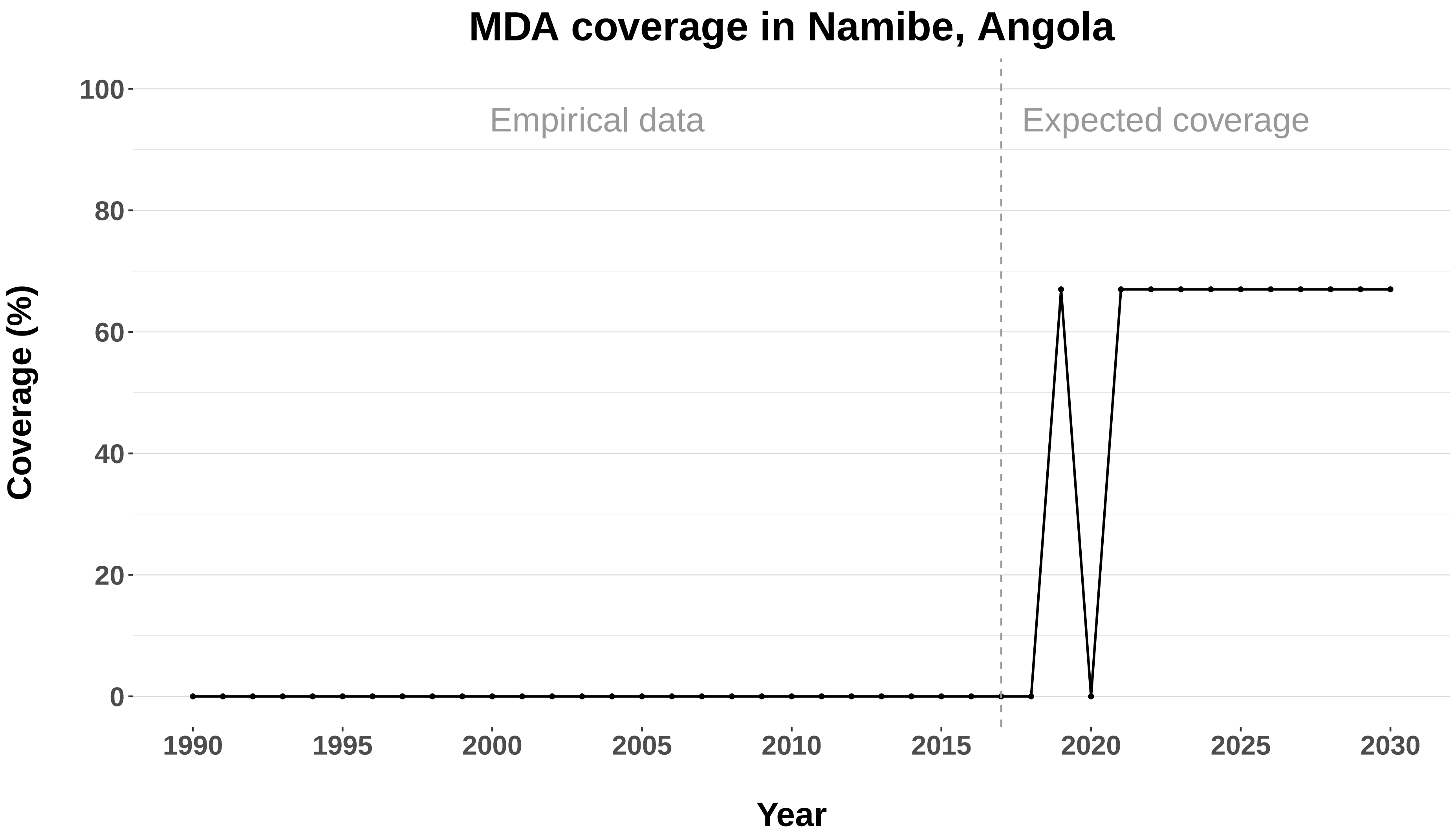
MDA coverage in Namibe, Angola
Empirical data
Expected coverage
Coverage (%)
100
80
60
40
20
0
1990
1995
2000
2005
2010
2015
2020
2025
2030
Year

**Project, country:**	**NY Benguela, Angola**
Bioclime:	
Endemicity level(s):	Hyperendemic
MDA start year:	2019
Mean MDA coverage per year:	61.4

	1990			2020			2030		
	N	%	DALYs	N	%	DALYs	N	%	DALYs
Total populaton at risk	57	100	_	128	100	_	168	100	_
**Infection:**									
Mf-positive	15	27.2	_	32	24.8	_	9	5.6	_
Mf-positive (age 5+)	15	27.2	_	32	24.8	_	9	5.6	_
Worm infected	20	35.7	_	44	34.6	_	29	17.3	_
**Clinical manifestations:**									
Palpable nodules	3	5.1	<0.5	6	4.9	<0.5	2	1.1	<0.5
Reversible skin disease:									
Severe itch	2	3.6	<0.5	4	3.0	1	1	0.4	<0.5
Reactive skin disease	1	2.4	<0.5	2	1.7	<0.5	<0.5	0.1	<0.5
**Reversible skin disease**	**3**	**6.1**	**<0.5**	**6**	**4.7**	**1**	**1**	**0.5**	**<0.5**
Irreversible skin disease:									
Mild depigmentation	1	0.9	<0.5	1	0.8	<0.5	1	0.5	<0.5
Severe depigmentation	<0.5	0.9	<0.5	1	0.7	<0.5	1	0.5	<0.5
Atrophy	<0.5	<0.05	<0.5	<0.5	<0.05	<0.5	<0.5	<0.05	<0.5
Hanging groin	<0.5	<0.05	<0.5	<0.5	<0.05	<0.5	<0.5	<0.05	<0.5
**Irreversible skin disease**	**1**	**1.8**	**<0.5**	**2**	**1.6**	**<0.5**	**2**	**1.0**	**<0.5**
**Total skin disease (OSD)**	**7**	**12.9**	**1**	**14**	**11.2**	**1**	**4**	**2.6**	**<0.5**
Eye disease (OED):									
Visual impairment	<0.5	<0.05	<0.5	<0.5	<0.05	<0.5	<0.5	<0.05	<0.5
Blindness	<0.5	<0.05	<0.5	<0.5	<0.05	<0.5	<0.5	<0.05	<0.5
**Eye disease (OED)**	**<0.5**	**<0.05**	**<0.5**	**<0.5**	**<0.05**	**<0.5**	**<0.5**	**<0.05**	**<0.5**
**Total all manifestations**	**7**	**13.0**	**1**	**14**	**11.2**	**1**	**4**	**2.6**	**<0.5**

Note 1: Population at risk, number of cases (N) and DALYs are presented in thousands.

Note 2: Total skin disease is defined as the sum of the number of cases with palpable nodules, reversible and irreversible skin disease.

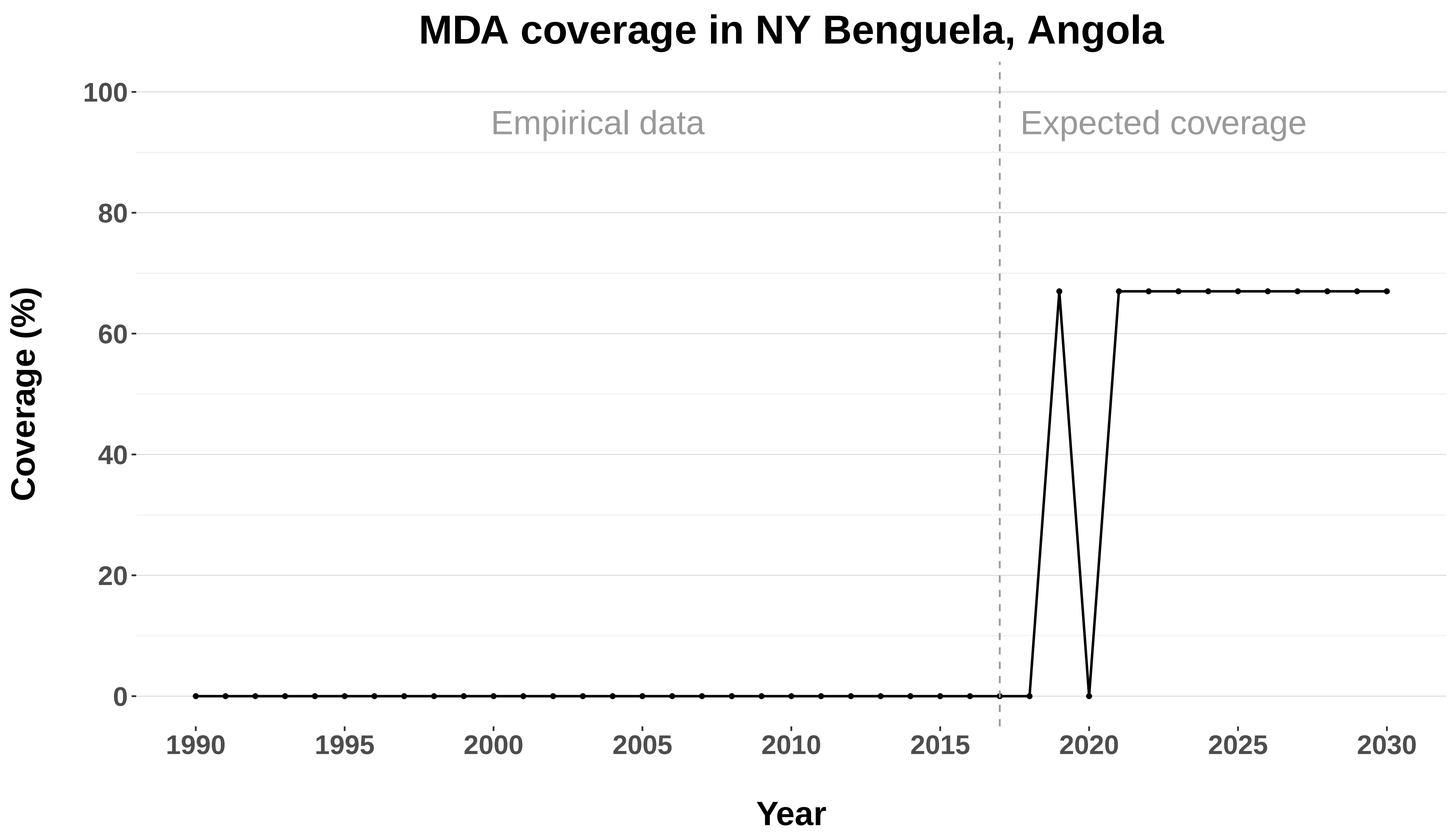

MDA coverage in NY Benguela, Angola
Empirical data
Expected coverage
Coverage (%)
100
80
60
40
20
0
1990
1995
2000
2005
2010
2015
2020
2025
2030
Year

**Project, country:**	**NY Cuanza Norte, Angola**
Bioclime:	
Endemicity level(s):	Mesoendemic
MDA start year:	2019
Mean MDA coverage per year:	61.4

	1990			2020			2030		
	N	%	DALYs	N	%	DALYs	N	%	DALYs
Total populaton at risk	9	100	_	21	100	_	27	100	_
**Infection:**									
Mf-positive	2	22.6	_	4	20.4	_	1	4.3	_
Mf-positive (age 5+)	2	22.6	_	4	20.4	_	1	4.3	_
Worm infected	3	29.8	_	6	28.9	_	4	14.2	_
**Clinical manifestations:**									
Palpable nodules	<0.5	2.4	<0.5	<0.5	2.3	<0.5	<0.5	0.5	<0.5
Reversible skin disease:									
Severe itch	<0.5	2.9	<0.5	1	2.4	<0.5	<0.5	0.3	<0.5
Reactive skin disease	<0.5	1.9	<0.5	<0.5	1.3	<0.5	<0.5	<0.05	<0.5
**Reversible skin disease**	**<0.5**	**4.8**	**<0.5**	**1**	**3.7**	**<0.5**	**<0.5**	**0.4**	**<0.5**
Irreversible skin disease:									
Mild depigmentation	<0.5	0.8	<0.5	<0.5	0.7	<0.5	<0.5	0.5	<0.5
Severe depigmentation	<0.5	0.7	<0.5	<0.5	0.6	<0.5	<0.5	0.4	<0.5
Atrophy	<0.5	<0.05	<0.5	<0.5	<0.05	<0.5	<0.5	<0.05	<0.5
Hanging groin	<0.5	<0.05	<0.5	<0.5	<0.05	<0.5	<0.5	<0.05	<0.5
**Irreversible skin disease**	**<0.5**	**1.5**	**<0.5**	**<0.5**	**1.4**	**<0.5**	**<0.5**	**0.9**	**<0.5**
**Total skin disease (OSD)**	**1**	**8.7**	**<0.5**	**2**	**7.4**	**<0.5**	**<0.5**	**1.7**	**<0.5**
Eye disease (OED):									
Visual impairment	<0.5	<0.05	<0.5	<0.5	<0.05	<0.5	<0.5	<0.05	<0.5
Blindness	<0.5	<0.05	<0.5	<0.5	<0.05	<0.5	<0.5	<0.05	<0.5
**Eye disease (OED)**	**<0.5**	**<0.05**	**<0.5**	**<0.5**	**<0.05**	**<0.5**	**<0.5**	**<0.05**	**<0.5**
**Total all manifestations**	**1**	**8.7**	**<0.5**	**2**	**7.4**	**<0.5**	**<0.5**	**1.7**	**<0.5**

Note 1: Population at risk, number of cases (N) and DALYs are presented in thousands.

Note 2: Total skin disease is defined as the sum of the number of cases with palpable nodules, reversible and irreversible skin disease.

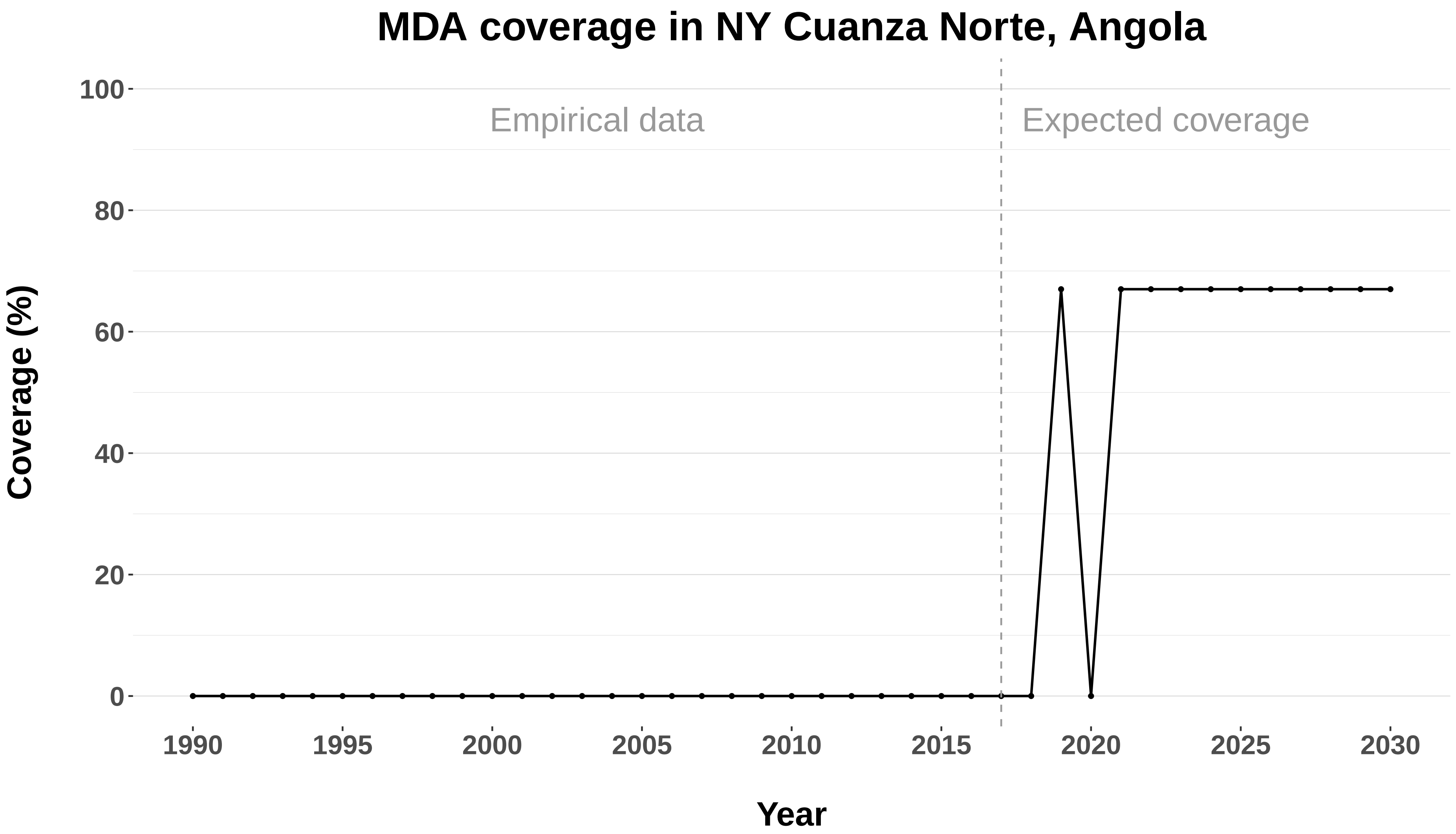

MDA coverage in NY Cuanza Norte, Angola
Empirical data
Expected coverage
Coverage (%)
100
80
60
40
20
0
1990
1995
2000
2005
2010
2015
2020
2025
2030
Year

**Project, country:**	**NY Huila, Angola**
Bioclime:	
Endemicity level(s):	Mesoendemic
MDA start year:	2019
Mean MDA coverage per year:	61.4

	1990			2020			2030		
	N	%	DALYs	N	%	DALYs	N	%	DALYs
Total populaton at risk	11	100	_	26	100	_	34	100	_
**Infection:**									
Mf-positive	3	26.5	_	6	24.4	_	2	5.6	_
Mf-positive (age 5+)	3	26.5	_	6	24.4	_	2	5.6	_
Worm infected	4	34.3	_	9	33.5	_	6	17.0	_
**Clinical manifestations:**									
Palpable nodules	1	4.5	<0.5	1	4.3	<0.5	<0.5	1.0	<0.5
Reversible skin disease:									
Severe itch	<0.5	3.6	<0.5	1	3.0	<0.5	<0.5	0.4	<0.5
Reactive skin disease	<0.5	2.5	<0.5	<0.5	1.7	<0.5	<0.5	0.1	<0.5
**Reversible skin disease**	**1**	**6.0**	**<0.5**	**1**	**4.7**	**<0.5**	**<0.5**	**0.5**	**<0.5**
Irreversible skin disease:									
Mild depigmentation	<0.5	0.9	<0.5	<0.5	0.9	<0.5	<0.5	0.6	<0.5
Severe depigmentation	<0.5	0.9	<0.5	<0.5	0.8	<0.5	<0.5	0.5	<0.5
Atrophy	<0.5	<0.05	<0.5	<0.5	<0.05	<0.5	<0.5	<0.05	<0.5
Hanging groin	<0.5	<0.05	<0.5	<0.5	<0.05	<0.5	<0.5	<0.05	<0.5
**Irreversible skin disease**	**<0.5**	**1.8**	**<0.5**	**<0.5**	**1.7**	**<0.5**	**<0.5**	**1.0**	**<0.5**
**Total skin disease (OSD)**	**1**	**12.3**	**<0.5**	**3**	**10.6**	**<0.5**	**1**	**2.5**	**<0.5**
Eye disease (OED):									
Visual impairment	<0.5	<0.05	<0.5	<0.5	<0.05	<0.5	<0.5	<0.05	<0.5
Blindness	<0.5	<0.05	<0.5	<0.5	<0.05	<0.5	<0.5	<0.05	<0.5
**Eye disease (OED)**	**<0.5**	**<0.05**	**<0.5**	**<0.5**	**<0.05**	**<0.5**	**<0.5**	**<0.05**	**<0.5**
**Total all manifestations**	**1**	**12.4**	**<0.5**	**3**	**10.7**	**<0.5**	**1**	**2.5**	**<0.5**

Note 1: Population at risk, number of cases (N) and DALYs are presented in thousands.

Note 2: Total skin disease is defined as the sum of the number of cases with palpable nodules, reversible and irreversible skin disease.

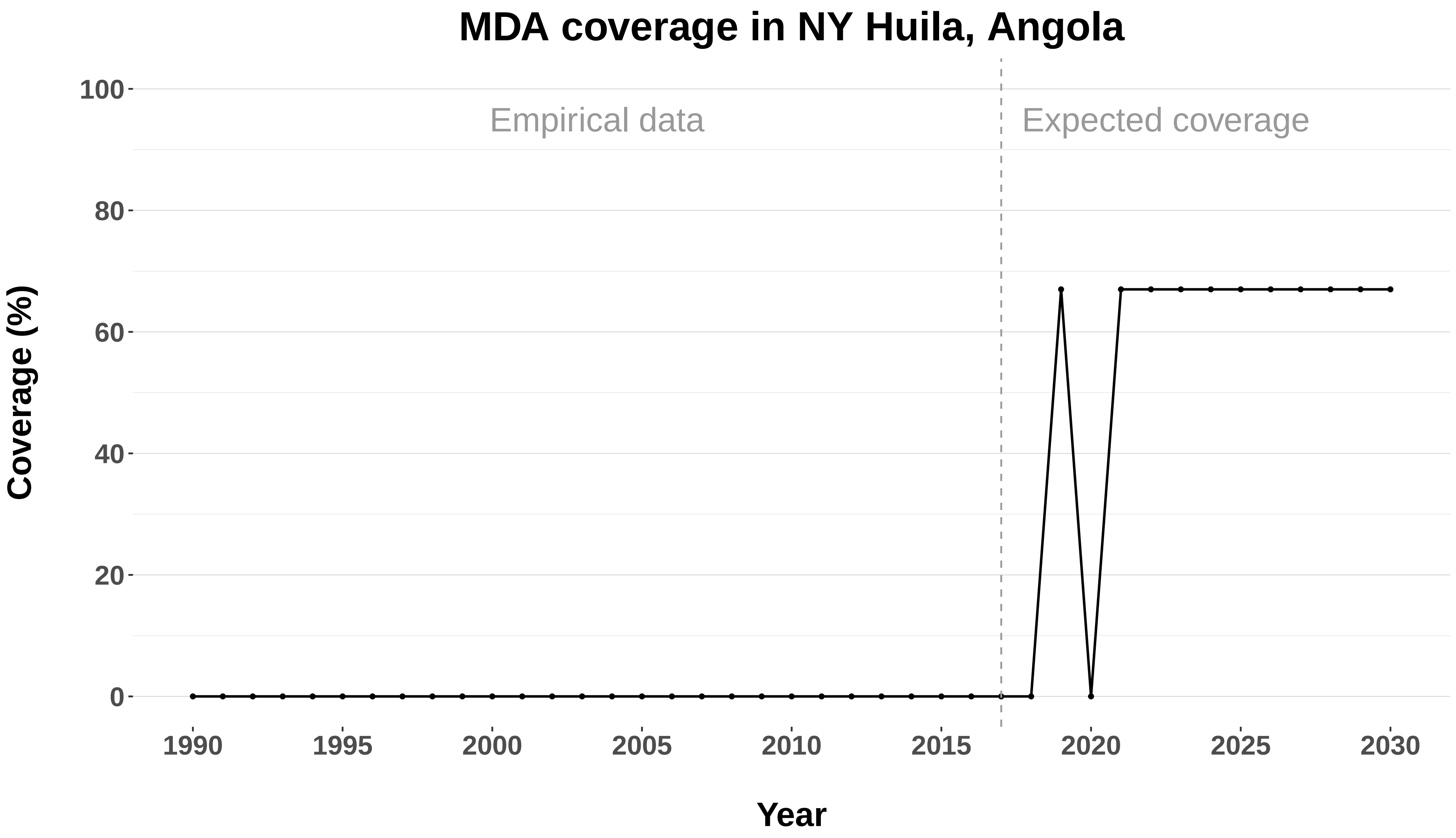

MDA coverage in NY Huila, Angola
Empirical data
Expected coverage
Coverage (%)
100
80
60
40
20
0
1990
1995
2000
2005
2010
2015
2020
2025
2030
Year

**Project, country:**	**NY Lunda Norte, Angola**
Bioclime:	
Endemicity level(s):	Mesoendemic
MDA start year:	2019
Mean MDA coverage per year:	61.4

	1990			2020			2030		
	N	%	DALYs	N	%	DALYs	N	%	DALYs
Total populaton at risk	33	100	_	75	100	_	99	100	_
**Infection:**									
Mf-positive	9	25.8	_	18	23.8	_	5	5.4	_
Mf-positive (age 5+)	9	25.8	_	18	23.8	_	5	5.4	_
Worm infected	11	33.9	_	25	33.0	_	16	16.5	_
**Clinical manifestations:**									
Palpable nodules	1	4.1	<0.5	3	4.0	<0.5	1	0.9	<0.5
Reversible skin disease:									
Severe itch	1	3.3	<0.5	2	2.9	<0.5	<0.5	0.4	<0.5
Reactive skin disease	1	2.3	<0.5	1	1.6	<0.5	<0.5	0.1	<0.5
**Reversible skin disease**	**2**	**5.6**	**<0.5**	**3**	**4.5**	**<0.5**	**<0.5**	**0.4**	**<0.5**
Irreversible skin disease:									
Mild depigmentation	<0.5	0.9	<0.5	1	0.8	<0.5	1	0.5	<0.5
Severe depigmentation	<0.5	0.8	<0.5	1	0.7	<0.5	<0.5	0.4	<0.5
Atrophy	<0.5	<0.05	<0.5	<0.5	<0.05	<0.5	<0.5	<0.05	<0.5
Hanging groin	<0.5	<0.05	<0.5	<0.5	<0.05	<0.5	<0.5	<0.05	<0.5
**Irreversible skin disease**	**1**	**1.7**	**<0.5**	**1**	**1.5**	**<0.5**	**1**	**1.0**	**<0.5**
**Total skin disease (OSD)**	**4**	**11.3**	**<0.5**	**8**	**10.1**	**1**	**2**	**2.3**	**<0.5**
Eye disease (OED):									
Visual impairment	<0.5	<0.05	<0.5	<0.5	<0.05	<0.5	<0.5	<0.05	<0.5
Blindness	<0.5	<0.05	<0.5	<0.5	<0.05	<0.5	<0.5	<0.05	<0.5
**Eye disease (OED)**	**<0.5**	**<0.05**	**<0.5**	**<0.5**	**<0.05**	**<0.5**	**<0.5**	**<0.05**	**<0.5**
**Total all manifestations**	**4**	**11.3**	**<0.5**	**8**	**10.1**	**1**	**2**	**2.3**	**<0.5**

Note 1: Population at risk, number of cases (N) and DALYs are presented in thousands.

Note 2: Total skin disease is defined as the sum of the number of cases with palpable nodules, reversible and irreversible skin disease.

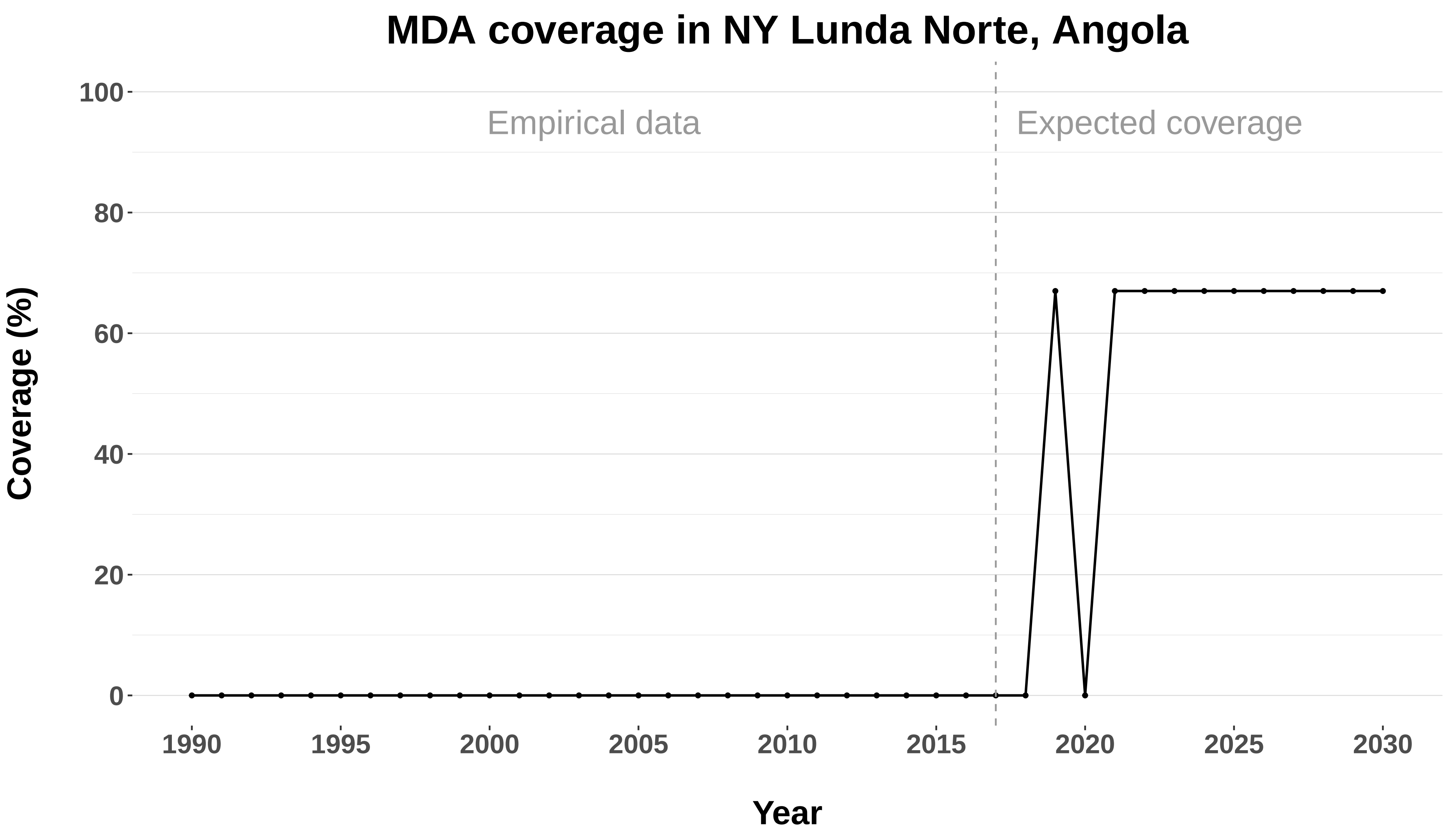

MDA coverage in NY Lunda Norte, Angola
Empirical data
Expected coverage
Coverage (%)
100
80
60
40
20
0
1990
1995
2000
2005
2010
2015
2020
2025
2030
Year

**Project, country:**	**NY Moxico 1, Angola**
Bioclime:	
Endemicity level(s):	Mesoendemic
MDA start year:	2019
Mean MDA coverage per year:	61.4

	1990			2020			2030		
	N	%	DALYs	N	%	DALYs	N	%	DALYs
Total populaton at risk	172	100	_	388	100	_	510	100	_
**Infection:**									
Mf-positive	34	19.9	_	62	16.1	_	14	2.8	_
Mf-positive (age 5+)	34	19.8	_	62	16.1	_	14	2.8	_
Worm infected	48	27.8	_	96	24.6	_	55	10.7	_
**Clinical manifestations:**									
Palpable nodules	4	2.1	<0.5	7	1.7	<0.5	2	0.3	<0.5
Reversible skin disease:									
Severe itch	4	2.3	1	7	1.7	1	1	0.2	<0.5
Reactive skin disease	3	1.5	<0.5	4	0.9	<0.5	<0.5	<0.05	<0.5
**Reversible skin disease**	**6**	**3.8**	**1**	**10**	**2.6**	**1**	**1**	**0.2**	**<0.5**
Irreversible skin disease:									
Mild depigmentation	1	0.7	<0.5	2	0.6	<0.5	2	0.3	<0.5
Severe depigmentation	1	0.6	<0.5	2	0.4	<0.5	1	0.3	<0.5
Atrophy	<0.5	<0.05	<0.5	<0.5	<0.05	<0.5	<0.5	<0.05	<0.5
Hanging groin	<0.5	<0.05	<0.5	<0.5	<0.05	<0.5	<0.5	<0.05	<0.5
**Irreversible skin disease**	**2**	**1.2**	**<0.5**	**4**	**1.0**	**<0.5**	**3**	**0.6**	**<0.5**
**Total skin disease (OSD)**	**12**	**7.0**	**1**	**21**	**5.3**	**2**	**6**	**1.2**	**<0.5**
Eye disease (OED):									
Visual impairment	<0.5	<0.05	<0.5	<0.5	<0.05	<0.5	<0.5	<0.05	<0.5
Blindness	<0.5	<0.05	<0.5	<0.5	<0.05	<0.5	<0.5	<0.05	<0.5
**Eye disease (OED)**	**<0.5**	**<0.05**	**<0.5**	**<0.5**	**<0.05**	**<0.5**	**<0.5**	**<0.05**	**<0.5**
**Total all manifestations**	**12**	**7.0**	**1**	**21**	**5.4**	**2**	**6**	**1.2**	**<0.5**

Note 1: Population at risk, number of cases (N) and DALYs are presented in thousands.

Note 2: Total skin disease is defined as the sum of the number of cases with palpable nodules, reversible and irreversible skin disease.

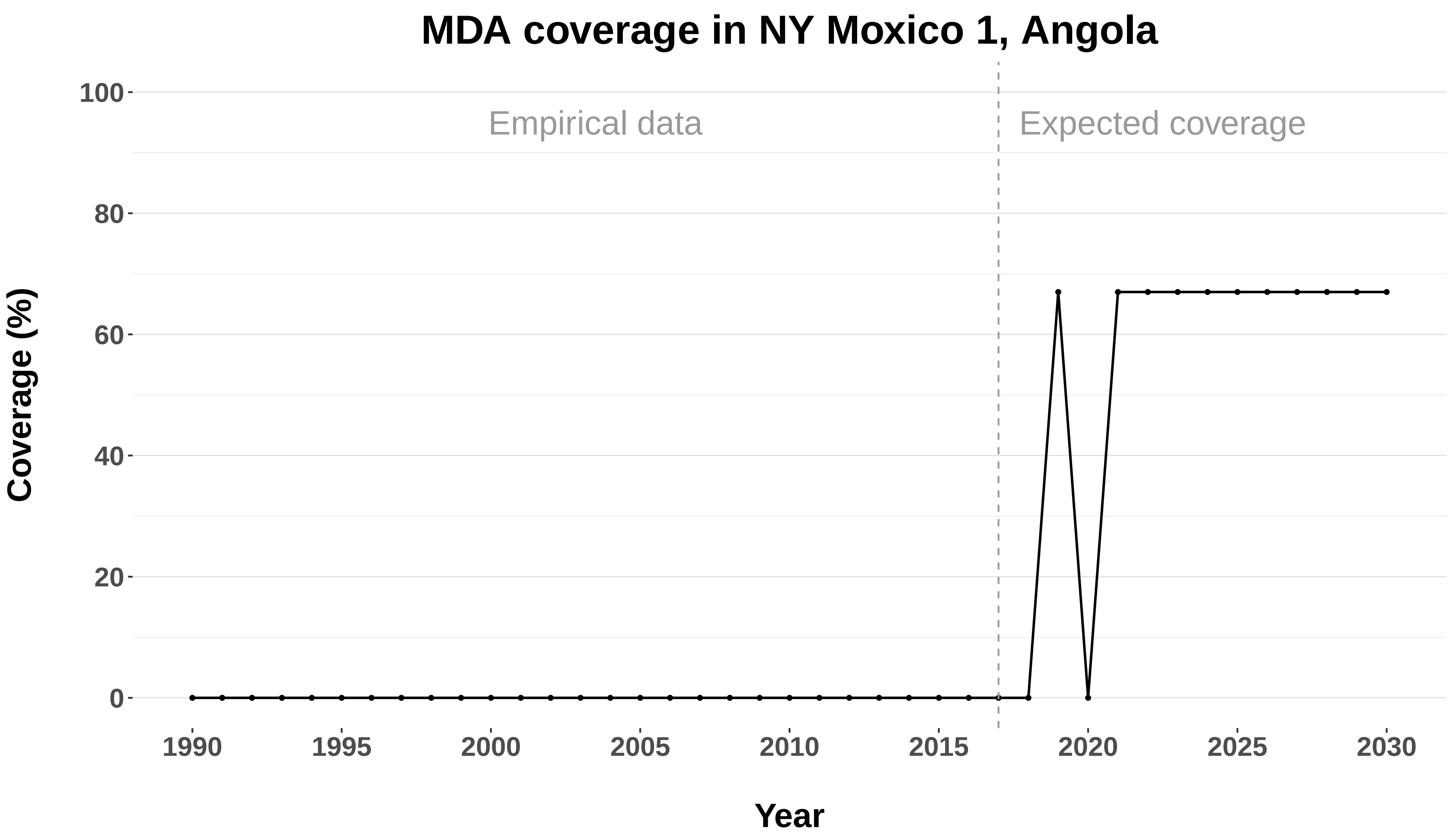

MDA coverage in NY Moxico 1, Angola
Empirical data
Expected coverage
Coverage (%)
100
80
60
40
20
0
1990
1995
2000
2005
2010
2015
2020
2025
2030
Year

**Project, country:**	**P5Angola, Angola**
Bioclime:	
Endemicity level(s):	Hypoendemic
MDA start year:	2025
Mean MDA coverage per year:	65.0

	1990			2020			2030		
	N	%	DALYs	N	%	DALYs	N	%	DALYs
Total populaton at risk	131	100	_	296	100	_	388	100	_
**Infection:**									
Mf-positive	29	22.4	_	63	21.2	_	50	12.8	_
Mf-positive (age 5+)	29	22.4	_	63	21.2	_	50	12.8	_
Worm infected	39	30.0	_	85	28.7	_	90	23.2	_
**Clinical manifestations:**									
Palpable nodules	3	2.6	<0.5	7	2.4	<0.5	6	1.6	<0.5
Reversible skin disease:									
Severe itch	4	2.8	1	8	2.6	1	4	1.1	1
Reactive skin disease	2	1.8	<0.5	5	1.7	<0.5	1	0.3	<0.5
**Reversible skin disease**	**6**	**4.6**	**1**	**13**	**4.3**	**2**	**5**	**1.4**	**1**
Irreversible skin disease:									
Mild depigmentation	1	0.8	<0.5	2	0.7	<0.5	2	0.6	<0.5
Severe depigmentation	1	0.6	<0.5	2	0.6	<0.5	2	0.5	<0.5
Atrophy	<0.5	<0.05	<0.5	<0.5	<0.05	<0.5	<0.5	<0.05	<0.5
Hanging groin	<0.5	<0.05	<0.5	<0.5	<0.05	<0.5	<0.5	<0.05	<0.5
**Irreversible skin disease**	**2**	**1.4**	**<0.5**	**4**	**1.3**	**<0.5**	**4**	**1.0**	**<0.5**
**Total skin disease (OSD)**	**11**	**8.6**	**1**	**24**	**8.0**	**2**	**15**	**4.0**	**1**
Eye disease (OED):									
Visual impairment	<0.5	<0.05	<0.5	<0.5	<0.05	<0.5	<0.5	<0.05	<0.5
Blindness	<0.5	<0.05	<0.5	<0.5	<0.05	<0.5	<0.5	<0.05	<0.5
**Eye disease (OED)**	**<0.5**	**<0.05**	**<0.5**	**<0.5**	**<0.05**	**<0.5**	**<0.5**	**<0.05**	**<0.5**
**Total all manifestations**	**11**	**8.6**	**1**	**24**	**8.0**	**2**	**16**	**4.0**	**1**

Note 1: Population at risk, number of cases (N) and DALYs are presented in thousands.

Note 2: Total skin disease is defined as the sum of the number of cases with palpable nodules, reversible and irreversible skin disease.

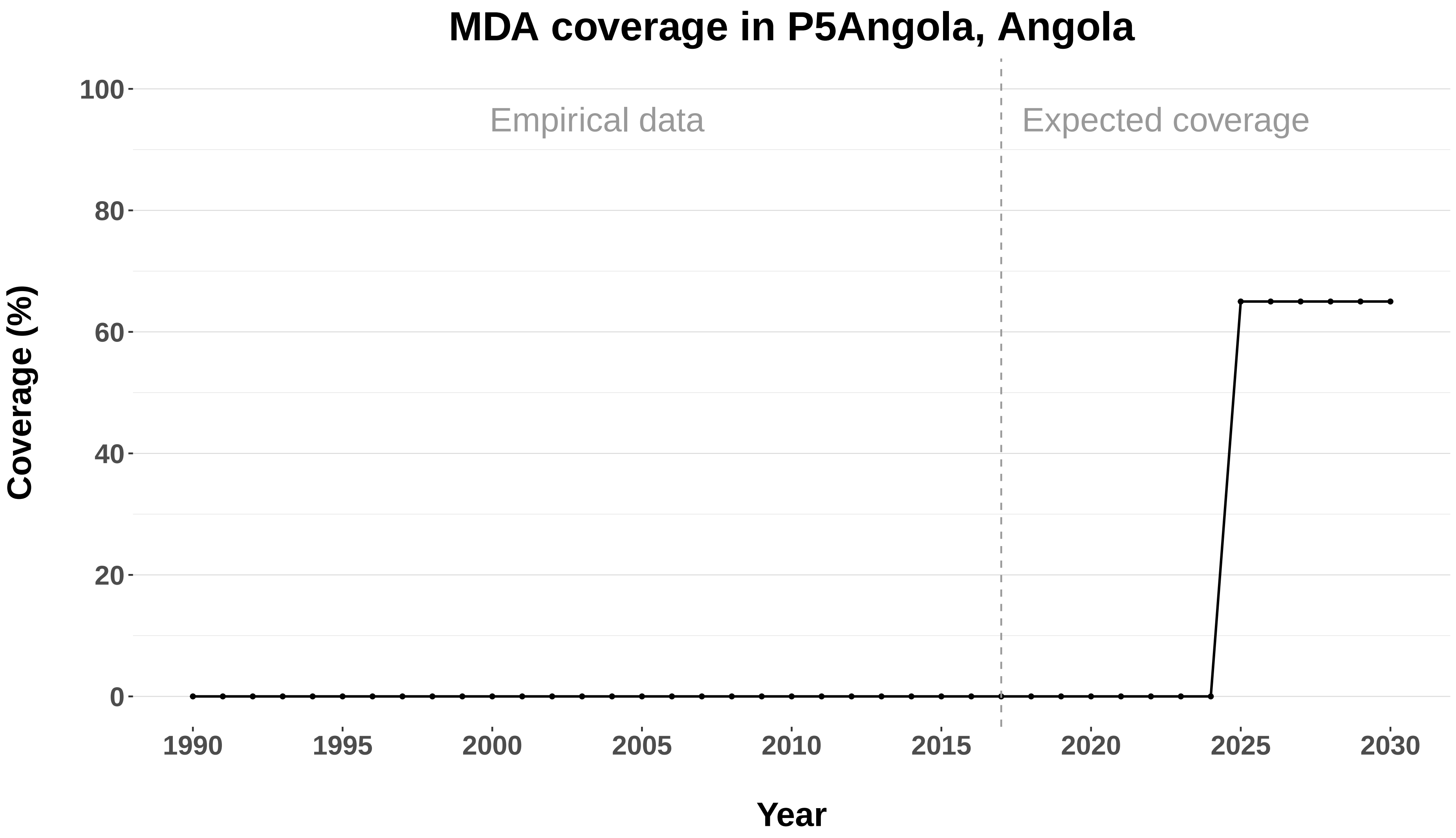
MDA coverage in P5Angola, Angola
Empirical data
Expected coverage
Coverage (%)
Year
0
20
40
60
80
100
1990
1995
2000
2005
2010
2015
2020
2025
2030

**Project, country:**	**Uige, Angola**
Bioclime:	
Endemicity level(s):	Hyperendemic
MDA start year:	2019
Mean MDA coverage per year:	61.4

	1990			2020			2030		
	N	%	DALYs	N	%	DALYs	N	%	DALYs
Total populaton at risk	95	100	_	214	100	_	280	100	_
**Infection:**									
Mf-positive	26	27.3	_	52	24.4	_	15	5.2	_
Mf-positive (age 5+)	26	27.3	_	52	24.3	_	15	5.2	_
Worm infected	34	36.1	_	74	34.5	_	47	16.8	_
**Clinical manifestations:**									
Palpable nodules	5	5.2	<0.5	10	4.8	<0.5	3	1.0	<0.5
Reversible skin disease:									
Severe itch	3	3.5	1	6	2.9	1	1	0.4	<0.5
Reactive skin disease	2	2.3	<0.5	3	1.6	<0.5	<0.5	0.1	<0.5
**Reversible skin disease**	**6**	**5.9**	**1**	**10**	**4.5**	**1**	**1**	**0.4**	**<0.5**
Irreversible skin disease:									
Mild depigmentation	1	0.9	<0.5	2	0.8	<0.5	1	0.5	<0.5
Severe depigmentation	1	0.8	<0.5	2	0.7	<0.5	1	0.4	<0.5
Atrophy	<0.5	<0.05	<0.5	<0.5	<0.05	<0.5	<0.5	<0.05	<0.5
Hanging groin	<0.5	<0.05	<0.5	<0.5	<0.05	<0.5	<0.5	<0.05	<0.5
**Irreversible skin disease**	**2**	**1.7**	**<0.5**	**3**	**1.5**	**<0.5**	**3**	**0.9**	**<0.5**
**Total skin disease (OSD)**	**12**	**12.7**	**1**	**23**	**10.9**	**2**	**7**	**2.4**	**<0.5**
Eye disease (OED):									
Visual impairment	<0.5	<0.05	<0.5	<0.5	<0.05	<0.5	<0.5	<0.05	<0.5
Blindness	<0.5	<0.05	<0.5	<0.5	<0.05	<0.5	<0.5	<0.05	<0.5
**Eye disease (OED)**	**<0.5**	**<0.05**	**<0.5**	**<0.5**	**<0.05**	**<0.5**	**<0.5**	**<0.05**	**<0.5**
**Total all manifestations**	**12**	**12.7**	**1**	**23**	**10.9**	**2**	**7**	**2.4**	**<0.5**

Note 1: Population at risk, number of cases (N) and DALYs are presented in thousands.

Note 2: Total skin disease is defined as the sum of the number of cases with palpable nodules, reversible and irreversible skin disease.

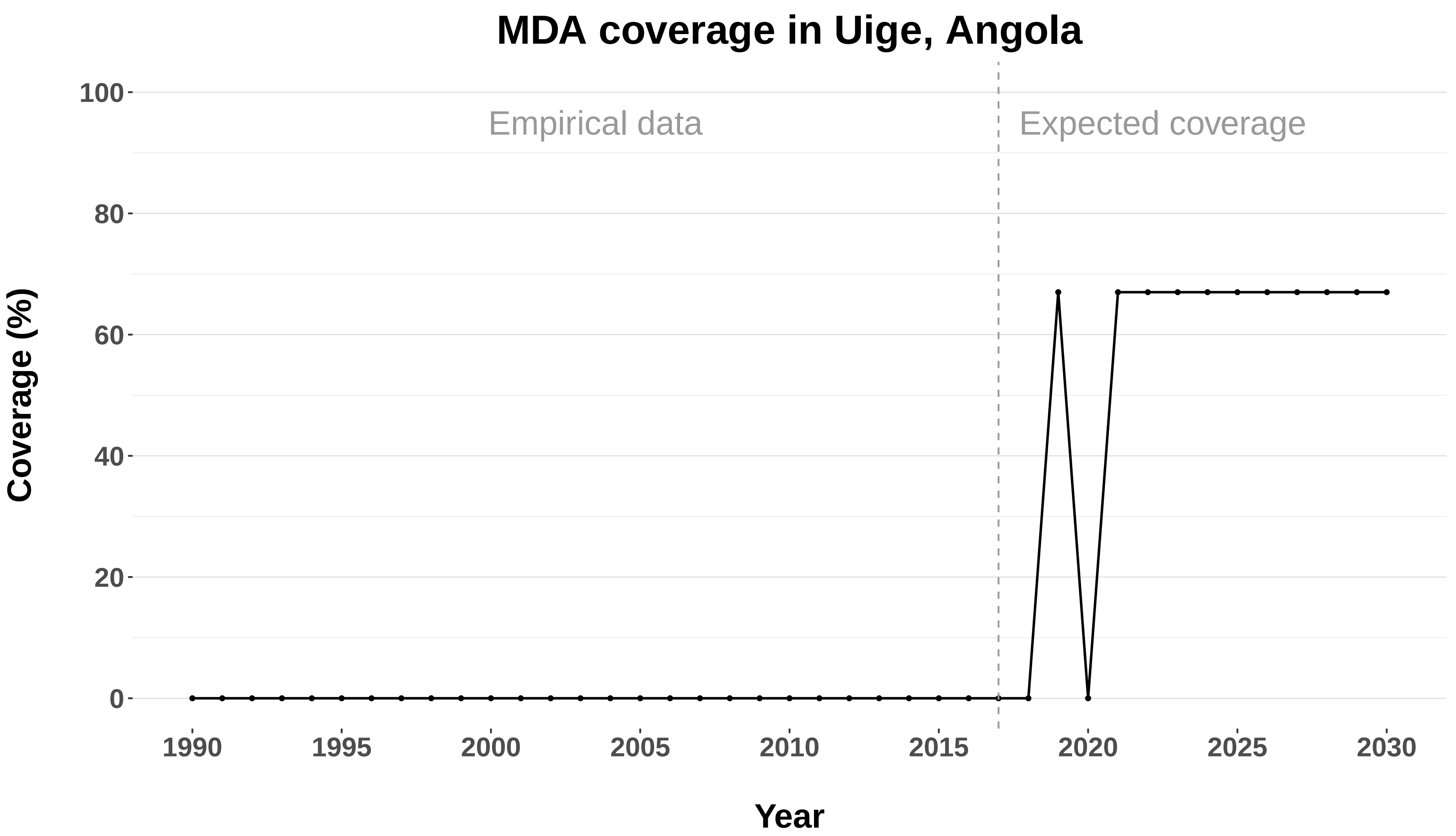
MDA coverage in Uige, Angola
Empirical data
Expected coverage
Coverage (%)
100
80
60
40
20
0
1990
1995
2000
2005
2010
2015
2020
2025
2030
Year

**Project, country:**	**Zaire, Angola**
Bioclime:	
Endemicity level(s):	Mesoendemic
MDA start year:	2019
Mean MDA coverage per year:	61.4

	1990			2020			2030		
	N	%	DALYs	N	%	DALYs	N	%	DALYs
Total populaton at risk	7	100	_	16	100	_	21	100	_
**Infection:**									
Mf-positive	1	20.3	_	3	16.7	_	1	2.9	_
Mf-positive (age 5+)	1	20.3	_	3	16.7	_	1	2.9	_
Worm infected	2	28.0	_	4	25.3	_	2	11.1	_
**Clinical manifestations:**									
Palpable nodules	<0.5	2.1	<0.5	<0.5	1.8	<0.5	<0.5	0.3	<0.5
Reversible skin disease:									
Severe itch	<0.5	2.4	<0.5	<0.5	1.7	<0.5	<0.5	0.2	<0.5
Reactive skin disease	<0.5	1.6	<0.5	<0.5	1.0	<0.5	<0.5	<0.05	<0.5
**Reversible skin disease**	**<0.5**	**4.0**	**<0.5**	**<0.5**	**2.7**	**<0.5**	**<0.5**	**0.2**	**<0.5**
Irreversible skin disease:									
Mild depigmentation	<0.5	0.7	<0.5	<0.5	0.6	<0.5	<0.5	0.3	<0.5
Severe depigmentation	<0.5	0.6	<0.5	<0.5	0.5	<0.5	<0.5	0.3	<0.5
Atrophy	<0.5	<0.05	<0.5	<0.5	<0.05	<0.5	<0.5	<0.05	<0.5
Hanging groin	<0.5	<0.05	<0.5	<0.5	<0.05	<0.5	<0.5	<0.05	<0.5
**Irreversible skin disease**	**<0.5**	**1.3**	**<0.5**	**<0.5**	**1.1**	**<0.5**	**<0.5**	**0.6**	**<0.5**
**Total skin disease (OSD)**	**1**	**7.3**	**<0.5**	**1**	**5.6**	**<0.5**	**<0.5**	**1.2**	**<0.5**
Eye disease (OED):									
Visual impairment	<0.5	<0.05	<0.5	<0.5	<0.05	<0.5	<0.5	<0.05	<0.5
Blindness	<0.5	<0.05	<0.5	<0.5	<0.05	<0.5	<0.5	<0.05	<0.5
**Eye disease (OED)**	**<0.5**	**<0.05**	**<0.5**	**<0.5**	**<0.05**	**<0.5**	**<0.5**	**<0.05**	**<0.5**
**Total all manifestations**	**1**	**7.4**	**<0.5**	**1**	**5.6**	**<0.5**	**<0.5**	**1.2**	**<0.5**

Note 1: Population at risk, number of cases (N) and DALYs are presented in thousands.

Note 2: Total skin disease is defined as the sum of the number of cases with palpable nodules, reversible and irreversible skin disease.

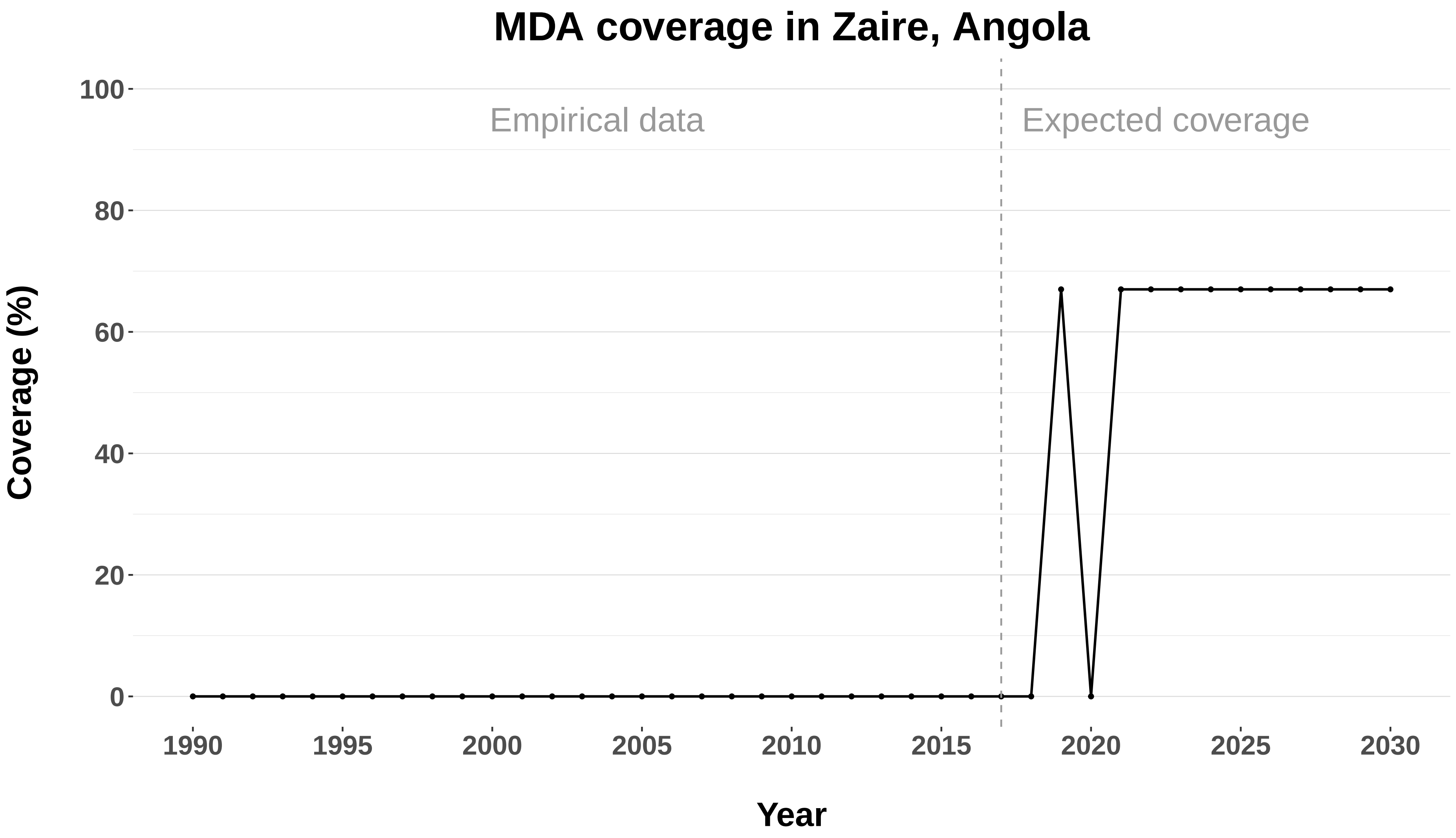

MDA coverage in Zaire, Angola
Empirical data
Expected coverage
Coverage (%)
100
80
60
40
20
0
1990
1995
2000
2005
2010
2015
2020
2025
2030
Year

**Project, country:**	**Bururi, Burundi**
Bioclime:	
Endemicity level(s):	Mesoendemic
MDA start year:	2006
Mean MDA coverage per year:	69.7

	1990			2020			2030		
	N	%	DALYs	N	%	DALYs	N	%	DALYs
Total populaton at risk	220	100	_	497	100	_	652	100	_
**Infection:**									
Mf-positive	53	23.9	_	4	0.9	_	<0.5	<0.05	_
Mf-positive (age 5+)	53	23.9	_	4	0.9	_	<0.5	<0.05	_
Worm infected	71	32.2	_	35	7.1	_	2	0.2	_
**Clinical manifestations:**									
Palpable nodules	8	3.5	<0.5	1	0.2	<0.5	<0.5	<0.05	<0.5
Reversible skin disease:									
Severe itch	6	2.9	1	<0.5	0.1	<0.5	<0.5	<0.05	<0.5
Reactive skin disease	4	1.9	<0.5	<0.5	<0.05	<0.5	<0.5	<0.05	<0.5
**Reversible skin disease**	**11**	**4.8**	**1**	**<0.5**	**0.1**	**<0.5**	**<0.5**	**<0.05**	**<0.5**
Irreversible skin disease:									
Mild depigmentation	2	0.8	<0.5	2	0.4	<0.5	1	0.2	<0.5
Severe depigmentation	1	0.7	<0.5	1	0.3	<0.5	1	0.2	<0.5
Atrophy	<0.5	<0.05	<0.5	<0.5	<0.05	<0.5	<0.5	<0.05	<0.5
Hanging groin	<0.5	<0.05	<0.5	<0.5	<0.05	<0.5	<0.5	<0.05	<0.5
**Irreversible skin disease**	**3**	**1.4**	**<0.5**	**3**	**0.7**	**<0.5**	**2**	**0.3**	**<0.5**
**Total skin disease (OSD)**	**22**	**9.8**	**2**	**5**	**0.9**	**<0.5**	**2**	**0.3**	**<0.5**
Eye disease (OED):									
Visual impairment	<0.5	<0.05	<0.5	<0.5	<0.05	<0.5	<0.5	<0.05	<0.5
Blindness	<0.5	<0.05	<0.5	<0.5	<0.05	<0.5	<0.5	<0.05	<0.5
**Eye disease (OED)**	**<0.5**	**<0.05**	**<0.5**	**<0.5**	**<0.05**	**<0.5**	**<0.5**	**<0.05**	**<0.5**
**Total all manifestations**	**22**	**9.8**	**2**	**5**	**0.9**	**<0.5**	**2**	**0.4**	**<0.5**

Note 1: Population at risk, number of cases (N) and DALYs are presented in thousands.

Note 2: Total skin disease is defined as the sum of the number of cases with palpable nodules, reversible and irreversible skin disease.

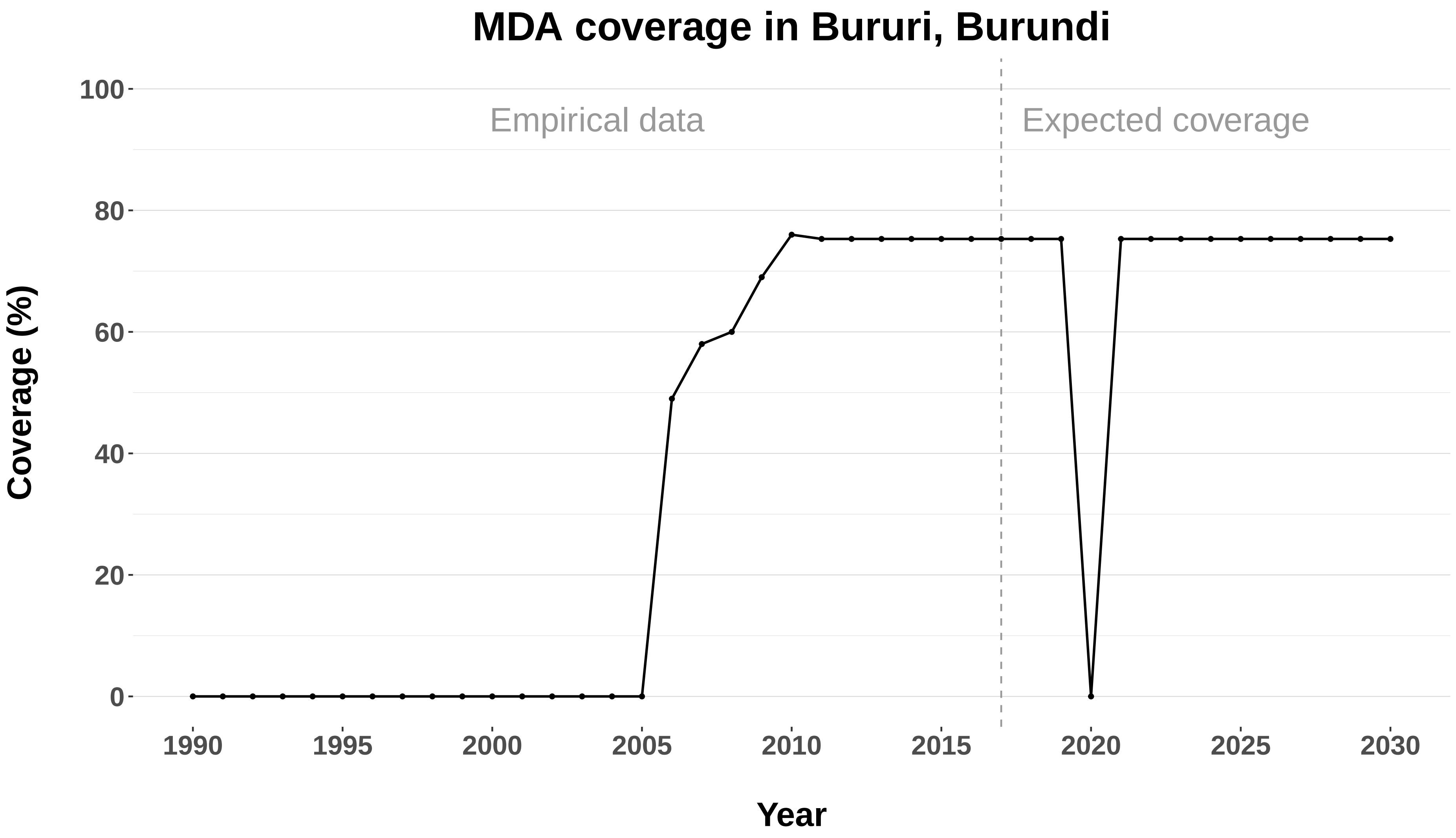
MDA coverage in Bururi, Burundi
Empirical data
Expected coverage
Coverage (%)
0
20
40
60
80
100
1990
1995
2000
2005
2010
2015
2020
2025
2030
Year

**Project, country:**	**Cibitoke-Bubanza, Burundi**
Bioclime:	
Endemicity level(s):	Mesoendemic
MDA start year:	2005
Mean MDA coverage per year:	72.4

	1990			2020			2030		
	N	%	DALYs	N	%	DALYs	N	%	DALYs
Total populaton at risk	526	100	_	1,187	100	_	1,557	100	_
**Infection:**									
Mf-positive	173	32.9	_	11	0.9	_	<0.5	<0.05	_
Mf-positive (age 5+)	173	32.9	_	11	0.9	_	<0.5	<0.05	_
Worm infected	222	42.2	_	99	8.3	_	4	0.2	_
**Clinical manifestations:**									
Palpable nodules	43	8.1	<0.5	4	0.3	<0.5	<0.5	<0.05	<0.5
Reversible skin disease:									
Severe itch	24	4.5	4	1	0.1	<0.5	<0.5	<0.05	<0.5
Reactive skin disease	17	3.1	1	<0.5	<0.05	<0.5	<0.5	<0.05	<0.5
**Reversible skin disease**	**40**	**7.7**	**5**	**1**	**0.1**	**<0.5**	**<0.5**	**<0.05**	**<0.5**
Irreversible skin disease:									
Mild depigmentation	6	1.1	<0.5	6	0.5	<0.5	4	0.3	<0.5
Severe depigmentation	6	1.0	<0.5	6	0.5	<0.5	4	0.2	<0.5
Atrophy	<0.5	<0.05	<0.5	<0.5	<0.05	<0.5	<0.5	<0.05	<0.5
Hanging groin	<0.5	<0.05	<0.5	<0.5	<0.05	<0.5	<0.5	<0.05	<0.5
**Irreversible skin disease**	**11**	**2.1**	**<0.5**	**12**	**1.0**	**<0.5**	**8**	**0.5**	**<0.5**
**Total skin disease (OSD)**	**94**	**17.9**	**6**	**17**	**1.4**	**1**	**8**	**0.5**	**<0.5**
Eye disease (OED):									
Visual impairment	<0.5	<0.05	<0.5	<0.5	<0.05	<0.5	<0.5	<0.05	<0.5
Blindness	<0.5	<0.05	<0.5	<0.5	<0.05	<0.5	<0.5	<0.05	<0.5
**Eye disease (OED)**	**<0.5**	**<0.05**	**<0.5**	**<0.5**	**<0.05**	**<0.5**	**<0.5**	**<0.05**	**<0.5**
**Total all manifestations**	**94**	**17.9**	**6**	**17**	**1.4**	**1**	**8**	**0.5**	**<0.5**

Note 1: Population at risk, number of cases (N) and DALYs are presented in thousands.

Note 2: Total skin disease is defined as the sum of the number of cases with palpable nodules, reversible and irreversible skin disease.

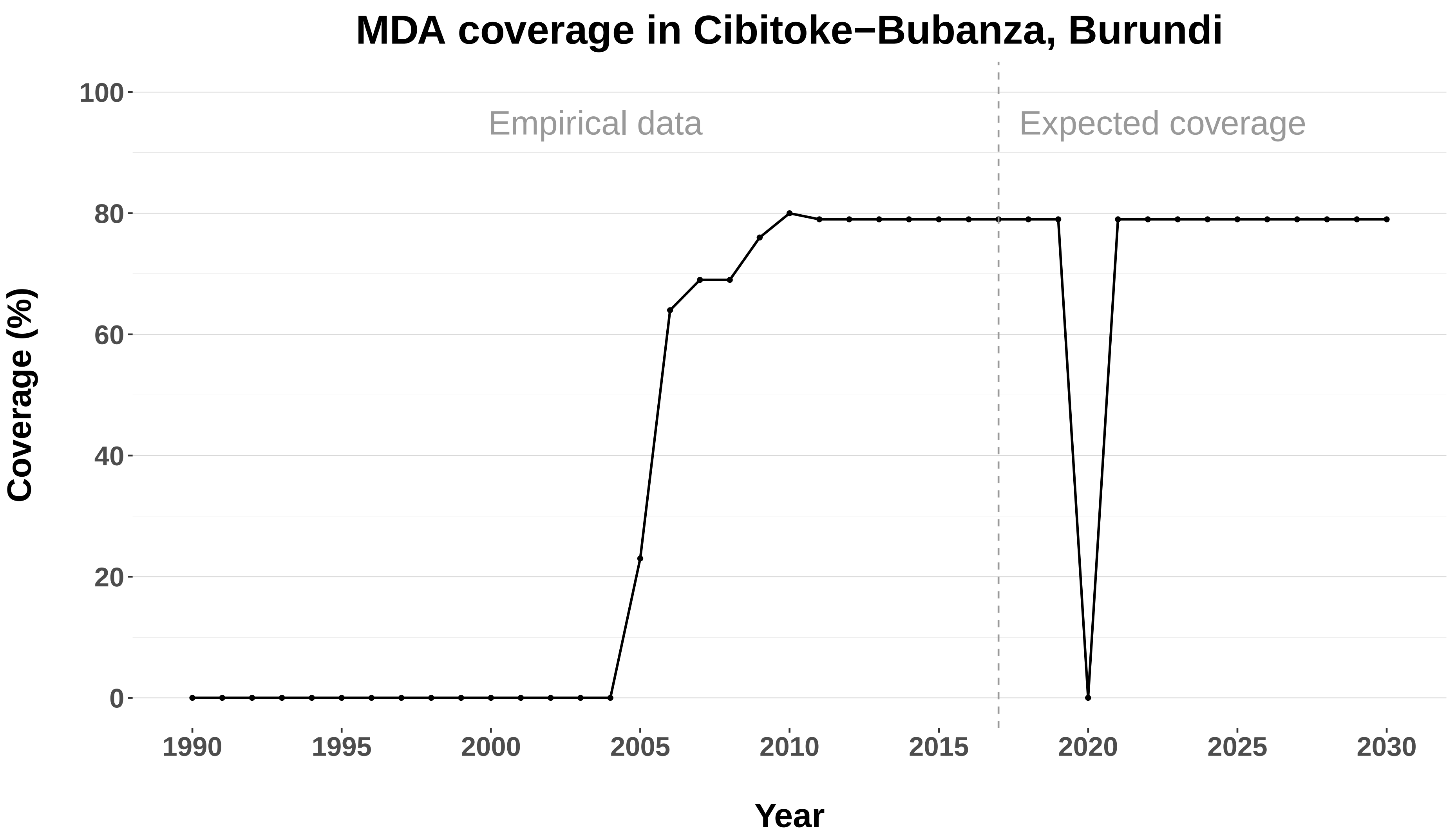

MDA coverage in Cibitoke−Bubanza, Burundi
Empirical data
Expected coverage
Coverage (%)
0
20
40
60
80
100
1990
1995
2000
2005
2010
2015
2020
2025
2030
Year

**Project, country:**	**P5Burundi, Burundi**
Bioclime:	
Endemicity level(s):	Hypoendemic
MDA start year:	2025
Mean MDA coverage per year:	65.0

	1990			2020			2030		
	N	%	DALYs	N	%	DALYs	N	%	DALYs
Total populaton at risk	415	100	_	936	100	_	1,228	100	_
**Infection:**									
Mf-positive	98	23.6	_	203	21.7	_	163	13.3	_
Mf-positive (age 5+)	98	23.6	_	203	21.7	_	163	13.3	_
Worm infected	131	31.5	_	278	29.7	_	294	24.0	_
**Clinical manifestations:**									
Palpable nodules	14	3.3	<0.5	28	3.0	<0.5	25	2.0	<0.5
Reversible skin disease:									
Severe itch	12	3.0	2	25	2.7	5	14	1.1	3
Reactive skin disease	8	2.0	<0.5	17	1.8	1	4	0.3	<0.5
**Reversible skin disease**	**20**	**4.9**	**3**	**42**	**4.5**	**5**	**17**	**1.4**	**3**
Irreversible skin disease:									
Mild depigmentation	3	0.8	<0.5	7	0.7	<0.5	7	0.6	<0.5
Severe depigmentation	3	0.7	<0.5	6	0.6	<0.5	6	0.5	<0.5
Atrophy	<0.5	<0.05	<0.5	<0.5	<0.05	<0.5	<0.5	<0.05	<0.5
Hanging groin	<0.5	<0.05	<0.5	<0.5	<0.05	<0.5	<0.5	<0.05	<0.5
**Irreversible skin disease**	**6**	**1.5**	**<0.5**	**13**	**1.3**	**<0.5**	**13**	**1.0**	**<0.5**
**Total skin disease (OSD)**	**40**	**9.7**	**3**	**83**	**8.9**	**6**	**55**	**4.5**	**3**
Eye disease (OED):									
Visual impairment	<0.5	<0.05	<0.5	<0.5	<0.05	<0.5	<0.5	<0.05	<0.5
Blindness	<0.5	<0.05	<0.5	<0.5	<0.05	<0.5	<0.5	<0.05	<0.5
**Eye disease (OED)**	**<0.5**	**<0.05**	**<0.5**	**<0.5**	**<0.05**	**<0.5**	**<0.5**	**<0.05**	**<0.5**
**Total all manifestations**	**40**	**9.7**	**3**	**83**	**8.9**	**7**	**55**	**4.5**	**4**

Note 1: Population at risk, number of cases (N) and DALYs are presented in thousands.

Note 2: Total skin disease is defined as the sum of the number of cases with palpable nodules, reversible and irreversible skin disease.

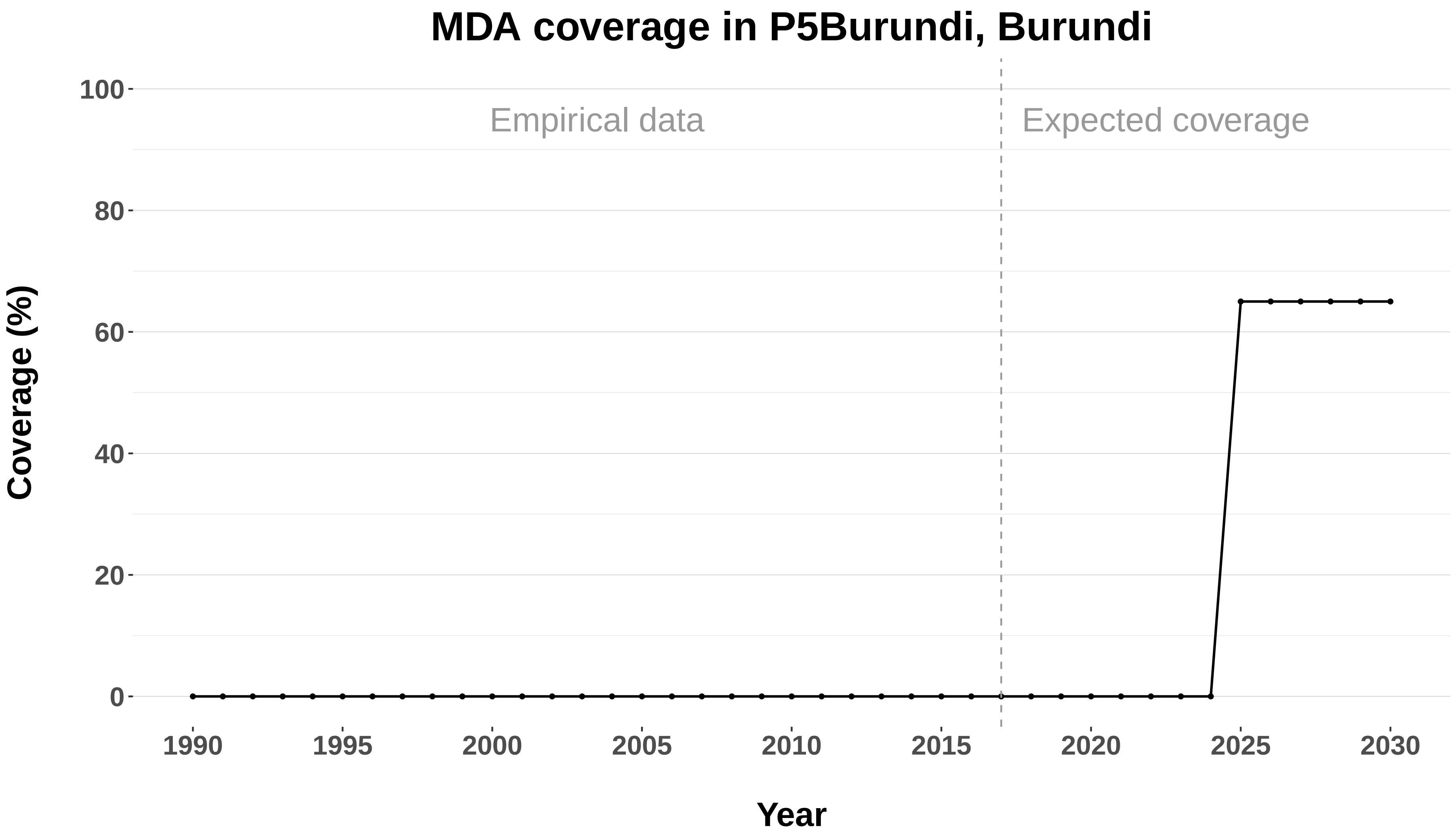

MDA coverage in P5Burundi, Burundi
Empirical data
Expected coverage
Coverage (%)
0
20
40
60
80
100
1990
1995
2000
2005
2010
2015
2020
2025
2030
Year

**Project, country:**	**Rutana, Burundi**
Bioclime:	
Endemicity level(s):	Hypoendemic
MDA start year:	2006
Mean MDA coverage per year:	71.1

	1990			2020			2030		
	N	%	DALYs	N	%	DALYs	N	%	DALYs
Total populaton at risk	171	100	_	387	100	_	508	100	_
**Infection:**									
Mf-positive	35	20.4	_	2	0.5	_	<0.5	<0.05	_
Mf-positive (age 5+)	35	20.4	_	2	0.5	_	<0.5	<0.05	_
Worm infected	48	28.1	_	20	5.2	_	1	0.1	_
**Clinical manifestations:**									
Palpable nodules	4	2.1	<0.5	<0.5	0.1	<0.5	<0.5	<0.05	<0.5
Reversible skin disease:									
Severe itch	4	2.4	1	<0.5	<0.05	<0.5	<0.5	<0.05	<0.5
Reactive skin disease	3	1.5	<0.5	<0.5	<0.05	<0.5	<0.5	<0.05	<0.5
**Reversible skin disease**	**7**	**3.9**	**1**	**<0.5**	**0.1**	**<0.5**	**<0.5**	**<0.05**	**<0.5**
Irreversible skin disease:									
Mild depigmentation	1	0.7	<0.5	1	0.3	<0.5	1	0.1	<0.5
Severe depigmentation	1	0.6	<0.5	1	0.2	<0.5	1	0.1	<0.5
Atrophy	<0.5	<0.05	<0.5	<0.5	<0.05	<0.5	<0.5	<0.05	<0.5
Hanging groin	<0.5	<0.05	<0.5	<0.5	<0.05	<0.5	<0.5	<0.05	<0.5
**Irreversible skin disease**	**2**	**1.3**	**<0.5**	**2**	**0.5**	**<0.5**	**1**	**0.3**	**<0.5**
**Total skin disease (OSD)**	**12**	**7.3**	**1**	**3**	**0.7**	**<0.5**	**1**	**0.3**	**<0.5**
Eye disease (OED):									
Visual impairment	<0.5	<0.05	<0.5	<0.5	<0.05	<0.5	<0.5	<0.05	<0.5
Blindness	<0.5	<0.05	<0.5	<0.5	<0.05	<0.5	<0.5	<0.05	<0.5
**Eye disease (OED)**	**<0.5**	**<0.05**	**<0.5**	**<0.5**	**<0.05**	**<0.5**	**<0.5**	**<0.05**	**<0.5**
**Total all manifestations**	**13**	**7.3**	**1**	**3**	**0.7**	**<0.5**	**1**	**0.3**	**<0.5**

Note 1: Population at risk, number of cases (N) and DALYs are presented in thousands.

Note 2: Total skin disease is defined as the sum of the number of cases with palpable nodules, reversible and irreversible skin disease.

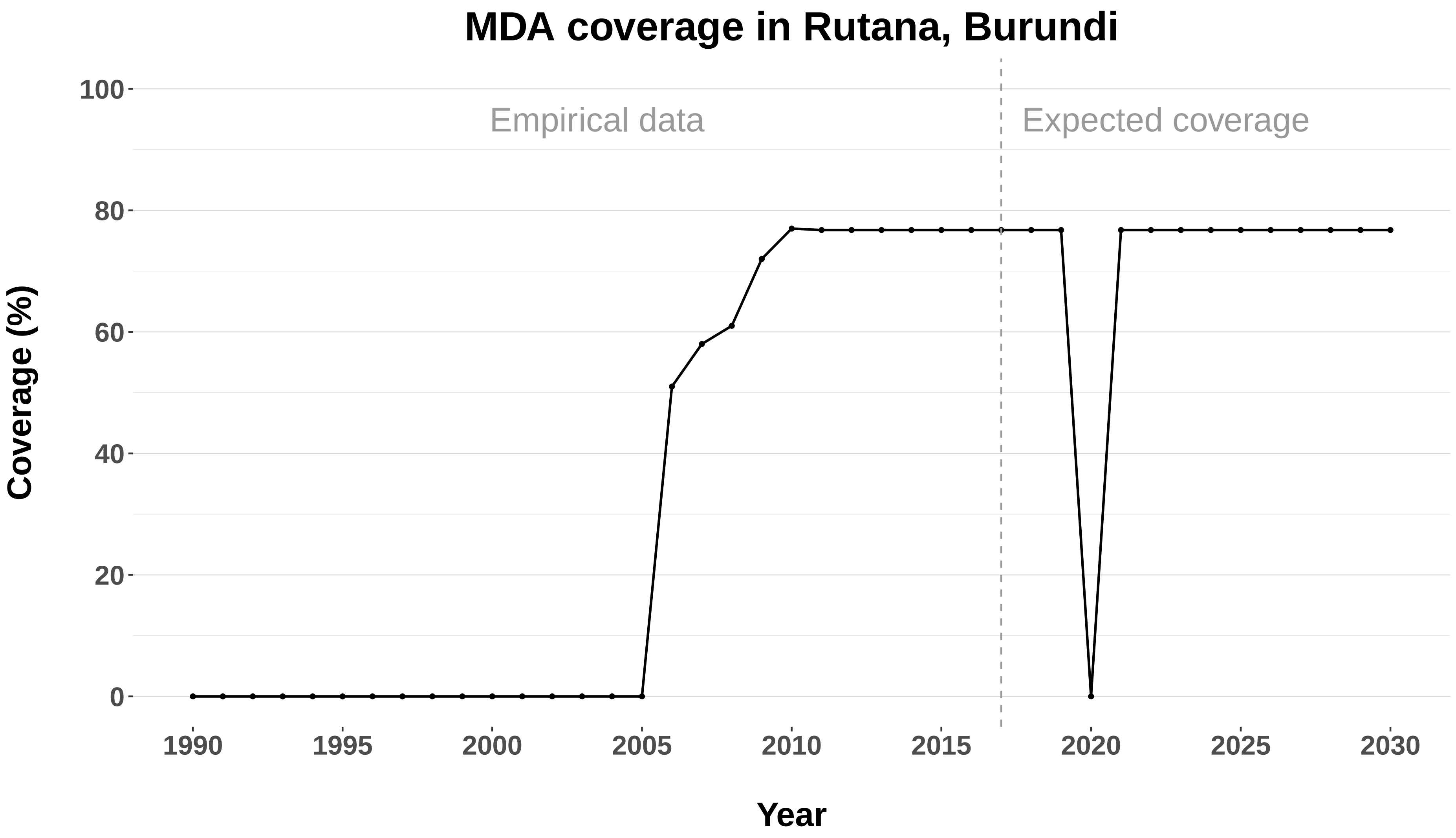

MDA coverage in Rutana, Burundi
Empirical data
Expected coverage
Coverage (%)
Year
0
20
40
60
80
100
1990
1995
2000
2005
2010
2015
2020
2025
2030

**Project, country:**	**Adamaoua 1, Cameroon**
Bioclime:	
Endemicity level(s):	Hyperendemic
MDA start year:	2004
Mean MDA coverage per year:	67.9

	1990			2020			2030		
	N	%	DALYs	N	%	DALYs	N	%	DALYs
Total populaton at risk	287	100	_	648	100	_	850	100	_
**Infection:**									
Mf-positive	88	30.6	_	9	1.4	_	<0.5	<0.05	_
Mf-positive (age 5+)	88	30.5	_	9	1.4	_	<0.5	<0.05	_
Worm infected	113	39.3	_	53	8.1	_	5	0.6	_
**Clinical manifestations:**									
Palpable nodules	22	7.8	<0.5	3	0.5	<0.5	<0.5	<0.05	<0.5
Reversible skin disease:									
Severe itch	13	4.4	2	1	0.1	<0.5	<0.5	<0.05	<0.5
Reactive skin disease	9	3.2	<0.5	<0.5	<0.05	<0.5	<0.5	<0.05	<0.5
**Reversible skin disease**	**22**	**7.5**	**3**	**1**	**0.1**	**<0.5**	**<0.5**	**<0.05**	**<0.5**
Irreversible skin disease:									
Mild depigmentation	3	1.0	<0.5	3	0.5	<0.5	2	0.2	<0.5
Severe depigmentation	3	1.0	<0.5	3	0.4	<0.5	2	0.2	<0.5
Atrophy	<0.5	<0.05	<0.5	<0.5	<0.05	<0.5	<0.5	<0.05	<0.5
Hanging groin	<0.5	<0.05	<0.5	<0.5	<0.05	<0.5	<0.5	<0.05	<0.5
**Irreversible skin disease**	**6**	**2.1**	**<0.5**	**6**	**0.9**	**<0.5**	**4**	**0.5**	**<0.5**
**Total skin disease (OSD)**	**50**	**17.4**	**3**	**10**	**1.5**	**<0.5**	**4**	**0.5**	**<0.5**
Eye disease (OED):									
Visual impairment	5	1.9	<0.5	6	0.9	<0.5	4	0.4	<0.5
Blindness	2	0.8	17	1	0.2	10	1	0.1	4
**Eye disease (OED)**	**8**	**2.7**	**17**	**7**	**1.1**	**10**	**4**	**0.5**	**4**
**Total all manifestations**	**58**	**20.1**	**20**	**17**	**2.6**	**10**	**8**	**1.0**	**4**

Note 1: Population at risk, number of cases (N) and DALYs are presented in thousands.

Note 2: Total skin disease is defined as the sum of the number of cases with palpable nodules, reversible and irreversible skin disease.

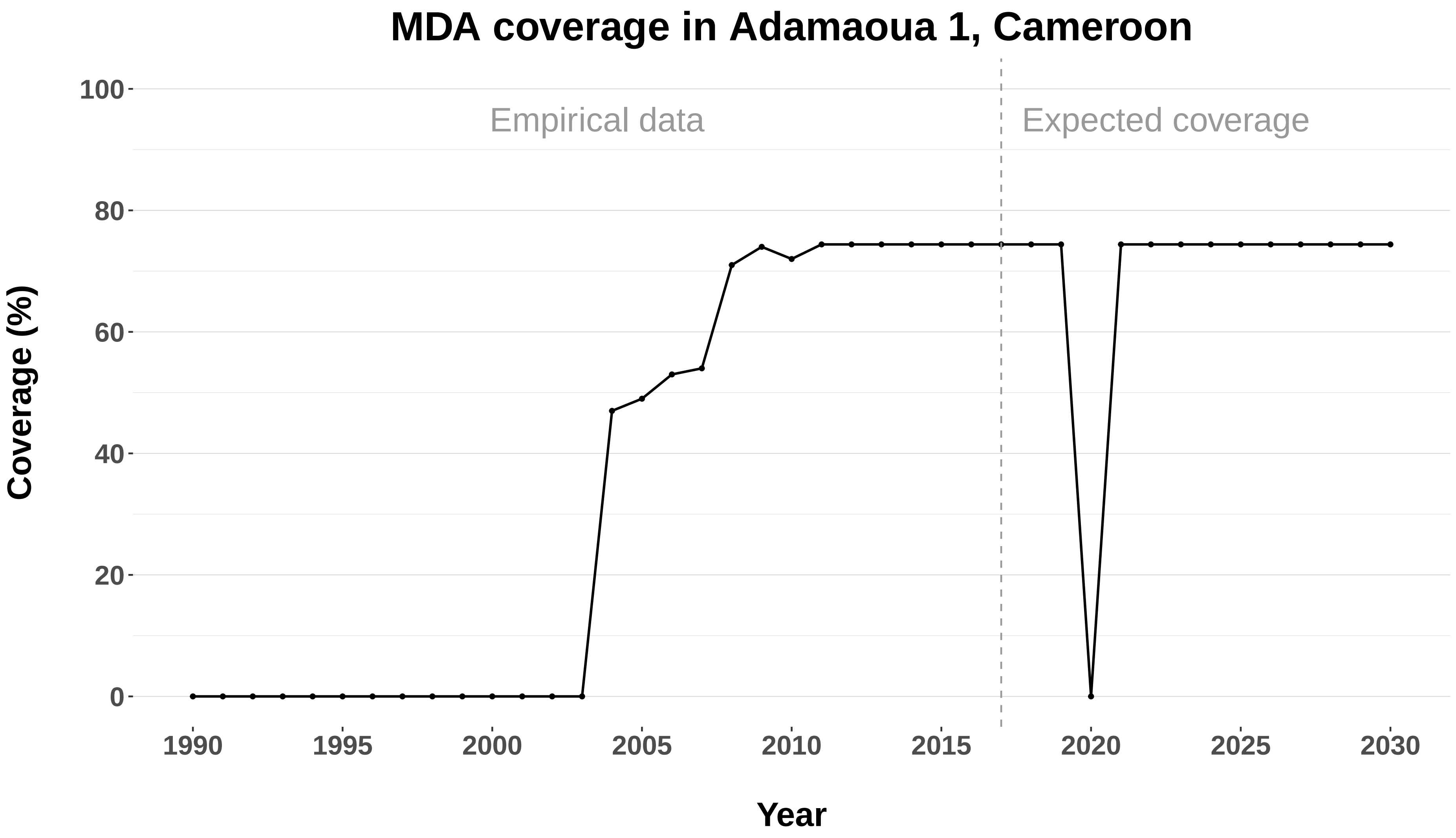

MDA coverage in Adamaoua 1, Cameroon
Empirical data
Expected coverage
Coverage (%)
100
80
60
40
20
0
1990
1995
2000
2005
2010
2015
2020
2025
2030
Year

**Project, country:**	**Adamaoua 2, Cameroon**
Bioclime:	
Endemicity level(s):	Hyperendemic
MDA start year:	1999
Mean MDA coverage per year:	65.8

	1990			2020			2030		
	N	%	DALYs	N	%	DALYs	N	%	DALYs
Total populaton at risk	261	100	_	589	100	_	772	100	_
**Infection:**									
Mf-positive	86	32.9	_	2	0.4	_	<0.5	<0.05	_
Mf-positive (age 5+)	86	32.9	_	2	0.4	_	<0.5	<0.05	_
Worm infected	110	42.2	_	25	4.3	_	1	0.1	_
**Clinical manifestations:**									
Palpable nodules	23	8.8	<0.5	1	0.1	<0.5	<0.5	<0.05	<0.5
Reversible skin disease:									
Severe itch	12	4.7	2	<0.5	<0.05	<0.5	<0.5	<0.05	<0.5
Reactive skin disease	9	3.3	<0.5	<0.5	<0.05	<0.5	<0.5	<0.05	<0.5
**Reversible skin disease**	**21**	**8.0**	**3**	**<0.5**	**<0.05**	**<0.5**	**<0.5**	**<0.05**	**<0.5**
Irreversible skin disease:									
Mild depigmentation	3	1.1	<0.5	2	0.4	<0.5	2	0.2	<0.5
Severe depigmentation	3	1.0	<0.5	2	0.4	<0.5	1	0.2	<0.5
Atrophy	<0.5	<0.05	<0.5	<0.5	<0.05	<0.5	<0.5	<0.05	<0.5
Hanging groin	<0.5	<0.05	<0.5	<0.5	<0.05	<0.5	<0.5	<0.05	<0.5
**Irreversible skin disease**	**6**	**2.1**	**<0.5**	**5**	**0.8**	**<0.5**	**3**	**0.4**	**<0.5**
**Total skin disease (OSD)**	**49**	**19.0**	**3**	**6**	**1.0**	**<0.5**	**3**	**0.4**	**<0.5**
Eye disease (OED):									
Visual impairment	5	2.0	<0.5	4	0.7	<0.5	3	0.4	<0.5
Blindness	2	0.8	15	1	0.1	6	<0.5	<0.05	2
**Eye disease (OED)**	**7**	**2.8**	**15**	**5**	**0.9**	**6**	**3**	**0.4**	**2**
**Total all manifestations**	**57**	**21.8**	**18**	**11**	**1.8**	**6**	**6**	**0.8**	**3**

Note 1: Population at risk, number of cases (N) and DALYs are presented in thousands.

Note 2: Total skin disease is defined as the sum of the number of cases with palpable nodules, reversible and irreversible skin disease.

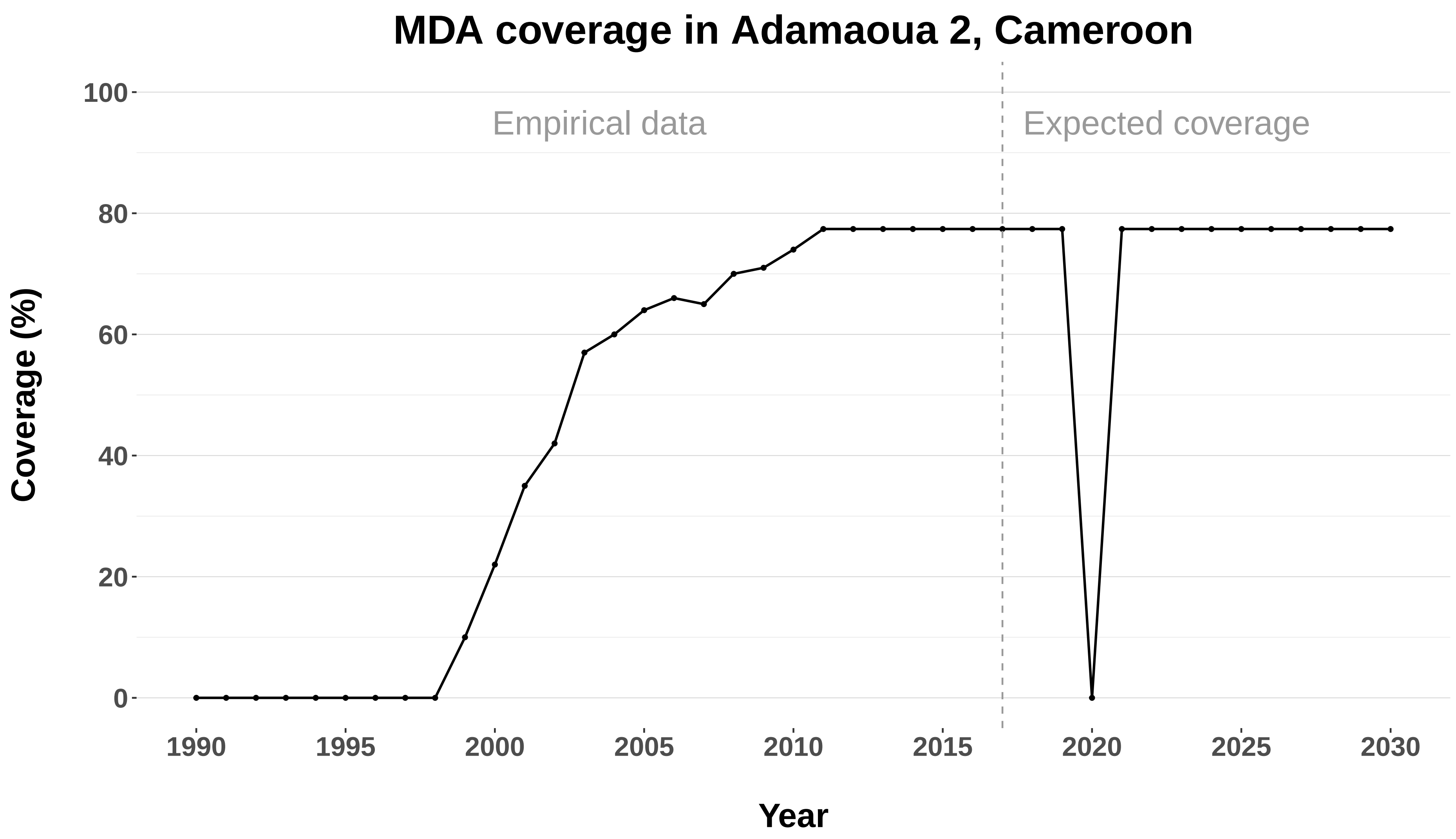
MDA coverage in Adamaoua 2, Cameroon
Empirical data
Expected coverage
Coverage (%)
100
80
60
40
20
0
1990
1995
2000
2005
2010
2015
2020
2025
2030
Year

**Project, country:**	**Centre 1, Cameroon**
Bioclime:	
Endemicity level(s):	Hyperendemic
MDA start year:	2000
Mean MDA coverage per year:	69.0

	1990			2020			2030		
	N	%	DALYs	N	%	DALYs	N	%	DALYs
Total populaton at risk	265	100	_	598	100	_	785	100	_
**Infection:**									
Mf-positive	136	51.2	_	14	2.4	_	1	0.1	_
Mf-positive (age 5+)	135	51.1	_	14	2.4	_	1	0.1	_
Worm infected	159	60.0	_	71	11.8	_	9	1.2	_
**Clinical manifestations:**									
Palpable nodules	61	23.0	1	5	0.9	<0.5	<0.5	<0.05	<0.5
Reversible skin disease:									
Severe itch	27	10.2	5	1	0.2	<0.5	<0.5	<0.05	<0.5
Reactive skin disease	22	8.2	1	<0.5	<0.05	<0.5	<0.5	<0.05	<0.5
**Reversible skin disease**	**49**	**18.4**	**6**	**1**	**0.2**	**<0.5**	**<0.5**	**<0.05**	**<0.5**
Irreversible skin disease:									
Mild depigmentation	5	2.1	<0.5	5	0.9	<0.5	4	0.5	<0.5
Severe depigmentation	8	2.9	<0.5	6	1.1	<0.5	4	0.5	<0.5
Atrophy	<0.5	0.1	<0.5	<0.5	<0.05	<0.5	<0.5	<0.05	<0.5
Hanging groin	<0.5	0.1	<0.5	<0.5	<0.05	<0.5	<0.5	<0.05	<0.5
**Irreversible skin disease**	**13**	**5.1**	**1**	**12**	**2.0**	**1**	**8**	**1.0**	**<0.5**
**Total skin disease (OSD)**	**123**	**46.4**	**7**	**18**	**3.0**	**1**	**8**	**1.0**	**<0.5**
Eye disease (OED):									
Visual impairment	<0.5	0.2	<0.5	<0.5	0.1	<0.5	<0.5	<0.05	<0.5
Blindness	1	0.2	4	<0.5	<0.05	1	<0.5	<0.05	<0.5
**Eye disease (OED)**	**1**	**0.4**	**4**	**1**	**0.1**	**1**	**<0.5**	**<0.05**	**<0.5**
**Total all manifestations**	**124**	**46.8**	**11**	**19**	**3.1**	**2**	**8**	**1.1**	**1**

Note 1: Population at risk, number of cases (N) and DALYs are presented in thousands.

Note 2: Total skin disease is defined as the sum of the number of cases with palpable nodules, reversible and irreversible skin disease.

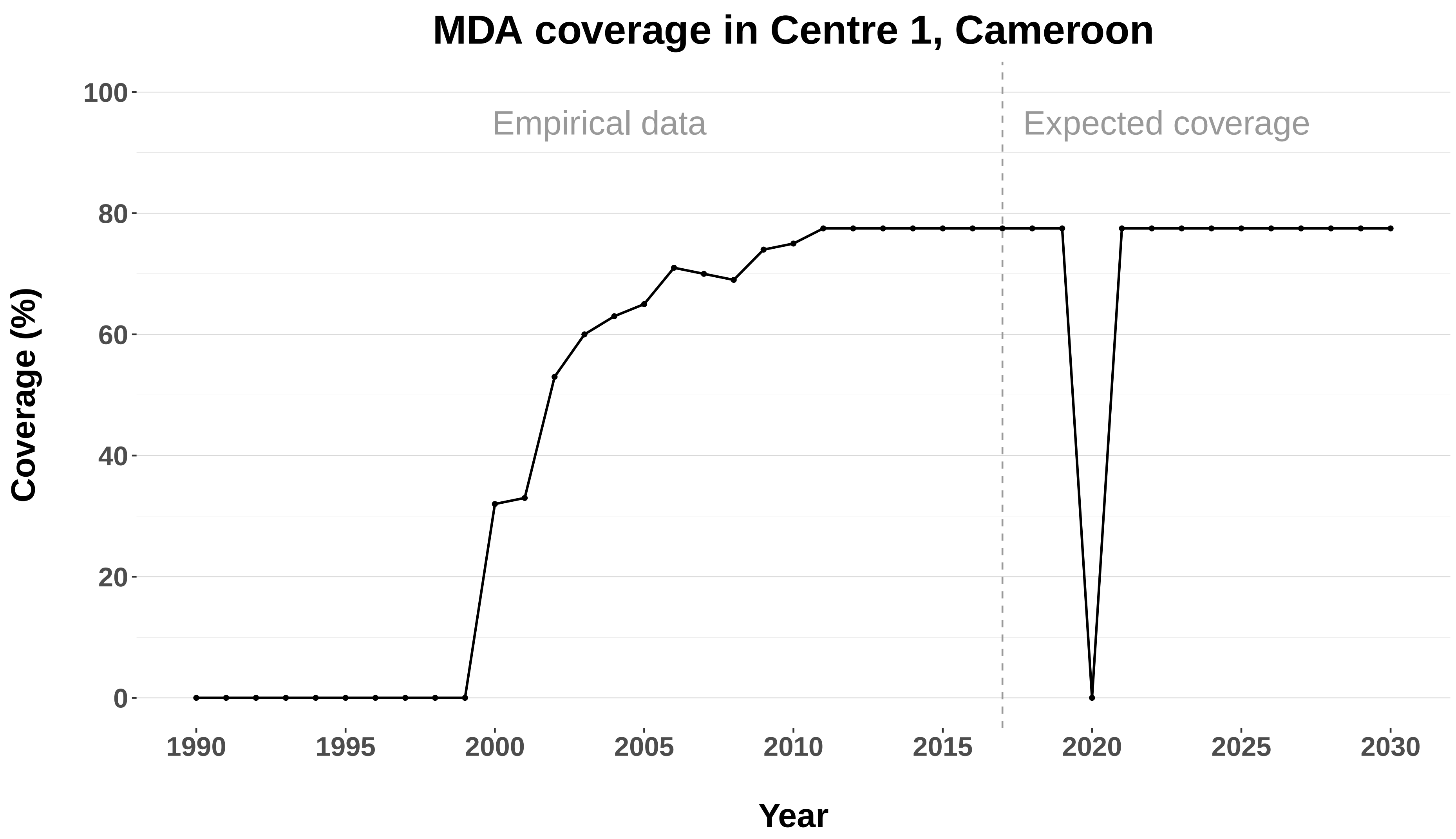

MDA coverage in Centre 1, Cameroon
Empirical data
Expected coverage
Coverage (%)
0
20
40
60
80
100
1990
1995
2000
2005
2010
2015
2020
2025
2030
Year

**Project, country:**	**Centre 2, Cameroon**
Bioclime:	
Endemicity level(s):	Hyperendemic
MDA start year:	2000
Mean MDA coverage per year:	64.1

	1990			2020			2030		
	N	%	DALYs	N	%	DALYs	N	%	DALYs
Total populaton at risk	62	100	_	141	100	_	185	100	_
**Infection:**									
Mf-positive	27	43.6	_	4	3.2	_	1	0.3	_
Mf-positive (age 5+)	27	43.5	_	4	3.2	_	1	0.3	_
Worm infected	33	52.2	_	18	12.7	_	3	1.9	_
**Clinical manifestations:**									
Palpable nodules	11	17.2	<0.5	2	1.2	<0.5	<0.5	0.1	<0.5
Reversible skin disease:									
Severe itch	5	8.1	1	<0.5	0.3	<0.5	<0.5	<0.05	<0.5
Reactive skin disease	4	6.4	<0.5	<0.5	<0.05	<0.5	<0.5	<0.05	<0.5
**Reversible skin disease**	**9**	**14.4**	**1**	**<0.5**	**0.3**	**<0.5**	**<0.5**	**<0.05**	**<0.5**
Irreversible skin disease:									
Mild depigmentation	1	1.7	<0.5	1	0.8	<0.5	1	0.4	<0.5
Severe depigmentation	1	2.2	<0.5	1	0.9	<0.5	1	0.5	<0.5
Atrophy	<0.5	<0.05	<0.5	<0.5	<0.05	<0.5	<0.5	<0.05	<0.5
Hanging groin	<0.5	0.1	<0.5	<0.5	<0.05	<0.5	<0.5	<0.05	<0.5
**Irreversible skin disease**	**3**	**4.1**	**<0.5**	**2**	**1.7**	**<0.5**	**2**	**0.9**	**<0.5**
**Total skin disease (OSD)**	**22**	**35.7**	**1**	**5**	**3.2**	**<0.5**	**2**	**1.0**	**<0.5**
Eye disease (OED):									
Visual impairment	<0.5	0.1	<0.5	<0.5	0.1	<0.5	<0.5	<0.05	<0.5
Blindness	<0.5	0.2	1	<0.5	<0.05	<0.5	<0.5	<0.05	<0.5
**Eye disease (OED)**	**<0.5**	**0.3**	**1**	**<0.5**	**0.1**	**<0.5**	**<0.5**	**<0.05**	**<0.5**
**Total all manifestations**	**22**	**36.0**	**2**	**5**	**3.3**	**1**	**2**	**1.0**	**<0.5**

Note 1: Population at risk, number of cases (N) and DALYs are presented in thousands.

Note 2: Total skin disease is defined as the sum of the number of cases with palpable nodules, reversible and irreversible skin disease.

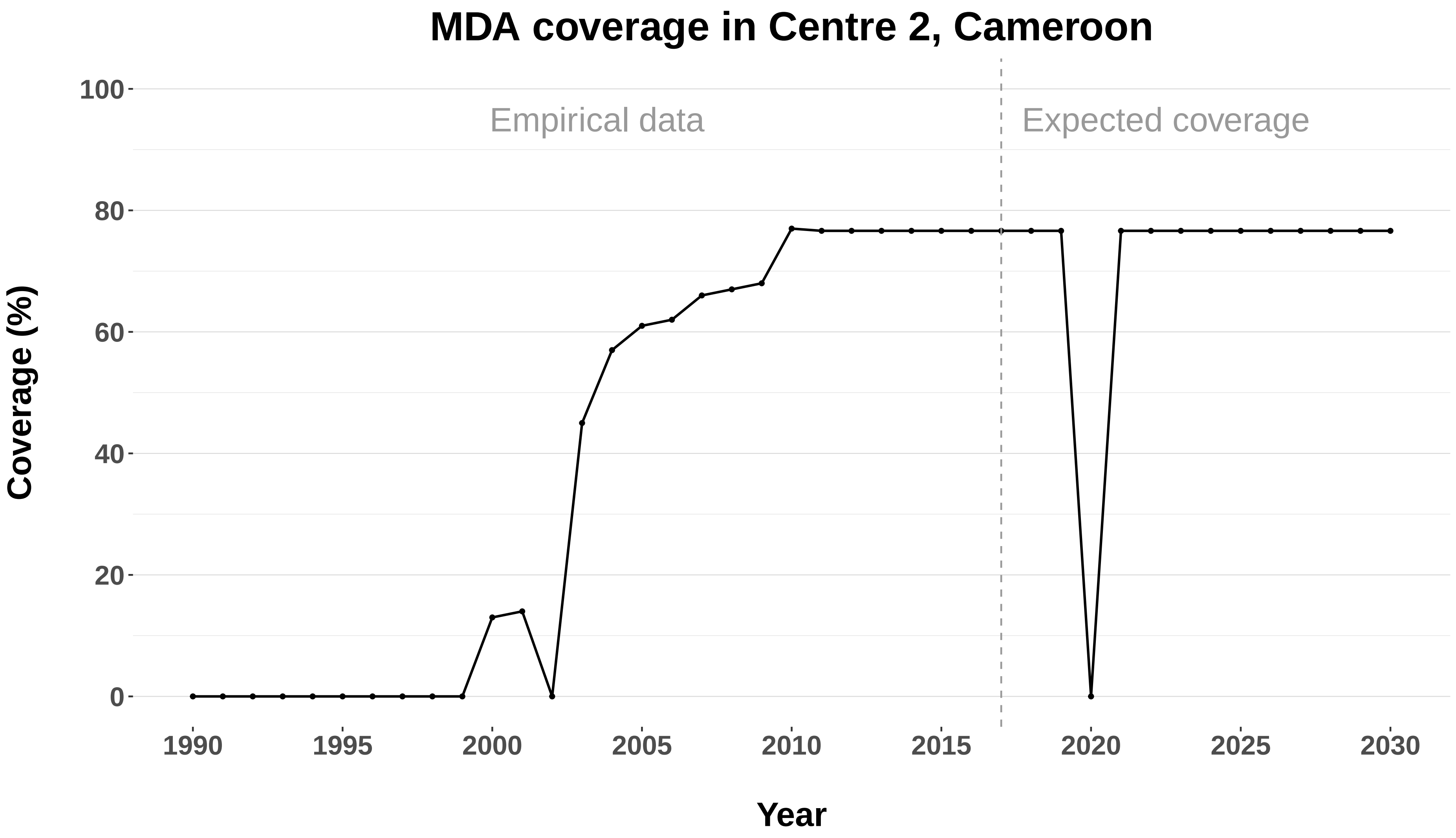
MDA coverage in Centre 2, Cameroon
Empirical data
Expected coverage
Coverage (%)
100
80
60
40
20
0
1990
1995
2000
2005
2010
2015
2020
2025
2030
Year

**Project, country:**	**Centre 3, Cameroon**
Bioclime:	
Endemicity level(s):	Hyperendemic
MDA start year:	1999
Mean MDA coverage per year:	67.4

	1990			2020			2030		
	N	%	DALYs	N	%	DALYs	N	%	DALYs
Total populaton at risk	201	100	_	455	100	_	596	100	_
**Infection:**									
Mf-positive	80	39.9	_	3	0.6	_	<0.5	<0.05	_
Mf-positive (age 5+)	80	39.9	_	3	0.6	_	<0.5	<0.05	_
Worm infected	99	48.9	_	26	5.7	_	1	0.2	_
**Clinical manifestations:**									
Palpable nodules	27	13.4	<0.5	1	0.2	<0.5	<0.5	<0.05	<0.5
Reversible skin disease:									
Severe itch	13	6.5	2	<0.5	0.1	<0.5	<0.5	<0.05	<0.5
Reactive skin disease	10	4.9	<0.5	<0.5	<0.05	<0.5	<0.5	<0.05	<0.5
**Reversible skin disease**	**23**	**11.4**	**3**	**<0.5**	**0.1**	**<0.5**	**<0.5**	**<0.05**	**<0.5**
Irreversible skin disease:									
Mild depigmentation	3	1.5	<0.5	3	0.6	<0.5	2	0.3	<0.5
Severe depigmentation	3	1.6	<0.5	3	0.6	<0.5	2	0.3	<0.5
Atrophy	<0.5	<0.05	<0.5	<0.5	<0.05	<0.5	<0.5	<0.05	<0.5
Hanging groin	<0.5	<0.05	<0.5	<0.5	<0.05	<0.5	<0.5	<0.05	<0.5
**Irreversible skin disease**	**6**	**3.1**	**<0.5**	**5**	**1.1**	**<0.5**	**3**	**0.6**	**<0.5**
**Total skin disease (OSD)**	**56**	**27.9**	**3**	**6**	**1.4**	**<0.5**	**3**	**0.6**	**<0.5**
Eye disease (OED):									
Visual impairment	<0.5	0.1	<0.5	<0.5	<0.05	<0.5	<0.5	<0.05	<0.5
Blindness	<0.5	0.1	1	<0.5	<0.05	<0.5	<0.5	<0.05	<0.5
**Eye disease (OED)**	**<0.5**	**0.1**	**1**	**<0.5**	**<0.05**	**<0.5**	**<0.5**	**<0.05**	**<0.5**
**Total all manifestations**	**56**	**28.0**	**4**	**7**	**1.5**	**<0.5**	**3**	**0.6**	**<0.5**

Note 1: Population at risk, number of cases (N) and DALYs are presented in thousands.

Note 2: Total skin disease is defined as the sum of the number of cases with palpable nodules, reversible and irreversible skin disease.

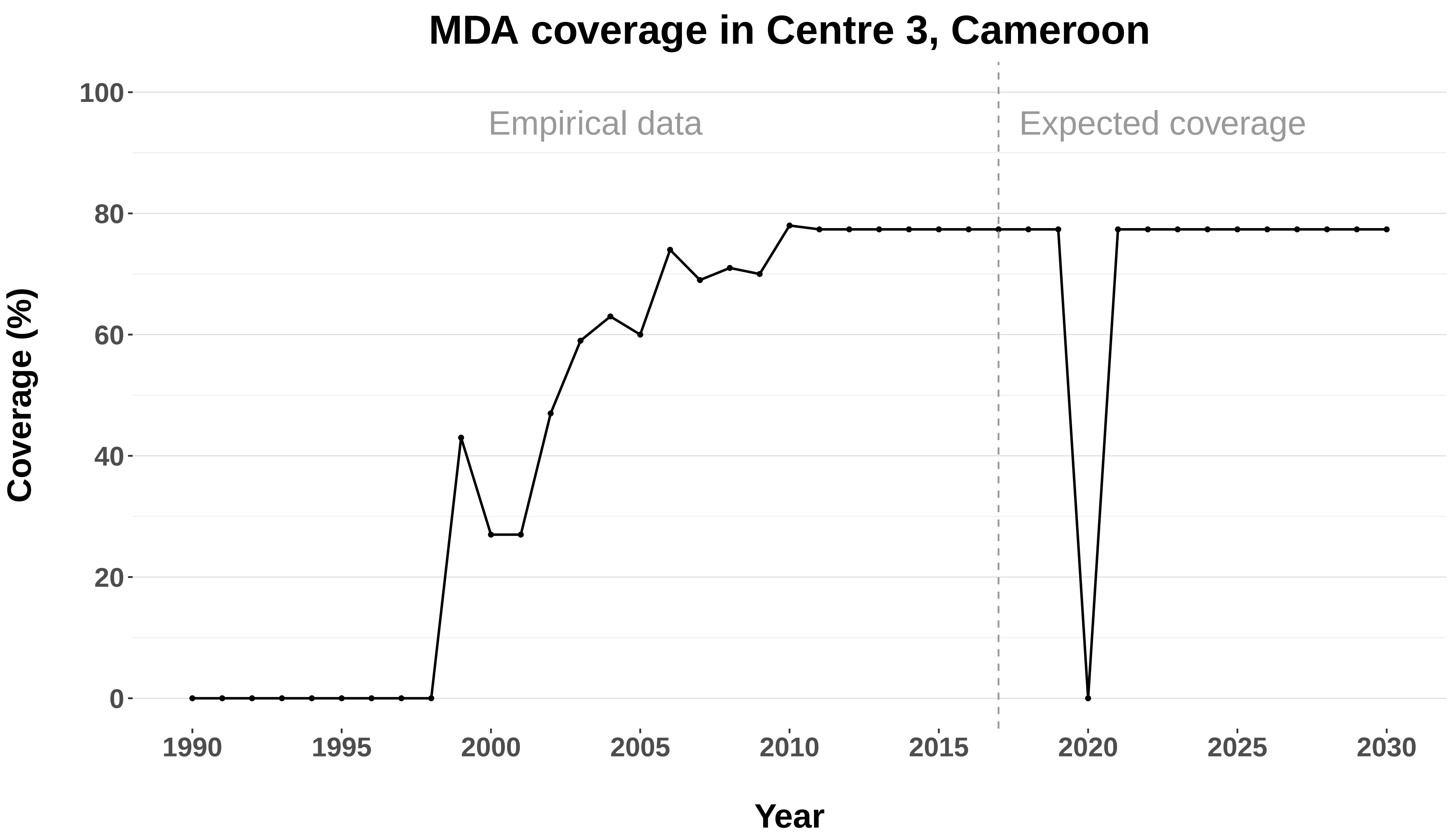
MDA coverage in Centre 3, Cameroon
Empirical data
Expected coverage
Coverage (%)
0
20
40
60
80
100
1990
1995
2000
2005
2010
2015
2020
2025
2030
Year

**Project, country:**	**East, Cameroon**
Bioclime:	
Endemicity level(s):	Hyperendemic
MDA start year:	2005
Mean MDA coverage per year:	74.7

	1990			2020			2030		
	N	%	DALYs	N	%	DALYs	N	%	DALYs
Total populaton at risk	74	100	_	166	100	_	218	100	_
**Infection:**									
Mf-positive	29	39.7	_	6	3.4	_	<0.5	0.2	_
Mf-positive (age 5+)	29	39.6	_	6	3.4	_	<0.5	0.2	_
Worm infected	35	48.1	_	24	14.2	_	4	1.6	_
**Clinical manifestations:**									
Palpable nodules	11	14.7	<0.5	3	1.6	<0.5	<0.5	0.1	<0.5
Reversible skin disease:									
Severe itch	5	7.2	1	1	0.4	<0.5	<0.5	<0.05	<0.5
Reactive skin disease	4	5.7	<0.5	<0.5	<0.05	<0.5	<0.5	<0.05	<0.5
**Reversible skin disease**	**10**	**13.0**	**1**	**1**	**0.4**	**<0.5**	**<0.5**	**<0.05**	**<0.5**
Irreversible skin disease:									
Mild depigmentation	1	1.6	<0.5	1	0.8	<0.5	1	0.4	<0.5
Severe depigmentation	1	2.0	<0.5	2	1.0	<0.5	1	0.5	<0.5
Atrophy	<0.5	<0.05	<0.5	<0.5	<0.05	<0.5	<0.5	<0.05	<0.5
Hanging groin	<0.5	0.1	<0.5	<0.5	<0.05	<0.5	<0.5	<0.05	<0.5
**Irreversible skin disease**	**3**	**3.6**	**<0.5**	**3**	**1.8**	**<0.5**	**2**	**0.9**	**<0.5**
**Total skin disease (OSD)**	**23**	**31.3**	**1**	**6**	**3.8**	**<0.5**	**2**	**1.0**	**<0.5**
Eye disease (OED):									
Visual impairment	<0.5	0.1	<0.5	<0.5	0.1	<0.5	<0.5	<0.05	<0.5
Blindness	<0.5	0.2	1	<0.5	<0.05	<0.5	<0.5	<0.05	<0.5
**Eye disease (OED)**	**<0.5**	**0.3**	**1**	**<0.5**	**0.1**	**<0.5**	**<0.5**	**<0.05**	**<0.5**
**Total all manifestations**	**23**	**31.6**	**2**	**6**	**3.9**	**1**	**2**	**1.1**	**<0.5**

Note 1: Population at risk, number of cases (N) and DALYs are presented in thousands.

Note 2: Total skin disease is defined as the sum of the number of cases with palpable nodules, reversible and irreversible skin disease.

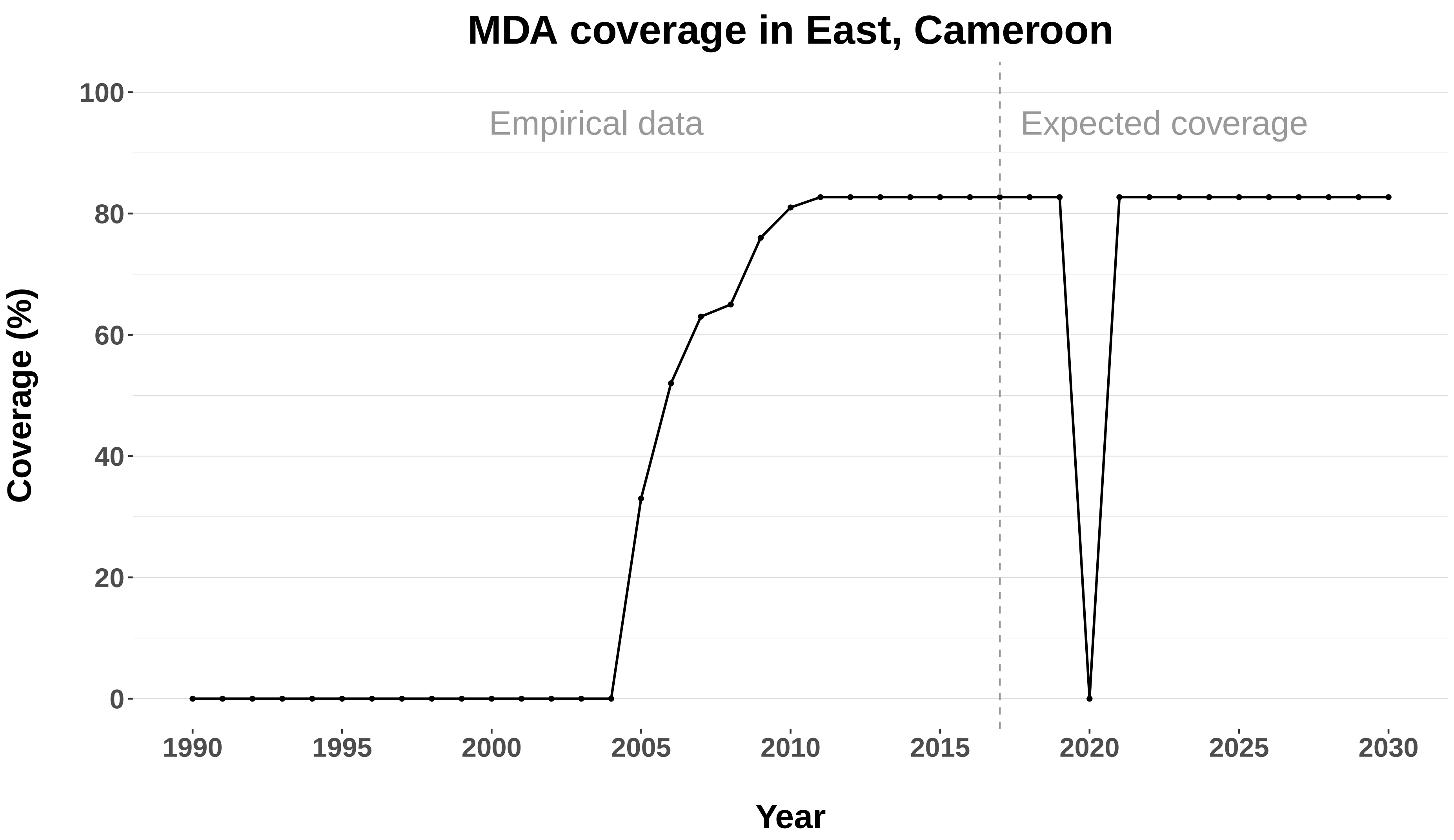
MDA coverage in East, Cameroon
Empirical data
Expected coverage
Coverage (%)
100
80
60
40
20
0
1990
1995
2000
2005
2010
2015
2020
2025
2030
Year

**Project, country:**	**Far North, Cameroon**
Bioclime:	
Endemicity level(s):	Mesoendemic
MDA start year:	2005
Mean MDA coverage per year:	74.5

	1990			2020			2030		
	N	%	DALYs	N	%	DALYs	N	%	DALYs
Total populaton at risk	174	100	_	393	100	_	515	100	_
**Infection:**									
Mf-positive	33	18.8	_	1	0.2	_	<0.5	<0.05	_
Mf-positive (age 5+)	33	18.8	_	1	0.2	_	<0.5	<0.05	_
Worm infected	47	26.8	_	12	2.9	_	<0.5	<0.05	_
**Clinical manifestations:**									
Palpable nodules	3	1.8	<0.5	<0.5	<0.05	<0.5	<0.5	<0.05	<0.5
Reversible skin disease:									
Severe itch	4	2.1	1	<0.5	<0.05	<0.5	<0.5	<0.05	<0.5
Reactive skin disease	2	1.4	<0.5	<0.5	<0.05	<0.5	<0.5	<0.05	<0.5
**Reversible skin disease**	**6**	**3.5**	**1**	**<0.5**	**<0.05**	**<0.5**	**<0.5**	**<0.05**	**<0.5**
Irreversible skin disease:									
Mild depigmentation	1	0.6	<0.5	1	0.2	<0.5	1	0.1	<0.5
Severe depigmentation	1	0.5	<0.5	1	0.2	<0.5	<0.5	0.1	<0.5
Atrophy	<0.5	<0.05	<0.5	<0.5	<0.05	<0.5	<0.5	<0.05	<0.5
Hanging groin	<0.5	<0.05	<0.5	<0.5	<0.05	<0.5	<0.5	<0.05	<0.5
**Irreversible skin disease**	**2**	**1.0**	**<0.5**	**2**	**0.4**	**<0.5**	**1**	**0.2**	**<0.5**
**Total skin disease (OSD)**	**11**	**6.4**	**1**	**2**	**0.4**	**<0.5**	**1**	**0.2**	**<0.5**
Eye disease (OED):									
Visual impairment	2	1.0	<0.5	2	0.4	<0.5	1	0.2	<0.5
Blindness	<0.5	0.2	3	<0.5	0.1	1	<0.5	<0.05	<0.5
**Eye disease (OED)**	**2**	**1.2**	**3**	**2**	**0.4**	**1**	**1**	**0.2**	**1**
**Total all manifestations**	**13**	**7.6**	**4**	**3**	**0.9**	**2**	**2**	**0.4**	**1**

Note 1: Population at risk, number of cases (N) and DALYs are presented in thousands.

Note 2: Total skin disease is defined as the sum of the number of cases with palpable nodules, reversible and irreversible skin disease.

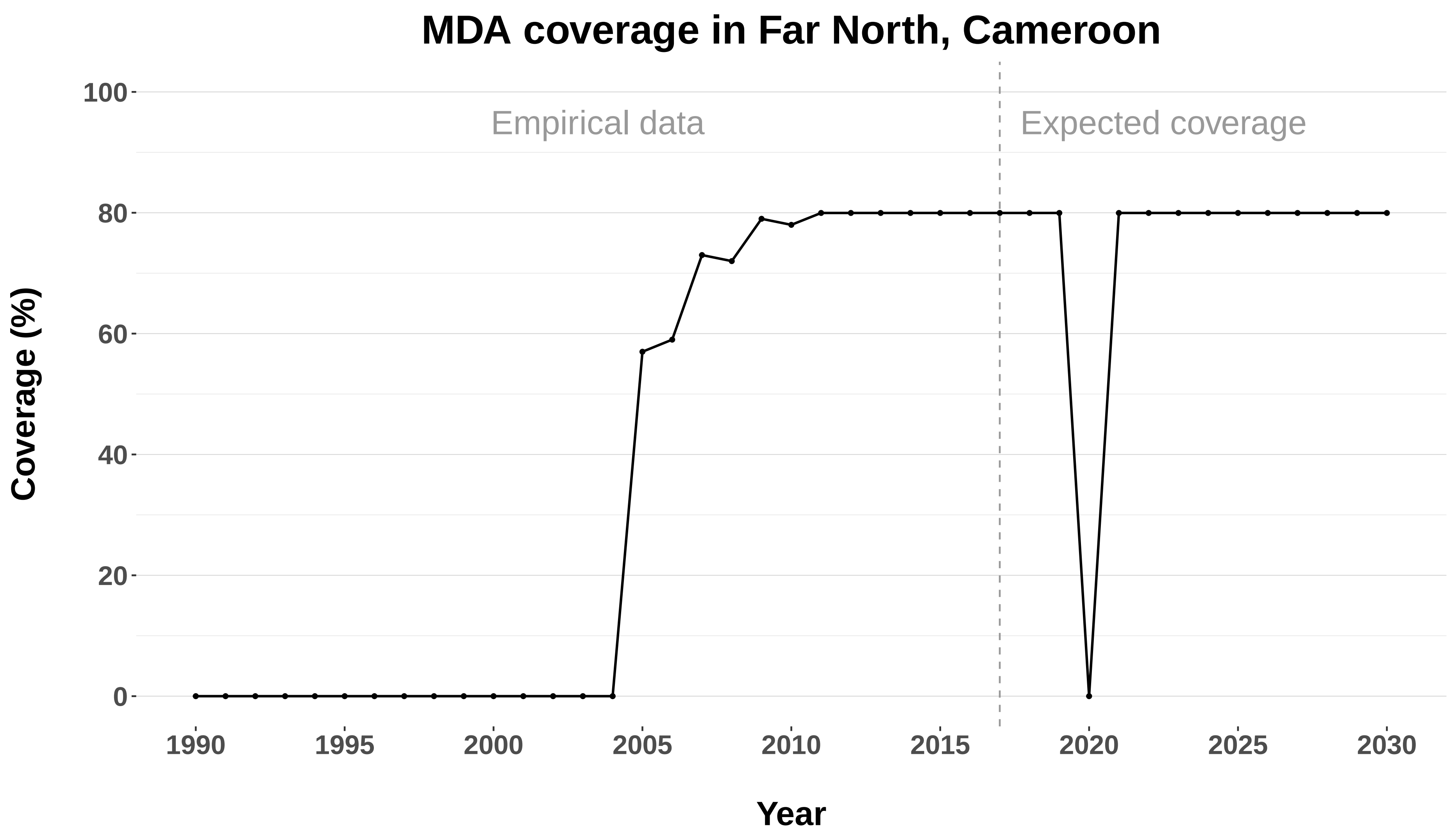
MDA coverage in Far North, Cameroon
Empirical data
Expected coverage
Coverage (%)
100
80
60
40
20
0
1990
1995
2000
2005
2010
2015
2020
2025
2030
Year

**Project, country:**	**Littoral 1, Cameroon**
Bioclime:	
Endemicity level(s):	Hyperendemic
MDA start year:	2006
Mean MDA coverage per year:	63.1

	1990			2020			2030		
	N	%	DALYs	N	%	DALYs	N	%	DALYs
Total populaton at risk	174	100	_	393	100	_	515	100	_
**Infection:**									
Mf-positive	92	52.6	_	49	12.5	_	8	1.5	_
Mf-positive (age 5+)	91	52.5	_	49	12.5	_	8	1.5	_
Worm infected	106	61.2	_	118	30.1	_	41	7.9	_
**Clinical manifestations:**									
Palpable nodules	42	24.0	<0.5	18	4.7	<0.5	2	0.4	<0.5
Reversible skin disease:									
Severe itch	18	10.6	3	4	1.1	1	<0.5	0.1	<0.5
Reactive skin disease	15	8.6	1	1	0.2	<0.5	<0.5	<0.05	<0.5
**Reversible skin disease**	**33**	**19.2**	**4**	**5**	**1.3**	**1**	**<0.5**	**0.1**	**<0.5**
Irreversible skin disease:									
Mild depigmentation	4	2.1	<0.5	5	1.2	<0.5	4	0.7	<0.5
Severe depigmentation	5	3.0	<0.5	6	1.5	<0.5	4	0.8	<0.5
Atrophy	<0.5	<0.05	<0.5	<0.5	<0.05	<0.5	<0.5	<0.05	<0.5
Hanging groin	<0.5	0.1	<0.5	<0.5	<0.05	<0.5	<0.5	<0.05	<0.5
**Irreversible skin disease**	**9**	**5.3**	**<0.5**	**11**	**2.8**	**<0.5**	**8**	**1.5**	**<0.5**
**Total skin disease (OSD)**	**84**	**48.5**	**5**	**34**	**8.8**	**1**	**11**	**2.1**	**<0.5**
Eye disease (OED):									
Visual impairment	<0.5	0.2	<0.5	<0.5	0.1	<0.5	<0.5	<0.05	<0.5
Blindness	<0.5	0.2	3	<0.5	0.1	2	<0.5	<0.05	1
**Eye disease (OED)**	**1**	**0.4**	**3**	**1**	**0.1**	**2**	**<0.5**	**0.1**	**1**
**Total all manifestations**	**85**	**48.9**	**8**	**35**	**8.9**	**3**	**11**	**2.1**	**1**

Note 1: Population at risk, number of cases (N) and DALYs are presented in thousands.

Note 2: Total skin disease is defined as the sum of the number of cases with palpable nodules, reversible and irreversible skin disease.

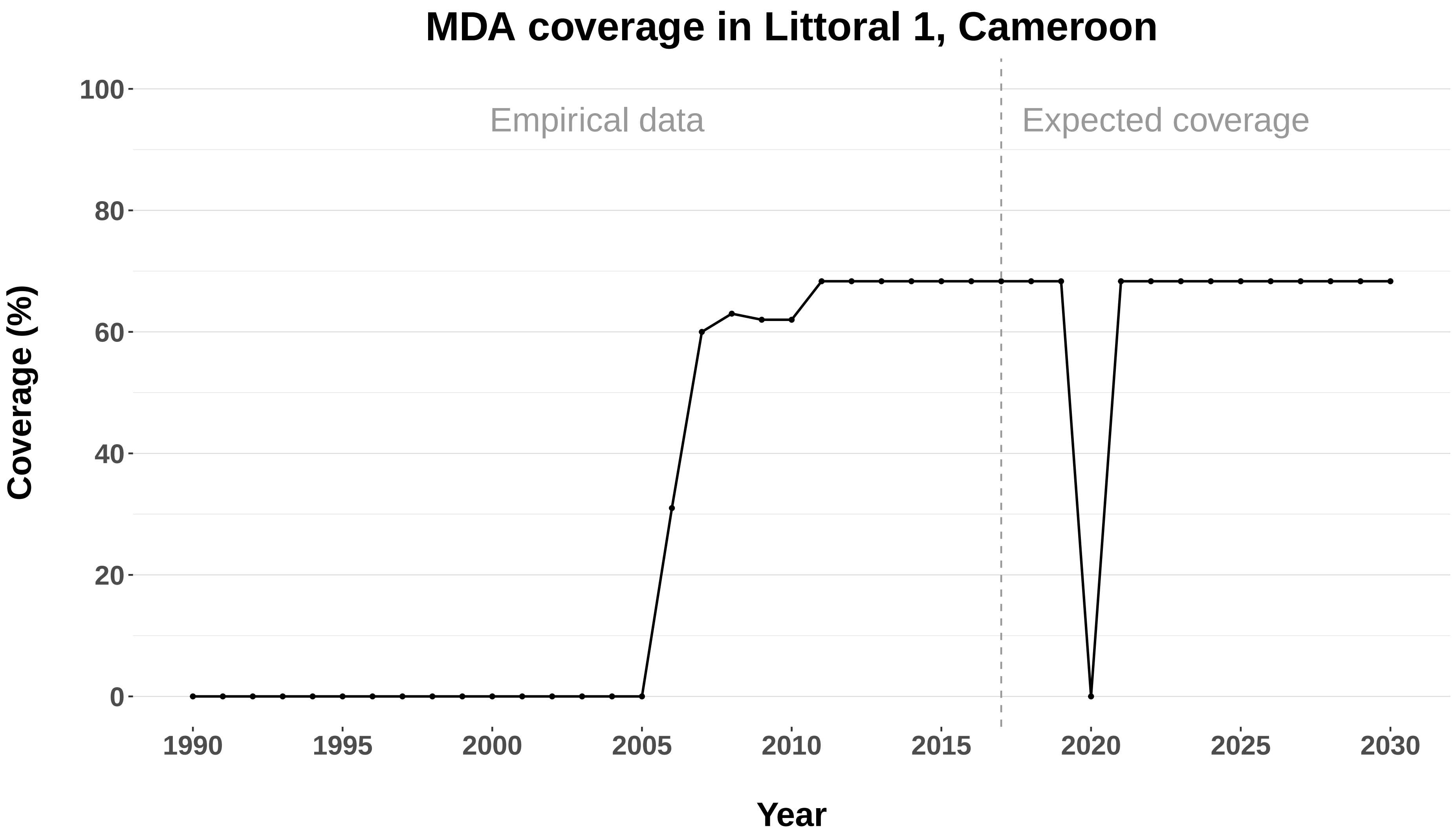
MDA coverage in Littoral 1, Cameroon
Empirical data
Expected coverage
Coverage (%)
100
80
60
40
20
0
1990
1995
2000
2005
2010
2015
2020
2025
2030
Year

**Project, country:**	**Littoral 2, Cameroon**
Bioclime:	
Endemicity level(s):	Hyperendemic
MDA start year:	1999
Mean MDA coverage per year:	67.2

	1990			2020			2030		
	N	%	DALYs	N	%	DALYs	N	%	DALYs
Total populaton at risk	93	100	_	209	100	_	275	100	_
**Infection:**									
Mf-positive	56	60.4	_	17	7.9	_	3	1.0	_
Mf-positive (age 5+)	56	60.1	_	17	7.9	_	3	1.0	_
Worm infected	63	68.0	_	47	22.6	_	16	6.0	_
**Clinical manifestations:**									
Palpable nodules	31	33.3	<0.5	7	3.2	<0.5	1	0.3	<0.5
Reversible skin disease:									
Severe itch	14	14.6	2	1	0.4	<0.5	<0.5	<0.05	<0.5
Reactive skin disease	12	12.7	1	<0.5	<0.05	<0.5	<0.5	<0.05	<0.5
**Reversible skin disease**	**25**	**27.3**	**3**	**1**	**0.5**	**<0.5**	**<0.5**	**<0.05**	**<0.5**
Irreversible skin disease:									
Mild depigmentation	3	2.7	<0.5	2	1.2	<0.5	2	0.7	<0.5
Severe depigmentation	4	4.6	<0.5	4	1.7	<0.5	2	0.9	<0.5
Atrophy	<0.5	0.1	<0.5	<0.5	<0.05	<0.5	<0.5	<0.05	<0.5
Hanging groin	<0.5	0.3	<0.5	<0.5	0.1	<0.5	<0.5	<0.05	<0.5
**Irreversible skin disease**	**7**	**7.9**	**<0.5**	**6**	**3.0**	**<0.5**	**4**	**1.6**	**<0.5**
**Total skin disease (OSD)**	**64**	**68.5**	**4**	**14**	**6.6**	**1**	**5**	**1.9**	**<0.5**
Eye disease (OED):									
Visual impairment	<0.5	0.4	<0.5	<0.5	0.1	<0.5	<0.5	0.1	<0.5
Blindness	1	0.6	4	<0.5	0.1	2	<0.5	<0.05	1
**Eye disease (OED)**	**1**	**1.0**	**4**	**<0.5**	**0.2**	**2**	**<0.5**	**0.1**	**1**
**Total all manifestations**	**65**	**69.5**	**8**	**14**	**6.9**	**2**	**6**	**2.0**	**1**

Note 1: Population at risk, number of cases (N) and DALYs are presented in thousands.

Note 2: Total skin disease is defined as the sum of the number of cases with palpable nodules, reversible and irreversible skin disease.

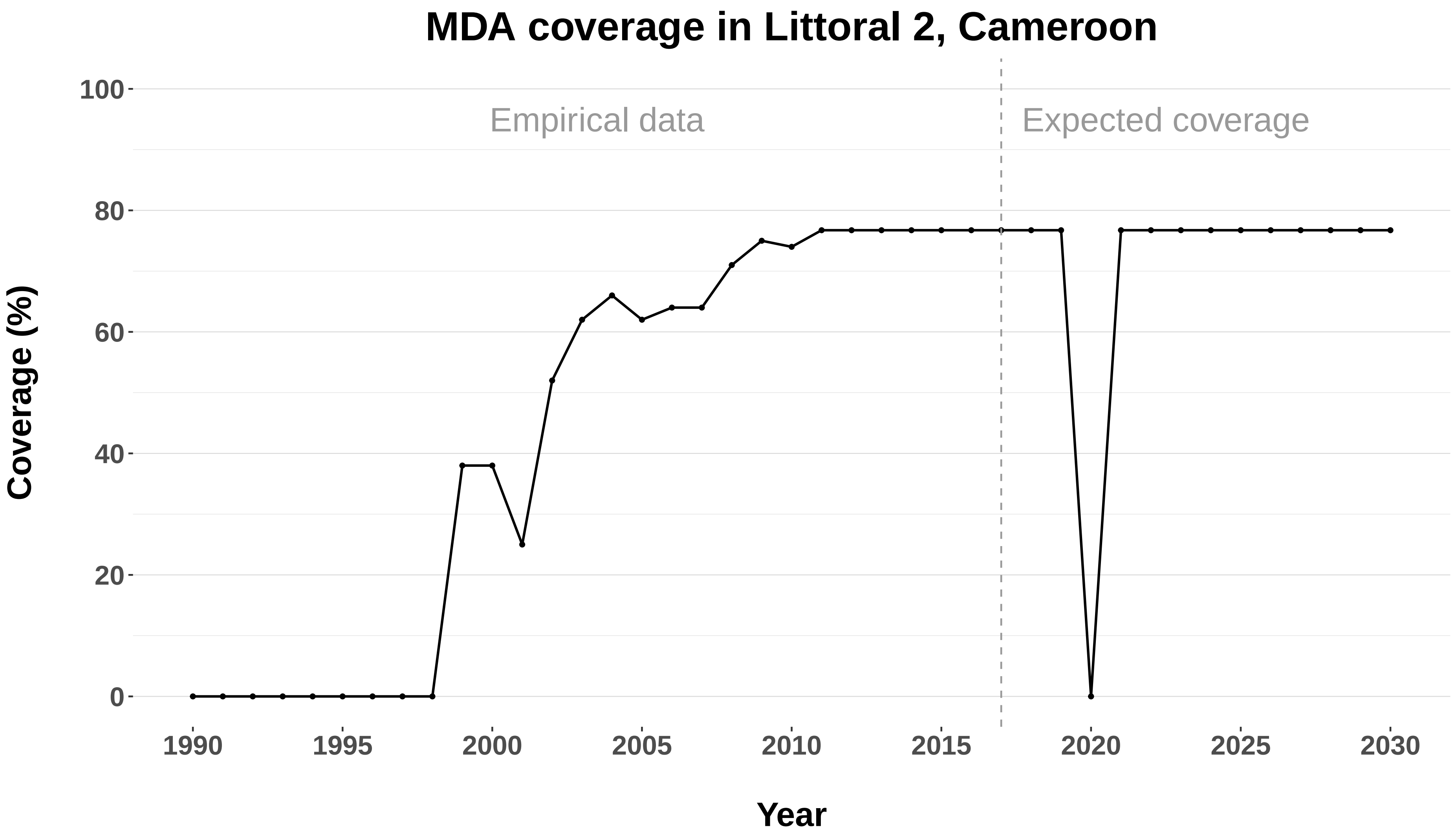
MDA coverage in Littoral 2, Cameroon
Empirical data
Expected coverage
Coverage (%)
100
80
60
40
20
0
1990
1995
2000
2005
2010
2015
2020
2025
2030
Year

**Project, country:**	**Northern, Cameroon**
Bioclime:	
Endemicity level(s):	Hyperendemic
MDA start year:	1999
Mean MDA coverage per year:	69.5

	1990			2020			2030		
	N	%	DALYs	N	%	DALYs	N	%	DALYs
Total populaton at risk	390	100	_	881	100	_	1,155	100	_
**Infection:**									
Mf-positive	162	41.4	_	9	1.0	_	<0.5	<0.05	_
Mf-positive (age 5+)	161	41.3	_	9	1.0	_	<0.5	<0.05	_
Worm infected	198	50.7	_	56	6.3	_	6	0.5	_
**Clinical manifestations:**									
Palpable nodules	61	15.5	1	4	0.4	<0.5	<0.5	<0.05	<0.5
Reversible skin disease:									
Severe itch	28	7.2	5	1	0.1	<0.5	<0.5	<0.05	<0.5
Reactive skin disease	22	5.6	1	<0.5	<0.05	<0.5	<0.5	<0.05	<0.5
**Reversible skin disease**	**50**	**12.8**	**6**	**1**	**0.1**	**<0.5**	**<0.5**	**<0.05**	**<0.5**
Irreversible skin disease:									
Mild depigmentation	6	1.5	<0.5	5	0.6	<0.5	3	0.3	<0.5
Severe depigmentation	7	1.8	<0.5	5	0.6	<0.5	3	0.3	<0.5
Atrophy	<0.5	<0.05	<0.5	<0.5	<0.05	<0.5	<0.5	<0.05	<0.5
Hanging groin	<0.5	<0.05	<0.5	<0.5	<0.05	<0.5	<0.5	<0.05	<0.5
**Irreversible skin disease**	**13**	**3.4**	**1**	**10**	**1.2**	**<0.5**	**7**	**0.6**	**<0.5**
**Total skin disease (OSD)**	**124**	**31.7**	**7**	**15**	**1.7**	**1**	**7**	**0.6**	**<0.5**
Eye disease (OED):									
Visual impairment	12	3.1	<0.5	10	1.1	<0.5	7	0.6	<0.5
Blindness	7	1.7	53	3	0.3	22	1	0.1	9
**Eye disease (OED)**	**19**	**4.8**	**54**	**13**	**1.4**	**22**	**8**	**0.7**	**10**
**Total all manifestations**	**142**	**36.5**	**61**	**27**	**3.1**	**23**	**15**	**1.3**	**10**

Note 1: Population at risk, number of cases (N) and DALYs are presented in thousands.

Note 2: Total skin disease is defined as the sum of the number of cases with palpable nodules, reversible and irreversible skin disease.

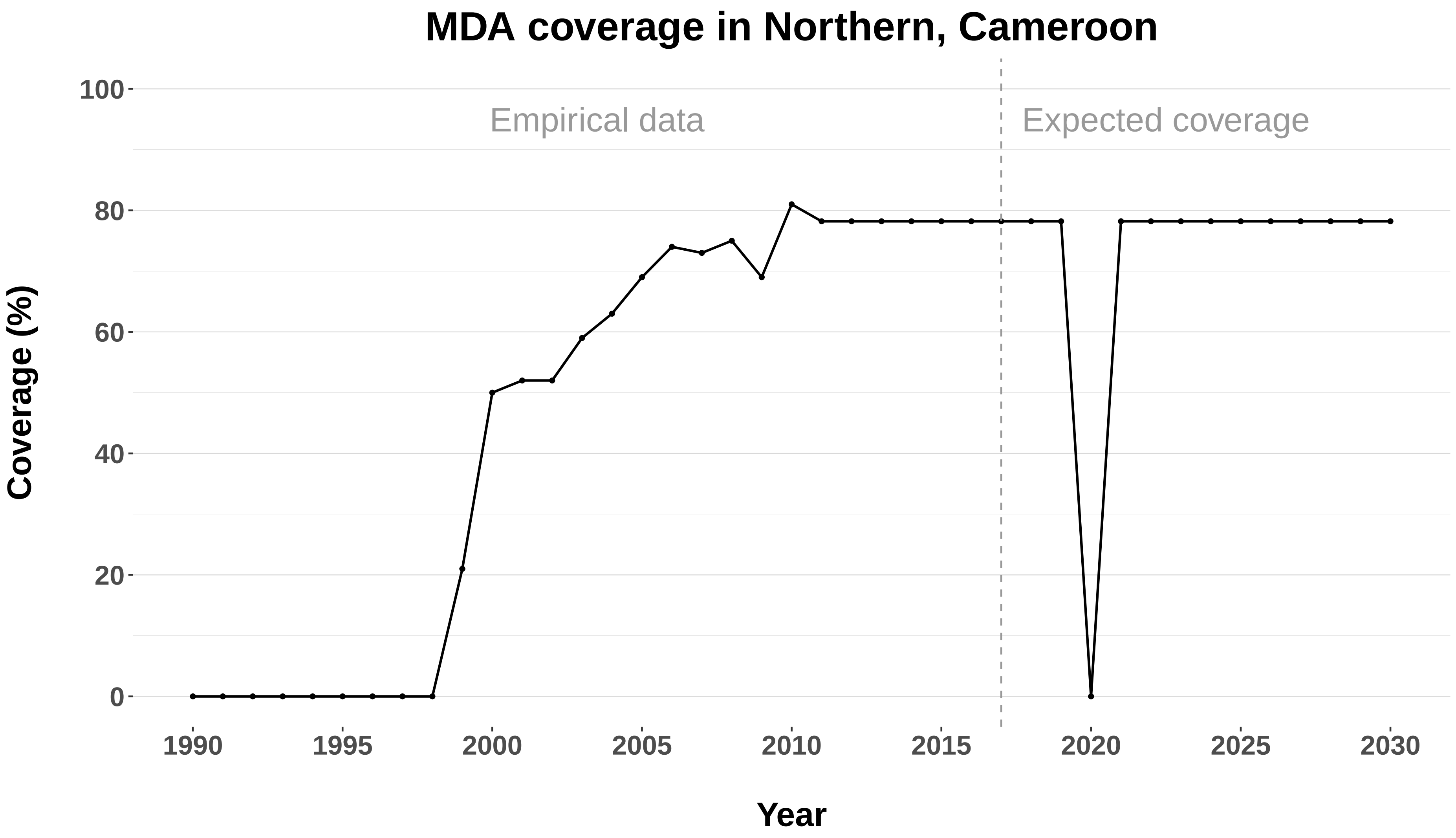
MDA coverage in Northern, Cameroon
Empirical data
Expected coverage
Coverage (%)
100
80
60
40
20
0
1990
1995
2000
2005
2010
2015
2020
2025
2030
Year

**Project, country:**	**Northwest, Cameroon**
Bioclime:	
Endemicity level(s):	Hyperendemic
MDA start year:	2004
Mean MDA coverage per year:	72.5

	1990			2020			2030		
	N	%	DALYs	N	%	DALYs	N	%	DALYs
Total populaton at risk	507	100	_	1,144	100	_	1,501	100	_
**Infection:**									
Mf-positive	223	44.1	_	18	1.6	_	1	0.1	_
Mf-positive (age 5+)	223	44.0	_	18	1.6	_	1	0.1	_
Worm infected	275	54.2	_	120	10.5	_	10	0.7	_
**Clinical manifestations:**									
Palpable nodules	78	15.5	1	7	0.6	<0.5	<0.5	<0.05	<0.5
Reversible skin disease:									
Severe itch	36	7.0	6	2	0.1	<0.5	<0.5	<0.05	<0.5
Reactive skin disease	26	5.2	1	<0.5	<0.05	<0.5	<0.5	<0.05	<0.5
**Reversible skin disease**	**62**	**12.2**	**8**	**2**	**0.1**	**<0.5**	**<0.5**	**<0.05**	**<0.5**
Irreversible skin disease:									
Mild depigmentation	8	1.5	<0.5	8	0.7	<0.5	6	0.4	<0.5
Severe depigmentation	8	1.7	1	8	0.7	1	5	0.3	<0.5
Atrophy	<0.5	<0.05	<0.5	<0.5	<0.05	<0.5	<0.5	<0.05	<0.5
Hanging groin	<0.5	<0.05	<0.5	<0.5	<0.05	<0.5	<0.5	<0.05	<0.5
**Irreversible skin disease**	**17**	**3.3**	**1**	**17**	**1.4**	**1**	**11**	**0.7**	**<0.5**
**Total skin disease (OSD)**	**157**	**30.9**	**9**	**26**	**2.2**	**1**	**11**	**0.8**	**<0.5**
Eye disease (OED):									
Visual impairment	<0.5	0.1	<0.5	<0.5	<0.05	<0.5	<0.5	<0.05	<0.5
Blindness	<0.5	0.1	2	<0.5	<0.05	1	<0.5	<0.05	<0.5
**Eye disease (OED)**	**1**	**0.1**	**2**	**<0.5**	**<0.05**	**1**	**<0.5**	**<0.05**	**<0.5**
**Total all manifestations**	**157**	**31.1**	**12**	**26**	**2.3**	**2**	**12**	**0.8**	**1**

Note 1: Population at risk, number of cases (N) and DALYs are presented in thousands.

Note 2: Total skin disease is defined as the sum of the number of cases with palpable nodules, reversible and irreversible skin disease.

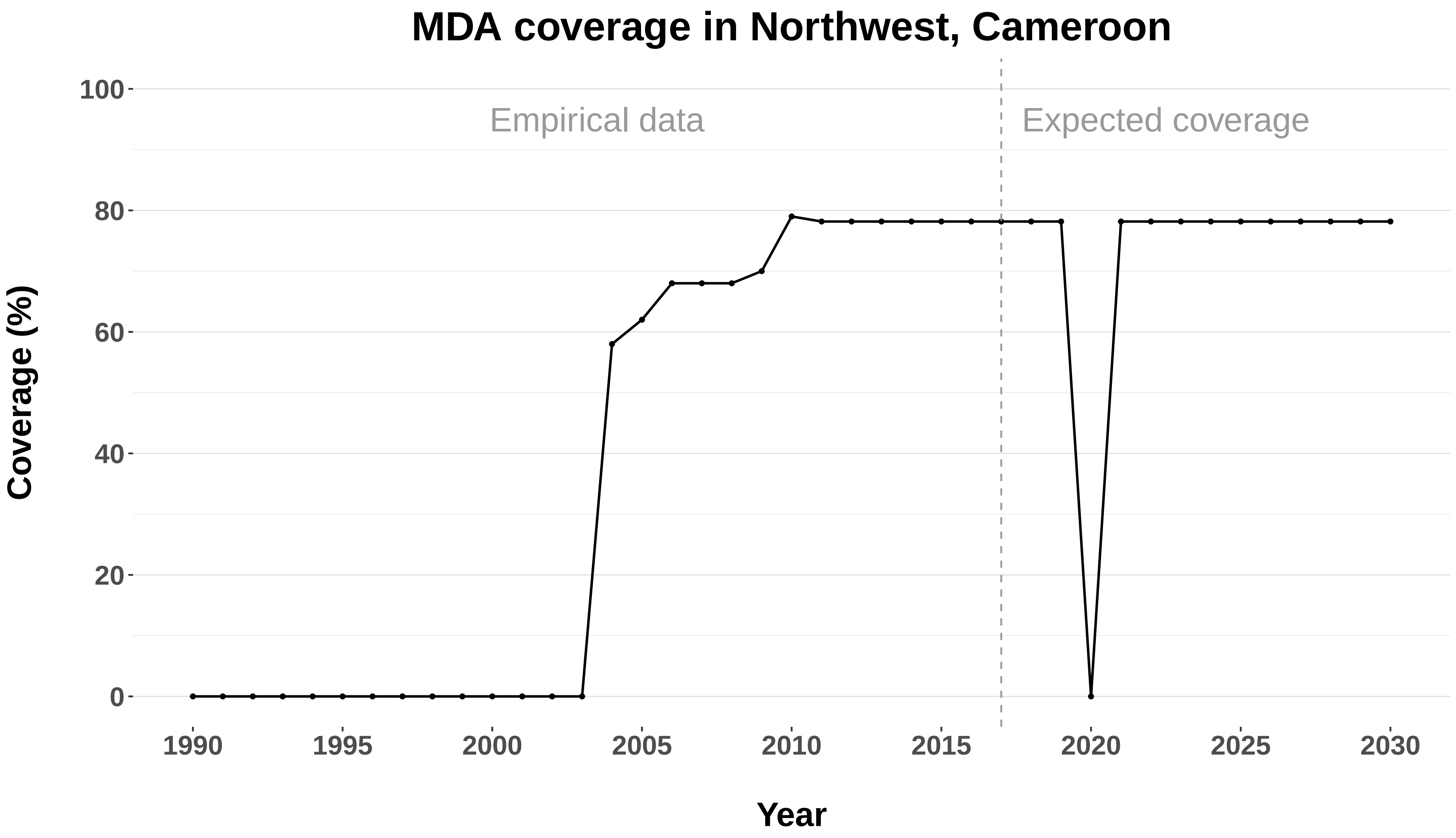

MDA coverage in Northwest, Cameroon
Empirical data
Expected coverage
Coverage (%)
100
80
60
40
20
0
1990
1995
2000
2005
2010
2015
2020
2025
2030
Year

**Project, country:**	**P20Cameroon, Cameroon**
Bioclime:	
Endemicity level(s):	Hyperendemic
MDA start year:	2016
Mean MDA coverage per year:	73.0

	1990			2020			2030		
	N	%	DALYs	N	%	DALYs	N	%	DALYs
Total populaton at risk	93	100	_	211	100	_	276	100	_
**Infection:**									
Mf-positive	40	43.3	_	64	30.5	_	8	2.9	_
Mf-positive (age 5+)	40	43.2	_	64	30.5	_	8	2.9	_
Worm infected	50	53.4	_	99	46.7	_	40	14.5	_
**Clinical manifestations:**									
Palpable nodules	14	15.2	<0.5	25	11.8	<0.5	3	1.1	<0.5
Reversible skin disease:									
Severe itch	6	6.9	1	7	3.5	1	1	0.2	<0.5
Reactive skin disease	5	5.1	<0.5	2	1.1	<0.5	<0.5	<0.05	<0.5
**Reversible skin disease**	**11**	**12.0**	**1**	**10**	**4.5**	**1**	**1**	**0.3**	**<0.5**
Irreversible skin disease:									
Mild depigmentation	1	1.5	<0.5	3	1.3	<0.5	2	0.8	<0.5
Severe depigmentation	2	1.7	<0.5	3	1.4	<0.5	2	0.8	<0.5
Atrophy	<0.5	<0.05	<0.5	<0.5	<0.05	<0.5	<0.5	<0.05	<0.5
Hanging groin	<0.5	<0.05	<0.5	<0.5	<0.05	<0.5	<0.5	<0.05	<0.5
**Irreversible skin disease**	**3**	**3.2**	**<0.5**	**6**	**2.7**	**<0.5**	**4**	**1.5**	**<0.5**
**Total skin disease (OSD)**	**28**	**30.4**	**2**	**40**	**19.0**	**2**	**8**	**2.9**	**<0.5**
Eye disease (OED):									
Visual impairment	<0.5	0.1	<0.5	<0.5	0.1	<0.5	<0.5	<0.05	<0.5
Blindness	<0.5	0.1	<0.5	<0.5	<0.05	1	<0.5	<0.05	<0.5
**Eye disease (OED)**	**<0.5**	**0.1**	**<0.5**	**<0.5**	**0.1**	**1**	**<0.5**	**<0.05**	**<0.5**
**Total all manifestations**	**29**	**30.6**	**2**	**40**	**19.1**	**3**	**8**	**2.9**	**1**

Note 1: Population at risk, number of cases (N) and DALYs are presented in thousands.

Note 2: Total skin disease is defined as the sum of the number of cases with palpable nodules, reversible and irreversible skin disease.

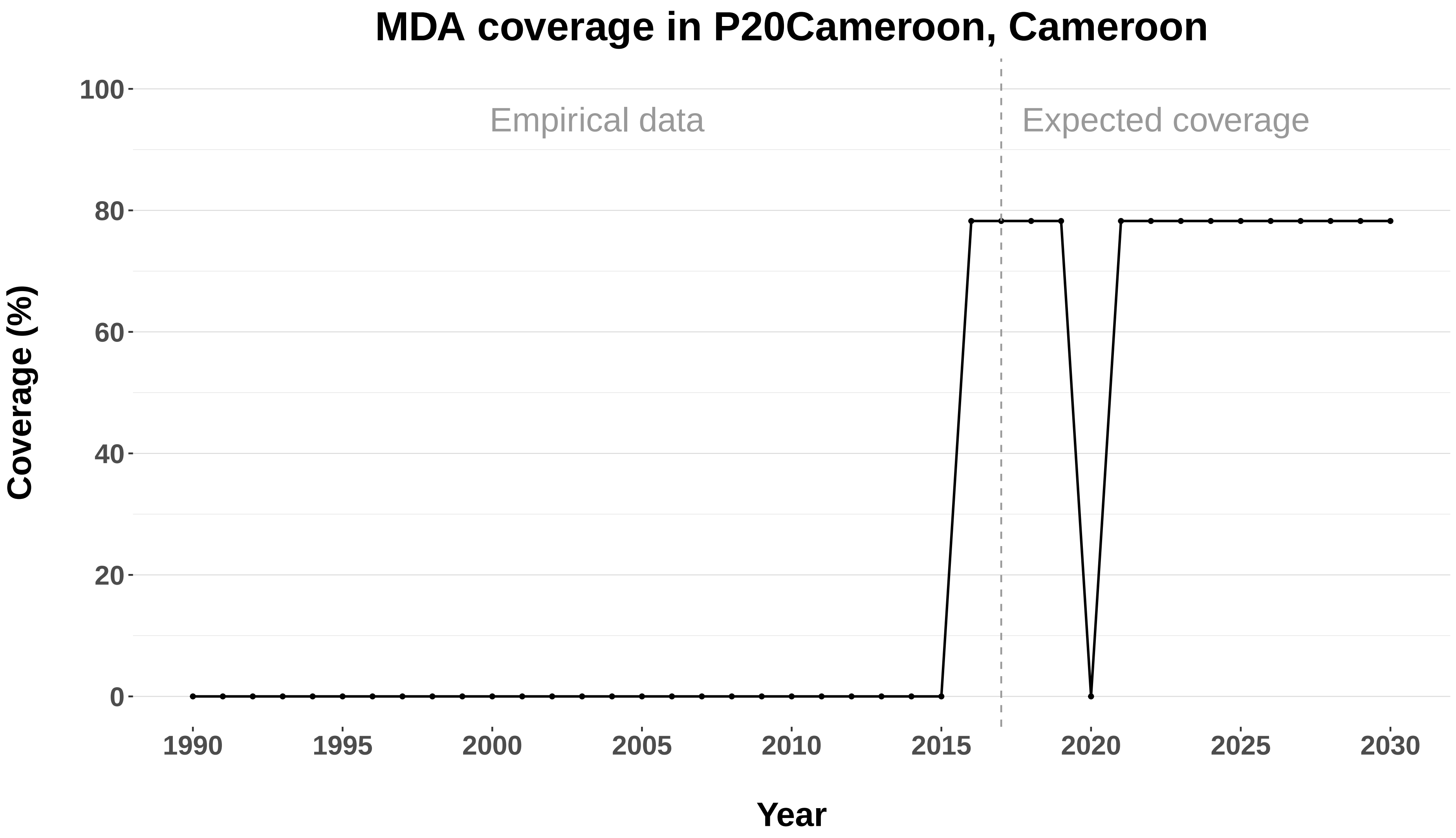
MDA coverage in P20Cameroon, Cameroon
Empirical data
Expected coverage
Coverage (%)
100
80
60
40
20
0
1990
1995
2000
2005
2010
2015
2020
2025
2030
Year

**Project, country:**	**P5Cameroon, Cameroon**
Bioclime:	
Endemicity level(s):	Hypoendemic
MDA start year:	2025
Mean MDA coverage per year:	65.0

	1990			2020			2030		
	N	%	DALYs	N	%	DALYs	N	%	DALYs
Total populaton at risk	929	100	_	2,097	100	_	2,751	100	_
**Infection:**									
Mf-positive	224	24.1	_	482	23.0	_	395	14.4	_
Mf-positive (age 5+)	224	24.1	_	482	23.0	_	395	14.4	_
Worm infected	298	32.1	_	648	30.9	_	702	25.5	_
**Clinical manifestations:**									
Palpable nodules	34	3.6	<0.5	72	3.4	1	64	2.3	1
Reversible skin disease:									
Severe itch	29	3.1	5	61	2.9	11	34	1.3	6
Reactive skin disease	20	2.1	1	41	2.0	2	10	0.4	<0.5
**Reversible skin disease**	**48**	**5.2**	**6**	**102**	**4.9**	**13**	**45**	**1.6**	**7**
Irreversible skin disease:									
Mild depigmentation	8	0.9	<0.5	16	0.8	<0.5	17	0.6	<0.5
Severe depigmentation	6	0.7	<0.5	14	0.7	1	15	0.5	1
Atrophy	<0.5	<0.05	<0.5	<0.5	<0.05	<0.5	<0.5	<0.05	<0.5
Hanging groin	<0.5	<0.05	<0.5	<0.5	<0.05	<0.5	<0.5	<0.05	<0.5
**Irreversible skin disease**	**14**	**1.5**	**1**	**31**	**1.5**	**1**	**31**	**1.1**	**1**
**Total skin disease (OSD)**	**96**	**10.4**	**7**	**205**	**9.8**	**15**	**140**	**5.1**	**9**
Eye disease (OED):									
Visual impairment	<0.5	<0.05	<0.5	<0.5	<0.05	<0.5	<0.5	<0.05	<0.5
Blindness	<0.5	<0.05	1	<0.5	<0.05	1	<0.5	<0.05	1
**Eye disease (OED)**	**<0.5**	**<0.05**	**1**	**<0.5**	**<0.05**	**1**	**1**	**<0.05**	**1**
**Total all manifestations**	**97**	**10.4**	**8**	**205**	**9.8**	**16**	**141**	**5.1**	**10**

Note 1: Population at risk, number of cases (N) and DALYs are presented in thousands.

Note 2: Total skin disease is defined as the sum of the number of cases with palpable nodules, reversible and irreversible skin disease.

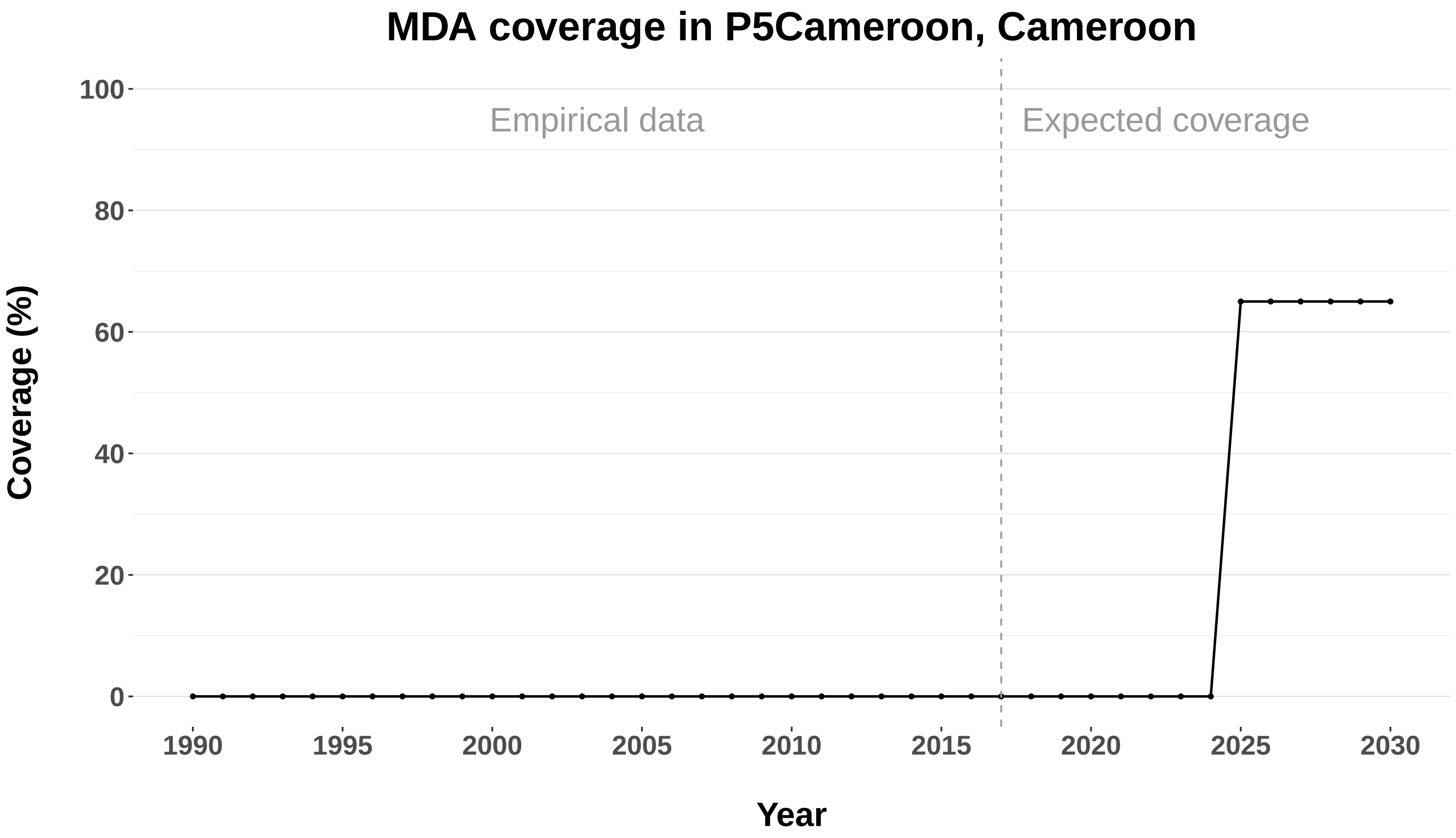

MDA coverage in P5Cameroon, Cameroon
Empirical data
Expected coverage
Coverage (%)
100
80
60
40
20
0
1990
1995
2000
2005
2010
2015
2020
2025
2030
Year

**Project, country:**	**South, Cameroon**
Bioclime:	
Endemicity level(s):	Hyperendemic
MDA start year:	2005
Mean MDA coverage per year:	70.7

	1990			2020			2030		
	N	%	DALYs	N	%	DALYs	N	%	DALYs
Total populaton at risk	185	100	_	418	100	_	548	100	_
**Infection:**									
Mf-positive	60	32.4	_	4	1.0	_	<0.5	<0.05	_
Mf-positive (age 5+)	60	32.4	_	4	1.0	_	<0.5	<0.05	_
Worm infected	78	42.1	_	35	8.4	_	2	0.3	_
**Clinical manifestations:**									
Palpable nodules	15	8.1	<0.5	1	0.3	<0.5	<0.5	<0.05	<0.5
Reversible skin disease:									
Severe itch	8	4.4	1	<0.5	0.1	<0.5	<0.5	<0.05	<0.5
Reactive skin disease	6	3.0	<0.5	<0.5	<0.05	<0.5	<0.5	<0.05	<0.5
**Reversible skin disease**	**14**	**7.4**	**2**	**<0.5**	**0.1**	**<0.5**	**<0.5**	**<0.05**	**<0.5**
Irreversible skin disease:									
Mild depigmentation	2	1.0	<0.5	2	0.5	<0.5	1	0.3	<0.5
Severe depigmentation	2	1.0	<0.5	2	0.4	<0.5	1	0.2	<0.5
Atrophy	<0.5	<0.05	<0.5	<0.5	<0.05	<0.5	<0.5	<0.05	<0.5
Hanging groin	<0.5	<0.05	<0.5	<0.5	<0.05	<0.5	<0.5	<0.05	<0.5
**Irreversible skin disease**	**4**	**2.0**	**<0.5**	**4**	**0.9**	**<0.5**	**2**	**0.5**	**<0.5**
**Total skin disease (OSD)**	**33**	**17.6**	**2**	**6**	**1.4**	**<0.5**	**3**	**0.5**	**<0.5**
Eye disease (OED):									
Visual impairment	<0.5	<0.05	<0.5	<0.5	<0.05	<0.5	<0.5	<0.05	<0.5
Blindness	<0.5	<0.05	<0.5	<0.5	<0.05	<0.5	<0.5	<0.05	<0.5
**Eye disease (OED)**	**<0.5**	**<0.05**	**<0.5**	**<0.5**	**<0.05**	**<0.5**	**<0.5**	**<0.05**	**<0.5**
**Total all manifestations**	**33**	**17.6**	**2**	**6**	**1.4**	**<0.5**	**3**	**0.5**	**<0.5**

Note 1: Population at risk, number of cases (N) and DALYs are presented in thousands.

Note 2: Total skin disease is defined as the sum of the number of cases with palpable nodules, reversible and irreversible skin disease.

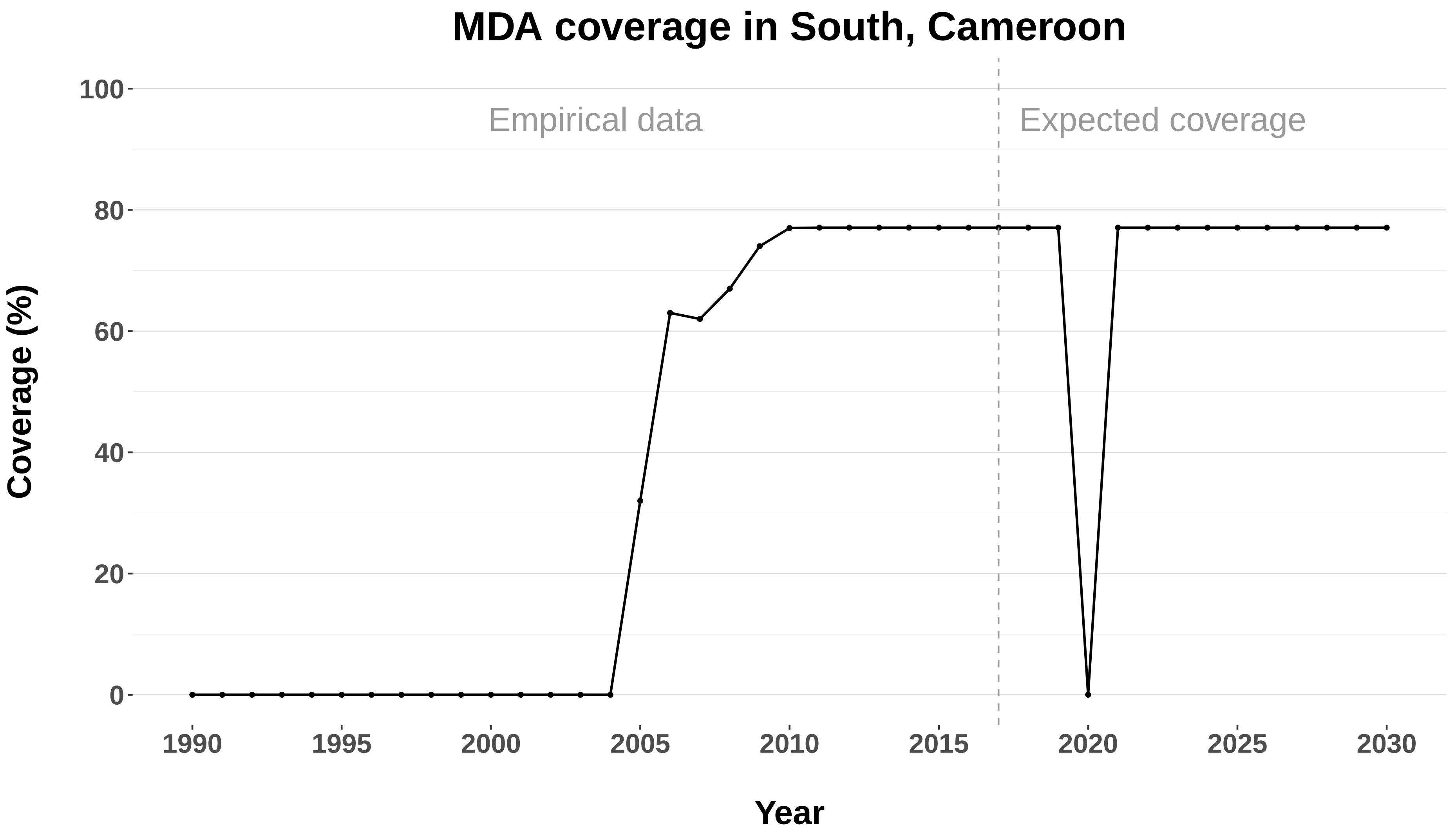
MDA coverage in South, Cameroon
Empirical data
Expected coverage
Coverage (%)
100
80
60
40
20
0
1990
1995
2000
2005
2010
2015
2020
2025
2030
Year

**Project, country:**	**South West 1, Cameroon**
Bioclime:	
Endemicity level(s):	Hyperendemic
MDA start year:	1999
Mean MDA coverage per year:	68.8

	1990			2020			2030		
	N	%	DALYs	N	%	DALYs	N	%	DALYs
Total populaton at risk	243	100	_	548	100	_	719	100	_
**Infection:**									
Mf-positive	143	59.0	_	16	3.0	_	<0.5	<0.05	_
Mf-positive (age 5+)	143	58.9	_	16	3.0	_	<0.5	<0.05	_
Worm infected	163	66.9	_	88	16.1	_	8	1.1	_
**Clinical manifestations:**									
Palpable nodules	72	29.6	1	7	1.2	<0.5	<0.5	<0.05	<0.5
Reversible skin disease:									
Severe itch	31	12.7	6	1	0.2	<0.5	<0.5	<0.05	<0.5
Reactive skin disease	26	10.5	1	<0.5	<0.05	<0.5	<0.5	<0.05	<0.5
**Reversible skin disease**	**56**	**23.2**	**7**	**1**	**0.2**	**<0.5**	**<0.5**	**<0.05**	**<0.5**
Irreversible skin disease:									
Mild depigmentation	6	2.6	<0.5	6	1.0	<0.5	4	0.6	<0.5
Severe depigmentation	9	3.7	1	7	1.3	<0.5	5	0.7	<0.5
Atrophy	<0.5	0.1	<0.5	<0.5	<0.05	<0.5	<0.5	<0.05	<0.5
Hanging groin	<0.5	0.1	<0.5	<0.5	<0.05	<0.5	<0.5	<0.05	<0.5
**Irreversible skin disease**	**16**	**6.5**	**1**	**13**	**2.4**	**1**	**9**	**1.2**	**<0.5**
**Total skin disease (OSD)**	**144**	**59.3**	**8**	**21**	**3.8**	**1**	**9**	**1.3**	**<0.5**
Eye disease (OED):									
Visual impairment	1	0.2	<0.5	<0.5	0.1	<0.5	<0.5	<0.05	<0.5
Blindness	1	0.3	5	<0.5	<0.05	1	<0.5	<0.05	<0.5
**Eye disease (OED)**	**1**	**0.5**	**5**	**1**	**0.1**	**1**	**<0.5**	**<0.05**	**<0.5**
**Total all manifestations**	**145**	**59.8**	**13**	**22**	**3.9**	**2**	**9**	**1.3**	**1**

Note 1: Population at risk, number of cases (N) and DALYs are presented in thousands.

Note 2: Total skin disease is defined as the sum of the number of cases with palpable nodules, reversible and irreversible skin disease.

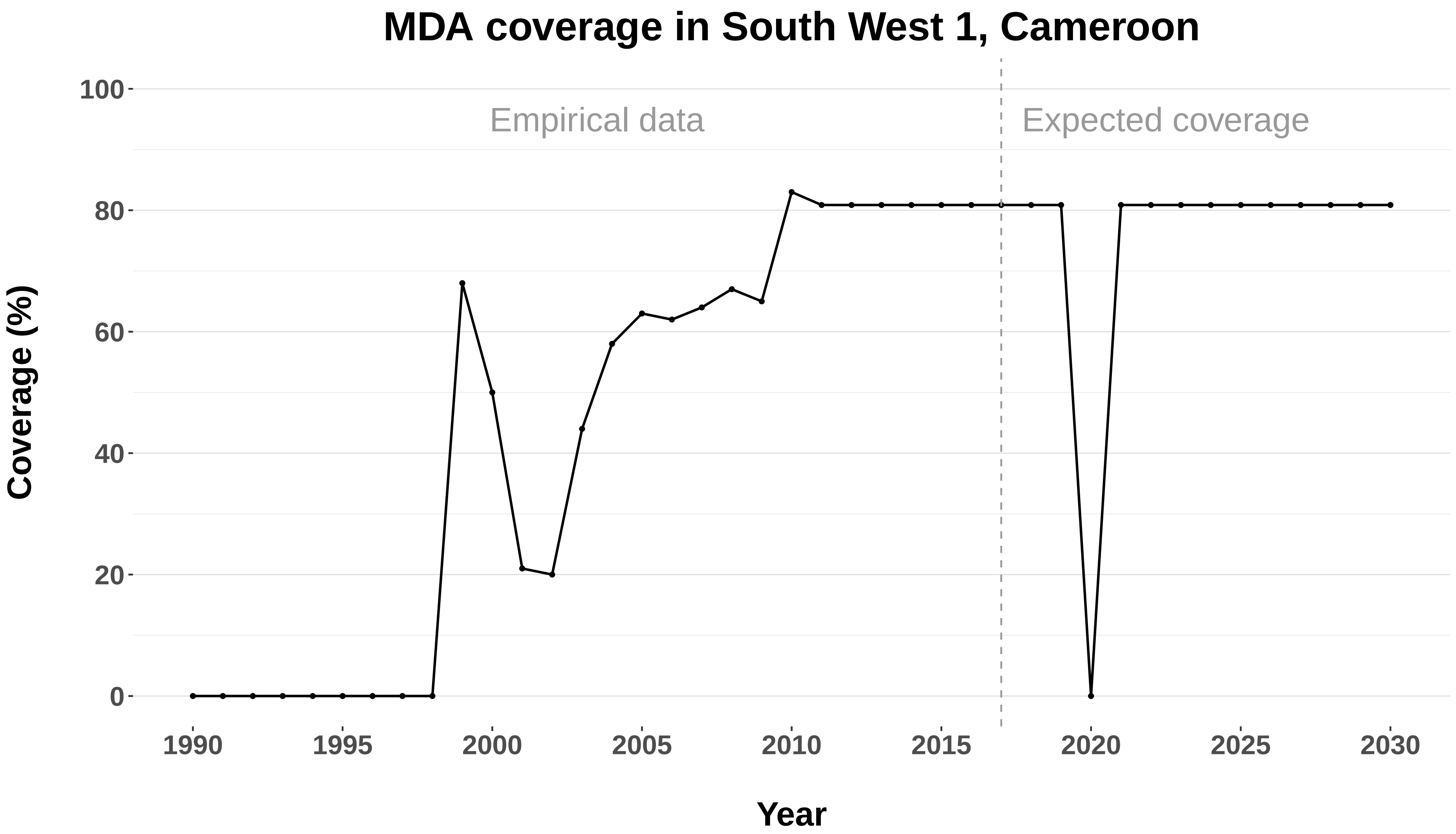
MDA coverage in South West 1, Cameroon
Empirical data
Expected coverage
Coverage (%)
100
80
60
40
20
0
1990
1995
2000
2005
2010
2015
2020
2025
2030
Year

**Project, country:**	**South West 2, Cameroon**
Bioclime:	
Endemicity level(s):	Hyperendemic
MDA start year:	2001
Mean MDA coverage per year:	74.6

	1990			2020			2030		
	N	%	DALYs	N	%	DALYs	N	%	DALYs
Total populaton at risk	165	100	_	373	100	_	489	100	_
**Infection:**									
Mf-positive	88	53.2	_	4	1.0	_	<0.5	<0.05	_
Mf-positive (age 5+)	88	53.1	_	4	1.0	_	<0.5	<0.05	_
Worm infected	103	62.1	_	35	9.3	_	2	0.3	_
**Clinical manifestations:**									
Palpable nodules	39	23.4	<0.5	2	0.5	<0.5	<0.5	<0.05	<0.5
Reversible skin disease:									
Severe itch	17	10.1	3	<0.5	0.1	<0.5	<0.5	<0.05	<0.5
Reactive skin disease	13	8.1	1	<0.5	<0.05	<0.5	<0.5	<0.05	<0.5
**Reversible skin disease**	**30**	**18.2**	**4**	**<0.5**	**0.1**	**<0.5**	**<0.5**	**<0.05**	**<0.5**
Irreversible skin disease:									
Mild depigmentation	3	2.1	<0.5	3	0.9	<0.5	2	0.5	<0.5
Severe depigmentation	5	2.8	<0.5	4	1.1	<0.5	2	0.5	<0.5
Atrophy	<0.5	<0.05	<0.5	<0.5	<0.05	<0.5	<0.5	<0.05	<0.5
Hanging groin	<0.5	0.1	<0.5	<0.5	<0.05	<0.5	<0.5	<0.05	<0.5
**Irreversible skin disease**	**8**	**5.0**	**<0.5**	**7**	**2.0**	**<0.5**	**5**	**1.0**	**<0.5**
**Total skin disease (OSD)**	**77**	**46.6**	**4**	**10**	**2.6**	**<0.5**	**5**	**1.0**	**<0.5**
Eye disease (OED):									
Visual impairment	<0.5	0.2	<0.5	<0.5	<0.05	<0.5	<0.5	<0.05	<0.5
Blindness	<0.5	0.2	2	<0.5	<0.05	1	<0.5	<0.05	<0.5
**Eye disease (OED)**	**1**	**0.3**	**2**	**<0.5**	**0.1**	**1**	**<0.5**	**<0.05**	**<0.5**
**Total all manifestations**	**78**	**46.9**	**6**	**10**	**2.6**	**1**	**5**	**1.0**	**<0.5**

Note 1: Population at risk, number of cases (N) and DALYs are presented in thousands.

Note 2: Total skin disease is defined as the sum of the number of cases with palpable nodules, reversible and irreversible skin disease.

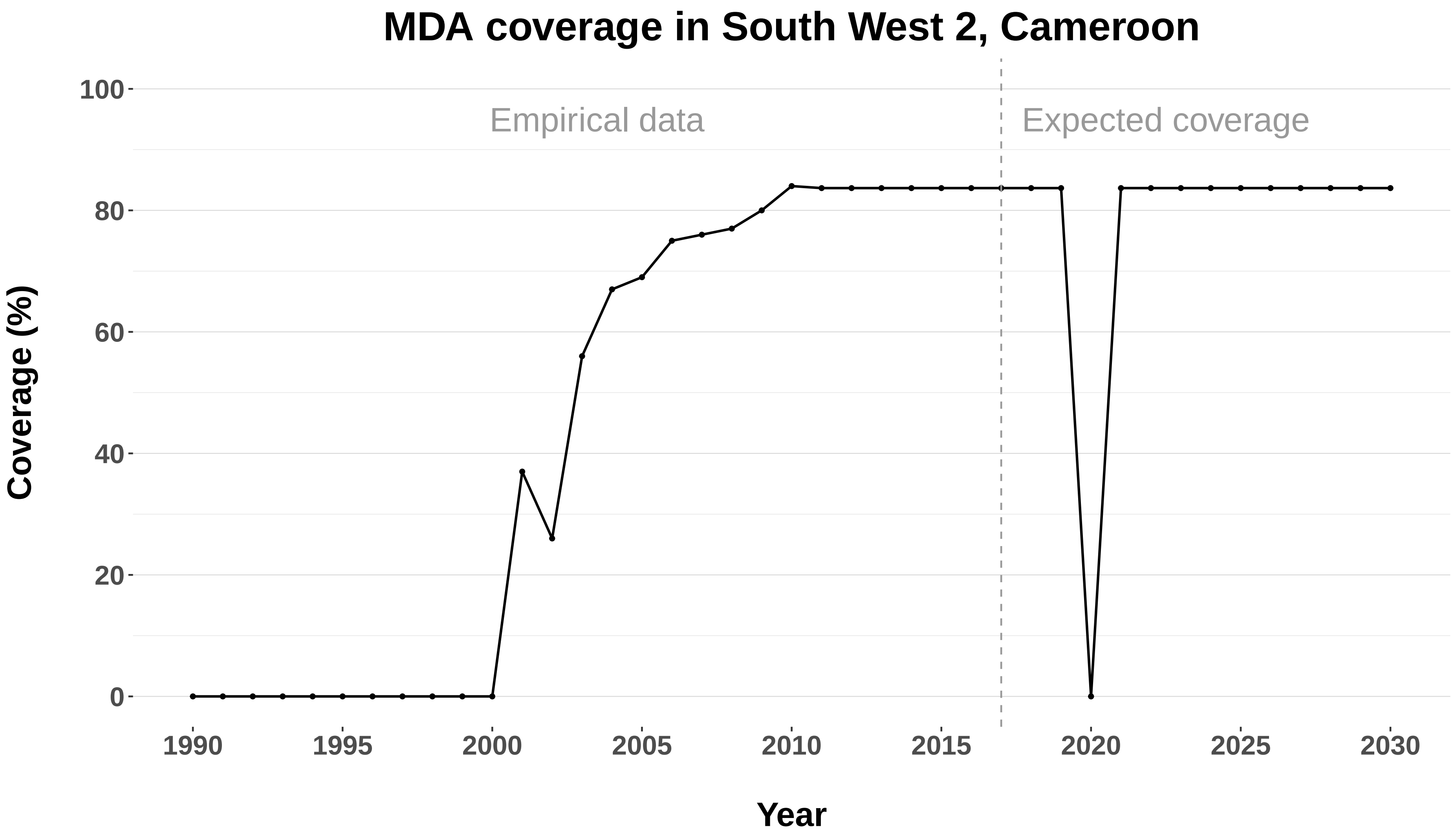
MDA coverage in South West 2, Cameroon
Empirical data
Expected coverage
Coverage (%)
100
80
60
40
20
0
1990
1995
2000
2005
2010
2015
2020
2025
2030
Year

**Project, country:**	**Western, Cameroon**
Bioclime:	
Endemicity level(s):	Hyperendemic
MDA start year:	2001
Mean MDA coverage per year:	72.9

	1990			2020			2030		
	N	%	DALYs	N	%	DALYs	N	%	DALYs
Total populaton at risk	1,016	100	_	2,292	100	_	3,007	100	_
**Infection:**									
Mf-positive	673	66.3	_	127	5.5	_	9	0.3	_
Mf-positive (age 5+)	670	65.9	_	127	5.5	_	9	0.3	_
Worm infected	743	73.2	_	497	21.7	_	95	3.2	_
**Clinical manifestations:**									
Palpable nodules	391	38.5	4	50	2.2	1	3	0.1	<0.5
Reversible skin disease:									
Severe itch	170	16.7	31	9	0.4	2	<0.5	<0.05	<0.5
Reactive skin disease	149	14.6	7	<0.5	<0.05	<0.5	<0.5	<0.05	<0.5
**Reversible skin disease**	**318**	**31.3**	**38**	**10**	**0.4**	**2**	**<0.5**	**<0.05**	**<0.5**
Irreversible skin disease:									
Mild depigmentation	31	3.1	<0.5	31	1.4	<0.5	23	0.8	<0.5
Severe depigmentation	54	5.3	3	49	2.1	3	33	1.1	2
Atrophy	2	0.2	<0.5	<0.5	<0.05	<0.5	<0.5	<0.05	<0.5
Hanging groin	4	0.3	1	2	0.1	1	1	<0.05	<0.5
**Irreversible skin disease**	**90**	**8.9**	**5**	**82**	**3.6**	**4**	**57**	**1.9**	**3**
**Total skin disease (OSD)**	**800**	**78.7**	**47**	**142**	**6.2**	**7**	**60**	**2.0**	**3**
Eye disease (OED):									
Visual impairment	5	0.5	<0.5	4	0.2	<0.5	2	0.1	<0.5
Blindness	7	0.7	48	2	0.1	17	1	<0.05	7
**Eye disease (OED)**	**11**	**1.1**	**48**	**6**	**0.3**	**17**	**3**	**0.1**	**7**
**Total all manifestations**	**811**	**79.9**	**95**	**148**	**6.5**	**24**	**63**	**2.1**	**9**

Note 1: Population at risk, number of cases (N) and DALYs are presented in thousands.

Note 2: Total skin disease is defined as the sum of the number of cases with palpable nodules, reversible and irreversible skin disease.

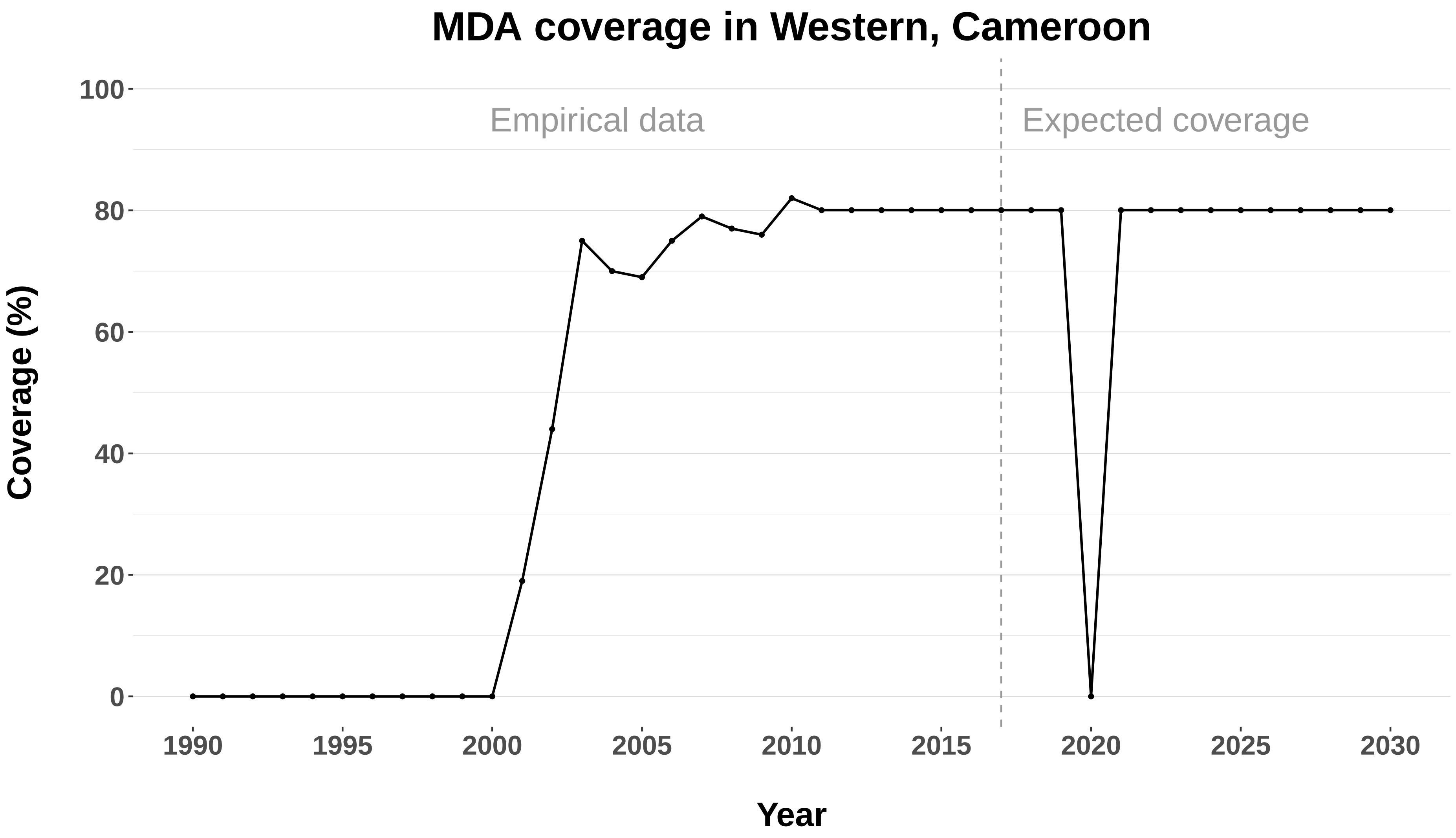

MDA coverage in Western, Cameroon
Empirical data
Expected coverage
Coverage (%)
100
80
60
40
20
0
1990
1995
2000
2005
2010
2015
2020
2025
2030
Year

**Project, country:**	**CAR region 3, CAR**
Bioclime:	
Endemicity level(s):	Hyperendemic
MDA start year:	1999
Mean MDA coverage per year:	62.5

	1990			2020			2030		
	N	%	DALYs	N	%	DALYs	N	%	DALYs
Total populaton at risk	265	100	_	598	100	_	784	100	_
**Infection:**									
Mf-positive	147	55.6	_	17	2.9	_	3	0.4	_
Mf-positive (age 5+)	147	55.5	_	17	2.9	_	3	0.4	_
Worm infected	172	64.8	_	67	11.2	_	17	2.2	_
**Clinical manifestations:**									
Palpable nodules	66	24.8	1	5	0.8	<0.5	1	0.1	<0.5
Reversible skin disease:									
Severe itch	28	10.5	5	1	0.1	<0.5	<0.5	<0.05	<0.5
Reactive skin disease	22	8.3	1	<0.5	<0.05	<0.5	<0.5	<0.05	<0.5
**Reversible skin disease**	**50**	**18.9**	**6**	**1**	**0.1**	**<0.5**	**<0.5**	**<0.05**	**<0.5**
Irreversible skin disease:									
Mild depigmentation	6	2.2	<0.5	5	0.8	<0.5	3	0.4	<0.5
Severe depigmentation	7	2.7	<0.5	5	0.8	<0.5	3	0.4	<0.5
Atrophy	<0.5	<0.05	<0.5	<0.5	<0.05	<0.5	<0.5	<0.05	<0.5
Hanging groin	<0.5	<0.05	<0.5	<0.5	<0.05	<0.5	<0.5	<0.05	<0.5
**Irreversible skin disease**	**13**	**4.9**	**1**	**9**	**1.6**	**<0.5**	**6**	**0.8**	**<0.5**
**Total skin disease (OSD)**	**129**	**48.6**	**7**	**15**	**2.5**	**1**	**7**	**0.9**	**<0.5**
Eye disease (OED):									
Visual impairment	12	4.5	<0.5	9	1.6	<0.5	6	0.8	<0.5
Blindness	7	2.5	53	2	0.4	19	1	0.1	8
**Eye disease (OED)**	**19**	**7.0**	**53**	**12**	**2.0**	**19**	**7**	**0.9**	**8**
**Total all manifestations**	**147**	**55.6**	**61**	**26**	**4.4**	**20**	**14**	**1.8**	**9**

Note 1: Population at risk, number of cases (N) and DALYs are presented in thousands.

Note 2: Total skin disease is defined as the sum of the number of cases with palpable nodules, reversible and irreversible skin disease.

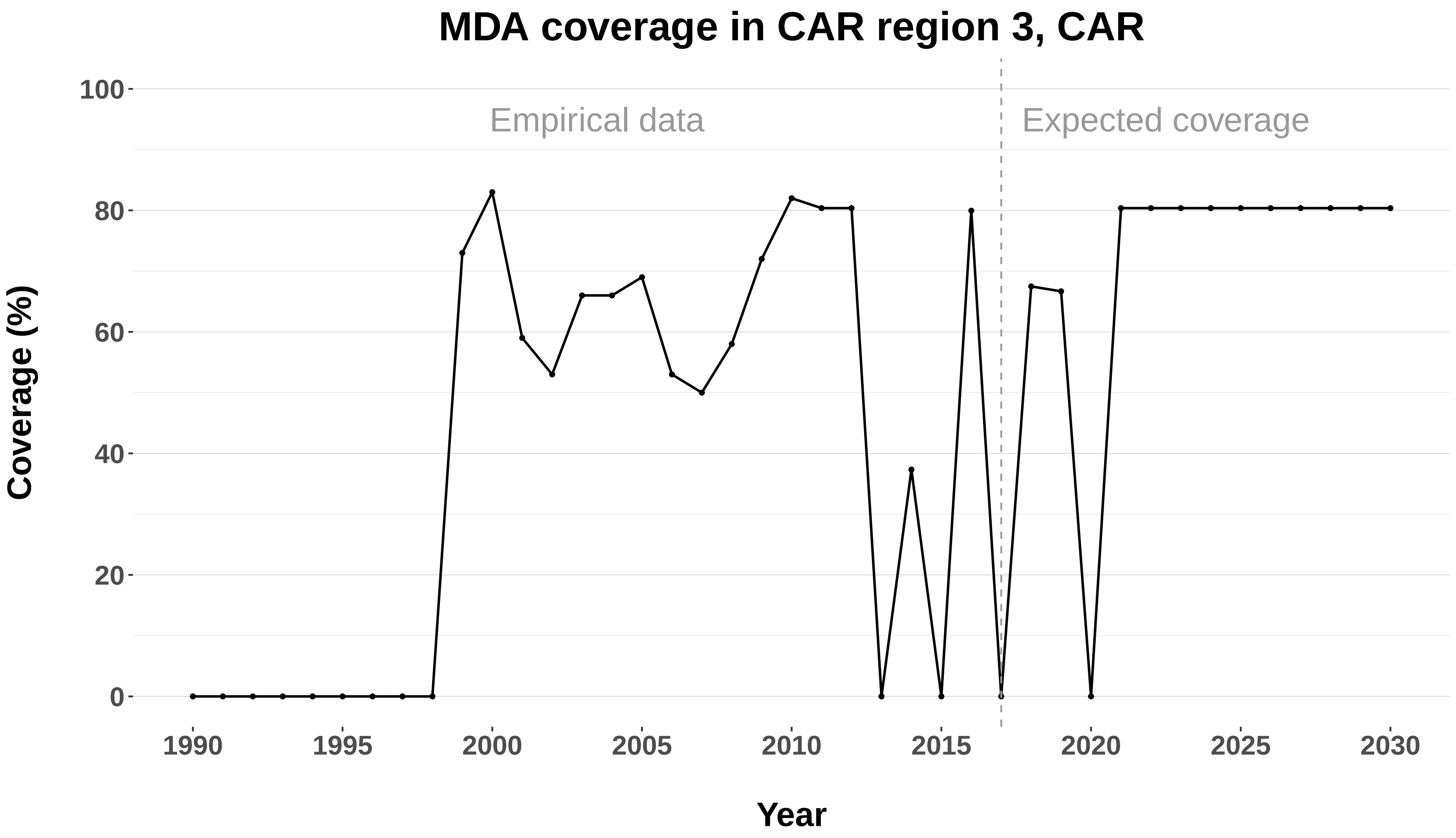
MDA coverage in CAR region 3, CAR
Empirical data
Expected coverage
Coverage (%)
0
20
40
60
80
100
1990
1995
2000
2005
2010
2015
2020
2025
2030
Year

**Project, country:**	**CAR region 4, CAR**
Bioclime:	
Endemicity level(s):	Hyperendemic
MDA start year:	1999
Mean MDA coverage per year:	61.5

	1990			2020			2030		
	N	%	DALYs	N	%	DALYs	N	%	DALYs
Total populaton at risk	276	100	_	623	100	_	817	100	_
**Infection:**									
Mf-positive	102	36.8	_	5	0.8	_	1	0.1	_
Mf-positive (age 5+)	101	36.7	_	5	0.8	_	1	0.1	_
Worm infected	127	46.0	_	27	4.3	_	5	0.6	_
**Clinical manifestations:**									
Palpable nodules	31	11.4	<0.5	1	0.2	<0.5	<0.5	<0.05	<0.5
Reversible skin disease:									
Severe itch	16	5.6	3	<0.5	<0.05	<0.5	<0.5	<0.05	<0.5
Reactive skin disease	11	4.2	1	<0.5	<0.05	<0.5	<0.5	<0.05	<0.5
**Reversible skin disease**	**27**	**9.8**	**3**	**<0.5**	**<0.05**	**<0.5**	**<0.5**	**<0.05**	**<0.5**
Irreversible skin disease:									
Mild depigmentation	4	1.3	<0.5	3	0.4	<0.5	2	0.2	<0.5
Severe depigmentation	4	1.3	<0.5	2	0.4	<0.5	1	0.2	<0.5
Atrophy	<0.5	<0.05	<0.5	<0.5	<0.05	<0.5	<0.5	<0.05	<0.5
Hanging groin	<0.5	<0.05	<0.5	<0.5	<0.05	<0.5	<0.5	<0.05	<0.5
**Irreversible skin disease**	**7**	**2.6**	**<0.5**	**5**	**0.8**	**<0.5**	**3**	**0.4**	**<0.5**
**Total skin disease (OSD)**	**66**	**23.8**	**4**	**6**	**1.0**	**<0.5**	**3**	**0.4**	**<0.5**
Eye disease (OED):									
Visual impairment	7	2.4	<0.5	5	0.8	<0.5	3	0.4	<0.5
Blindness	3	1.0	22	1	0.1	7	<0.5	<0.05	3
**Eye disease (OED)**	**10**	**3.5**	**22**	**6**	**0.9**	**7**	**3**	**0.4**	**3**
**Total all manifestations**	**75**	**27.3**	**26**	**12**	**2.0**	**7**	**7**	**0.8**	**3**

Note 1: Population at risk, number of cases (N) and DALYs are presented in thousands.

Note 2: Total skin disease is defined as the sum of the number of cases with palpable nodules, reversible and irreversible skin disease.

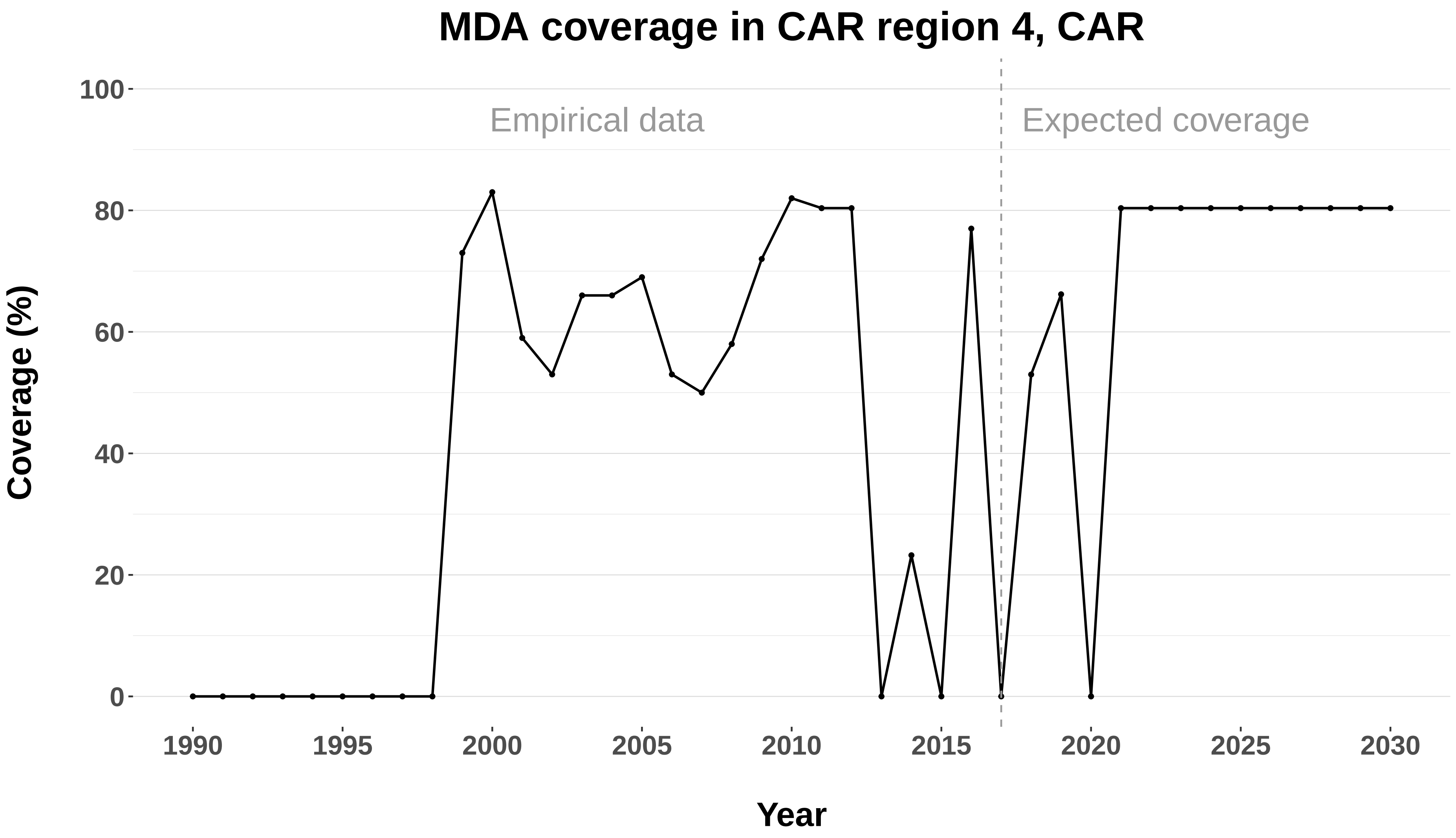

MDA coverage in CAR region 4, CAR
Empirical data
Expected coverage
Coverage (%)
100
80
60
40
20
0
1990
1995
2000
2005
2010
2015
2020
2025
2030
Year

**Project, country:**	**CAR region 5, CAR**
Bioclime:	
Endemicity level(s):	Hyperendemic
MDA start year:	1999
Mean MDA coverage per year:	58.9

	1990			2020			2030		
	N	%	DALYs	N	%	DALYs	N	%	DALYs
Total populaton at risk	255	100	_	576	100	_	756	100	_
**Infection:**									
Mf-positive	80	31.4	_	2	0.3	_	<0.5	<0.05	_
Mf-positive (age 5+)	80	31.4	_	2	0.3	_	<0.5	<0.05	_
Worm infected	104	40.6	_	14	2.4	_	2	0.3	_
**Clinical manifestations:**									
Palpable nodules	19	7.6	<0.5	<0.5	0.1	<0.5	<0.5	<0.05	<0.5
Reversible skin disease:									
Severe itch	11	4.3	2	<0.5	<0.05	<0.5	<0.5	<0.05	<0.5
Reactive skin disease	8	3.0	<0.5	<0.5	<0.05	<0.5	<0.5	<0.05	<0.5
**Reversible skin disease**	**19**	**7.3**	**2**	**<0.5**	**<0.05**	**<0.5**	**<0.5**	**<0.05**	**<0.5**
Irreversible skin disease:									
Mild depigmentation	3	1.1	<0.5	2	0.3	<0.5	1	0.2	<0.5
Severe depigmentation	3	1.0	<0.5	2	0.3	<0.5	1	0.1	<0.5
Atrophy	<0.5	<0.05	<0.5	<0.5	<0.05	<0.5	<0.5	<0.05	<0.5
Hanging groin	<0.5	<0.05	<0.5	<0.5	<0.05	<0.5	<0.5	<0.05	<0.5
**Irreversible skin disease**	**5**	**2.1**	**<0.5**	**3**	**0.6**	**<0.5**	**2**	**0.3**	**<0.5**
**Total skin disease (OSD)**	**43**	**16.9**	**3**	**4**	**0.7**	**<0.5**	**2**	**0.3**	**<0.5**
Eye disease (OED):									
Visual impairment	5	1.9	<0.5	3	0.6	<0.5	2	0.3	<0.5
Blindness	2	0.7	12	<0.5	0.1	3	<0.5	<0.05	1
**Eye disease (OED)**	**6**	**2.5**	**12**	**4**	**0.7**	**4**	**2**	**0.3**	**1**
**Total all manifestations**	**50**	**19.4**	**15**	**8**	**1.3**	**4**	**4**	**0.6**	**1**

Note 1: Population at risk, number of cases (N) and DALYs are presented in thousands.

Note 2: Total skin disease is defined as the sum of the number of cases with palpable nodules, reversible and irreversible skin disease.

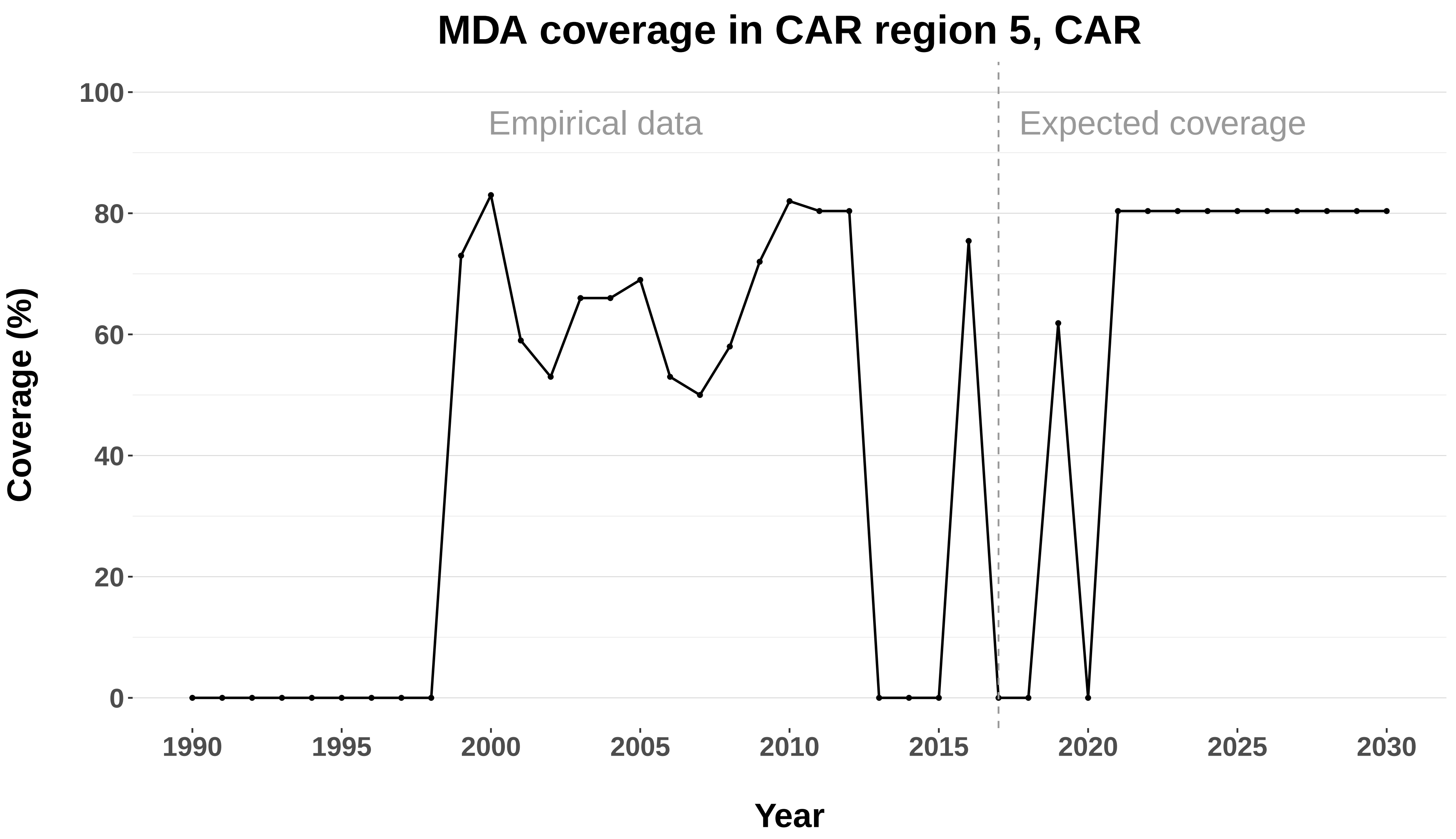
MDA coverage in CAR region 5, CAR
Empirical data
Expected coverage
Coverage (%)
Year
0
20
40
60
80
100
1990
1995
2000
2005
2010
2015
2020
2025
2030

**Project, country:**	**CAR region 6, CAR**
Bioclime:	
Endemicity level(s):	Hyperendemic
MDA start year:	1999
Mean MDA coverage per year:	60.3

	1990			2020			2030		
	N	%	DALYs	N	%	DALYs	N	%	DALYs
Total populaton at risk	393	100	_	887	100	_	1,163	100	_
**Infection:**									
Mf-positive	181	46.0	_	18	2.0	_	3	0.2	_
Mf-positive (age 5+)	180	45.9	_	18	2.0	_	3	0.2	_
Worm infected	218	55.4	_	66	7.5	_	19	1.6	_
**Clinical manifestations:**									
Palpable nodules	69	17.7	1	4	0.5	<0.5	1	0.1	<0.5
Reversible skin disease:									
Severe itch	31	7.9	6	1	0.1	<0.5	<0.5	<0.05	<0.5
Reactive skin disease	24	6.1	1	<0.5	<0.05	<0.5	<0.5	<0.05	<0.5
**Reversible skin disease**	**55**	**13.9**	**7**	**1**	**0.1**	**<0.5**	**<0.5**	**<0.05**	**<0.5**
Irreversible skin disease:									
Mild depigmentation	7	1.7	<0.5	5	0.6	<0.5	3	0.3	<0.5
Severe depigmentation	8	1.9	<0.5	5	0.6	<0.5	3	0.3	<0.5
Atrophy	<0.5	<0.05	<0.5	<0.5	<0.05	<0.5	<0.5	<0.05	<0.5
Hanging groin	<0.5	<0.05	<0.5	<0.5	<0.05	<0.5	<0.5	<0.05	<0.5
**Irreversible skin disease**	**14**	**3.7**	**1**	**10**	**1.2**	**<0.5**	**7**	**0.6**	**<0.5**
**Total skin disease (OSD)**	**139**	**35.2**	**8**	**15**	**1.7**	**1**	**7**	**0.6**	**<0.5**
Eye disease (OED):									
Visual impairment	13	3.4	<0.5	10	1.1	<0.5	6	0.5	<0.5
Blindness	7	1.7	52	2	0.2	17	1	0.1	7
**Eye disease (OED)**	**20**	**5.1**	**52**	**12**	**1.3**	**17**	**7**	**0.6**	**7**
**Total all manifestations**	**158**	**40.3**	**61**	**27**	**3.1**	**18**	**15**	**1.2**	**7**

Note 1: Population at risk, number of cases (N) and DALYs are presented in thousands.

Note 2: Total skin disease is defined as the sum of the number of cases with palpable nodules, reversible and irreversible skin disease.

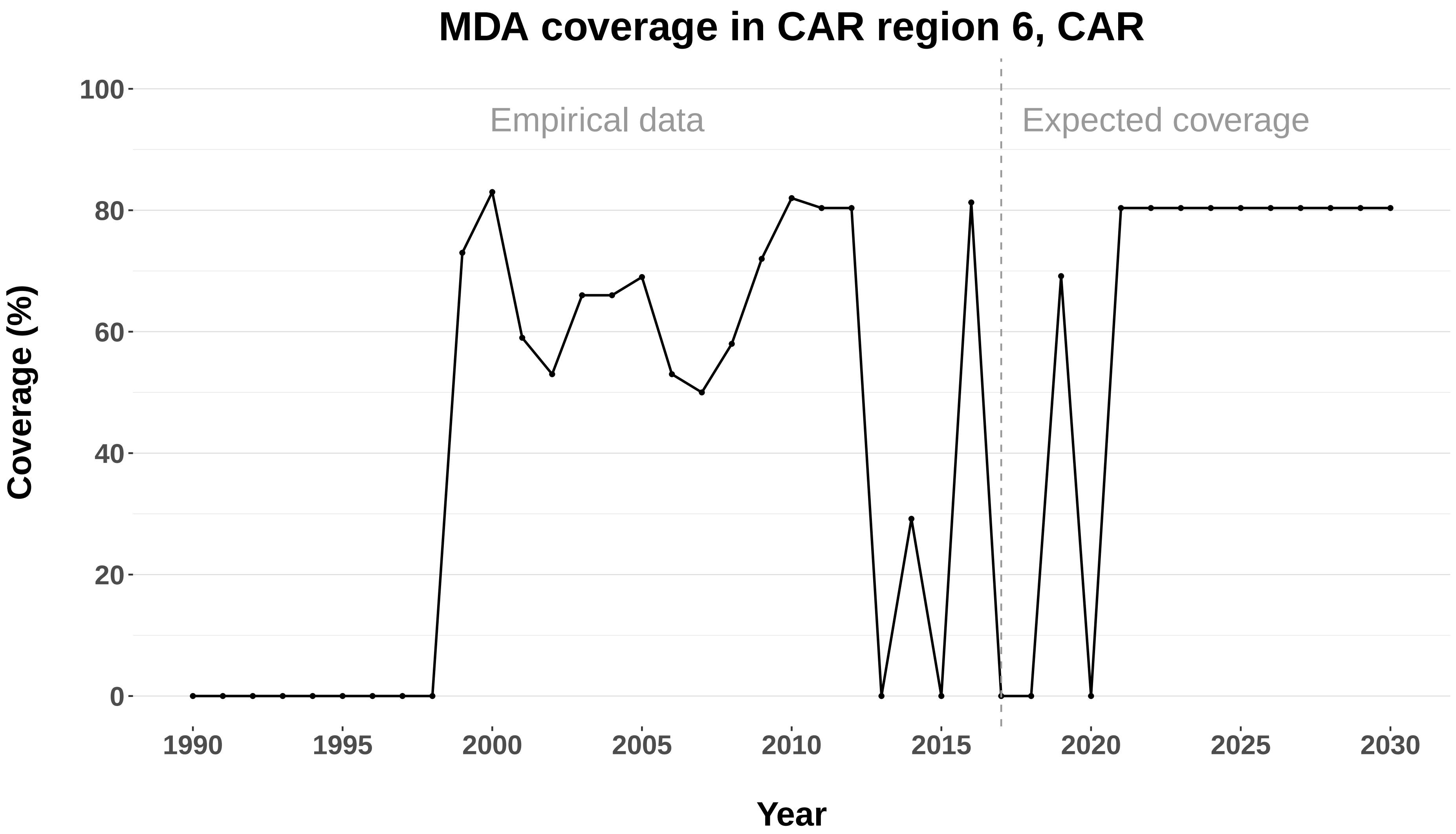

MDA coverage in CAR region 6, CAR
Empirical data
Expected coverage
Coverage (%)
100
80
60
40
20
0
1990
1995
2000
2005
2010
2015
2020
2025
2030
Year

**Project, country:**	**P20CAR, CAR**
Bioclime:	
Endemicity level(s):	Hyperendemic
MDA start year:	2019
Mean MDA coverage per year:	73.7

	1990			2020			2030		
	N	%	DALYs	N	%	DALYs	N	%	DALYs
Total populaton at risk	39	100	_	87	100	_	115	100	_
**Infection:**									
Mf-positive	16	41.3	_	34	39.2	_	14	11.9	_
Mf-positive (age 5+)	16	41.2	_	34	39.2	_	14	11.9	_
Worm infected	20	51.3	_	44	50.9	_	33	29.1	_
**Clinical manifestations:**									
Palpable nodules	5	13.5	<0.5	12	13.4	<0.5	4	3.8	<0.5
Reversible skin disease:									
Severe itch	2	6.3	<0.5	5	5.5	1	1	1.0	<0.5
Reactive skin disease	2	4.6	<0.5	3	3.4	<0.5	<0.5	0.2	<0.5
**Reversible skin disease**	**4**	**10.9**	**1**	**8**	**8.9**	**1**	**1**	**1.1**	**<0.5**
Irreversible skin disease:									
Mild depigmentation	1	1.4	<0.5	1	1.4	<0.5	1	0.9	<0.5
Severe depigmentation	1	1.5	<0.5	1	1.4	<0.5	1	0.9	<0.5
Atrophy	<0.5	<0.05	<0.5	<0.5	<0.05	<0.5	<0.5	<0.05	<0.5
Hanging groin	<0.5	<0.05	<0.5	<0.5	<0.05	<0.5	<0.5	<0.05	<0.5
**Irreversible skin disease**	**1**	**2.9**	**<0.5**	**2**	**2.8**	**<0.5**	**2**	**1.8**	**<0.5**
**Total skin disease (OSD)**	**11**	**27.3**	**1**	**22**	**25.2**	**1**	**8**	**6.7**	**<0.5**
Eye disease (OED):									
Visual impairment	<0.5	<0.05	<0.5	<0.5	0.1	<0.5	<0.5	<0.05	<0.5
Blindness	<0.5	<0.05	<0.5	<0.5	<0.05	<0.5	<0.5	<0.05	<0.5
**Eye disease (OED)**	**<0.5**	**0.1**	**<0.5**	**<0.5**	**0.1**	**<0.5**	**<0.5**	**<0.05**	**<0.5**
**Total all manifestations**	**11**	**27.4**	**1**	**22**	**25.3**	**1**	**8**	**6.8**	**<0.5**

Note 1: Population at risk, number of cases (N) and DALYs are presented in thousands.

Note 2: Total skin disease is defined as the sum of the number of cases with palpable nodules, reversible and irreversible skin disease.

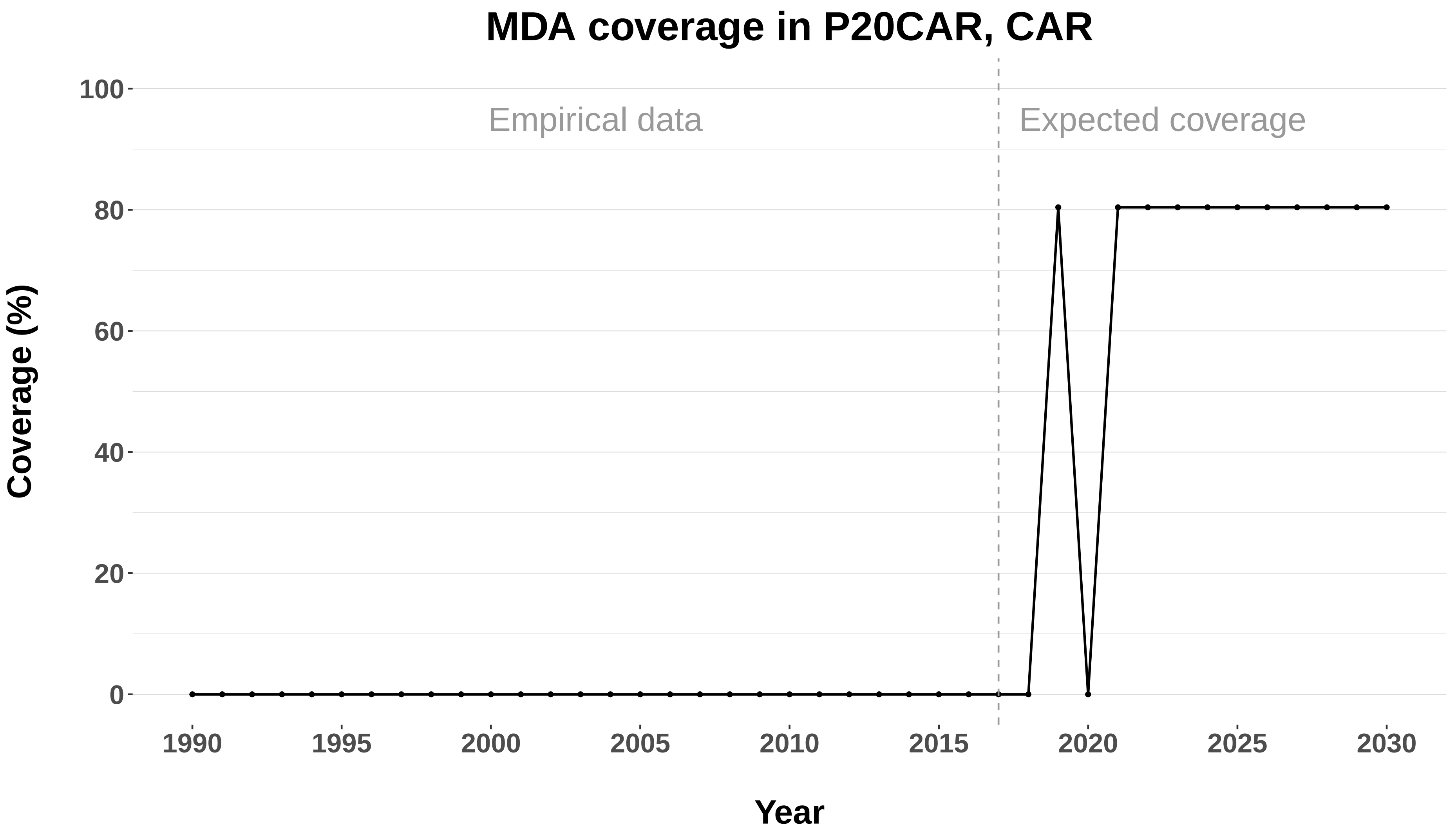
MDA coverage in P20CAR, CAR
Empirical data
Expected coverage
Coverage (%)
100
80
60
40
20
0
1990
1995
2000
2005
2010
2015
2020
2025
2030
Year

**Project, country:**	**P5CAR, CAR**
Bioclime:	
Endemicity level(s):	Hypoendemic
MDA start year:	2025
Mean MDA coverage per year:	65.0

	1990			2020			2030		
	N	%	DALYs	N	%	DALYs	N	%	DALYs
Total populaton at risk	88	100	_	198	100	_	260	100	_
**Infection:**									
Mf-positive	23	26.3	_	50	25.3	_	42	16.1	_
Mf-positive (age 5+)	23	26.3	_	50	25.2	_	42	16.1	_
Worm infected	30	34.6	_	66	33.5	_	72	27.7	_
**Clinical manifestations:**									
Palpable nodules	4	4.8	<0.5	9	4.6	<0.5	8	3.2	<0.5
Reversible skin disease:									
Severe itch	3	3.5	1	7	3.3	1	4	1.5	1
Reactive skin disease	2	2.4	<0.5	4	2.3	<0.5	1	0.4	<0.5
**Reversible skin disease**	**5**	**5.9**	**1**	**11**	**5.6**	**1**	**5**	**1.9**	**1**
Irreversible skin disease:									
Mild depigmentation	1	0.9	<0.5	2	0.8	<0.5	2	0.7	<0.5
Severe depigmentation	1	0.8	<0.5	2	0.8	<0.5	2	0.6	<0.5
Atrophy	<0.5	<0.05	<0.5	<0.5	<0.05	<0.5	<0.5	<0.05	<0.5
Hanging groin	<0.5	<0.05	<0.5	<0.5	<0.05	<0.5	<0.5	<0.05	<0.5
**Irreversible skin disease**	**2**	**1.7**	**<0.5**	**3**	**1.6**	**<0.5**	**3**	**1.3**	**<0.5**
**Total skin disease (OSD)**	**11**	**12.4**	**1**	**23**	**11.8**	**2**	**17**	**6.5**	**1**
Eye disease (OED):									
Visual impairment	<0.5	<0.05	<0.5	<0.5	<0.05	<0.5	<0.5	<0.05	<0.5
Blindness	<0.5	<0.05	<0.5	<0.5	<0.05	<0.5	<0.5	<0.05	<0.5
**Eye disease (OED)**	**<0.5**	**<0.05**	**<0.5**	**<0.5**	**<0.05**	**<0.5**	**<0.5**	**<0.05**	**<0.5**
**Total all manifestations**	**11**	**12.5**	**1**	**24**	**11.9**	**2**	**17**	**6.5**	**1**

Note 1: Population at risk, number of cases (N) and DALYs are presented in thousands.

Note 2: Total skin disease is defined as the sum of the number of cases with palpable nodules, reversible and irreversible skin disease.

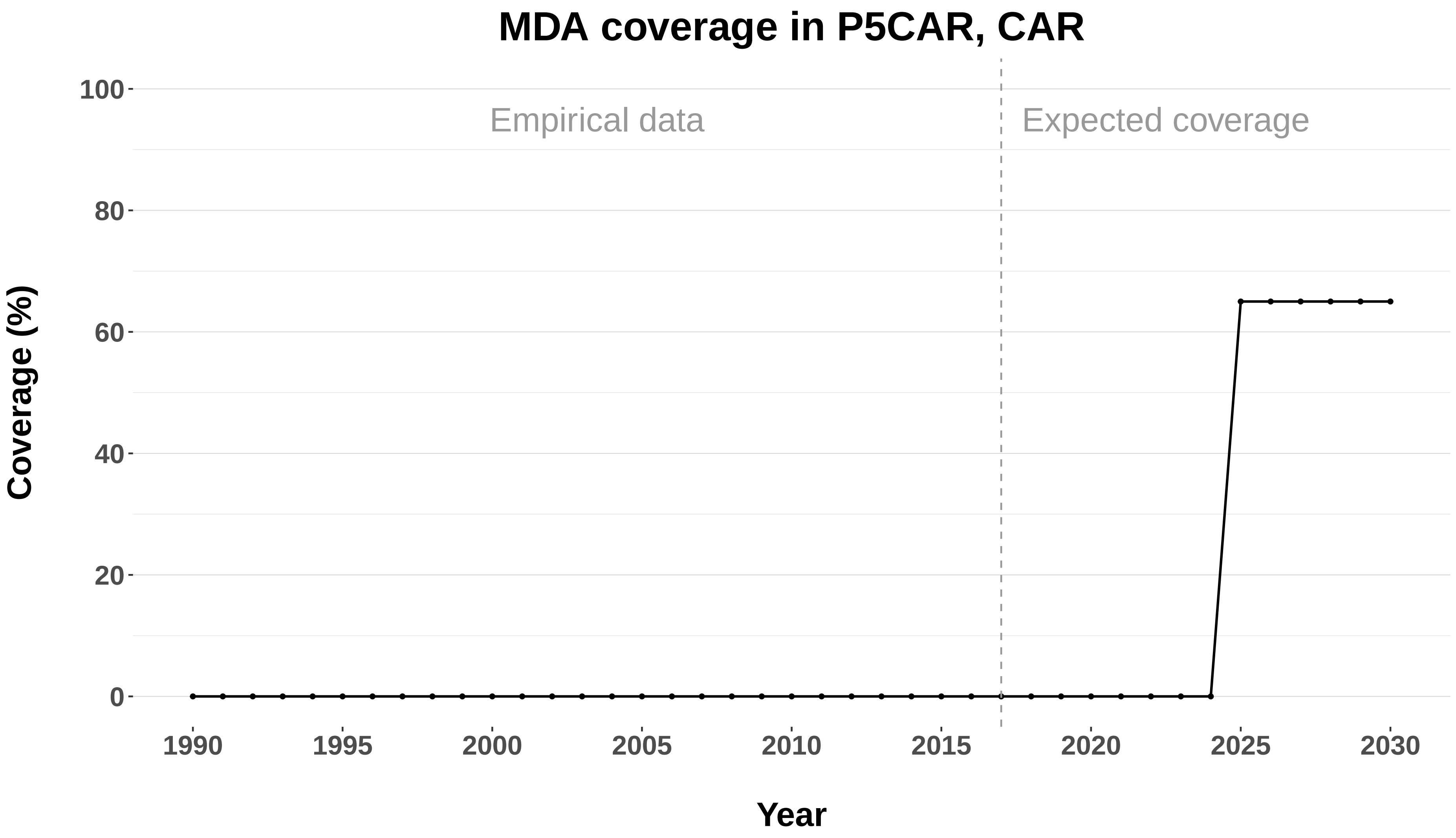
MDA coverage in P5CAR, CAR
Empirical data
Expected coverage
Coverage (%)
100
80
60
40
20
0
1990
1995
2000
2005
2010
2015
2020
2025
2030
Year

**Project, country:**	**Chad, Chad**
Bioclime:	
Endemicity level(s):	Hyperendemic
MDA start year:	1998
Mean MDA coverage per year:	72.2

	1990			2020			2030		
	N	%	DALYs	N	%	DALYs	N	%	DALYs
Total populaton at risk	1,068	100	_	2,409	100	_	3,160	100	_
**Infection:**									
Mf-positive	326	30.5	_	1	<0.05	_	<0.5	<0.05	_
Mf-positive (age 5+)	325	30.5	_	1	<0.05	_	<0.5	<0.05	_
Worm infected	425	39.8	_	29	1.2	_	<0.5	<0.05	_
**Clinical manifestations:**									
Palpable nodules	75	7.1	1	<0.5	<0.05	<0.5	<0.5	<0.05	<0.5
Reversible skin disease:									
Severe itch	42	4.0	8	<0.5	<0.05	<0.5	<0.5	<0.05	<0.5
Reactive skin disease	29	2.8	1	<0.5	<0.05	<0.5	<0.5	<0.05	<0.5
**Reversible skin disease**	**72**	**6.7**	**9**	**<0.5**	**<0.05**	**<0.5**	**<0.5**	**<0.05**	**<0.5**
Irreversible skin disease:									
Mild depigmentation	10	1.0	<0.5	8	0.3	<0.5	5	0.2	<0.5
Severe depigmentation	9	0.9	1	6	0.2	<0.5	3	0.1	<0.5
Atrophy	<0.5	<0.05	<0.5	<0.5	<0.05	<0.5	<0.5	<0.05	<0.5
Hanging groin	<0.5	<0.05	<0.5	<0.5	<0.05	<0.5	<0.5	<0.05	<0.5
**Irreversible skin disease**	**20**	**1.8**	**1**	**14**	**0.6**	**<0.5**	**8**	**0.3**	**<0.5**
**Total skin disease (OSD)**	**167**	**15.6**	**11**	**14**	**0.6**	**<0.5**	**8**	**0.3**	**<0.5**
Eye disease (OED):									
Visual impairment	18	1.7	1	12	0.5	<0.5	7	0.2	<0.5
Blindness	6	0.6	46	2	0.1	14	1	<0.05	5
**Eye disease (OED)**	**25**	**2.3**	**47**	**14**	**0.6**	**14**	**8**	**0.3**	**6**
**Total all manifestations**	**192**	**17.9**	**57**	**28**	**1.2**	**15**	**16**	**0.5**	**6**

Note 1: Population at risk, number of cases (N) and DALYs are presented in thousands.

Note 2: Total skin disease is defined as the sum of the number of cases with palpable nodules, reversible and irreversible skin disease.

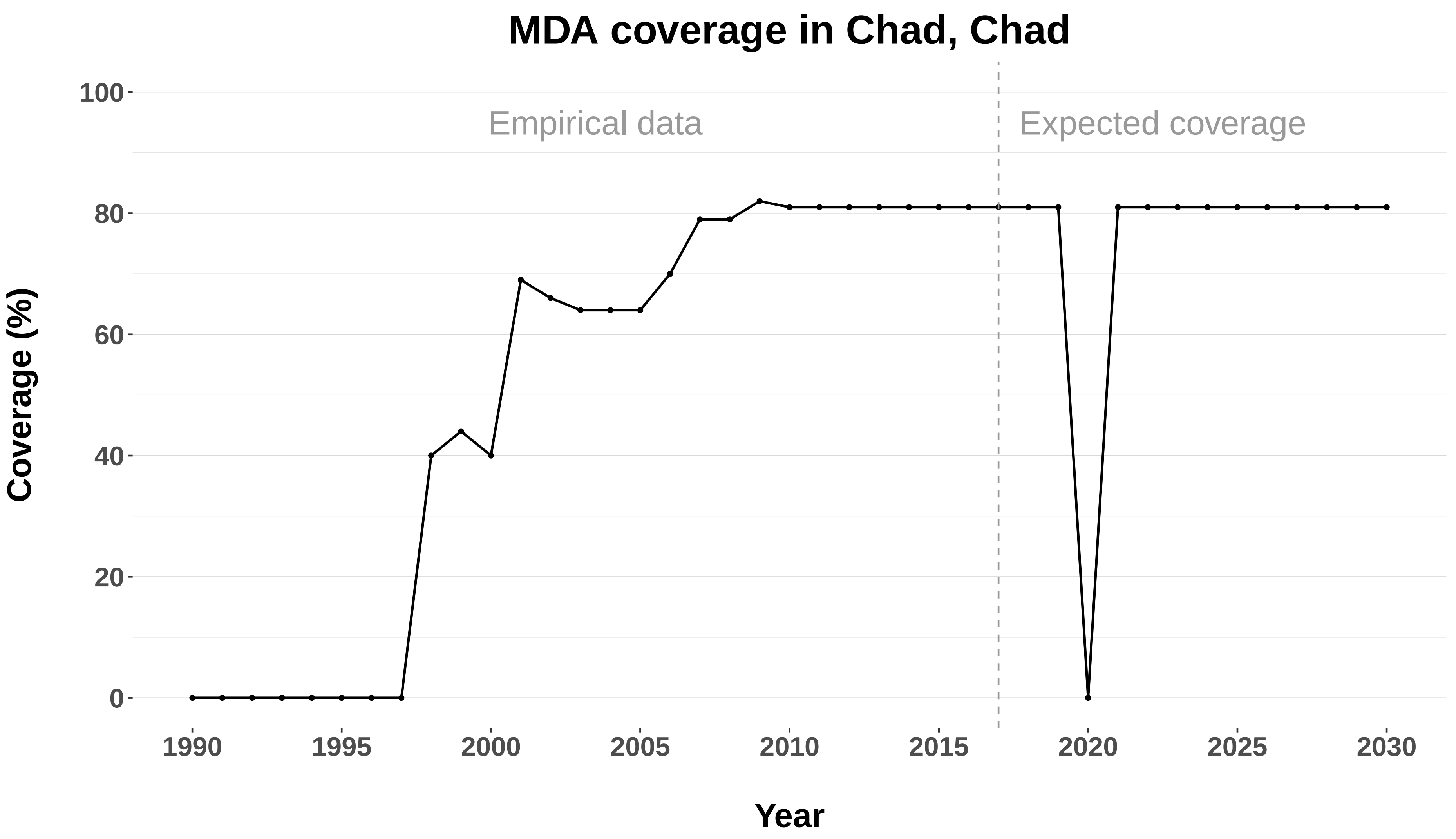
MDA coverage in Chad, Chad
Empirical data
Expected coverage
Coverage (%)
100
80
60
40
20
0
1990
1995
2000
2005
2010
2015
2020
2025
2030
Year

**Project, country:**	**P5Chad, Chad**
Bioclime:	
Endemicity level(s):	Hypoendemic
MDA start year:	2025
Mean MDA coverage per year:	65.0

	1990			2020			2030		
	N	%	DALYs	N	%	DALYs	N	%	DALYs
Total populaton at risk	133	100	_	300	100	_	393	100	_
**Infection:**									
Mf-positive	36	27.3	_	74	24.7	_	59	15.0	_
Mf-positive (age 5+)	36	27.3	_	74	24.7	_	59	15.0	_
Worm infected	50	37.3	_	103	34.3	_	109	27.7	_
**Clinical manifestations:**									
Palpable nodules	8	5.8	<0.5	16	5.3	<0.5	14	3.6	<0.5
Reversible skin disease:									
Severe itch	4	3.3	1	9	3.0	2	5	1.3	1
Reactive skin disease	3	2.3	<0.5	6	2.0	<0.5	1	0.4	<0.5
**Reversible skin disease**	**7**	**5.6**	**1**	**15**	**5.1**	**2**	**7**	**1.7**	**1**
Irreversible skin disease:									
Mild depigmentation	1	0.9	<0.5	2	0.8	<0.5	3	0.6	<0.5
Severe depigmentation	1	0.7	<0.5	2	0.6	<0.5	2	0.5	<0.5
Atrophy	<0.5	<0.05	<0.5	<0.5	<0.05	<0.5	<0.5	<0.05	<0.5
Hanging groin	<0.5	<0.05	<0.5	<0.5	<0.05	<0.5	<0.5	<0.05	<0.5
**Irreversible skin disease**	**2**	**1.5**	**<0.5**	**4**	**1.4**	**<0.5**	**4**	**1.1**	**<0.5**
**Total skin disease (OSD)**	**17**	**13.0**	**1**	**35**	**11.8**	**2**	**25**	**6.4**	**1**
Eye disease (OED):									
Visual impairment	2	1.4	<0.5	4	1.3	<0.5	4	1.0	<0.5
Blindness	1	0.5	4	1	0.4	9	1	0.3	8
**Eye disease (OED)**	**3**	**1.9**	**4**	**5**	**1.7**	**9**	**5**	**1.3**	**8**
**Total all manifestations**	**20**	**14.8**	**6**	**40**	**13.5**	**12**	**30**	**7.7**	**10**

Note 1: Population at risk, number of cases (N) and DALYs are presented in thousands.

Note 2: Total skin disease is defined as the sum of the number of cases with palpable nodules, reversible and irreversible skin disease.

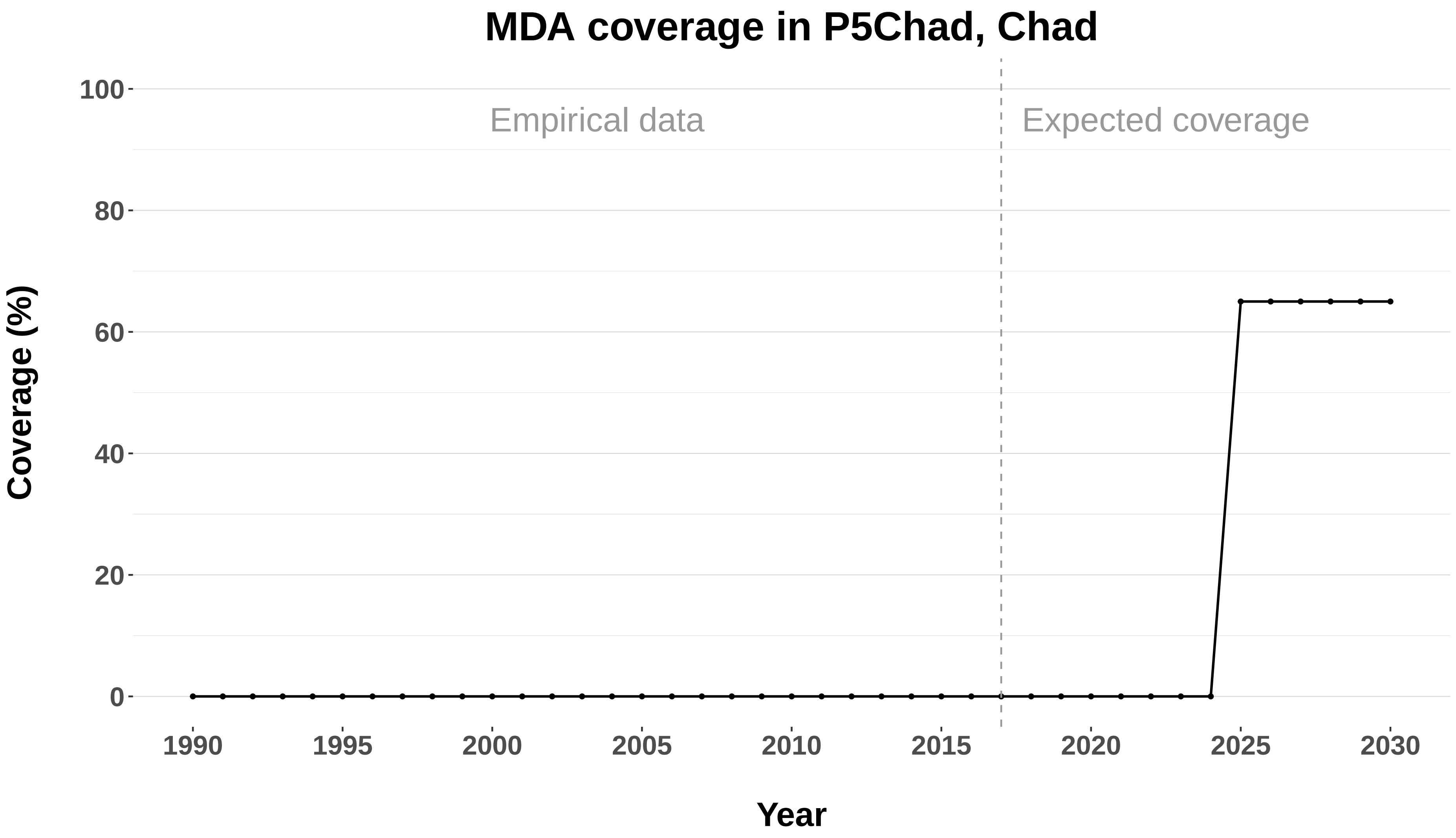

MDA coverage in P5Chad, Chad
Empirical data
Expected coverage
Coverage (%)
100
80
60
40
20
0
1990
1995
2000
2005
2010
2015
2020
2025
2030
Year

**Project, country:**	**P5Chad propext, Chad**
Bioclime:	
Endemicity level(s):	Hypoendemic
MDA start year:	2025
Mean MDA coverage per year:	65.0

	1990			2020			2030		
	N	%	DALYs	N	%	DALYs	N	%	DALYs
Total populaton at risk	76	100	_	172	100	_	226	100	_
**Infection:**									
Mf-positive	17	22.3	_	34	20.0	_	27	11.7	_
Mf-positive (age 5+)	17	22.3	_	34	19.9	_	27	11.7	_
Worm infected	24	30.8	_	49	28.2	_	51	22.5	_
**Clinical manifestations:**									
Palpable nodules	2	3.1	<0.5	5	2.7	<0.5	4	1.8	<0.5
Reversible skin disease:									
Severe itch	2	2.6	<0.5	4	2.3	1	2	1.0	<0.5
Reactive skin disease	1	1.7	<0.5	3	1.5	<0.5	1	0.2	<0.5
**Reversible skin disease**	**3**	**4.3**	**<0.5**	**7**	**3.8**	**1**	**3**	**1.2**	**<0.5**
Irreversible skin disease:									
Mild depigmentation	1	0.7	<0.5	1	0.6	<0.5	1	0.5	<0.5
Severe depigmentation	<0.5	0.5	<0.5	1	0.5	<0.5	1	0.4	<0.5
Atrophy	<0.5	<0.05	<0.5	<0.5	<0.05	<0.5	<0.5	<0.05	<0.5
Hanging groin	<0.5	<0.05	<0.5	<0.5	<0.05	<0.5	<0.5	<0.05	<0.5
**Irreversible skin disease**	**1**	**1.2**	**<0.5**	**2**	**1.1**	**<0.5**	**2**	**0.9**	**<0.5**
**Total skin disease (OSD)**	**7**	**8.6**	**<0.5**	**13**	**7.6**	**1**	**9**	**3.9**	**1**
Eye disease (OED):									
Visual impairment	1	1.2	<0.5	2	1.0	<0.5	2	0.8	<0.5
Blindness	<0.5	0.3	2	1	0.3	3	<0.5	0.2	3
**Eye disease (OED)**	**1**	**1.5**	**2**	**2**	**1.3**	**4**	**2**	**1.0**	**3**
**Total all manifestations**	**8**	**10.0**	**2**	**15**	**8.9**	**5**	**11**	**4.9**	**4**

Note 1: Population at risk, number of cases (N) and DALYs are presented in thousands.

Note 2: Total skin disease is defined as the sum of the number of cases with palpable nodules, reversible and irreversible skin disease.

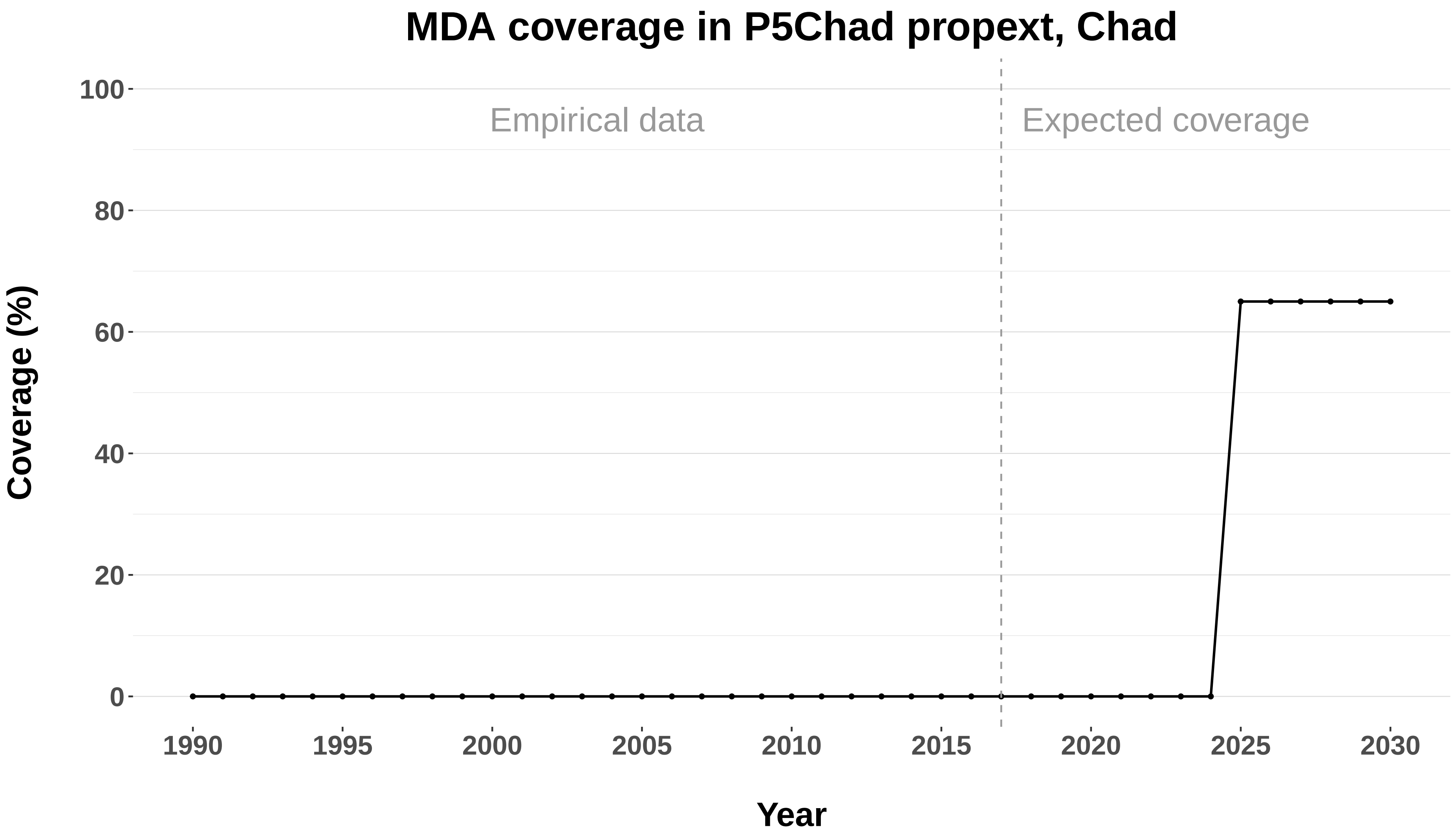
MDA coverage in P5Chad propext, Chad
Empirical data
Expected coverage
Coverage (%)
100
80
60
40
20
0
1990
1995
2000
2005
2010
2015
2020
2025
2030
Year

**Project, country:**	**Congo 1, Congo**
Bioclime:	
Endemicity level(s):	Hyperendemic
MDA start year:	2001
Mean MDA coverage per year:	70.4

	1990			2020			2030		
	N	%	DALYs	N	%	DALYs	N	%	DALYs
Total populaton at risk	487	100	_	1,099	100	_	1,441	100	_
**Infection:**									
Mf-positive	133	27.3	_	3	0.3	_	<0.5	<0.05	_
Mf-positive (age 5+)	133	27.3	_	3	0.3	_	<0.5	<0.05	_
Worm infected	175	36.0	_	40	3.6	_	1	0.1	_
**Clinical manifestations:**									
Palpable nodules	27	5.5	<0.5	1	0.1	<0.5	<0.5	<0.05	<0.5
Reversible skin disease:									
Severe itch	17	3.6	3	<0.5	<0.05	<0.5	<0.5	<0.05	<0.5
Reactive skin disease	12	2.5	1	<0.5	<0.05	<0.5	<0.5	<0.05	<0.5
**Reversible skin disease**	**29**	**6.0**	**4**	**<0.5**	**<0.05**	**<0.5**	**<0.5**	**<0.05**	**<0.5**
Irreversible skin disease:									
Mild depigmentation	4	0.9	<0.5	4	0.4	<0.5	3	0.2	<0.5
Severe depigmentation	4	0.8	<0.5	3	0.3	<0.5	2	0.1	<0.5
Atrophy	<0.5	<0.05	<0.5	<0.5	<0.05	<0.5	<0.5	<0.05	<0.5
Hanging groin	<0.5	<0.05	<0.5	<0.5	<0.05	<0.5	<0.5	<0.05	<0.5
**Irreversible skin disease**	**8**	**1.7**	**<0.5**	**7**	**0.7**	**<0.5**	**5**	**0.3**	**<0.5**
**Total skin disease (OSD)**	**64**	**13.2**	**4**	**9**	**0.8**	**<0.5**	**5**	**0.3**	**<0.5**
Eye disease (OED):									
Visual impairment	<0.5	<0.05	<0.5	<0.5	<0.05	<0.5	<0.5	<0.05	<0.5
Blindness	<0.5	<0.05	1	<0.5	<0.05	<0.5	<0.5	<0.05	<0.5
**Eye disease (OED)**	**<0.5**	**<0.05**	**1**	**<0.5**	**<0.05**	**<0.5**	**<0.5**	**<0.05**	**<0.5**
**Total all manifestations**	**65**	**13.3**	**5**	**9**	**0.8**	**1**	**5**	**0.3**	**<0.5**

Note 1: Population at risk, number of cases (N) and DALYs are presented in thousands.

Note 2: Total skin disease is defined as the sum of the number of cases with palpable nodules, reversible and irreversible skin disease.

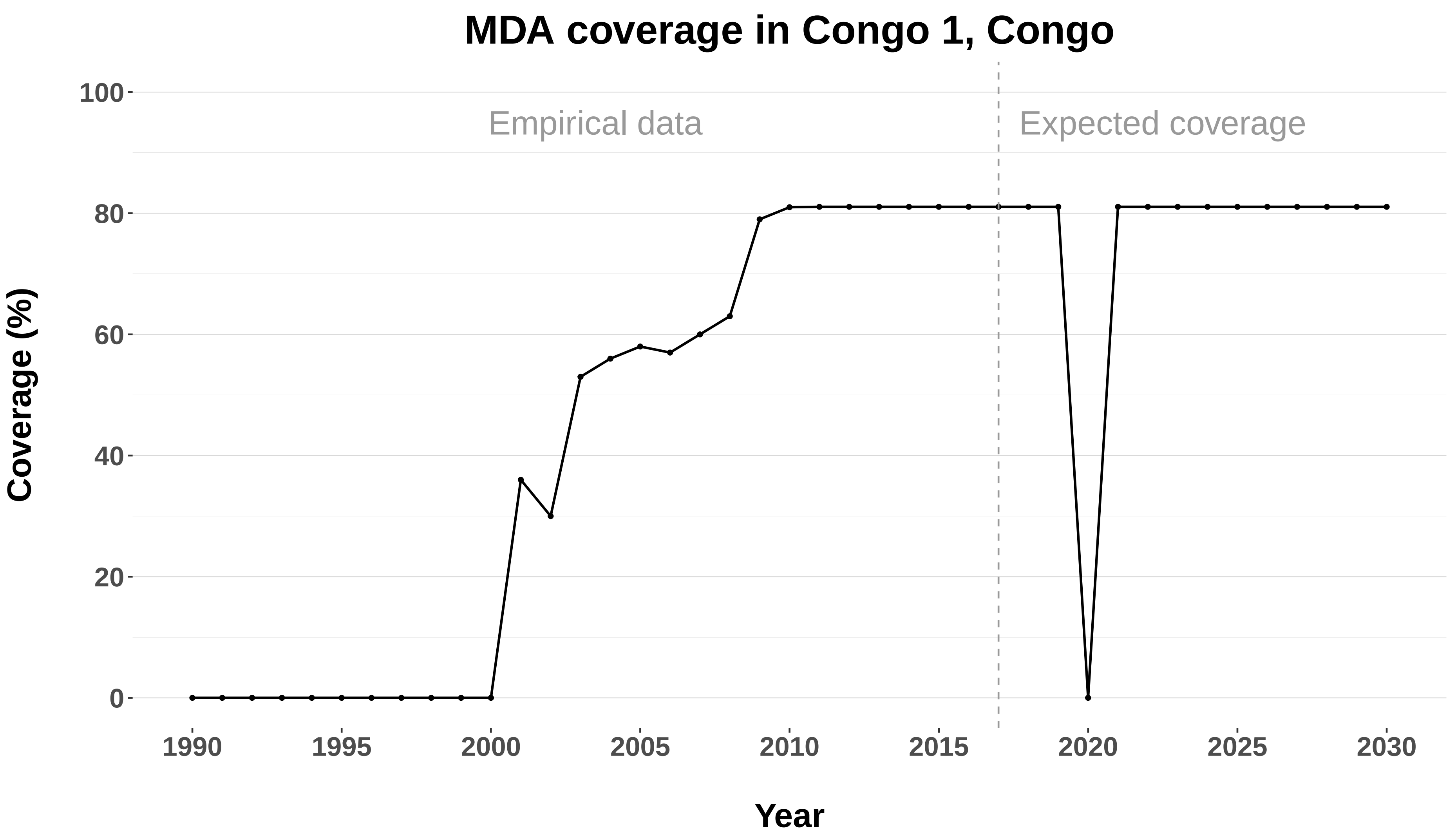
MDA coverage in Congo 1, Congo
Empirical data
Expected coverage
Coverage (%)
100
80
60
40
20
0
1990
1995
2000
2005
2010
2015
2020
2025
2030
Year

**Project, country:**	**P20Congo, Congo**
Bioclime:	
Endemicity level(s):	Hyperendemic
MDA start year:	2015
Mean MDA coverage per year:	76.0

	1990			2020			2030		
	N	%	DALYs	N	%	DALYs	N	%	DALYs
Total populaton at risk	21	100	_	48	100	_	63	100	_
**Infection:**									
Mf-positive	12	56.7	_	18	37.4	_	2	2.7	_
Mf-positive (age 5+)	12	56.6	_	18	37.4	_	2	2.7	_
Worm infected	14	65.9	_	27	56.6	_	11	16.7	_
**Clinical manifestations:**									
Palpable nodules	5	24.7	<0.5	8	17.3	<0.5	1	1.1	<0.5
Reversible skin disease:									
Severe itch	2	10.5	<0.5	2	4.5	<0.5	<0.5	0.2	<0.5
Reactive skin disease	2	8.2	<0.5	<0.5	1.0	<0.5	<0.5	<0.05	<0.5
**Reversible skin disease**	**4**	**18.7**	**<0.5**	**3**	**5.5**	**<0.5**	**<0.5**	**0.2**	**<0.5**
Irreversible skin disease:									
Mild depigmentation	<0.5	2.2	<0.5	1	1.8	<0.5	1	1.0	<0.5
Severe depigmentation	1	2.7	<0.5	1	2.2	<0.5	1	1.2	<0.5
Atrophy	<0.5	<0.05	<0.5	<0.5	<0.05	<0.5	<0.5	<0.05	<0.5
Hanging groin	<0.5	<0.05	<0.5	<0.5	<0.05	<0.5	<0.5	<0.05	<0.5
**Irreversible skin disease**	**1**	**5.0**	**<0.5**	**2**	**4.0**	**<0.5**	**1**	**2.3**	**<0.5**
**Total skin disease (OSD)**	**10**	**48.3**	**1**	**13**	**26.8**	**1**	**2**	**3.6**	**<0.5**
Eye disease (OED):									
Visual impairment	<0.5	0.1	<0.5	<0.5	0.1	<0.5	<0.5	<0.05	<0.5
Blindness	<0.5	0.1	<0.5	<0.5	0.1	<0.5	<0.5	<0.05	<0.5
**Eye disease (OED)**	**<0.5**	**0.3**	**<0.5**	**<0.5**	**0.2**	**<0.5**	**<0.5**	**0.1**	**<0.5**
**Total all manifestations**	**10**	**48.6**	**1**	**13**	**26.9**	**1**	**2**	**3.6**	**<0.5**

Note 1: Population at risk, number of cases (N) and DALYs are presented in thousands.

Note 2: Total skin disease is defined as the sum of the number of cases with palpable nodules, reversible and irreversible skin disease.

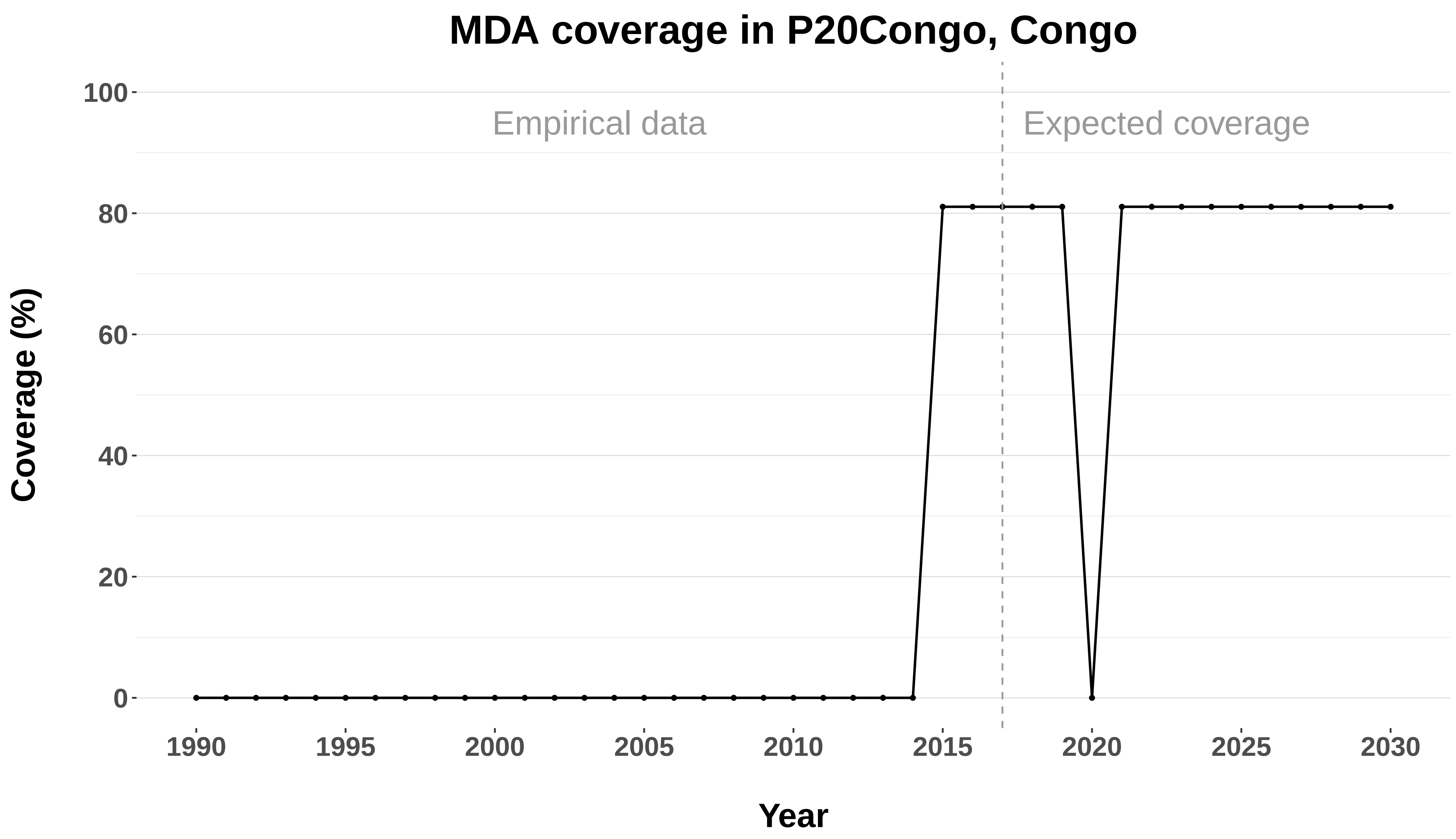

MDA coverage in P20Congo, Congo
Empirical data
Expected coverage
Coverage (%)
100
80
60
40
20
0
1990
1995
2000
2005
2010
2015
2020
2025
2030
Year

**Project, country:**	**P5Congo, Congo**
Bioclime:	
Endemicity level(s):	Hypoendemic
MDA start year:	2025
Mean MDA coverage per year:	65.0

	1990			2020			2030		
	N	%	DALYs	N	%	DALYs	N	%	DALYs
Total populaton at risk	286	100	_	645	100	_	846	100	_
**Infection:**									
Mf-positive	75	26.4	_	166	25.7	_	142	16.8	_
Mf-positive (age 5+)	75	26.4	_	165	25.7	_	142	16.8	_
Worm infected	97	34.1	_	214	33.2	_	237	28.0	_
**Clinical manifestations:**									
Palpable nodules	14	4.8	<0.5	31	4.7	<0.5	29	3.4	<0.5
Reversible skin disease:									
Severe itch	11	3.7	2	23	3.6	4	14	1.6	3
Reactive skin disease	7	2.6	<0.5	16	2.5	1	4	0.5	<0.5
**Reversible skin disease**	**18**	**6.3**	**2**	**40**	**6.1**	**5**	**18**	**2.1**	**3**
Irreversible skin disease:									
Mild depigmentation	3	0.9	<0.5	6	0.9	<0.5	6	0.8	<0.5
Severe depigmentation	3	0.9	<0.5	6	0.9	<0.5	6	0.7	<0.5
Atrophy	<0.5	<0.05	<0.5	<0.5	<0.05	<0.5	<0.5	<0.05	<0.5
Hanging groin	<0.5	<0.05	<0.5	<0.5	<0.05	<0.5	<0.5	<0.05	<0.5
**Irreversible skin disease**	**5**	**1.8**	**<0.5**	**12**	**1.8**	**<0.5**	**12**	**1.5**	**<0.5**
**Total skin disease (OSD)**	**37**	**13.0**	**3**	**82**	**12.7**	**6**	**59**	**7.0**	**3**
Eye disease (OED):									
Visual impairment	<0.5	<0.05	<0.5	<0.5	<0.05	<0.5	<0.5	<0.05	<0.5
Blindness	<0.5	<0.05	<0.5	<0.5	<0.05	1	<0.5	<0.05	1
**Eye disease (OED)**	**<0.5**	**0.1**	**<0.5**	**<0.5**	**<0.05**	**1**	**<0.5**	**<0.05**	**1**
**Total all manifestations**	**37**	**13.0**	**3**	**82**	**12.7**	**6**	**59**	**7.0**	**4**

Note 1: Population at risk, number of cases (N) and DALYs are presented in thousands.

Note 2: Total skin disease is defined as the sum of the number of cases with palpable nodules, reversible and irreversible skin disease.

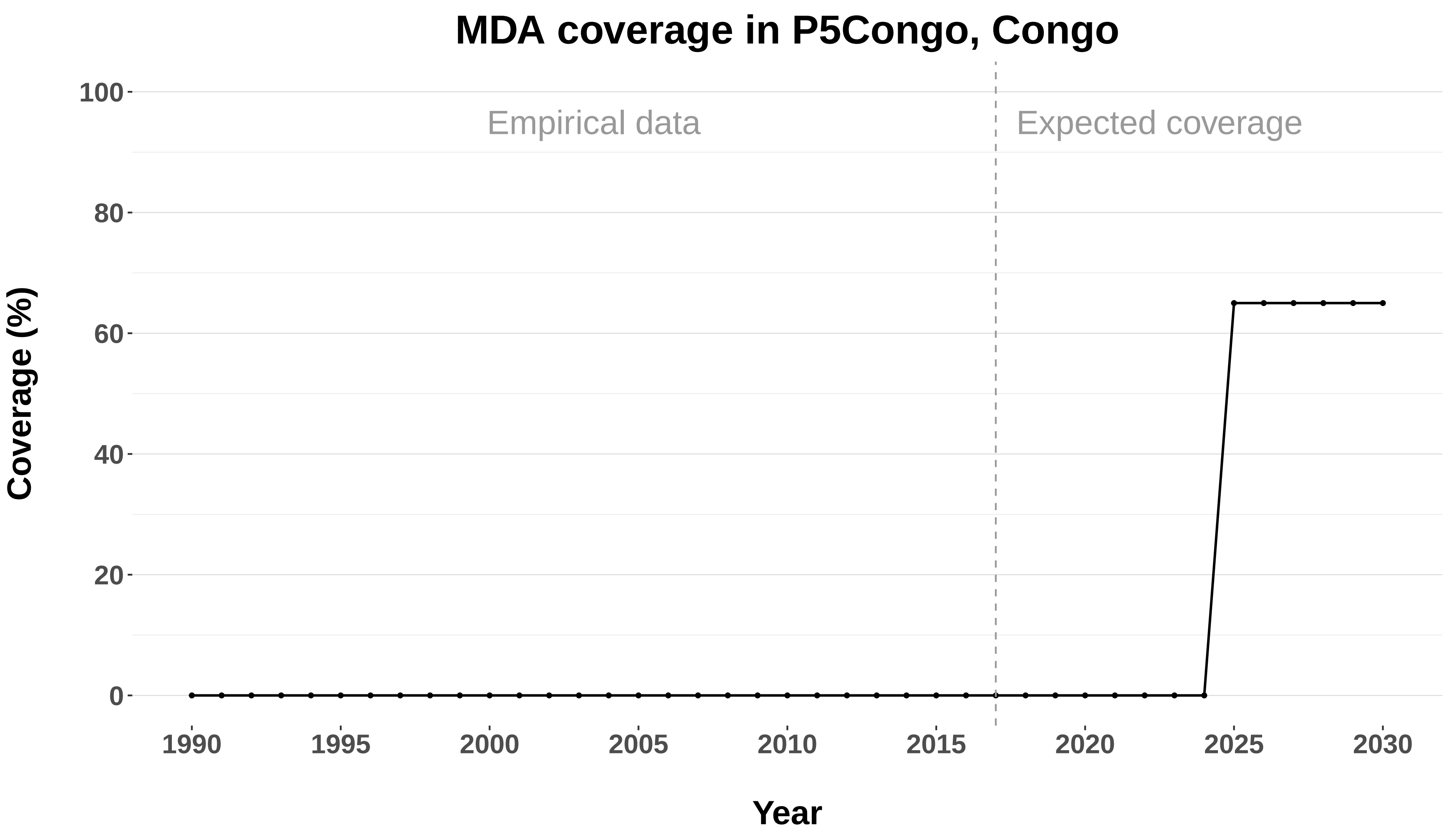
MDA coverage in P5Congo, Congo
Empirical data
Expected coverage
Coverage (%)
0
20
40
60
80
100
1990
1995
2000
2005
2010
2015
2020
2025
2030
Year

**Project, country:**	**Bandundu, DRC**
Bioclime:	
Endemicity level(s):	Hyperendemic
MDA start year:	2003
Mean MDA coverage per year:	70.7

	1990			2020			2030		
	N	%	DALYs	N	%	DALYs	N	%	DALYs
Total populaton at risk	3	100	_	8	100	_	10	100	_
**Infection:**									
Mf-positive	1	27.1	_	<0.5	0.5	_	<0.5	<0.05	_
Mf-positive (age 5+)	1	27.1	_	<0.5	0.5	_	<0.5	<0.05	_
Worm infected	1	34.8	_	<0.5	5.6	_	<0.5	0.1	_
**Clinical manifestations:**									
Palpable nodules	<0.5	4.9	<0.5	<0.5	0.2	<0.5	<0.5	<0.05	<0.5
Reversible skin disease:									
Severe itch	<0.5	3.7	<0.5	<0.5	0.1	<0.5	<0.5	<0.05	<0.5
Reactive skin disease	<0.5	2.6	<0.5	<0.5	<0.05	<0.5	<0.5	<0.05	<0.5
**Reversible skin disease**	**<0.5**	**6.3**	**<0.5**	**<0.5**	**0.1**	**<0.5**	**<0.5**	**<0.05**	**<0.5**
Irreversible skin disease:									
Mild depigmentation	<0.5	1.0	<0.5	<0.5	0.4	<0.5	<0.5	0.2	<0.5
Severe depigmentation	<0.5	0.9	<0.5	<0.5	0.4	<0.5	<0.5	0.2	<0.5
Atrophy	<0.5	<0.05	<0.5	<0.5	<0.05	<0.5	<0.5	<0.05	<0.5
Hanging groin	<0.5	<0.05	<0.5	<0.5	<0.05	<0.5	<0.5	<0.05	<0.5
**Irreversible skin disease**	**<0.5**	**1.9**	**<0.5**	**<0.5**	**0.8**	**<0.5**	**<0.5**	**0.4**	**<0.5**
**Total skin disease (OSD)**	**<0.5**	**13.1**	**<0.5**	**<0.5**	**1.0**	**<0.5**	**<0.5**	**0.4**	**<0.5**
Eye disease (OED):									
Visual impairment	<0.5	<0.05	<0.5	<0.5	<0.05	<0.5	<0.5	<0.05	<0.5
Blindness	<0.5	<0.05	<0.5	<0.5	<0.05	<0.5	<0.5	<0.05	<0.5
**Eye disease (OED)**	**<0.5**	**<0.05**	**<0.5**	**<0.5**	**<0.05**	**<0.5**	**<0.5**	**<0.05**	**<0.5**
**Total all manifestations**	**<0.5**	**13.1**	**<0.5**	**<0.5**	**1.0**	**<0.5**	**<0.5**	**0.4**	**<0.5**

Note 1: Population at risk, number of cases (N) and DALYs are presented in thousands.

Note 2: Total skin disease is defined as the sum of the number of cases with palpable nodules, reversible and irreversible skin disease.

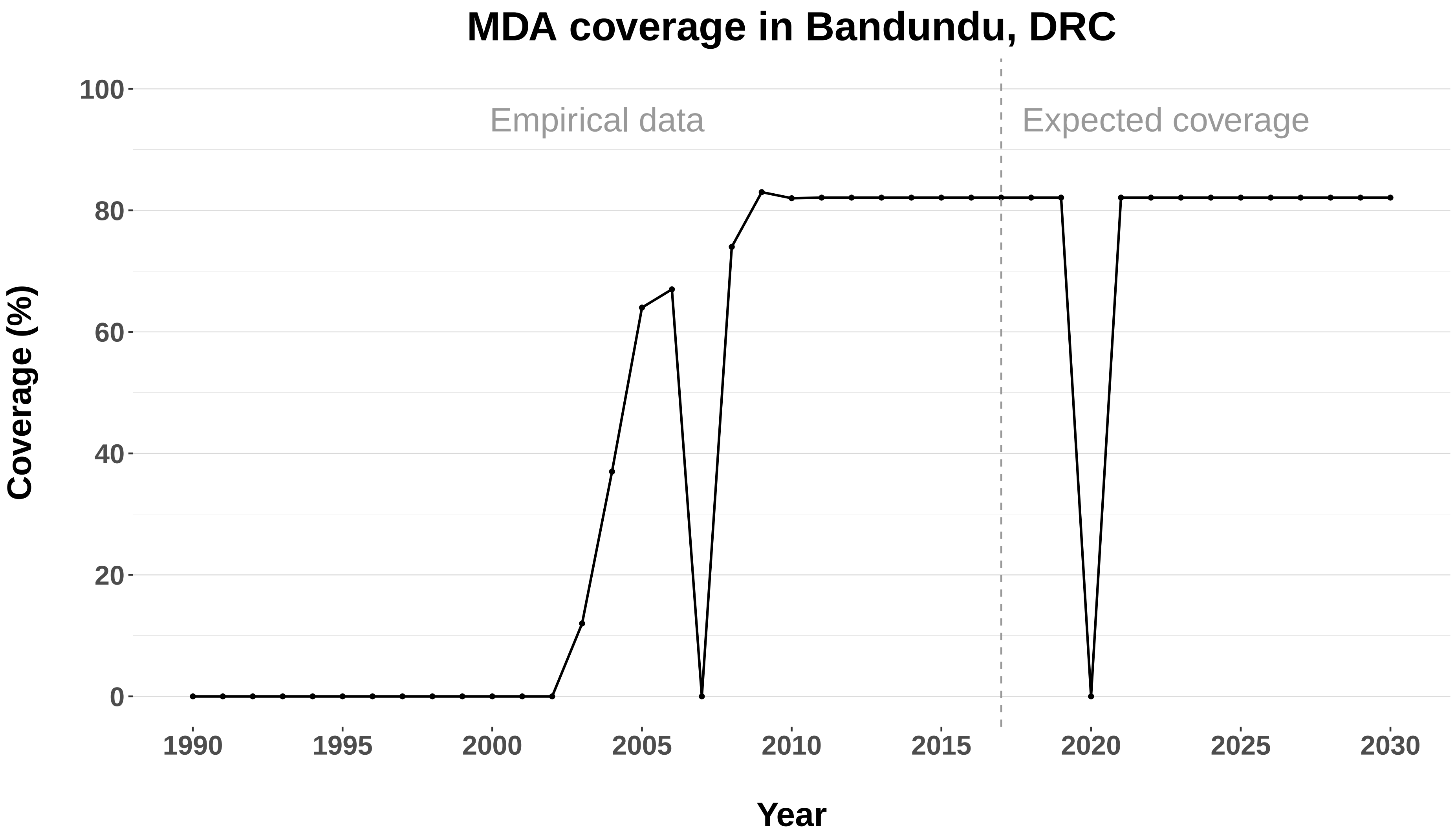
MDA coverage in Bandundu, DRC
Empirical data
Expected coverage
Coverage (%)
100
80
60
40
20
0
1990
1995
2000
2005
2010
2015
2020
2025
2030
Year

**Project, country:**	**Bas-Congo Kinshasa, DRC**
Bioclime:	
Endemicity level(s):	Hyperendemic
MDA start year:	2004
Mean MDA coverage per year:	61.9

	1990			2020			2030		
	N	%	DALYs	N	%	DALYs	N	%	DALYs
Total populaton at risk	803	100	_	1,813	100	_	2,378	100	_
**Infection:**									
Mf-positive	298	37.0	_	57	3.1	_	1	0.1	_
Mf-positive (age 5+)	297	37.0	_	57	3.1	_	1	0.1	_
Worm infected	373	46.4	_	251	13.8	_	29	1.2	_
**Clinical manifestations:**									
Palpable nodules	89	11.1	1	17	0.9	<0.5	1	<0.05	<0.5
Reversible skin disease:									
Severe itch	45	5.6	8	4	0.2	1	<0.5	<0.05	<0.5
Reactive skin disease	32	4.0	1	<0.5	<0.05	<0.5	<0.5	<0.05	<0.5
**Reversible skin disease**	**77**	**9.6**	**10**	**5**	**0.3**	**1**	**<0.5**	**<0.05**	**<0.5**
Irreversible skin disease:									
Mild depigmentation	10	1.3	<0.5	12	0.6	<0.5	8	0.3	<0.5
Severe depigmentation	11	1.3	1	11	0.6	1	7	0.3	<0.5
Atrophy	<0.5	<0.05	<0.5	<0.5	<0.05	<0.5	<0.5	<0.05	<0.5
Hanging groin	<0.5	<0.05	<0.5	<0.5	<0.05	<0.5	<0.5	<0.05	<0.5
**Irreversible skin disease**	**21**	**2.6**	**1**	**23**	**1.2**	**1**	**15**	**0.6**	**1**
**Total skin disease (OSD)**	**187**	**23.3**	**11**	**44**	**2.4**	**2**	**16**	**0.7**	**1**
Eye disease (OED):									
Visual impairment	<0.5	<0.05	<0.5	<0.5	<0.05	<0.5	<0.5	<0.05	<0.5
Blindness	<0.5	<0.05	2	<0.5	<0.05	1	<0.5	<0.05	<0.5
**Eye disease (OED)**	**1**	**0.1**	**2**	**<0.5**	**<0.05**	**1**	**<0.5**	**<0.05**	**<0.5**
**Total all manifestations**	**188**	**23.3**	**13**	**44**	**2.4**	**3**	**16**	**0.7**	**1**

Note 1: Population at risk, number of cases (N) and DALYs are presented in thousands.

Note 2: Total skin disease is defined as the sum of the number of cases with palpable nodules, reversible and irreversible skin disease.

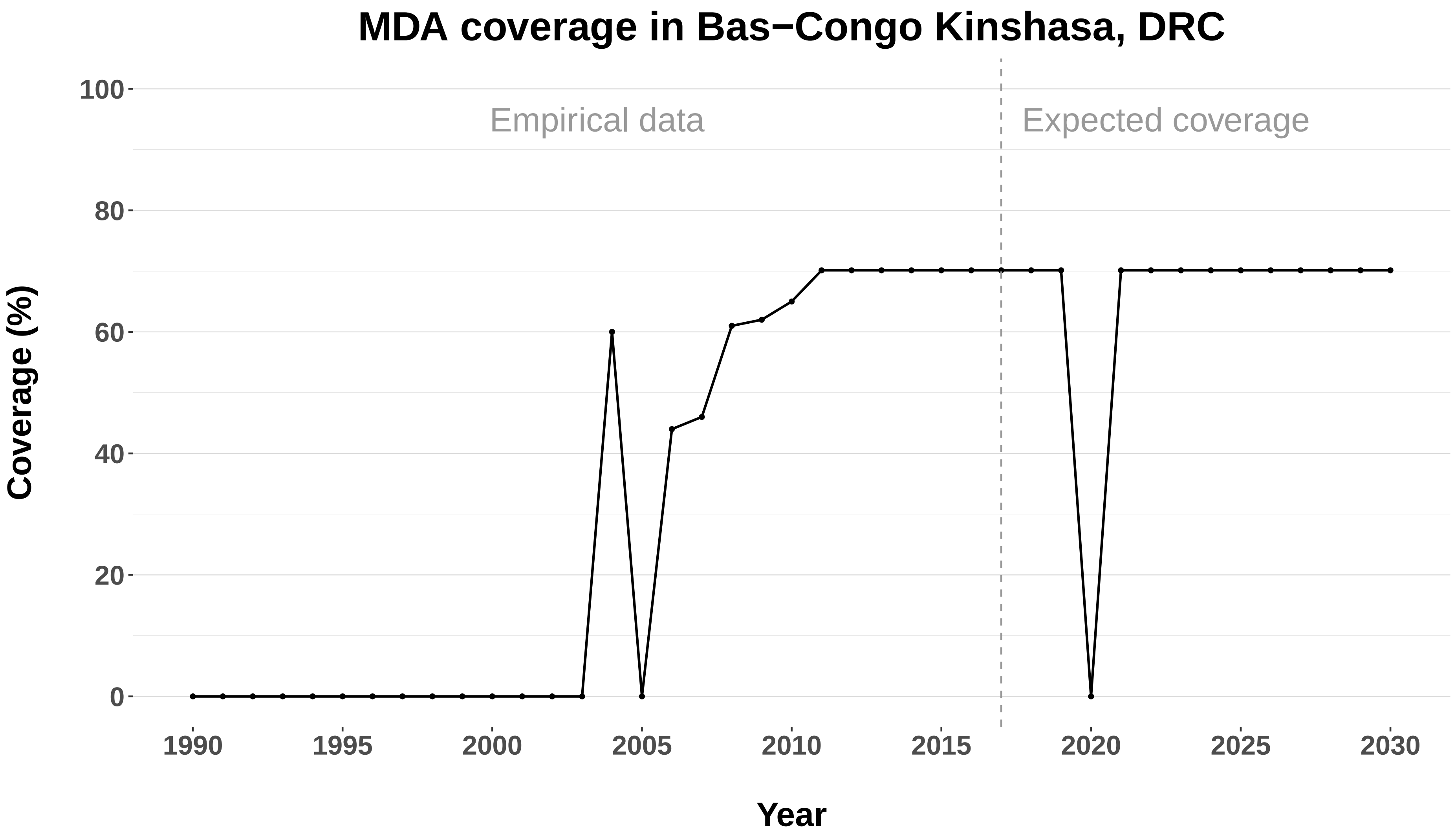
MDA coverage in Bas−Congo Kinshasa, DRC
Empirical data
Expected coverage
Coverage (%)
0
20
40
60
80
100
1990
1995
2000
2005
2010
2015
2020
2025
2030
Year

**Project, country:**	**Butembo-Beni, DRC**
Bioclime:	
Endemicity level(s):	Hyperendemic
MDA start year:	2008
Mean MDA coverage per year:	61.2

	1990			2020			2030		
	N	%	DALYs	N	%	DALYs	N	%	DALYs
Total populaton at risk	499	100	_	1,127	100	_	1,478	100	_
**Infection:**									
Mf-positive	215	43.1	_	127	11.3	_	11	0.7	_
Mf-positive (age 5+)	215	43.1	_	127	11.3	_	11	0.7	_
Worm infected	266	53.3	_	326	29.0	_	77	5.2	_
**Clinical manifestations:**									
Palpable nodules	76	15.1	1	45	4.0	<0.5	3	0.2	<0.5
Reversible skin disease:									
Severe itch	34	6.8	6	11	1.0	2	1	<0.05	<0.5
Reactive skin disease	26	5.1	1	2	0.2	<0.5	<0.5	<0.05	<0.5
**Reversible skin disease**	**60**	**12.0**	**7**	**13**	**1.2**	**2**	**1**	**<0.05**	**<0.5**
Irreversible skin disease:									
Mild depigmentation	7	1.5	<0.5	11	1.0	<0.5	8	0.5	<0.5
Severe depigmentation	8	1.7	1	11	1.0	1	7	0.5	<0.5
Atrophy	<0.5	<0.05	<0.5	<0.5	<0.05	<0.5	<0.5	<0.05	<0.5
Hanging groin	<0.5	<0.05	<0.5	<0.5	<0.05	<0.5	<0.5	<0.05	<0.5
**Irreversible skin disease**	**16**	**3.2**	**1**	**22**	**2.0**	**1**	**15**	**1.0**	**1**
**Total skin disease (OSD)**	**151**	**30.3**	**9**	**81**	**7.1**	**4**	**20**	**1.3**	**1**
Eye disease (OED):									
Visual impairment	<0.5	0.1	<0.5	<0.5	<0.05	<0.5	<0.5	<0.05	<0.5
Blindness	<0.5	0.1	2	<0.5	<0.05	2	<0.5	<0.05	1
**Eye disease (OED)**	**1**	**0.1**	**2**	**1**	**0.1**	**2**	**<0.5**	**<0.05**	**1**
**Total all manifestations**	**152**	**30.4**	**11**	**81**	**7.2**	**5**	**20**	**1.4**	**1**

Note 1: Population at risk, number of cases (N) and DALYs are presented in thousands.

Note 2: Total skin disease is defined as the sum of the number of cases with palpable nodules, reversible and irreversible skin disease.

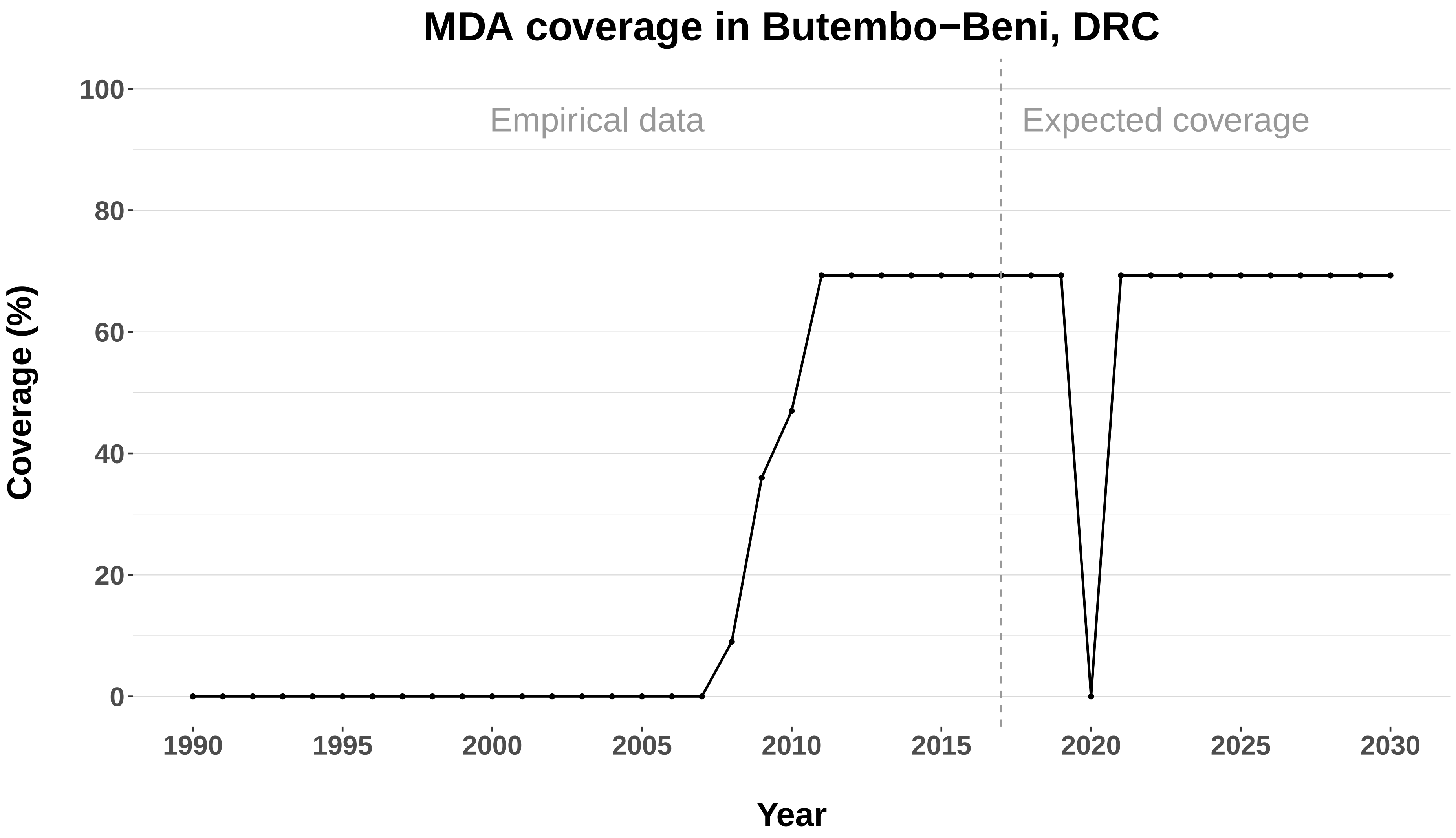
MDA coverage in Butembo−Beni, DRC
Empirical data
Expected coverage
Coverage (%)
100
80
60
40
20
0
1990
1995
2000
2005
2010
2015
2020
2025
2030
Year

**Project, country:**	**Equateur-Kiri, DRC**
Bioclime:	
Endemicity level(s):	Hyperendemic
MDA start year:	2006
Mean MDA coverage per year:	69.9

	1990			2020			2030		
	N	%	DALYs	N	%	DALYs	N	%	DALYs
Total populaton at risk	664	100	_	1,497	100	_	1,964	100	_
**Infection:**									
Mf-positive	272	40.9	_	52	3.5	_	2	0.1	_
Mf-positive (age 5+)	271	40.9	_	52	3.5	_	2	0.1	_
Worm infected	339	51.1	_	253	16.9	_	23	1.2	_
**Clinical manifestations:**									
Palpable nodules	89	13.4	1	21	1.4	<0.5	1	<0.05	<0.5
Reversible skin disease:									
Severe itch	42	6.3	8	5	0.4	1	<0.5	<0.05	<0.5
Reactive skin disease	30	4.6	1	<0.5	<0.05	<0.5	<0.5	<0.05	<0.5
**Reversible skin disease**	**72**	**10.9**	**9**	**6**	**0.4**	**1**	**<0.5**	**<0.05**	**<0.5**
Irreversible skin disease:									
Mild depigmentation	9	1.4	<0.5	12	0.8	<0.5	8	0.4	<0.5
Severe depigmentation	10	1.5	1	11	0.8	1	7	0.4	<0.5
Atrophy	<0.5	<0.05	<0.5	<0.5	<0.05	<0.5	<0.5	<0.05	<0.5
Hanging groin	<0.5	<0.05	<0.5	<0.5	<0.05	<0.5	<0.5	<0.05	<0.5
**Irreversible skin disease**	**19**	**2.9**	**1**	**23**	**1.5**	**1**	**16**	**0.8**	**1**
**Total skin disease (OSD)**	**181**	**27.2**	**11**	**50**	**3.3**	**2**	**17**	**0.9**	**1**
Eye disease (OED):									
Visual impairment	<0.5	0.1	<0.5	<0.5	<0.05	<0.5	<0.5	<0.05	<0.5
Blindness	<0.5	0.1	2	<0.5	<0.05	1	<0.5	<0.05	<0.5
**Eye disease (OED)**	**1**	**0.1**	**2**	**1**	**<0.05**	**1**	**<0.5**	**<0.05**	**<0.5**
**Total all manifestations**	**181**	**27.3**	**13**	**50**	**3.4**	**3**	**17**	**0.9**	**1**

Note 1: Population at risk, number of cases (N) and DALYs are presented in thousands.

Note 2: Total skin disease is defined as the sum of the number of cases with palpable nodules, reversible and irreversible skin disease.

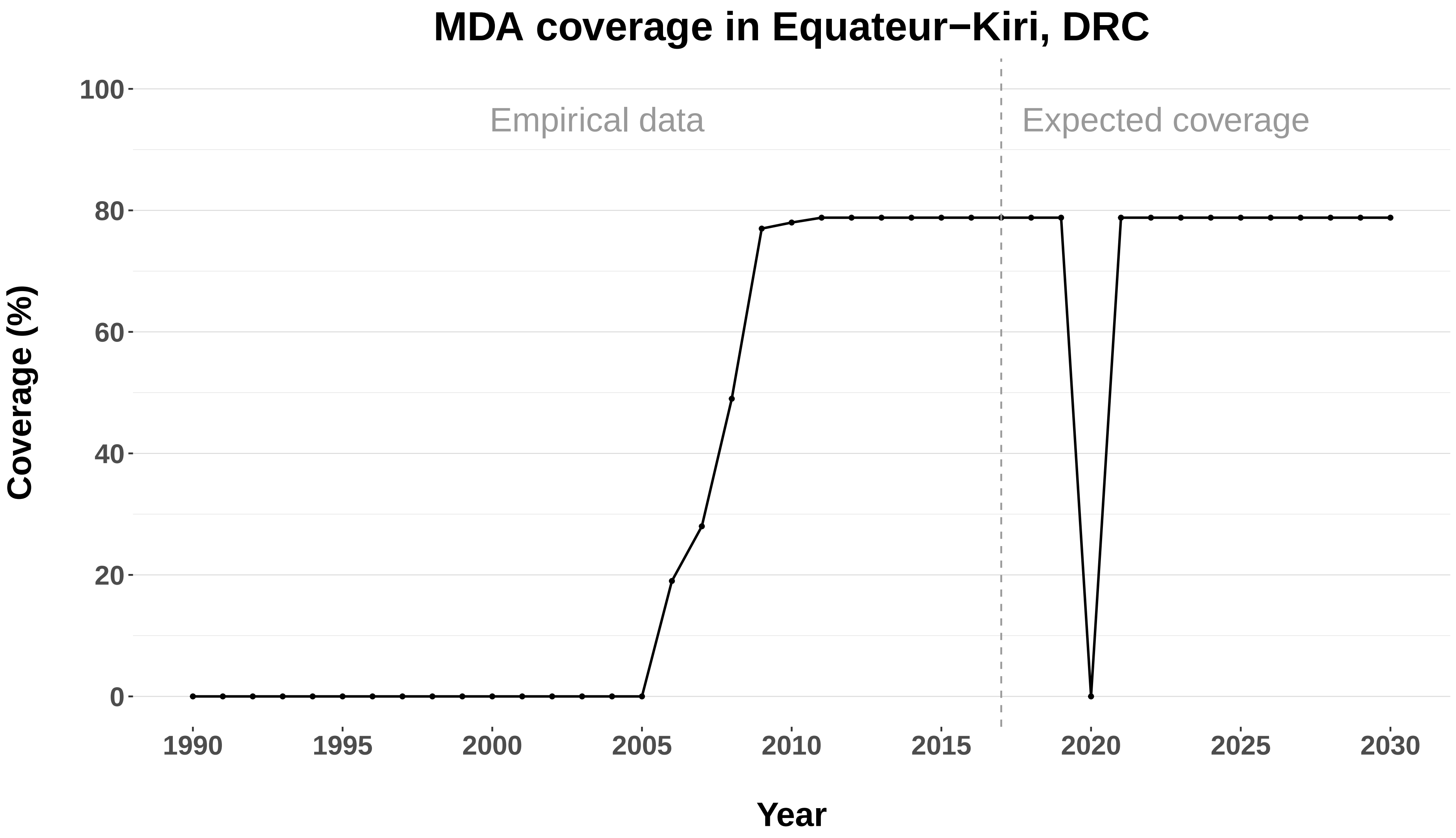

MDA coverage in Equateur−Kiri, DRC
Empirical data
Expected coverage
Coverage (%)
100
80
60
40
20
0
1990
1995
2000
2005
2010
2015
2020
2025
2030
Year

**Project, country:**	**Ituri-Nord, DRC**
Bioclime:	
Endemicity level(s):	Hyperendemic
MDA start year:	2008
Mean MDA coverage per year:	75.3

	1990			2020			2030		
	N	%	DALYs	N	%	DALYs	N	%	DALYs
Total populaton at risk	671	100	_	1,515	100	_	1,987	100	_
**Infection:**									
Mf-positive	423	63.0	_	306	20.2	_	57	2.9	_
Mf-positive (age 5+)	421	62.7	_	306	20.2	_	57	2.9	_
Worm infected	469	69.9	_	648	42.8	_	257	12.9	_
**Clinical manifestations:**									
Palpable nodules	246	36.7	3	172	11.4	2	20	1.0	<0.5
Reversible skin disease:									
Severe itch	108	16.1	20	37	2.4	7	2	0.1	<0.5
Reactive skin disease	96	14.3	4	3	0.2	<0.5	<0.5	<0.05	<0.5
**Reversible skin disease**	**205**	**30.5**	**24**	**39**	**2.6**	**7**	**2**	**0.1**	**<0.5**
Irreversible skin disease:									
Mild depigmentation	20	3.0	<0.5	28	1.8	<0.5	22	1.1	<0.5
Severe depigmentation	35	5.3	2	46	3.0	3	33	1.7	2
Atrophy	1	0.2	<0.5	<0.5	<0.05	<0.5	<0.5	<0.05	<0.5
Hanging groin	3	0.4	1	3	0.2	1	2	0.1	1
**Irreversible skin disease**	**59**	**8.8**	**4**	**77**	**5.1**	**4**	**57**	**2.9**	**3**
**Total skin disease (OSD)**	**510**	**76.0**	**30**	**289**	**19.1**	**13**	**79**	**4.0**	**4**
Eye disease (OED):									
Visual impairment	3	0.5	<0.5	4	0.3	<0.5	3	0.1	<0.5
Blindness	5	0.8	38	4	0.3	28	2	0.1	11
**Eye disease (OED)**	**9**	**1.3**	**38**	**8**	**0.5**	**28**	**4**	**0.2**	**11**
**Total all manifestations**	**519**	**77.3**	**68**	**297**	**19.6**	**41**	**83**	**4.2**	**15**

Note 1: Population at risk, number of cases (N) and DALYs are presented in thousands.

Note 2: Total skin disease is defined as the sum of the number of cases with palpable nodules, reversible and irreversible skin disease.

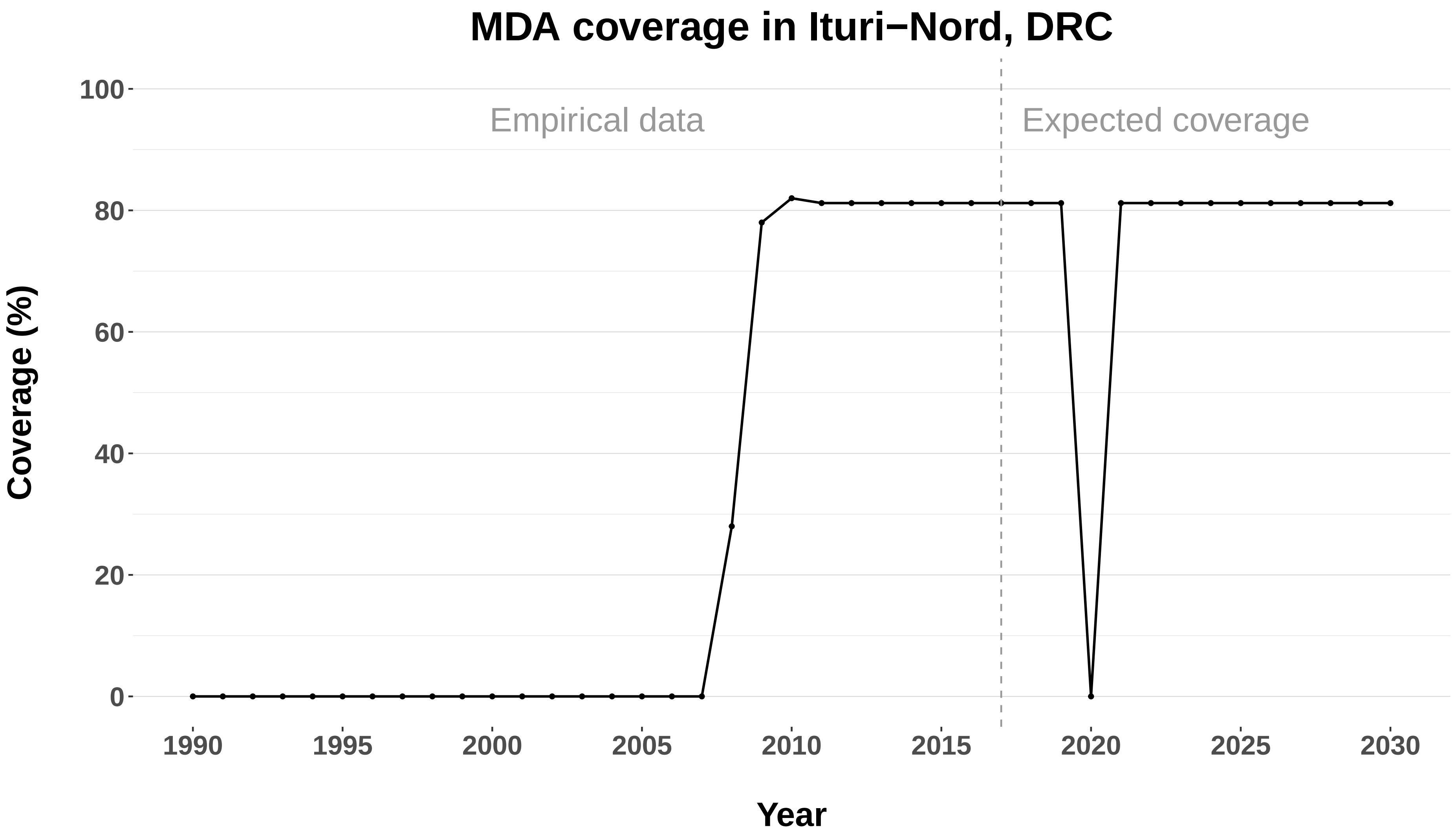

MDA coverage in Ituri−Nord, DRC
Empirical data
Expected coverage
Coverage (%)
100
80
60
40
20
0
1990
1995
2000
2005
2010
2015
2020
2025
2030
Year

**Project, country:**	**Ituri-Sud, DRC**
Bioclime:	
Endemicity level(s):	Hyperendemic
MDA start year:	2012
Mean MDA coverage per year:	68.1

	1990			2020			2030		
	N	%	DALYs	N	%	DALYs	N	%	DALYs
Total populaton at risk	614	100	_	1,385	100	_	1,816	100	_
**Infection:**									
Mf-positive	354	57.7	_	462	33.4	_	177	9.7	_
Mf-positive (age 5+)	352	57.4	_	462	33.4	_	177	9.7	_
Worm infected	399	65.0	_	709	51.2	_	443	24.4	_
**Clinical manifestations:**									
Palpable nodules	192	31.2	2	255	18.4	3	66	3.6	1
Reversible skin disease:									
Severe itch	85	13.9	15	63	4.6	12	10	0.5	2
Reactive skin disease	74	12.0	3	15	1.1	1	2	0.1	<0.5
**Reversible skin disease**	**159**	**25.9**	**19**	**78**	**5.7**	**12**	**11**	**0.6**	**2**
Irreversible skin disease:									
Mild depigmentation	16	2.6	<0.5	26	1.9	<0.5	21	1.2	<0.5
Severe depigmentation	27	4.4	2	42	3.1	3	32	1.8	2
Atrophy	1	0.1	<0.5	<0.5	<0.05	<0.5	<0.5	<0.05	<0.5
Hanging groin	2	0.3	1	2	0.2	1	1	0.1	1
**Irreversible skin disease**	**46**	**7.4**	**3**	**71**	**5.2**	**4**	**55**	**3.0**	**3**
**Total skin disease (OSD)**	**396**	**64.5**	**24**	**405**	**29.2**	**19**	**132**	**7.3**	**5**
Eye disease (OED):									
Visual impairment	2	0.4	<0.5	4	0.3	<0.5	2	0.1	<0.5
Blindness	3	0.5	24	4	0.3	26	2	0.1	12
**Eye disease (OED)**	**6**	**0.9**	**24**	**7**	**0.5**	**26**	**4**	**0.2**	**12**
**Total all manifestations**	**402**	**65.5**	**48**	**412**	**29.7**	**45**	**136**	**7.5**	**17**

Note 1: Population at risk, number of cases (N) and DALYs are presented in thousands.

Note 2: Total skin disease is defined as the sum of the number of cases with palpable nodules, reversible and irreversible skin disease.

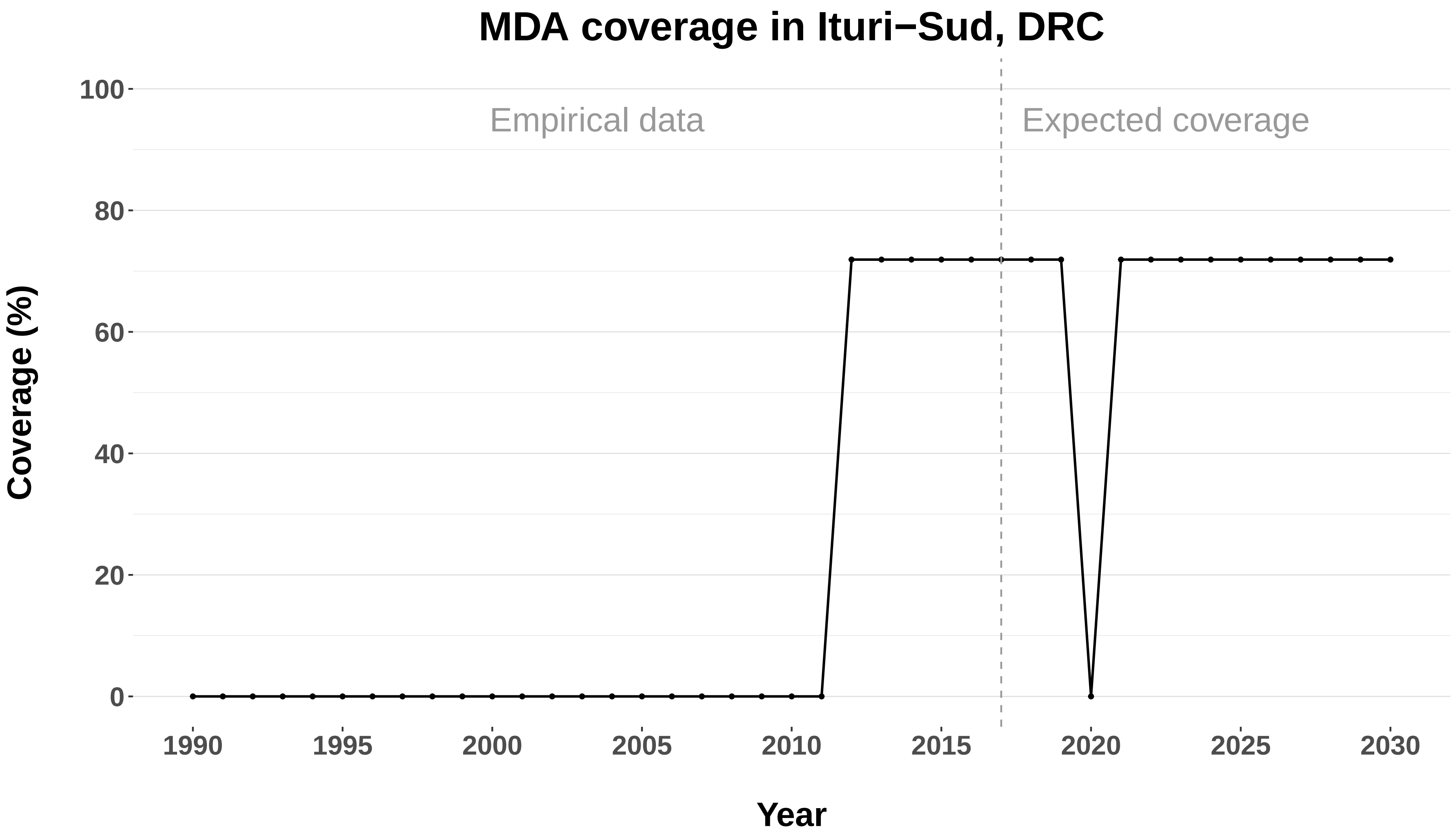
MDA coverage in Ituri−Sud, DRC
Empirical data
Expected coverage
Coverage (%)
100
80
60
40
20
0
1990
1995
2000
2005
2010
2015
2020
2025
2030
Year

**Project, country:**	**Kasai, DRC**
Bioclime:	
Endemicity level(s):	Hyperendemic
MDA start year:	2001
Mean MDA coverage per year:	61.9

	1990			2020			2030		
	N	%	DALYs	N	%	DALYs	N	%	DALYs
Total populaton at risk	5,756	100	_	12,990	100	_	17,038	100	_
**Infection:**									
Mf-positive	2,210	38.4	_	608	4.7	_	138	0.8	_
Mf-positive (age 5+)	2,205	38.3	_	607	4.7	_	138	0.8	_
Worm infected	2,690	46.7	_	1,912	14.7	_	542	3.2	_
**Clinical manifestations:**									
Palpable nodules	801	13.9	9	260	2.0	3	46	0.3	<0.5
Reversible skin disease:									
Severe itch	403	7.0	73	49	0.4	9	4	<0.05	1
Reactive skin disease	319	5.5	15	5	<0.05	<0.5	1	<0.05	<0.5
**Reversible skin disease**	**722**	**12.5**	**88**	**55**	**0.4**	**9**	**4**	**<0.05**	**1**
Irreversible skin disease:									
Mild depigmentation	86	1.5	1	94	0.7	1	67	0.4	1
Severe depigmentation	113	2.0	7	114	0.9	7	78	0.5	5
Atrophy	2	<0.05	<0.5	<0.5	<0.05	<0.5	<0.5	<0.05	<0.5
Hanging groin	5	0.1	2	4	<0.05	2	2	<0.05	1
**Irreversible skin disease**	**207**	**3.6**	**10**	**212**	**1.6**	**10**	**146**	**0.9**	**7**
**Total skin disease (OSD)**	**1,730**	**30.1**	**107**	**526**	**4.1**	**22**	**196**	**1.2**	**8**
Eye disease (OED):									
Visual impairment	7	0.1	<0.5	6	<0.05	<0.5	4	<0.05	<0.5
Blindness	10	0.2	72	5	<0.05	38	2	<0.05	16
**Eye disease (OED)**	**17**	**0.3**	**72**	**12**	**0.1**	**38**	**6**	**<0.05**	**16**
**Total all manifestations**	**1,748**	**30.4**	**179**	**538**	**4.1**	**60**	**202**	**1.2**	**23**

Note 1: Population at risk, number of cases (N) and DALYs are presented in thousands.

Note 2: Total skin disease is defined as the sum of the number of cases with palpable nodules, reversible and irreversible skin disease.

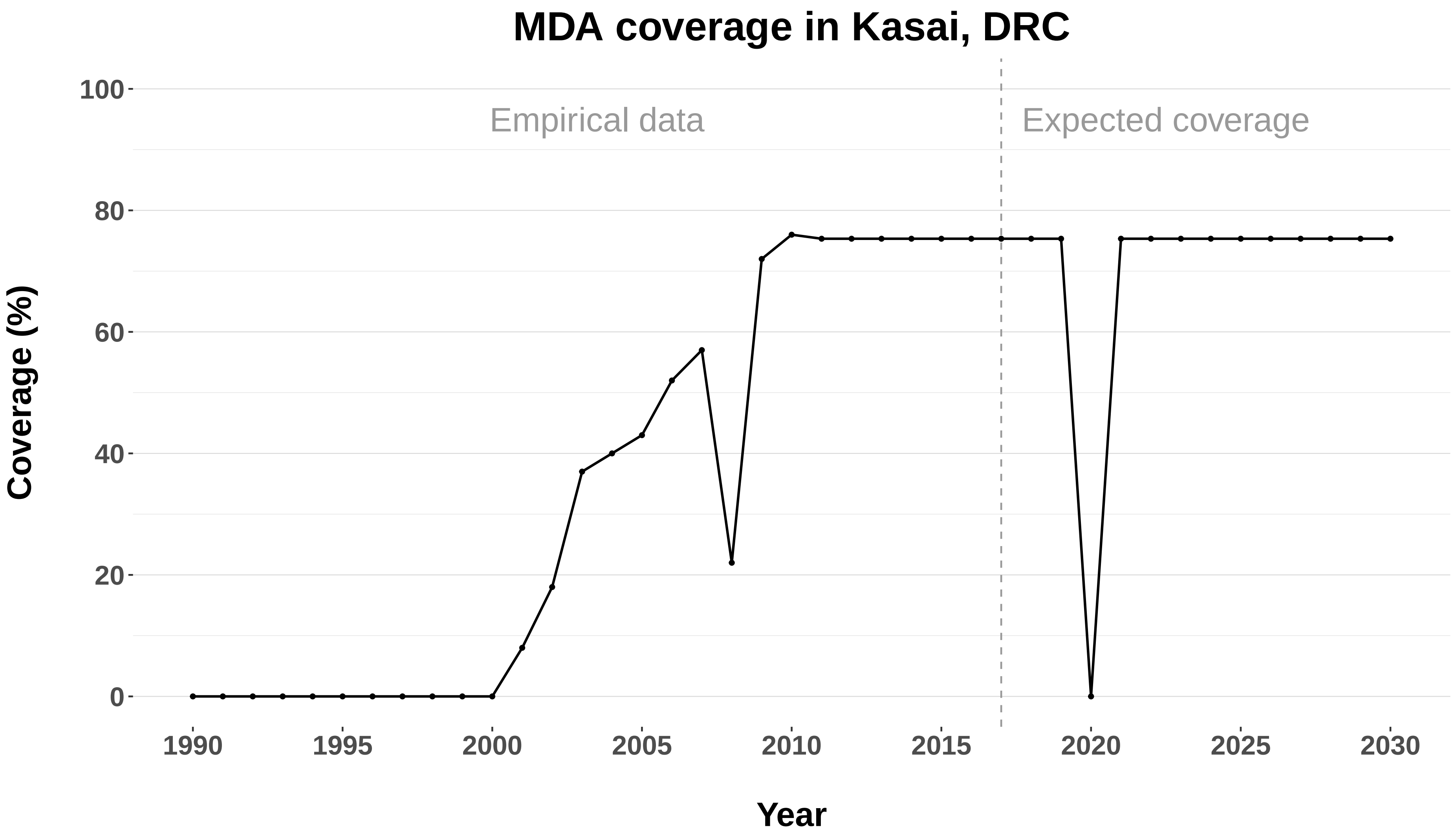

MDA coverage in Kasai, DRC
Empirical data
Expected coverage
Coverage (%)
100
80
60
40
20
0
1990
1995
2000
2005
2010
2015
2020
2025
2030
Year

**Project, country:**	**Kasongo, DRC**
Bioclime:	
Endemicity level(s):	Hyperendemic
MDA start year:	2007
Mean MDA coverage per year:	63.6

	1990			2020			2030		
	N	%	DALYs	N	%	DALYs	N	%	DALYs
Total populaton at risk	721	100	_	1,627	100	_	2,134	100	_
**Infection:**									
Mf-positive	228	31.6	_	81	5.0	_	8	0.4	_
Mf-positive (age 5+)	227	31.5	_	81	5.0	_	8	0.4	_
Worm infected	289	40.1	_	270	16.6	_	50	2.3	_
**Clinical manifestations:**									
Palpable nodules	59	8.2	1	25	1.6	<0.5	3	0.1	<0.5
Reversible skin disease:									
Severe itch	34	4.7	6	7	0.4	1	<0.5	<0.05	<0.5
Reactive skin disease	25	3.4	1	1	0.1	<0.5	<0.5	<0.05	<0.5
**Reversible skin disease**	**59**	**8.1**	**7**	**8**	**0.5**	**1**	**<0.5**	**<0.05**	**<0.5**
Irreversible skin disease:									
Mild depigmentation	8	1.1	<0.5	10	0.6	<0.5	8	0.4	<0.5
Severe depigmentation	8	1.2	1	10	0.6	1	7	0.3	<0.5
Atrophy	<0.5	<0.05	<0.5	<0.5	<0.05	<0.5	<0.5	<0.05	<0.5
Hanging groin	<0.5	<0.05	<0.5	<0.5	<0.05	<0.5	<0.5	<0.05	<0.5
**Irreversible skin disease**	**17**	**2.3**	**1**	**21**	**1.3**	**1**	**15**	**0.7**	**1**
**Total skin disease (OSD)**	**134**	**18.6**	**9**	**54**	**3.3**	**2**	**18**	**0.8**	**1**
Eye disease (OED):									
Visual impairment	<0.5	<0.05	<0.5	<0.5	<0.05	<0.5	<0.5	<0.05	<0.5
Blindness	<0.5	0.1	2	<0.5	<0.05	2	<0.5	<0.05	1
**Eye disease (OED)**	**1**	**0.1**	**2**	**1**	**<0.05**	**2**	**<0.5**	**<0.05**	**1**
**Total all manifestations**	**135**	**18.7**	**11**	**55**	**3.4**	**4**	**18**	**0.9**	**1**

Note 1: Population at risk, number of cases (N) and DALYs are presented in thousands.

Note 2: Total skin disease is defined as the sum of the number of cases with palpable nodules, reversible and irreversible skin disease.

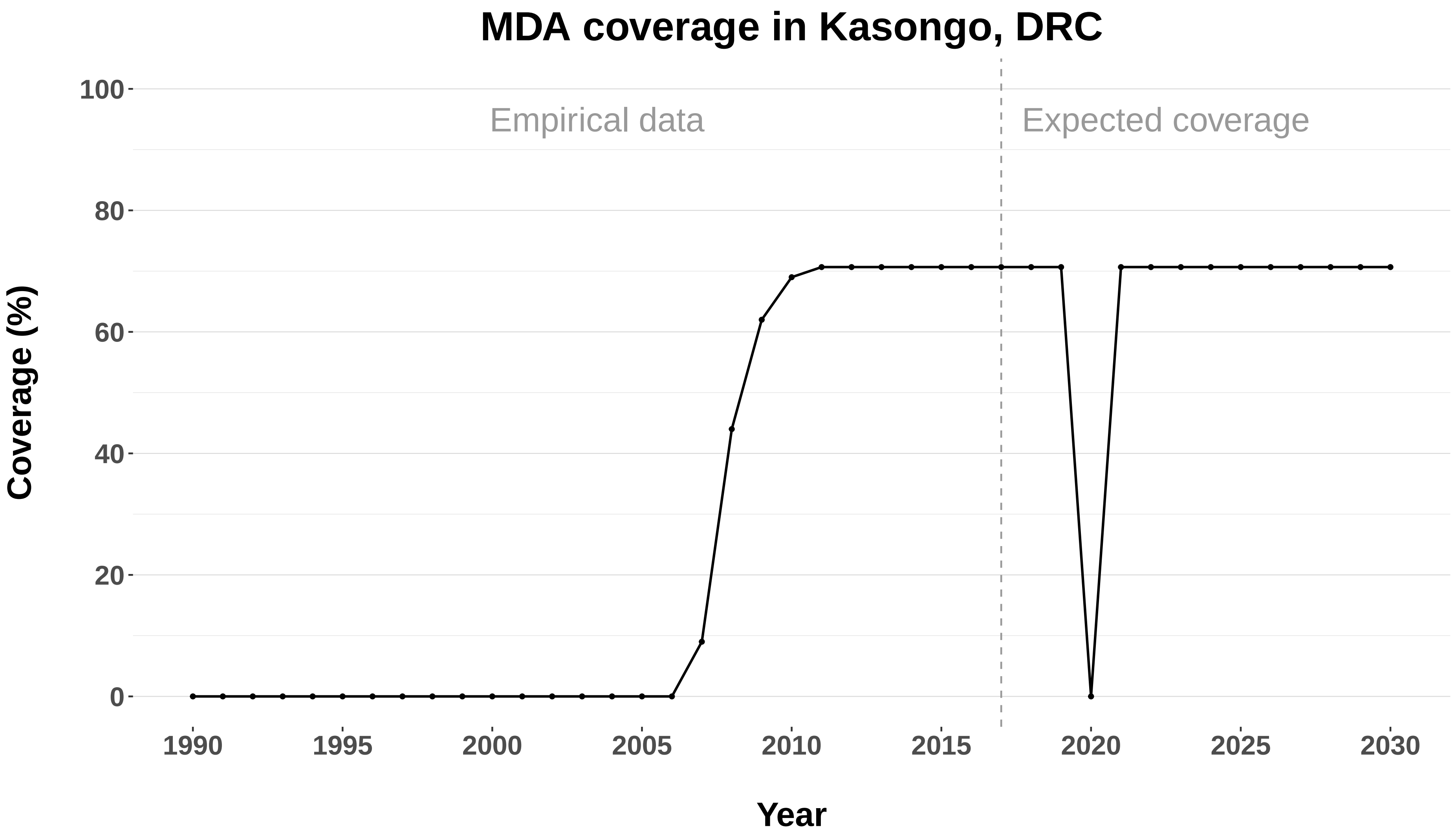

MDA coverage in Kasongo, DRC
Empirical data
Expected coverage
Coverage (%)
100
80
60
40
20
0
1990
1995
2000
2005
2010
2015
2020
2025
2030
Year

**Project, country:**	**Katanga-Nord, DRC**
Bioclime:	
Endemicity level(s):	Hyperendemic
MDA start year:	2006
Mean MDA coverage per year:	71.7

	1990			2020			2030		
	N	%	DALYs	N	%	DALYs	N	%	DALYs
Total populaton at risk	335	100	_	756	100	_	992	100	_
**Infection:**									
Mf-positive	126	37.6	_	37	4.9	_	3	0.3	_
Mf-positive (age 5+)	126	37.5	_	37	4.9	_	3	0.3	_
Worm infected	153	45.8	_	128	17.0	_	24	2.4	_
**Clinical manifestations:**									
Palpable nodules	45	13.4	<0.5	17	2.2	<0.5	1	0.1	<0.5
Reversible skin disease:									
Severe itch	23	6.7	4	4	0.5	1	<0.5	<0.05	<0.5
Reactive skin disease	18	5.4	1	<0.5	<0.05	<0.5	<0.5	<0.05	<0.5
**Reversible skin disease**	**41**	**12.1**	**5**	**4**	**0.5**	**1**	**<0.5**	**<0.05**	**<0.5**
Irreversible skin disease:									
Mild depigmentation	5	1.4	<0.5	6	0.8	<0.5	4	0.4	<0.5
Severe depigmentation	6	1.9	<0.5	7	1.0	<0.5	5	0.5	<0.5
Atrophy	<0.5	<0.05	<0.5	<0.5	<0.05	<0.5	<0.5	<0.05	<0.5
Hanging groin	<0.5	0.1	<0.5	<0.5	<0.05	<0.5	<0.5	<0.05	<0.5
**Irreversible skin disease**	**11**	**3.4**	**1**	**13**	**1.8**	**1**	**9**	**0.9**	**<0.5**
**Total skin disease (OSD)**	**97**	**28.9**	**6**	**34**	**4.5**	**2**	**11**	**1.1**	**<0.5**
Eye disease (OED):									
Visual impairment	<0.5	0.1	<0.5	<0.5	0.1	<0.5	<0.5	<0.05	<0.5
Blindness	<0.5	0.1	3	<0.5	<0.05	2	<0.5	<0.05	1
**Eye disease (OED)**	**1**	**0.3**	**3**	**1**	**0.1**	**2**	**<0.5**	**<0.05**	**1**
**Total all manifestations**	**98**	**29.1**	**9**	**35**	**4.6**	**4**	**11**	**1.1**	**1**

Note 1: Population at risk, number of cases (N) and DALYs are presented in thousands.

Note 2: Total skin disease is defined as the sum of the number of cases with palpable nodules, reversible and irreversible skin disease.

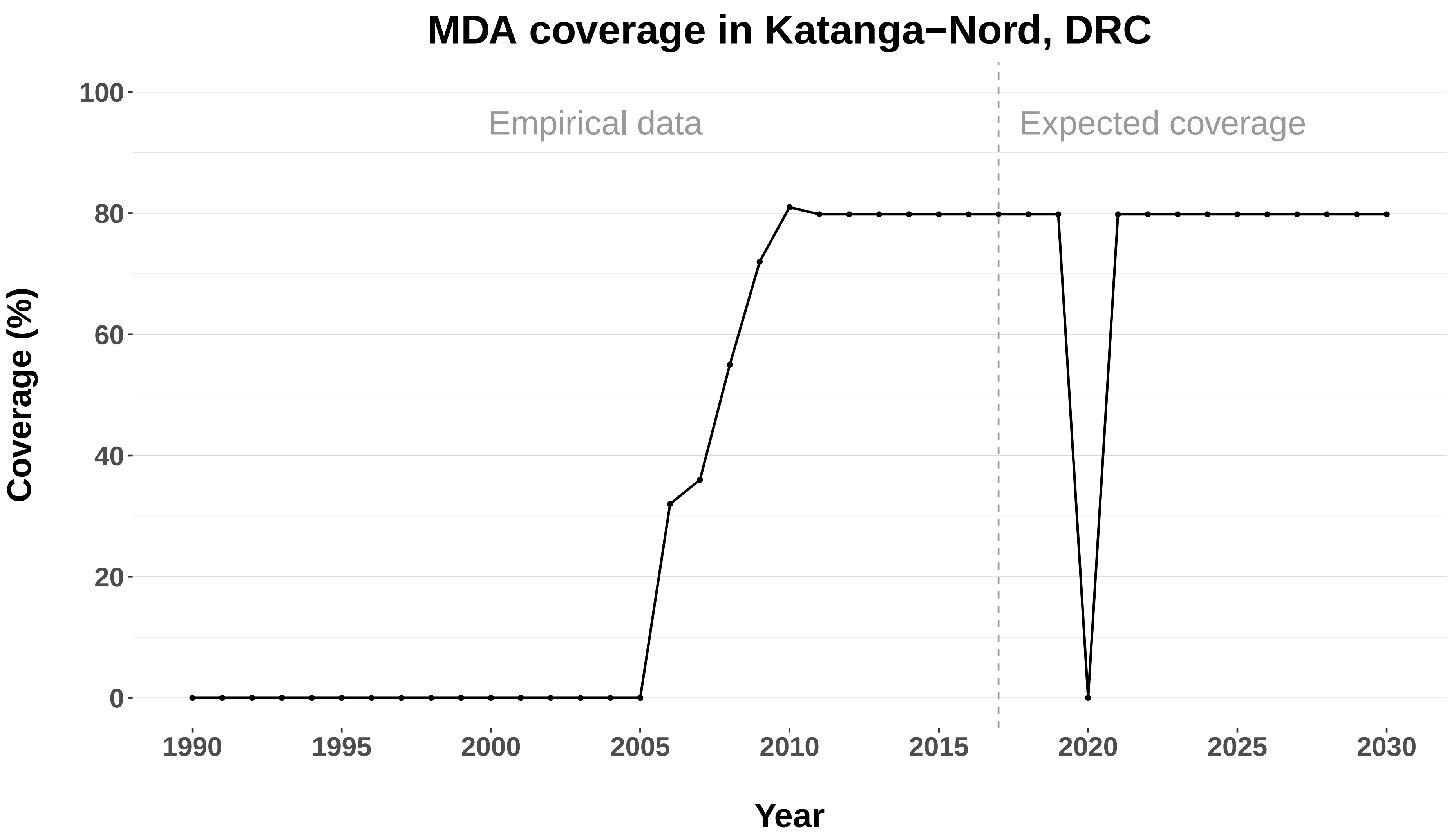
MDA coverage in Katanga−Nord, DRC
Empirical data
Expected coverage
Coverage (%)
100
80
60
40
20
0
1990
1995
2000
2005
2010
2015
2020
2025
2030
Year

**Project, country:**	**Katanga-Sud, DRC**
Bioclime:	
Endemicity level(s):	Hyperendemic
MDA start year:	2006
Mean MDA coverage per year:	68.1

	1990			2020			2030		
	N	%	DALYs	N	%	DALYs	N	%	DALYs
Total populaton at risk	371	100	_	837	100	_	1,098	100	_
**Infection:**									
Mf-positive	141	38.0	_	52	6.2	_	9	0.8	_
Mf-positive (age 5+)	141	37.9	_	52	6.2	_	9	0.8	_
Worm infected	172	46.3	_	158	18.8	_	39	3.6	_
**Clinical manifestations:**									
Palpable nodules	50	13.6	1	23	2.7	<0.5	3	0.3	<0.5
Reversible skin disease:									
Severe itch	25	6.8	5	5	0.6	1	<0.5	<0.05	<0.5
Reactive skin disease	20	5.4	1	1	0.1	<0.5	<0.5	<0.05	<0.5
**Reversible skin disease**	**45**	**12.2**	**6**	**6**	**0.7**	**1**	**<0.5**	**<0.05**	**<0.5**
Irreversible skin disease:									
Mild depigmentation	5	1.5	<0.5	7	0.8	<0.5	5	0.5	<0.5
Severe depigmentation	7	1.9	<0.5	9	1.0	1	6	0.5	<0.5
Atrophy	<0.5	<0.05	<0.5	<0.5	<0.05	<0.5	<0.5	<0.05	<0.5
Hanging groin	<0.5	0.1	<0.5	<0.5	<0.05	<0.5	<0.5	<0.05	<0.5
**Irreversible skin disease**	**13**	**3.5**	**1**	**16**	**1.9**	**1**	**11**	**1.0**	**<0.5**
**Total skin disease (OSD)**	**109**	**29.3**	**7**	**44**	**5.2**	**2**	**14**	**1.3**	**1**
Eye disease (OED):									
Visual impairment	<0.5	0.1	<0.5	<0.5	0.1	<0.5	<0.5	<0.05	<0.5
Blindness	1	0.2	4	<0.5	<0.05	3	<0.5	<0.05	1
**Eye disease (OED)**	**1**	**0.3**	**4**	**1**	**0.1**	**3**	**<0.5**	**<0.05**	**1**
**Total all manifestations**	**110**	**29.6**	**11**	**45**	**5.3**	**5**	**15**	**1.3**	**2**

Note 1: Population at risk, number of cases (N) and DALYs are presented in thousands.

Note 2: Total skin disease is defined as the sum of the number of cases with palpable nodules, reversible and irreversible skin disease.

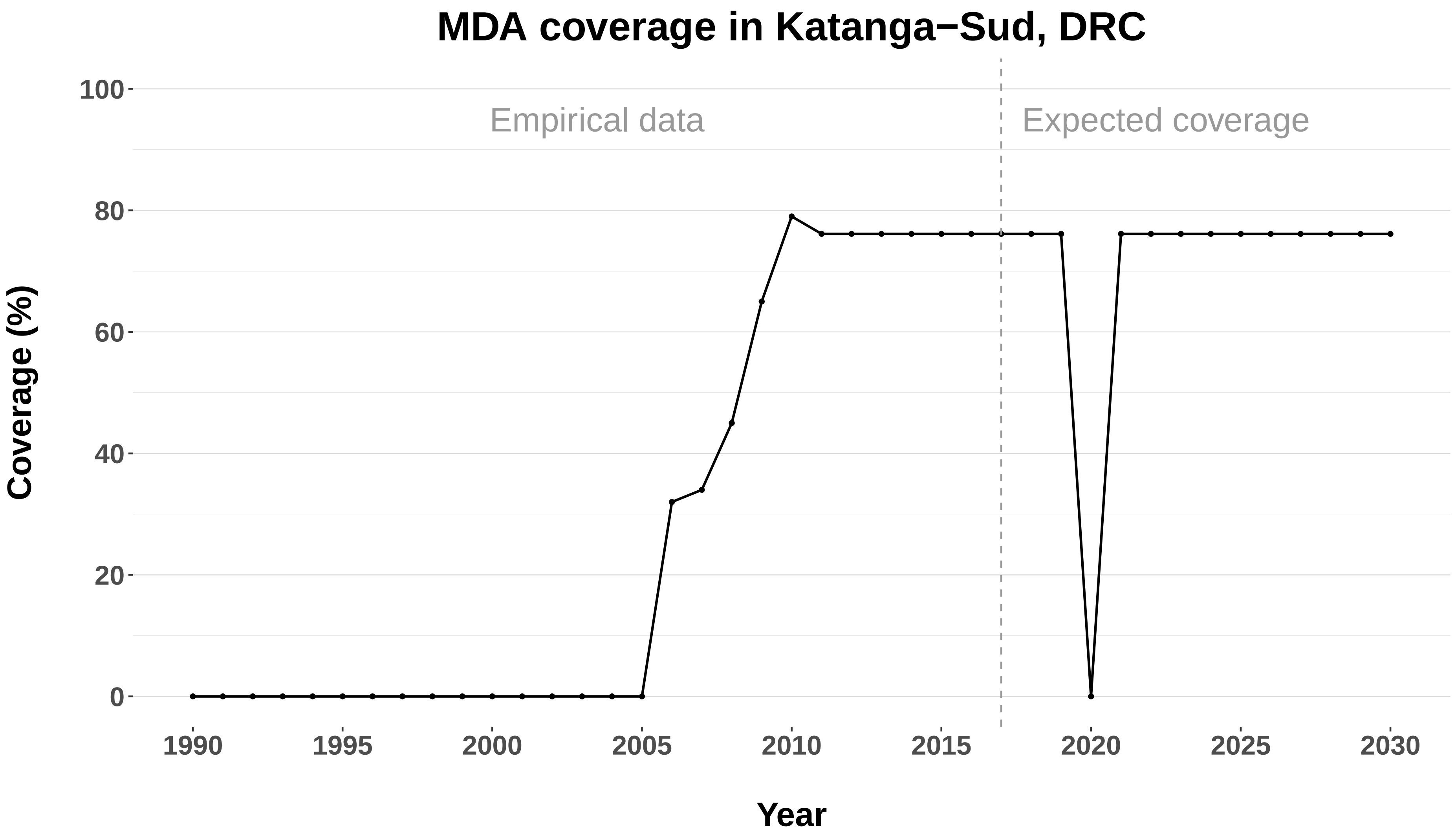

MDA coverage in Katanga−Sud, DRC
Empirical data
Expected coverage
Coverage (%)
100
80
60
40
20
0
1990
1995
2000
2005
2010
2015
2020
2025
2030
Year

**Project, country:**	**Lualaba, DRC**
Bioclime:	
Endemicity level(s):	Hyperendemic
MDA start year:	2006
Mean MDA coverage per year:	74.3

	1990			2020			2030		
	N	%	DALYs	N	%	DALYs	N	%	DALYs
Total populaton at risk	120	100	_	272	100	_	356	100	_
**Infection:**									
Mf-positive	48	39.7	_	7	2.5	_	<0.5	<0.05	_
Mf-positive (age 5+)	48	39.7	_	7	2.5	_	<0.5	<0.05	_
Worm infected	59	49.1	_	37	13.6	_	3	0.8	_
**Clinical manifestations:**									
Palpable nodules	16	13.2	<0.5	3	1.0	<0.5	<0.5	<0.05	<0.5
Reversible skin disease:									
Severe itch	8	6.4	1	1	0.3	<0.5	<0.5	<0.05	<0.5
Reactive skin disease	6	4.7	<0.5	<0.5	<0.05	<0.5	<0.5	<0.05	<0.5
**Reversible skin disease**	**13**	**11.1**	**2**	**1**	**0.3**	**<0.5**	**<0.5**	**<0.05**	**<0.5**
Irreversible skin disease:									
Mild depigmentation	2	1.4	<0.5	2	0.7	<0.5	1	0.4	<0.5
Severe depigmentation	2	1.6	<0.5	2	0.8	<0.5	1	0.4	<0.5
Atrophy	<0.5	<0.05	<0.5	<0.5	<0.05	<0.5	<0.5	<0.05	<0.5
Hanging groin	<0.5	<0.05	<0.5	<0.5	<0.05	<0.5	<0.5	<0.05	<0.5
**Irreversible skin disease**	**4**	**3.0**	**<0.5**	**4**	**1.5**	**<0.5**	**3**	**0.8**	**<0.5**
**Total skin disease (OSD)**	**33**	**27.3**	**2**	**8**	**2.8**	**<0.5**	**3**	**0.8**	**<0.5**
Eye disease (OED):									
Visual impairment	<0.5	0.1	<0.5	<0.5	<0.05	<0.5	<0.5	<0.05	<0.5
Blindness	<0.5	0.1	1	<0.5	<0.05	<0.5	<0.5	<0.05	<0.5
**Eye disease (OED)**	**<0.5**	**0.1**	**1**	**<0.5**	**<0.05**	**<0.5**	**<0.5**	**<0.05**	**<0.5**
**Total all manifestations**	**33**	**27.5**	**2**	**8**	**2.8**	**1**	**3**	**0.8**	**<0.5**

Note 1: Population at risk, number of cases (N) and DALYs are presented in thousands.

Note 2: Total skin disease is defined as the sum of the number of cases with palpable nodules, reversible and irreversible skin disease.

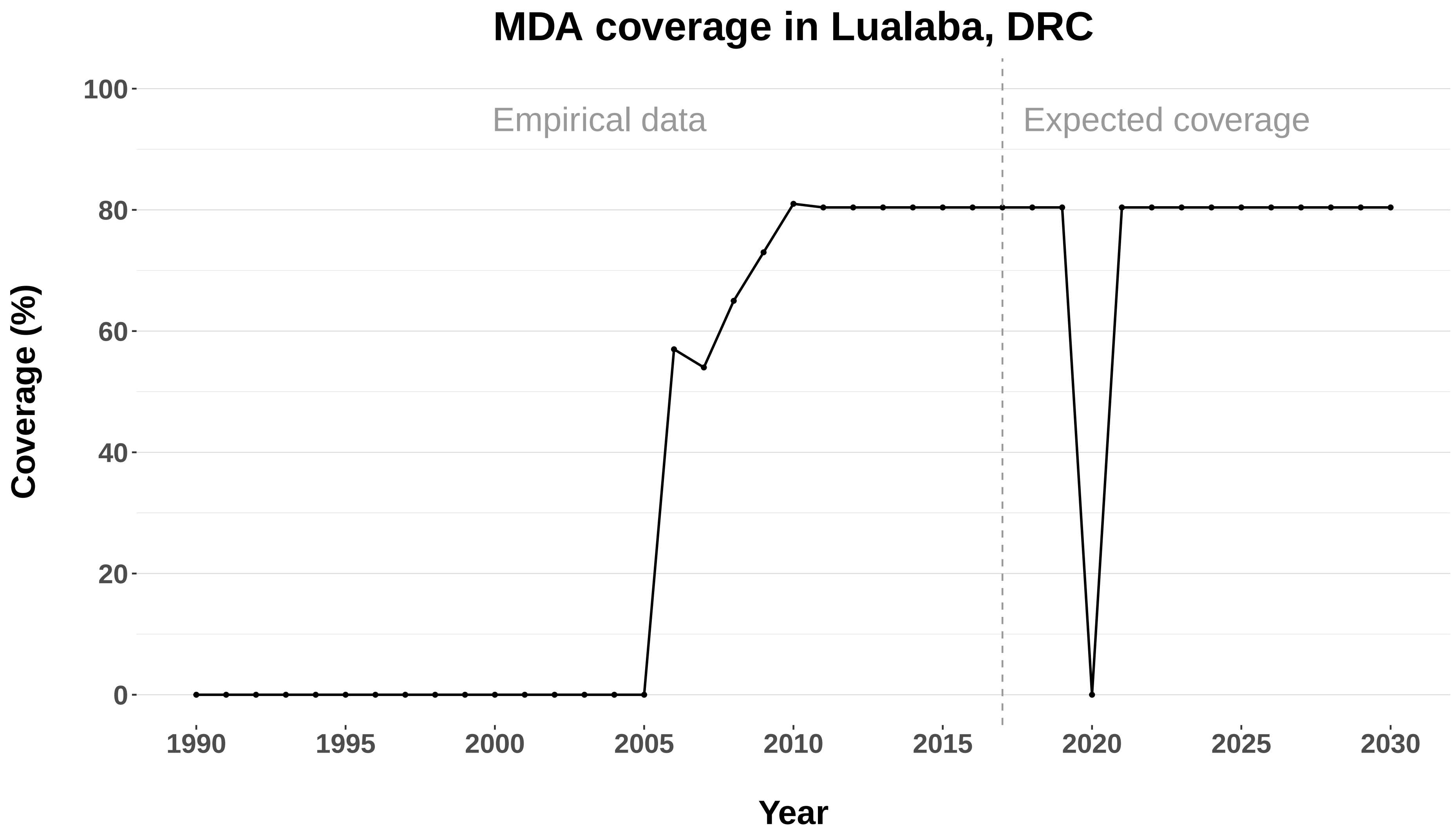
MDA coverage in Lualaba, DRC
Empirical data
Expected coverage
Coverage (%)
100
80
60
40
20
0
1990
1995
2000
2005
2010
2015
2020
2025
2030
Year

**Project, country:**	**Lubutu, DRC**
Bioclime:	
Endemicity level(s):	Hyperendemic
MDA start year:	2008
Mean MDA coverage per year:	66.7

	1990			2020			2030		
	N	%	DALYs	N	%	DALYs	N	%	DALYs
Total populaton at risk	179	100	_	403	100	_	529	100	_
**Infection:**									
Mf-positive	101	56.7	_	60	14.8	_	5	0.9	_
Mf-positive (age 5+)	101	56.6	_	60	14.8	_	5	0.9	_
Worm infected	118	66.0	_	149	36.8	_	36	6.8	_
**Clinical manifestations:**									
Palpable nodules	45	25.2	<0.5	24	6.1	<0.5	2	0.3	<0.5
Reversible skin disease:									
Severe itch	19	10.8	4	6	1.4	1	<0.5	<0.05	<0.5
Reactive skin disease	15	8.4	1	1	0.2	<0.5	<0.5	<0.05	<0.5
**Reversible skin disease**	**34**	**19.2**	**4**	**6**	**1.6**	**1**	**<0.5**	**0.1**	**<0.5**
Irreversible skin disease:									
Mild depigmentation	4	2.2	<0.5	6	1.4	<0.5	4	0.8	<0.5
Severe depigmentation	5	2.8	<0.5	7	1.6	<0.5	5	0.9	<0.5
Atrophy	<0.5	<0.05	<0.5	<0.5	<0.05	<0.5	<0.5	<0.05	<0.5
Hanging groin	<0.5	0.1	<0.5	<0.5	<0.05	<0.5	<0.5	<0.05	<0.5
**Irreversible skin disease**	**9**	**5.2**	**<0.5**	**12**	**3.1**	**1**	**9**	**1.7**	**<0.5**
**Total skin disease (OSD)**	**89**	**49.6**	**5**	**43**	**10.7**	**2**	**11**	**2.0**	**<0.5**
Eye disease (OED):									
Visual impairment	<0.5	0.2	<0.5	<0.5	0.1	<0.5	<0.5	<0.05	<0.5
Blindness	<0.5	0.2	2	<0.5	0.1	1	<0.5	<0.05	<0.5
**Eye disease (OED)**	**1**	**0.3**	**2**	**1**	**0.1**	**1**	**<0.5**	**<0.05**	**<0.5**
**Total all manifestations**	**89**	**49.9**	**7**	**44**	**10.8**	**3**	**11**	**2.1**	**1**

Note 1: Population at risk, number of cases (N) and DALYs are presented in thousands.

Note 2: Total skin disease is defined as the sum of the number of cases with palpable nodules, reversible and irreversible skin disease.

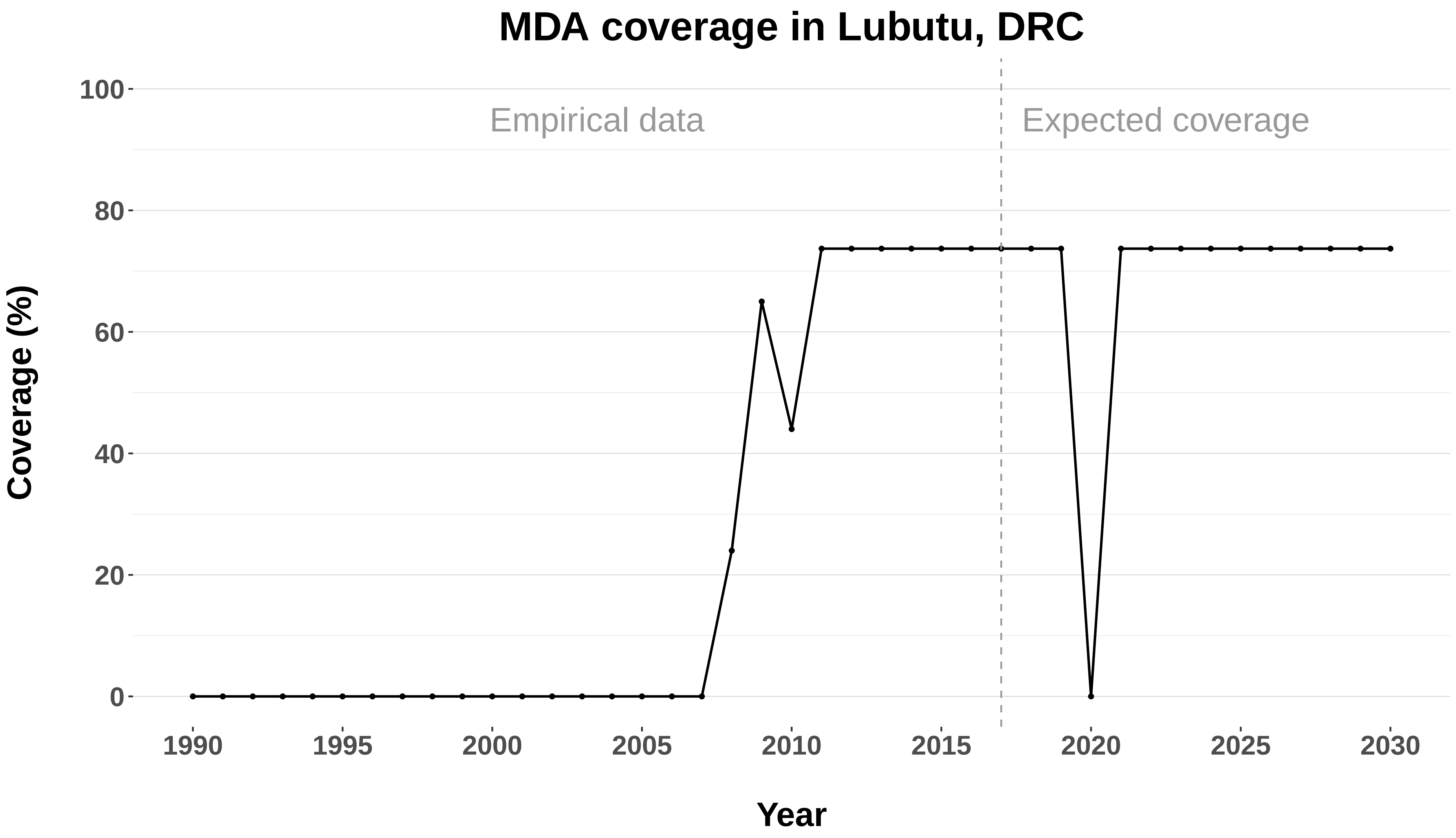
MDA coverage in Lubutu, DRC
Empirical data
Expected coverage
Coverage (%)
100
80
60
40
20
0
1990
1995
2000
2005
2010
2015
2020
2025
2030
Year

**Project, country:**	**Masisi-Walikale, DRC**
Bioclime:	
Endemicity level(s):	Hyperendemic
MDA start year:	2009
Mean MDA coverage per year:	67.7

	1990			2020			2030		
	N	%	DALYs	N	%	DALYs	N	%	DALYs
Total populaton at risk	560	100	_	1,265	100	_	1,659	100	_
**Infection:**									
Mf-positive	290	51.7	_	200	15.8	_	40	2.4	_
Mf-positive (age 5+)	289	51.5	_	200	15.8	_	40	2.4	_
Worm infected	341	60.8	_	449	35.5	_	149	9.0	_
**Clinical manifestations:**									
Palpable nodules	128	22.8	1	91	7.2	1	14	0.9	<0.5
Reversible skin disease:									
Severe itch	56	10.0	10	21	1.7	4	2	0.1	<0.5
Reactive skin disease	45	8.1	2	3	0.2	<0.5	<0.5	<0.05	<0.5
**Reversible skin disease**	**101**	**18.1**	**12**	**24**	**1.9**	**4**	**2**	**0.1**	**<0.5**
Irreversible skin disease:									
Mild depigmentation	11	2.0	<0.5	16	1.3	<0.5	12	0.7	<0.5
Severe depigmentation	16	2.8	1	21	1.7	1	15	0.9	1
Atrophy	<0.5	0.1	<0.5	<0.5	<0.05	<0.5	<0.5	<0.05	<0.5
Hanging groin	1	0.1	<0.5	1	0.1	<0.5	<0.5	<0.05	<0.5
**Irreversible skin disease**	**28**	**5.0**	**1**	**39**	**3.0**	**2**	**28**	**1.7**	**1**
**Total skin disease (OSD)**	**257**	**45.9**	**15**	**154**	**12.1**	**7**	**44**	**2.7**	**2**
Eye disease (OED):									
Visual impairment	1	0.2	<0.5	1	0.1	<0.5	1	0.1	<0.5
Blindness	1	0.2	10	1	0.1	8	<0.5	<0.05	3
**Eye disease (OED)**	**2**	**0.4**	**10**	**2**	**0.2**	**8**	**1**	**0.1**	**3**
**Total all manifestations**	**260**	**46.3**	**25**	**156**	**12.3**	**15**	**46**	**2.8**	**5**

Note 1: Population at risk, number of cases (N) and DALYs are presented in thousands.

Note 2: Total skin disease is defined as the sum of the number of cases with palpable nodules, reversible and irreversible skin disease.

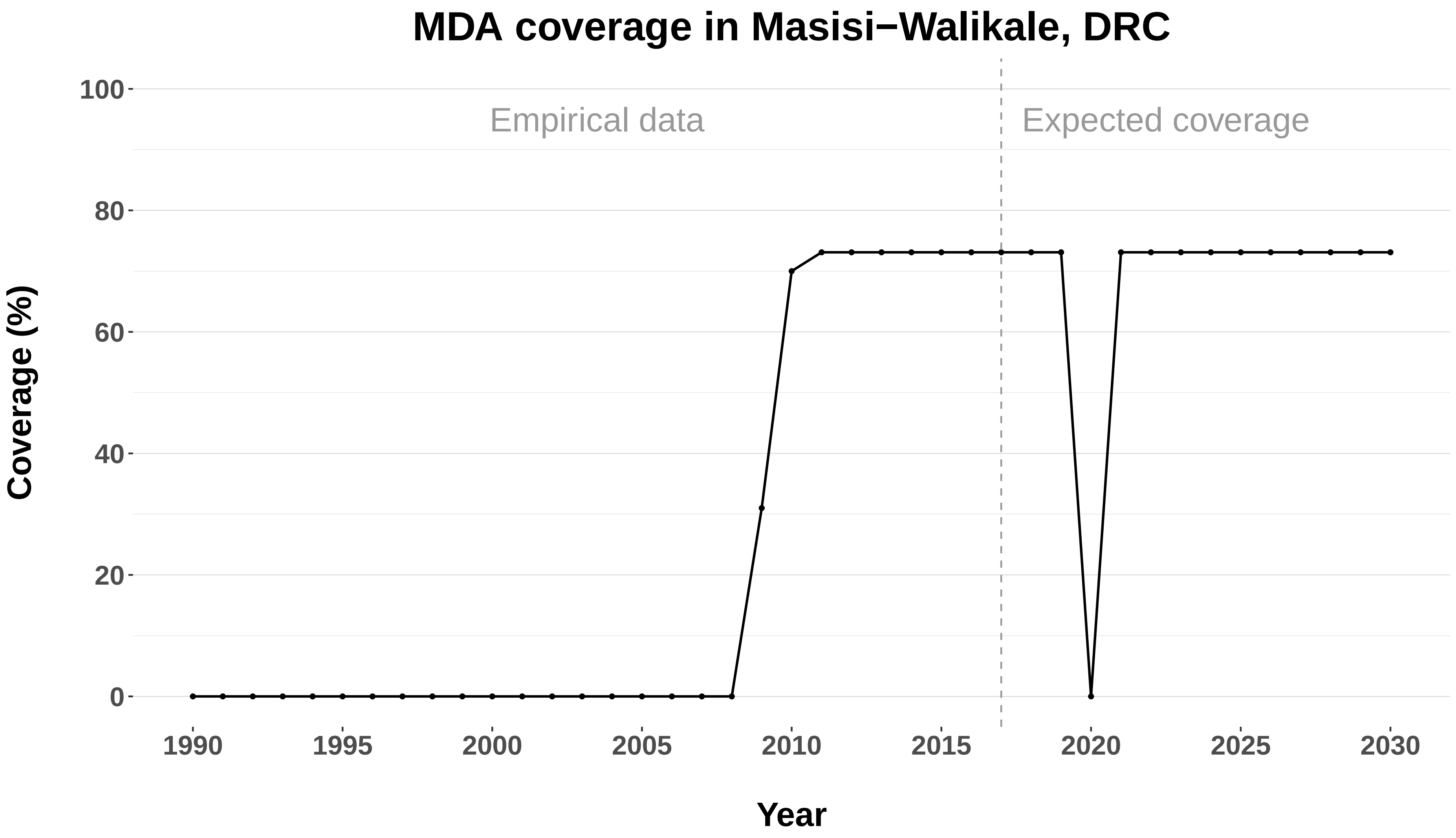
MDA coverage in Masisi−Walikale, DRC
Empirical data
Expected coverage
Coverage (%)
100
80
60
40
20
0
1990
1995
2000
2005
2010
2015
2020
2025
2030
Year

**Project, country:**	**Mongala, DRC**
Bioclime:	
Endemicity level(s):	Hyperendemic
MDA start year:	2006
Mean MDA coverage per year:	68.8

	1990			2020			2030		
	N	%	DALYs	N	%	DALYs	N	%	DALYs
Total populaton at risk	779	100	_	1,758	100	_	2,306	100	_
**Infection:**									
Mf-positive	286	36.7	_	59	3.4	_	2	0.1	_
Mf-positive (age 5+)	286	36.7	_	59	3.4	_	2	0.1	_
Worm infected	363	46.6	_	272	15.5	_	28	1.2	_
**Clinical manifestations:**									
Palpable nodules	86	11.1	1	23	1.3	<0.5	1	<0.05	<0.5
Reversible skin disease:									
Severe itch	43	5.5	8	6	0.3	1	<0.5	<0.05	<0.5
Reactive skin disease	31	3.9	1	<0.5	<0.05	<0.5	<0.5	<0.05	<0.5
**Reversible skin disease**	**74**	**9.4**	**9**	**6**	**0.3**	**1**	**<0.5**	**<0.05**	**<0.5**
Irreversible skin disease:									
Mild depigmentation	10	1.3	<0.5	12	0.7	<0.5	9	0.4	<0.5
Severe depigmentation	10	1.3	1	12	0.7	1	8	0.3	<0.5
Atrophy	<0.5	<0.05	<0.5	<0.5	<0.05	<0.5	<0.5	<0.05	<0.5
Hanging groin	<0.5	<0.05	<0.5	<0.5	<0.05	<0.5	<0.5	<0.05	<0.5
**Irreversible skin disease**	**21**	**2.6**	**1**	**24**	**1.3**	**1**	**16**	**0.7**	**1**
**Total skin disease (OSD)**	**181**	**23.2**	**11**	**53**	**3.0**	**2**	**17**	**0.8**	**1**
Eye disease (OED):									
Visual impairment	<0.5	<0.05	<0.5	<0.5	<0.05	<0.5	<0.5	<0.05	<0.5
Blindness	<0.5	0.1	3	<0.5	<0.05	2	<0.5	<0.05	1
**Eye disease (OED)**	**1**	**0.1**	**3**	**1**	**<0.05**	**2**	**<0.5**	**<0.05**	**1**
**Total all manifestations**	**181**	**23.3**	**14**	**54**	**3.0**	**4**	**18**	**0.8**	**1**

Note 1: Population at risk, number of cases (N) and DALYs are presented in thousands.

Note 2: Total skin disease is defined as the sum of the number of cases with palpable nodules, reversible and irreversible skin disease.

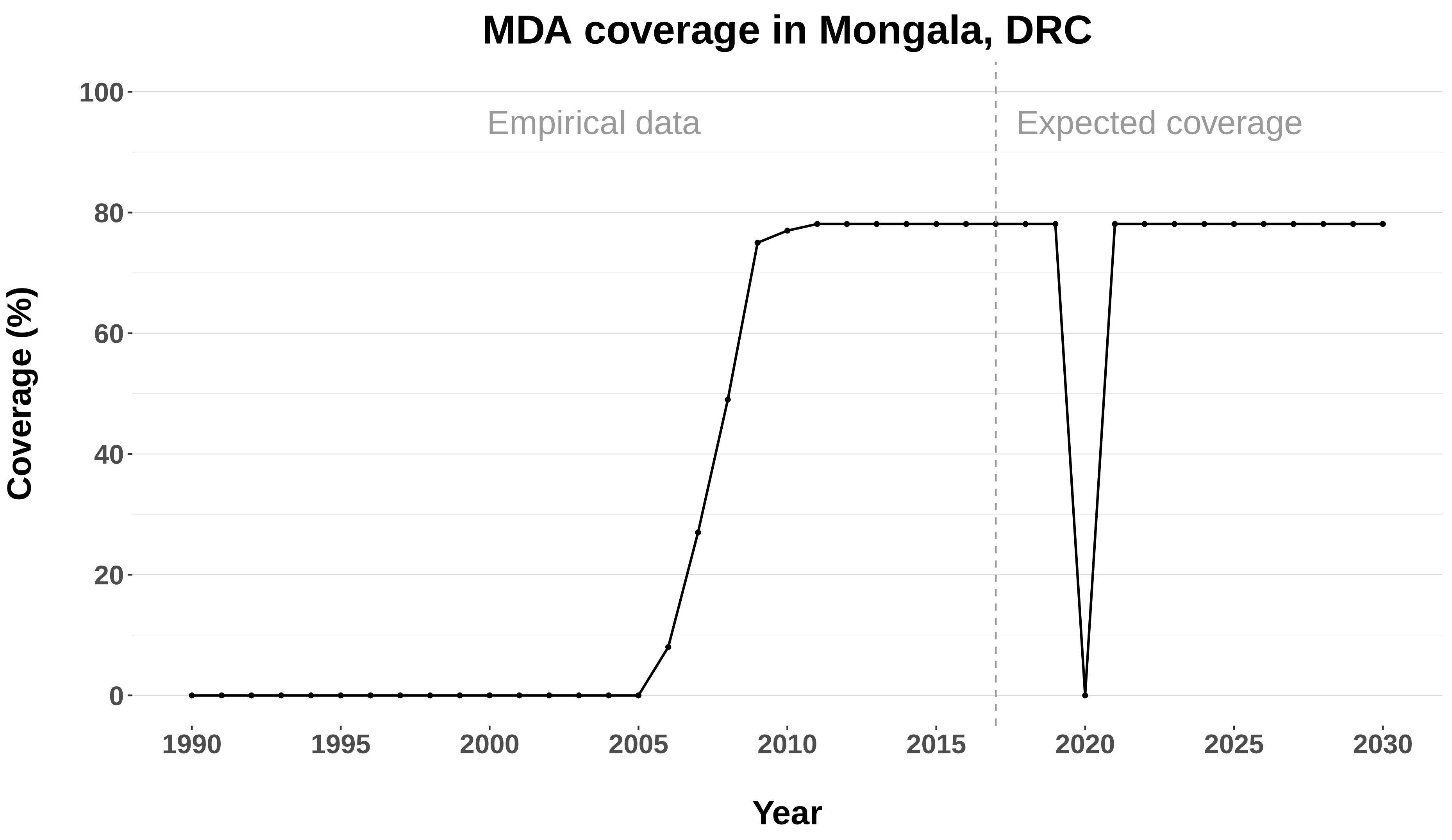
MDA coverage in Mongala, DRC
Empirical data
Expected coverage
Coverage (%)
100
80
60
40
20
0
1990
1995
2000
2005
2010
2015
2020
2025
2030
Year

**Project, country:**	**NY Katanga-Nord, DRC**
Bioclime:	
Endemicity level(s):	Mesoendemic
MDA start year:	2016
Mean MDA coverage per year:	66.3

	1990			2020			2030		
	N	%	DALYs	N	%	DALYs	N	%	DALYs
Total populaton at risk	243	100	_	549	100	_	720	100	_
**Infection:**									
Mf-positive	60	24.5	_	91	16.5	_	10	1.4	_
Mf-positive (age 5+)	60	24.5	_	91	16.5	_	10	1.4	_
Worm infected	78	32.2	_	150	27.4	_	58	8.0	_
**Clinical manifestations:**									
Palpable nodules	9	3.8	<0.5	16	2.9	<0.5	2	0.3	<0.5
Reversible skin disease:									
Severe itch	8	3.2	1	9	1.6	2	1	0.1	<0.5
Reactive skin disease	5	2.2	<0.5	3	0.5	<0.5	<0.5	<0.05	<0.5
**Reversible skin disease**	**13**	**5.4**	**2**	**11**	**2.0**	**2**	**1**	**0.1**	**<0.5**
Irreversible skin disease:									
Mild depigmentation	2	0.8	<0.5	4	0.7	<0.5	3	0.4	<0.5
Severe depigmentation	2	0.8	<0.5	3	0.6	<0.5	2	0.3	<0.5
Atrophy	<0.5	<0.05	<0.5	<0.5	<0.05	<0.5	<0.5	<0.05	<0.5
Hanging groin	<0.5	<0.05	<0.5	<0.5	<0.05	<0.5	<0.5	<0.05	<0.5
**Irreversible skin disease**	**4**	**1.6**	**<0.5**	**7**	**1.3**	**<0.5**	**5**	**0.7**	**<0.5**
**Total skin disease (OSD)**	**26**	**10.9**	**2**	**34**	**6.2**	**2**	**8**	**1.1**	**<0.5**
Eye disease (OED):									
Visual impairment	<0.5	<0.05	<0.5	<0.5	<0.05	<0.5	<0.5	<0.05	<0.5
Blindness	<0.5	<0.05	<0.5	<0.5	<0.05	<0.5	<0.5	<0.05	<0.5
**Eye disease (OED)**	**<0.5**	**<0.05**	**<0.5**	**<0.5**	**<0.05**	**<0.5**	**<0.5**	**<0.05**	**<0.5**
**Total all manifestations**	**26**	**10.9**	**2**	**34**	**6.2**	**2**	**8**	**1.2**	**<0.5**

Note 1: Population at risk, number of cases (N) and DALYs are presented in thousands.

Note 2: Total skin disease is defined as the sum of the number of cases with palpable nodules, reversible and irreversible skin disease.

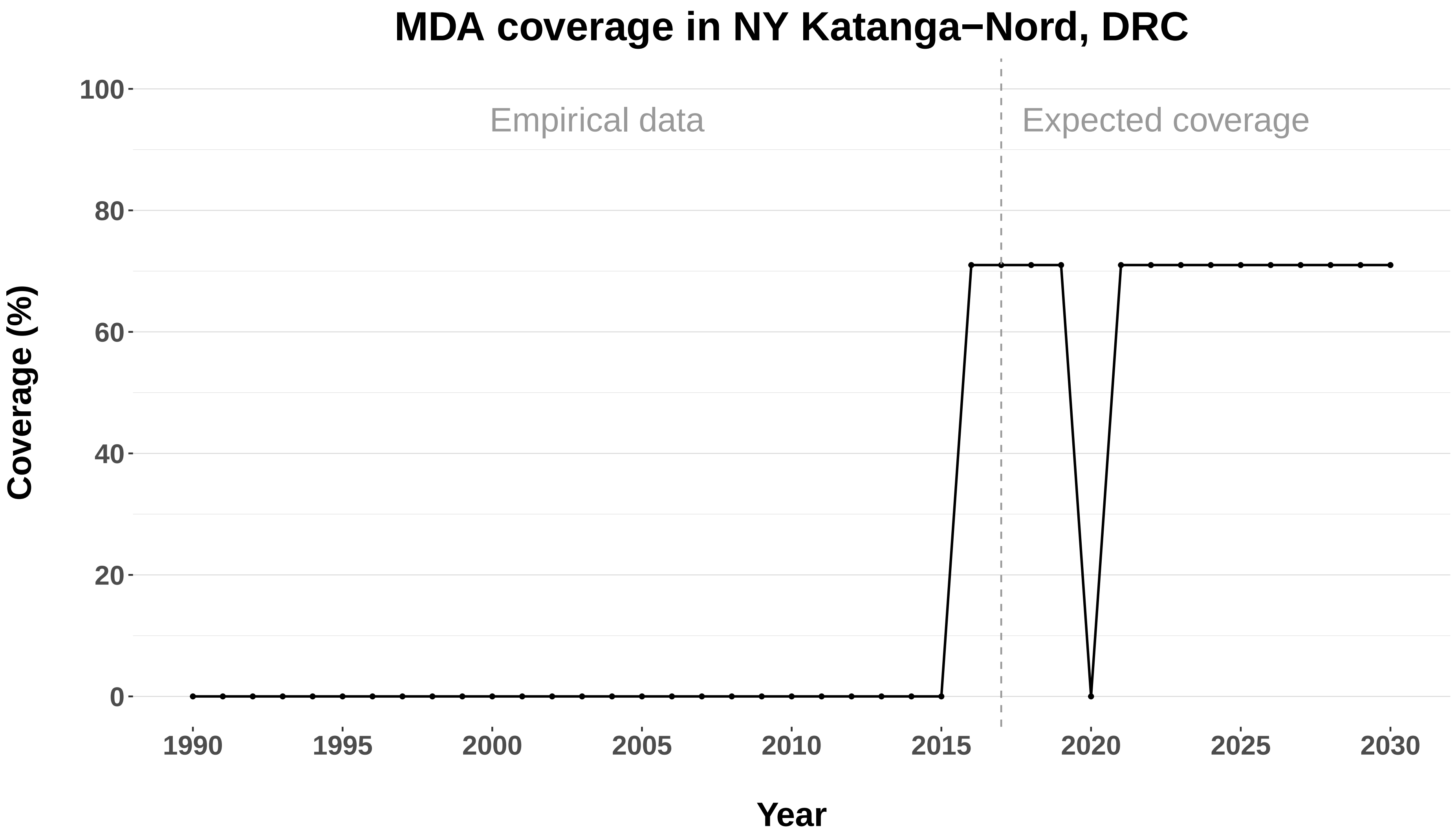
MDA coverage in NY Katanga−Nord, DRC
Empirical data
Expected coverage
Coverage (%)
0
20
40
60
80
100
1990
1995
2000
2005
2010
2015
2020
2025
2030
Year

**Project, country:**	**NY Lualaba, DRC**
Bioclime:	
Endemicity level(s):	Hyperendemic
MDA start year:	2016
Mean MDA coverage per year:	66.3

	1990			2020			2030		
	N	%	DALYs	N	%	DALYs	N	%	DALYs
Total populaton at risk	526	100	_	1,187	100	_	1,557	100	_
**Infection:**									
Mf-positive	146	27.7	_	229	19.3	_	27	1.7	_
Mf-positive (age 5+)	146	27.7	_	229	19.3	_	27	1.7	_
Worm infected	188	35.8	_	368	31.0	_	148	9.5	_
**Clinical manifestations:**									
Palpable nodules	27	5.1	<0.5	46	3.9	<0.5	5	0.3	<0.5
Reversible skin disease:									
Severe itch	20	3.7	4	22	1.8	4	2	0.1	<0.5
Reactive skin disease	14	2.6	1	6	0.5	<0.5	<0.5	<0.05	<0.5
**Reversible skin disease**	**33**	**6.3**	**4**	**28**	**2.4**	**4**	**2**	**0.1**	**<0.5**
Irreversible skin disease:									
Mild depigmentation	5	1.0	<0.5	9	0.8	<0.5	7	0.5	<0.5
Severe depigmentation	5	0.9	<0.5	8	0.7	1	6	0.4	<0.5
Atrophy	<0.5	<0.05	<0.5	<0.5	<0.05	<0.5	<0.5	<0.05	<0.5
Hanging groin	<0.5	<0.05	<0.5	<0.5	<0.05	<0.5	<0.5	<0.05	<0.5
**Irreversible skin disease**	**10**	**1.8**	**<0.5**	**18**	**1.5**	**1**	**13**	**0.9**	**<0.5**
**Total skin disease (OSD)**	**70**	**13.3**	**5**	**92**	**7.8**	**5**	**21**	**1.3**	**1**
Eye disease (OED):									
Visual impairment	<0.5	<0.05	<0.5	<0.5	<0.05	<0.5	<0.5	<0.05	<0.5
Blindness	<0.5	<0.05	<0.5	<0.5	<0.05	<0.5	<0.5	<0.05	<0.5
**Eye disease (OED)**	**<0.5**	**<0.05**	**<0.5**	**<0.5**	**<0.05**	**<0.5**	**<0.5**	**<0.05**	**<0.5**
**Total all manifestations**	**70**	**13.3**	**5**	**93**	**7.8**	**6**	**21**	**1.3**	**1**

Note 1: Population at risk, number of cases (N) and DALYs are presented in thousands.

Note 2: Total skin disease is defined as the sum of the number of cases with palpable nodules, reversible and irreversible skin disease.

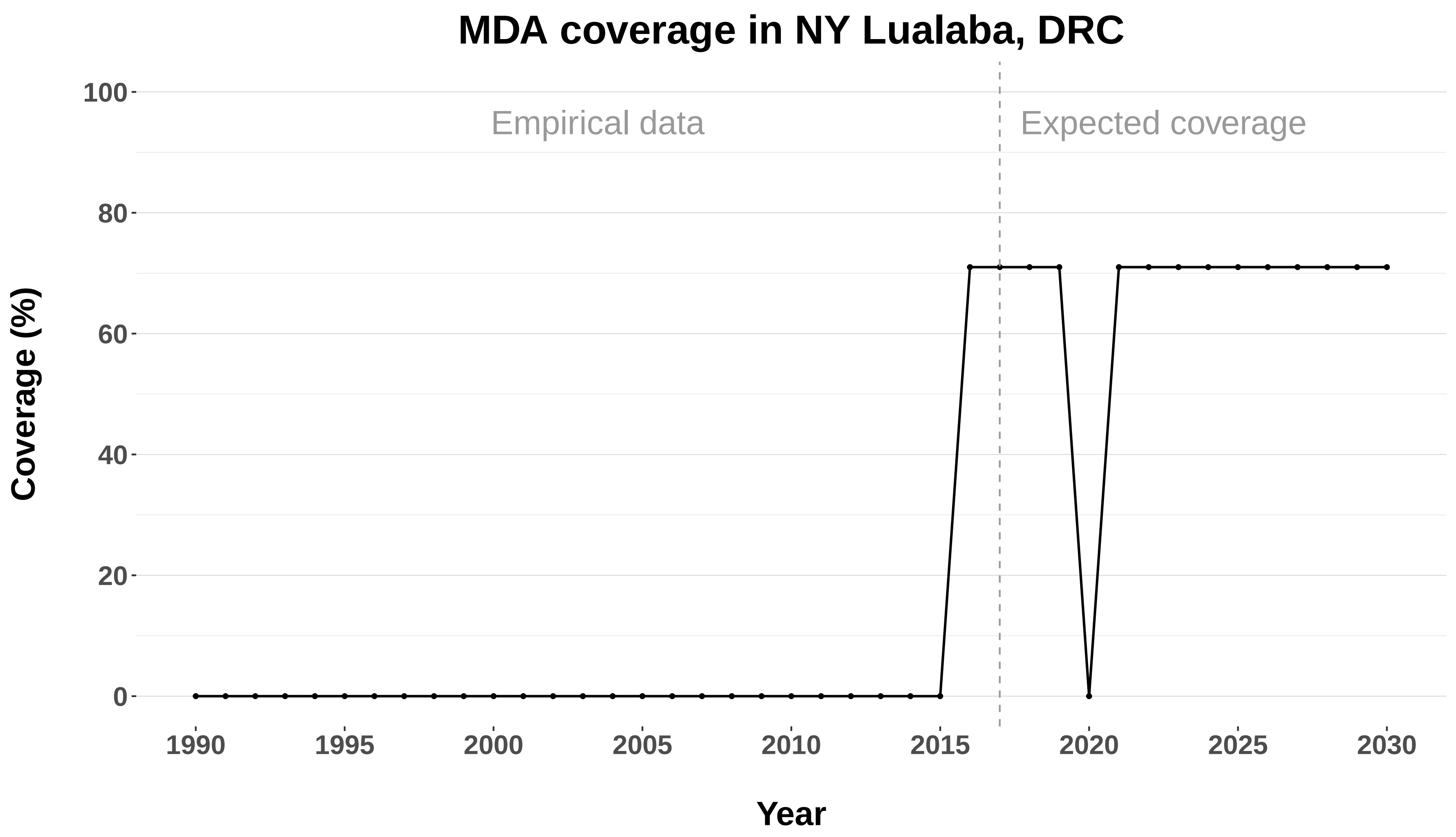
MDA coverage in NY Lualaba, DRC
Empirical data
Expected coverage
Coverage (%)
100
80
60
40
20
0
1990
1995
2000
2005
2010
2015
2020
2025
2030
Year

**Project, country:**	**NY Masisi-Walikale, DRC**
Bioclime:	
Endemicity level(s):	Hyperendemic
MDA start year:	2016
Mean MDA coverage per year:	67.1

	1990			2020			2030		
	N	%	DALYs	N	%	DALYs	N	%	DALYs
Total populaton at risk	29	100	_	66	100	_	86	100	_
**Infection:**									
Mf-positive	11	37.2	_	17	26.5	_	2	2.5	_
Mf-positive (age 5+)	11	37.2	_	17	26.5	_	2	2.5	_
Worm infected	14	47.0	_	27	41.4	_	11	13.1	_
**Clinical manifestations:**									
Palpable nodules	3	10.4	<0.5	5	8.0	<0.5	1	0.7	<0.5
Reversible skin disease:									
Severe itch	2	5.2	<0.5	2	2.7	<0.5	<0.5	0.2	<0.5
Reactive skin disease	1	3.7	<0.5	1	0.8	<0.5	<0.5	<0.05	<0.5
**Reversible skin disease**	**3**	**9.0**	**<0.5**	**2**	**3.5**	**<0.5**	**<0.5**	**0.2**	**<0.5**
Irreversible skin disease:									
Mild depigmentation	<0.5	1.2	<0.5	1	1.1	<0.5	1	0.6	<0.5
Severe depigmentation	<0.5	1.2	<0.5	1	1.0	<0.5	<0.5	0.6	<0.5
Atrophy	<0.5	<0.05	<0.5	<0.5	<0.05	<0.5	<0.5	<0.05	<0.5
Hanging groin	<0.5	<0.05	<0.5	<0.5	<0.05	<0.5	<0.5	<0.05	<0.5
**Irreversible skin disease**	**1**	**2.4**	**<0.5**	**1**	**2.0**	**<0.5**	**1**	**1.2**	**<0.5**
**Total skin disease (OSD)**	**6**	**21.8**	**<0.5**	**9**	**13.5**	**<0.5**	**2**	**2.1**	**<0.5**
Eye disease (OED):									
Visual impairment	<0.5	<0.05	<0.5	<0.5	<0.05	<0.5	<0.5	<0.05	<0.5
Blindness	<0.5	<0.05	<0.5	<0.5	<0.05	<0.5	<0.5	<0.05	<0.5
**Eye disease (OED)**	**<0.5**	**<0.05**	**<0.5**	**<0.5**	**<0.05**	**<0.5**	**<0.5**	**<0.05**	**<0.5**
**Total all manifestations**	**6**	**21.9**	**<0.5**	**9**	**13.6**	**1**	**2**	**2.1**	**<0.5**

Note 1: Population at risk, number of cases (N) and DALYs are presented in thousands.

Note 2: Total skin disease is defined as the sum of the number of cases with palpable nodules, reversible and irreversible skin disease.

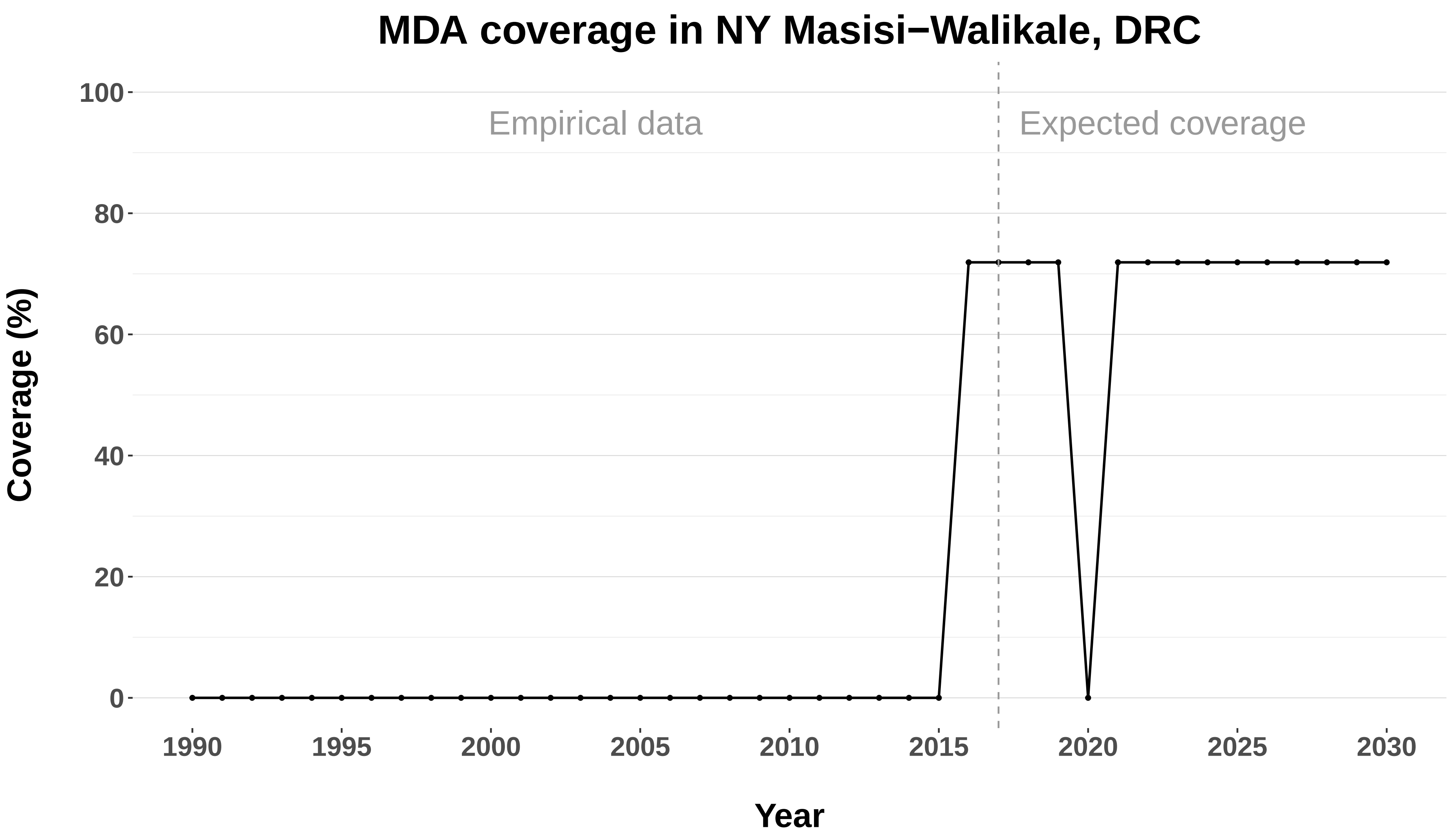

MDA coverage in NY Masisi−Walikale, DRC
Empirical data
Expected coverage
Coverage (%)
0
20
40
60
80
100
1990
1995
2000
2005
2010
2015
2020
2025
2030
Year

**Project, country:**	**NY Rutshuru-Ngoma, DRC**
Bioclime:	
Endemicity level(s):	Mesoendemic
MDA start year:	2013
Mean MDA coverage per year:	67.9

	1990			2020			2030		
	N	%	DALYs	N	%	DALYs	N	%	DALYs
Total populaton at risk	5	100	_	10	100	_	13	100	_
**Infection:**									
Mf-positive	2	50.8	_	2	24.3	_	<0.5	1.3	_
Mf-positive (age 5+)	2	50.8	_	2	24.3	_	<0.5	1.3	_
Worm infected	3	61.4	_	5	46.0	_	1	10.4	_
**Clinical manifestations:**									
Palpable nodules	1	18.8	<0.5	1	9.4	<0.5	<0.5	0.4	<0.5
Reversible skin disease:									
Severe itch	<0.5	8.1	<0.5	<0.5	2.3	<0.5	<0.5	0.1	<0.5
Reactive skin disease	<0.5	5.7	<0.5	<0.5	0.4	<0.5	<0.5	<0.05	<0.5
**Reversible skin disease**	**1**	**13.8**	**<0.5**	**<0.5**	**2.7**	**<0.5**	**<0.5**	**0.1**	**<0.5**
Irreversible skin disease:									
Mild depigmentation	<0.5	1.8	<0.5	<0.5	1.3	<0.5	<0.5	0.8	<0.5
Severe depigmentation	<0.5	1.9	<0.5	<0.5	1.3	<0.5	<0.5	0.7	<0.5
Atrophy	<0.5	<0.05	<0.5	<0.5	<0.05	<0.5	<0.5	<0.05	<0.5
Hanging groin	<0.5	<0.05	<0.5	<0.5	<0.05	<0.5	<0.5	<0.05	<0.5
**Irreversible skin disease**	**<0.5**	**3.7**	**<0.5**	**<0.5**	**2.6**	**<0.5**	**<0.5**	**1.5**	**<0.5**
**Total skin disease (OSD)**	**2**	**36.4**	**<0.5**	**2**	**14.8**	**<0.5**	**<0.5**	**2.0**	**<0.5**
Eye disease (OED):									
Visual impairment	<0.5	0.1	<0.5	<0.5	<0.05	<0.5	<0.5	<0.05	<0.5
Blindness	<0.5	<0.05	<0.5	<0.5	<0.05	<0.5	<0.5	<0.05	<0.5
**Eye disease (OED)**	**<0.5**	**0.1**	**<0.5**	**<0.5**	**0.1**	**<0.5**	**<0.5**	**<0.05**	**<0.5**
**Total all manifestations**	**2**	**36.5**	**<0.5**	**2**	**14.8**	**<0.5**	**<0.5**	**2.1**	**<0.5**

Note 1: Population at risk, number of cases (N) and DALYs are presented in thousands.

Note 2: Total skin disease is defined as the sum of the number of cases with palpable nodules, reversible and irreversible skin disease.

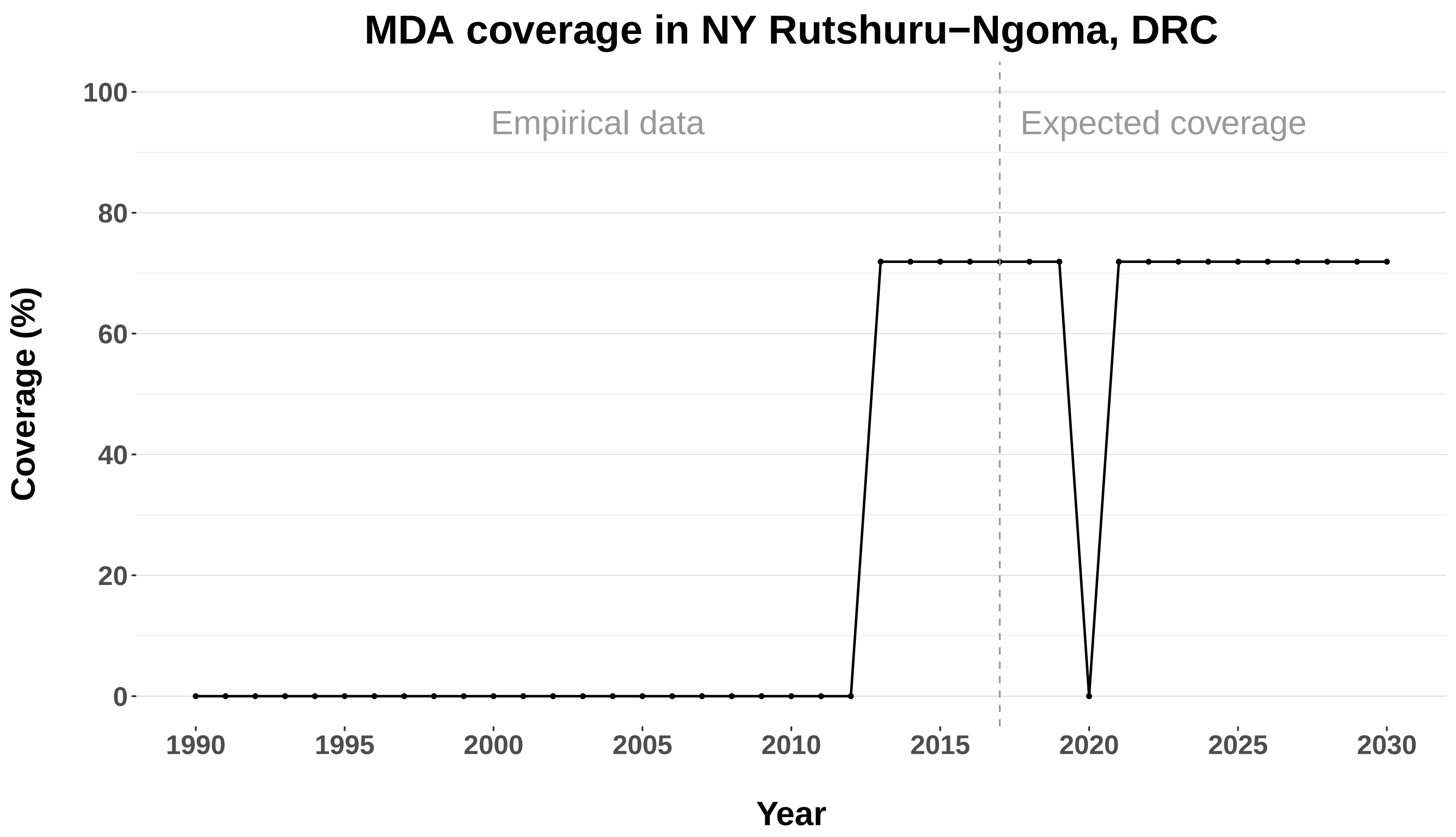

MDA coverage in NY Rutshuru−Ngoma, DRC
Empirical data
Expected coverage
Coverage (%)
100
80
60
40
20
0
1990
1995
2000
2005
2010
2015
2020
2025
2030
Year

**Project, country:**	**NY Sankuru, DRC**
Bioclime:	
Endemicity level(s):	Hyperendemic
MDA start year:	2014
Mean MDA coverage per year:	66.8

	1990			2020			2030		
	N	%	DALYs	N	%	DALYs	N	%	DALYs
Total populaton at risk	251	100	_	566	100	_	743	100	_
**Infection:**									
Mf-positive	70	27.9	_	83	14.6	_	10	1.3	_
Mf-positive (age 5+)	70	27.9	_	83	14.6	_	10	1.3	_
Worm infected	89	35.6	_	154	27.2	_	51	6.8	_
**Clinical manifestations:**									
Palpable nodules	15	5.9	<0.5	21	3.6	<0.5	3	0.4	<0.5
Reversible skin disease:									
Severe itch	10	4.1	2	8	1.4	1	1	0.1	<0.5
Reactive skin disease	7	2.9	<0.5	2	0.3	<0.5	<0.5	<0.05	<0.5
**Reversible skin disease**	**18**	**7.1**	**2**	**10**	**1.7**	**2**	**1**	**0.1**	**<0.5**
Irreversible skin disease:									
Mild depigmentation	2	1.0	<0.5	4	0.8	<0.5	3	0.4	<0.5
Severe depigmentation	3	1.0	<0.5	4	0.8	<0.5	3	0.4	<0.5
Atrophy	<0.5	<0.05	<0.5	<0.5	<0.05	<0.5	<0.5	<0.05	<0.5
Hanging groin	<0.5	<0.05	<0.5	<0.5	<0.05	<0.5	<0.5	<0.05	<0.5
**Irreversible skin disease**	**5**	**2.0**	**<0.5**	**9**	**1.5**	**<0.5**	**6**	**0.9**	**<0.5**
**Total skin disease (OSD)**	**38**	**15.0**	**3**	**39**	**6.9**	**2**	**10**	**1.4**	**<0.5**
Eye disease (OED):									
Visual impairment	<0.5	<0.05	<0.5	<0.5	<0.05	<0.5	<0.5	<0.05	<0.5
Blindness	<0.5	<0.05	1	<0.5	<0.05	1	<0.5	<0.05	<0.5
**Eye disease (OED)**	**<0.5**	**0.1**	**1**	**<0.5**	**0.1**	**1**	**<0.5**	**<0.05**	**<0.5**
**Total all manifestations**	**38**	**15.1**	**3**	**39**	**6.9**	**3**	**10**	**1.4**	**1**

Note 1: Population at risk, number of cases (N) and DALYs are presented in thousands.

Note 2: Total skin disease is defined as the sum of the number of cases with palpable nodules, reversible and irreversible skin disease.

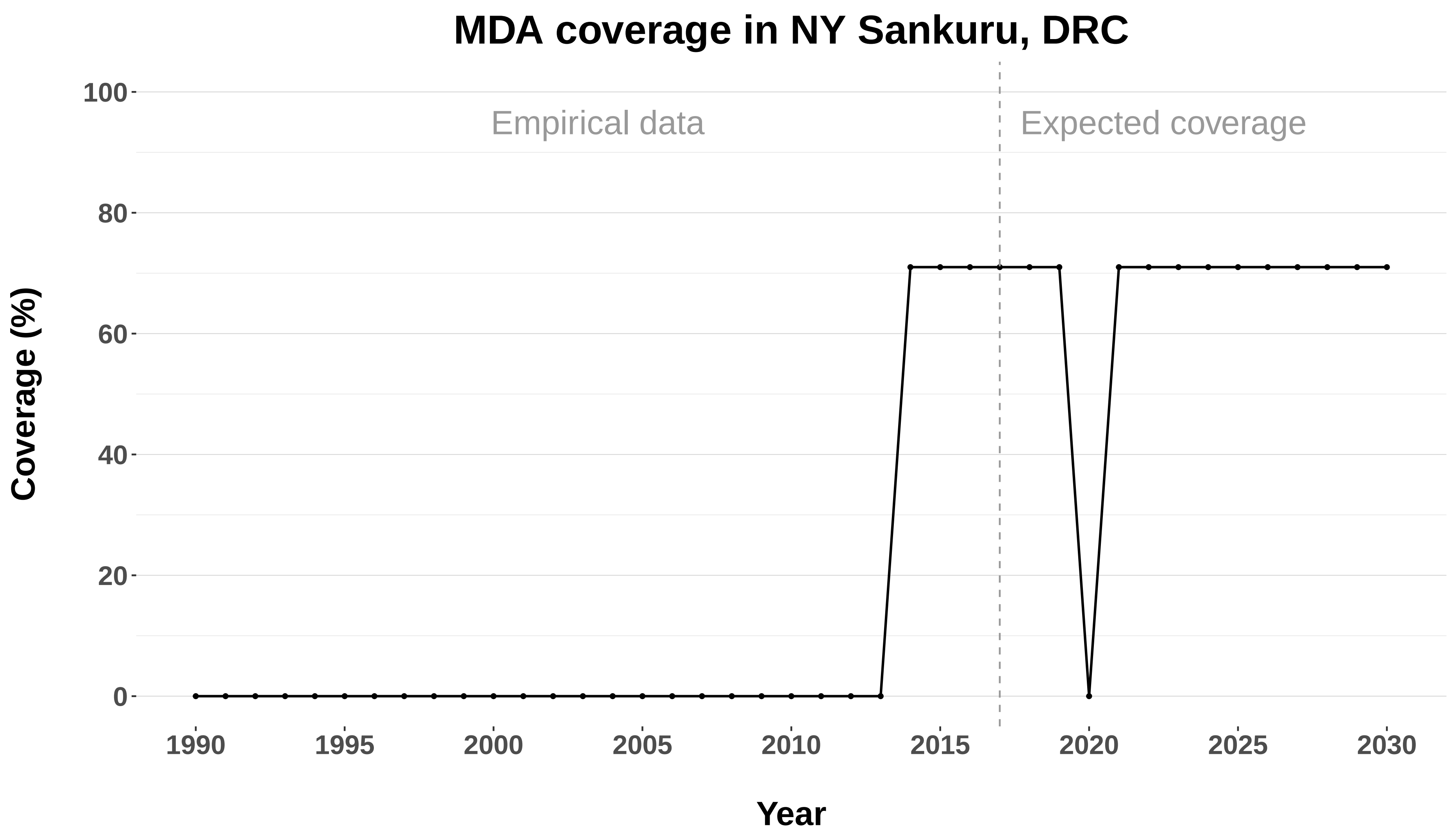
MDA coverage in NY Sankuru, DRC
Empirical data
Expected coverage
Coverage (%)
100
80
60
40
20
0
1990
1995
2000
2005
2010
2015
2020
2025
2030
Year

**Project, country:**	**NY Ueles, DRC**
Bioclime:	
Endemicity level(s):	Hyperendemic
MDA start year:	2014
Mean MDA coverage per year:	67.7

	1990			2020			2030		
	N	%	DALYs	N	%	DALYs	N	%	DALYs
Total populaton at risk	85	100	_	193	100	_	253	100	_
**Infection:**									
Mf-positive	50	59.0	_	81	42.1	_	36	14.4	_
Mf-positive (age 5+)	50	58.7	_	81	42.1	_	36	14.3	_
Worm infected	57	66.4	_	110	57.3	_	79	31.3	_
**Clinical manifestations:**									
Palpable nodules	28	32.7	<0.5	47	24.3	<0.5	15	5.9	<0.5
Reversible skin disease:									
Severe itch	12	14.4	2	13	6.7	2	2	1.0	<0.5
Reactive skin disease	11	12.6	<0.5	4	2.3	<0.5	<0.5	0.1	<0.5
**Reversible skin disease**	**23**	**27.0**	**3**	**17**	**9.0**	**3**	**3**	**1.1**	**<0.5**
Irreversible skin disease:									
Mild depigmentation	2	2.7	<0.5	4	2.2	<0.5	3	1.4	<0.5
Severe depigmentation	4	4.6	<0.5	7	3.6	<0.5	5	2.1	<0.5
Atrophy	<0.5	0.1	<0.5	<0.5	<0.05	<0.5	<0.5	<0.05	<0.5
Hanging groin	<0.5	0.3	<0.5	<0.5	0.2	<0.5	<0.5	0.1	<0.5
**Irreversible skin disease**	**7**	**7.8**	**<0.5**	**12**	**6.0**	**1**	**9**	**3.6**	**<0.5**
**Total skin disease (OSD)**	**58**	**67.5**	**3**	**76**	**39.2**	**4**	**27**	**10.6**	**1**
Eye disease (OED):									
Visual impairment	<0.5	0.4	<0.5	1	0.3	<0.5	<0.5	0.2	<0.5
Blindness	1	0.6	4	1	0.4	5	<0.5	0.1	2
**Eye disease (OED)**	**1**	**1.0**	**4**	**1**	**0.7**	**5**	**1**	**0.3**	**2**
**Total all manifestations**	**58**	**68.5**	**7**	**77**	**39.9**	**9**	**27**	**10.9**	**3**

Note 1: Population at risk, number of cases (N) and DALYs are presented in thousands.

Note 2: Total skin disease is defined as the sum of the number of cases with palpable nodules, reversible and irreversible skin disease.

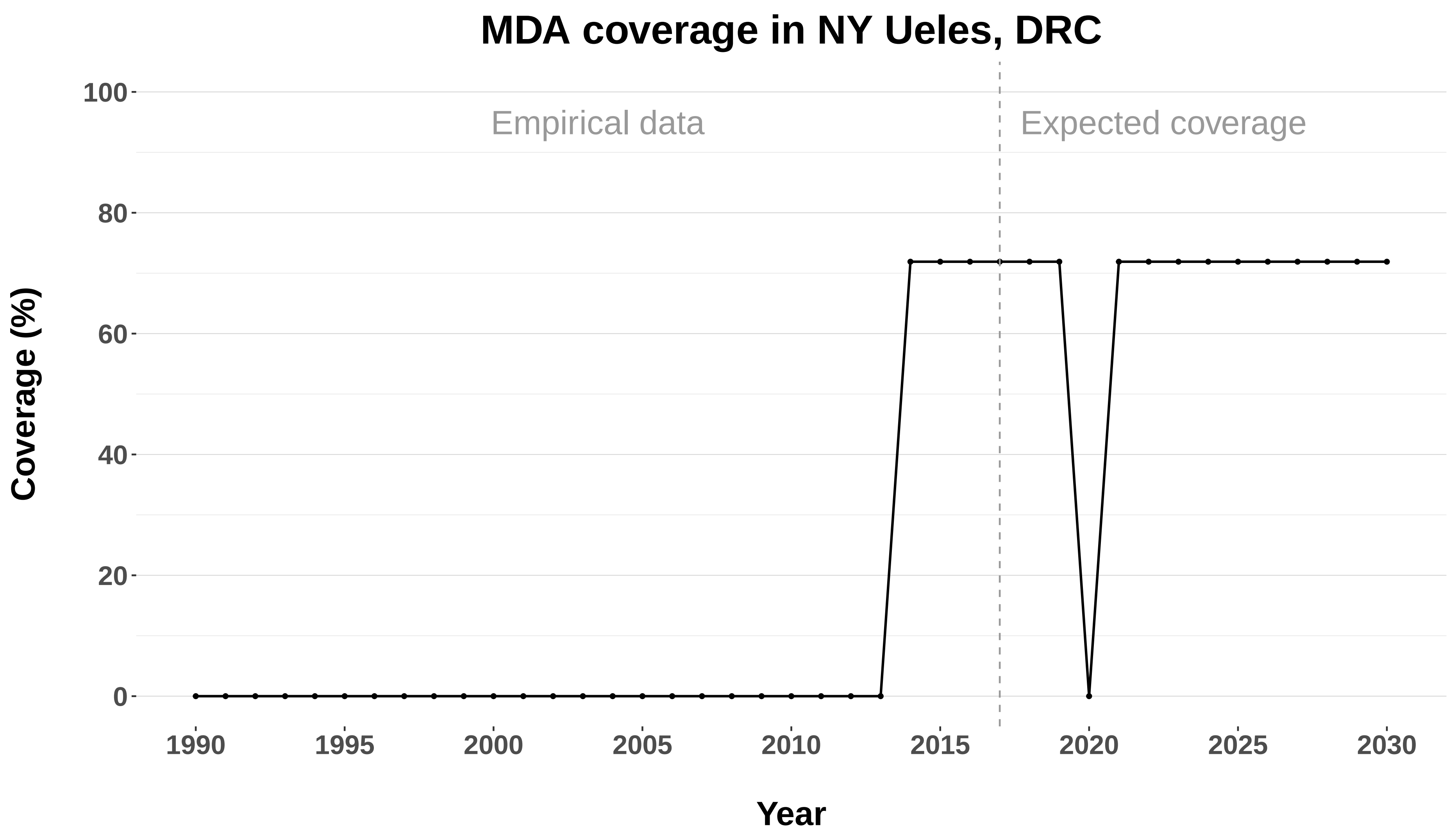
MDA coverage in NY Ueles, DRC
Empirical data
Expected coverage
Coverage (%)
100
80
60
40
20
0
1990
1995
2000
2005
2010
2015
2020
2025
2030
Year

**Project, country:**	**P20DRC, DRC**
Bioclime:	
Endemicity level(s):	Hyperendemic
MDA start year:	2019
Mean MDA coverage per year:	65.9

	1990			2020			2030		
	N	%	DALYs	N	%	DALYs	N	%	DALYs
Total populaton at risk	1,259	100	_	2,841	100	_	3,726	100	_
**Infection:**									
Mf-positive	592	47.0	_	1,268	44.6	_	518	13.9	_
Mf-positive (age 5+)	591	46.9	_	1,266	44.6	_	518	13.9	_
Worm infected	712	56.5	_	1,591	56.0	_	1,201	32.2	_
**Clinical manifestations:**									
Palpable nodules	238	18.9	3	532	18.7	6	219	5.9	2
Reversible skin disease:									
Severe itch	107	8.5	19	210	7.4	38	50	1.3	9
Reactive skin disease	83	6.6	4	141	5.0	7	7	0.2	<0.5
**Reversible skin disease**	**190**	**15.1**	**23**	**352**	**12.4**	**45**	**57**	**1.5**	**9**
Irreversible skin disease:									
Mild depigmentation	22	1.8	<0.5	48	1.7	1	41	1.1	<0.5
Severe depigmentation	28	2.2	2	62	2.2	4	51	1.4	3
Atrophy	<0.5	<0.05	<0.5	1	<0.05	<0.5	<0.5	<0.05	<0.5
Hanging groin	1	0.1	<0.5	2	0.1	1	1	<0.05	1
**Irreversible skin disease**	**52**	**4.1**	**2**	**114**	**4.0**	**5**	**94**	**2.5**	**4**
**Total skin disease (OSD)**	**480**	**38.1**	**28**	**997**	**35.1**	**56**	**370**	**9.9**	**16**
Eye disease (OED):									
Visual impairment	2	0.1	<0.5	3	0.1	<0.5	3	0.1	<0.5
Blindness	2	0.2	14	4	0.1	28	2	0.1	15
**Eye disease (OED)**	**4**	**0.3**	**14**	**8**	**0.3**	**28**	**5**	**0.1**	**15**
**Total all manifestations**	**484**	**38.4**	**42**	**1,005**	**35.4**	**84**	**375**	**10.1**	**31**

Note 1: Population at risk, number of cases (N) and DALYs are presented in thousands.

Note 2: Total skin disease is defined as the sum of the number of cases with palpable nodules, reversible and irreversible skin disease.

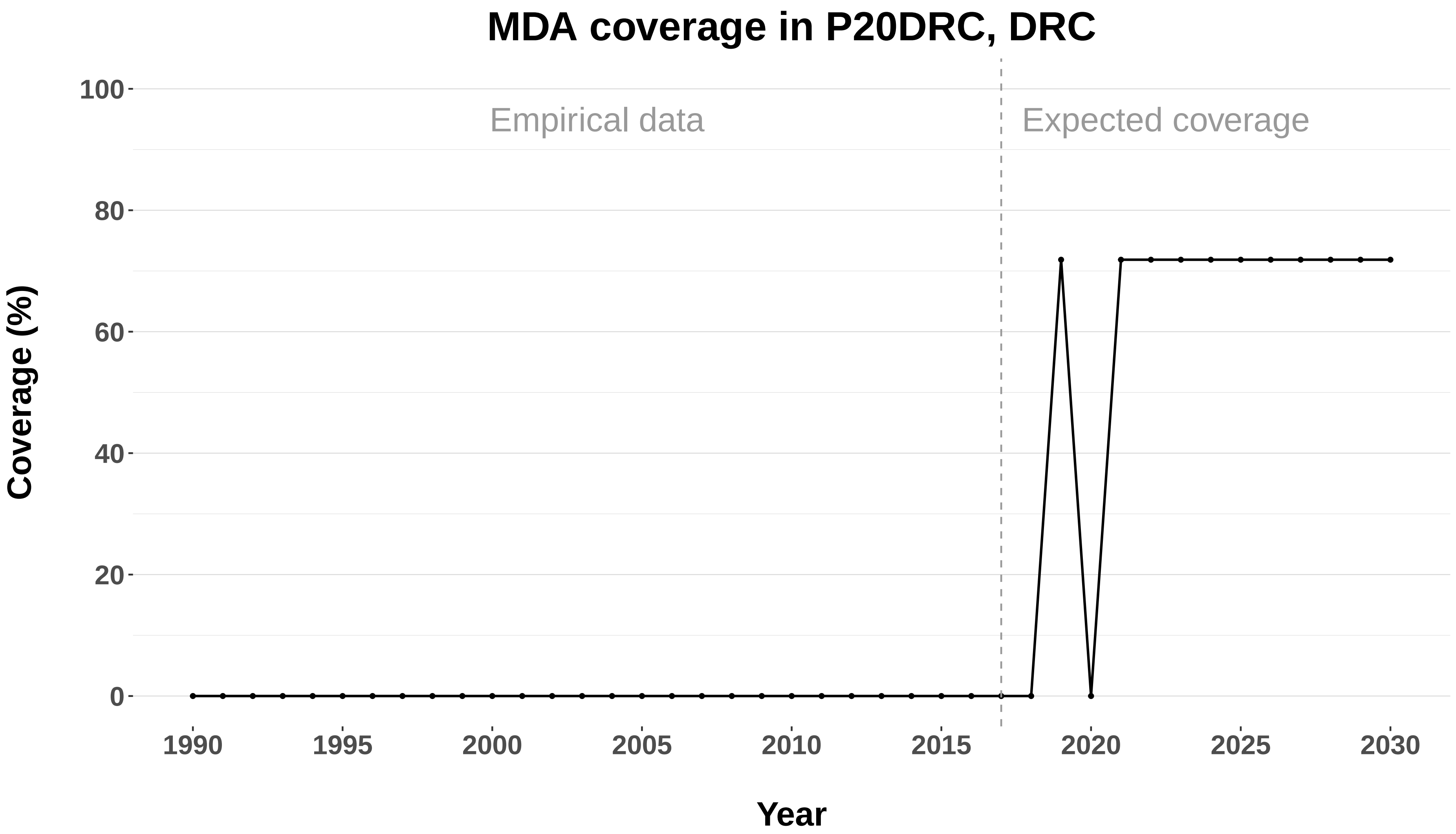
MDA coverage in P20DRC, DRC
Empirical data
Expected coverage
Coverage (%)
100
80
60
40
20
0
1990
1995
2000
2005
2010
2015
2020
2025
2030
Year

**Project, country:**	**P5DRC, DRC**
Bioclime:	
Endemicity level(s):	Hypoendemic
MDA start year:	2025
Mean MDA coverage per year:	65.0

	1990			2020			2030		
	N	%	DALYs	N	%	DALYs	N	%	DALYs
Total populaton at risk	4,003	100	_	9,033	100	_	11,848	100	_
**Infection:**									
Mf-positive	975	24.4	_	2,113	23.4	_	1,732	14.6	_
Mf-positive (age 5+)	974	24.3	_	2,111	23.4	_	1,732	14.6	_
Worm infected	1,294	32.3	_	2,820	31.2	_	3,069	25.9	_
**Clinical manifestations:**									
Palpable nodules	148	3.7	2	321	3.6	3	287	2.4	3
Reversible skin disease:									
Severe itch	125	3.1	23	268	3.0	49	154	1.3	28
Reactive skin disease	84	2.1	4	180	2.0	8	42	0.4	2
**Reversible skin disease**	**209**	**5.2**	**27**	**448**	**5.0**	**57**	**196**	**1.7**	**30**
Irreversible skin disease:									
Mild depigmentation	32	0.8	<0.5	72	0.8	1	79	0.7	1
Severe depigmentation	29	0.7	2	62	0.7	4	64	0.5	4
Atrophy	<0.5	<0.05	<0.5	<0.5	<0.05	<0.5	<0.5	<0.05	<0.5
Hanging groin	<0.5	<0.05	<0.5	<0.5	<0.05	<0.5	<0.5	<0.05	<0.5
**Irreversible skin disease**	**62**	**1.5**	**2**	**134**	**1.5**	**5**	**143**	**1.2**	**5**
**Total skin disease (OSD)**	**418**	**10.4**	**30**	**903**	**10.0**	**65**	**627**	**5.3**	**38**
Eye disease (OED):									
Visual impairment	1	<0.05	<0.5	1	<0.05	<0.5	1	<0.05	<0.5
Blindness	<0.5	<0.05	2	1	<0.05	4	<0.5	<0.05	2
**Eye disease (OED)**	**1**	**<0.05**	**2**	**2**	**<0.05**	**4**	**2**	**<0.05**	**2**
**Total all manifestations**	**419**	**10.5**	**32**	**905**	**10.0**	**70**	**629**	**5.3**	**40**

Note 1: Population at risk, number of cases (N) and DALYs are presented in thousands.

Note 2: Total skin disease is defined as the sum of the number of cases with palpable nodules, reversible and irreversible skin disease.

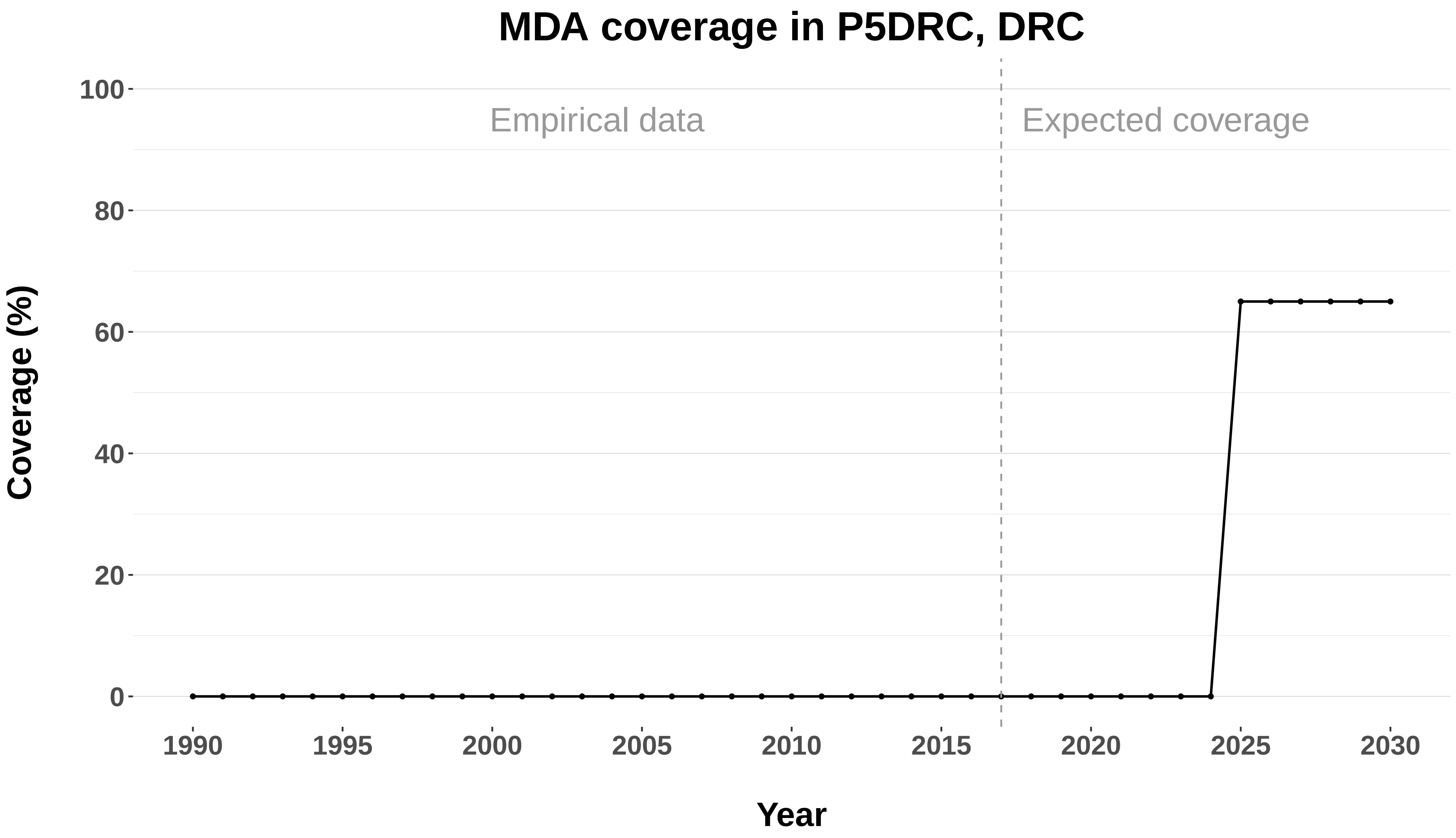

MDA coverage in P5DRC, DRC
Empirical data
Expected coverage
Coverage (%)
100
80
60
40
20
0
1990
1995
2000
2005
2010
2015
2020
2025
2030
Year

**Project, country:**	**Rutshuru-Ngoma, DRC**
Bioclime:	
Endemicity level(s):	Hyperendemic
MDA start year:	2006
Mean MDA coverage per year:	65.2

	1990			2020			2030		
	N	%	DALYs	N	%	DALYs	N	%	DALYs
Total populaton at risk	353	100	_	796	100	_	1,045	100	_
**Infection:**									
Mf-positive	138	39.2	_	28	3.5	_	<0.5	<0.05	_
Mf-positive (age 5+)	138	39.1	_	28	3.5	_	<0.5	<0.05	_
Worm infected	176	49.9	_	137	17.1	_	11	1.0	_
**Clinical manifestations:**									
Palpable nodules	40	11.2	<0.5	9	1.1	<0.5	<0.5	<0.05	<0.5
Reversible skin disease:									
Severe itch	19	5.4	3	2	0.3	<0.5	<0.5	<0.05	<0.5
Reactive skin disease	13	3.8	1	<0.5	<0.05	<0.5	<0.5	<0.05	<0.5
**Reversible skin disease**	**32**	**9.2**	**4**	**3**	**0.3**	**<0.5**	**<0.5**	**<0.05**	**<0.5**
Irreversible skin disease:									
Mild depigmentation	4	1.3	<0.5	5	0.7	<0.5	4	0.4	<0.5
Severe depigmentation	4	1.2	<0.5	5	0.6	<0.5	3	0.3	<0.5
Atrophy	<0.5	<0.05	<0.5	<0.5	<0.05	<0.5	<0.5	<0.05	<0.5
Hanging groin	<0.5	<0.05	<0.5	<0.5	<0.05	<0.5	<0.5	<0.05	<0.5
**Irreversible skin disease**	**9**	**2.5**	**<0.5**	**10**	**1.3**	**<0.5**	**7**	**0.6**	**<0.5**
**Total skin disease (OSD)**	**81**	**22.9**	**5**	**21**	**2.7**	**1**	**7**	**0.7**	**<0.5**
Eye disease (OED):									
Visual impairment	<0.5	<0.05	<0.5	<0.5	<0.05	<0.5	<0.5	<0.05	<0.5
Blindness	<0.5	<0.05	<0.5	<0.5	<0.05	<0.5	<0.5	<0.05	<0.5
**Eye disease (OED)**	**<0.5**	**<0.05**	**<0.5**	**<0.5**	**<0.05**	**<0.5**	**<0.5**	**<0.05**	**<0.5**
**Total all manifestations**	**81**	**22.9**	**5**	**21**	**2.7**	**1**	**7**	**0.7**	**<0.5**

Note 1: Population at risk, number of cases (N) and DALYs are presented in thousands.

Note 2: Total skin disease is defined as the sum of the number of cases with palpable nodules, reversible and irreversible skin disease.

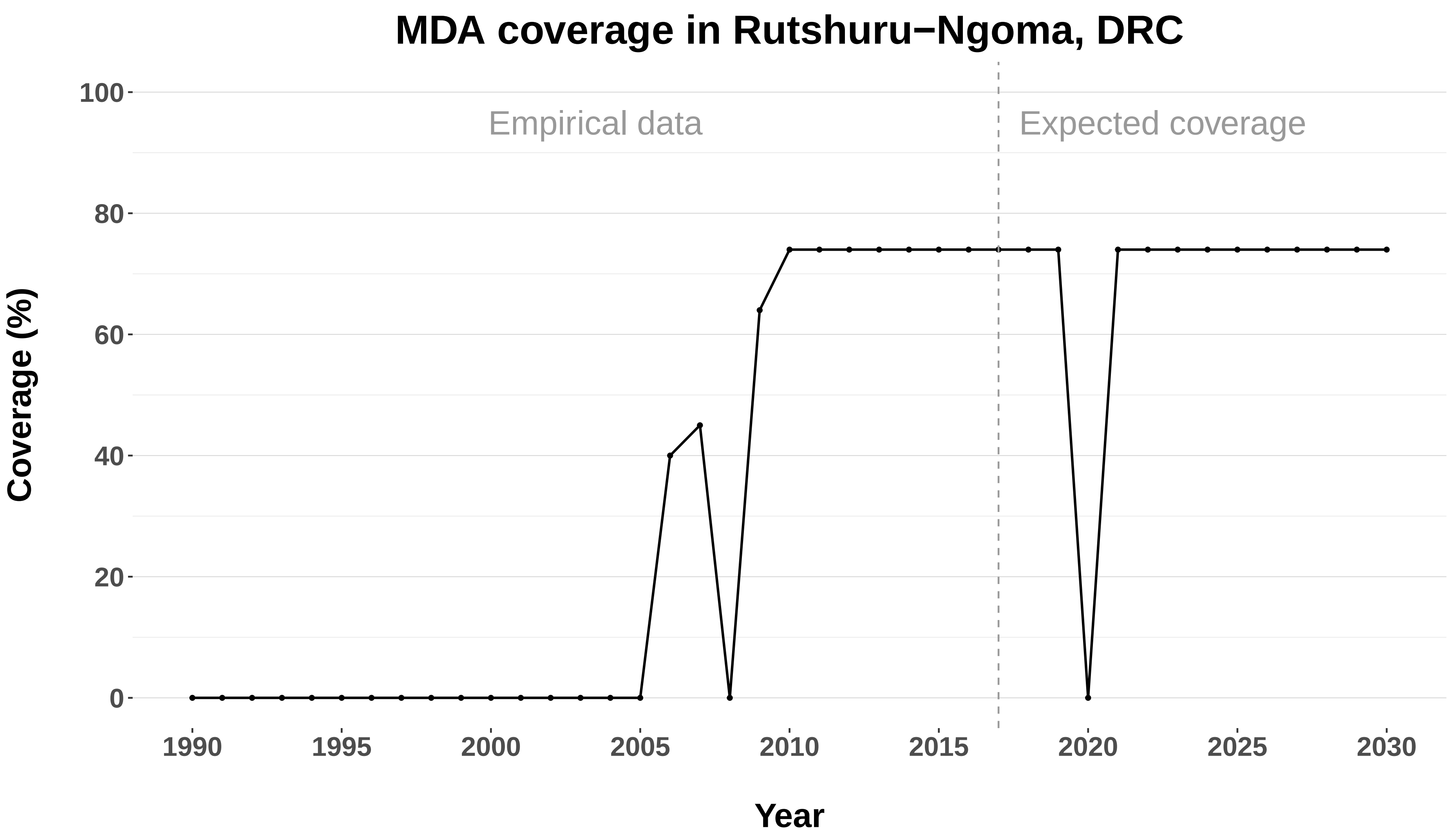
MDA coverage in Rutshuru−Ngoma, DRC
Empirical data
Expected coverage
Coverage (%)
0
20
40
60
80
100
Year
1990
1995
2000
2005
2010
2015
2020
2025
2030

**Project, country:**	**Sankuru, DRC**
Bioclime:	
Endemicity level(s):	Hyperendemic
MDA start year:	2003
Mean MDA coverage per year:	71.3

	1990			2020			2030		
	N	%	DALYs	N	%	DALYs	N	%	DALYs
Total populaton at risk	571	100	_	1,289	100	_	1,690	100	_
**Infection:**									
Mf-positive	270	47.2	_	98	7.6	_	13	0.8	_
Mf-positive (age 5+)	268	47.0	_	98	7.6	_	13	0.8	_
Worm infected	312	54.6	_	271	21.1	_	83	4.9	_
**Clinical manifestations:**									
Palpable nodules	133	23.2	1	48	3.7	1	5	0.3	<0.5
Reversible skin disease:									
Severe itch	62	10.9	11	9	0.7	2	<0.5	<0.05	<0.5
Reactive skin disease	54	9.4	2	1	<0.05	<0.5	<0.5	<0.05	<0.5
**Reversible skin disease**	**116**	**20.3**	**14**	**9**	**0.7**	**2**	**<0.5**	**<0.05**	**<0.5**
Irreversible skin disease:									
Mild depigmentation	12	2.1	<0.5	13	1.0	<0.5	10	0.6	<0.5
Severe depigmentation	20	3.5	1	21	1.6	1	14	0.8	1
Atrophy	1	0.1	<0.5	<0.5	<0.05	<0.5	<0.5	<0.05	<0.5
Hanging groin	2	0.3	1	1	0.1	<0.5	1	<0.05	<0.5
**Irreversible skin disease**	**34**	**6.0**	**2**	**35**	**2.7**	**2**	**25**	**1.5**	**1**
**Total skin disease (OSD)**	**283**	**49.5**	**17**	**92**	**7.2**	**4**	**30**	**1.8**	**1**
Eye disease (OED):									
Visual impairment	2	0.3	<0.5	2	0.1	<0.5	1	0.1	<0.5
Blindness	3	0.5	20	1	0.1	11	1	<0.05	4
**Eye disease (OED)**	**5**	**0.8**	**20**	**3**	**0.2**	**11**	**2**	**0.1**	**4**
**Total all manifestations**	**287**	**50.3**	**37**	**96**	**7.4**	**15**	**32**	**1.9**	**6**

Note 1: Population at risk, number of cases (N) and DALYs are presented in thousands.

Note 2: Total skin disease is defined as the sum of the number of cases with palpable nodules, reversible and irreversible skin disease.

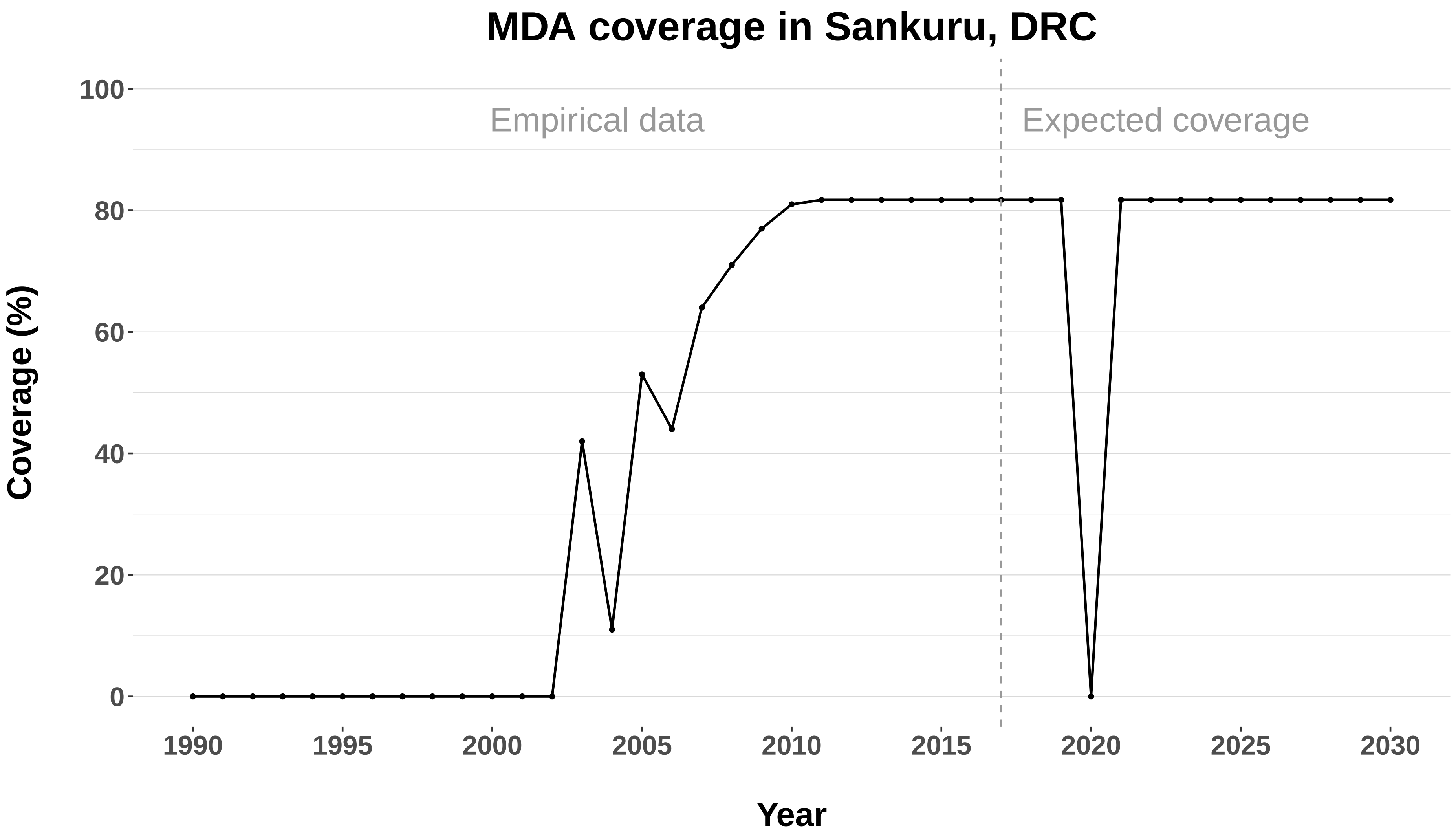
MDA coverage in Sankuru, DRC
Empirical data
Expected coverage
Coverage (%)
100
80
60
40
20
0
1990
1995
2000
2005
2010
2015
2020
2025
2030
Year

**Project, country:**	**Tshopo, DRC**
Bioclime:	
Endemicity level(s):	Hyperendemic
MDA start year:	2003
Mean MDA coverage per year:	54.0

	1990			2020			2030		
	N	%	DALYs	N	%	DALYs	N	%	DALYs
Total populaton at risk	853	100	_	1,925	100	_	2,525	100	_
**Infection:**									
Mf-positive	438	51.4	_	296	15.4	_	92	3.6	_
Mf-positive (age 5+)	437	51.2	_	296	15.4	_	92	3.6	_
Worm infected	512	60.1	_	630	32.7	_	279	11.0	_
**Clinical manifestations:**									
Palpable nodules	200	23.4	2	124	6.4	1	31	1.2	<0.5
Reversible skin disease:									
Severe itch	89	10.4	16	27	1.4	5	4	0.1	1
Reactive skin disease	72	8.5	3	6	0.3	<0.5	1	<0.05	<0.5
**Reversible skin disease**	**161**	**18.9**	**19**	**33**	**1.7**	**5**	**5**	**0.2**	**1**
Irreversible skin disease:									
Mild depigmentation	18	2.1	<0.5	24	1.2	<0.5	18	0.7	<0.5
Severe depigmentation	26	3.0	2	31	1.6	2	22	0.9	1
Atrophy	1	0.1	<0.5	<0.5	<0.05	<0.5	<0.5	<0.05	<0.5
Hanging groin	1	0.1	<0.5	1	0.1	<0.5	1	<0.05	<0.5
**Irreversible skin disease**	**45**	**5.3**	**2**	**56**	**2.9**	**3**	**40**	**1.6**	**2**
**Total skin disease (OSD)**	**406**	**47.6**	**24**	**214**	**11.1**	**9**	**76**	**3.0**	**3**
Eye disease (OED):									
Visual impairment	2	0.2	<0.5	2	0.1	<0.5	1	0.1	<0.5
Blindness	2	0.3	17	2	0.1	12	1	<0.05	5
**Eye disease (OED)**	**4**	**0.5**	**17**	**4**	**0.2**	**12**	**2**	**0.1**	**5**
**Total all manifestations**	**410**	**48.1**	**41**	**217**	**11.3**	**21**	**78**	**3.1**	**8**

Note 1: Population at risk, number of cases (N) and DALYs are presented in thousands.

Note 2: Total skin disease is defined as the sum of the number of cases with palpable nodules, reversible and irreversible skin disease.

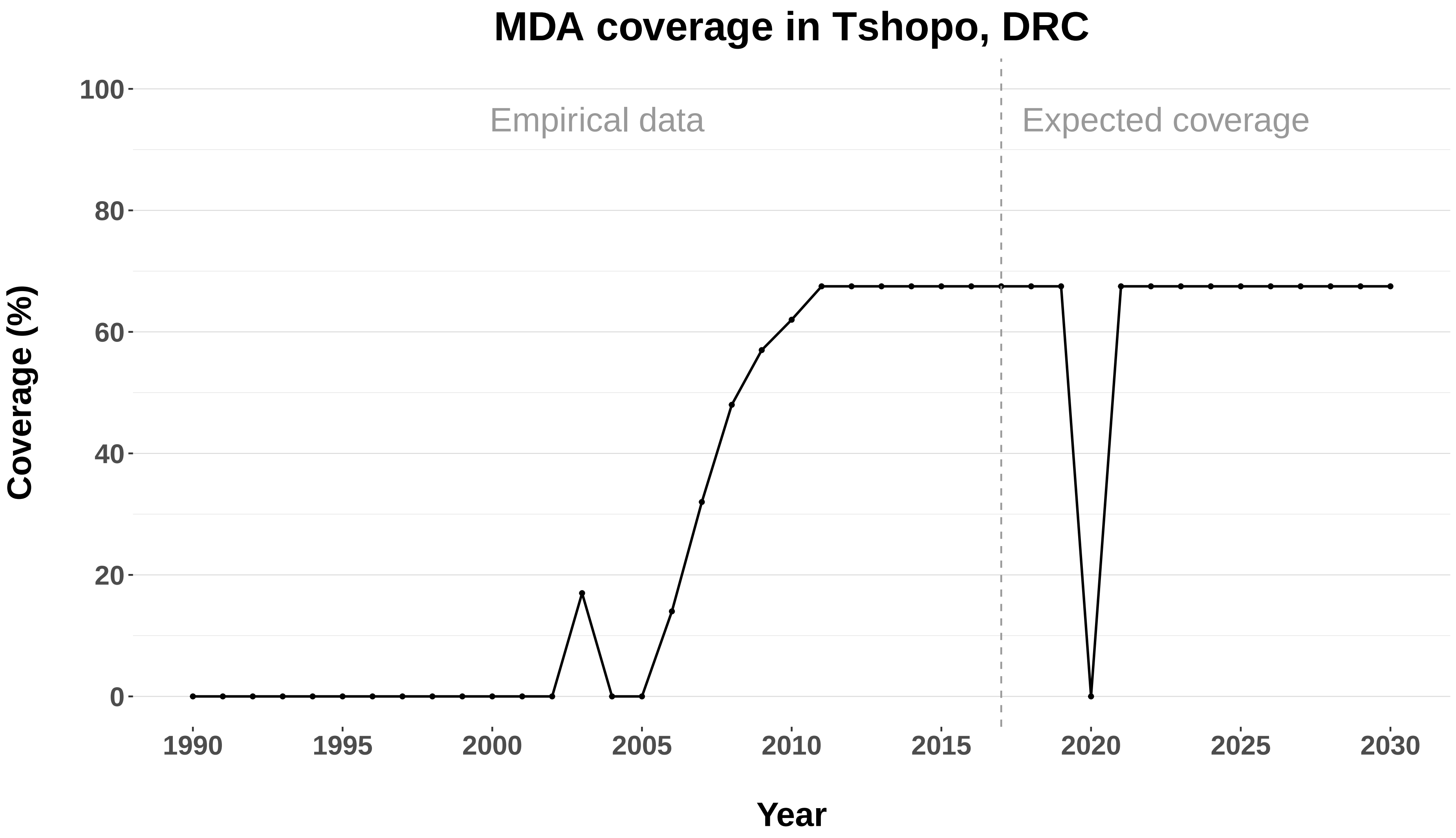
MDA coverage in Tshopo, DRC
Empirical data
Expected coverage
Coverage (%)
100
80
60
40
20
0
1990
1995
2000
2005
2010
2015
2020
2025
2030
Year

**Project, country:**	**Tshuapa, DRC**
Bioclime:	
Endemicity level(s):	Hyperendemic
MDA start year:	2006
Mean MDA coverage per year:	57.2

	1990			2020			2030		
	N	%	DALYs	N	%	DALYs	N	%	DALYs
Total populaton at risk	760	100	_	1,715	100	_	2,249	100	_
**Infection:**									
Mf-positive	381	50.2	_	264	15.4	_	67	3.0	_
Mf-positive (age 5+)	380	50.1	_	264	15.4	_	67	3.0	_
Worm infected	449	59.0	_	570	33.2	_	229	10.2	_
**Clinical manifestations:**									
Palpable nodules	165	21.7	2	105	6.1	1	22	1.0	<0.5
Reversible skin disease:									
Severe itch	74	9.7	13	25	1.5	5	3	0.1	1
Reactive skin disease	59	7.8	3	5	0.3	<0.5	1	<0.05	<0.5
**Reversible skin disease**	**133**	**17.5**	**16**	**30**	**1.8**	**5**	**4**	**0.2**	**1**
Irreversible skin disease:									
Mild depigmentation	15	2.0	<0.5	21	1.2	<0.5	16	0.7	<0.5
Severe depigmentation	21	2.7	1	27	1.6	2	19	0.8	1
Atrophy	<0.5	<0.05	<0.5	<0.5	<0.05	<0.5	<0.5	<0.05	<0.5
Hanging groin	1	0.1	<0.5	1	<0.05	<0.5	<0.5	<0.05	<0.5
**Irreversible skin disease**	**37**	**4.9**	**2**	**49**	**2.9**	**2**	**35**	**1.6**	**2**
**Total skin disease (OSD)**	**335**	**44.0**	**20**	**184**	**10.8**	**8**	**61**	**2.7**	**2**
Eye disease (OED):									
Visual impairment	1	0.2	<0.5	1	0.1	<0.5	1	<0.05	<0.5
Blindness	2	0.2	11	1	0.1	9	<0.5	<0.05	3
**Eye disease (OED)**	**3**	**0.4**	**11**	**3**	**0.2**	**9**	**1**	**0.1**	**3**
**Total all manifestations**	**337**	**44.4**	**30**	**187**	**10.9**	**17**	**62**	**2.8**	**6**

Note 1: Population at risk, number of cases (N) and DALYs are presented in thousands.

Note 2: Total skin disease is defined as the sum of the number of cases with palpable nodules, reversible and irreversible skin disease.

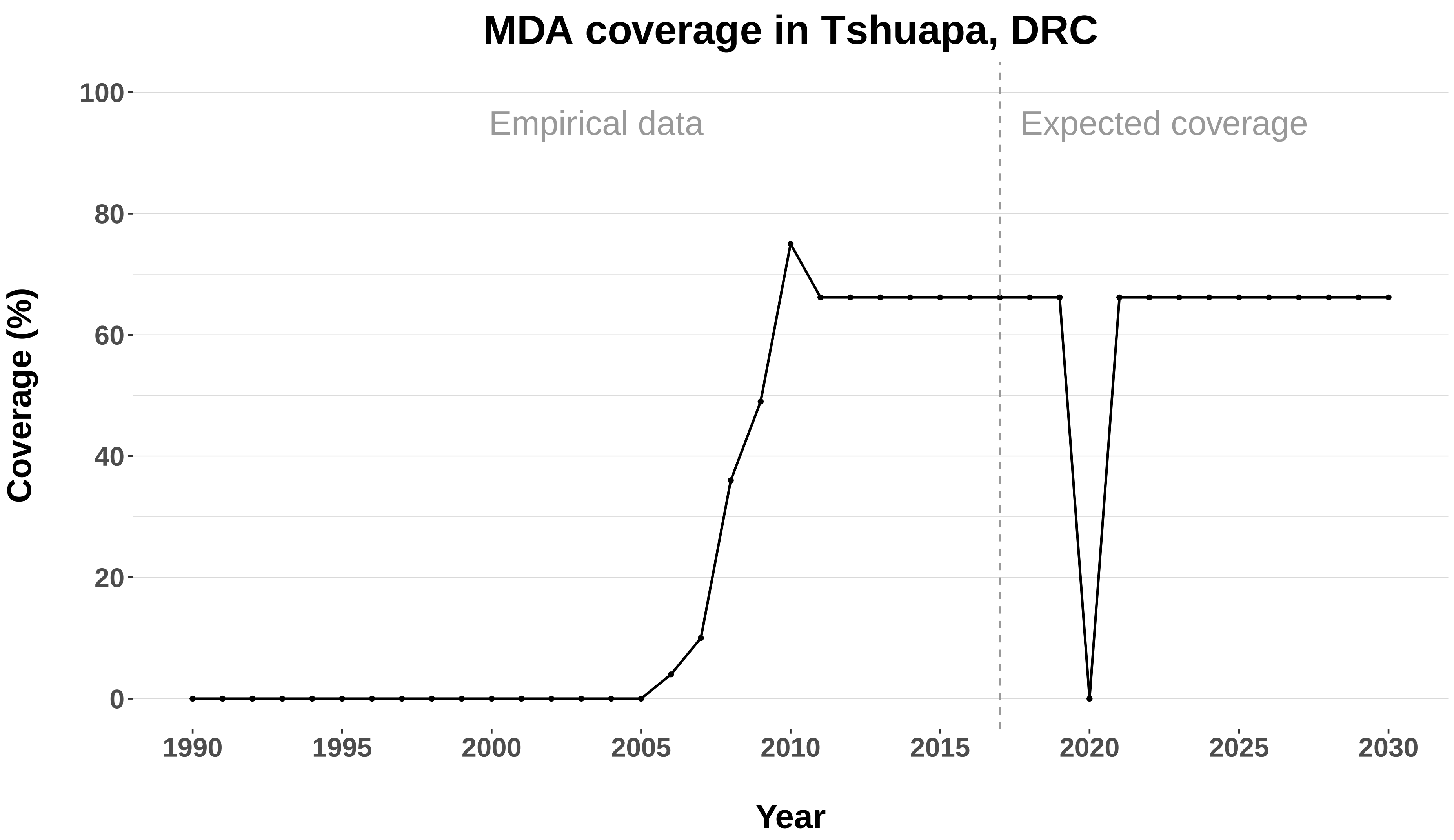
MDA coverage in Tshuapa, DRC
Empirical data
Expected coverage
Coverage (%)
100
80
60
40
20
0
1990
1995
2000
2005
2010
2015
2020
2025
2030
Year

**Project, country:**	**Ubangi-Nord, DRC**
Bioclime:	
Endemicity level(s):	Hyperendemic
MDA start year:	2006
Mean MDA coverage per year:	59.6

	1990			2020			2030		
	N	%	DALYs	N	%	DALYs	N	%	DALYs
Total populaton at risk	428	100	_	966	100	_	1,267	100	_
**Infection:**									
Mf-positive	207	48.3	_	157	16.2	_	56	4.4	_
Mf-positive (age 5+)	206	48.1	_	156	16.2	_	56	4.4	_
Worm infected	244	57.1	_	316	32.7	_	152	12.0	_
**Clinical manifestations:**									
Palpable nodules	94	21.9	1	71	7.4	1	19	1.5	<0.5
Reversible skin disease:									
Severe itch	42	9.9	8	16	1.6	3	2	0.2	<0.5
Reactive skin disease	35	8.2	2	3	0.4	<0.5	<0.5	<0.05	<0.5
**Reversible skin disease**	**78**	**18.1**	**9**	**19**	**2.0**	**3**	**3**	**0.2**	**<0.5**
Irreversible skin disease:									
Mild depigmentation	9	2.0	<0.5	12	1.2	<0.5	9	0.7	<0.5
Severe depigmentation	12	2.9	1	16	1.7	1	11	0.9	1
Atrophy	<0.5	0.1	<0.5	<0.5	<0.05	<0.5	<0.5	<0.05	<0.5
Hanging groin	1	0.2	<0.5	1	0.1	<0.5	<0.5	<0.05	<0.5
**Irreversible skin disease**	**22**	**5.1**	**1**	**28**	**2.9**	**1**	**21**	**1.6**	**1**
**Total skin disease (OSD)**	**193**	**45.2**	**12**	**119**	**12.3**	**5**	**43**	**3.4**	**2**
Eye disease (OED):									
Visual impairment	1	0.2	<0.5	1	0.1	<0.5	1	0.1	<0.5
Blindness	1	0.3	10	1	0.1	8	<0.5	<0.05	3
**Eye disease (OED)**	**2**	**0.5**	**10**	**2**	**0.2**	**8**	**1**	**0.1**	**3**
**Total all manifestations**	**196**	**45.7**	**21**	**121**	**12.5**	**13**	**44**	**3.5**	**5**

Note 1: Population at risk, number of cases (N) and DALYs are presented in thousands.

Note 2: Total skin disease is defined as the sum of the number of cases with palpable nodules, reversible and irreversible skin disease.

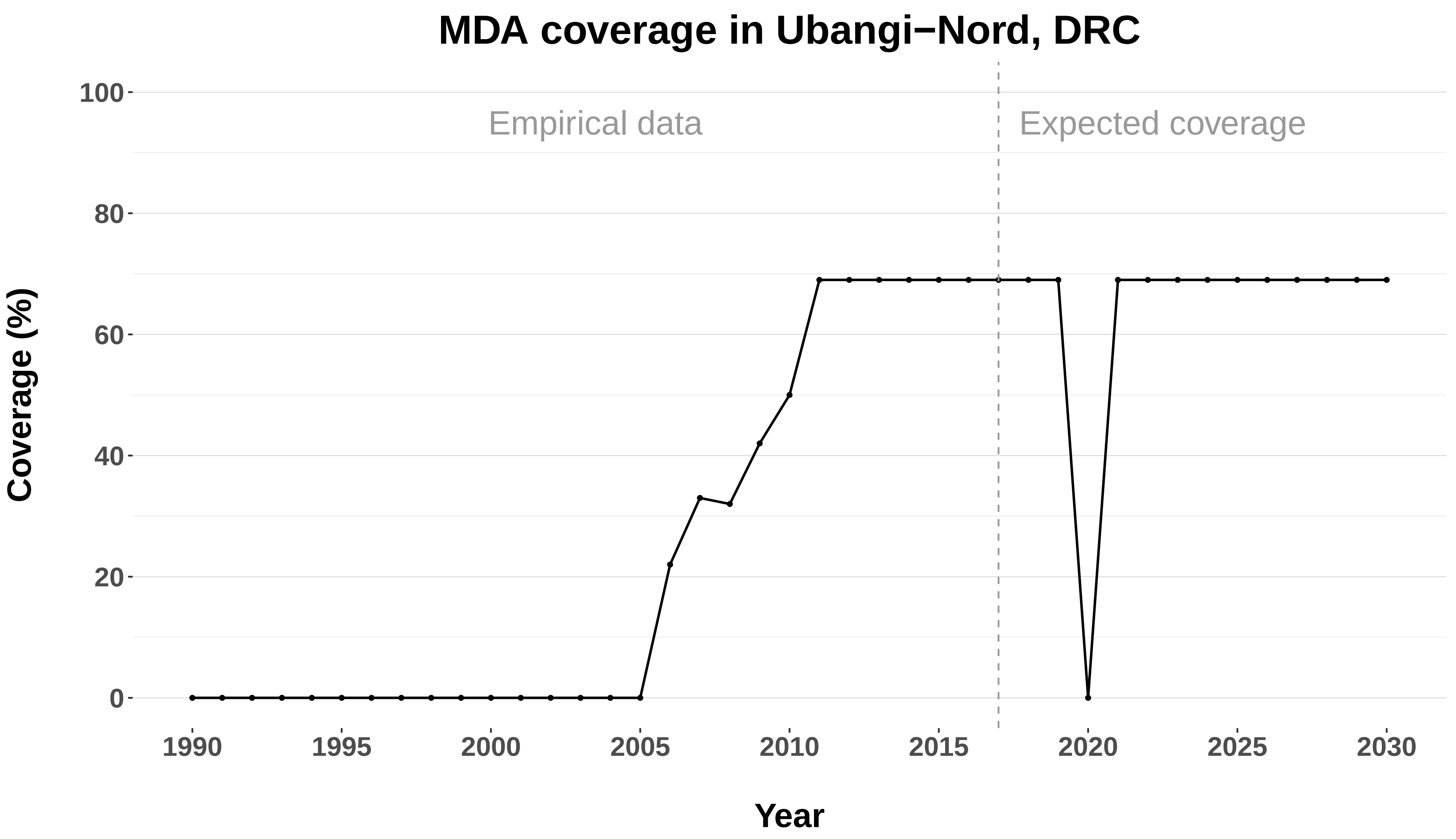
MDA coverage in Ubangi−Nord, DRC
Empirical data
Expected coverage
Coverage (%)
100
80
60
40
20
0
1990
1995
2000
2005
2010
2015
2020
2025
2030
Year

**Project, country:**	**Ubangi-Sud, DRC**
Bioclime:	
Endemicity level(s):	Hyperendemic
MDA start year:	2006
Mean MDA coverage per year:	60.8

	1990			2020			2030		
	N	%	DALYs	N	%	DALYs	N	%	DALYs
Total populaton at risk	722	100	_	1,629	100	_	2,136	100	_
**Infection:**									
Mf-positive	231	32.0	_	80	4.9	_	2	0.1	_
Mf-positive (age 5+)	231	32.0	_	80	4.9	_	2	0.1	_
Worm infected	297	41.2	_	289	17.7	_	36	1.7	_
**Clinical manifestations:**									
Palpable nodules	56	7.7	1	21	1.3	<0.5	1	<0.05	<0.5
Reversible skin disease:									
Severe itch	32	4.4	6	7	0.4	1	<0.5	<0.05	<0.5
Reactive skin disease	22	3.0	1	1	<0.05	<0.5	<0.5	<0.05	<0.5
**Reversible skin disease**	**54**	**7.5**	**7**	**7**	**0.4**	**1**	**<0.5**	**<0.05**	**<0.5**
Irreversible skin disease:									
Mild depigmentation	8	1.1	<0.5	10	0.6	<0.5	7	0.3	<0.5
Severe depigmentation	7	1.0	<0.5	9	0.6	1	6	0.3	<0.5
Atrophy	<0.5	<0.05	<0.5	<0.5	<0.05	<0.5	<0.5	<0.05	<0.5
Hanging groin	<0.5	<0.05	<0.5	<0.5	<0.05	<0.5	<0.5	<0.05	<0.5
**Irreversible skin disease**	**15**	**2.1**	**1**	**19**	**1.2**	**1**	**13**	**0.6**	**<0.5**
**Total skin disease (OSD)**	**125**	**17.3**	**8**	**47**	**2.9**	**2**	**14**	**0.7**	**1**
Eye disease (OED):									
Visual impairment	<0.5	<0.05	<0.5	<0.5	<0.05	<0.5	<0.5	<0.05	<0.5
Blindness	<0.5	<0.05	1	<0.5	<0.05	1	<0.5	<0.05	<0.5
**Eye disease (OED)**	**<0.5**	**<0.05**	**1**	**<0.5**	**<0.05**	**1**	**<0.5**	**<0.05**	**<0.5**
**Total all manifestations**	**125**	**17.3**	**9**	**47**	**2.9**	**3**	**14**	**0.7**	**1**

Note 1: Population at risk, number of cases (N) and DALYs are presented in thousands.

Note 2: Total skin disease is defined as the sum of the number of cases with palpable nodules, reversible and irreversible skin disease.

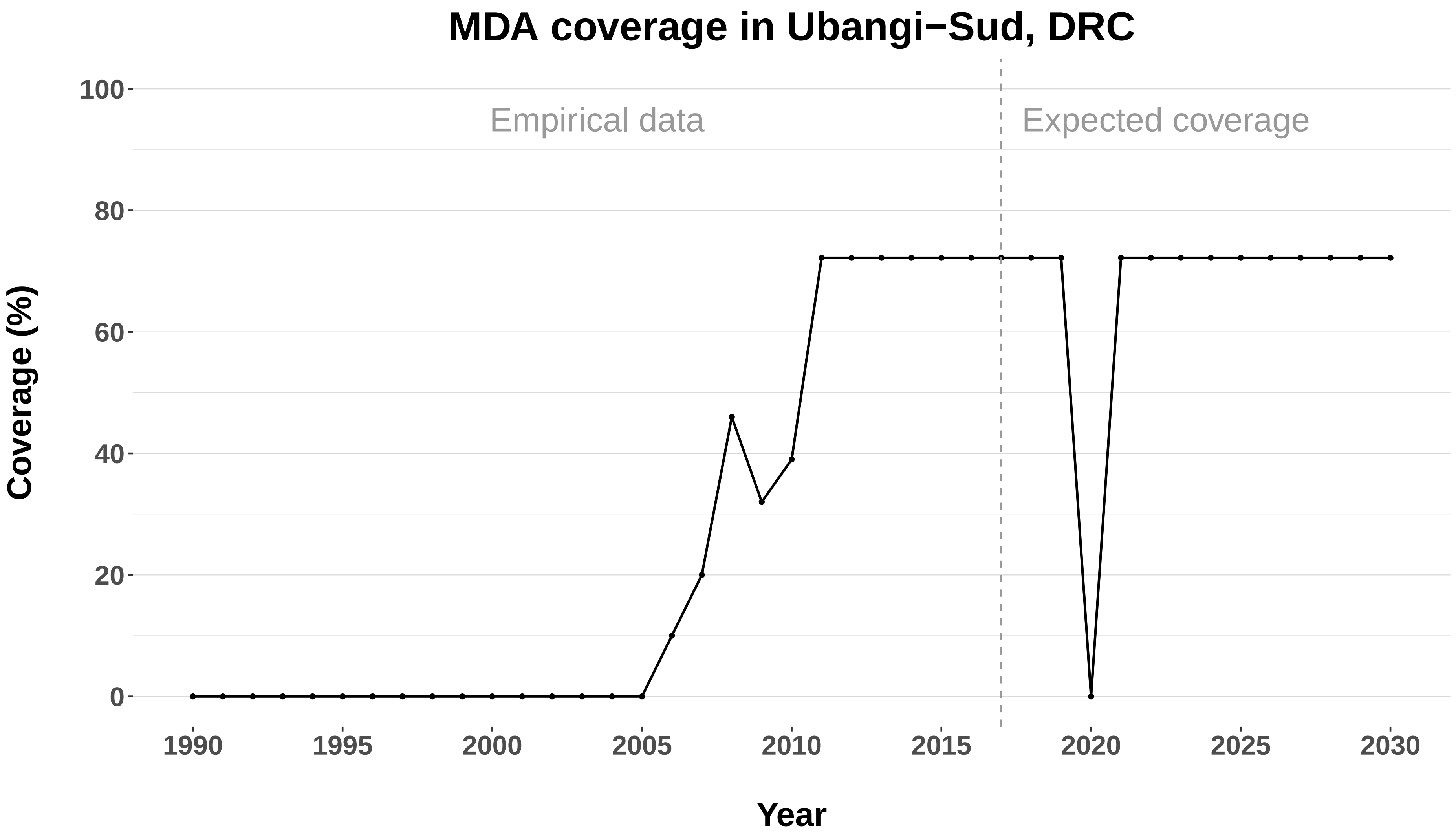
MDA coverage in Ubangi−Sud, DRC
Empirical data
Expected coverage
Coverage (%)
0
20
40
60
80
100
1990
1995
2000
2005
2010
2015
2020
2025
2030
Year

**Project, country:**	**Ueles, DRC**
Bioclime:	
Endemicity level(s):	Hyperendemic
MDA start year:	2002
Mean MDA coverage per year:	65.5

	1990			2020			2030		
	N	%	DALYs	N	%	DALYs	N	%	DALYs
Total populaton at risk	842	100	_	1,900	100	_	2,492	100	_
**Infection:**									
Mf-positive	512	60.8	_	268	14.1	_	76	3.0	_
Mf-positive (age 5+)	509	60.5	_	268	14.1	_	76	3.0	_
Worm infected	574	68.2	_	607	32.0	_	282	11.3	_
**Clinical manifestations:**									
Palpable nodules	288	34.3	3	122	6.4	1	24	1.0	<0.5
Reversible skin disease:									
Severe itch	127	15.1	23	20	1.1	4	2	0.1	<0.5
Reactive skin disease	112	13.3	5	2	0.1	<0.5	<0.5	<0.05	<0.5
**Reversible skin disease**	**239**	**28.3**	**28**	**23**	**1.2**	**4**	**2**	**0.1**	**<0.5**
Irreversible skin disease:									
Mild depigmentation	24	2.8	<0.5	27	1.4	<0.5	20	0.8	<0.5
Severe depigmentation	41	4.9	3	42	2.2	3	29	1.2	2
Atrophy	1	0.2	<0.5	<0.5	<0.05	<0.5	<0.5	<0.05	<0.5
Hanging groin	3	0.4	1	2	0.1	1	1	0.1	1
**Irreversible skin disease**	**69**	**8.2**	**4**	**72**	**3.8**	**4**	**51**	**2.1**	**3**
**Total skin disease (OSD)**	**596**	**70.9**	**36**	**216**	**11.4**	**9**	**78**	**3.1**	**3**
Eye disease (OED):									
Visual impairment	4	0.5	<0.5	3	0.2	<0.5	2	0.1	<0.5
Blindness	6	0.7	44	3	0.2	22	1	<0.05	9
**Eye disease (OED)**	**10**	**1.2**	**44**	**6**	**0.3**	**22**	**3**	**0.1**	**9**
**Total all manifestations**	**606**	**72.0**	**79**	**222**	**11.7**	**31**	**81**	**3.2**	**12**

Note 1: Population at risk, number of cases (N) and DALYs are presented in thousands.

Note 2: Total skin disease is defined as the sum of the number of cases with palpable nodules, reversible and irreversible skin disease.

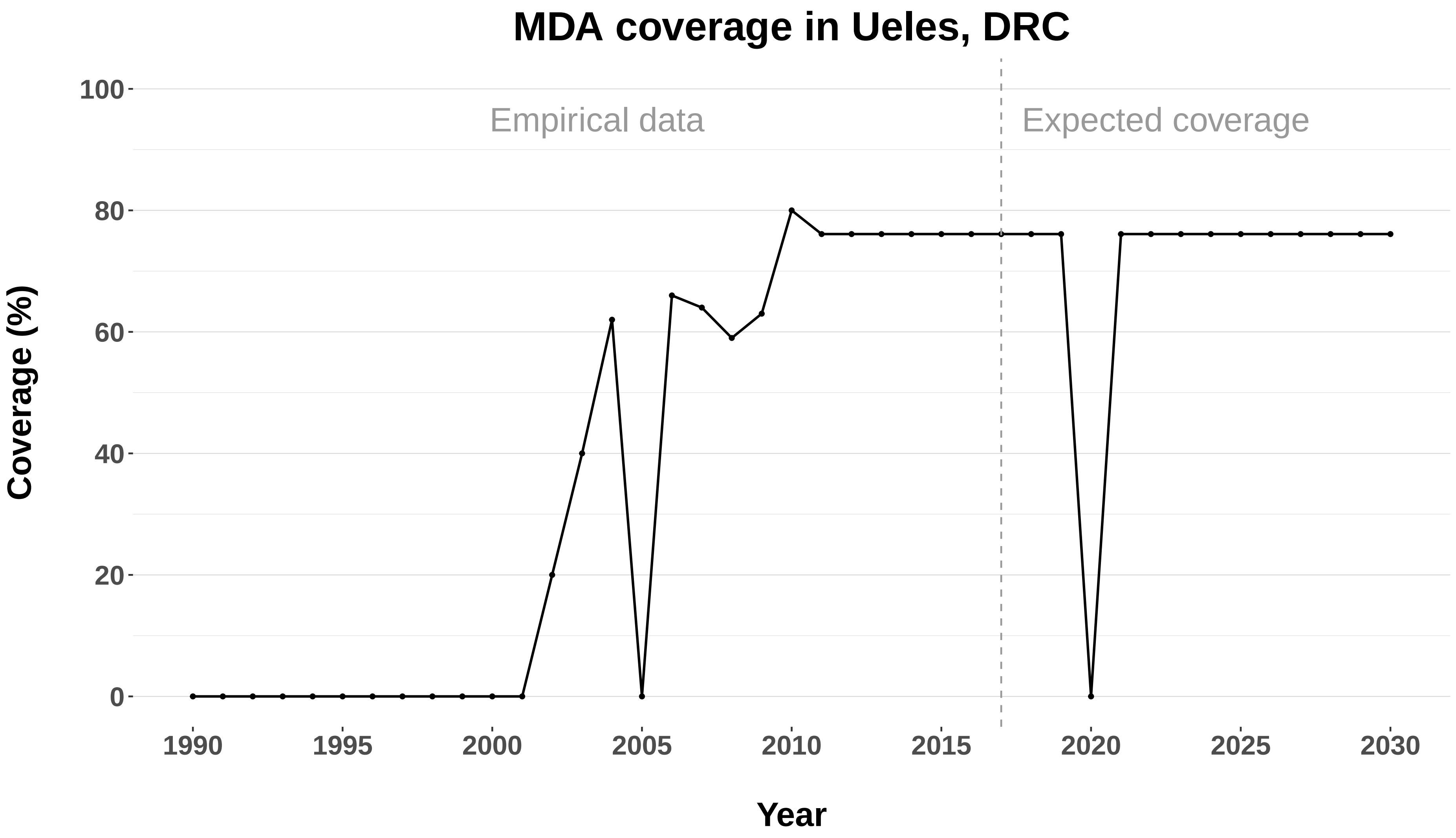

MDA coverage in Ueles, DRC
Empirical data
Expected coverage
Coverage (%)
100
80
60
40
20
0
1990
1995
2000
2005
2010
2015
2020
2025
2030
Year

**Project, country:**	**Bioko, Equatorial Guinea**
Bioclime:	
Endemicity level(s):	Hyperendemic
MDA start year:	1990
Mean MDA coverage per year:	49.2

	1990			2020			2030		
	N	%	DALYs	N	%	DALYs	N	%	DALYs
Total populaton at risk	59	100	_	134	100	_	176	100	_
**Infection:**									
Mf-positive	44	74.8	_	<0.5	<0.05	_	<0.5	<0.05	_
Mf-positive (age 5+)	44	73.9	_	<0.5	<0.05	_	<0.5	<0.05	_
Worm infected	47	79.8	_	<0.5	0.2	_	<0.5	<0.05	_
**Clinical manifestations:**									
Palpable nodules	32	53.4	<0.5	<0.5	<0.05	<0.5	<0.5	<0.05	<0.5
Reversible skin disease:									
Severe itch	15	24.5	3	<0.5	<0.05	<0.5	<0.5	<0.05	<0.5
Reactive skin disease	14	23.4	1	<0.5	<0.05	<0.5	<0.5	<0.05	<0.5
**Reversible skin disease**	**28**	**47.8**	**3**	**<0.5**	**<0.05**	**<0.5**	**<0.5**	**<0.05**	**<0.5**
Irreversible skin disease:									
Mild depigmentation	2	3.9	<0.5	1	1.0	<0.5	1	0.5	<0.5
Severe depigmentation	5	9.2	<0.5	2	1.8	<0.5	1	0.8	<0.5
Atrophy	<0.5	0.7	<0.5	<0.5	<0.05	<0.5	<0.5	<0.05	<0.5
Hanging groin	1	1.5	<0.5	<0.5	0.2	<0.5	<0.5	0.1	<0.5
**Irreversible skin disease**	**9**	**15.3**	**1**	**4**	**2.9**	**<0.5**	**2**	**1.4**	**<0.5**
**Total skin disease (OSD)**	**69**	**116.5**	**4**	**4**	**2.9**	**<0.5**	**2**	**1.4**	**<0.5**
Eye disease (OED):									
Visual impairment	1	1.1	<0.5	<0.5	0.2	<0.5	<0.5	0.1	<0.5
Blindness	1	2.4	11	<0.5	0.1	1	<0.5	<0.05	<0.5
**Eye disease (OED)**	**2**	**3.6**	**11**	**<0.5**	**0.3**	**1**	**<0.5**	**0.1**	**<0.5**
**Total all manifestations**	**71**	**120.1**	**15**	**4**	**3.2**	**1**	**3**	**1.4**	**<0.5**

Note 1: Population at risk, number of cases (N) and DALYs are presented in thousands.

Note 2: Total skin disease is defined as the sum of the number of cases with palpable nodules, reversible and irreversible skin disease.

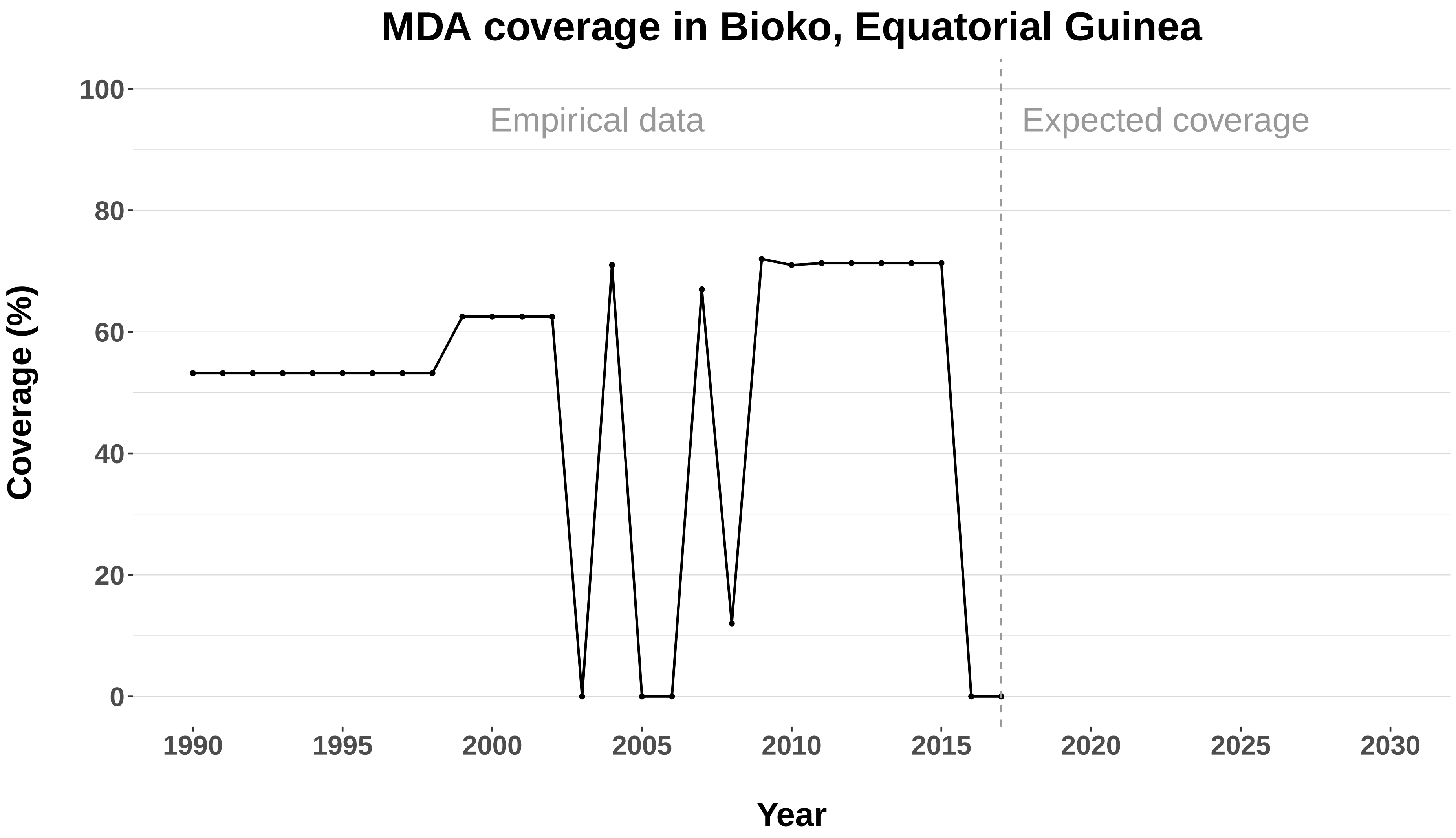

Note: We assume no ivermectic MDA for Bioko since 2018 as the island is currently under post-treatment surveillance [1-3][‡, page 354].

**Project, country:**	**P5EqGuinea, Equatorial Guinea**
Bioclime:	
Endemicity level(s):	Hypoendemic
MDA start year:	2025
Mean MDA coverage per year:	65.0

	1990			2020			2030		
	N	%	DALYs	N	%	DALYs	N	%	DALYs
Total populaton at risk	139	100	_	314	100	_	412	100	_
**Infection:**									
Mf-positive	31	22.6	_	<0.5	<0.05	_	<0.5	<0.05	_
Mf-positive (age 5+)	31	22.6	_	<0.5	<0.05	_	<0.5	<0.05	_
Worm infected	41	29.8	_	<0.5	0.1	_	<0.5	<0.05	_
**Clinical manifestations:**									
Palpable nodules	3	2.4	<0.5	<0.5	<0.05	<0.5	<0.5	<0.05	<0.5
Reversible skin disease:									
Severe itch	4	2.9	1	<0.5	<0.05	<0.5	<0.5	<0.05	<0.5
Reactive skin disease	3	1.9	<0.5	<0.5	<0.05	<0.5	<0.5	<0.05	<0.5
**Reversible skin disease**	**7**	**4.8**	**1**	**<0.5**	**<0.05**	**<0.5**	**<0.5**	**<0.05**	**<0.5**
Irreversible skin disease:									
Mild depigmentation	1	0.8	<0.5	1	0.2	<0.5	<0.5	0.1	<0.5
Severe depigmentation	1	0.7	<0.5	1	0.2	<0.5	<0.5	0.1	<0.5
Atrophy	<0.5	<0.05	<0.5	<0.5	<0.05	<0.5	<0.5	<0.05	<0.5
Hanging groin	<0.5	<0.05	<0.5	<0.5	<0.05	<0.5	<0.5	<0.05	<0.5
**Irreversible skin disease**	**2**	**1.5**	**<0.5**	**1**	**0.4**	**<0.5**	**1**	**0.2**	**<0.5**
**Total skin disease (OSD)**	**12**	**8.7**	**1**	**1**	**0.4**	**<0.5**	**1**	**0.2**	**<0.5**
Eye disease (OED):									
Visual impairment	<0.5	<0.05	<0.5	<0.5	<0.05	<0.5	<0.5	<0.05	<0.5
Blindness	<0.5	<0.05	<0.5	<0.5	<0.05	<0.5	<0.5	<0.05	<0.5
**Eye disease (OED)**	**<0.5**	**<0.05**	**<0.5**	**<0.5**	**<0.05**	**<0.5**	**<0.5**	**<0.05**	**<0.5**
**Total all manifestations**	**12**	**8.7**	**1**	**1**	**0.4**	**<0.5**	**1**	**0.2**	**<0.5**

Note 1: Population at risk, number of cases (N) and DALYs are presented in thousands.

Note 2: Total skin disease is defined as the sum of the number of cases with palpable nodules, reversible and irreversible skin disease.

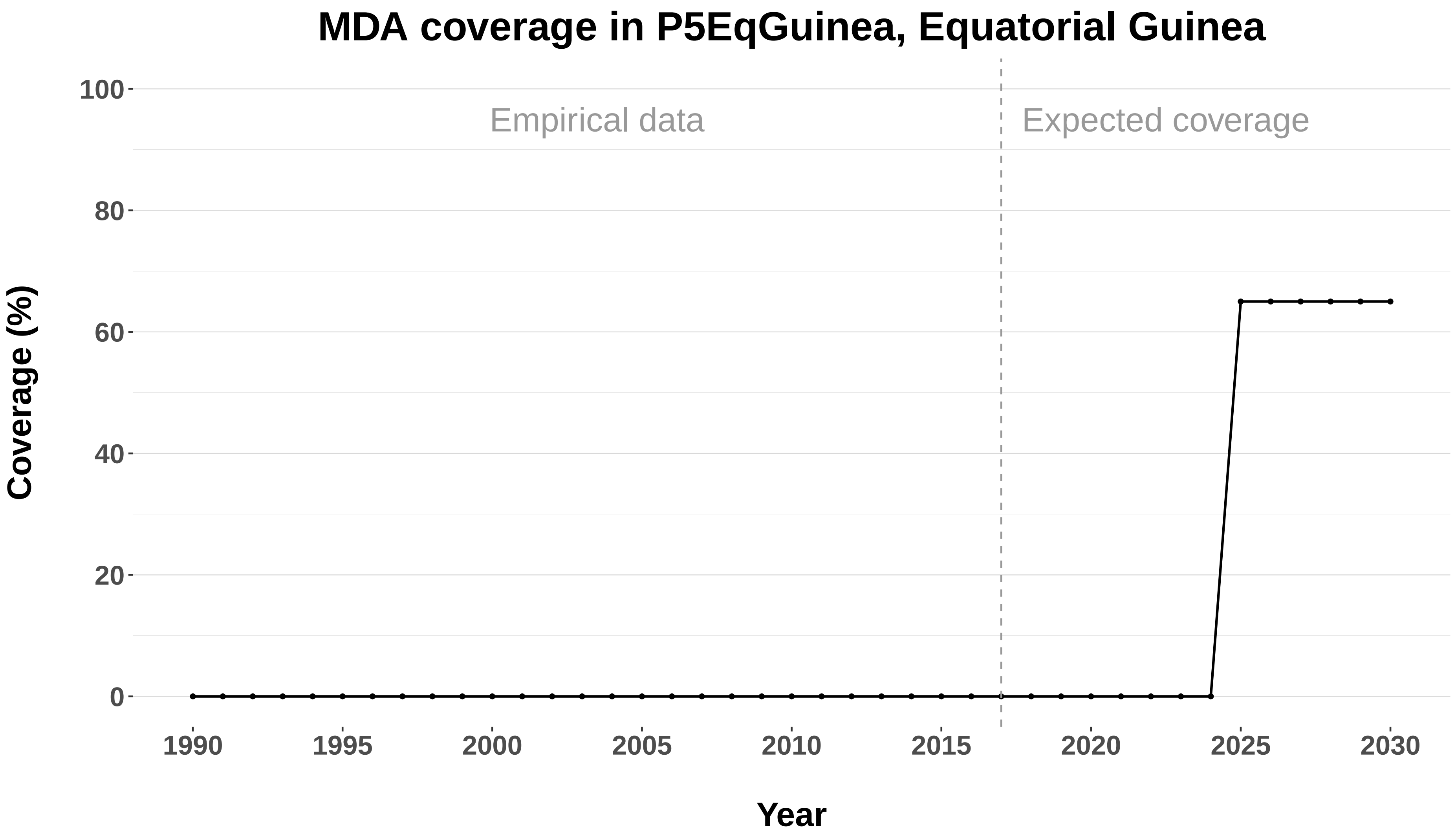
MDA coverage in P5EqGuinea, Equatorial Guinea
Empirical data
Expected coverage
Coverage (%)
100
80
60
40
20
0
1990
1995
2000
2005
2010
2015
2020
2025
2030
Year

**Project, country:**	**Assosa, Ethiopia**
Bioclime:	
Endemicity level(s):	Mesoendemic
MDA start year:	2019
Mean MDA coverage per year:	72.4

	1990			2020			2030		
	N	%	DALYs	N	%	DALYs	N	%	DALYs
Total populaton at risk	317	100	_	716	100	_	939	100	_
**Infection:**									
Mf-positive	84	26.5	_	123	17.2	_	1	0.1	_
Mf-positive (age 5+)	84	26.5	_	123	17.2	_	1	0.1	_
Worm infected	111	34.8	_	244	34.1	_	81	8.6	_
**Clinical manifestations:**									
Palpable nodules	15	4.7	<0.5	32	4.5	<0.5	3	0.3	<0.5
Reversible skin disease:									
Severe itch	11	3.4	2	20	2.7	4	1	0.1	<0.5
Reactive skin disease	7	2.3	<0.5	10	1.5	<0.5	<0.5	<0.05	<0.5
**Reversible skin disease**	**18**	**5.7**	**2**	**30**	**4.2**	**4**	**1**	**0.1**	**<0.5**
Irreversible skin disease:									
Mild depigmentation	3	0.9	<0.5	6	0.8	<0.5	5	0.5	<0.5
Severe depigmentation	2	0.7	<0.5	5	0.7	<0.5	4	0.4	<0.5
Atrophy	<0.5	<0.05	<0.5	<0.5	<0.05	<0.5	<0.5	<0.05	<0.5
Hanging groin	<0.5	<0.05	<0.5	<0.5	<0.05	<0.5	<0.5	<0.05	<0.5
**Irreversible skin disease**	**5**	**1.6**	**<0.5**	**11**	**1.5**	**<0.5**	**8**	**0.9**	**<0.5**
**Total skin disease (OSD)**	**38**	**12.0**	**3**	**73**	**10.3**	**5**	**13**	**1.3**	**1**
Eye disease (OED):									
Visual impairment	<0.5	<0.05	<0.5	<0.5	<0.05	<0.5	<0.5	<0.05	<0.5
Blindness	<0.5	<0.05	<0.5	<0.5	<0.05	<0.5	<0.5	<0.05	<0.5
**Eye disease (OED)**	**<0.5**	**<0.05**	**<0.5**	**<0.5**	**<0.05**	**<0.5**	**<0.5**	**<0.05**	**<0.5**
**Total all manifestations**	**38**	**12.1**	**3**	**74**	**10.3**	**5**	**13**	**1.3**	**1**

Note 1: Population at risk, number of cases (N) and DALYs are presented in thousands.

Note 2: Total skin disease is defined as the sum of the number of cases with palpable nodules, reversible and irreversible skin disease.

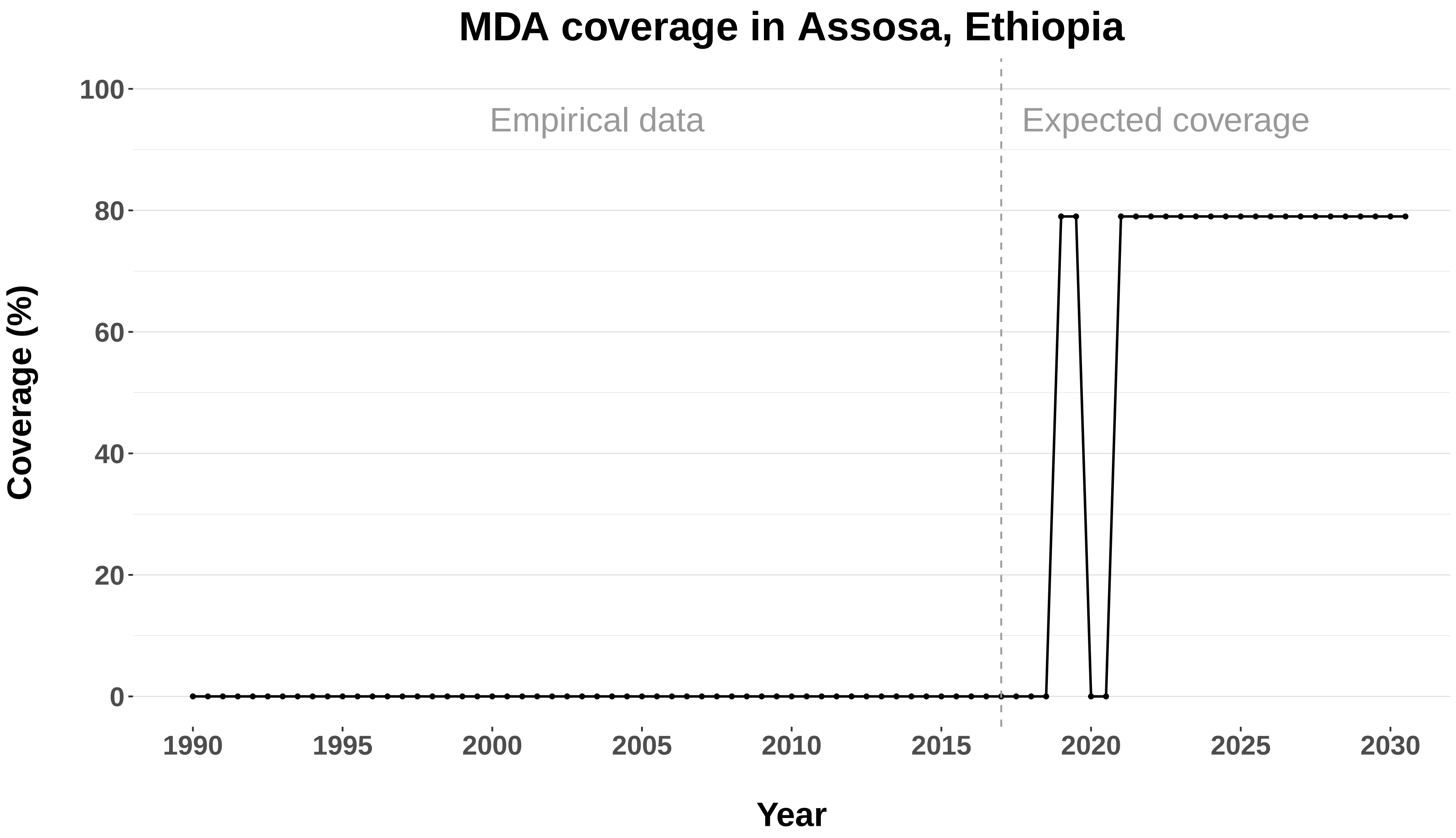
MDA coverage in Assosa, Ethiopia
Empirical data
Expected coverage
Coverage (%)
100
80
60
40
20
0
1990
1995
2000
2005
2010
2015
2020
2025
2030
Year

**Project, country:**	**Bench-Maji, Ethiopia**
Bioclime:	
Endemicity level(s):	Hyperendemic
MDA start year:	2003
Mean MDA coverage per year:	70.1

	1990			2020			2030		
	N	%	DALYs	N	%	DALYs	N	%	DALYs
Total populaton at risk	420	100	_	947	100	_	1,242	100	_
**Infection:**									
Mf-positive	162	38.7	_	6	0.6	_	<0.5	<0.05	_
Mf-positive (age 5+)	162	38.6	_	6	0.6	_	<0.5	<0.05	_
Worm infected	206	49.0	_	66	6.9	_	2	0.2	_
**Clinical manifestations:**									
Palpable nodules	49	11.6	1	2	0.2	<0.5	<0.5	<0.05	<0.5
Reversible skin disease:									
Severe itch	23	5.5	4	1	0.1	<0.5	<0.5	<0.05	<0.5
Reactive skin disease	16	3.9	1	<0.5	<0.05	<0.5	<0.5	<0.05	<0.5
**Reversible skin disease**	**40**	**9.5**	**5**	**1**	**0.1**	**<0.5**	**<0.5**	**<0.05**	**<0.5**
Irreversible skin disease:									
Mild depigmentation	5	1.3	<0.5	5	0.6	<0.5	4	0.3	<0.5
Severe depigmentation	5	1.3	<0.5	5	0.5	<0.5	3	0.2	<0.5
Atrophy	<0.5	<0.05	<0.5	<0.5	<0.05	<0.5	<0.5	<0.05	<0.5
Hanging groin	<0.5	<0.05	<0.5	<0.5	<0.05	<0.5	<0.5	<0.05	<0.5
**Irreversible skin disease**	**11**	**2.6**	**<0.5**	**10**	**1.1**	**<0.5**	**7**	**0.5**	**<0.5**
**Total skin disease (OSD)**	**99**	**23.7**	**6**	**13**	**1.4**	**1**	**7**	**0.5**	**<0.5**
Eye disease (OED):									
Visual impairment	<0.5	<0.05	<0.5	<0.5	<0.05	<0.5	<0.5	<0.05	<0.5
Blindness	<0.5	<0.05	1	<0.5	<0.05	<0.5	<0.5	<0.05	<0.5
**Eye disease (OED)**	**<0.5**	**0.1**	**1**	**<0.5**	**<0.05**	**<0.5**	**<0.5**	**<0.05**	**<0.5**
**Total all manifestations**	**100**	**23.7**	**7**	**13**	**1.4**	**1**	**7**	**0.5**	**<0.5**

Note 1: Population at risk, number of cases (N) and DALYs are presented in thousands.

Note 2: Total skin disease is defined as the sum of the number of cases with palpable nodules, reversible and irreversible skin disease.

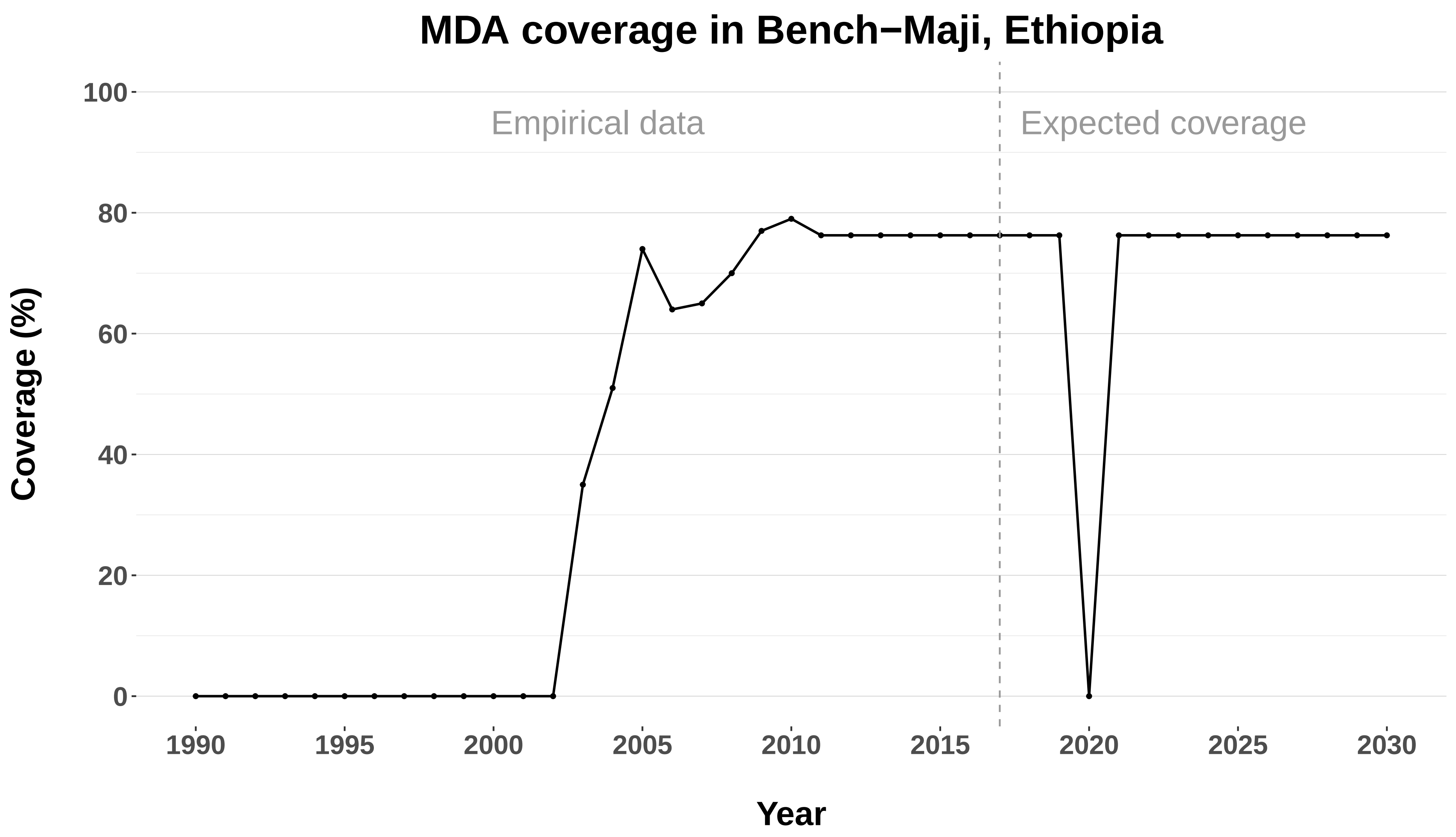
MDA coverage in Bench−Maji, Ethiopia
Empirical data
Expected coverage
Coverage (%)
100
80
60
40
20
0
1990
1995
2000
2005
2010
2015
2020
2025
2030
Year

**Project, country:**	**East Wellega, Ethiopia**
Bioclime:	
Endemicity level(s):	Mesoendemic
MDA start year:	2004
Mean MDA coverage per year:	72.5

	1990			2020			2030		
	N	%	DALYs	N	%	DALYs	N	%	DALYs
Total populaton at risk	514	100	_	1,160	100	_	1,521	100	_
**Infection:**									
Mf-positive	175	34.1	_	9	0.8	_	<0.5	<0.05	_
Mf-positive (age 5+)	175	34.0	_	9	0.8	_	<0.5	<0.05	_
Worm infected	224	43.5	_	91	7.8	_	3	0.2	_
**Clinical manifestations:**									
Palpable nodules	46	9.0	<0.5	4	0.3	<0.5	<0.5	<0.05	<0.5
Reversible skin disease:									
Severe itch	25	4.8	4	1	0.1	<0.5	<0.5	<0.05	<0.5
Reactive skin disease	18	3.4	1	<0.5	<0.05	<0.5	<0.5	<0.05	<0.5
**Reversible skin disease**	**42**	**8.2**	**5**	**1**	**0.1**	**<0.5**	**<0.5**	**<0.05**	**<0.5**
Irreversible skin disease:									
Mild depigmentation	6	1.1	<0.5	6	0.5	<0.5	4	0.3	<0.5
Severe depigmentation	6	1.1	<0.5	6	0.5	<0.5	4	0.2	<0.5
Atrophy	<0.5	<0.05	<0.5	<0.5	<0.05	<0.5	<0.5	<0.05	<0.5
Hanging groin	<0.5	<0.05	<0.5	<0.5	<0.05	<0.5	<0.5	<0.05	<0.5
**Irreversible skin disease**	**12**	**2.3**	**<0.5**	**12**	**1.0**	**<0.5**	**8**	**0.5**	**<0.5**
**Total skin disease (OSD)**	**100**	**19.5**	**6**	**17**	**1.4**	**1**	**8**	**0.5**	**<0.5**
Eye disease (OED):									
Visual impairment	<0.5	<0.05	<0.5	<0.5	<0.05	<0.5	<0.5	<0.05	<0.5
Blindness	<0.5	<0.05	1	<0.5	<0.05	<0.5	<0.5	<0.05	<0.5
**Eye disease (OED)**	**<0.5**	**0.1**	**1**	**<0.5**	**<0.05**	**<0.5**	**<0.5**	**<0.05**	**<0.5**
**Total all manifestations**	**101**	**19.6**	**7**	**17**	**1.5**	**1**	**8**	**0.5**	**<0.5**

Note 1: Population at risk, number of cases (N) and DALYs are presented in thousands.

Note 2: Total skin disease is defined as the sum of the number of cases with palpable nodules, reversible and irreversible skin disease.

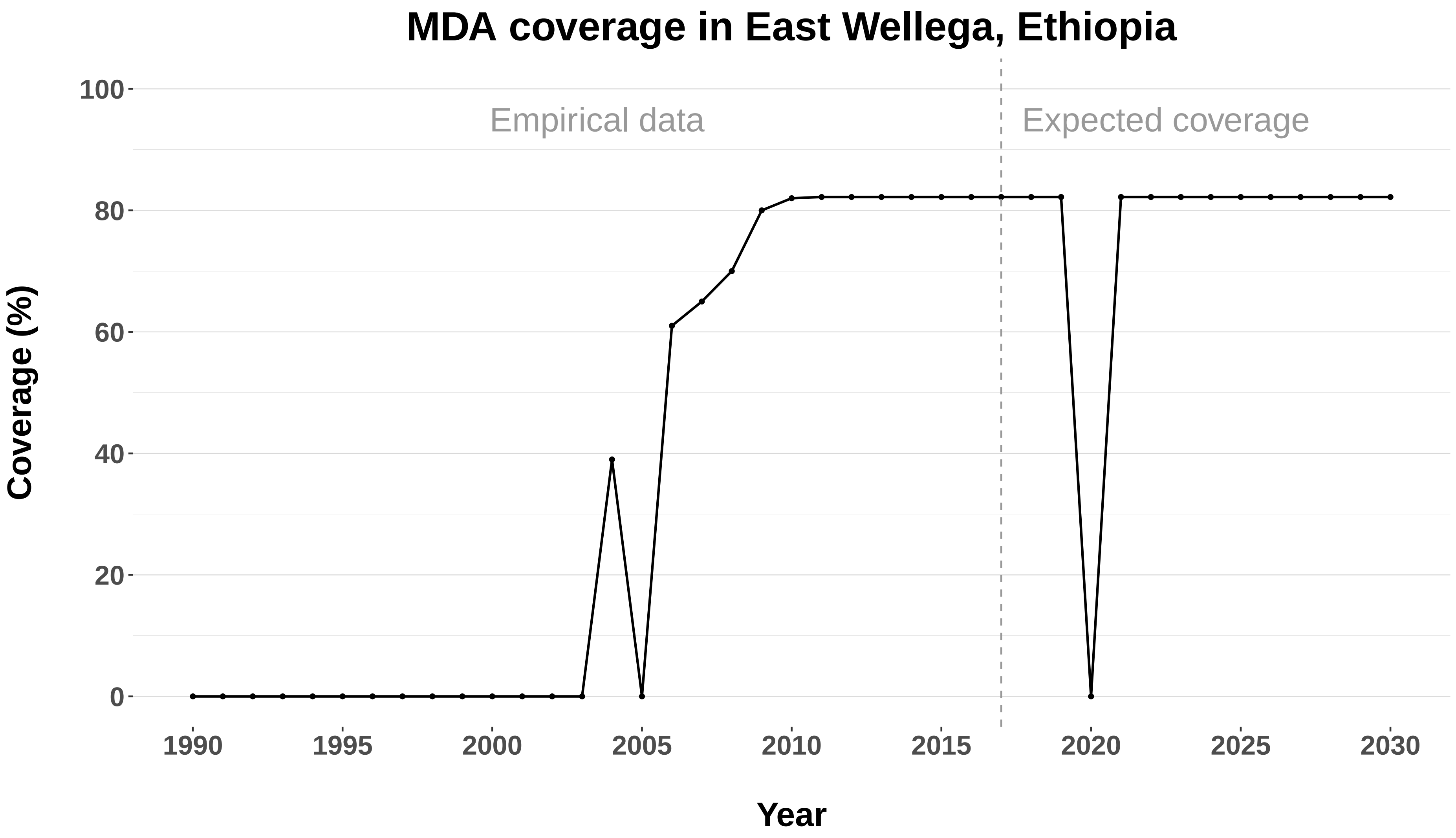

MDA coverage in East Wellega, Ethiopia
Empirical data
Expected coverage
Coverage (%)
100
80
60
40
20
0
1990
1995
2000
2005
2010
2015
2020
2025
2030
Year

**Project, country:**	**Gambella, Ethiopia**
Bioclime:	
Endemicity level(s):	Hyperendemic
MDA start year:	2004
Mean MDA coverage per year:	70.8

	1990			2020			2030		
	N	%	DALYs	N	%	DALYs	N	%	DALYs
Total populaton at risk	61	100	_	139	100	_	182	100	_
**Infection:**									
Mf-positive	22	36.4	_	1	0.9	_	<0.5	<0.05	_
Mf-positive (age 5+)	22	36.4	_	1	0.9	_	<0.5	<0.05	_
Worm infected	28	45.7	_	11	7.9	_	<0.5	0.2	_
**Clinical manifestations:**									
Palpable nodules	6	10.3	<0.5	<0.5	0.3	<0.5	<0.5	<0.05	<0.5
Reversible skin disease:									
Severe itch	3	5.3	1	<0.5	0.1	<0.5	<0.5	<0.05	<0.5
Reactive skin disease	2	3.8	<0.5	<0.5	<0.05	<0.5	<0.5	<0.05	<0.5
**Reversible skin disease**	**6**	**9.1**	**1**	**<0.5**	**0.1**	**<0.5**	**<0.5**	**<0.05**	**<0.5**
Irreversible skin disease:									
Mild depigmentation	1	1.3	<0.5	1	0.6	<0.5	1	0.3	<0.5
Severe depigmentation	1	1.2	<0.5	1	0.5	<0.5	<0.5	0.2	<0.5
Atrophy	<0.5	<0.05	<0.5	<0.5	<0.05	<0.5	<0.5	<0.05	<0.5
Hanging groin	<0.5	<0.05	<0.5	<0.5	<0.05	<0.5	<0.5	<0.05	<0.5
**Irreversible skin disease**	**2**	**2.5**	**<0.5**	**2**	**1.1**	**<0.5**	**1**	**0.5**	**<0.5**
**Total skin disease (OSD)**	**13**	**21.9**	**1**	**2**	**1.5**	**<0.5**	**1**	**0.5**	**<0.5**
Eye disease (OED):									
Visual impairment	<0.5	<0.05	<0.5	<0.5	<0.05	<0.5	<0.5	<0.05	<0.5
Blindness	<0.5	<0.05	<0.5	<0.5	<0.05	<0.5	<0.5	<0.05	<0.5
**Eye disease (OED)**	**<0.5**	**0.1**	**<0.5**	**<0.5**	**<0.05**	**<0.5**	**<0.5**	**<0.05**	**<0.5**
**Total all manifestations**	**13**	**22.0**	**1**	**2**	**1.5**	**<0.5**	**1**	**0.5**	**<0.5**

Note 1: Population at risk, number of cases (N) and DALYs are presented in thousands.

Note 2: Total skin disease is defined as the sum of the number of cases with palpable nodules, reversible and irreversible skin disease.

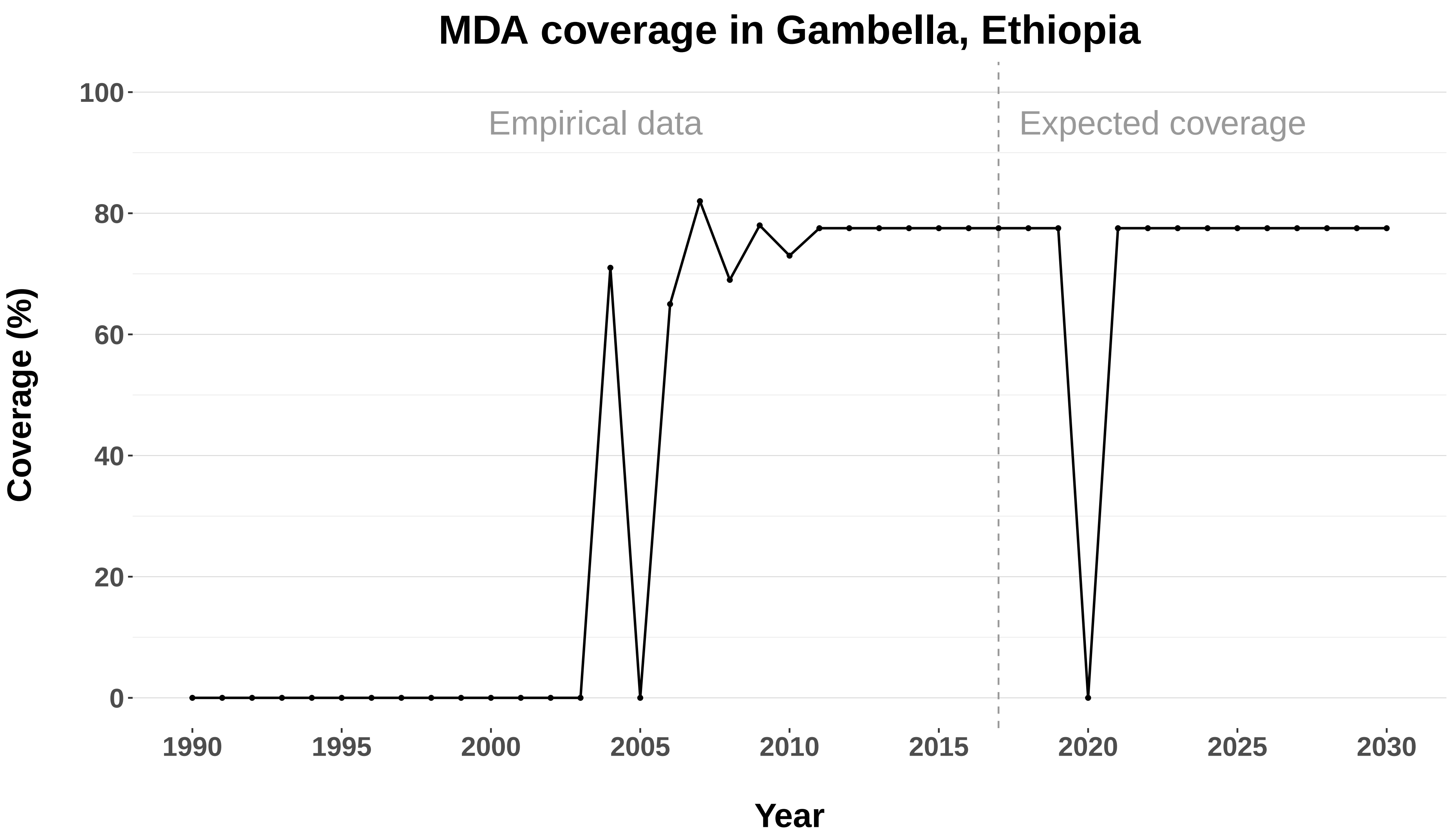
MDA coverage in Gambella, Ethiopia
Empirical data
Expected coverage
Coverage (%)
100
80
60
40
20
0
1990
1995
2000
2005
2010
2015
2020
2025
2030
Year

**Project, country:**	**Horo Guduru, Ethiopia**
Bioclime:	
Endemicity level(s):	Mesoendemic
MDA start year:	2019
Mean MDA coverage per year:	72.4

	1990			2020			2030		
	N	%	DALYs	N	%	DALYs	N	%	DALYs
Total populaton at risk	30	100	_	68	100	_	89	100	_
**Infection:**									
Mf-positive	10	34.7	_	22	32.0	_	4	4.4	_
Mf-positive (age 5+)	10	34.6	_	22	32.0	_	4	4.4	_
Worm infected	13	44.8	_	30	44.3	_	17	19.0	_
**Clinical manifestations:**									
Palpable nodules	3	9.0	<0.5	6	8.7	<0.5	1	1.4	<0.5
Reversible skin disease:									
Severe itch	1	4.6	<0.5	3	3.9	<0.5	<0.5	0.3	<0.5
Reactive skin disease	1	3.2	<0.5	1	2.1	<0.5	<0.5	<0.05	<0.5
**Reversible skin disease**	**2**	**7.8**	**<0.5**	**4**	**6.0**	**1**	**<0.5**	**0.3**	**<0.5**
Irreversible skin disease:									
Mild depigmentation	<0.5	1.1	<0.5	1	1.0	<0.5	1	0.7	<0.5
Severe depigmentation	<0.5	1.0	<0.5	1	0.9	<0.5	<0.5	0.5	<0.5
Atrophy	<0.5	<0.05	<0.5	<0.5	<0.05	<0.5	<0.5	<0.05	<0.5
Hanging groin	<0.5	<0.05	<0.5	<0.5	<0.05	<0.5	<0.5	<0.05	<0.5
**Irreversible skin disease**	**1**	**2.1**	**<0.5**	**1**	**2.0**	**<0.5**	**1**	**1.2**	**<0.5**
**Total skin disease (OSD)**	**6**	**19.0**	**<0.5**	**11**	**16.7**	**1**	**3**	**3.0**	**<0.5**
Eye disease (OED):									
Visual impairment	<0.5	<0.05	<0.5	<0.5	<0.05	<0.5	<0.5	<0.05	<0.5
Blindness	<0.5	<0.05	<0.5	<0.5	<0.05	<0.5	<0.5	<0.05	<0.5
**Eye disease (OED)**	**<0.5**	**<0.05**	**<0.5**	**<0.5**	**<0.05**	**<0.5**	**<0.5**	**<0.05**	**<0.5**
**Total all manifestations**	**6**	**19.0**	**<0.5**	**11**	**16.8**	**1**	**3**	**3.0**	**<0.5**

Note 1: Population at risk, number of cases (N) and DALYs are presented in thousands.

Note 2: Total skin disease is defined as the sum of the number of cases with palpable nodules, reversible and irreversible skin disease.

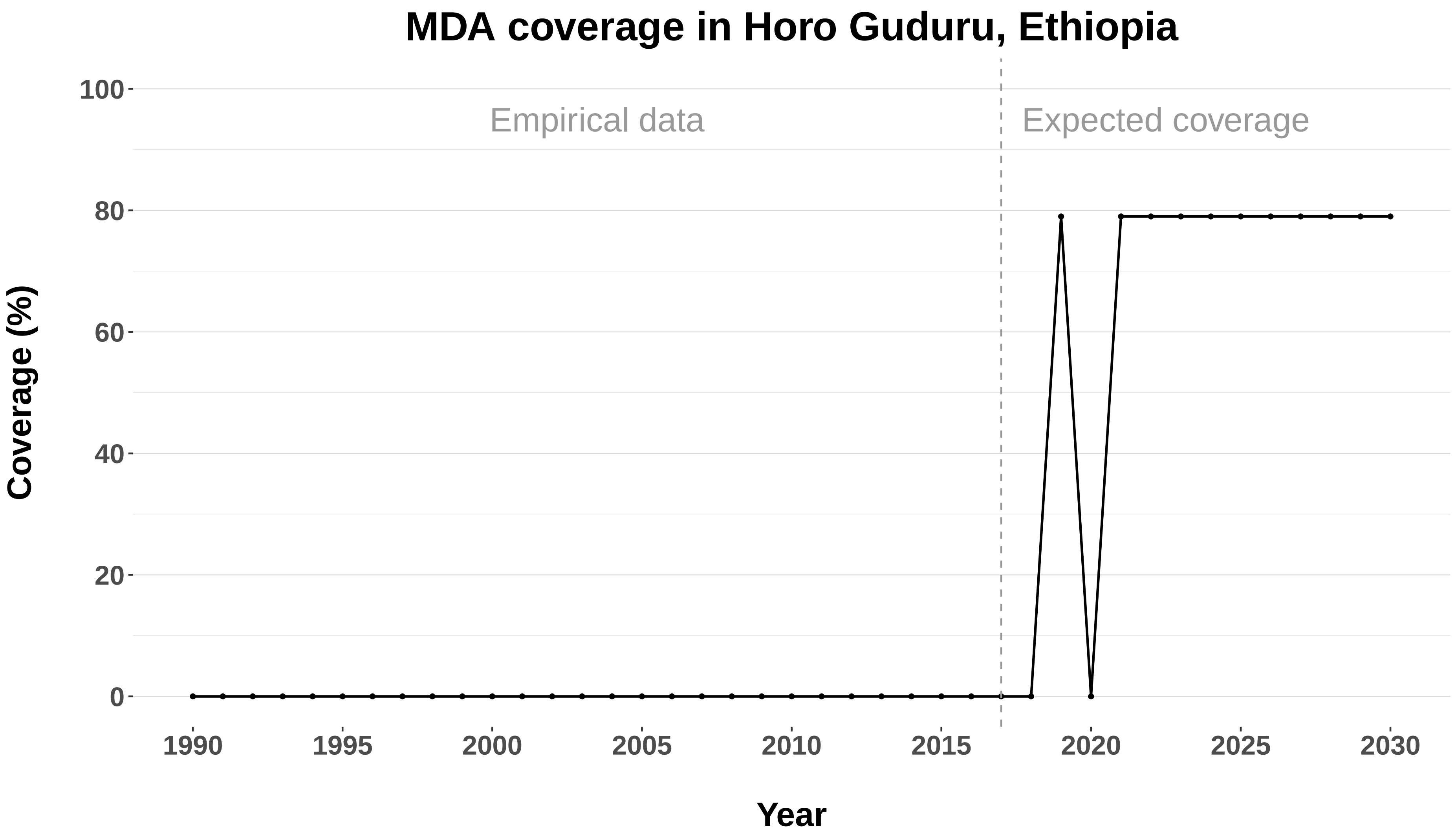
MDA coverage in Horo Guduru, Ethiopia
Empirical data
Expected coverage
Coverage (%)
100
80
60
40
20
0
1990
1995
2000
2005
2010
2015
2020
2025
2030
Year

**Project, country:**	**Illubabor, Ethiopia**
Bioclime:	
Endemicity level(s):	Hyperendemic
MDA start year:	2004
Mean MDA coverage per year:	76.9

	1990			2020			2030		
	N	%	DALYs	N	%	DALYs	N	%	DALYs
Total populaton at risk	435	100	_	981	100	_	1,287	100	_
**Infection:**									
Mf-positive	169	38.9	_	6	0.6	_	<0.5	<0.05	_
Mf-positive (age 5+)	169	38.9	_	6	0.6	_	<0.5	<0.05	_
Worm infected	214	49.3	_	63	6.4	_	3	0.2	_
**Clinical manifestations:**									
Palpable nodules	54	12.3	1	3	0.3	<0.5	<0.5	<0.05	<0.5
Reversible skin disease:									
Severe itch	25	5.8	5	1	0.1	<0.5	<0.5	<0.05	<0.5
Reactive skin disease	18	4.2	1	<0.5	<0.05	<0.5	<0.5	<0.05	<0.5
**Reversible skin disease**	**44**	**10.0**	**5**	**1**	**0.1**	**<0.5**	**<0.5**	**<0.05**	**<0.5**
Irreversible skin disease:									
Mild depigmentation	6	1.3	<0.5	6	0.6	<0.5	4	0.3	<0.5
Severe depigmentation	6	1.4	<0.5	5	0.5	<0.5	3	0.3	<0.5
Atrophy	<0.5	<0.05	<0.5	<0.5	<0.05	<0.5	<0.5	<0.05	<0.5
Hanging groin	<0.5	<0.05	<0.5	<0.5	<0.05	<0.5	<0.5	<0.05	<0.5
**Irreversible skin disease**	**12**	**2.7**	**<0.5**	**11**	**1.1**	**<0.5**	**7**	**0.6**	**<0.5**
**Total skin disease (OSD)**	**109**	**25.1**	**7**	**14**	**1.5**	**1**	**7**	**0.6**	**<0.5**
Eye disease (OED):									
Visual impairment	<0.5	<0.05	<0.5	<0.5	<0.05	<0.5	<0.5	<0.05	<0.5
Blindness	<0.5	<0.05	1	<0.5	<0.05	<0.5	<0.5	<0.05	<0.5
**Eye disease (OED)**	**<0.5**	**0.1**	**1**	**<0.5**	**<0.05**	**<0.5**	**<0.5**	**<0.05**	**<0.5**
**Total all manifestations**	**109**	**25.2**	**8**	**15**	**1.5**	**1**	**7**	**0.6**	**<0.5**

Note 1: Population at risk, number of cases (N) and DALYs are presented in thousands.

Note 2: Total skin disease is defined as the sum of the number of cases with palpable nodules, reversible and irreversible skin disease.

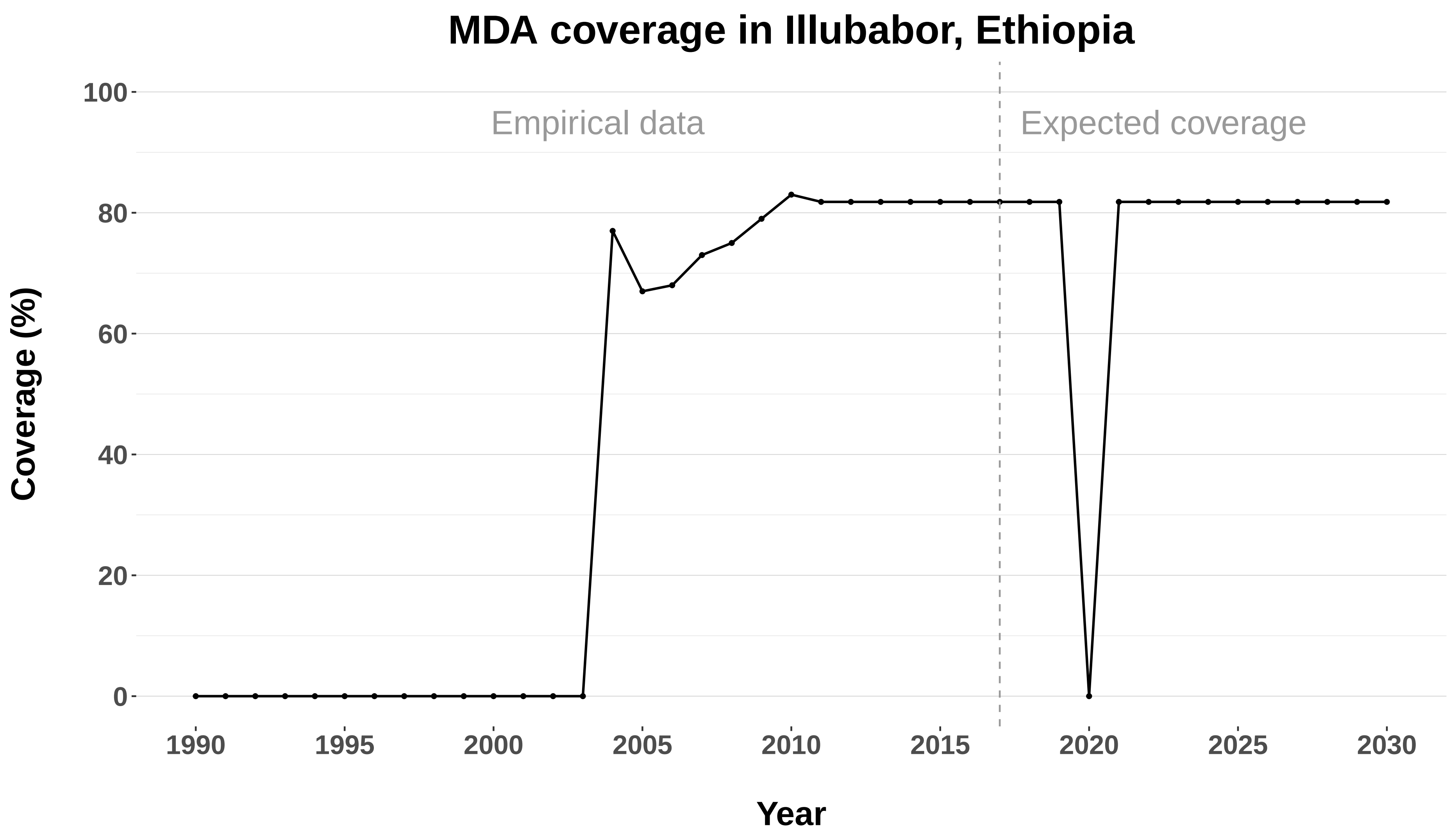
MDA coverage in Illubabor, Ethiopia
Empirical data
Expected coverage
Coverage (%)
100
80
60
40
20
0
1990
1995
2000
2005
2010
2015
2020
2025
2030
Year

**Project, country:**	**Jimma, Ethiopia**
Bioclime:	
Endemicity level(s):	Mesoendemic
MDA start year:	2004
Mean MDA coverage per year:	78.7

	1990			2020			2030		
	N	%	DALYs	N	%	DALYs	N	%	DALYs
Total populaton at risk	513	100	_	1,158	100	_	1,519	100	_
**Infection:**									
Mf-positive	145	28.2	_	1	0.1	_	<0.5	<0.05	_
Mf-positive (age 5+)	145	28.2	_	1	0.1	_	<0.5	<0.05	_
Worm infected	191	37.1	_	35	3.0	_	<0.5	<0.05	_
**Clinical manifestations:**									
Palpable nodules	29	5.6	<0.5	1	0.1	<0.5	<0.5	<0.05	<0.5
Reversible skin disease:									
Severe itch	19	3.7	3	<0.5	<0.05	<0.5	<0.5	<0.05	<0.5
Reactive skin disease	13	2.5	1	<0.5	<0.05	<0.5	<0.5	<0.05	<0.5
**Reversible skin disease**	**31**	**6.1**	**4**	**<0.5**	**<0.05**	**<0.5**	**<0.5**	**<0.05**	**<0.5**
Irreversible skin disease:									
Mild depigmentation	5	0.9	<0.5	4	0.4	<0.5	3	0.2	<0.5
Severe depigmentation	4	0.8	<0.5	4	0.3	<0.5	2	0.1	<0.5
Atrophy	<0.5	<0.05	<0.5	<0.5	<0.05	<0.5	<0.5	<0.05	<0.5
Hanging groin	<0.5	<0.05	<0.5	<0.5	<0.05	<0.5	<0.5	<0.05	<0.5
**Irreversible skin disease**	**9**	**1.8**	**<0.5**	**8**	**0.7**	**<0.5**	**5**	**0.3**	**<0.5**
**Total skin disease (OSD)**	**69**	**13.5**	**5**	**9**	**0.8**	**<0.5**	**5**	**0.3**	**<0.5**
Eye disease (OED):									
Visual impairment	<0.5	<0.05	<0.5	<0.5	<0.05	<0.5	<0.5	<0.05	<0.5
Blindness	<0.5	<0.05	<0.5	<0.5	<0.05	<0.5	<0.5	<0.05	<0.5
**Eye disease (OED)**	**<0.5**	**<0.05**	**<0.5**	**<0.5**	**<0.05**	**<0.5**	**<0.5**	**<0.05**	**<0.5**
**Total all manifestations**	**69**	**13.5**	**5**	**9**	**0.8**	**<0.5**	**5**	**0.3**	**<0.5**

Note 1: Population at risk, number of cases (N) and DALYs are presented in thousands.

Note 2: Total skin disease is defined as the sum of the number of cases with palpable nodules, reversible and irreversible skin disease.

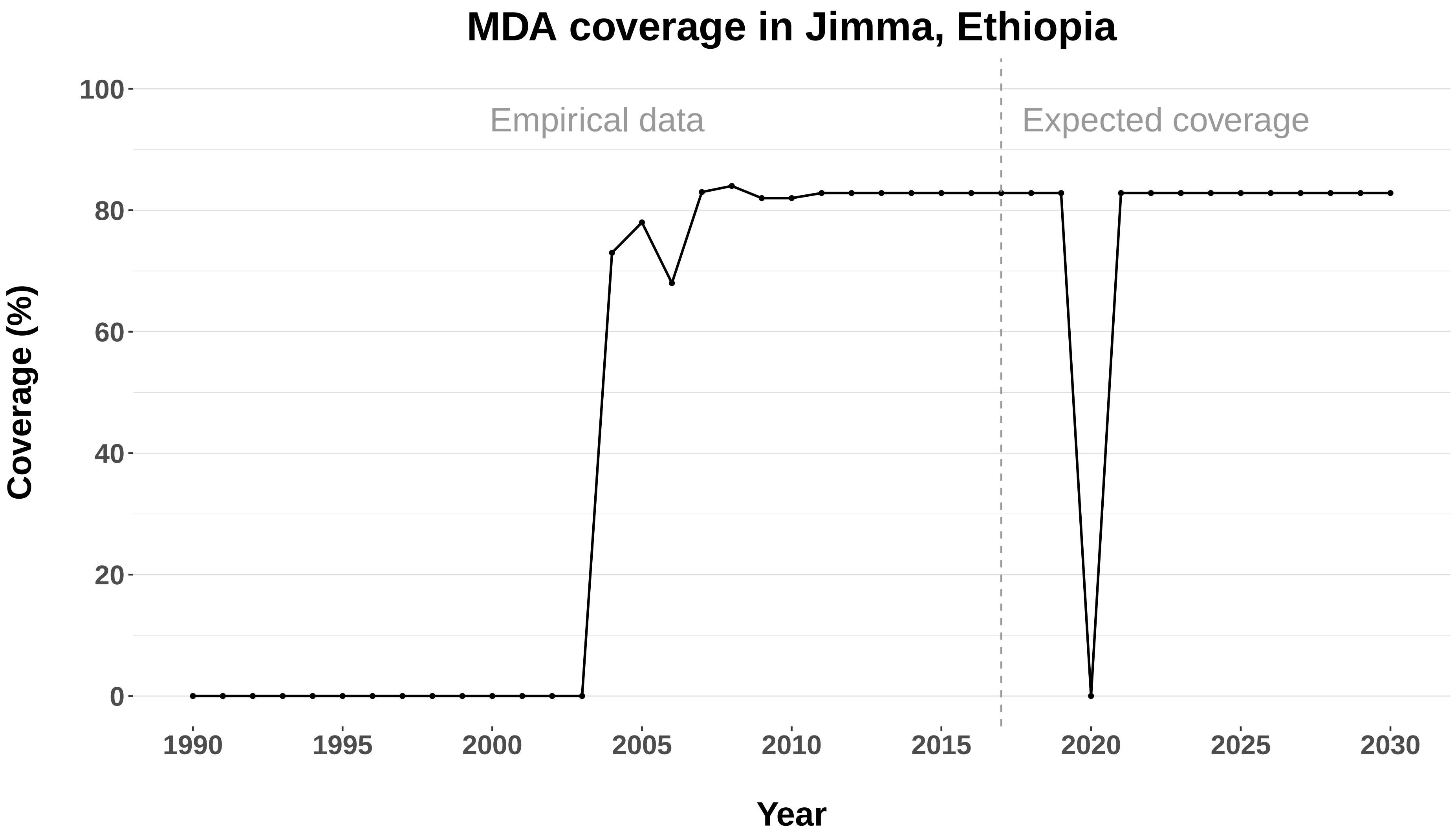
MDA coverage in Jimma, Ethiopia
Empirical data
Expected coverage
Coverage (%)
100
80
60
40
20
0
1990
1995
2000
2005
2010
2015
2020
2025
2030
Year

**Project, country:**	**Kaffa-Sheka, Ethiopia**
Bioclime:	
Endemicity level(s):	Hyperendemic
MDA start year:	2001
Mean MDA coverage per year:	71.8

	1990			2020			2030		
	N	%	DALYs	N	%	DALYs	N	%	DALYs
Total populaton at risk	729	100	_	1,645	100	_	2,157	100	_
**Infection:**									
Mf-positive	326	44.8	_	7	0.4	_	<0.5	<0.05	_
Mf-positive (age 5+)	326	44.7	_	7	0.4	_	<0.5	<0.05	_
Worm infected	396	54.4	_	90	5.5	_	3	0.1	_
**Clinical manifestations:**									
Palpable nodules	113	15.5	1	3	0.2	<0.5	<0.5	<0.05	<0.5
Reversible skin disease:									
Severe itch	52	7.2	9	1	<0.05	<0.5	<0.5	<0.05	<0.5
Reactive skin disease	38	5.3	2	<0.5	<0.05	<0.5	<0.5	<0.05	<0.5
**Reversible skin disease**	**91**	**12.4**	**11**	**1**	**<0.05**	**<0.5**	**<0.5**	**<0.05**	**<0.5**
Irreversible skin disease:									
Mild depigmentation	12	1.6	<0.5	11	0.7	<0.5	7	0.3	<0.5
Severe depigmentation	12	1.7	1	10	0.6	1	6	0.3	<0.5
Atrophy	<0.5	<0.05	<0.5	<0.5	<0.05	<0.5	<0.5	<0.05	<0.5
Hanging groin	<0.5	<0.05	<0.5	<0.5	<0.05	<0.5	<0.5	<0.05	<0.5
**Irreversible skin disease**	**24**	**3.4**	**1**	**21**	**1.3**	**1**	**14**	**0.6**	**<0.5**
**Total skin disease (OSD)**	**228**	**31.3**	**13**	**25**	**1.5**	**1**	**14**	**0.6**	**<0.5**
Eye disease (OED):									
Visual impairment	<0.5	0.1	<0.5	<0.5	<0.05	<0.5	<0.5	<0.05	<0.5
Blindness	<0.5	0.1	2	<0.5	<0.05	1	<0.5	<0.05	<0.5
**Eye disease (OED)**	**1**	**0.1**	**2**	**<0.5**	**<0.05**	**1**	**<0.5**	**<0.05**	**<0.5**
**Total all manifestations**	**229**	**31.5**	**16**	**25**	**1.5**	**2**	**14**	**0.6**	**1**

Note 1: Population at risk, number of cases (N) and DALYs are presented in thousands.

Note 2: Total skin disease is defined as the sum of the number of cases with palpable nodules, reversible and irreversible skin disease.

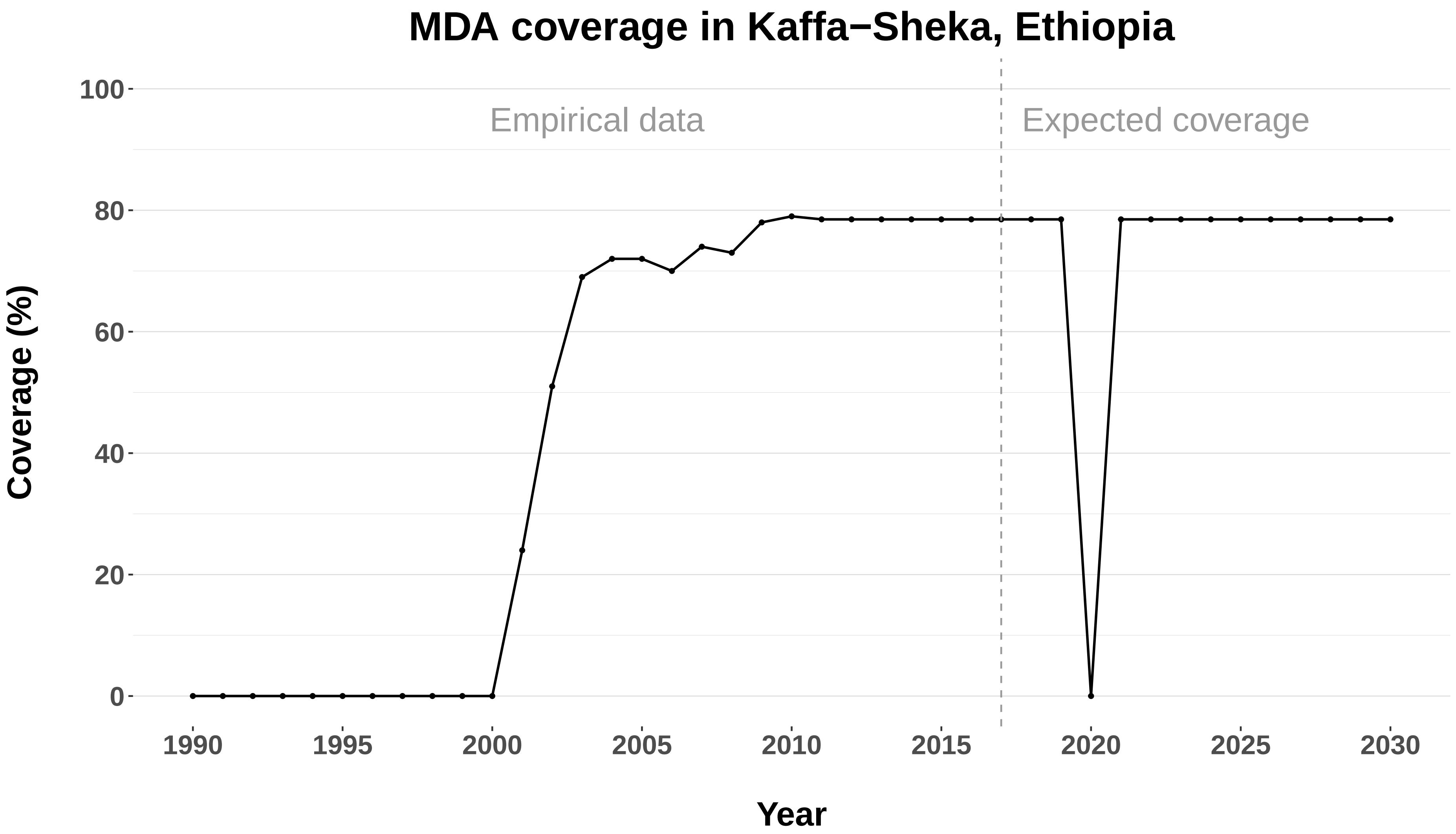
MDA coverage in Kaffa−Sheka, Ethiopia
Empirical data
Expected coverage
Coverage (%)
100
80
60
40
20
0
1990
1995
2000
2005
2010
2015
2020
2025
2030
Year

**Project, country:**	**Kamashi, Ethiopia**
Bioclime:	
Endemicity level(s):	Hyperendemic
MDA start year:	2016
Mean MDA coverage per year:	74.7

	1990			2020			2030		
	N	%	DALYs	N	%	DALYs	N	%	DALYs
Total populaton at risk	271	100	_	612	100	_	802	100	_
**Infection:**									
Mf-positive	102	37.7	_	55	9.0	_	<0.5	<0.05	_
Mf-positive (age 5+)	102	37.7	_	55	9.0	_	<0.5	<0.05	_
Worm infected	129	47.5	_	235	38.4	_	30	3.8	_
**Clinical manifestations:**									
Palpable nodules	33	12.3	<0.5	52	8.4	1	1	0.1	<0.5
Reversible skin disease:									
Severe itch	16	5.9	3	16	2.6	3	1	0.1	<0.5
Reactive skin disease	12	4.4	1	3	0.6	<0.5	<0.5	<0.05	<0.5
**Reversible skin disease**	**28**	**10.2**	**3**	**20**	**3.2**	**3**	**1**	**0.1**	**<0.5**
Irreversible skin disease:									
Mild depigmentation	4	1.3	<0.5	6	1.1	<0.5	5	0.6	<0.5
Severe depigmentation	4	1.4	<0.5	7	1.1	<0.5	5	0.6	<0.5
Atrophy	<0.5	<0.05	<0.5	<0.5	<0.05	<0.5	<0.5	<0.05	<0.5
Hanging groin	<0.5	<0.05	<0.5	<0.5	<0.05	<0.5	<0.5	<0.05	<0.5
**Irreversible skin disease**	**8**	**2.8**	**<0.5**	**14**	**2.2**	**1**	**10**	**1.3**	**<0.5**
**Total skin disease (OSD)**	**69**	**25.3**	**4**	**85**	**13.9**	**4**	**12**	**1.5**	**1**
Eye disease (OED):									
Visual impairment	<0.5	0.1	<0.5	<0.5	<0.05	<0.5	<0.5	<0.05	<0.5
Blindness	<0.5	0.1	1	<0.5	<0.05	2	<0.5	<0.05	1
**Eye disease (OED)**	**<0.5**	**0.1**	**1**	**1**	**0.1**	**2**	**<0.5**	**<0.05**	**1**
**Total all manifestations**	**69**	**25.5**	**5**	**85**	**13.9**	**6**	**12**	**1.5**	**1**

Note 1: Population at risk, number of cases (N) and DALYs are presented in thousands.

Note 2: Total skin disease is defined as the sum of the number of cases with palpable nodules, reversible and irreversible skin disease.

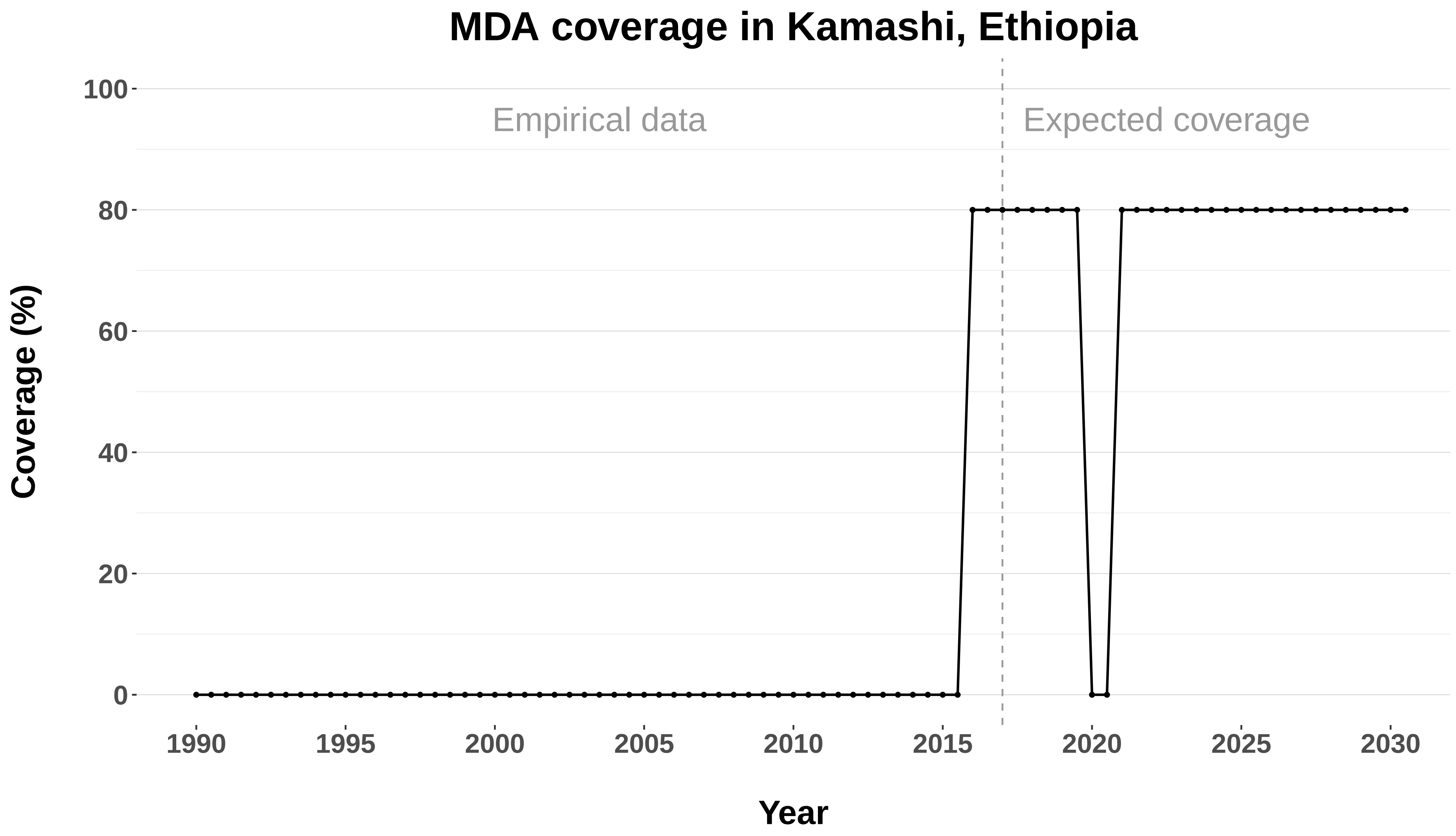
MDA coverage in Kamashi, Ethiopia
Empirical data
Expected coverage
Coverage (%)
Year
0
20
40
60
80
100
1990
1995
2000
2005
2010
2015
2020
2025
2030

**Project, country:**	**Metekel, Ethiopia**
Bioclime:	
Endemicity level(s):	Mesoendemic
MDA start year:	2004
Mean MDA coverage per year:	66.8

	1990			2020			2030		
	N	%	DALYs	N	%	DALYs	N	%	DALYs
Total populaton at risk	93	100	_	209	100	_	275	100	_
**Infection:**									
Mf-positive	28	30.1	_	2	0.8	_	<0.5	<0.05	_
Mf-positive (age 5+)	28	30.1	_	2	0.8	_	<0.5	<0.05	_
Worm infected	37	39.5	_	15	7.2	_	1	0.2	_
**Clinical manifestations:**									
Palpable nodules	6	6.6	<0.5	<0.5	0.2	<0.5	<0.5	<0.05	<0.5
Reversible skin disease:									
Severe itch	4	4.0	1	<0.5	0.1	<0.5	<0.5	<0.05	<0.5
Reactive skin disease	3	2.7	<0.5	<0.5	<0.05	<0.5	<0.5	<0.05	<0.5
**Reversible skin disease**	**6**	**6.7**	**1**	**<0.5**	**0.1**	**<0.5**	**<0.5**	**<0.05**	**<0.5**
Irreversible skin disease:									
Mild depigmentation	1	1.0	<0.5	1	0.5	<0.5	1	0.2	<0.5
Severe depigmentation	1	0.9	<0.5	1	0.4	<0.5	<0.5	0.2	<0.5
Atrophy	<0.5	<0.05	<0.5	<0.5	<0.05	<0.5	<0.5	<0.05	<0.5
Hanging groin	<0.5	<0.05	<0.5	<0.5	<0.05	<0.5	<0.5	<0.05	<0.5
**Irreversible skin disease**	**2**	**1.9**	**<0.5**	**2**	**0.8**	**<0.5**	**1**	**0.4**	**<0.5**
**Total skin disease (OSD)**	**14**	**15.2**	**1**	**2**	**1.1**	**<0.5**	**1**	**0.4**	**<0.5**
Eye disease (OED):									
Visual impairment	<0.5	<0.05	<0.5	<0.5	<0.05	<0.5	<0.5	<0.05	<0.5
Blindness	<0.5	<0.05	<0.5	<0.5	<0.05	<0.5	<0.5	<0.05	<0.5
**Eye disease (OED)**	**<0.5**	**<0.05**	**<0.5**	**<0.5**	**<0.05**	**<0.5**	**<0.5**	**<0.05**	**<0.5**
**Total all manifestations**	**14**	**15.2**	**1**	**2**	**1.1**	**<0.5**	**1**	**0.4**	**<0.5**

Note 1: Population at risk, number of cases (N) and DALYs are presented in thousands.

Note 2: Total skin disease is defined as the sum of the number of cases with palpable nodules, reversible and irreversible skin disease.

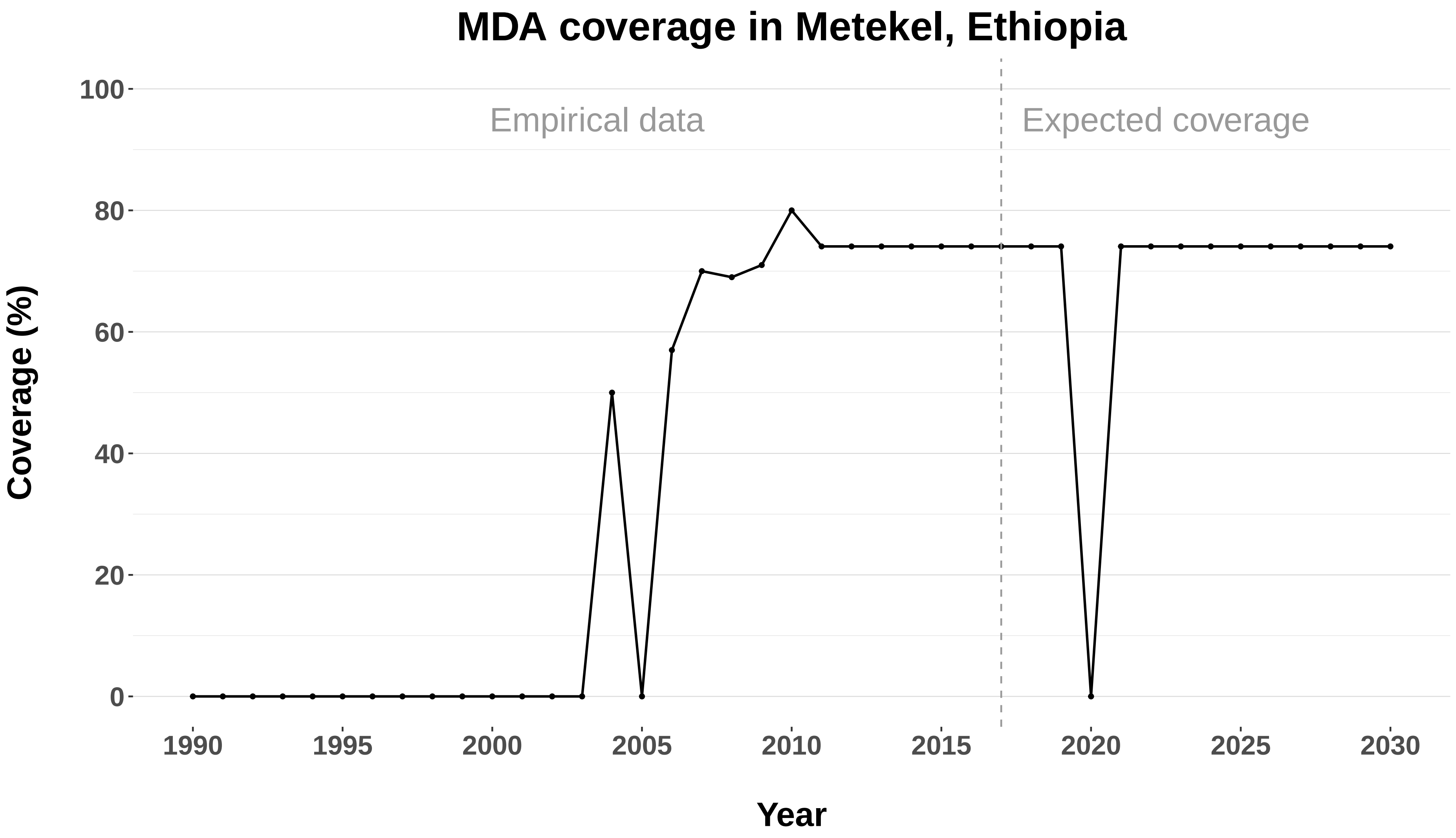

MDA coverage in Metekel, Ethiopia
Empirical data
Expected coverage
Coverage (%)
100
80
60
40
20
0
1990
1995
2000
2005
2010
2015
2020
2025
2030
Year

**Project, country:**	**North Gondar, Ethiopia**
Bioclime:	
Endemicity level(s):	Mesoendemic
MDA start year:	2003
Mean MDA coverage per year:	68.3

	1990			2020			2030		
	N	%	DALYs	N	%	DALYs	N	%	DALYs
Total populaton at risk	180	100	_	406	100	_	533	100	_
**Infection:**									
Mf-positive	51	28.5	_	2	0.5	_	<0.5	<0.05	_
Mf-positive (age 5+)	51	28.5	_	2	0.5	_	<0.5	<0.05	_
Worm infected	67	37.0	_	21	5.3	_	1	0.2	_
**Clinical manifestations:**									
Palpable nodules	11	5.9	<0.5	1	0.2	<0.5	<0.5	<0.05	<0.5
Reversible skin disease:									
Severe itch	7	3.9	1	<0.5	0.1	<0.5	<0.5	<0.05	<0.5
Reactive skin disease	5	2.7	<0.5	<0.5	<0.05	<0.5	<0.5	<0.05	<0.5
**Reversible skin disease**	**12**	**6.6**	**1**	**<0.5**	**0.1**	**<0.5**	**<0.5**	**<0.05**	**<0.5**
Irreversible skin disease:									
Mild depigmentation	2	1.0	<0.5	2	0.4	<0.5	1	0.2	<0.5
Severe depigmentation	2	0.9	<0.5	1	0.4	<0.5	1	0.2	<0.5
Atrophy	<0.5	<0.05	<0.5	<0.5	<0.05	<0.5	<0.5	<0.05	<0.5
Hanging groin	<0.5	<0.05	<0.5	<0.5	<0.05	<0.5	<0.5	<0.05	<0.5
**Irreversible skin disease**	**3**	**1.9**	**<0.5**	**3**	**0.8**	**<0.5**	**2**	**0.4**	**<0.5**
**Total skin disease (OSD)**	**26**	**14.4**	**2**	**4**	**1.0**	**<0.5**	**2**	**0.4**	**<0.5**
Eye disease (OED):									
Visual impairment	<0.5	<0.05	<0.5	<0.5	<0.05	<0.5	<0.5	<0.05	<0.5
Blindness	<0.5	<0.05	<0.5	<0.5	<0.05	<0.5	<0.5	<0.05	<0.5
**Eye disease (OED)**	**<0.5**	**<0.05**	**<0.5**	**<0.5**	**<0.05**	**<0.5**	**<0.5**	**<0.05**	**<0.5**
**Total all manifestations**	**26**	**14.4**	**2**	**4**	**1.0**	**<0.5**	**2**	**0.4**	**<0.5**

Note 1: Population at risk, number of cases (N) and DALYs are presented in thousands.

Note 2: Total skin disease is defined as the sum of the number of cases with palpable nodules, reversible and irreversible skin disease.

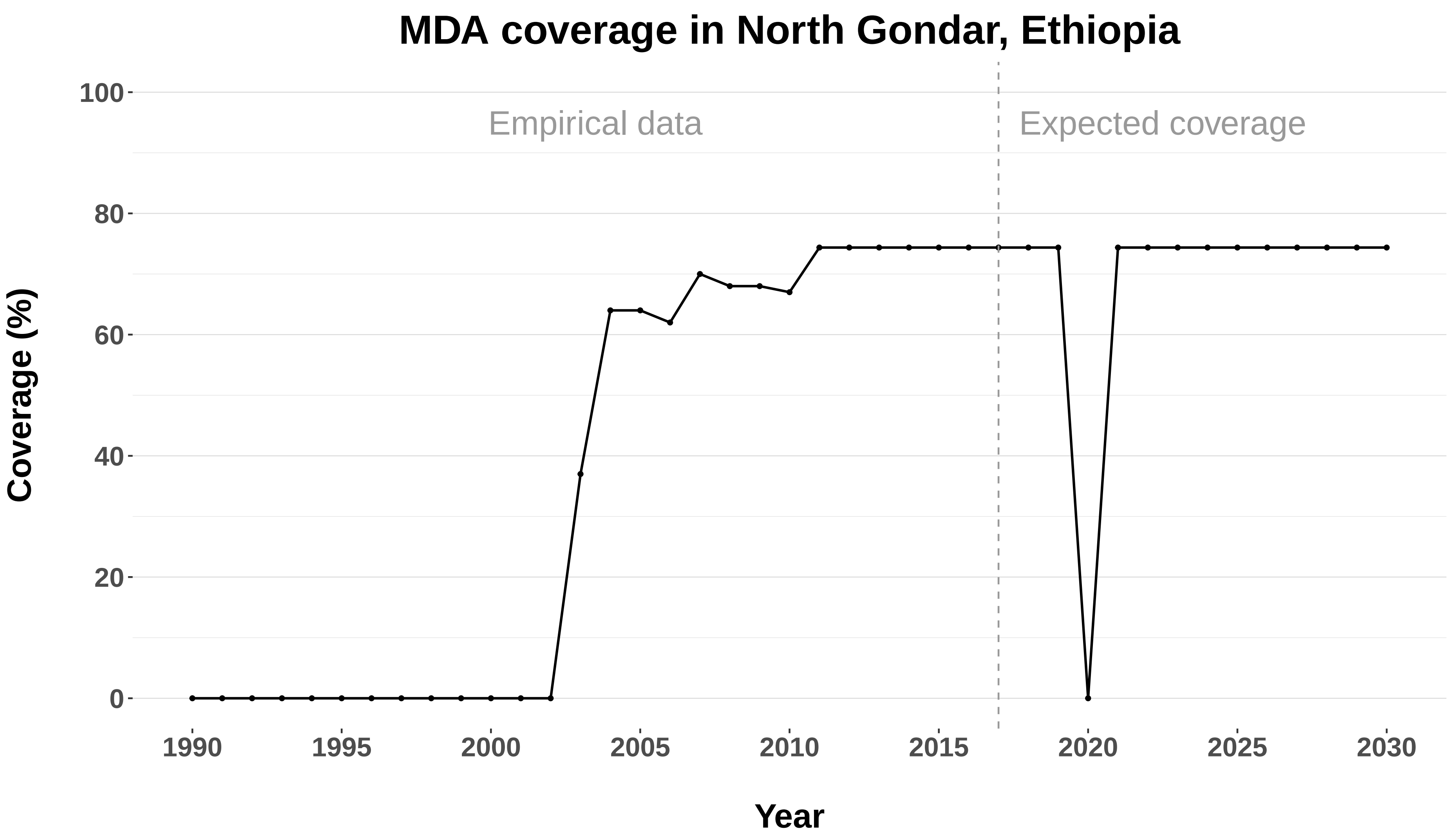

MDA coverage in North Gondar, Ethiopia
Empirical data
Expected coverage
Coverage (%)
0
20
40
60
80
100
1990
1995
2000
2005
2010
2015
2020
2025
2030
Year

**Project, country:**	**NY East Wellega, Ethiopia**
Bioclime:	
Endemicity level(s):	Hyperendemic
MDA start year:	2016
Mean MDA coverage per year:	74.7

	1990			2020			2030		
	N	%	DALYs	N	%	DALYs	N	%	DALYs
Total populaton at risk	165	100	_	371	100	_	487	100	_
**Infection:**									
Mf-positive	63	38.5	_	96	25.8	_	8	1.7	_
Mf-positive (age 5+)	63	38.4	_	96	25.8	_	8	1.7	_
Worm infected	81	49.0	_	155	41.8	_	55	11.2	_
**Clinical manifestations:**									
Palpable nodules	20	12.2	<0.5	34	9.2	<0.5	3	0.7	<0.5
Reversible skin disease:									
Severe itch	9	5.7	2	10	2.7	2	1	0.1	<0.5
Reactive skin disease	7	4.1	<0.5	3	0.7	<0.5	<0.5	<0.05	<0.5
**Reversible skin disease**	**16**	**9.8**	**2**	**13**	**3.4**	**2**	**1**	**0.2**	**<0.5**
Irreversible skin disease:									
Mild depigmentation	2	1.3	<0.5	4	1.1	<0.5	3	0.6	<0.5
Severe depigmentation	2	1.3	<0.5	4	1.1	<0.5	3	0.6	<0.5
Atrophy	<0.5	<0.05	<0.5	<0.5	<0.05	<0.5	<0.5	<0.05	<0.5
Hanging groin	<0.5	<0.05	<0.5	<0.5	<0.05	<0.5	<0.5	<0.05	<0.5
**Irreversible skin disease**	**4**	**2.7**	**<0.5**	**8**	**2.2**	**<0.5**	**6**	**1.2**	**<0.5**
**Total skin disease (OSD)**	**41**	**24.7**	**2**	**55**	**14.8**	**3**	**10**	**2.1**	**<0.5**
Eye disease (OED):									
Visual impairment	<0.5	<0.05	<0.5	<0.5	<0.05	<0.5	<0.5	<0.05	<0.5
Blindness	<0.5	<0.05	<0.5	<0.5	<0.05	1	<0.5	<0.05	<0.5
**Eye disease (OED)**	**<0.5**	**0.1**	**<0.5**	**<0.5**	**0.1**	**1**	**<0.5**	**<0.05**	**<0.5**
**Total all manifestations**	**41**	**24.8**	**3**	**55**	**14.9**	**3**	**10**	**2.1**	**1**

Note 1: Population at risk, number of cases (N) and DALYs are presented in thousands.

Note 2: Total skin disease is defined as the sum of the number of cases with palpable nodules, reversible and irreversible skin disease.

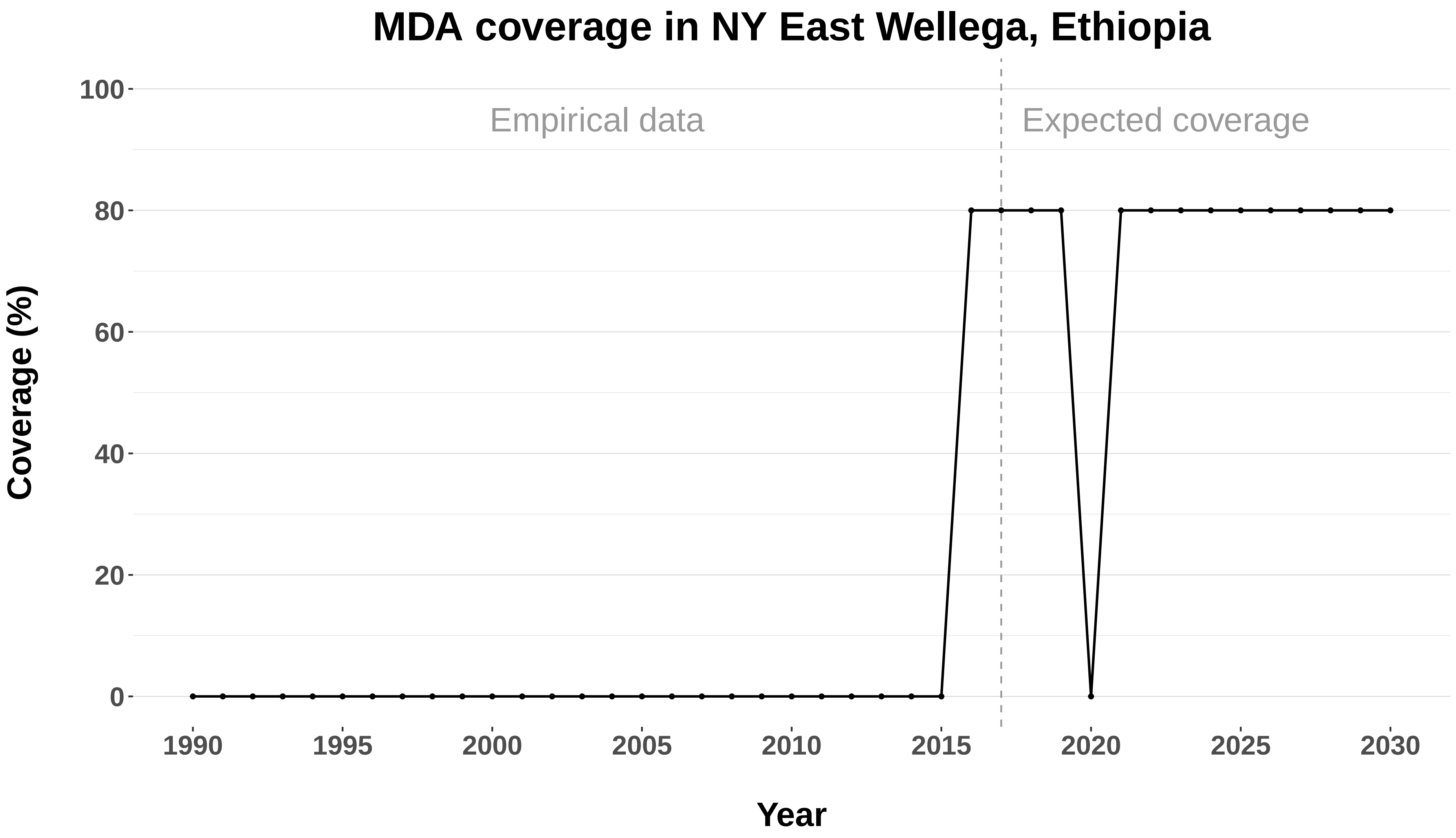
MDA coverage in NY East Wellega, Ethiopia
Empirical data
Expected coverage
Coverage (%)
100
80
60
40
20
0
1990
1995
2000
2005
2010
2015
2020
2025
2030
Year

**Project, country:**	**NY West Wellega, Ethiopia**
Bioclime:	
Endemicity level(s):	Mesoendemic
MDA start year:	2016
Mean MDA coverage per year:	73.7

	1990			2020			2030		
	N	%	DALYs	N	%	DALYs	N	%	DALYs
Total populaton at risk	167	100	_	377	100	_	495	100	_
**Infection:**									
Mf-positive	41	24.8	_	18	4.8	_	<0.5	<0.05	_
Mf-positive (age 5+)	41	24.8	_	18	4.8	_	<0.5	<0.05	_
Worm infected	56	33.7	_	96	25.4	_	9	1.7	_
**Clinical manifestations:**									
Palpable nodules	7	4.2	<0.5	9	2.5	<0.5	<0.5	<0.05	<0.5
Reversible skin disease:									
Severe itch	5	3.0	1	4	1.1	1	<0.5	<0.05	<0.5
Reactive skin disease	3	2.0	<0.5	1	0.1	<0.5	<0.5	<0.05	<0.5
**Reversible skin disease**	**8**	**5.0**	**1**	**5**	**1.3**	**1**	**<0.5**	**<0.05**	**<0.5**
Irreversible skin disease:									
Mild depigmentation	1	0.8	<0.5	2	0.6	<0.5	2	0.4	<0.5
Severe depigmentation	1	0.7	<0.5	2	0.5	<0.5	1	0.3	<0.5
Atrophy	<0.5	<0.05	<0.5	<0.5	<0.05	<0.5	<0.5	<0.05	<0.5
Hanging groin	<0.5	<0.05	<0.5	<0.5	<0.05	<0.5	<0.5	<0.05	<0.5
**Irreversible skin disease**	**2**	**1.5**	**<0.5**	**4**	**1.1**	**<0.5**	**3**	**0.6**	**<0.5**
**Total skin disease (OSD)**	**18**	**10.7**	**1**	**18**	**4.9**	**1**	**3**	**0.7**	**<0.5**
Eye disease (OED):									
Visual impairment	<0.5	<0.05	<0.5	<0.5	<0.05	<0.5	<0.5	<0.05	<0.5
Blindness	<0.5	<0.05	<0.5	<0.5	<0.05	<0.5	<0.5	<0.05	<0.5
**Eye disease (OED)**	**<0.5**	**<0.05**	**<0.5**	**<0.5**	**<0.05**	**<0.5**	**<0.5**	**<0.05**	**<0.5**
**Total all manifestations**	**18**	**10.7**	**1**	**18**	**4.9**	**1**	**3**	**0.7**	**<0.5**

Note 1: Population at risk, number of cases (N) and DALYs are presented in thousands.

Note 2: Total skin disease is defined as the sum of the number of cases with palpable nodules, reversible and irreversible skin disease.

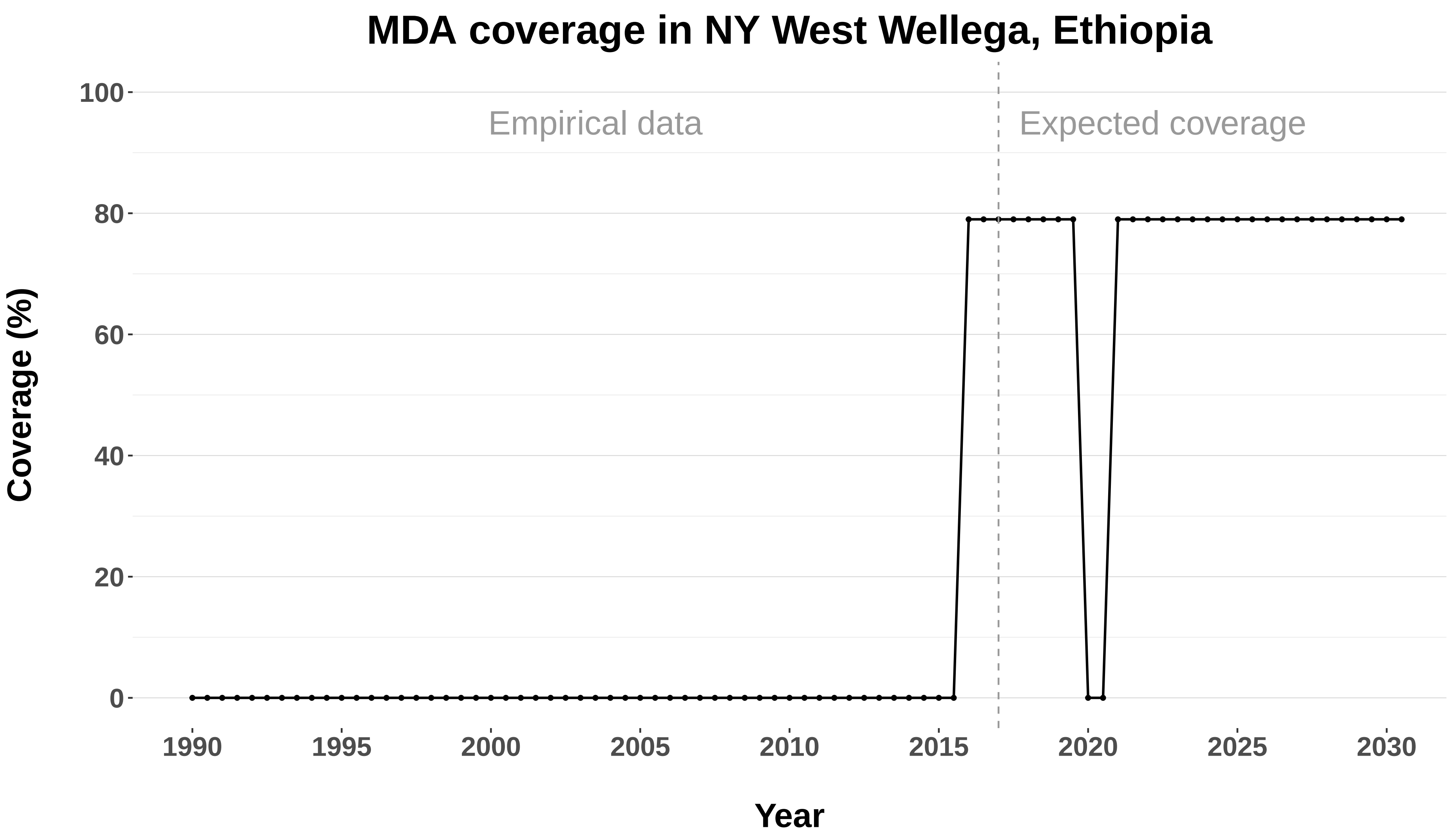
MDA coverage in NY West Wellega, Ethiopia
Empirical data
Expected coverage
Coverage (%)
100
80
60
40
20
0
1990
1995
2000
2005
2010
2015
2020
2025
2030
Year

**Project, country:**	**P20Ethiopia, Ethiopia**
Bioclime:	
Endemicity level(s):	Hyperendemic
MDA start year:	2019
Mean MDA coverage per year:	73.3

	1990			2020			2030		
	N	%	DALYs	N	%	DALYs	N	%	DALYs
Total populaton at risk	191	100	_	431	100	_	566	100	_
**Infection:**									
Mf-positive	81	42.1	_	168	39.0	_	39	6.9	_
Mf-positive (age 5+)	80	42.1	_	168	39.0	_	39	6.9	_
Worm infected	101	52.8	_	223	51.8	_	136	24.1	_
**Clinical manifestations:**									
Palpable nodules	27	14.2	<0.5	60	14.0	1	16	2.9	<0.5
Reversible skin disease:									
Severe itch	12	6.4	2	24	5.5	4	4	0.6	1
Reactive skin disease	9	4.7	<0.5	14	3.3	1	<0.5	<0.05	<0.5
**Reversible skin disease**	**21**	**11.2**	**3**	**38**	**8.8**	**5**	**4**	**0.7**	**1**
Irreversible skin disease:									
Mild depigmentation	3	1.5	<0.5	6	1.3	<0.5	5	0.9	<0.5
Severe depigmentation	3	1.5	<0.5	6	1.4	<0.5	5	0.9	<0.5
Atrophy	<0.5	<0.05	<0.5	<0.5	<0.05	<0.5	<0.5	<0.05	<0.5
Hanging groin	<0.5	<0.05	<0.5	<0.5	<0.05	<0.5	<0.5	<0.05	<0.5
**Irreversible skin disease**	**6**	**3.0**	**<0.5**	**12**	**2.8**	**1**	**10**	**1.7**	**<0.5**
**Total skin disease (OSD)**	**54**	**28.4**	**3**	**110**	**25.6**	**6**	**30**	**5.3**	**1**
Eye disease (OED):									
Visual impairment	<0.5	0.1	<0.5	<0.5	0.1	<0.5	<0.5	<0.05	<0.5
Blindness	<0.5	0.1	1	<0.5	0.1	1	<0.5	<0.05	1
**Eye disease (OED)**	**<0.5**	**0.1**	**1**	**<0.5**	**0.1**	**1**	**<0.5**	**<0.05**	**1**
**Total all manifestations**	**55**	**28.5**	**4**	**111**	**25.7**	**8**	**30**	**5.3**	**2**

Note 1: Population at risk, number of cases (N) and DALYs are presented in thousands.

Note 2: Total skin disease is defined as the sum of the number of cases with palpable nodules, reversible and irreversible skin disease.

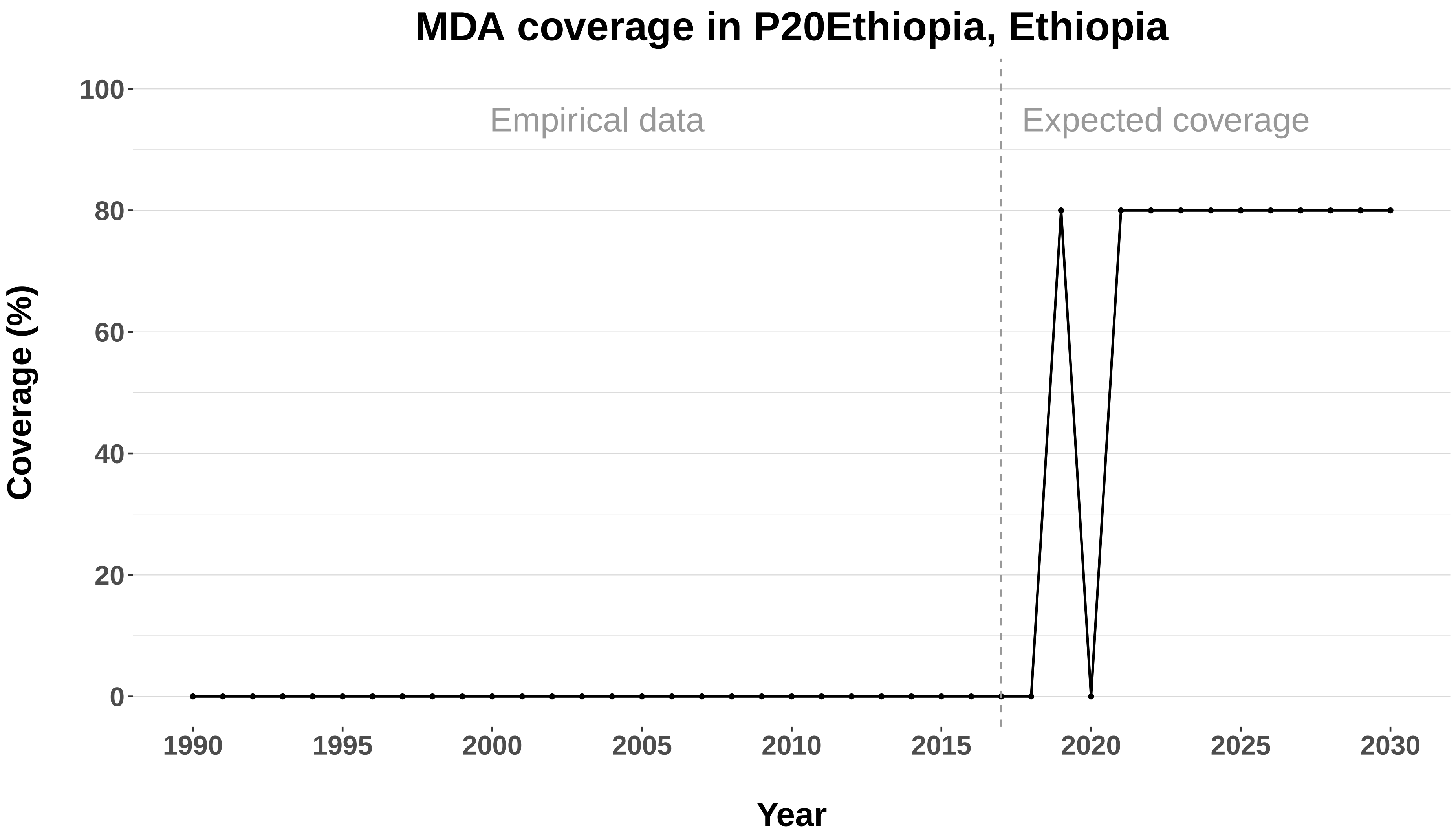

MDA coverage in P20Ethiopia, Ethiopia
Empirical data
Expected coverage
Coverage (%)
100
80
60
40
20
0
1990
1995
2000
2005
2010
2015
2020
2025
2030
Year

**Project, country:**	**P5Ethiopia, Ethiopia**
Bioclime:	
Endemicity level(s):	Hypoendemic
MDA start year:	2023
Mean MDA coverage per year:	65.0

	1990			2020			2030		
	N	%	DALYs	N	%	DALYs	N	%	DALYs
Total populaton at risk	2,018	100	_	4,553	100	_	5,972	100	_
**Infection:**									
Mf-positive	494	24.5	_	1,059	23.3	_	619	10.4	_
Mf-positive (age 5+)	494	24.5	_	1,059	23.2	_	619	10.4	_
Worm infected	656	32.5	_	1,424	31.3	_	1,327	22.2	_
**Clinical manifestations:**									
Palpable nodules	75	3.7	1	161	3.5	2	100	1.7	1
Reversible skin disease:									
Severe itch	62	3.1	11	130	2.9	24	47	0.8	8
Reactive skin disease	41	2.0	2	89	2.0	4	10	0.2	<0.5
**Reversible skin disease**	**103**	**5.1**	**13**	**219**	**4.8**	**28**	**56**	**0.9**	**9**
Irreversible skin disease:									
Mild depigmentation	16	0.8	<0.5	36	0.8	<0.5	36	0.6	<0.5
Severe depigmentation	14	0.7	1	30	0.7	2	28	0.5	2
Atrophy	<0.5	<0.05	<0.5	<0.5	<0.05	<0.5	<0.5	<0.05	<0.5
Hanging groin	<0.5	<0.05	<0.5	<0.5	<0.05	<0.5	<0.5	<0.05	<0.5
**Irreversible skin disease**	**30**	**1.5**	**1**	**66**	**1.5**	**2**	**64**	**1.1**	**2**
**Total skin disease (OSD)**	**209**	**10.3**	**15**	**447**	**9.8**	**32**	**220**	**3.7**	**12**
Eye disease (OED):									
Visual impairment	<0.5	<0.05	<0.5	1	<0.05	<0.5	<0.5	<0.05	<0.5
Blindness	<0.5	<0.05	1	<0.5	<0.05	2	<0.5	<0.05	1
**Eye disease (OED)**	**<0.5**	**<0.05**	**1**	**1**	**<0.05**	**2**	**1**	**<0.05**	**1**
**Total all manifestations**	**209**	**10.4**	**16**	**448**	**9.8**	**34**	**221**	**3.7**	**14**

Note 1: Population at risk, number of cases (N) and DALYs are presented in thousands.

Note 2: Total skin disease is defined as the sum of the number of cases with palpable nodules, reversible and irreversible skin disease.

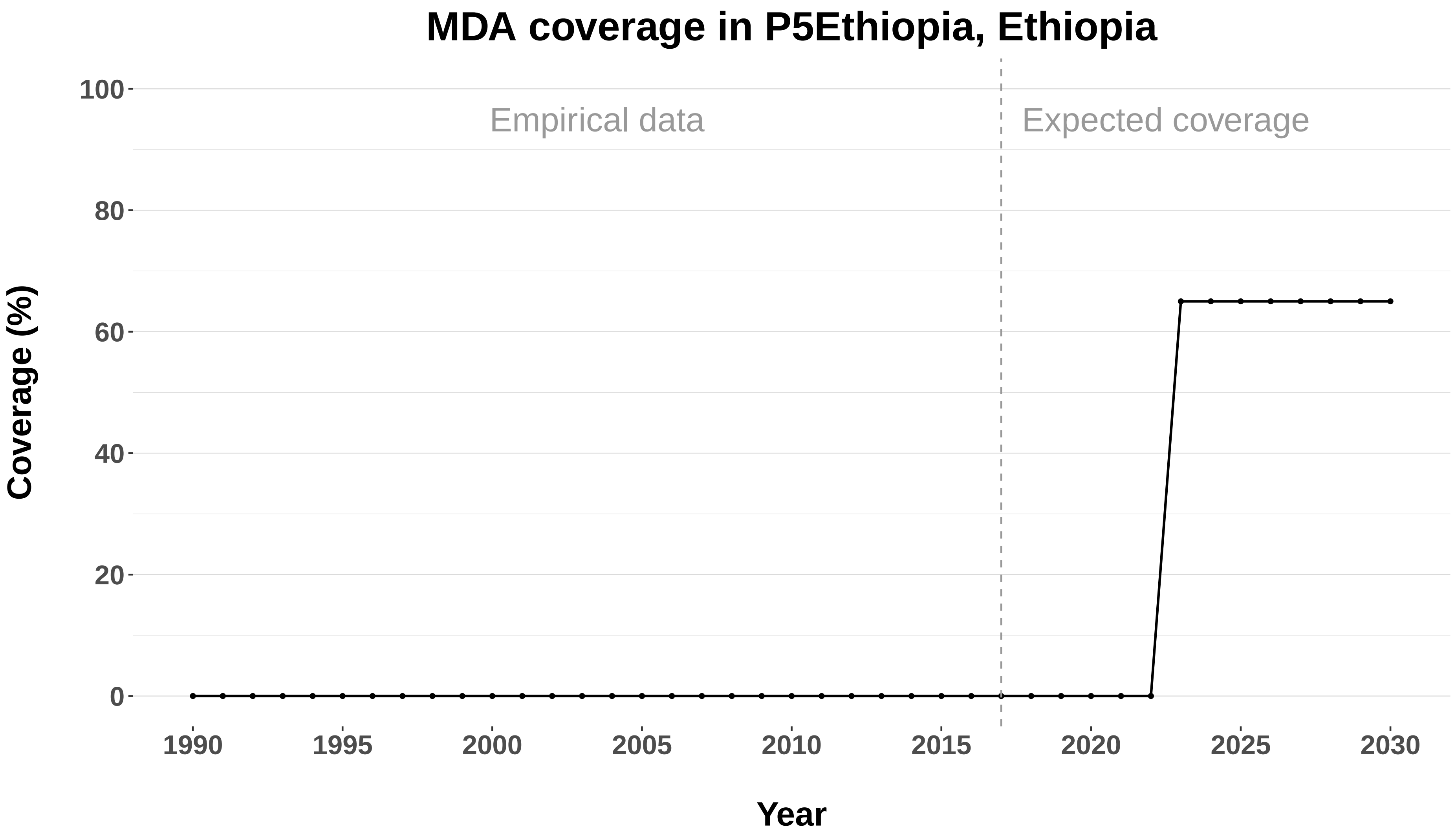
MDA coverage in P5Ethiopia, Ethiopia
Empirical data
Expected coverage
Coverage (%)
100
80
60
40
20
0
1990
1995
2000
2005
2010
2015
2020
2025
2030
Year

**Project, country:**	**West Shewa, Ethiopia**
Bioclime:	
Endemicity level(s):	Hypoendemic
MDA start year:	2023
Mean MDA coverage per year:	65.0

	1990			2020			2030		
	N	%	DALYs	N	%	DALYs	N	%	DALYs
Total populaton at risk	33	100	_	75	100	_	98	100	_
**Infection:**									
Mf-positive	7	22.1	_	16	21.0	_	9	9.0	_
Mf-positive (age 5+)	7	22.1	_	16	21.0	_	9	9.0	_
Worm infected	10	30.0	_	22	28.7	_	20	20.0	_
**Clinical manifestations:**									
Palpable nodules	1	2.7	<0.5	2	2.6	<0.5	1	1.2	<0.5
Reversible skin disease:									
Severe itch	1	2.7	<0.5	2	2.5	<0.5	1	0.7	<0.5
Reactive skin disease	1	1.8	<0.5	1	1.7	<0.5	<0.5	0.1	<0.5
**Reversible skin disease**	**1**	**4.5**	**<0.5**	**3**	**4.2**	**<0.5**	**1**	**0.8**	**<0.5**
Irreversible skin disease:									
Mild depigmentation	<0.5	0.7	<0.5	1	0.7	<0.5	<0.5	0.5	<0.5
Severe depigmentation	<0.5	0.6	<0.5	<0.5	0.6	<0.5	<0.5	0.4	<0.5
Atrophy	<0.5	<0.05	<0.5	<0.5	<0.05	<0.5	<0.5	<0.05	<0.5
Hanging groin	<0.5	<0.05	<0.5	<0.5	<0.05	<0.5	<0.5	<0.05	<0.5
**Irreversible skin disease**	**<0.5**	**1.3**	**<0.5**	**1**	**1.3**	**<0.5**	**1**	**0.9**	**<0.5**
**Total skin disease (OSD)**	**3**	**8.5**	**<0.5**	**6**	**8.1**	**<0.5**	**3**	**2.9**	**<0.5**
Eye disease (OED):									
Visual impairment	<0.5	<0.05	<0.5	<0.5	<0.05	<0.5	<0.5	<0.05	<0.5
Blindness	<0.5	<0.05	<0.5	<0.5	<0.05	<0.5	<0.5	<0.05	<0.5
**Eye disease (OED)**	**<0.5**	**<0.05**	**<0.5**	**<0.5**	**<0.05**	**<0.5**	**<0.5**	**<0.05**	**<0.5**
**Total all manifestations**	**3**	**8.5**	**<0.5**	**6**	**8.1**	**<0.5**	**3**	**2.9**	**<0.5**

Note 1: Population at risk, number of cases (N) and DALYs are presented in thousands.

Note 2: Total skin disease is defined as the sum of the number of cases with palpable nodules, reversible and irreversible skin disease.

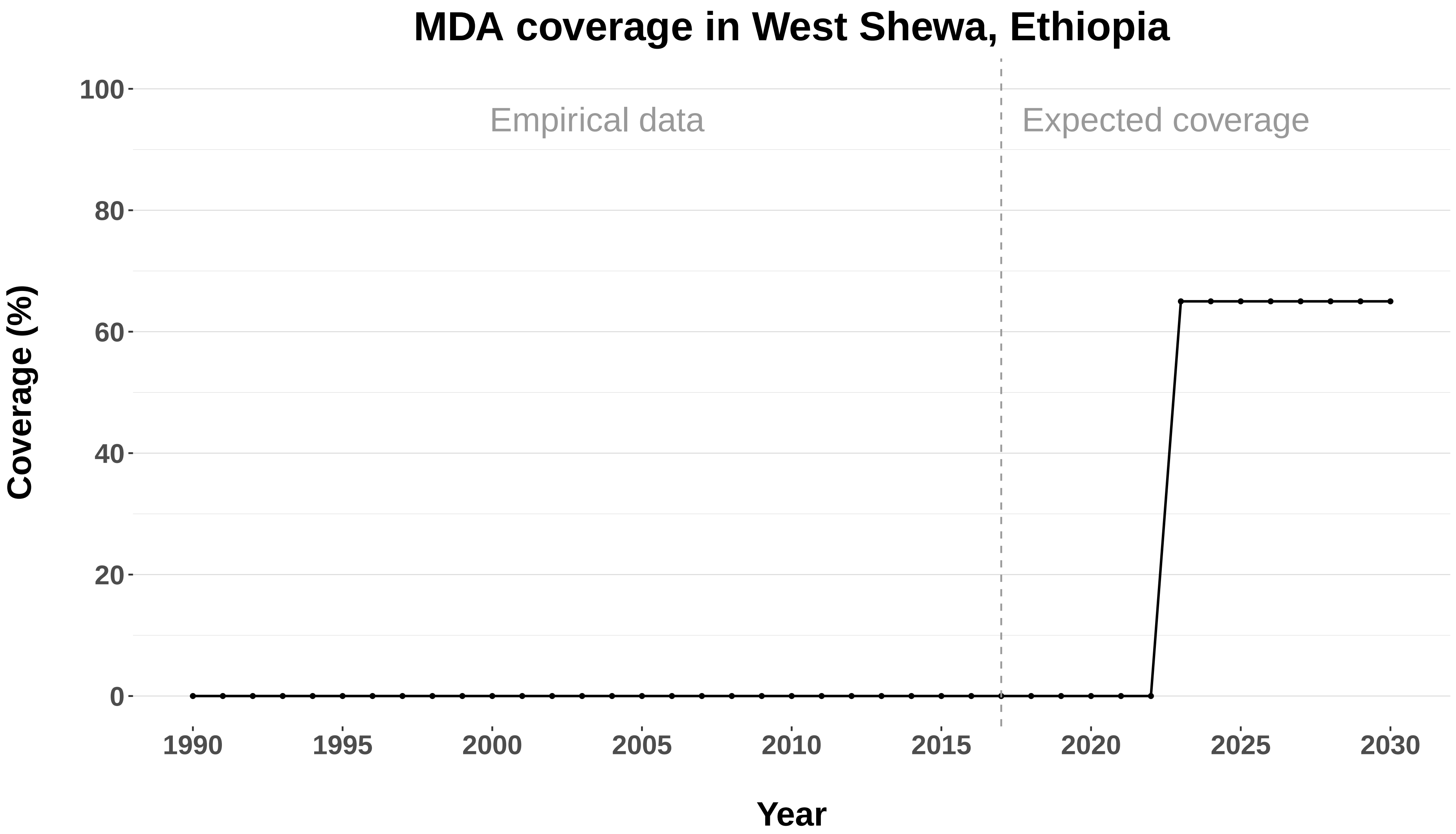
MDA coverage in West Shewa, Ethiopia
Empirical data
Expected coverage
Coverage (%)
100
80
60
40
20
0
1990
1995
2000
2005
2010
2015
2020
2025
2030
Year

**Project, country:**	**West Wellega, Ethiopia**
Bioclime:	
Endemicity level(s):	Hyperendemic
MDA start year:	2004
Mean MDA coverage per year:	72.7

	1990			2020			2030		
	N	%	DALYs	N	%	DALYs	N	%	DALYs
Total populaton at risk	590	100	_	1,332	100	_	1,747	100	_
**Infection:**									
Mf-positive	244	41.3	_	22	1.7	_	<0.5	<0.05	_
Mf-positive (age 5+)	243	41.3	_	22	1.7	_	<0.5	<0.05	_
Worm infected	297	50.3	_	149	11.2	_	9	0.5	_
**Clinical manifestations:**									
Palpable nodules	86	14.6	1	10	0.7	<0.5	<0.5	<0.05	<0.5
Reversible skin disease:									
Severe itch	41	6.9	7	3	0.2	<0.5	<0.5	<0.05	<0.5
Reactive skin disease	31	5.2	1	<0.5	<0.05	<0.5	<0.5	<0.05	<0.5
**Reversible skin disease**	**72**	**12.2**	**9**	**3**	**0.2**	**<0.5**	**<0.5**	**<0.05**	**<0.5**
Irreversible skin disease:									
Mild depigmentation	9	1.6	<0.5	10	0.8	<0.5	7	0.4	<0.5
Severe depigmentation	11	1.8	1	11	0.8	1	7	0.4	<0.5
Atrophy	<0.5	<0.05	<0.5	<0.5	<0.05	<0.5	<0.5	<0.05	<0.5
Hanging groin	<0.5	<0.05	<0.5	<0.5	<0.05	<0.5	<0.5	<0.05	<0.5
**Irreversible skin disease**	**20**	**3.4**	**1**	**21**	**1.6**	**1**	**14**	**0.8**	**1**
**Total skin disease (OSD)**	**178**	**30.2**	**11**	**33**	**2.5**	**1**	**15**	**0.8**	**1**
Eye disease (OED):									
Visual impairment	<0.5	0.1	<0.5	<0.5	<0.05	<0.5	<0.5	<0.05	<0.5
Blindness	<0.5	0.1	3	<0.5	<0.05	1	<0.5	<0.05	<0.5
**Eye disease (OED)**	**1**	**0.2**	**3**	**1**	**<0.05**	**1**	**<0.5**	**<0.05**	**<0.5**
**Total all manifestations**	**179**	**30.3**	**14**	**34**	**2.5**	**3**	**15**	**0.9**	**1**

Note 1: Population at risk, number of cases (N) and DALYs are presented in thousands.

Note 2: Total skin disease is defined as the sum of the number of cases with palpable nodules, reversible and irreversible skin disease.

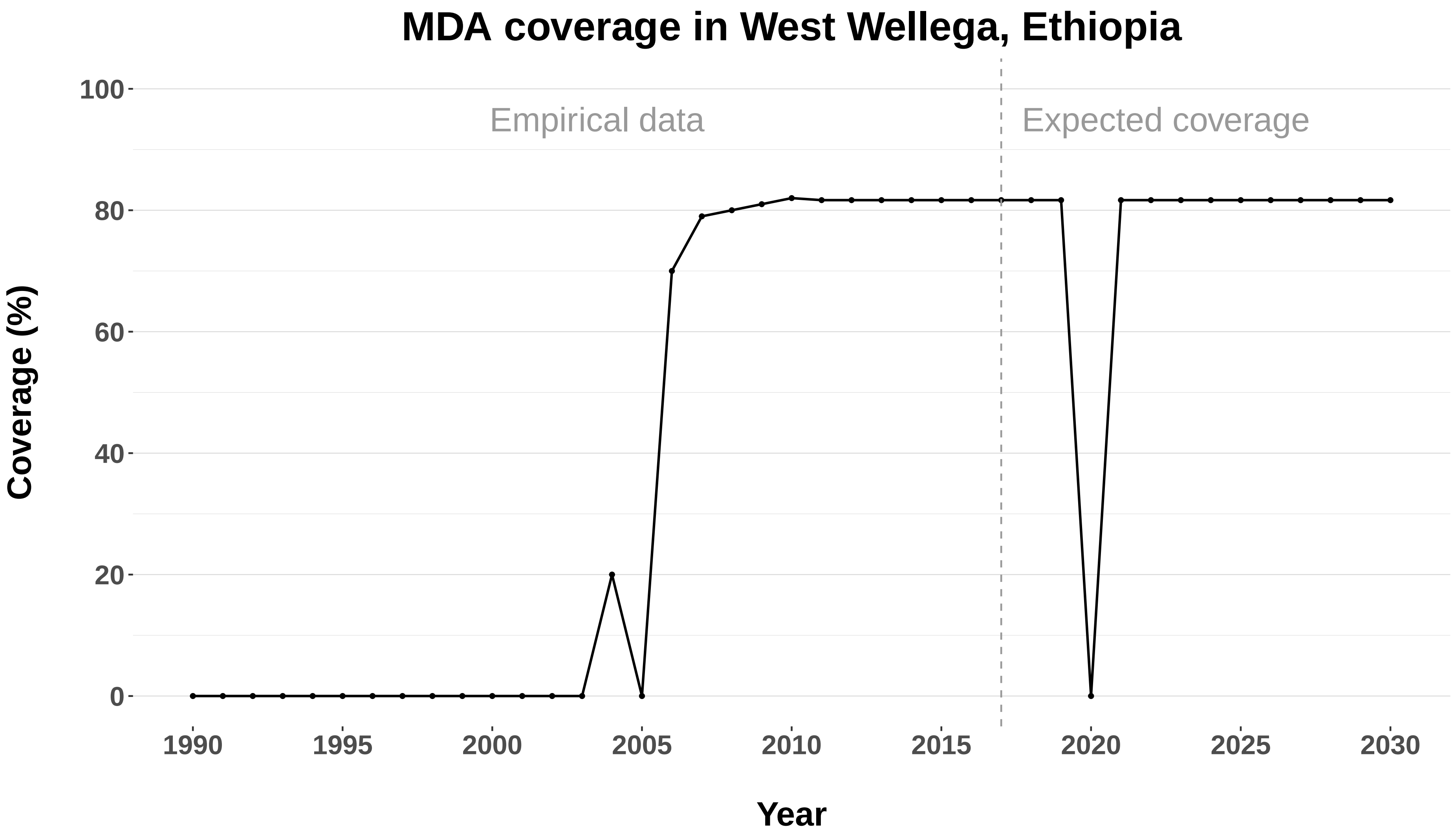
MDA coverage in West Wellega, Ethiopia
Empirical data
Expected coverage
Coverage (%)
100
80
60
40
20
0
1990
1995
2000
2005
2010
2015
2020
2025
2030
Year

**Project, country:**	**P5Gabon, Gabon**
Bioclime:	
Endemicity level(s):	Hypoendemic
MDA start year:	2025
Mean MDA coverage per year:	65.0

	1990			2020			2030		
	N	%	DALYs	N	%	DALYs	N	%	DALYs
Total populaton at risk	49	100	_	112	100	_	146	100	_
**Infection:**									
Mf-positive	11	21.6	_	22	20.0	_	17	11.9	_
Mf-positive (age 5+)	11	21.6	_	22	20.0	_	17	11.9	_
Worm infected	15	29.3	_	31	27.6	_	32	22.1	_
**Clinical manifestations:**									
Palpable nodules	1	2.5	<0.5	3	2.3	<0.5	2	1.5	<0.5
Reversible skin disease:									
Severe itch	1	2.6	<0.5	3	2.3	<0.5	1	0.9	<0.5
Reactive skin disease	1	1.7	<0.5	2	1.5	<0.5	<0.5	0.3	<0.5
**Reversible skin disease**	**2**	**4.3**	**<0.5**	**4**	**3.8**	**1**	**2**	**1.2**	**<0.5**
Irreversible skin disease:									
Mild depigmentation	<0.5	0.7	<0.5	1	0.7	<0.5	1	0.5	<0.5
Severe depigmentation	<0.5	0.6	<0.5	1	0.5	<0.5	1	0.4	<0.5
Atrophy	<0.5	<0.05	<0.5	<0.5	<0.05	<0.5	<0.5	<0.05	<0.5
Hanging groin	<0.5	<0.05	<0.5	<0.5	<0.05	<0.5	<0.5	<0.05	<0.5
**Irreversible skin disease**	**1**	**1.3**	**<0.5**	**1**	**1.2**	**<0.5**	**1**	**0.9**	**<0.5**
**Total skin disease (OSD)**	**4**	**8.1**	**<0.5**	**8**	**7.3**	**1**	**5**	**3.6**	**<0.5**
Eye disease (OED):									
Visual impairment	<0.5	<0.05	<0.5	<0.5	<0.05	<0.5	<0.5	<0.05	<0.5
Blindness	<0.5	<0.05	<0.5	<0.5	<0.05	<0.5	<0.5	<0.05	<0.5
**Eye disease (OED)**	**<0.5**	**<0.05**	**<0.5**	**<0.5**	**<0.05**	**<0.5**	**<0.5**	**<0.05**	**<0.5**
**Total all manifestations**	**4**	**8.1**	**<0.5**	**8**	**7.3**	**1**	**5**	**3.6**	**<0.5**

Note 1: Population at risk, number of cases (N) and DALYs are presented in thousands.

Note 2: Total skin disease is defined as the sum of the number of cases with palpable nodules, reversible and irreversible skin disease.

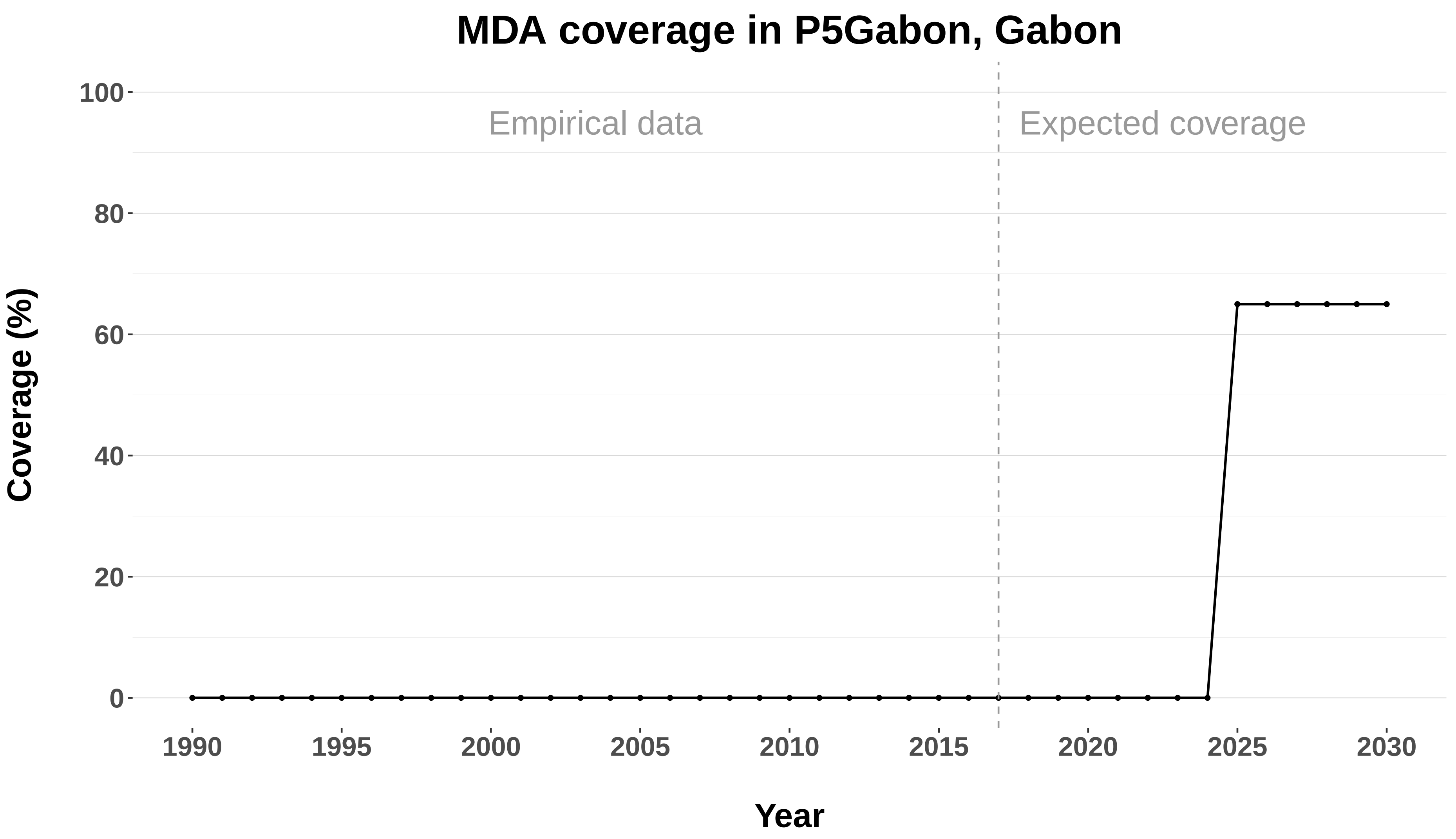
MDA coverage in P5Gabon, Gabon
Empirical data
Expected coverage
Coverage (%)
100
80
60
40
20
0
1990
1995
2000
2005
2010
2015
2020
2025
2030
Year

**Project, country:**	**All_Liberia, Liberia**
Bioclime:	
Endemicity level(s):	Mesoendemic
MDA start year:	2004
Mean MDA coverage per year:	66.3

	1990			2020			2030		
	N	%	DALYs	N	%	DALYs	N	%	DALYs
Total populaton at risk	881	100	_	1,987	100	_	2,606	100	_
**Infection:**									
Mf-positive	343	39.0	_	5	0.3	_	<0.5	<0.05	_
Mf-positive (age 5+)	343	38.9	_	5	0.3	_	<0.5	<0.05	_
Worm infected	455	51.7	_	54	2.7	_	2	0.1	_
**Clinical manifestations:**									
Palpable nodules	100	11.3	1	2	0.1	<0.5	<0.5	<0.05	<0.5
Reversible skin disease:									
Severe itch	43	4.9	8	<0.5	<0.05	<0.5	<0.5	<0.05	<0.5
Reactive skin disease	29	3.3	1	<0.5	<0.05	<0.5	<0.5	<0.05	<0.5
**Reversible skin disease**	**72**	**8.2**	**9**	**1**	**<0.05**	**<0.5**	**<0.5**	**<0.05**	**<0.5**
Irreversible skin disease:									
Mild depigmentation	10	1.1	<0.5	7	0.4	<0.5	5	0.2	<0.5
Severe depigmentation	9	1.0	1	6	0.3	<0.5	3	0.1	<0.5
Atrophy	<0.5	<0.05	<0.5	<0.5	<0.05	<0.5	<0.5	<0.05	<0.5
Hanging groin	<0.5	<0.05	<0.5	<0.5	<0.05	<0.5	<0.5	<0.05	<0.5
**Irreversible skin disease**	**19**	**2.1**	**1**	**13**	**0.7**	**<0.5**	**8**	**0.3**	**<0.5**
**Total skin disease (OSD)**	**191**	**21.7**	**11**	**15**	**0.8**	**1**	**8**	**0.3**	**<0.5**
Eye disease (OED):									
Visual impairment	<0.5	<0.05	<0.5	<0.5	<0.05	<0.5	<0.5	<0.05	<0.5
Blindness	<0.5	<0.05	<0.5	<0.5	<0.05	<0.5	<0.5	<0.05	<0.5
**Eye disease (OED)**	**<0.5**	**<0.05**	**<0.5**	**<0.5**	**<0.05**	**<0.5**	**<0.5**	**<0.05**	**<0.5**
**Total all manifestations**	**191**	**21.7**	**11**	**15**	**0.8**	**1**	**8**	**0.3**	**<0.5**

Note 1: Population at risk, number of cases (N) and DALYs are presented in thousands.

Note 2: Total skin disease is defined as the sum of the number of cases with palpable nodules, reversible and irreversible skin disease.

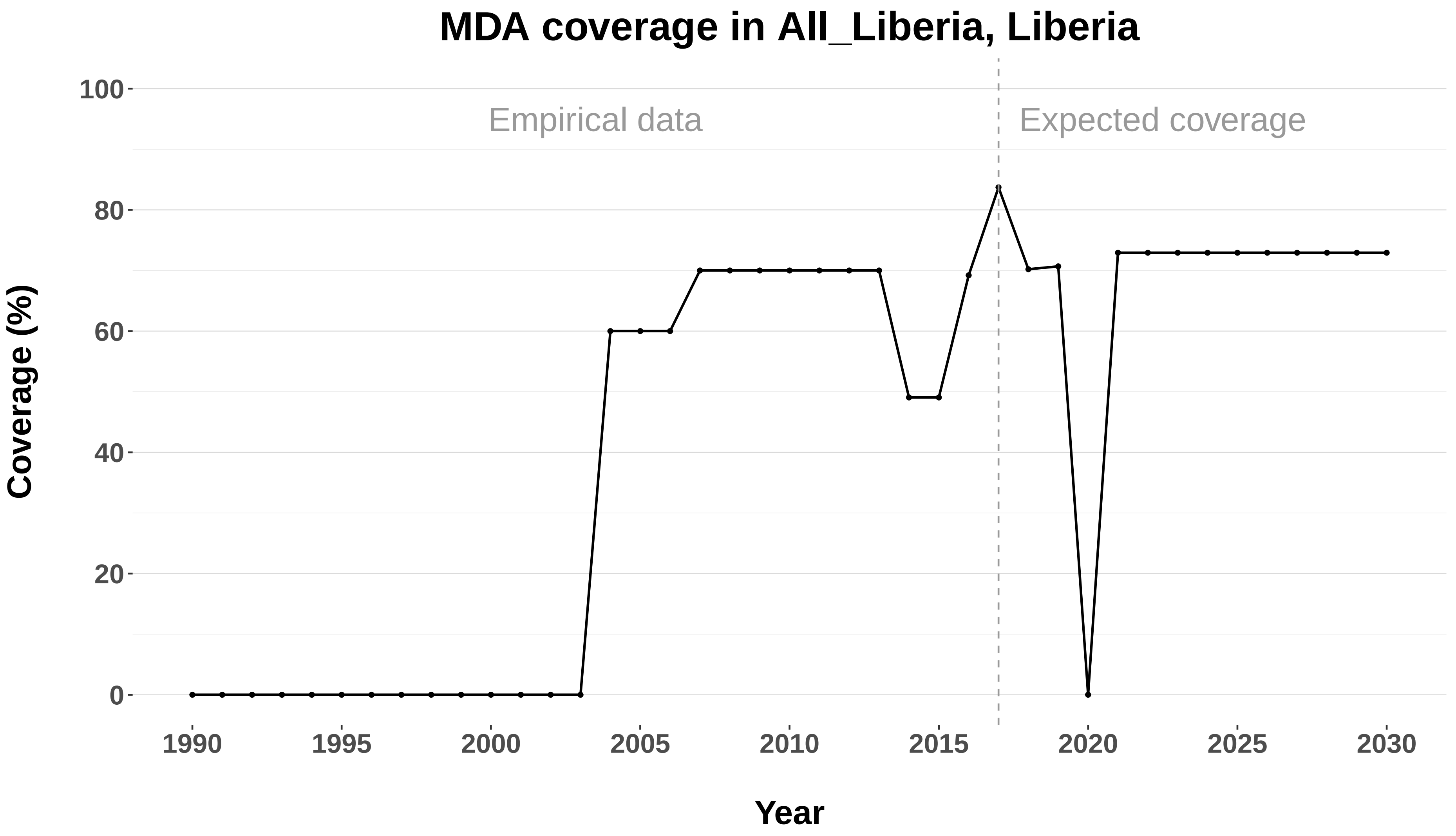
MDA coverage in All_Liberia, Liberia
Empirical data
Expected coverage
Coverage (%)
100
80
60
40
20
0
1990
1995
2000
2005
2010
2015
2020
2025
2030
Year

**Project, country:**	**Malawi Extension, Malawi**
Bioclime:	
Endemicity level(s):	Mesoendemic
MDA start year:	2000
Mean MDA coverage per year:	69.4

	1990			2020			2030		
	N	%	DALYs	N	%	DALYs	N	%	DALYs
Total populaton at risk	665	100	_	1,501	100	_	1,968	100	_
**Infection:**									
Mf-positive	136	20.4	_	1	0.1	_	<0.5	<0.05	_
Mf-positive (age 5+)	136	20.4	_	1	0.1	_	<0.5	<0.05	_
Worm infected	188	28.3	_	26	1.7	_	<0.5	<0.05	_
**Clinical manifestations:**									
Palpable nodules	15	2.2	<0.5	<0.5	<0.05	<0.5	<0.5	<0.05	<0.5
Reversible skin disease:									
Severe itch	16	2.4	3	<0.5	<0.05	<0.5	<0.5	<0.05	<0.5
Reactive skin disease	10	1.6	<0.5	<0.5	<0.05	<0.5	<0.5	<0.05	<0.5
**Reversible skin disease**	**26**	**3.9**	**3**	**<0.5**	**<0.05**	**<0.5**	**<0.5**	**<0.05**	**<0.5**
Irreversible skin disease:									
Mild depigmentation	4	0.7	<0.5	4	0.3	<0.5	3	0.1	<0.5
Severe depigmentation	4	0.6	<0.5	3	0.2	<0.5	2	0.1	<0.5
Atrophy	<0.5	<0.05	<0.5	<0.5	<0.05	<0.5	<0.5	<0.05	<0.5
Hanging groin	<0.5	<0.05	<0.5	<0.5	<0.05	<0.5	<0.5	<0.05	<0.5
**Irreversible skin disease**	**8**	**1.2**	**<0.5**	**7**	**0.5**	**<0.5**	**4**	**0.2**	**<0.5**
**Total skin disease (OSD)**	**49**	**7.4**	**4**	**7**	**0.5**	**<0.5**	**4**	**0.2**	**<0.5**
Eye disease (OED):									
Visual impairment	<0.5	<0.05	<0.5	<0.5	<0.05	<0.5	<0.5	<0.05	<0.5
Blindness	<0.5	<0.05	<0.5	<0.5	<0.05	<0.5	<0.5	<0.05	<0.5
**Eye disease (OED)**	**<0.5**	**<0.05**	**<0.5**	**<0.5**	**<0.05**	**<0.5**	**<0.5**	**<0.05**	**<0.5**
**Total all manifestations**	**49**	**7.4**	**4**	**7**	**0.5**	**<0.5**	**4**	**0.2**	**<0.5**

Note 1: Population at risk, number of cases (N) and DALYs are presented in thousands.

Note 2: Total skin disease is defined as the sum of the number of cases with palpable nodules, reversible and irreversible skin disease.

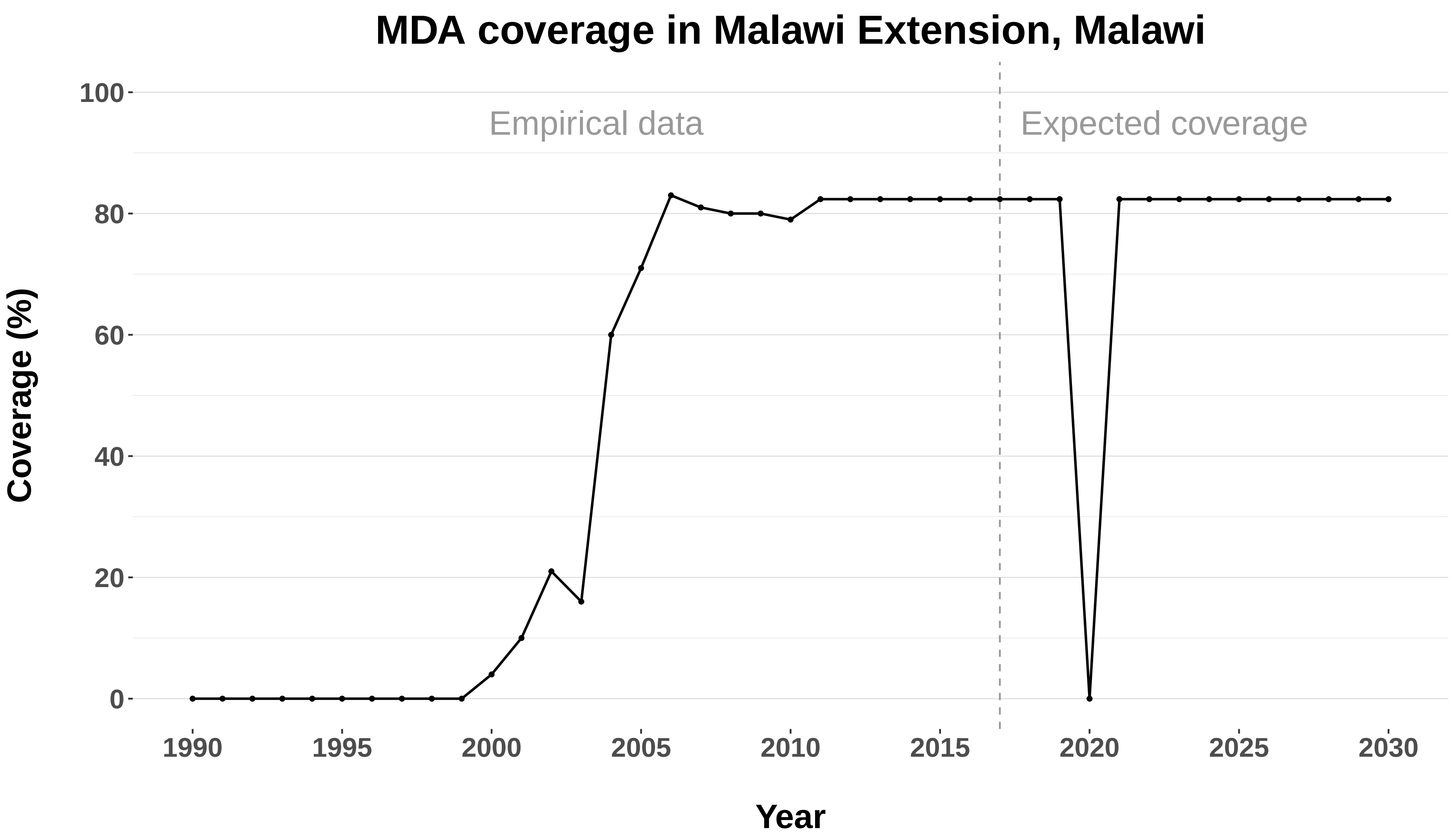

MDA coverage in Malawi Extension, Malawi
Empirical data
Expected coverage
Coverage (%)
100
80
60
40
20
0
1990
1995
2000
2005
2010
2015
2020
2025
2030
Year

**Project, country:**	**Thyolo Mwanza, Malawi**
Bioclime:	
Endemicity level(s):	Mesoendemic
MDA start year:	1997
Mean MDA coverage per year:	69.1

	1990			2020			2030		
	N	%	DALYs	N	%	DALYs	N	%	DALYs
Total populaton at risk	507	100	_	1,145	100	_	1,501	100	_
**Infection:**									
Mf-positive	137	27.0	_	<0.5	<0.05	_	<0.5	<0.05	_
Mf-positive (age 5+)	137	27.0	_	<0.5	<0.05	_	<0.5	<0.05	_
Worm infected	185	36.6	_	15	1.3	_	<0.5	<0.05	_
**Clinical manifestations:**									
Palpable nodules	26	5.2	<0.5	<0.5	<0.05	<0.5	<0.5	<0.05	<0.5
Reversible skin disease:									
Severe itch	17	3.3	3	<0.5	<0.05	<0.5	<0.5	<0.05	<0.5
Reactive skin disease	11	2.2	1	<0.5	<0.05	<0.5	<0.5	<0.05	<0.5
**Reversible skin disease**	**28**	**5.5**	**4**	**<0.5**	**<0.05**	**<0.5**	**<0.5**	**<0.05**	**<0.5**
Irreversible skin disease:									
Mild depigmentation	4	0.8	<0.5	3	0.3	<0.5	2	0.1	<0.5
Severe depigmentation	4	0.7	<0.5	2	0.2	<0.5	1	0.1	<0.5
Atrophy	<0.5	<0.05	<0.5	<0.5	<0.05	<0.5	<0.5	<0.05	<0.5
Hanging groin	<0.5	<0.05	<0.5	<0.5	<0.05	<0.5	<0.5	<0.05	<0.5
**Irreversible skin disease**	**8**	**1.6**	**<0.5**	**6**	**0.5**	**<0.5**	**3**	**0.2**	**<0.5**
**Total skin disease (OSD)**	**62**	**12.3**	**4**	**6**	**0.5**	**<0.5**	**3**	**0.2**	**<0.5**
Eye disease (OED):									
Visual impairment	<0.5	<0.05	<0.5	<0.5	<0.05	<0.5	<0.5	<0.05	<0.5
Blindness	<0.5	<0.05	<0.5	<0.5	<0.05	<0.5	<0.5	<0.05	<0.5
**Eye disease (OED)**	**<0.5**	**<0.05**	**<0.5**	**<0.5**	**<0.05**	**<0.5**	**<0.5**	**<0.05**	**<0.5**
**Total all manifestations**	**62**	**12.3**	**4**	**6**	**0.5**	**<0.5**	**3**	**0.2**	**<0.5**

Note 1: Population at risk, number of cases (N) and DALYs are presented in thousands.

Note 2: Total skin disease is defined as the sum of the number of cases with palpable nodules, reversible and irreversible skin disease.

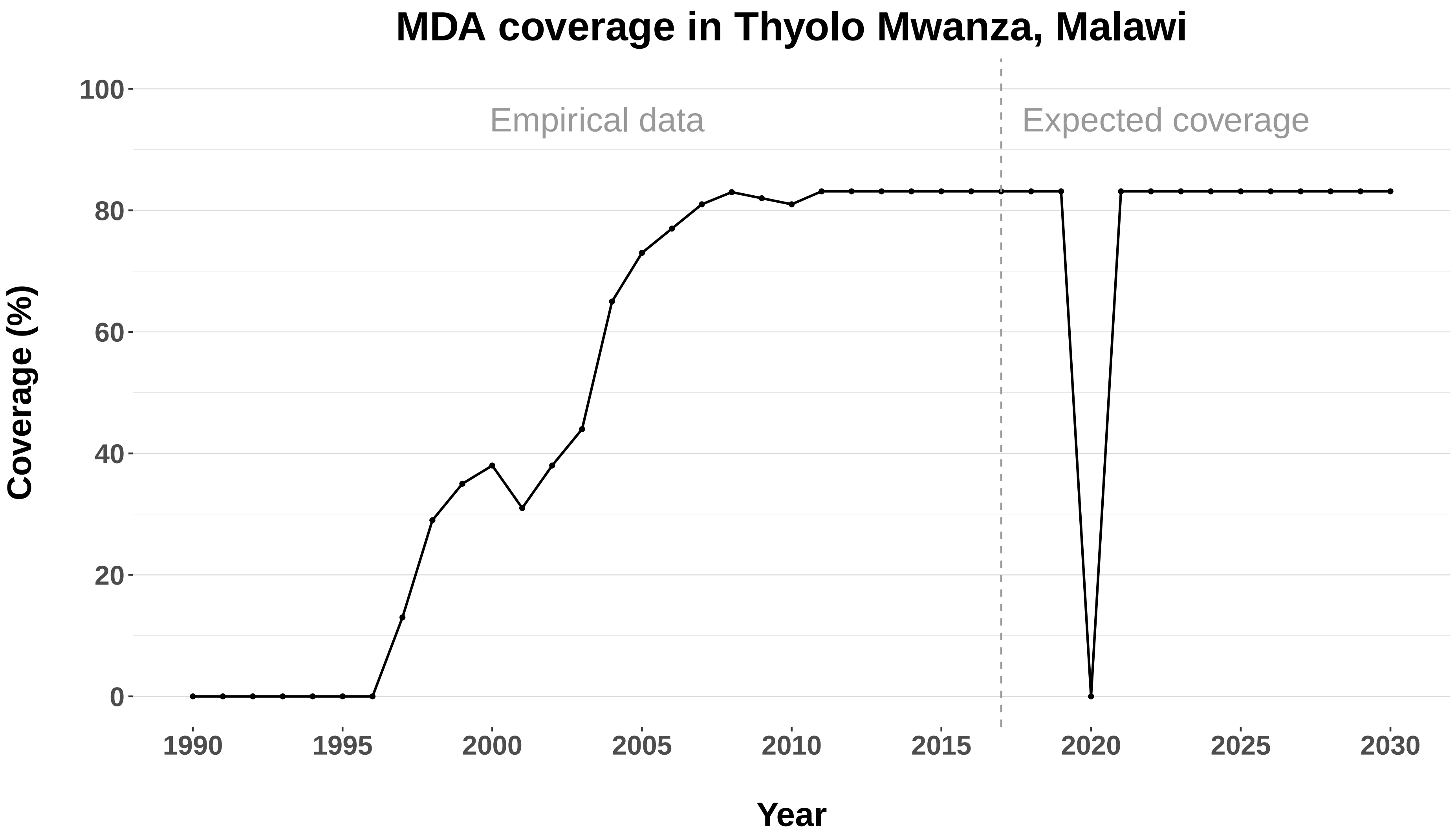
MDA coverage in Thyolo Mwanza, Malawi
Empirical data
Expected coverage
Coverage (%)
100
80
60
40
20
0
1990
1995
2000
2005
2010
2015
2020
2025
2030
Year

**Project, country:**	**P20Mozambique, Mozambique**
Bioclime:	
Endemicity level(s):	Mesoendemic
MDA start year:	2019
Mean MDA coverage per year:	64.2

	1990			2020			2030		
	N	%	DALYs	N	%	DALYs	N	%	DALYs
Total populaton at risk	9	100	_	21	100	_	27	100	_
**Infection:**									
Mf-positive	2	20.4	_	4	16.8	_	1	2.7	_
Mf-positive (age 5+)	2	20.3	_	4	16.8	_	1	2.7	_
Worm infected	3	28.2	_	5	25.7	_	3	10.6	_
**Clinical manifestations:**									
Palpable nodules	<0.5	2.2	<0.5	<0.5	1.9	<0.5	<0.5	0.3	<0.5
Reversible skin disease:									
Severe itch	<0.5	2.4	<0.5	<0.5	1.8	<0.5	<0.5	0.2	<0.5
Reactive skin disease	<0.5	1.6	<0.5	<0.5	1.0	<0.5	<0.5	<0.05	<0.5
**Reversible skin disease**	**<0.5**	**4.0**	**<0.5**	**1**	**2.8**	**<0.5**	**<0.5**	**0.2**	**<0.5**
Irreversible skin disease:									
Mild depigmentation	<0.5	0.7	<0.5	<0.5	0.6	<0.5	<0.5	0.4	<0.5
Severe depigmentation	<0.5	0.5	<0.5	<0.5	0.5	<0.5	<0.5	0.3	<0.5
Atrophy	<0.5	<0.05	<0.5	<0.5	<0.05	<0.5	<0.5	<0.05	<0.5
Hanging groin	<0.5	<0.05	<0.5	<0.5	<0.05	<0.5	<0.5	<0.05	<0.5
**Irreversible skin disease**	**<0.5**	**1.2**	**<0.5**	**<0.5**	**1.0**	**<0.5**	**<0.5**	**0.6**	**<0.5**
**Total skin disease (OSD)**	**1**	**7.4**	**<0.5**	**1**	**5.7**	**<0.5**	**<0.5**	**1.2**	**<0.5**
Eye disease (OED):									
Visual impairment	<0.5	<0.05	<0.5	<0.5	<0.05	<0.5	<0.5	<0.05	<0.5
Blindness	<0.5	<0.05	<0.5	<0.5	<0.05	<0.5	<0.5	<0.05	<0.5
**Eye disease (OED)**	**<0.5**	**<0.05**	**<0.5**	**<0.5**	**<0.05**	**<0.5**	**<0.5**	**<0.05**	**<0.5**
**Total all manifestations**	**1**	**7.4**	**<0.5**	**1**	**5.7**	**<0.5**	**<0.5**	**1.2**	**<0.5**

Note 1: Population at risk, number of cases (N) and DALYs are presented in thousands.

Note 2: Total skin disease is defined as the sum of the number of cases with palpable nodules, reversible and irreversible skin disease.

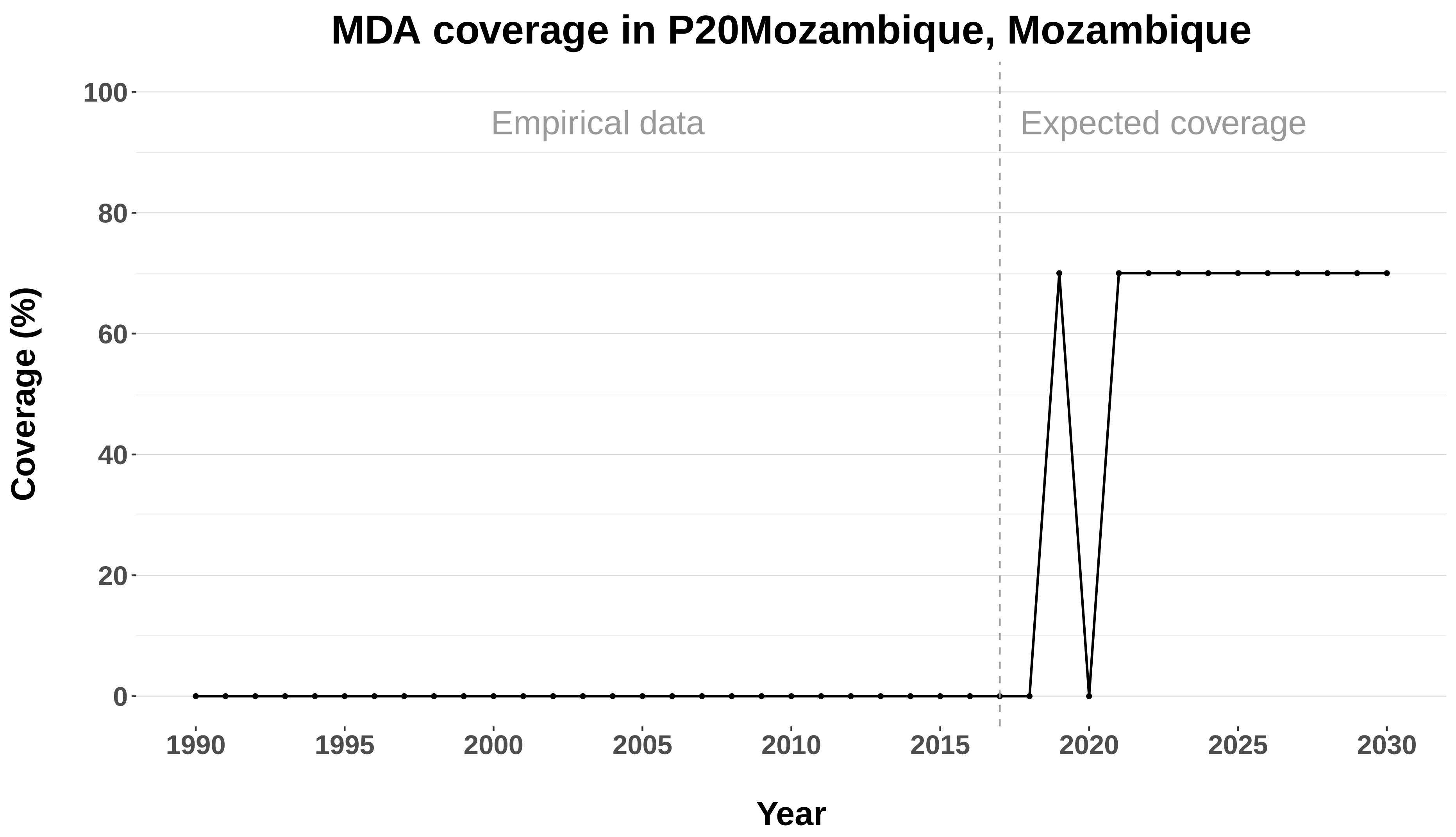
MDA coverage in P20Mozambique, Mozambique
Empirical data
Expected coverage
Coverage (%)
100
80
60
40
20
0
1990
1995
2000
2005
2010
2015
2020
2025
2030
Year

**Project, country:**	**P5Mozambique, Mozambique**
Bioclime:	
Endemicity level(s):	Hypoendemic
MDA start year:	2023
Mean MDA coverage per year:	65.0

	1990			2020			2030		
	N	%	DALYs	N	%	DALYs	N	%	DALYs
Total populaton at risk	27	100	_	61	100	_	80	100	_
**Infection:**									
Mf-positive	5	19.9	_	11	17.3	_	5	6.8	_
Mf-positive (age 5+)	5	19.9	_	11	17.3	_	5	6.8	_
Worm infected	7	27.5	_	15	24.7	_	13	16.5	_
**Clinical manifestations:**									
Palpable nodules	1	2.0	<0.5	1	1.7	<0.5	1	0.8	<0.5
Reversible skin disease:									
Severe itch	1	2.3	<0.5	1	2.0	<0.5	<0.5	0.5	<0.5
Reactive skin disease	<0.5	1.5	<0.5	1	1.3	<0.5	<0.5	0.1	<0.5
**Reversible skin disease**	**1**	**3.9**	**<0.5**	**2**	**3.3**	**<0.5**	**<0.5**	**0.6**	**<0.5**
Irreversible skin disease:									
Mild depigmentation	<0.5	0.7	<0.5	<0.5	0.5	<0.5	<0.5	0.4	<0.5
Severe depigmentation	<0.5	0.6	<0.5	<0.5	0.5	<0.5	<0.5	0.3	<0.5
Atrophy	<0.5	<0.05	<0.5	<0.5	<0.05	<0.5	<0.5	<0.05	<0.5
Hanging groin	<0.5	<0.05	<0.5	<0.5	<0.05	<0.5	<0.5	<0.05	<0.5
**Irreversible skin disease**	**<0.5**	**1.2**	**<0.5**	**1**	**1.0**	**<0.5**	**1**	**0.7**	**<0.5**
**Total skin disease (OSD)**	**2**	**7.1**	**<0.5**	**4**	**6.0**	**<0.5**	**2**	**2.0**	**<0.5**
Eye disease (OED):									
Visual impairment	<0.5	<0.05	<0.5	<0.5	<0.05	<0.5	<0.5	<0.05	<0.5
Blindness	<0.5	<0.05	<0.5	<0.5	<0.05	<0.5	<0.5	<0.05	<0.5
**Eye disease (OED)**	**<0.5**	**<0.05**	**<0.5**	**<0.5**	**<0.05**	**<0.5**	**<0.5**	**<0.05**	**<0.5**
**Total all manifestations**	**2**	**7.1**	**<0.5**	**4**	**6.0**	**<0.5**	**2**	**2.1**	**<0.5**

Note 1: Population at risk, number of cases (N) and DALYs are presented in thousands.

Note 2: Total skin disease is defined as the sum of the number of cases with palpable nodules, reversible and irreversible skin disease.

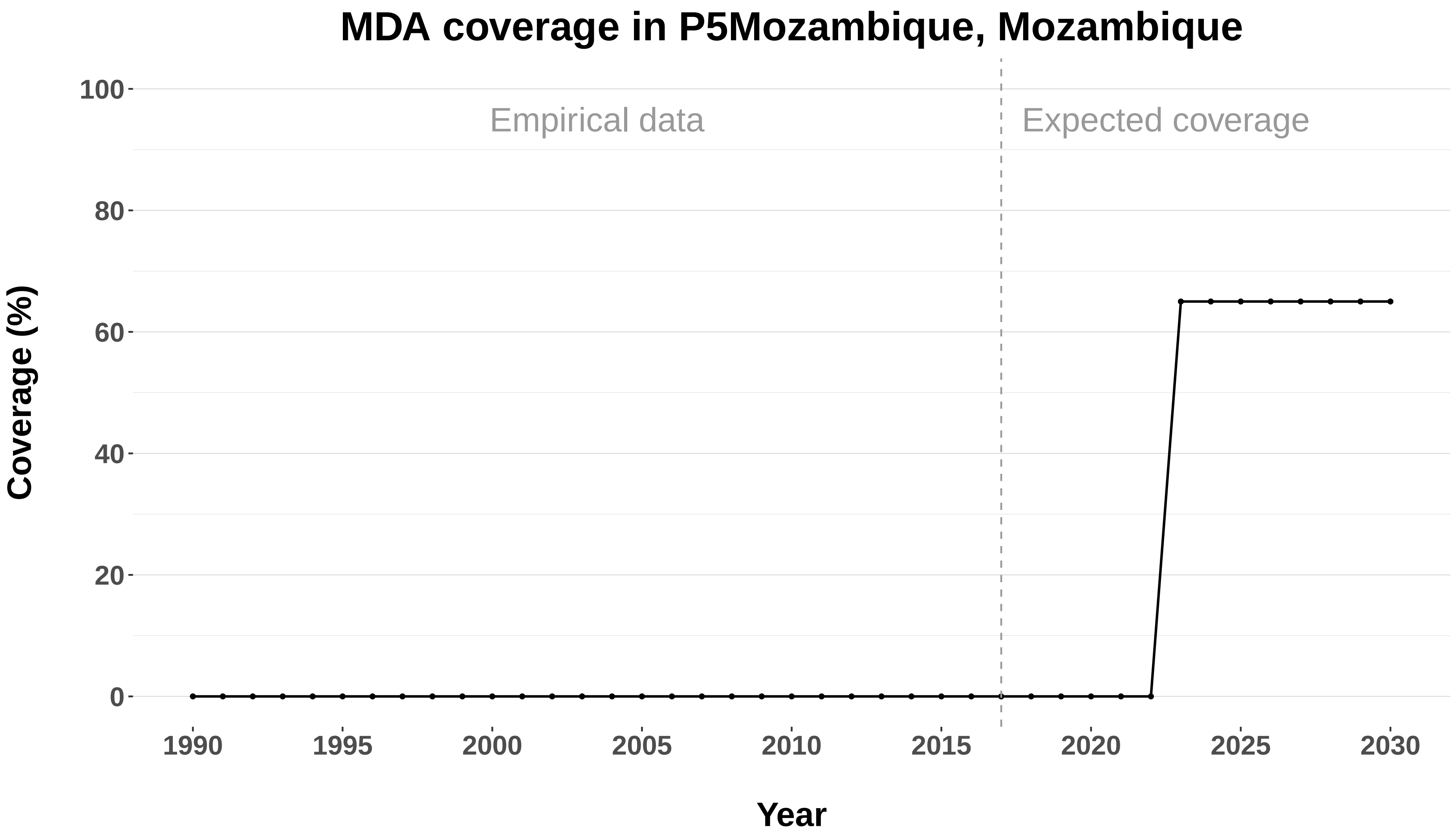
MDA coverage in P5Mozambique, Mozambique
Empirical data
Expected coverage
Coverage (%)
100
80
60
40
20
0
1990
1995
2000
2005
2010
2015
2020
2025
2030
Year

**Project, country:**	**Adamawa, Nigeria**
Bioclime:	
Endemicity level(s):	Hyperendemic
MDA start year:	1999
Mean MDA coverage per year:	72.9

	1990			2020			2030		
	N	%	DALYs	N	%	DALYs	N	%	DALYs
Total populaton at risk	1,019	100	_	2,298	100	_	3,015	100	_
**Infection:**									
Mf-positive	241	23.7	_	<0.5	<0.05	_	<0.5	<0.05	_
Mf-positive (age 5+)	241	23.7	_	<0.5	<0.05	_	<0.5	<0.05	_
Worm infected	323	31.7	_	22	0.9	_	<0.5	<0.05	_
**Clinical manifestations:**									
Palpable nodules	34	3.4	<0.5	<0.5	<0.05	<0.5	<0.5	<0.05	<0.5
Reversible skin disease:									
Severe itch	29	2.9	5	<0.5	<0.05	<0.5	<0.5	<0.05	<0.5
Reactive skin disease	20	2.0	1	<0.5	<0.05	<0.5	<0.5	<0.05	<0.5
**Reversible skin disease**	**49**	**4.9**	**6**	**<0.5**	**<0.05**	**<0.5**	**<0.5**	**<0.05**	**<0.5**
Irreversible skin disease:									
Mild depigmentation	7	0.7	<0.5	6	0.3	<0.5	4	0.1	<0.5
Severe depigmentation	7	0.7	<0.5	4	0.2	<0.5	3	0.1	<0.5
Atrophy	<0.5	<0.05	<0.5	<0.5	<0.05	<0.5	<0.5	<0.05	<0.5
Hanging groin	<0.5	<0.05	<0.5	<0.5	<0.05	<0.5	<0.5	<0.05	<0.5
**Irreversible skin disease**	**14**	**1.4**	**1**	**11**	**0.5**	**<0.5**	**6**	**0.2**	**<0.5**
**Total skin disease (OSD)**	**98**	**9.6**	**7**	**11**	**0.5**	**<0.5**	**6**	**0.2**	**<0.5**
Eye disease (OED):									
Visual impairment	13	1.3	<0.5	9	0.4	<0.5	5	0.2	<0.5
Blindness	4	0.4	27	1	0.1	8	1	<0.05	4
**Eye disease (OED)**	**17**	**1.7**	**27**	**10**	**0.4**	**8**	**5**	**0.2**	**4**
**Total all manifestations**	**115**	**11.3**	**35**	**21**	**0.9**	**9**	**12**	**0.4**	**4**

Note 1: Population at risk, number of cases (N) and DALYs are presented in thousands.

Note 2: Total skin disease is defined as the sum of the number of cases with palpable nodules, reversible and irreversible skin disease.

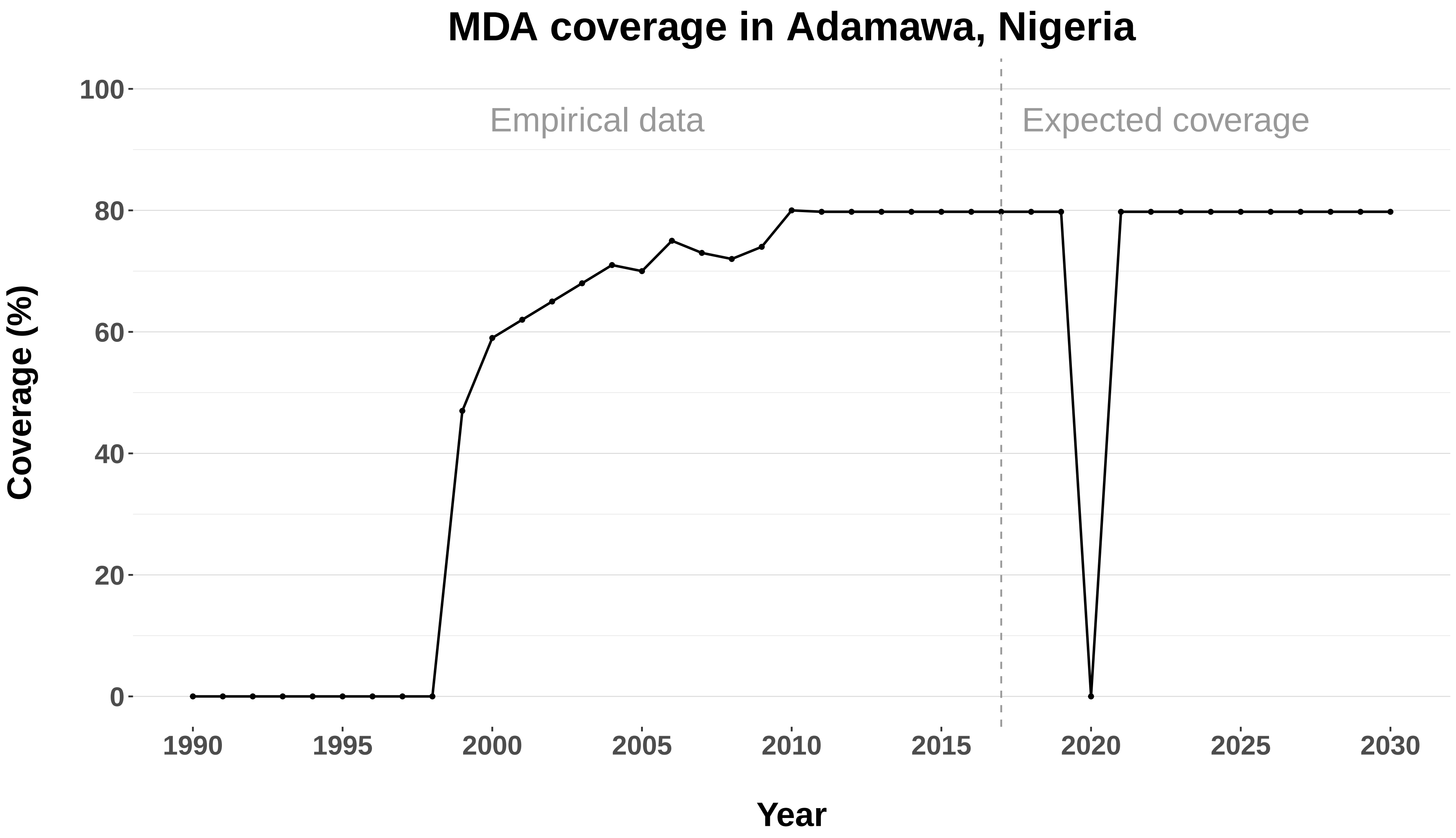
MDA coverage in Adamawa, Nigeria
Empirical data
Expected coverage
Coverage (%)
100
80
60
40
20
0
1990
1995
2000
2005
2010
2015
2020
2025
2030
Year

**Project, country:**	**Akwa Ibom, Nigeria**
Bioclime:	
Endemicity level(s):	Mesoendemic
MDA start year:	2004
Mean MDA coverage per year:	74.1

	1990			2020			2030		
	N	%	DALYs	N	%	DALYs	N	%	DALYs
Total populaton at risk	17	100	_	39	100	_	52	100	_
**Infection:**									
Mf-positive	4	21.6	_	<0.5	0.2	_	<0.5	<0.05	_
Mf-positive (age 5+)	4	21.6	_	<0.5	0.2	_	<0.5	<0.05	_
Worm infected	5	29.3	_	2	4.1	_	<0.5	0.1	_
**Clinical manifestations:**									
Palpable nodules	<0.5	2.5	<0.5	<0.5	0.1	<0.5	<0.5	<0.05	<0.5
Reversible skin disease:									
Severe itch	<0.5	2.6	<0.5	<0.5	<0.05	<0.5	<0.5	<0.05	<0.5
Reactive skin disease	<0.5	1.7	<0.5	<0.5	<0.05	<0.5	<0.5	<0.05	<0.5
**Reversible skin disease**	**1**	**4.3**	**<0.5**	**<0.5**	**<0.05**	**<0.5**	**<0.5**	**<0.05**	**<0.5**
Irreversible skin disease:									
Mild depigmentation	<0.5	0.7	<0.5	<0.5	0.3	<0.5	<0.5	0.2	<0.5
Severe depigmentation	<0.5	0.6	<0.5	<0.5	0.2	<0.5	<0.5	0.1	<0.5
Atrophy	<0.5	<0.05	<0.5	<0.5	<0.05	<0.5	<0.5	<0.05	<0.5
Hanging groin	<0.5	<0.05	<0.5	<0.5	<0.05	<0.5	<0.5	<0.05	<0.5
**Irreversible skin disease**	**<0.5**	**1.3**	**<0.5**	**<0.5**	**0.6**	**<0.5**	**<0.5**	**0.3**	**<0.5**
**Total skin disease (OSD)**	**1**	**8.1**	**<0.5**	**<0.5**	**0.6**	**<0.5**	**<0.5**	**0.3**	**<0.5**
Eye disease (OED):									
Visual impairment	<0.5	<0.05	<0.5	<0.5	<0.05	<0.5	<0.5	<0.05	<0.5
Blindness	<0.5	<0.05	<0.5	<0.5	<0.05	<0.5	<0.5	<0.05	<0.5
**Eye disease (OED)**	**<0.5**	**<0.05**	**<0.5**	**<0.5**	**<0.05**	**<0.5**	**<0.5**	**<0.05**	**<0.5**
**Total all manifestations**	**1**	**8.1**	**<0.5**	**<0.5**	**0.6**	**<0.5**	**<0.5**	**0.3**	**<0.5**

Note 1: Population at risk, number of cases (N) and DALYs are presented in thousands.

Note 2: Total skin disease is defined as the sum of the number of cases with palpable nodules, reversible and irreversible skin disease.

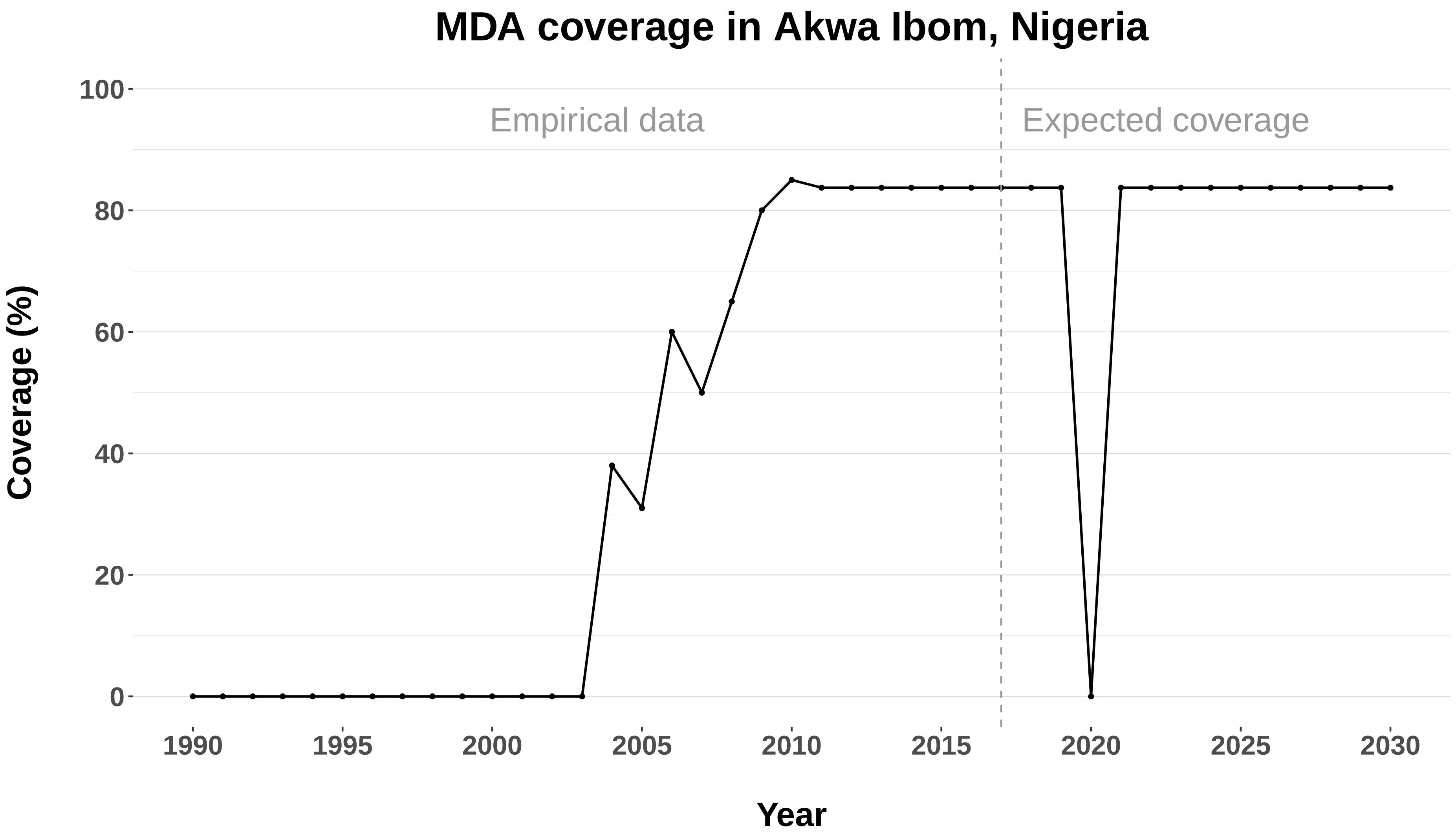
MDA coverage in Akwa Ibom, Nigeria
Empirical data
Expected coverage
Coverage (%)
100
80
60
40
20
0
1990
1995
2000
2005
2010
2015
2020
2025
2030
Year

**Project, country:**	**Bauchi, Nigeria**
Bioclime:	
Endemicity level(s):	Mesoendemic
MDA start year:	1999
Mean MDA coverage per year:	63.2

	1990			2020			2030		
	N	%	DALYs	N	%	DALYs	N	%	DALYs
Total populaton at risk	1,044	100	_	2,355	100	_	3,089	100	_
**Infection:**									
Mf-positive	225	21.5	_	5	0.2	_	<0.5	<0.05	_
Mf-positive (age 5+)	225	21.5	_	5	0.2	_	<0.5	<0.05	_
Worm infected	307	29.4	_	75	3.2	_	2	0.1	_
**Clinical manifestations:**									
Palpable nodules	26	2.5	<0.5	1	<0.05	<0.5	<0.5	<0.05	<0.5
Reversible skin disease:									
Severe itch	27	2.6	5	1	<0.05	<0.5	<0.5	<0.05	<0.5
Reactive skin disease	18	1.7	1	<0.5	<0.05	<0.5	<0.5	<0.05	<0.5
**Reversible skin disease**	**45**	**4.3**	**6**	**1**	**<0.05**	**<0.5**	**<0.5**	**<0.05**	**<0.5**
Irreversible skin disease:									
Mild depigmentation	8	0.7	<0.5	7	0.3	<0.5	4	0.1	<0.5
Severe depigmentation	6	0.6	<0.5	5	0.2	<0.5	3	0.1	<0.5
Atrophy	<0.5	<0.05	<0.5	<0.5	<0.05	<0.5	<0.5	<0.05	<0.5
Hanging groin	<0.5	<0.05	<0.5	<0.5	<0.05	<0.5	<0.5	<0.05	<0.5
**Irreversible skin disease**	**14**	**1.3**	**<0.5**	**11**	**0.5**	**<0.5**	**7**	**0.2**	**<0.5**
**Total skin disease (OSD)**	**85**	**8.1**	**7**	**13**	**0.6**	**<0.5**	**7**	**0.2**	**<0.5**
Eye disease (OED):									
Visual impairment	12	1.2	<0.5	10	0.4	<0.5	6	0.2	<0.5
Blindness	4	0.4	26	2	0.1	11	1	<0.05	5
**Eye disease (OED)**	**16**	**1.5**	**26**	**12**	**0.5**	**11**	**7**	**0.2**	**5**
**Total all manifestations**	**101**	**9.6**	**33**	**25**	**1.1**	**12**	**14**	**0.5**	**5**

Note 1: Population at risk, number of cases (N) and DALYs are presented in thousands.

Note 2: Total skin disease is defined as the sum of the number of cases with palpable nodules, reversible and irreversible skin disease.

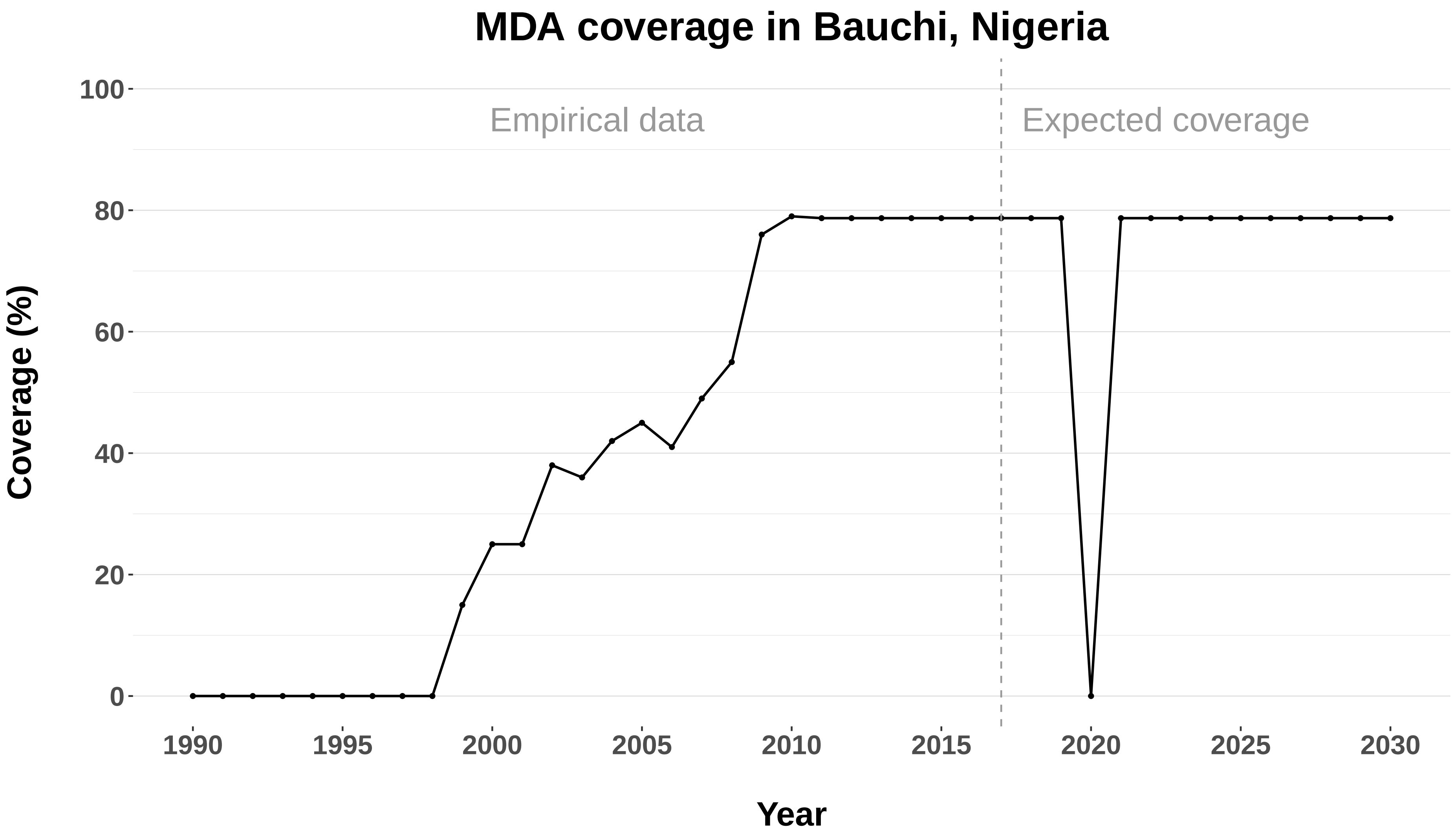
MDA coverage in Bauchi, Nigeria
Empirical data
Expected coverage
Coverage (%)
0
20
40
60
80
100
1990
1995
2000
2005
2010
2015
2020
2025
2030
Year

**Project, country:**	**Benue, Nigeria**
Bioclime:	
Endemicity level(s):	Hyperendemic
MDA start year:	1999
Mean MDA coverage per year:	62.9

	1990			2020			2030		
	N	%	DALYs	N	%	DALYs	N	%	DALYs
Total populaton at risk	2,058	100	_	4,644	100	_	6,092	100	_
**Infection:**									
Mf-positive	783	38.0	_	41	0.9	_	1	<0.05	_
Mf-positive (age 5+)	782	38.0	_	41	0.9	_	1	<0.05	_
Worm infected	984	47.8	_	340	7.3	_	20	0.3	_
**Clinical manifestations:**									
Palpable nodules	239	11.6	3	16	0.3	<0.5	<0.5	<0.05	<0.5
Reversible skin disease:									
Severe itch	118	5.7	21	4	0.1	1	<0.5	<0.05	<0.5
Reactive skin disease	85	4.1	4	<0.5	<0.05	<0.5	<0.5	<0.05	<0.5
**Reversible skin disease**	**203**	**9.9**	**25**	**4**	**0.1**	**1**	**<0.5**	**<0.05**	**<0.5**
Irreversible skin disease:									
Mild depigmentation	27	1.3	<0.5	26	0.6	<0.5	18	0.3	<0.5
Severe depigmentation	28	1.4	2	24	0.5	2	15	0.3	1
Atrophy	<0.5	<0.05	<0.5	<0.5	<0.05	<0.5	<0.5	<0.05	<0.5
Hanging groin	<0.5	<0.05	<0.5	<0.5	<0.05	<0.5	<0.5	<0.05	<0.5
**Irreversible skin disease**	**56**	**2.7**	**2**	**51**	**1.1**	**2**	**34**	**0.6**	**1**
**Total skin disease (OSD)**	**498**	**24.2**	**30**	**71**	**1.5**	**3**	**34**	**0.6**	**1**
Eye disease (OED):									
Visual impairment	1	<0.05	<0.5	1	<0.05	<0.5	<0.5	<0.05	<0.5
Blindness	1	<0.05	5	<0.5	<0.05	2	<0.5	<0.05	1
**Eye disease (OED)**	**2**	**0.1**	**5**	**1**	**<0.05**	**2**	**<0.5**	**<0.05**	**1**
**Total all manifestations**	**500**	**24.3**	**36**	**72**	**1.5**	**5**	**34**	**0.6**	**2**

Note 1: Population at risk, number of cases (N) and DALYs are presented in thousands.

Note 2: Total skin disease is defined as the sum of the number of cases with palpable nodules, reversible and irreversible skin disease.

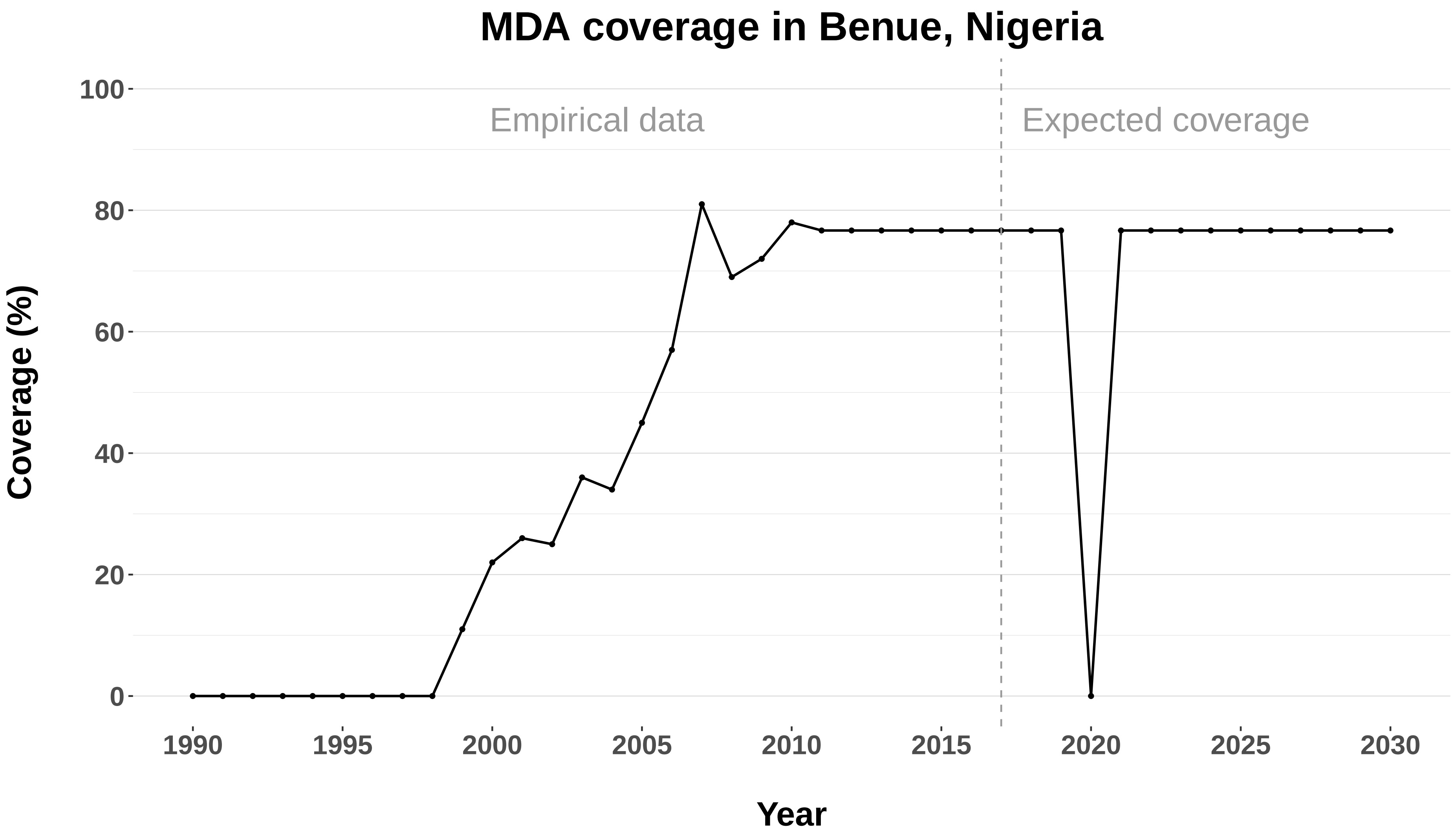
MDA coverage in Benue, Nigeria
Empirical data
Expected coverage
Coverage (%)
100
80
60
40
20
0
1990
1995
2000
2005
2010
2015
2020
2025
2030
Year

**Project, country:**	**Borno, Nigeria**
Bioclime:	
Endemicity level(s):	Mesoendemic
MDA start year:	1999
Mean MDA coverage per year:	72.1

	1990			2020			2030		
	N	%	DALYs	N	%	DALYs	N	%	DALYs
Total populaton at risk	820	100	_	1,851	100	_	2,428	100	_
**Infection:**									
Mf-positive	160	19.5	_	<0.5	<0.05	_	<0.5	<0.05	_
Mf-positive (age 5+)	160	19.5	_	<0.5	<0.05	_	<0.5	<0.05	_
Worm infected	229	27.9	_	17	0.9	_	<0.5	<0.05	_
**Clinical manifestations:**									
Palpable nodules	18	2.2	<0.5	<0.5	<0.05	<0.5	<0.5	<0.05	<0.5
Reversible skin disease:									
Severe itch	18	2.2	3	<0.5	<0.05	<0.5	<0.5	<0.05	<0.5
Reactive skin disease	11	1.4	1	<0.5	<0.05	<0.5	<0.5	<0.05	<0.5
**Reversible skin disease**	**29**	**3.5**	**4**	**<0.5**	**<0.05**	**<0.5**	**<0.5**	**<0.05**	**<0.5**
Irreversible skin disease:									
Mild depigmentation	5	0.6	<0.5	3	0.2	<0.5	2	0.1	<0.5
Severe depigmentation	4	0.5	<0.5	2	0.1	<0.5	1	0.1	<0.5
Atrophy	<0.5	<0.05	<0.5	<0.5	<0.05	<0.5	<0.5	<0.05	<0.5
Hanging groin	<0.5	<0.05	<0.5	<0.5	<0.05	<0.5	<0.5	<0.05	<0.5
**Irreversible skin disease**	**9**	**1.1**	**<0.5**	**5**	**0.3**	**<0.5**	**3**	**0.1**	**<0.5**
**Total skin disease (OSD)**	**56**	**6.8**	**4**	**5**	**0.3**	**<0.5**	**3**	**0.1**	**<0.5**
Eye disease (OED):									
Visual impairment	8	1.0	<0.5	5	0.3	<0.5	3	0.1	<0.5
Blindness	2	0.3	15	1	<0.05	4	<0.5	<0.05	1
**Eye disease (OED)**	**10**	**1.3**	**16**	**6**	**0.3**	**4**	**3**	**0.1**	**1**
**Total all manifestations**	**66**	**8.1**	**20**	**11**	**0.6**	**4**	**6**	**0.3**	**1**

Note 1: Population at risk, number of cases (N) and DALYs are presented in thousands.

Note 2: Total skin disease is defined as the sum of the number of cases with palpable nodules, reversible and irreversible skin disease.

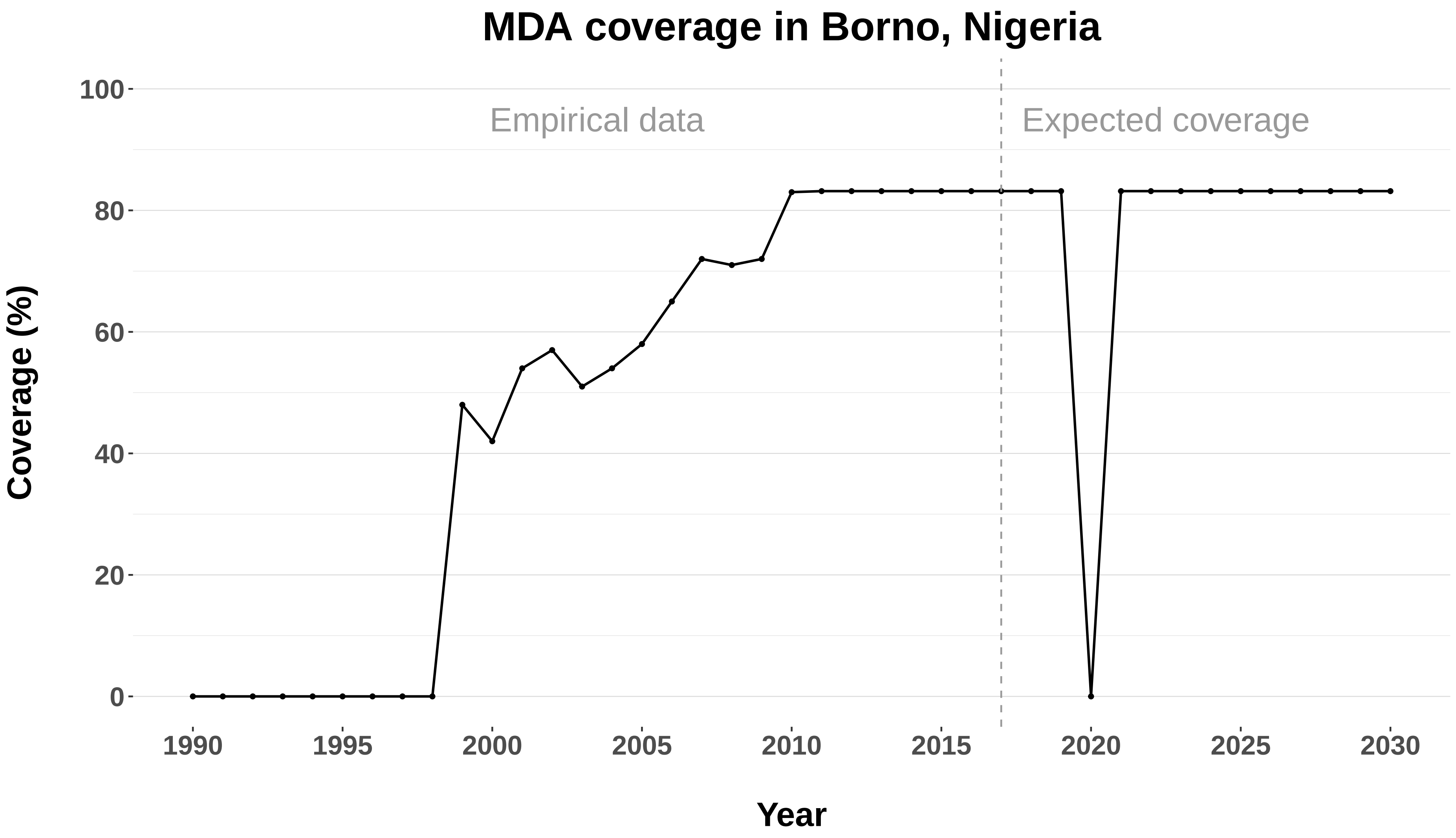

MDA coverage in Borno, Nigeria
Empirical data
Expected coverage
Coverage (%)
100
80
60
40
20
0
1990
1995
2000
2005
2010
2015
2020
2025
2030
Year

**Project, country:**	**Cross River, Nigeria**
Bioclime:	
Endemicity level(s):	Hyperendemic
MDA start year:	1998
Mean MDA coverage per year:	75.1

	1990			2020			2030		
	N	%	DALYs	N	%	DALYs	N	%	DALYs
Total populaton at risk	744	100	_	1,679	100	_	2,202	100	_
**Infection:**									
Mf-positive	222	29.8	_	1	<0.05	_	<0.5	<0.05	_
Mf-positive (age 5+)	221	29.8	_	1	<0.05	_	<0.5	<0.05	_
Worm infected	285	38.4	_	17	1.0	_	<0.5	<0.05	_
**Clinical manifestations:**									
Palpable nodules	55	7.4	1	<0.5	<0.05	<0.5	<0.5	<0.05	<0.5
Reversible skin disease:									
Severe itch	32	4.3	6	<0.5	<0.05	<0.5	<0.5	<0.05	<0.5
Reactive skin disease	23	3.1	1	<0.5	<0.05	<0.5	<0.5	<0.05	<0.5
**Reversible skin disease**	**55**	**7.4**	**7**	**<0.5**	**<0.05**	**<0.5**	**<0.5**	**<0.05**	**<0.5**
Irreversible skin disease:									
Mild depigmentation	8	1.0	<0.5	5	0.3	<0.5	4	0.2	<0.5
Severe depigmentation	8	1.1	1	5	0.3	<0.5	3	0.1	<0.5
Atrophy	<0.5	<0.05	<0.5	<0.5	<0.05	<0.5	<0.5	<0.05	<0.5
Hanging groin	<0.5	<0.05	<0.5	<0.5	<0.05	<0.5	<0.5	<0.05	<0.5
**Irreversible skin disease**	**16**	**2.1**	**1**	**10**	**0.6**	**<0.5**	**6**	**0.3**	**<0.5**
**Total skin disease (OSD)**	**126**	**17.0**	**8**	**11**	**0.7**	**<0.5**	**6**	**0.3**	**<0.5**
Eye disease (OED):									
Visual impairment	<0.5	<0.05	<0.5	<0.5	<0.05	<0.5	<0.5	<0.05	<0.5
Blindness	<0.5	<0.05	2	<0.5	<0.05	<0.5	<0.5	<0.05	<0.5
**Eye disease (OED)**	**1**	**0.1**	**2**	**<0.5**	**<0.05**	**<0.5**	**<0.5**	**<0.05**	**<0.5**
**Total all manifestations**	**127**	**17.1**	**10**	**11**	**0.7**	**1**	**7**	**0.3**	**<0.5**

Note 1: Population at risk, number of cases (N) and DALYs are presented in thousands.

Note 2: Total skin disease is defined as the sum of the number of cases with palpable nodules, reversible and irreversible skin disease.

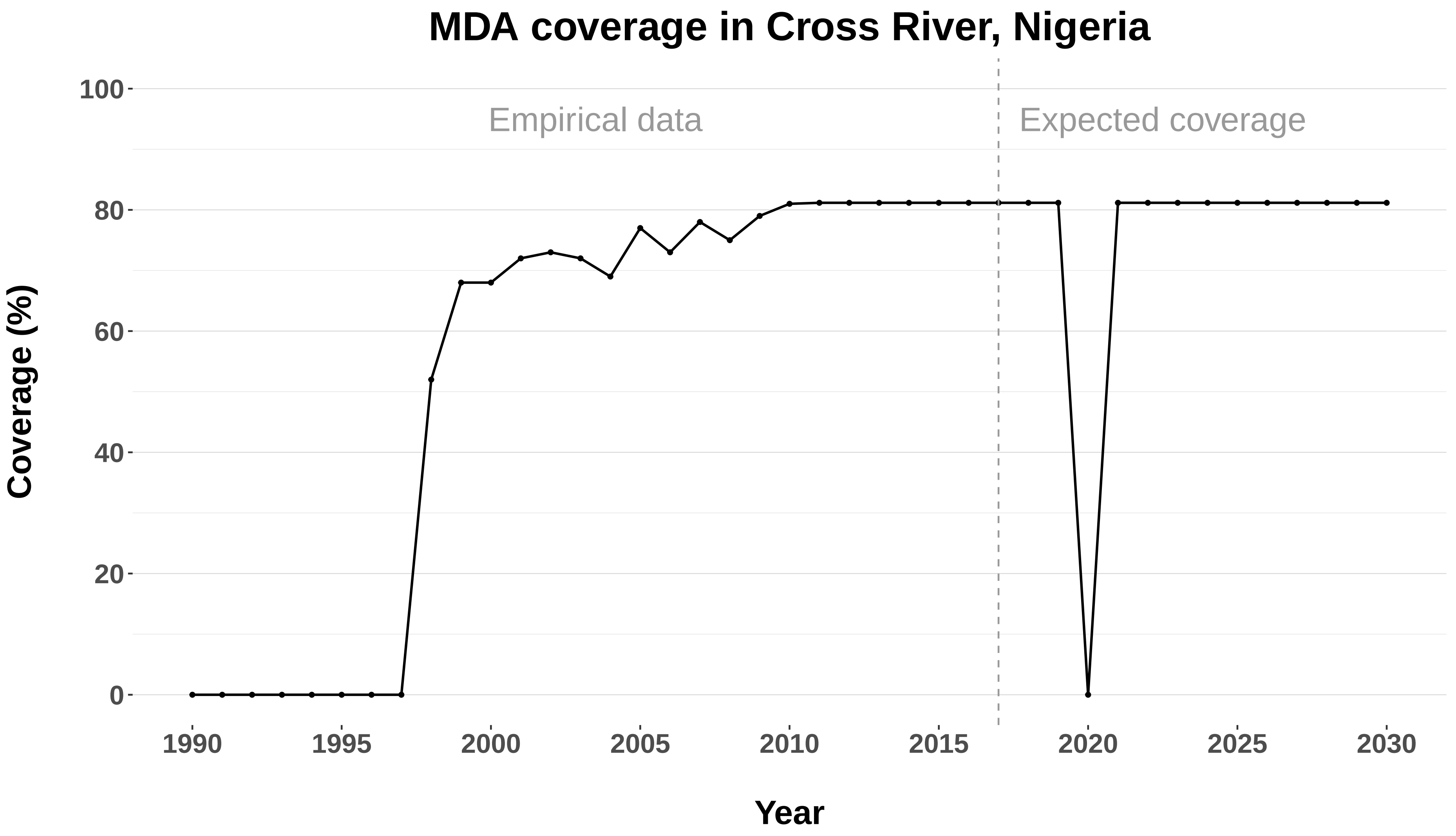
MDA coverage in Cross River, Nigeria
Empirical data
Expected coverage
Coverage (%)
Year
100
80
60
40
20
0
1990
1995
2000
2005
2010
2015
2020
2025
2030

**Project, country:**	**Edo Delta, Nigeria**
Bioclime:	
Endemicity level(s):	Hyperendemic
MDA start year:	1999
Mean MDA coverage per year:	75.6

	1990			2020			2030		
	N	%	DALYs	N	%	DALYs	N	%	DALYs
Total populaton at risk	956	100	_	2,158	100	_	2,831	100	_
**Infection:**									
Mf-positive	266	27.8	_	<0.5	<0.05	_	<0.5	<0.05	_
Mf-positive (age 5+)	265	27.8	_	<0.5	<0.05	_	<0.5	<0.05	_
Worm infected	351	36.7	_	12	0.6	_	<0.5	<0.05	_
**Clinical manifestations:**									
Palpable nodules	52	5.4	1	<0.5	<0.05	<0.5	<0.5	<0.05	<0.5
Reversible skin disease:									
Severe itch	34	3.5	6	<0.5	<0.05	<0.5	<0.5	<0.05	<0.5
Reactive skin disease	23	2.4	1	<0.5	<0.05	<0.5	<0.5	<0.05	<0.5
**Reversible skin disease**	**57**	**5.9**	**7**	**<0.5**	**<0.05**	**<0.5**	**<0.5**	**<0.05**	**<0.5**
Irreversible skin disease:									
Mild depigmentation	9	0.9	<0.5	6	0.3	<0.5	3	0.1	<0.5
Severe depigmentation	8	0.8	<0.5	5	0.2	<0.5	3	0.1	<0.5
Atrophy	<0.5	<0.05	<0.5	<0.5	<0.05	<0.5	<0.5	<0.05	<0.5
Hanging groin	<0.5	<0.05	<0.5	<0.5	<0.05	<0.5	<0.5	<0.05	<0.5
**Irreversible skin disease**	**16**	**1.7**	**1**	**11**	**0.5**	**<0.5**	**6**	**0.2**	**<0.5**
**Total skin disease (OSD)**	**125**	**13.1**	**8**	**11**	**0.5**	**<0.5**	**6**	**0.2**	**<0.5**
Eye disease (OED):									
Visual impairment	<0.5	<0.05	<0.5	<0.5	<0.05	<0.5	<0.5	<0.05	<0.5
Blindness	<0.5	<0.05	<0.5	<0.5	<0.05	<0.5	<0.5	<0.05	<0.5
**Eye disease (OED)**	**<0.5**	**<0.05**	**<0.5**	**<0.5**	**<0.05**	**<0.5**	**<0.5**	**<0.05**	**<0.5**
**Total all manifestations**	**125**	**13.1**	**9**	**11**	**0.5**	**<0.5**	**6**	**0.2**	**<0.5**

Note 1: Population at risk, number of cases (N) and DALYs are presented in thousands.

Note 2: Total skin disease is defined as the sum of the number of cases with palpable nodules, reversible and irreversible skin disease.

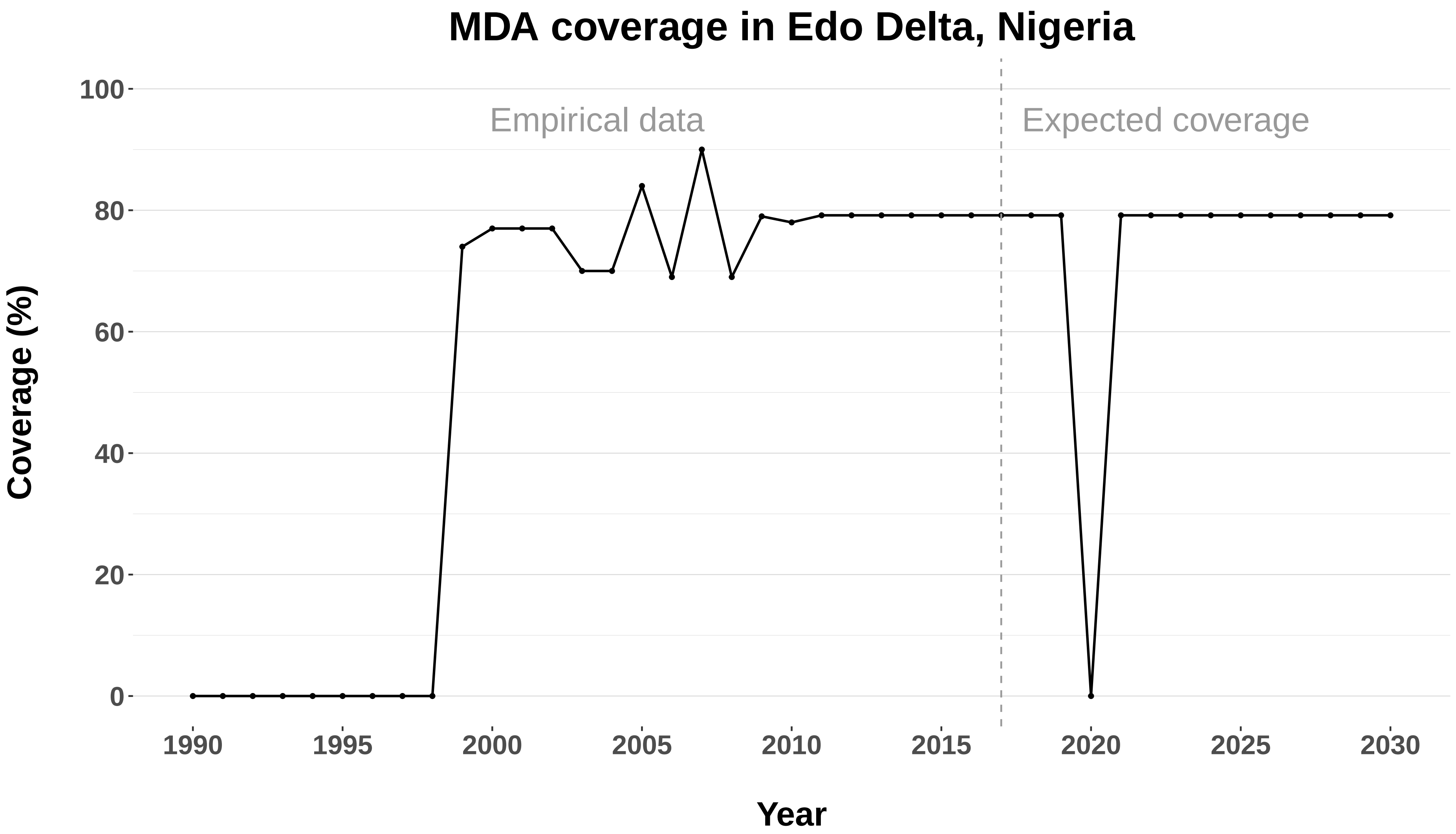

MDA coverage in Edo Delta, Nigeria
Empirical data
Expected coverage
Coverage (%)
100
80
60
40
20
0
1990
1995
2000
2005
2010
2015
2020
2025
2030
Year

**Project, country:**	**Ekiti, Nigeria**
Bioclime:	
Endemicity level(s):	Mesoendemic
MDA start year:	1999
Mean MDA coverage per year:	68.7

	1990			2020			2030		
	N	%	DALYs	N	%	DALYs	N	%	DALYs
Total populaton at risk	1,291	100	_	2,913	100	_	3,821	100	_
**Infection:**									
Mf-positive	265	20.5	_	1	<0.05	_	<0.5	<0.05	_
Mf-positive (age 5+)	264	20.5	_	1	<0.05	_	<0.5	<0.05	_
Worm infected	363	28.1	_	32	1.1	_	<0.5	<0.05	_
**Clinical manifestations:**									
Palpable nodules	28	2.2	<0.5	<0.5	<0.05	<0.5	<0.5	<0.05	<0.5
Reversible skin disease:									
Severe itch	31	2.4	6	<0.5	<0.05	<0.5	<0.5	<0.05	<0.5
Reactive skin disease	21	1.6	1	<0.5	<0.05	<0.5	<0.5	<0.05	<0.5
**Reversible skin disease**	**52**	**4.0**	**7**	**<0.5**	**<0.05**	**<0.5**	**<0.5**	**<0.05**	**<0.5**
Irreversible skin disease:									
Mild depigmentation	8	0.7	<0.5	7	0.2	<0.5	4	0.1	<0.5
Severe depigmentation	7	0.5	<0.5	4	0.1	<0.5	2	0.1	<0.5
Atrophy	<0.5	<0.05	<0.5	<0.5	<0.05	<0.5	<0.5	<0.05	<0.5
Hanging groin	<0.5	<0.05	<0.5	<0.5	<0.05	<0.5	<0.5	<0.05	<0.5
**Irreversible skin disease**	**16**	**1.2**	**1**	**11**	**0.4**	**<0.5**	**7**	**0.2**	**<0.5**
**Total skin disease (OSD)**	**95**	**7.3**	**7**	**11**	**0.4**	**<0.5**	**7**	**0.2**	**<0.5**
Eye disease (OED):									
Visual impairment	<0.5	<0.05	<0.5	<0.5	<0.05	<0.5	<0.5	<0.05	<0.5
Blindness	<0.5	<0.05	<0.5	<0.5	<0.05	<0.5	<0.5	<0.05	<0.5
**Eye disease (OED)**	**<0.5**	**<0.05**	**<0.5**	**<0.5**	**<0.05**	**<0.5**	**<0.5**	**<0.05**	**<0.5**
**Total all manifestations**	**95**	**7.4**	**8**	**11**	**0.4**	**<0.5**	**7**	**0.2**	**<0.5**

Note 1: Population at risk, number of cases (N) and DALYs are presented in thousands.

Note 2: Total skin disease is defined as the sum of the number of cases with palpable nodules, reversible and irreversible skin disease.

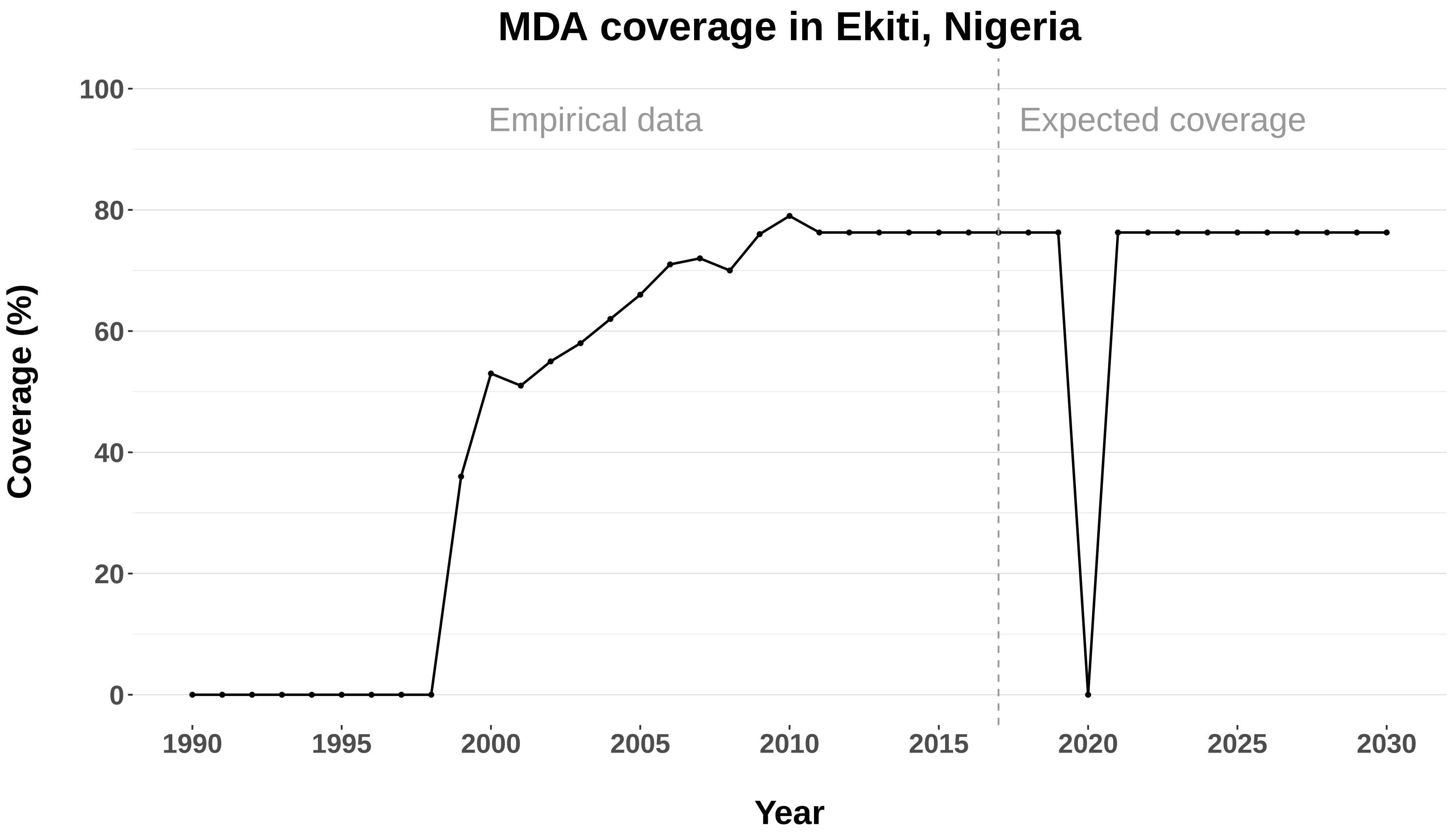

MDA coverage in Ekiti, Nigeria
Empirical data
Expected coverage
Coverage (%)
100
80
60
40
20
0
1990
1995
2000
2005
2010
2015
2020
2025
2030
Year

**Project, country:**	**Enugu Anambra Ebony, Nigeria**
Bioclime:	
Endemicity level(s):	Hyperendemic
MDA start year:	1999
Mean MDA coverage per year:	76.6

	1990			2020			2030		
	N	%	DALYs	N	%	DALYs	N	%	DALYs
Total populaton at risk	1,421	100	_	3,207	100	_	4,206	100	_
**Infection:**									
Mf-positive	691	48.6	_	5	0.2	_	<0.5	<0.05	_
Mf-positive (age 5+)	690	48.6	_	5	0.2	_	<0.5	<0.05	_
Worm infected	831	58.5	_	76	2.4	_	4	0.1	_
**Clinical manifestations:**									
Palpable nodules	273	19.2	3	3	0.1	<0.5	<0.5	<0.05	<0.5
Reversible skin disease:									
Severe itch	119	8.4	22	<0.5	<0.05	<0.5	<0.5	<0.05	<0.5
Reactive skin disease	92	6.5	4	<0.5	<0.05	<0.5	<0.5	<0.05	<0.5
**Reversible skin disease**	**211**	**14.9**	**26**	**<0.5**	**<0.05**	**<0.5**	**<0.5**	**<0.05**	**<0.5**
Irreversible skin disease:									
Mild depigmentation	25	1.8	<0.5	19	0.6	<0.5	13	0.3	<0.5
Severe depigmentation	31	2.2	2	21	0.6	1	13	0.3	1
Atrophy	<0.5	<0.05	<0.5	<0.5	<0.05	<0.5	<0.5	<0.05	<0.5
Hanging groin	1	<0.05	<0.5	<0.5	<0.05	<0.5	<0.5	<0.05	<0.5
**Irreversible skin disease**	**57**	**4.0**	**3**	**40**	**1.2**	**2**	**25**	**0.6**	**1**
**Total skin disease (OSD)**	**542**	**38.1**	**31**	**43**	**1.3**	**2**	**25**	**0.6**	**1**
Eye disease (OED):									
Visual impairment	1	0.1	<0.5	1	<0.05	<0.5	<0.5	<0.05	<0.5
Blindness	2	0.1	10	<0.5	<0.05	2	<0.5	<0.05	1
**Eye disease (OED)**	**3**	**0.2**	**10**	**1**	**<0.05**	**2**	**<0.5**	**<0.05**	**1**
**Total all manifestations**	**545**	**38.3**	**42**	**44**	**1.4**	**4**	**26**	**0.6**	**2**

Note 1: Population at risk, number of cases (N) and DALYs are presented in thousands.

Note 2: Total skin disease is defined as the sum of the number of cases with palpable nodules, reversible and irreversible skin disease.

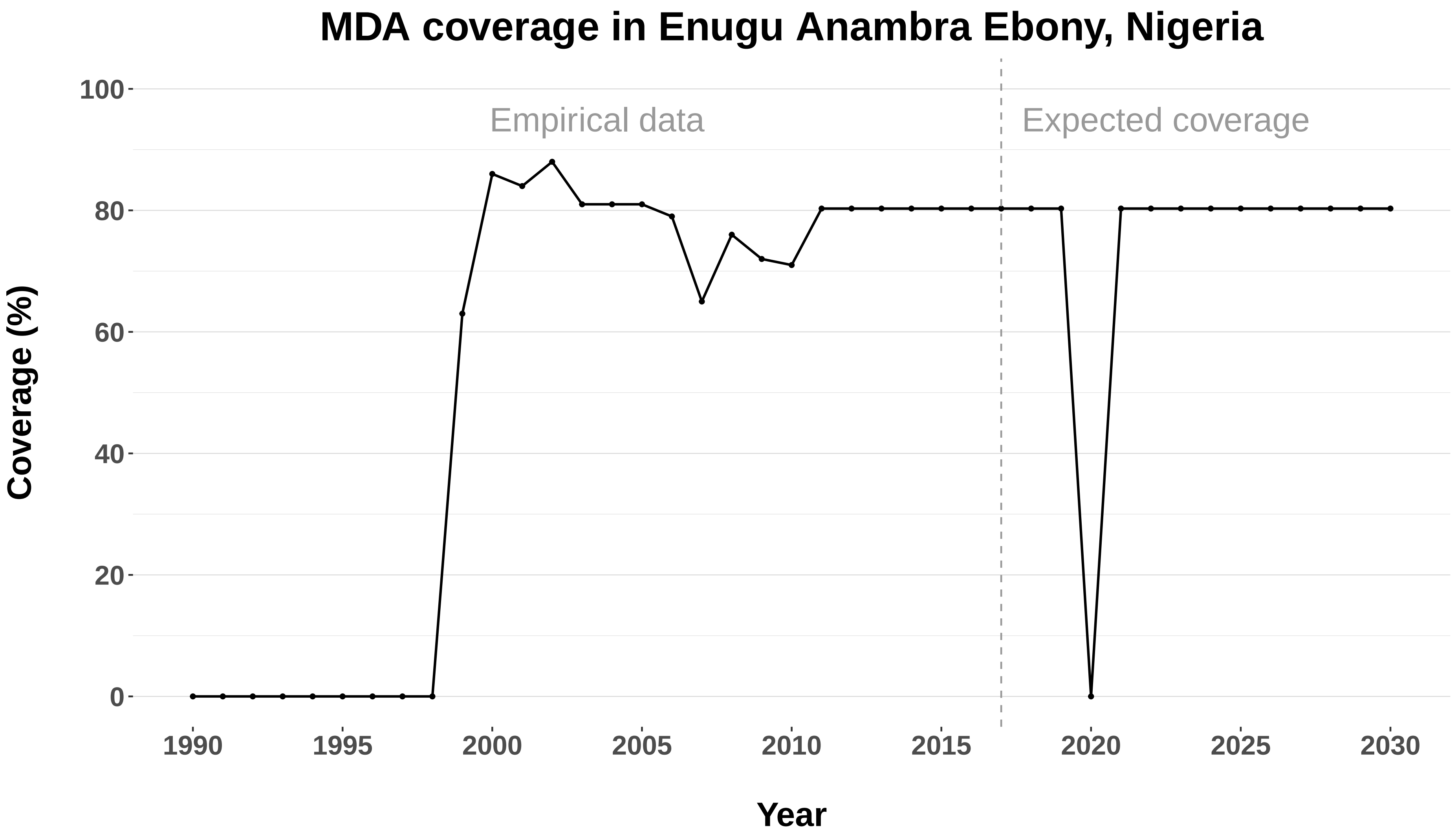
MDA coverage in Enugu Anambra Ebony, Nigeria
Empirical data
Expected coverage
Coverage (%)
100
80
60
40
20
0
1990
1995
2000
2005
2010
2015
2020
2025
2030
Year

**Project, country:**	**FCT, Nigeria**
Bioclime:	
Endemicity level(s):	Mesoendemic
MDA start year:	1998
Mean MDA coverage per year:	71.0

	1990			2020			2030		
	N	%	DALYs	N	%	DALYs	N	%	DALYs
Total populaton at risk	302	100	_	681	100	_	893	100	_
**Infection:**									
Mf-positive	67	22.3	_	<0.5	<0.05	_	<0.5	<0.05	_
Mf-positive (age 5+)	67	22.3	_	<0.5	<0.05	_	<0.5	<0.05	_
Worm infected	90	29.8	_	7	1.1	_	<0.5	<0.05	_
**Clinical manifestations:**									
Palpable nodules	8	2.6	<0.5	<0.5	<0.05	<0.5	<0.5	<0.05	<0.5
Reversible skin disease:									
Severe itch	8	2.8	2	<0.5	<0.05	<0.5	<0.5	<0.05	<0.5
Reactive skin disease	6	1.9	<0.5	<0.5	<0.05	<0.5	<0.5	<0.05	<0.5
**Reversible skin disease**	**14**	**4.7**	**2**	**<0.5**	**<0.05**	**<0.5**	**<0.5**	**<0.05**	**<0.5**
Irreversible skin disease:									
Mild depigmentation	2	0.8	<0.5	2	0.3	<0.5	1	0.1	<0.5
Severe depigmentation	2	0.6	<0.5	1	0.2	<0.5	1	0.1	<0.5
Atrophy	<0.5	<0.05	<0.5	<0.5	<0.05	<0.5	<0.5	<0.05	<0.5
Hanging groin	<0.5	<0.05	<0.5	<0.5	<0.05	<0.5	<0.5	<0.05	<0.5
**Irreversible skin disease**	**4**	**1.4**	**<0.5**	**3**	**0.4**	**<0.5**	**2**	**0.2**	**<0.5**
**Total skin disease (OSD)**	**26**	**8.7**	**2**	**3**	**0.5**	**<0.5**	**2**	**0.2**	**<0.5**
Eye disease (OED):									
Visual impairment	<0.5	<0.05	<0.5	<0.5	<0.05	<0.5	<0.5	<0.05	<0.5
Blindness	<0.5	<0.05	<0.5	<0.5	<0.05	<0.5	<0.5	<0.05	<0.5
**Eye disease (OED)**	**<0.5**	**<0.05**	**<0.5**	**<0.5**	**<0.05**	**<0.5**	**<0.5**	**<0.05**	**<0.5**
**Total all manifestations**	**26**	**8.7**	**2**	**3**	**0.5**	**<0.5**	**2**	**0.2**	**<0.5**

Note 1: Population at risk, number of cases (N) and DALYs are presented in thousands.

Note 2: Total skin disease is defined as the sum of the number of cases with palpable nodules, reversible and irreversible skin disease.

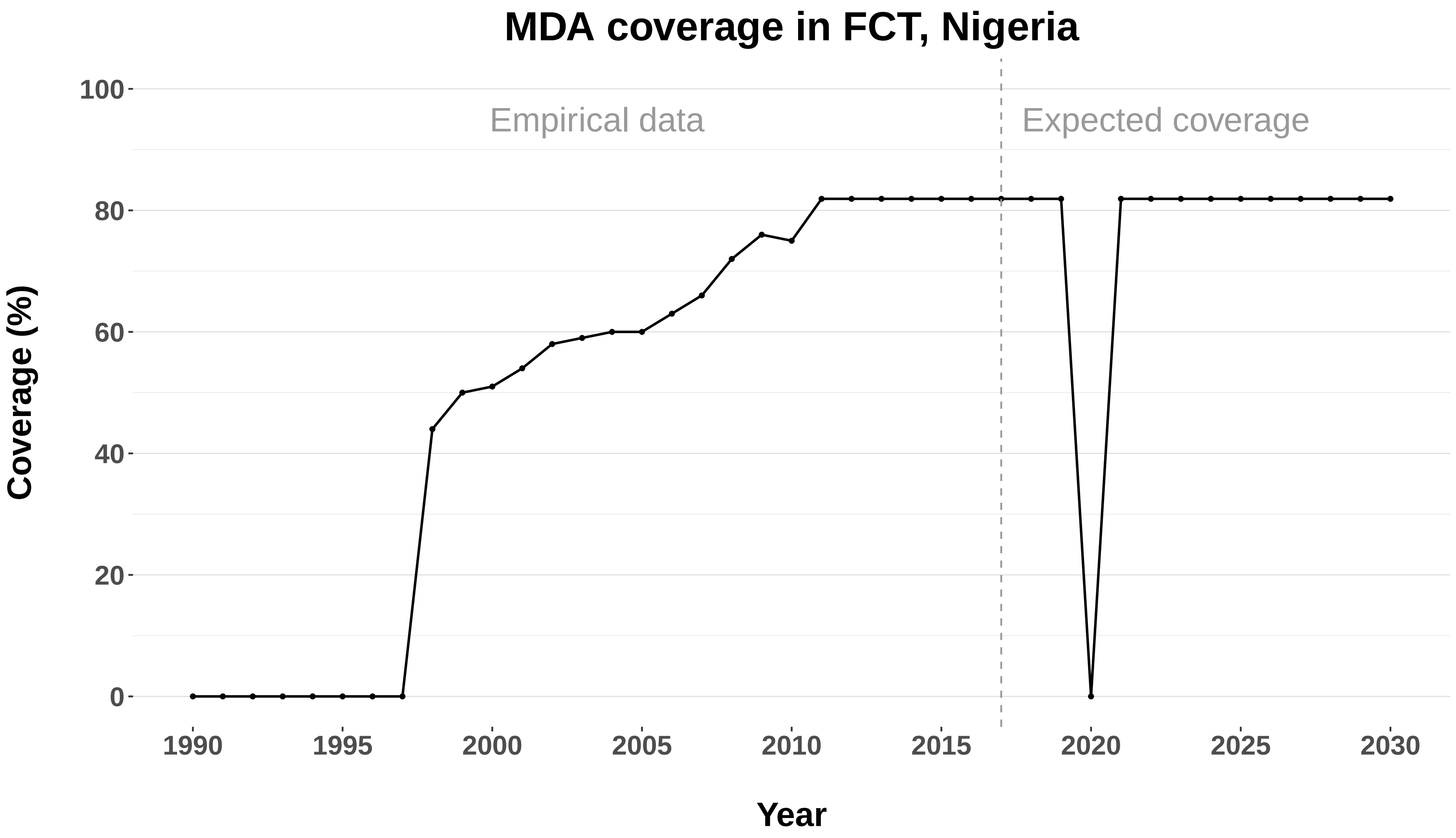

MDA coverage in FCT, Nigeria
Empirical data
Expected coverage
Coverage (%)
100
80
60
40
20
0
1990
1995
2000
2005
2010
2015
2020
2025
2030
Year

**Project, country:**	**Gombe, Nigeria**
Bioclime:	
Endemicity level(s):	Hyperendemic
MDA start year:	1999
Mean MDA coverage per year:	68.3

	1990			2020			2030		
	N	%	DALYs	N	%	DALYs	N	%	DALYs
Total populaton at risk	1,134	100	_	2,559	100	_	3,356	100	_
**Infection:**									
Mf-positive	261	23.1	_	2	0.1	_	<0.5	<0.05	_
Mf-positive (age 5+)	261	23.0	_	2	0.1	_	<0.5	<0.05	_
Worm infected	351	31.0	_	57	2.2	_	1	<0.05	_
**Clinical manifestations:**									
Palpable nodules	35	3.1	<0.5	1	<0.05	<0.5	<0.5	<0.05	<0.5
Reversible skin disease:									
Severe itch	32	2.9	6	<0.5	<0.05	<0.5	<0.5	<0.05	<0.5
Reactive skin disease	22	1.9	1	<0.5	<0.05	<0.5	<0.5	<0.05	<0.5
**Reversible skin disease**	**54**	**4.8**	**7**	**<0.5**	**<0.05**	**<0.5**	**<0.5**	**<0.05**	**<0.5**
Irreversible skin disease:									
Mild depigmentation	9	0.8	<0.5	7	0.3	<0.5	5	0.1	<0.5
Severe depigmentation	7	0.6	<0.5	5	0.2	<0.5	3	0.1	<0.5
Atrophy	<0.5	<0.05	<0.5	<0.5	<0.05	<0.5	<0.5	<0.05	<0.5
Hanging groin	<0.5	<0.05	<0.5	<0.5	<0.05	<0.5	<0.5	<0.05	<0.5
**Irreversible skin disease**	**16**	**1.4**	**1**	**12**	**0.5**	**<0.5**	**8**	**0.2**	**<0.5**
**Total skin disease (OSD)**	**106**	**9.3**	**8**	**14**	**0.5**	**<0.5**	**8**	**0.2**	**<0.5**
Eye disease (OED):									
Visual impairment	15	1.3	<0.5	11	0.4	<0.5	7	0.2	<0.5
Blindness	5	0.4	32	1	0.1	10	<0.5	<0.05	3
**Eye disease (OED)**	**20**	**1.7**	**33**	**13**	**0.5**	**10**	**7**	**0.2**	**3**
**Total all manifestations**	**125**	**11.0**	**41**	**26**	**1.0**	**11**	**15**	**0.4**	**4**

Note 1: Population at risk, number of cases (N) and DALYs are presented in thousands.

Note 2: Total skin disease is defined as the sum of the number of cases with palpable nodules, reversible and irreversible skin disease.

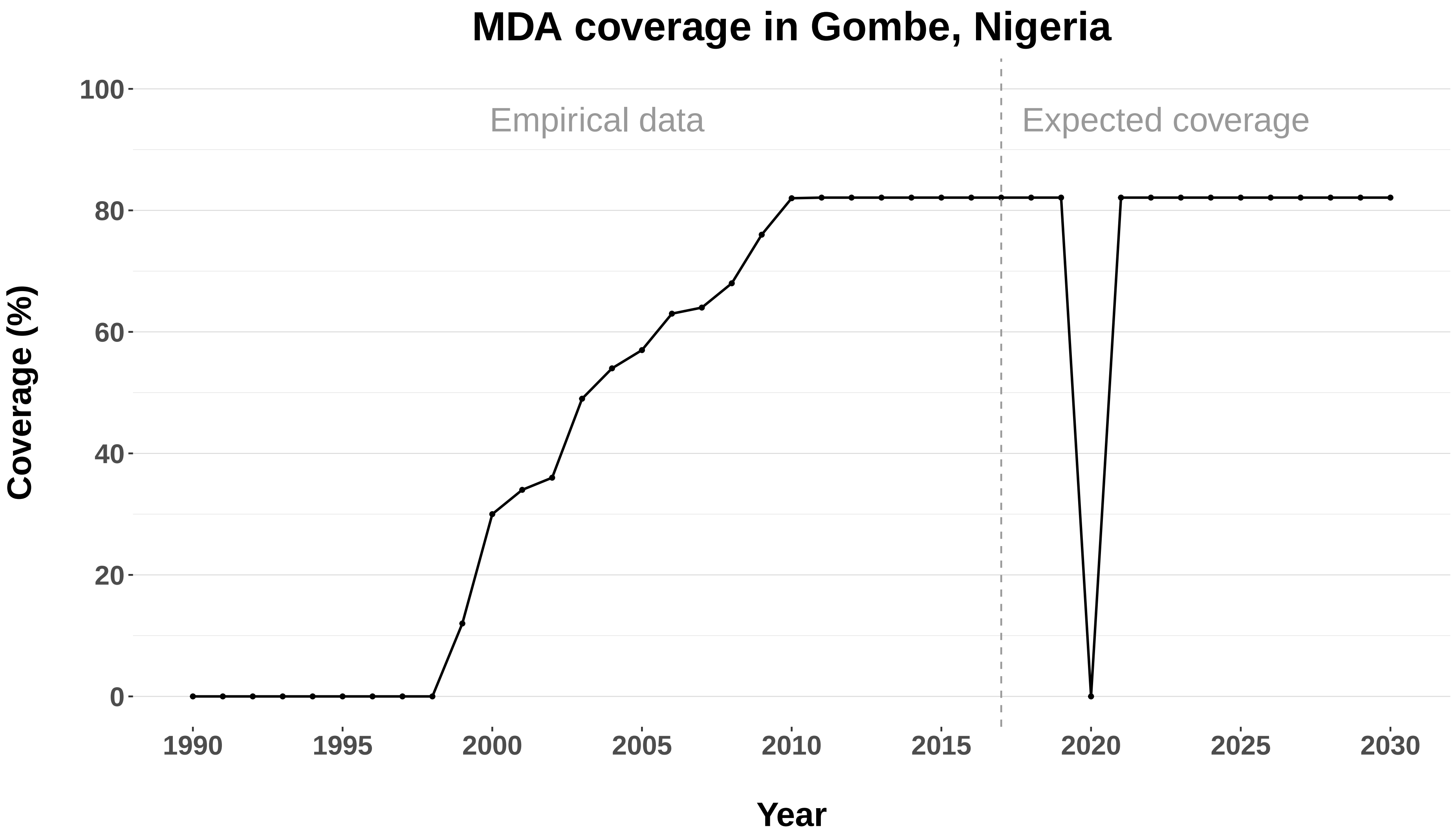
MDA coverage in Gombe, Nigeria
Empirical data
Expected coverage
Coverage (%)
100
80
60
40
20
0
1990
1995
2000
2005
2010
2015
2020
2025
2030
Year

**Project, country:**	**Imo Abia, Nigeria**
Bioclime:	
Endemicity level(s):	Hyperendemic
MDA start year:	1999
Mean MDA coverage per year:	65.5

	1990			2020			2030		
	N	%	DALYs	N	%	DALYs	N	%	DALYs
Total populaton at risk	783	100	_	1,768	100	_	2,319	100	_
**Infection:**									
Mf-positive	226	28.8	_	5	0.3	_	<0.5	<0.05	_
Mf-positive (age 5+)	226	28.8	_	5	0.3	_	<0.5	<0.05	_
Worm infected	293	37.4	_	63	3.6	_	2	0.1	_
**Clinical manifestations:**									
Palpable nodules	49	6.3	1	2	0.1	<0.5	<0.5	<0.05	<0.5
Reversible skin disease:									
Severe itch	31	3.9	6	<0.5	<0.05	<0.5	<0.5	<0.05	<0.5
Reactive skin disease	22	2.8	1	<0.5	<0.05	<0.5	<0.5	<0.05	<0.5
**Reversible skin disease**	**52**	**6.7**	**7**	**1**	**<0.05**	**<0.5**	**<0.5**	**<0.05**	**<0.5**
Irreversible skin disease:									
Mild depigmentation	7	1.0	<0.5	6	0.3	<0.5	4	0.2	<0.5
Severe depigmentation	7	0.9	<0.5	5	0.3	<0.5	3	0.1	<0.5
Atrophy	<0.5	<0.05	<0.5	<0.5	<0.05	<0.5	<0.5	<0.05	<0.5
Hanging groin	<0.5	<0.05	<0.5	<0.5	<0.05	<0.5	<0.5	<0.05	<0.5
**Irreversible skin disease**	**15**	**1.9**	**1**	**11**	**0.6**	**<0.5**	**6**	**0.3**	**<0.5**
**Total skin disease (OSD)**	**116**	**14.8**	**8**	**13**	**0.7**	**<0.5**	**6**	**0.3**	**<0.5**
Eye disease (OED):									
Visual impairment	<0.5	<0.05	<0.5	<0.5	<0.05	<0.5	<0.5	<0.05	<0.5
Blindness	<0.5	<0.05	1	<0.5	<0.05	<0.5	<0.5	<0.05	<0.5
**Eye disease (OED)**	**<0.5**	**<0.05**	**1**	**<0.5**	**<0.05**	**<0.5**	**<0.5**	**<0.05**	**<0.5**
**Total all manifestations**	**117**	**14.9**	**8**	**13**	**0.7**	**1**	**6**	**0.3**	**<0.5**

Note 1: Population at risk, number of cases (N) and DALYs are presented in thousands.

Note 2: Total skin disease is defined as the sum of the number of cases with palpable nodules, reversible and irreversible skin disease.

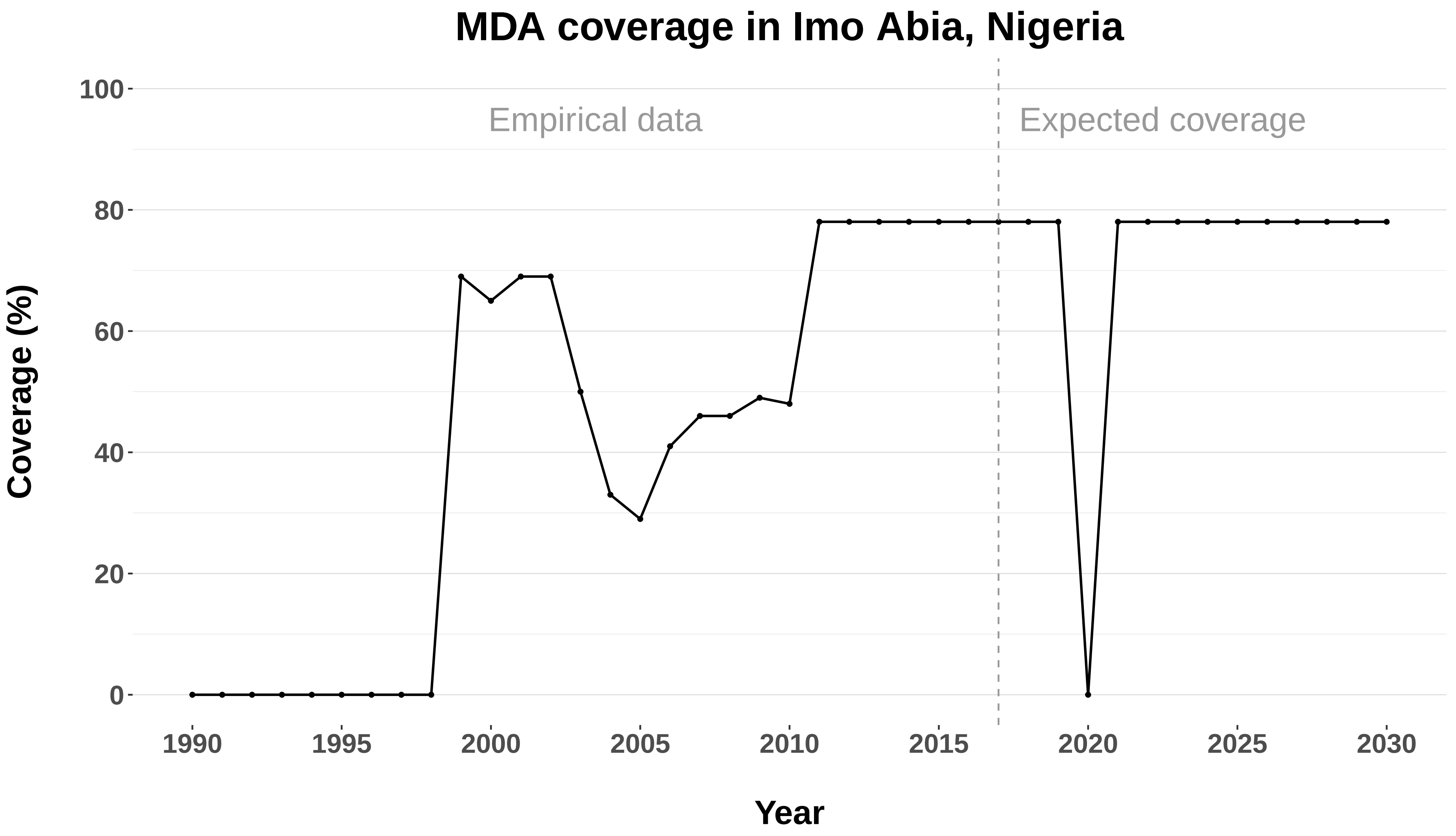
MDA coverage in Imo Abia, Nigeria
Empirical data
Expected coverage
Coverage (%)
0
20
40
60
80
100
1990
1995
2000
2005
2010
2015
2020
2025
2030
Year

**Project, country:**	**Jigawa, Nigeria**
Bioclime:	
Endemicity level(s):	Hypoendemic
MDA start year:	1999
Mean MDA coverage per year:	63.0

	1990			2020			2030		
	N	%	DALYs	N	%	DALYs	N	%	DALYs
Total populaton at risk	212	100	_	479	100	_	629	100	_
**Infection:**									
Mf-positive	45	21.1	_	<0.5	0.1	_	<0.5	<0.05	_
Mf-positive (age 5+)	45	21.1	_	<0.5	0.1	_	<0.5	<0.05	_
Worm infected	61	28.6	_	10	2.0	_	<0.5	<0.05	_
**Clinical manifestations:**									
Palpable nodules	5	2.2	<0.5	<0.5	<0.05	<0.5	<0.5	<0.05	<0.5
Reversible skin disease:									
Severe itch	5	2.5	1	<0.5	<0.05	<0.5	<0.5	<0.05	<0.5
Reactive skin disease	3	1.6	<0.5	<0.5	<0.05	<0.5	<0.5	<0.05	<0.5
**Reversible skin disease**	**9**	**4.2**	**1**	**<0.5**	**<0.05**	**<0.5**	**<0.5**	**<0.05**	**<0.5**
Irreversible skin disease:									
Mild depigmentation	1	0.7	<0.5	1	0.3	<0.5	1	0.1	<0.5
Severe depigmentation	1	0.6	<0.5	1	0.2	<0.5	<0.5	0.1	<0.5
Atrophy	<0.5	<0.05	<0.5	<0.5	<0.05	<0.5	<0.5	<0.05	<0.5
Hanging groin	<0.5	<0.05	<0.5	<0.5	<0.05	<0.5	<0.5	<0.05	<0.5
**Irreversible skin disease**	**3**	**1.2**	**<0.5**	**2**	**0.4**	**<0.5**	**1**	**0.2**	**<0.5**
**Total skin disease (OSD)**	**16**	**7.6**	**1**	**2**	**0.5**	**<0.5**	**1**	**0.2**	**<0.5**
Eye disease (OED):									
Visual impairment	2	1.2	<0.5	2	0.4	<0.5	1	0.2	<0.5
Blindness	1	0.3	5	<0.5	<0.05	2	<0.5	<0.05	1
**Eye disease (OED)**	**3**	**1.5**	**5**	**2**	**0.4**	**2**	**1**	**0.2**	**1**
**Total all manifestations**	**19**	**9.1**	**6**	**4**	**0.9**	**2**	**2**	**0.4**	**1**

Note 1: Population at risk, number of cases (N) and DALYs are presented in thousands.

Note 2: Total skin disease is defined as the sum of the number of cases with palpable nodules, reversible and irreversible skin disease.

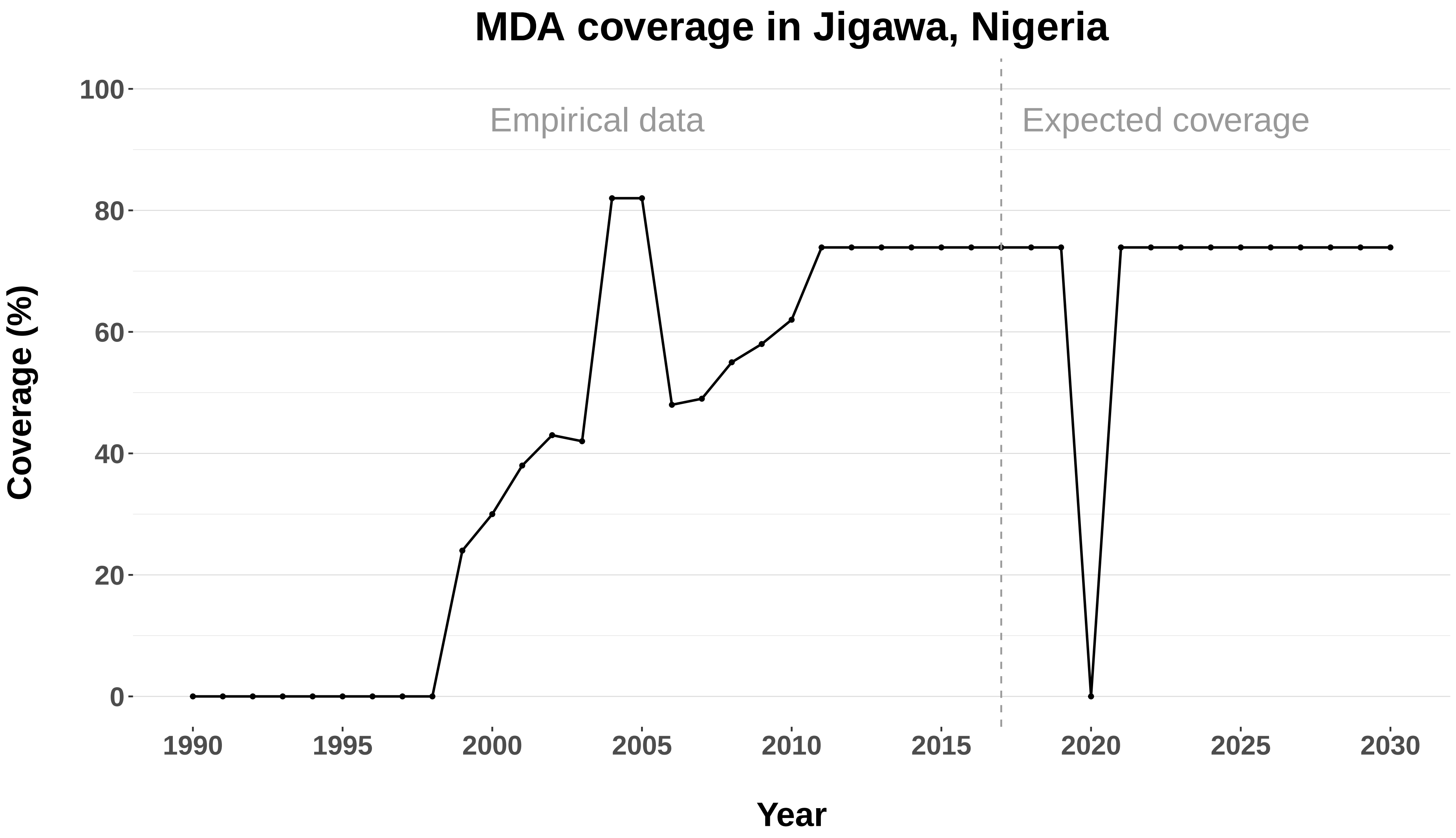

MDA coverage in Jigawa, Nigeria
Empirical data
Expected coverage
Coverage (%)
Year
0
20
40
60
80
100
1990
1995
2000
2005
2010
2015
2020
2025
2030

**Project, country:**	**Kaduna, Nigeria**
Bioclime:	
Endemicity level(s):	Mesoendemic
MDA start year:	1991
Mean MDA coverage per year:	77.0

	1990			2020			2030		
	N	%	DALYs	N	%	DALYs	N	%	DALYs
Total populaton at risk	1,650	100	_	3,723	100	_	4,884	100	_
**Infection:**									
Mf-positive	393	23.8	_	<0.5	<0.05	_	<0.5	<0.05	_
Mf-positive (age 5+)	392	23.8	_	<0.5	<0.05	_	<0.5	<0.05	_
Worm infected	522	31.6	_	<0.5	<0.05	_	<0.5	<0.05	_
**Clinical manifestations:**									
Palpable nodules	57	3.4	1	<0.5	<0.05	<0.5	<0.5	<0.05	<0.5
Reversible skin disease:									
Severe itch	50	3.0	9	<0.5	<0.05	<0.5	<0.5	<0.05	<0.5
Reactive skin disease	34	2.1	2	<0.5	<0.05	<0.5	<0.5	<0.05	<0.5
**Reversible skin disease**	**84**	**5.1**	**11**	**<0.5**	**<0.05**	**<0.5**	**<0.5**	**<0.05**	**<0.5**
Irreversible skin disease:									
Mild depigmentation	13	0.8	<0.5	5	0.1	<0.5	3	0.1	<0.5
Severe depigmentation	11	0.7	1	3	0.1	<0.5	2	<0.05	<0.5
Atrophy	<0.5	<0.05	<0.5	<0.5	<0.05	<0.5	<0.5	<0.05	<0.5
Hanging groin	<0.5	<0.05	<0.5	<0.5	<0.05	<0.5	<0.5	<0.05	<0.5
**Irreversible skin disease**	**24**	**1.5**	**1**	**9**	**0.2**	**<0.5**	**4**	**0.1**	**<0.5**
**Total skin disease (OSD)**	**164**	**10.0**	**12**	**9**	**0.2**	**<0.5**	**4**	**0.1**	**<0.5**
Eye disease (OED):									
Visual impairment	22	1.3	1	8	0.2	<0.5	4	0.1	<0.5
Blindness	7	0.4	49	1	<0.05	5	<0.5	<0.05	1
**Eye disease (OED)**	**29**	**1.8**	**50**	**9**	**0.2**	**5**	**4**	**0.1**	**1**
**Total all manifestations**	**194**	**11.7**	**62**	**17**	**0.5**	**6**	**8**	**0.2**	**1**

Note 1: Population at risk, number of cases (N) and DALYs are presented in thousands.

Note 2: Total skin disease is defined as the sum of the number of cases with palpable nodules, reversible and irreversible skin disease.

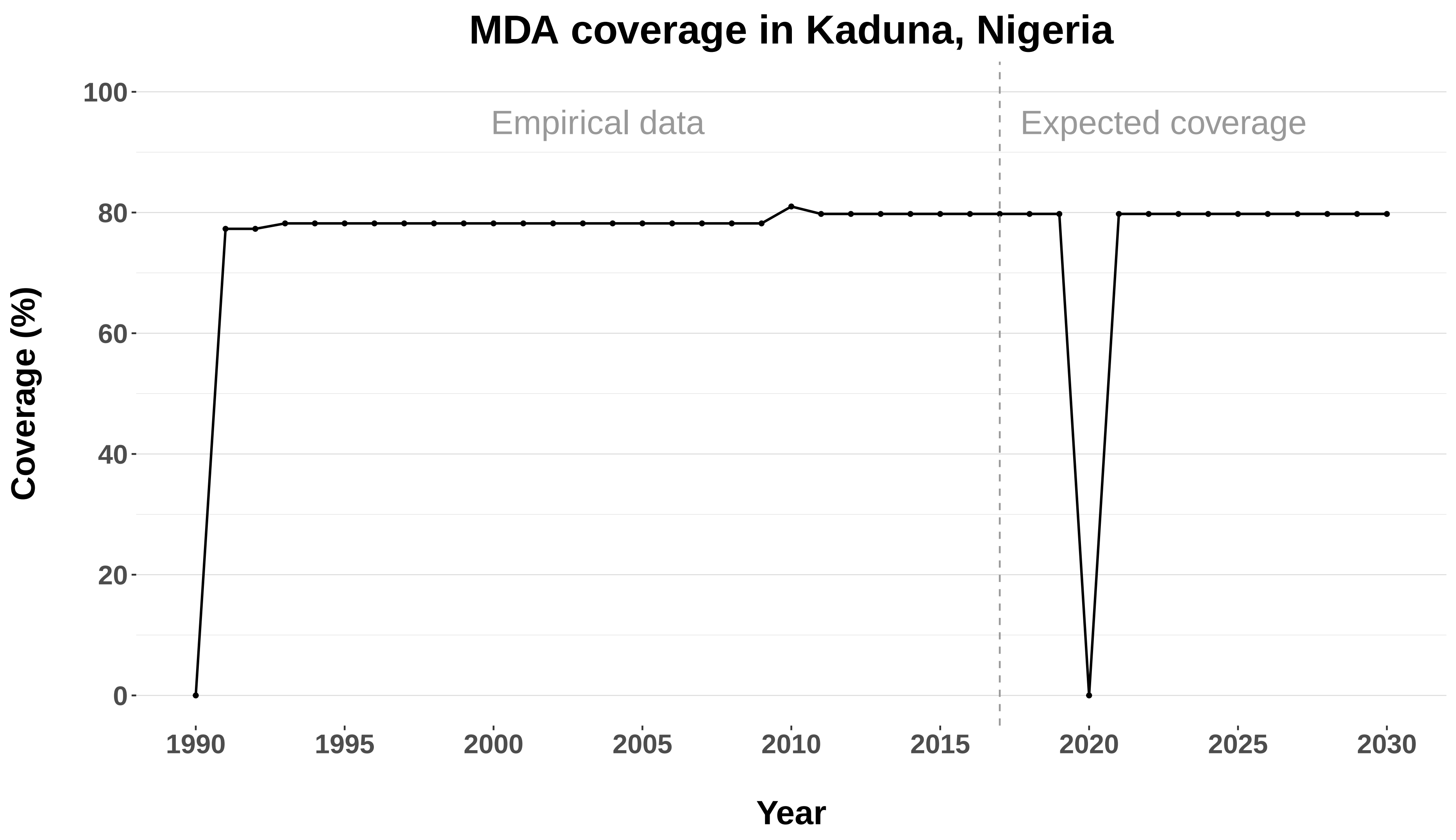

MDA coverage in Kaduna, Nigeria
Empirical data
Expected coverage
Coverage (%)
100
80
60
40
20
0
1990
1995
2000
2005
2010
2015
2020
2025
2030
Year

**Project, country:**	**Kano, Nigeria**
Bioclime:	
Endemicity level(s):	Hyperendemic
MDA start year:	1999
Mean MDA coverage per year:	76.0

	1990			2020			2030		
	N	%	DALYs	N	%	DALYs	N	%	DALYs
Total populaton at risk	589	100	_	1,329	100	_	1,743	100	_
**Infection:**									
Mf-positive	121	20.5	_	<0.5	<0.05	_	<0.5	<0.05	_
Mf-positive (age 5+)	121	20.5	_	<0.5	<0.05	_	<0.5	<0.05	_
Worm infected	167	28.3	_	5	0.4	_	<0.5	<0.05	_
**Clinical manifestations:**									
Palpable nodules	13	2.2	<0.5	<0.5	<0.05	<0.5	<0.5	<0.05	<0.5
Reversible skin disease:									
Severe itch	14	2.4	3	<0.5	<0.05	<0.5	<0.5	<0.05	<0.5
Reactive skin disease	9	1.6	<0.5	<0.5	<0.05	<0.5	<0.5	<0.05	<0.5
**Reversible skin disease**	**23**	**4.0**	**3**	**<0.5**	**<0.05**	**<0.5**	**<0.5**	**<0.05**	**<0.5**
Irreversible skin disease:									
Mild depigmentation	4	0.7	<0.5	3	0.2	<0.5	1	0.1	<0.5
Severe depigmentation	3	0.5	<0.5	2	0.1	<0.5	1	0.1	<0.5
Atrophy	<0.5	<0.05	<0.5	<0.5	<0.05	<0.5	<0.5	<0.05	<0.5
Hanging groin	<0.5	<0.05	<0.5	<0.5	<0.05	<0.5	<0.5	<0.05	<0.5
**Irreversible skin disease**	**7**	**1.2**	**<0.5**	**4**	**0.3**	**<0.5**	**3**	**0.2**	**<0.5**
**Total skin disease (OSD)**	**43**	**7.4**	**3**	**4**	**0.3**	**<0.5**	**3**	**0.2**	**<0.5**
Eye disease (OED):									
Visual impairment	7	1.1	<0.5	4	0.3	<0.5	2	0.1	<0.5
Blindness	2	0.3	13	1	<0.05	4	<0.5	<0.05	1
**Eye disease (OED)**	**8**	**1.4**	**13**	**5**	**0.3**	**4**	**2**	**0.1**	**1**
**Total all manifestations**	**52**	**8.8**	**17**	**9**	**0.7**	**4**	**5**	**0.3**	**1**

Note 1: Population at risk, number of cases (N) and DALYs are presented in thousands.

Note 2: Total skin disease is defined as the sum of the number of cases with palpable nodules, reversible and irreversible skin disease.

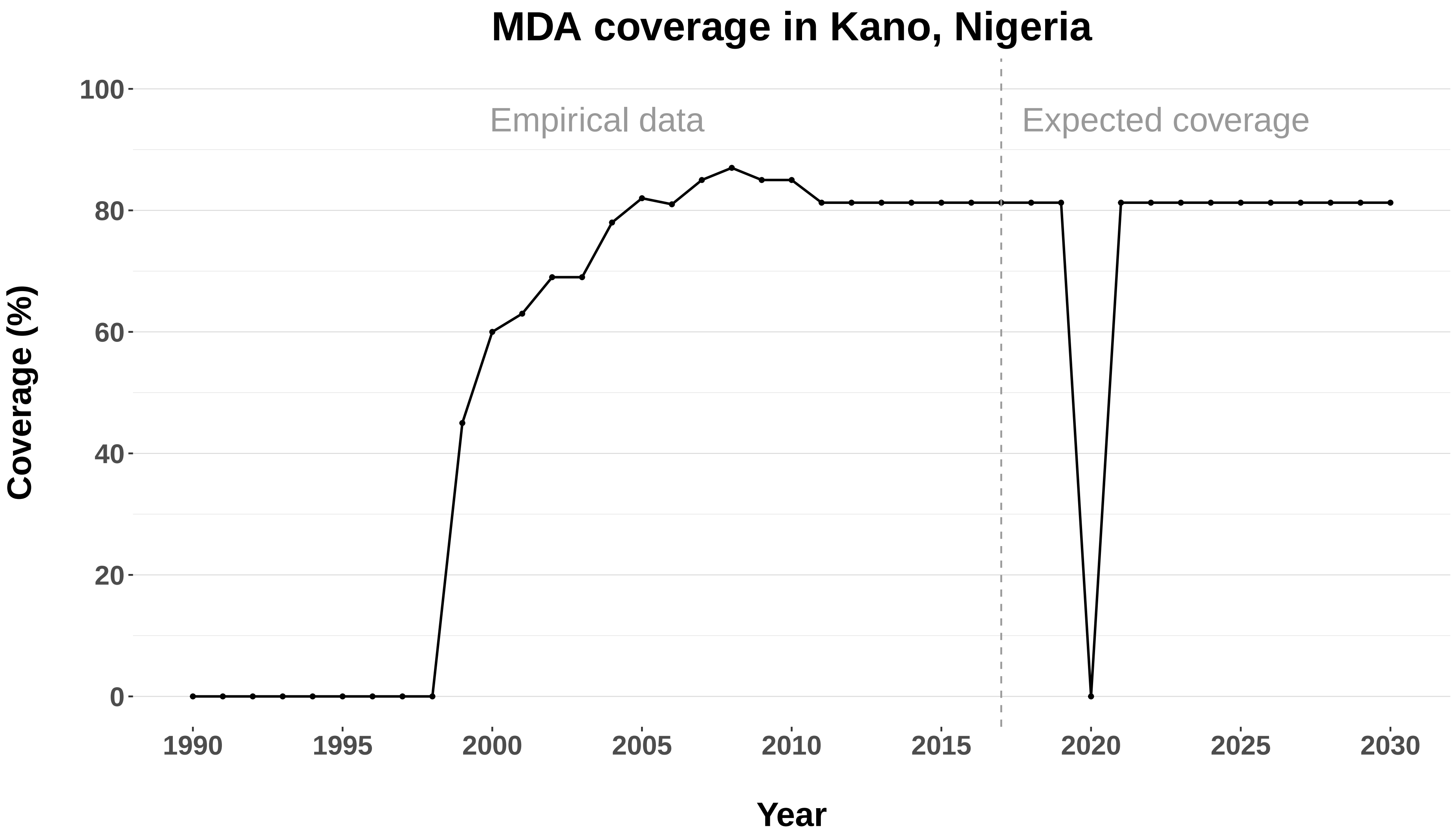
MDA coverage in Kano, Nigeria
Empirical data
Expected coverage
Coverage (%)
Year
0
20
40
60
80
100
1990
1995
2000
2005
2010
2015
2020
2025
2030

**Project, country:**	**Kebbi, Nigeria**
Bioclime:	
Endemicity level(s):	Hypoendemic
MDA start year:	1999
Mean MDA coverage per year:	66.8

	1990			2020			2030		
	N	%	DALYs	N	%	DALYs	N	%	DALYs
Total populaton at risk	124	100	_	279	100	_	366	100	_
**Infection:**									
Mf-positive	24	19.3	_	<0.5	<0.05	_	<0.5	<0.05	_
Mf-positive (age 5+)	24	19.2	_	<0.5	<0.05	_	<0.5	<0.05	_
Worm infected	34	27.1	_	3	1.2	_	<0.5	<0.05	_
**Clinical manifestations:**									
Palpable nodules	2	1.9	<0.5	<0.5	<0.05	<0.5	<0.5	<0.05	<0.5
Reversible skin disease:									
Severe itch	3	2.2	<0.5	<0.5	<0.05	<0.5	<0.5	<0.05	<0.5
Reactive skin disease	2	1.5	<0.5	<0.5	<0.05	<0.5	<0.5	<0.05	<0.5
**Reversible skin disease**	**5**	**3.7**	**1**	**<0.5**	**<0.05**	**<0.5**	**<0.5**	**<0.05**	**<0.5**
Irreversible skin disease:									
Mild depigmentation	1	0.6	<0.5	1	0.2	<0.5	<0.5	0.1	<0.5
Severe depigmentation	1	0.4	<0.5	<0.5	0.1	<0.5	<0.5	0.1	<0.5
Atrophy	<0.5	<0.05	<0.5	<0.5	<0.05	<0.5	<0.5	<0.05	<0.5
Hanging groin	<0.5	<0.05	<0.5	<0.5	<0.05	<0.5	<0.5	<0.05	<0.5
**Irreversible skin disease**	**1**	**1.1**	**<0.5**	**1**	**0.3**	**<0.5**	**1**	**0.2**	**<0.5**
**Total skin disease (OSD)**	**8**	**6.7**	**1**	**1**	**0.3**	**<0.5**	**1**	**0.2**	**<0.5**
Eye disease (OED):									
Visual impairment	1	1.0	<0.5	1	0.3	<0.5	<0.5	0.1	<0.5
Blindness	<0.5	0.3	2	<0.5	<0.05	1	<0.5	<0.05	<0.5
**Eye disease (OED)**	**2**	**1.3**	**3**	**1**	**0.3**	**1**	**1**	**0.1**	**<0.5**
**Total all manifestations**	**10**	**8.0**	**3**	**2**	**0.7**	**1**	**1**	**0.3**	**<0.5**

Note 1: Population at risk, number of cases (N) and DALYs are presented in thousands.

Note 2: Total skin disease is defined as the sum of the number of cases with palpable nodules, reversible and irreversible skin disease.

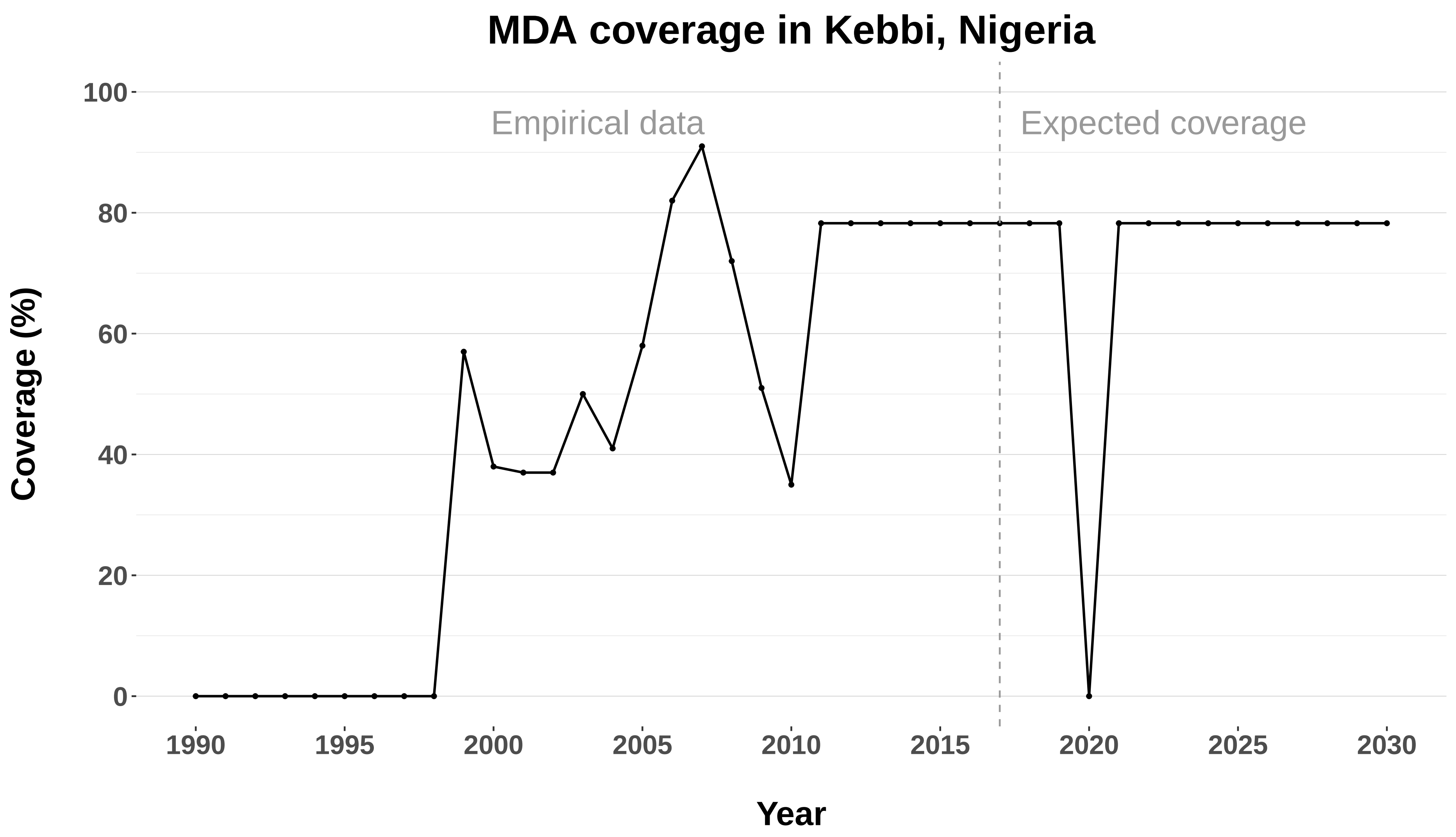
MDA coverage in Kebbi, Nigeria
Empirical data
Expected coverage
Coverage (%)
100
80
60
40
20
0
1990
1995
2000
2005
2010
2015
2020
2025
2030
Year

**Project, country:**	**Kogi, Nigeria**
Bioclime:	
Endemicity level(s):	Hyperendemic
MDA start year:	1998
Mean MDA coverage per year:	75.8

	1990			2020			2030		
	N	%	DALYs	N	%	DALYs	N	%	DALYs
Total populaton at risk	1,063	100	_	2,399	100	_	3,146	100	_
**Infection:**									
Mf-positive	275	25.9	_	<0.5	<0.05	_	<0.5	<0.05	_
Mf-positive (age 5+)	275	25.9	_	<0.5	<0.05	_	<0.5	<0.05	_
Worm infected	364	34.3	_	9	0.4	_	<0.5	<0.05	_
**Clinical manifestations:**									
Palpable nodules	48	4.5	1	<0.5	<0.05	<0.5	<0.5	<0.05	<0.5
Reversible skin disease:									
Severe itch	35	3.3	6	<0.5	<0.05	<0.5	<0.5	<0.05	<0.5
Reactive skin disease	24	2.2	1	<0.5	<0.05	<0.5	<0.5	<0.05	<0.5
**Reversible skin disease**	**58**	**5.5**	**7**	**<0.5**	**<0.05**	**<0.5**	**<0.5**	**<0.05**	**<0.5**
Irreversible skin disease:									
Mild depigmentation	9	0.8	<0.5	6	0.2	<0.5	4	0.1	<0.5
Severe depigmentation	8	0.7	1	5	0.2	<0.5	2	0.1	<0.5
Atrophy	<0.5	<0.05	<0.5	<0.5	<0.05	<0.5	<0.5	<0.05	<0.5
Hanging groin	<0.5	<0.05	<0.5	<0.5	<0.05	<0.5	<0.5	<0.05	<0.5
**Irreversible skin disease**	**17**	**1.6**	**1**	**10**	**0.4**	**<0.5**	**6**	**0.2**	**<0.5**
**Total skin disease (OSD)**	**123**	**11.6**	**9**	**11**	**0.4**	**<0.5**	**6**	**0.2**	**<0.5**
Eye disease (OED):									
Visual impairment	<0.5	<0.05	<0.5	<0.5	<0.05	<0.5	<0.5	<0.05	<0.5
Blindness	<0.5	<0.05	<0.5	<0.5	<0.05	<0.5	<0.5	<0.05	<0.5
**Eye disease (OED)**	**<0.5**	**<0.05**	**<0.5**	**<0.5**	**<0.05**	**<0.5**	**<0.5**	**<0.05**	**<0.5**
**Total all manifestations**	**124**	**11.6**	**9**	**11**	**0.4**	**<0.5**	**6**	**0.2**	**<0.5**

Note 1: Population at risk, number of cases (N) and DALYs are presented in thousands.

Note 2: Total skin disease is defined as the sum of the number of cases with palpable nodules, reversible and irreversible skin disease.

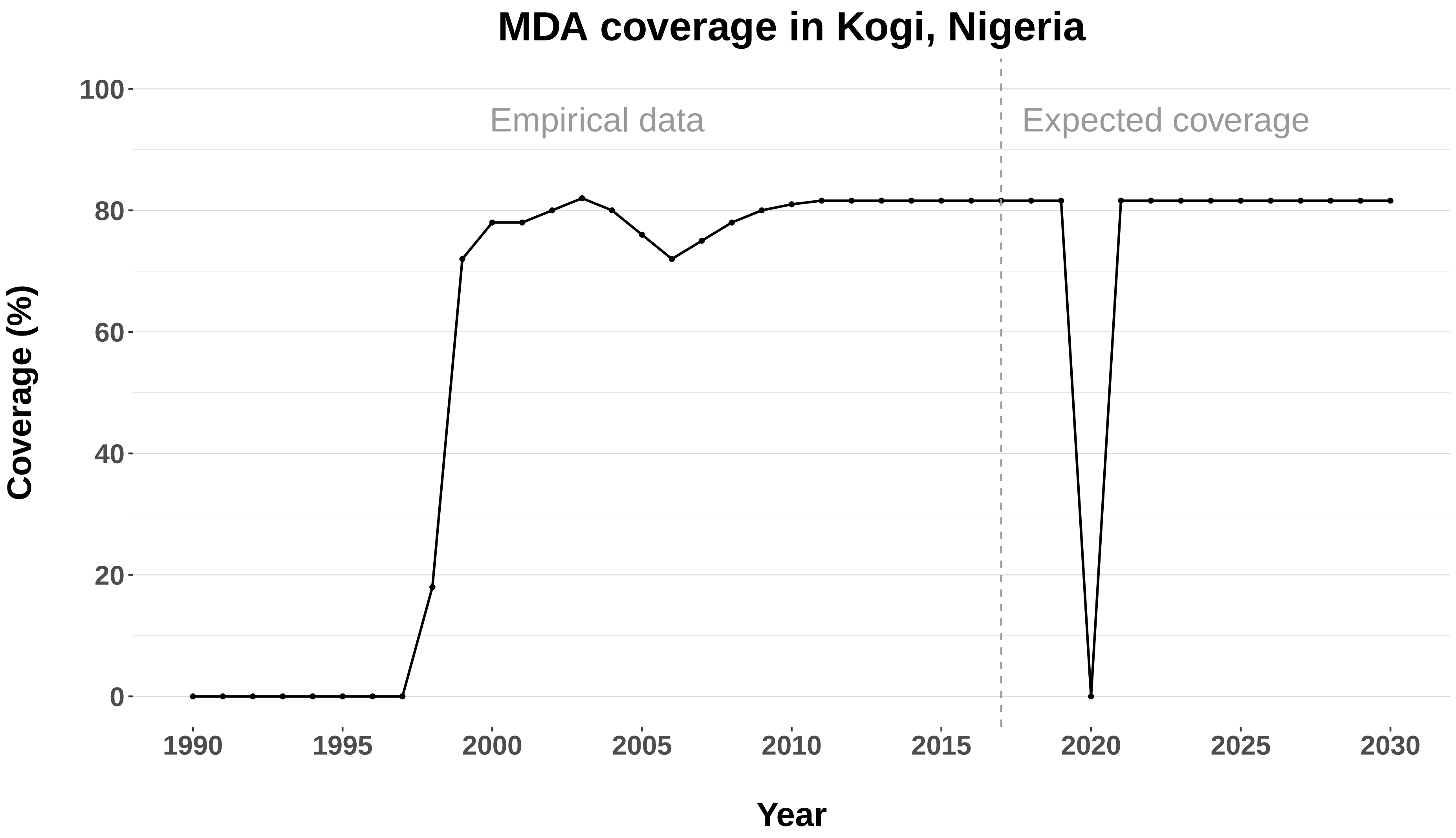

MDA coverage in Kogi, Nigeria
Empirical data
Expected coverage
Coverage (%)
100
80
60
40
20
0
1990
1995
2000
2005
2010
2015
2020
2025
2030
Year

**Project, country:**	**Kwara, Nigeria**
Bioclime:	
Endemicity level(s):	Hyperendemic
MDA start year:	1999
Mean MDA coverage per year:	73.0

	1990			2020			2030		
	N	%	DALYs	N	%	DALYs	N	%	DALYs
Total populaton at risk	898	100	_	2,027	100	_	2,659	100	_
**Infection:**									
Mf-positive	223	24.8	_	<0.5	<0.05	_	<0.5	<0.05	_
Mf-positive (age 5+)	223	24.8	_	<0.5	<0.05	_	<0.5	<0.05	_
Worm infected	301	33.5	_	20	1.0	_	<0.5	<0.05	_
**Clinical manifestations:**									
Palpable nodules	37	4.1	<0.5	<0.5	<0.05	<0.5	<0.5	<0.05	<0.5
Reversible skin disease:									
Severe itch	26	2.9	5	<0.5	<0.05	<0.5	<0.5	<0.05	<0.5
Reactive skin disease	18	2.0	1	<0.5	<0.05	<0.5	<0.5	<0.05	<0.5
**Reversible skin disease**	**45**	**5.0**	**6**	**<0.5**	**<0.05**	**<0.5**	**<0.5**	**<0.05**	**<0.5**
Irreversible skin disease:									
Mild depigmentation	7	0.8	<0.5	5	0.3	<0.5	3	0.1	<0.5
Severe depigmentation	6	0.6	<0.5	4	0.2	<0.5	2	0.1	<0.5
Atrophy	<0.5	<0.05	<0.5	<0.5	<0.05	<0.5	<0.5	<0.05	<0.5
Hanging groin	<0.5	<0.05	<0.5	<0.5	<0.05	<0.5	<0.5	<0.05	<0.5
**Irreversible skin disease**	**13**	**1.4**	**<0.5**	**9**	**0.4**	**<0.5**	**5**	**0.2**	**<0.5**
**Total skin disease (OSD)**	**94**	**10.5**	**6**	**9**	**0.5**	**<0.5**	**5**	**0.2**	**<0.5**
Eye disease (OED):									
Visual impairment	12	1.3	<0.5	8	0.4	<0.5	5	0.2	<0.5
Blindness	4	0.4	25	1	0.1	8	<0.5	<0.05	2
**Eye disease (OED)**	**16**	**1.7**	**26**	**9**	**0.5**	**9**	**5**	**0.2**	**2**
**Total all manifestations**	**110**	**12.2**	**32**	**19**	**0.9**	**9**	**10**	**0.4**	**3**

Note 1: Population at risk, number of cases (N) and DALYs are presented in thousands.

Note 2: Total skin disease is defined as the sum of the number of cases with palpable nodules, reversible and irreversible skin disease.

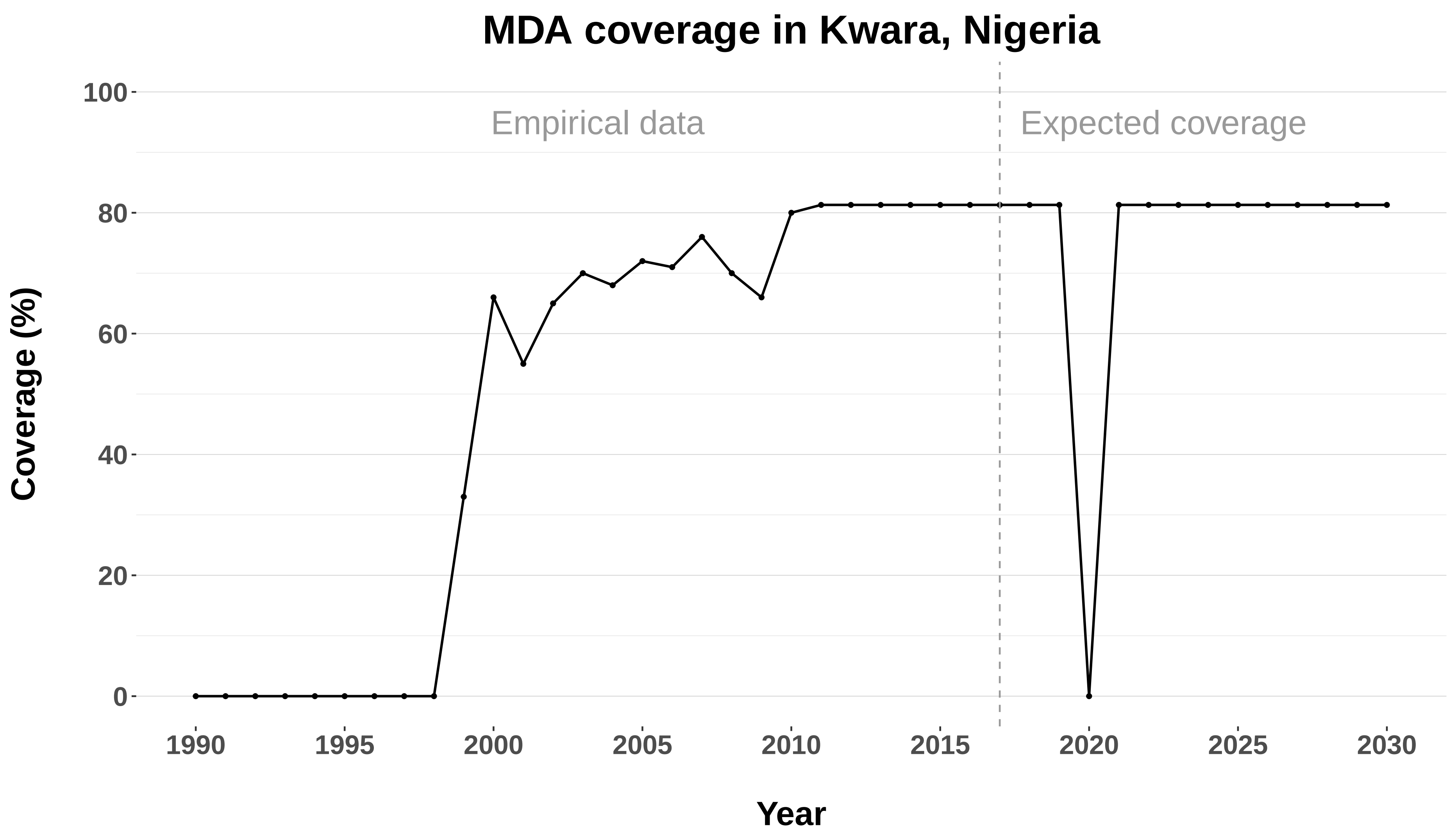
MDA coverage in Kwara, Nigeria
Empirical data
Expected coverage
Coverage (%)
100
80
60
40
20
0
1990
1995
2000
2005
2010
2015
2020
2025
2030
Year

**Project, country:**	**Niger, Nigeria**
Bioclime:	
Endemicity level(s):	Mesoendemic
MDA start year:	1999
Mean MDA coverage per year:	66.6

	1990			2020			2030		
	N	%	DALYs	N	%	DALYs	N	%	DALYs
Total populaton at risk	1,579	100	_	3,564	100	_	4,675	100	_
**Infection:**									
Mf-positive	332	21.0	_	2	0.1	_	<0.5	<0.05	_
Mf-positive (age 5+)	332	21.0	_	2	0.1	_	<0.5	<0.05	_
Worm infected	458	29.0	_	63	1.8	_	1	<0.05	_
**Clinical manifestations:**									
Palpable nodules	39	2.5	<0.5	1	<0.05	<0.5	<0.5	<0.05	<0.5
Reversible skin disease:									
Severe itch	39	2.5	7	<0.5	<0.05	<0.5	<0.5	<0.05	<0.5
Reactive skin disease	26	1.6	1	<0.5	<0.05	<0.5	<0.5	<0.05	<0.5
**Reversible skin disease**	**65**	**4.1**	**8**	**<0.5**	**<0.05**	**<0.5**	**<0.5**	**<0.05**	**<0.5**
Irreversible skin disease:									
Mild depigmentation	10	0.7	<0.5	8	0.2	<0.5	5	0.1	<0.5
Severe depigmentation	8	0.5	1	6	0.2	<0.5	4	0.1	<0.5
Atrophy	<0.5	<0.05	<0.5	<0.5	<0.05	<0.5	<0.5	<0.05	<0.5
Hanging groin	<0.5	<0.05	<0.5	<0.5	<0.05	<0.5	<0.5	<0.05	<0.5
**Irreversible skin disease**	**19**	**1.2**	**1**	**15**	**0.4**	**1**	**9**	**0.2**	**<0.5**
**Total skin disease (OSD)**	**123**	**7.8**	**9**	**16**	**0.4**	**1**	**9**	**0.2**	**<0.5**
Eye disease (OED):									
Visual impairment	18	1.1	1	13	0.4	<0.5	8	0.2	<0.5
Blindness	5	0.3	34	2	<0.05	11	1	<0.05	4
**Eye disease (OED)**	**23**	**1.4**	**35**	**15**	**0.4**	**11**	**8**	**0.2**	**4**
**Total all manifestations**	**145**	**9.2**	**44**	**30**	**0.8**	**12**	**17**	**0.4**	**5**

Note 1: Population at risk, number of cases (N) and DALYs are presented in thousands.

Note 2: Total skin disease is defined as the sum of the number of cases with palpable nodules, reversible and irreversible skin disease.

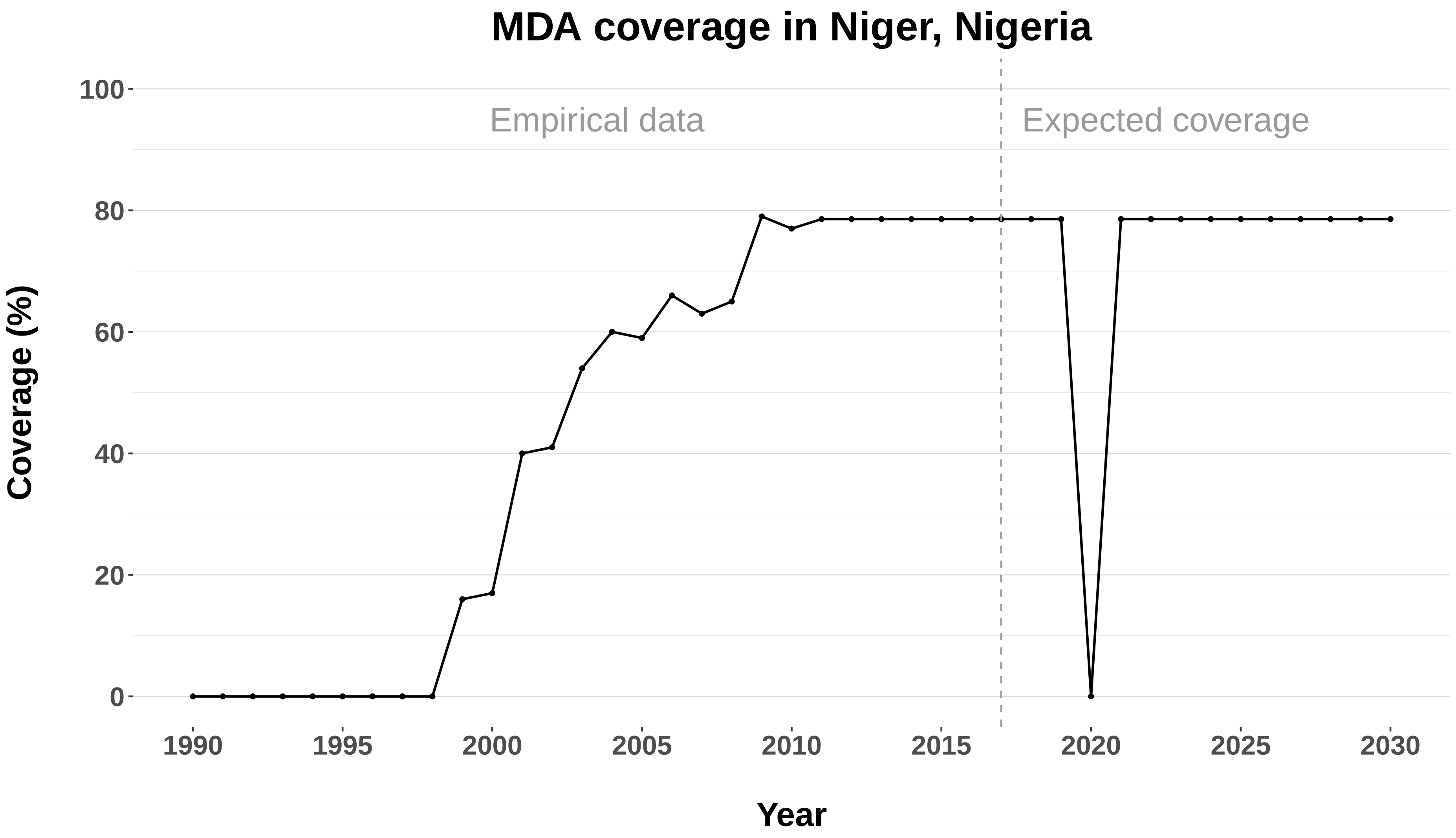
MDA coverage in Niger, Nigeria
Empirical data
Expected coverage
Coverage (%)
100
80
60
40
20
0
1990
1995
2000
2005
2010
2015
2020
2025
2030
Year

**Project, country:**	**Ogun, Nigeria**
Bioclime:	
Endemicity level(s):	Mesoendemic
MDA start year:	2002
Mean MDA coverage per year:	77.2

	1990			2020			2030		
	N	%	DALYs	N	%	DALYs	N	%	DALYs
Total populaton at risk	205	100	_	462	100	_	606	100	_
**Infection:**									
Mf-positive	57	27.9	_	<0.5	<0.05	_	<0.5	<0.05	_
Mf-positive (age 5+)	57	27.9	_	<0.5	<0.05	_	<0.5	<0.05	_
Worm infected	76	37.0	_	8	1.7	_	<0.5	<0.05	_
**Clinical manifestations:**									
Palpable nodules	11	5.4	<0.5	<0.5	<0.05	<0.5	<0.5	<0.05	<0.5
Reversible skin disease:									
Severe itch	7	3.5	1	<0.5	<0.05	<0.5	<0.5	<0.05	<0.5
Reactive skin disease	5	2.4	<0.5	<0.5	<0.05	<0.5	<0.5	<0.05	<0.5
**Reversible skin disease**	**12**	**5.9**	**2**	**<0.5**	**<0.05**	**<0.5**	**<0.5**	**<0.05**	**<0.5**
Irreversible skin disease:									
Mild depigmentation	2	0.9	<0.5	2	0.4	<0.5	1	0.2	<0.5
Severe depigmentation	2	0.8	<0.5	1	0.3	<0.5	1	0.1	<0.5
Atrophy	<0.5	<0.05	<0.5	<0.5	<0.05	<0.5	<0.5	<0.05	<0.5
Hanging groin	<0.5	<0.05	<0.5	<0.5	<0.05	<0.5	<0.5	<0.05	<0.5
**Irreversible skin disease**	**3**	**1.7**	**<0.5**	**3**	**0.6**	**<0.5**	**2**	**0.3**	**<0.5**
**Total skin disease (OSD)**	**27**	**13.0**	**2**	**3**	**0.6**	**<0.5**	**2**	**0.3**	**<0.5**
Eye disease (OED):									
Visual impairment	<0.5	<0.05	<0.5	<0.5	<0.05	<0.5	<0.5	<0.05	<0.5
Blindness	<0.5	<0.05	<0.5	<0.5	<0.05	<0.5	<0.5	<0.05	<0.5
**Eye disease (OED)**	**<0.5**	**<0.05**	**<0.5**	**<0.5**	**<0.05**	**<0.5**	**<0.5**	**<0.05**	**<0.5**
**Total all manifestations**	**27**	**13.0**	**2**	**3**	**0.6**	**<0.5**	**2**	**0.3**	**<0.5**

Note 1: Population at risk, number of cases (N) and DALYs are presented in thousands.

Note 2: Total skin disease is defined as the sum of the number of cases with palpable nodules, reversible and irreversible skin disease.

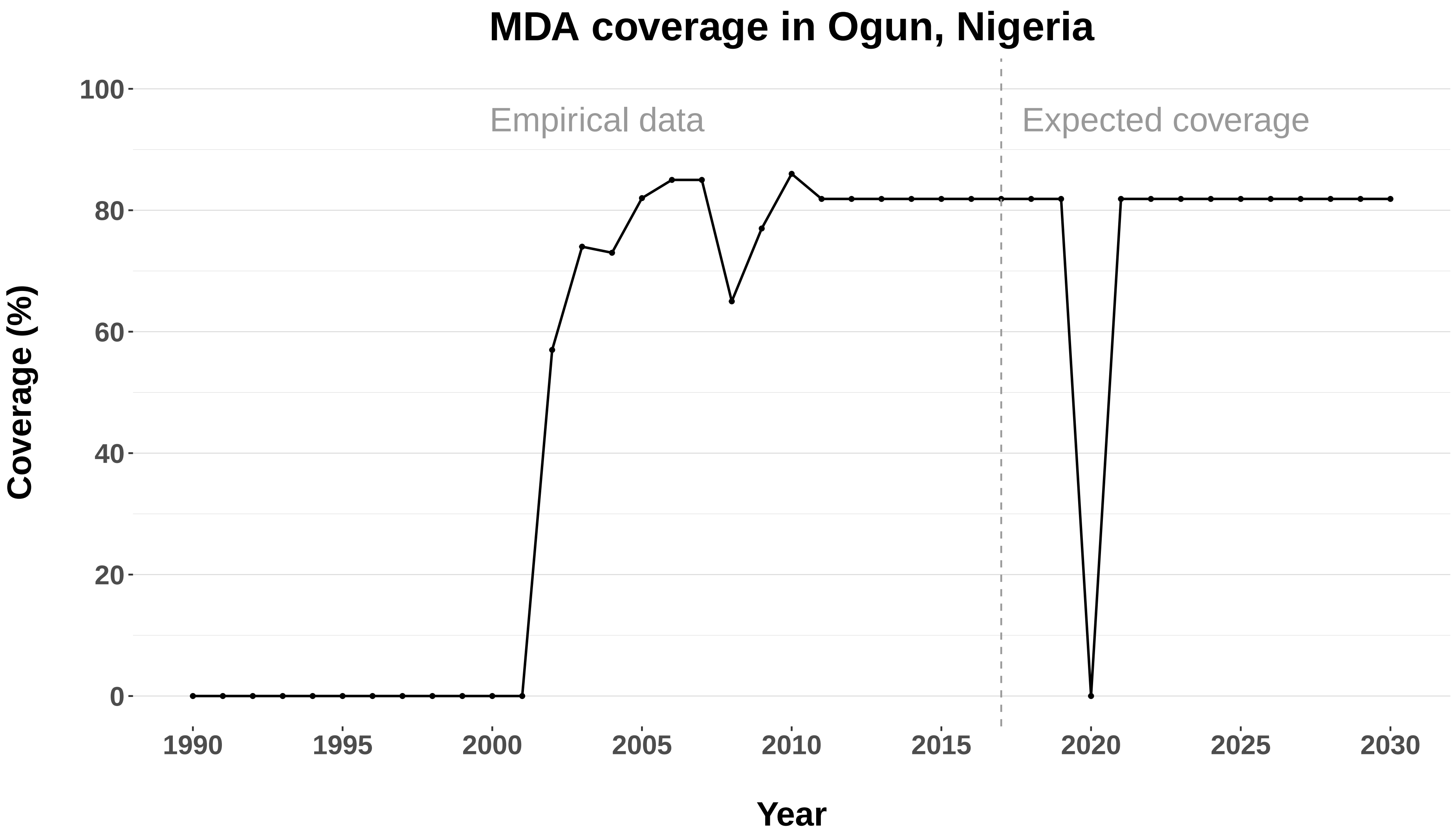
MDA coverage in Ogun, Nigeria
Empirical data
Expected coverage
Coverage (%)
100
80
60
40
20
0
1990
1995
2000
2005
2010
2015
2020
2025
2030
Year

**Project, country:**	**Ondo, Nigeria**
Bioclime:	
Endemicity level(s):	Mesoendemic
MDA start year:	1999
Mean MDA coverage per year:	75.9

	1990			2020			2030		
	N	%	DALYs	N	%	DALYs	N	%	DALYs
Total populaton at risk	814	100	_	1,837	100	_	2,409	100	_
**Infection:**									
Mf-positive	206	25.3	_	<0.5	<0.05	_	<0.5	<0.05	_
Mf-positive (age 5+)	206	25.3	_	<0.5	<0.05	_	<0.5	<0.05	_
Worm infected	279	34.3	_	8	0.5	_	<0.5	<0.05	_
**Clinical manifestations:**									
Palpable nodules	35	4.3	<0.5	<0.5	<0.05	<0.5	<0.5	<0.05	<0.5
Reversible skin disease:									
Severe itch	26	3.1	5	<0.5	<0.05	<0.5	<0.5	<0.05	<0.5
Reactive skin disease	17	2.1	1	<0.5	<0.05	<0.5	<0.5	<0.05	<0.5
**Reversible skin disease**	**42**	**5.2**	**5**	**<0.5**	**<0.05**	**<0.5**	**<0.5**	**<0.05**	**<0.5**
Irreversible skin disease:									
Mild depigmentation	7	0.8	<0.5	5	0.2	<0.5	3	0.1	<0.5
Severe depigmentation	6	0.7	<0.5	3	0.2	<0.5	2	0.1	<0.5
Atrophy	<0.5	<0.05	<0.5	<0.5	<0.05	<0.5	<0.5	<0.05	<0.5
Hanging groin	<0.5	<0.05	<0.5	<0.5	<0.05	<0.5	<0.5	<0.05	<0.5
**Irreversible skin disease**	**12**	**1.5**	**<0.5**	**8**	**0.4**	**<0.5**	**5**	**0.2**	**<0.5**
**Total skin disease (OSD)**	**90**	**11.0**	**6**	**8**	**0.4**	**<0.5**	**5**	**0.2**	**<0.5**
Eye disease (OED):									
Visual impairment	<0.5	<0.05	<0.5	<0.5	<0.05	<0.5	<0.5	<0.05	<0.5
Blindness	<0.5	<0.05	<0.5	<0.5	<0.05	<0.5	<0.5	<0.05	<0.5
**Eye disease (OED)**	**<0.5**	**<0.05**	**<0.5**	**<0.5**	**<0.05**	**<0.5**	**<0.5**	**<0.05**	**<0.5**
**Total all manifestations**	**90**	**11.0**	**7**	**8**	**0.4**	**<0.5**	**5**	**0.2**	**<0.5**

Note 1: Population at risk, number of cases (N) and DALYs are presented in thousands.

Note 2: Total skin disease is defined as the sum of the number of cases with palpable nodules, reversible and irreversible skin disease.

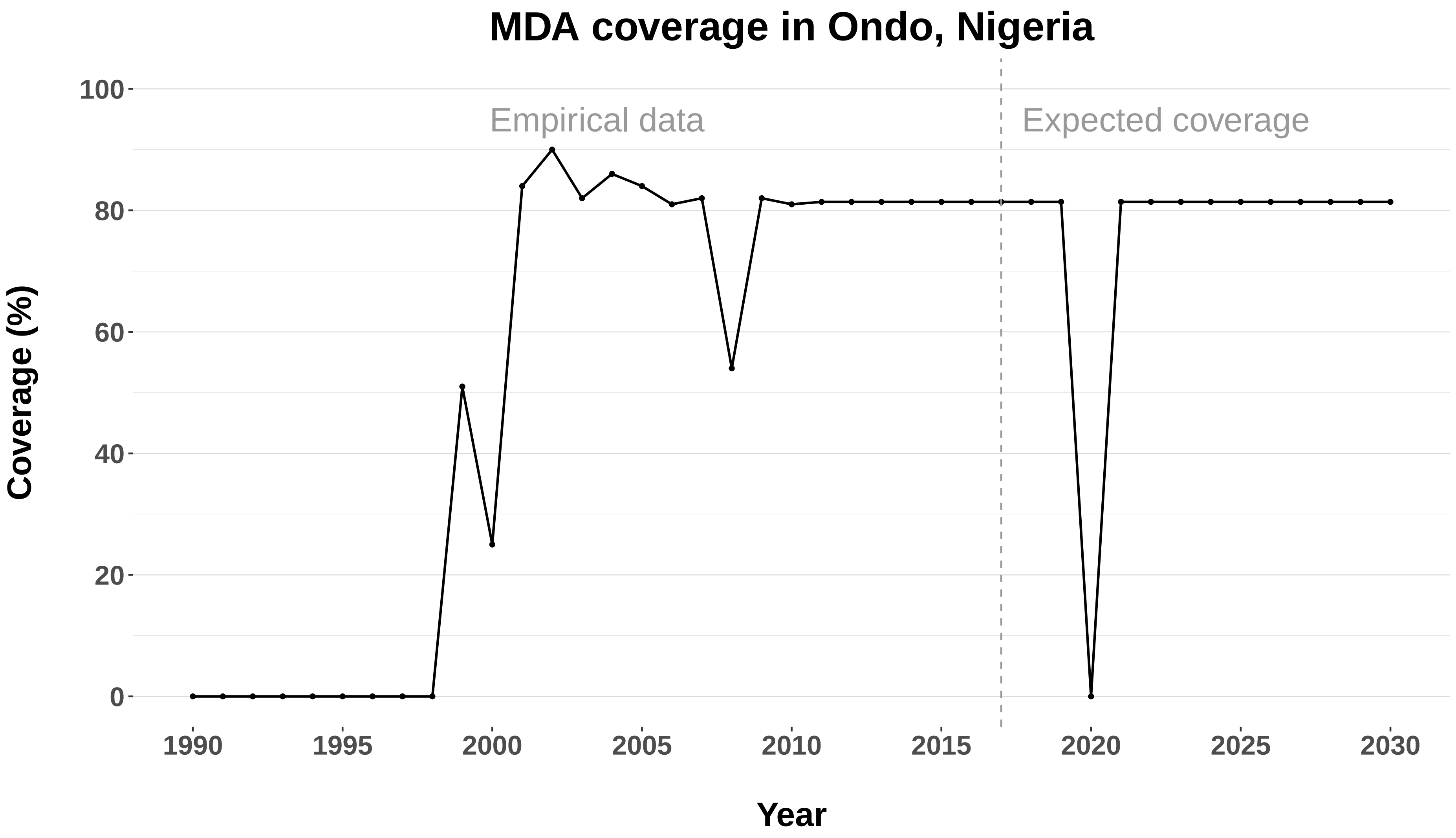
MDA coverage in Ondo, Nigeria
Empirical data
Expected coverage
Coverage (%)
100
80
60
40
20
0
1990
1995
2000
2005
2010
2015
2020
2025
2030
Year

**Project, country:**	**Osun, Nigeria**
Bioclime:	
Endemicity level(s):	Mesoendemic
MDA start year:	1998
Mean MDA coverage per year:	63.3

	1990			2020			2030		
	N	%	DALYs	N	%	DALYs	N	%	DALYs
Total populaton at risk	960	100	_	2,167	100	_	2,842	100	_
**Infection:**									
Mf-positive	211	22.0	_	2	0.1	_	<0.5	<0.05	_
Mf-positive (age 5+)	211	22.0	_	2	0.1	_	<0.5	<0.05	_
Worm infected	292	30.5	_	48	2.2	_	1	<0.05	_
**Clinical manifestations:**									
Palpable nodules	29	3.0	<0.5	1	<0.05	<0.5	<0.5	<0.05	<0.5
Reversible skin disease:									
Severe itch	25	2.6	5	<0.5	<0.05	<0.5	<0.5	<0.05	<0.5
Reactive skin disease	16	1.7	1	<0.5	<0.05	<0.5	<0.5	<0.05	<0.5
**Reversible skin disease**	**41**	**4.3**	**5**	**<0.5**	**<0.05**	**<0.5**	**<0.5**	**<0.05**	**<0.5**
Irreversible skin disease:									
Mild depigmentation	7	0.7	<0.5	5	0.2	<0.5	3	0.1	<0.5
Severe depigmentation	5	0.6	<0.5	4	0.2	<0.5	2	0.1	<0.5
Atrophy	<0.5	<0.05	<0.5	<0.5	<0.05	<0.5	<0.5	<0.05	<0.5
Hanging groin	<0.5	<0.05	<0.5	<0.5	<0.05	<0.5	<0.5	<0.05	<0.5
**Irreversible skin disease**	**12**	**1.3**	**<0.5**	**9**	**0.4**	**<0.5**	**5**	**0.2**	**<0.5**
**Total skin disease (OSD)**	**82**	**8.5**	**6**	**10**	**0.5**	**<0.5**	**5**	**0.2**	**<0.5**
Eye disease (OED):									
Visual impairment	<0.5	<0.05	<0.5	<0.5	<0.05	<0.5	<0.5	<0.05	<0.5
Blindness	<0.5	<0.05	<0.5	<0.5	<0.05	<0.5	<0.5	<0.05	<0.5
**Eye disease (OED)**	**<0.5**	**<0.05**	**<0.5**	**<0.5**	**<0.05**	**<0.5**	**<0.5**	**<0.05**	**<0.5**
**Total all manifestations**	**82**	**8.6**	**6**	**10**	**0.5**	**<0.5**	**5**	**0.2**	**<0.5**

Note 1: Population at risk, number of cases (N) and DALYs are presented in thousands.

Note 2: Total skin disease is defined as the sum of the number of cases with palpable nodules, reversible and irreversible skin disease.

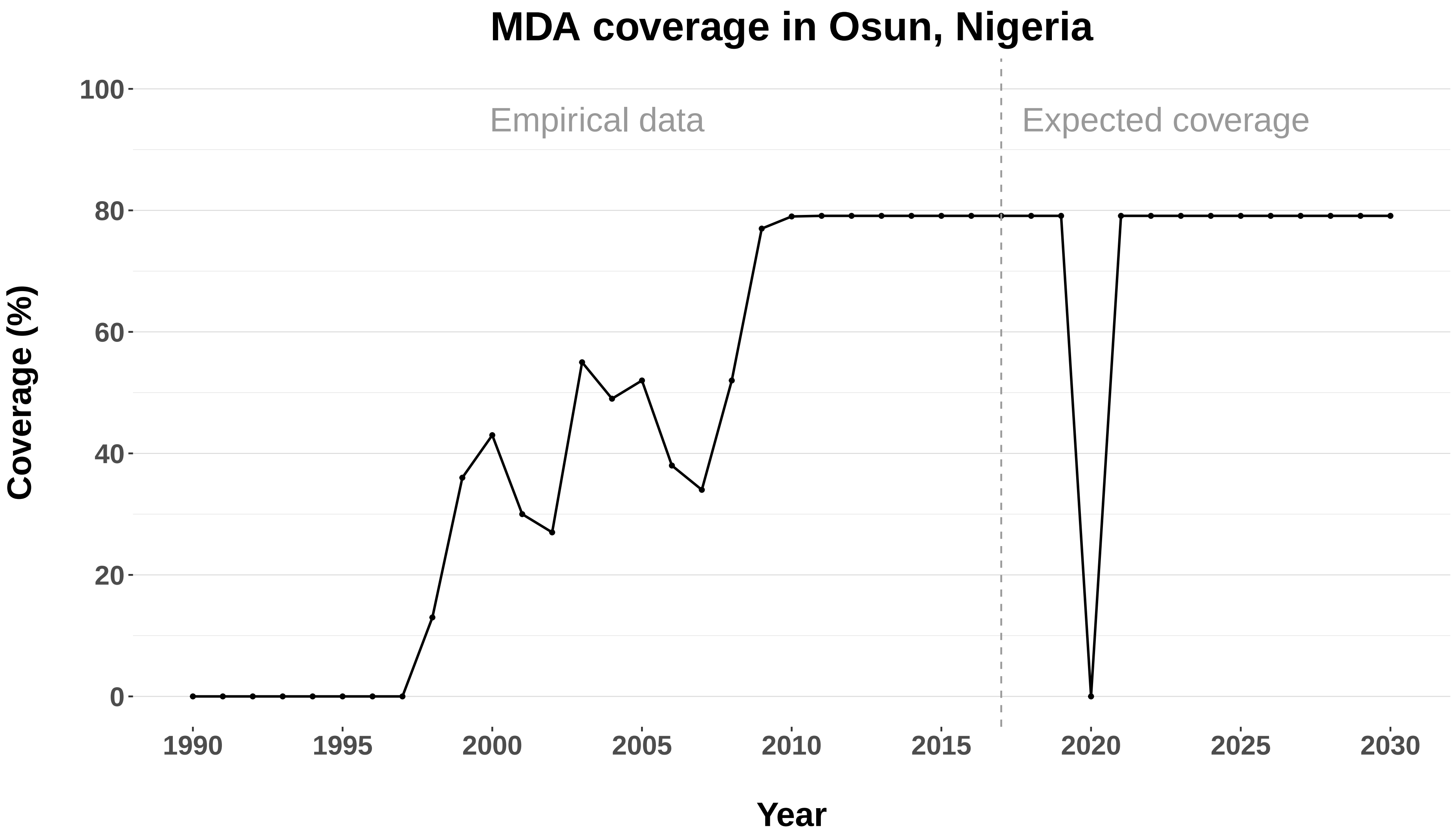
MDA coverage in Osun, Nigeria
Empirical data
Expected coverage
Coverage (%)
100
80
60
40
20
0
1990
1995
2000
2005
2010
2015
2020
2025
2030
Year

**Project, country:**	**Oyo, Nigeria**
Bioclime:	
Endemicity level(s):	Mesoendemic
MDA start year:	1999
Mean MDA coverage per year:	65.4

	1990			2020			2030		
	N	%	DALYs	N	%	DALYs	N	%	DALYs
Total populaton at risk	655	100	_	1,478	100	_	1,938	100	_
**Infection:**									
Mf-positive	149	22.8	_	2	0.1	_	<0.5	<0.05	_
Mf-positive (age 5+)	149	22.8	_	2	0.1	_	<0.5	<0.05	_
Worm infected	208	31.8	_	32	2.1	_	1	<0.05	_
**Clinical manifestations:**									
Palpable nodules	23	3.5	<0.5	1	<0.05	<0.5	<0.5	<0.05	<0.5
Reversible skin disease:									
Severe itch	17	2.6	3	<0.5	<0.05	<0.5	<0.5	<0.05	<0.5
Reactive skin disease	11	1.7	1	<0.5	<0.05	<0.5	<0.5	<0.05	<0.5
**Reversible skin disease**	**28**	**4.3**	**4**	**<0.5**	**<0.05**	**<0.5**	**<0.5**	**<0.05**	**<0.5**
Irreversible skin disease:									
Mild depigmentation	5	0.7	<0.5	3	0.2	<0.5	2	0.1	<0.5
Severe depigmentation	4	0.5	<0.5	2	0.1	<0.5	1	0.1	<0.5
Atrophy	<0.5	<0.05	<0.5	<0.5	<0.05	<0.5	<0.5	<0.05	<0.5
Hanging groin	<0.5	<0.05	<0.5	<0.5	<0.05	<0.5	<0.5	<0.05	<0.5
**Irreversible skin disease**	**8**	**1.3**	**<0.5**	**5**	**0.3**	**<0.5**	**3**	**0.2**	**<0.5**
**Total skin disease (OSD)**	**59**	**9.1**	**4**	**6**	**0.4**	**<0.5**	**3**	**0.2**	**<0.5**
Eye disease (OED):									
Visual impairment	8	1.1	<0.5	5	0.3	<0.5	3	0.1	<0.5
Blindness	2	0.3	15	1	<0.05	5	<0.5	<0.05	2
**Eye disease (OED)**	**10**	**1.5**	**15**	**6**	**0.4**	**5**	**3**	**0.2**	**2**
**Total all manifestations**	**69**	**10.5**	**19**	**11**	**0.8**	**5**	**6**	**0.3**	**2**

Note 1: Population at risk, number of cases (N) and DALYs are presented in thousands.

Note 2: Total skin disease is defined as the sum of the number of cases with palpable nodules, reversible and irreversible skin disease.

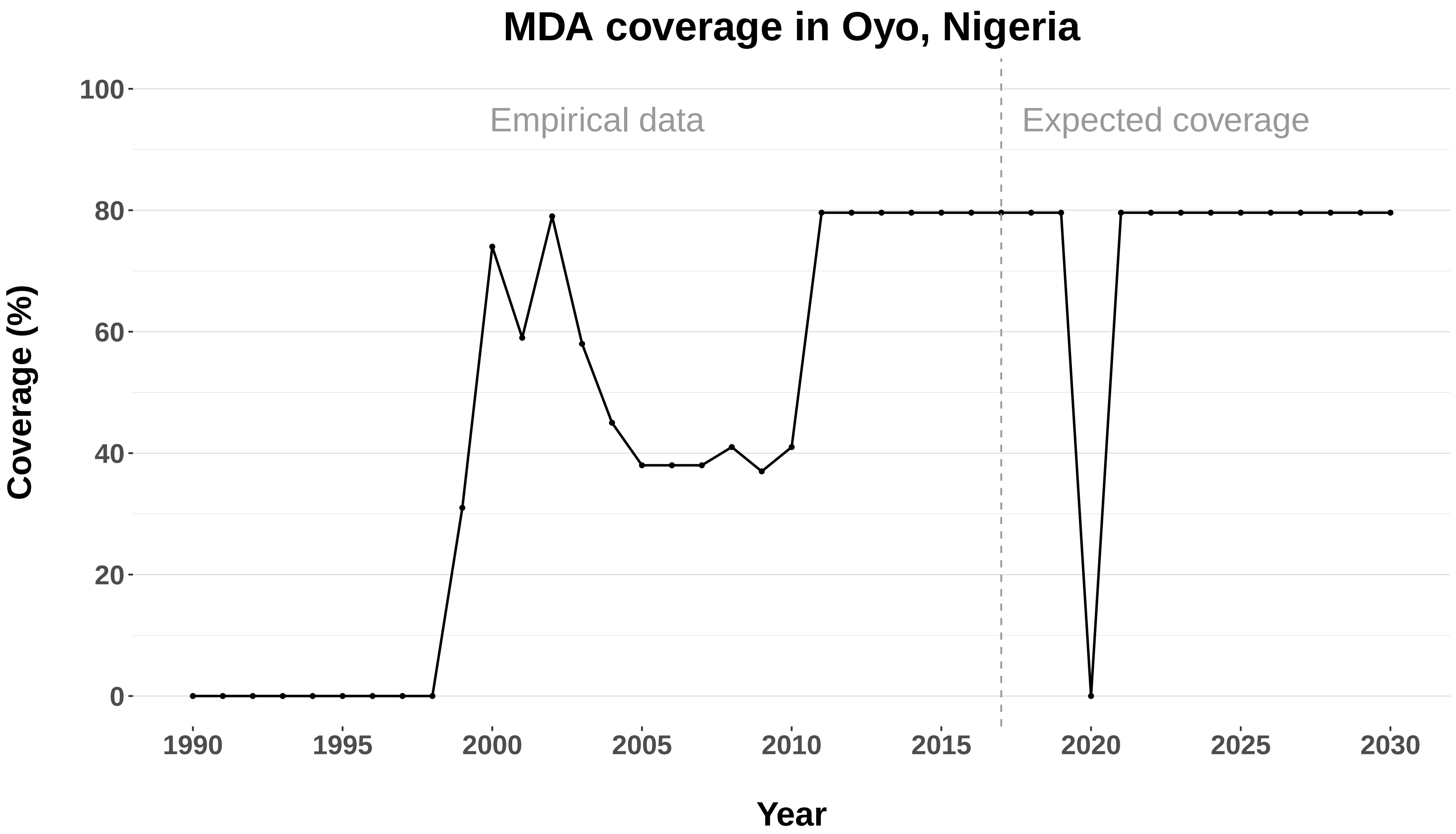
MDA coverage in Oyo, Nigeria
Empirical data
Expected coverage
Coverage (%)
Year
100
80
60
40
20
0
1990
1995
2000
2005
2010
2015
2020
2025
2030

**Project, country:**	**P20Nigeria, Nigeria**
Bioclime:	
Endemicity level(s):	Hyperendemic
MDA start year:	2019
Mean MDA coverage per year:	73.0

	1990			2020			2030		
	N	%	DALYs	N	%	DALYs	N	%	DALYs
Total populaton at risk	132	100	_	298	100	_	391	100	_
**Infection:**									
Mf-positive	54	41.0	_	115	38.5	_	23	6.0	_
Mf-positive (age 5+)	54	41.0	_	115	38.5	_	23	6.0	_
Worm infected	68	51.4	_	153	51.1	_	91	23.3	_
**Clinical manifestations:**									
Palpable nodules	17	12.9	<0.5	38	12.8	<0.5	9	2.2	<0.5
Reversible skin disease:									
Severe itch	8	6.0	1	15	5.1	3	2	0.5	<0.5
Reactive skin disease	6	4.3	<0.5	9	3.0	<0.5	<0.5	<0.05	<0.5
**Reversible skin disease**	**14**	**10.3**	**2**	**24**	**8.1**	**3**	**2**	**0.5**	**<0.5**
Irreversible skin disease:									
Mild depigmentation	2	1.4	<0.5	4	1.3	<0.5	3	0.8	<0.5
Severe depigmentation	2	1.3	<0.5	4	1.3	<0.5	3	0.7	<0.5
Atrophy	<0.5	<0.05	<0.5	<0.5	<0.05	<0.5	<0.5	<0.05	<0.5
Hanging groin	<0.5	<0.05	<0.5	<0.5	<0.05	<0.5	<0.5	<0.05	<0.5
**Irreversible skin disease**	**4**	**2.7**	**<0.5**	**8**	**2.6**	**<0.5**	**6**	**1.6**	**<0.5**
**Total skin disease (OSD)**	**34**	**25.9**	**2**	**70**	**23.5**	**4**	**17**	**4.3**	**1**
Eye disease (OED):									
Visual impairment	<0.5	<0.05	<0.5	<0.5	<0.05	<0.5	<0.5	<0.05	<0.5
Blindness	<0.5	<0.05	<0.5	<0.5	<0.05	<0.5	<0.5	<0.05	<0.5
**Eye disease (OED)**	**<0.5**	**0.1**	**<0.5**	**<0.5**	**0.1**	**<0.5**	**<0.5**	**<0.05**	**<0.5**
**Total all manifestations**	**34**	**26.0**	**2**	**70**	**23.6**	**4**	**17**	**4.3**	**1**

Note 1: Population at risk, number of cases (N) and DALYs are presented in thousands.

Note 2: Total skin disease is defined as the sum of the number of cases with palpable nodules, reversible and irreversible skin disease.

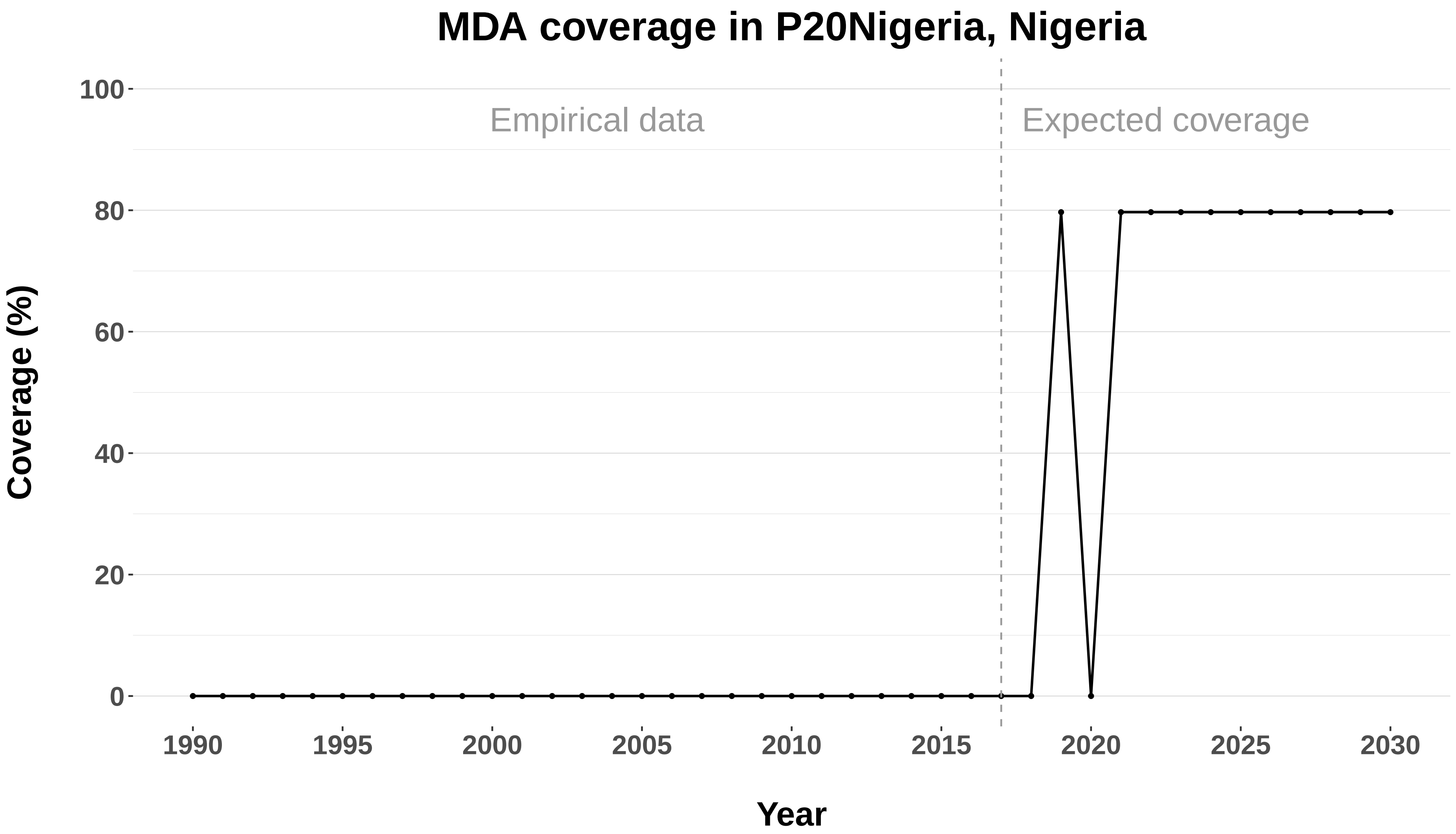
MDA coverage in P20Nigeria, Nigeria
Empirical data
Expected coverage
Coverage (%)
100
80
60
40
20
0
1990
1995
2000
2005
2010
2015
2020
2025
2030
Year

**Project, country:**	**P5Nigeria, Nigeria**
Bioclime:	
Endemicity level(s):	Hypoendemic
MDA start year:	2025
Mean MDA coverage per year:	65.0

	1990			2020			2030		
	N	%	DALYs	N	%	DALYs	N	%	DALYs
Total populaton at risk	4,491	100	_	10,135	100	_	13,294	100	_
**Infection:**									
Mf-positive	930	20.7	_	1,892	18.7	_	1,450	10.9	_
Mf-positive (age 5+)	929	20.7	_	1,891	18.7	_	1,450	10.9	_
Worm infected	1,288	28.7	_	2,665	26.3	_	2,781	20.9	_
**Clinical manifestations:**									
Palpable nodules	105	2.3	1	210	2.1	2	178	1.3	2
Reversible skin disease:									
Severe itch	111	2.5	20	218	2.2	40	117	0.9	21
Reactive skin disease	72	1.6	3	140	1.4	6	30	0.2	1
**Reversible skin disease**	**183**	**4.1**	**23**	**358**	**3.5**	**46**	**147**	**1.1**	**23**
Irreversible skin disease:									
Mild depigmentation	30	0.7	<0.5	60	0.6	1	62	0.5	1
Severe depigmentation	23	0.5	1	49	0.5	3	49	0.4	3
Atrophy	<0.5	<0.05	<0.5	<0.5	<0.05	<0.5	<0.5	<0.05	<0.5
Hanging groin	<0.5	<0.05	<0.5	<0.5	<0.05	<0.5	<0.5	<0.05	<0.5
**Irreversible skin disease**	**54**	**1.2**	**2**	**109**	**1.1**	**4**	**112**	**0.8**	**4**
**Total skin disease (OSD)**	**341**	**7.6**	**26**	**677**	**6.7**	**52**	**436**	**3.3**	**28**
Eye disease (OED):									
Visual impairment	49	1.1	2	103	1.0	3	103	0.8	3
Blindness	14	0.3	94	28	0.3	194	23	0.2	161
**Eye disease (OED)**	**62**	**1.4**	**95**	**131**	**1.3**	**197**	**126**	**1.0**	**164**
**Total all manifestations**	**404**	**9.0**	**122**	**808**	**8.0**	**249**	**563**	**4.2**	**192**

Note 1: Population at risk, number of cases (N) and DALYs are presented in thousands.

Note 2: Total skin disease is defined as the sum of the number of cases with palpable nodules, reversible and irreversible skin disease.

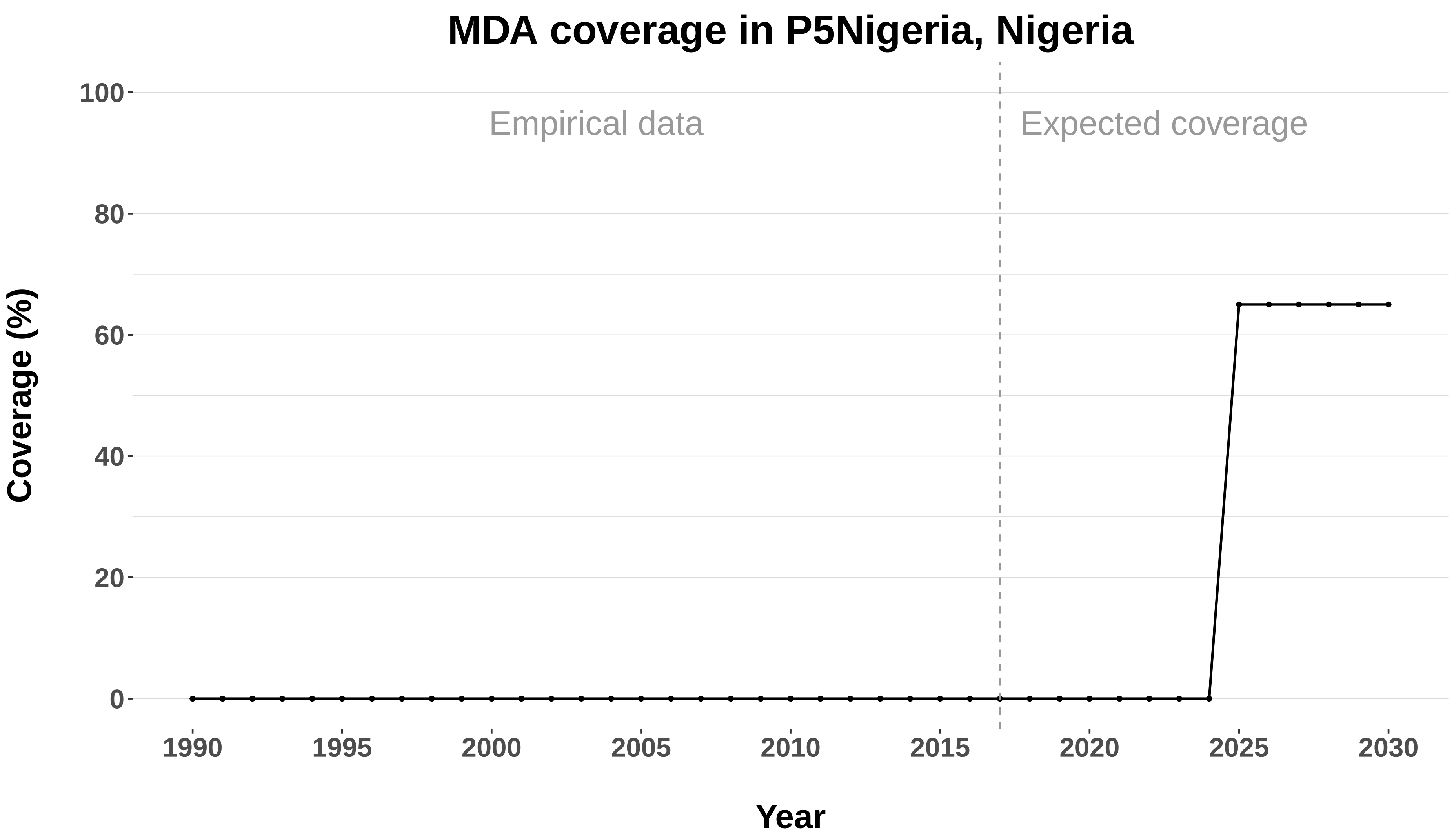
MDA coverage in P5Nigeria, Nigeria
Empirical data
Expected coverage
Coverage (%)
0
20
40
60
80
100
1990
1995
2000
2005
2010
2015
2020
2025
2030
Year

**Project, country:**	**Plateau Nassarawa, Nigeria**
Bioclime:	
Endemicity level(s):	Mesoendemic
MDA start year:	1998
Mean MDA coverage per year:	72.7

	1990			2020			2030		
	N	%	DALYs	N	%	DALYs	N	%	DALYs
Total populaton at risk	911	100	_	2,055	100	_	2,695	100	_
**Infection:**									
Mf-positive	227	24.9	_	<0.5	<0.05	_	<0.5	<0.05	_
Mf-positive (age 5+)	227	24.9	_	<0.5	<0.05	_	<0.5	<0.05	_
Worm infected	301	33.0	_	13	0.6	_	<0.5	<0.05	_
**Clinical manifestations:**									
Palpable nodules	35	3.8	<0.5	<0.5	<0.05	<0.5	<0.5	<0.05	<0.5
Reversible skin disease:									
Severe itch	29	3.2	5	<0.5	<0.05	<0.5	<0.5	<0.05	<0.5
Reactive skin disease	19	2.1	1	<0.5	<0.05	<0.5	<0.5	<0.05	<0.5
**Reversible skin disease**	**48**	**5.3**	**6**	**<0.5**	**<0.05**	**<0.5**	**<0.5**	**<0.05**	**<0.5**
Irreversible skin disease:									
Mild depigmentation	7	0.8	<0.5	5	0.3	<0.5	3	0.1	<0.5
Severe depigmentation	6	0.7	<0.5	4	0.2	<0.5	2	0.1	<0.5
Atrophy	<0.5	<0.05	<0.5	<0.5	<0.05	<0.5	<0.5	<0.05	<0.5
Hanging groin	<0.5	<0.05	<0.5	<0.5	<0.05	<0.5	<0.5	<0.05	<0.5
**Irreversible skin disease**	**13**	**1.5**	**<0.5**	**9**	**0.4**	**<0.5**	**6**	**0.2**	**<0.5**
**Total skin disease (OSD)**	**96**	**10.5**	**7**	**9**	**0.4**	**<0.5**	**6**	**0.2**	**<0.5**
Eye disease (OED):									
Visual impairment	13	1.4	<0.5	8	0.4	<0.5	5	0.2	<0.5
Blindness	4	0.4	28	1	<0.05	7	<0.5	<0.05	3
**Eye disease (OED)**	**17**	**1.9**	**28**	**9**	**0.4**	**7**	**5**	**0.2**	**3**
**Total all manifestations**	**113**	**12.4**	**35**	**18**	**0.9**	**7**	**11**	**0.4**	**3**

Note 1: Population at risk, number of cases (N) and DALYs are presented in thousands.

Note 2: Total skin disease is defined as the sum of the number of cases with palpable nodules, reversible and irreversible skin disease.

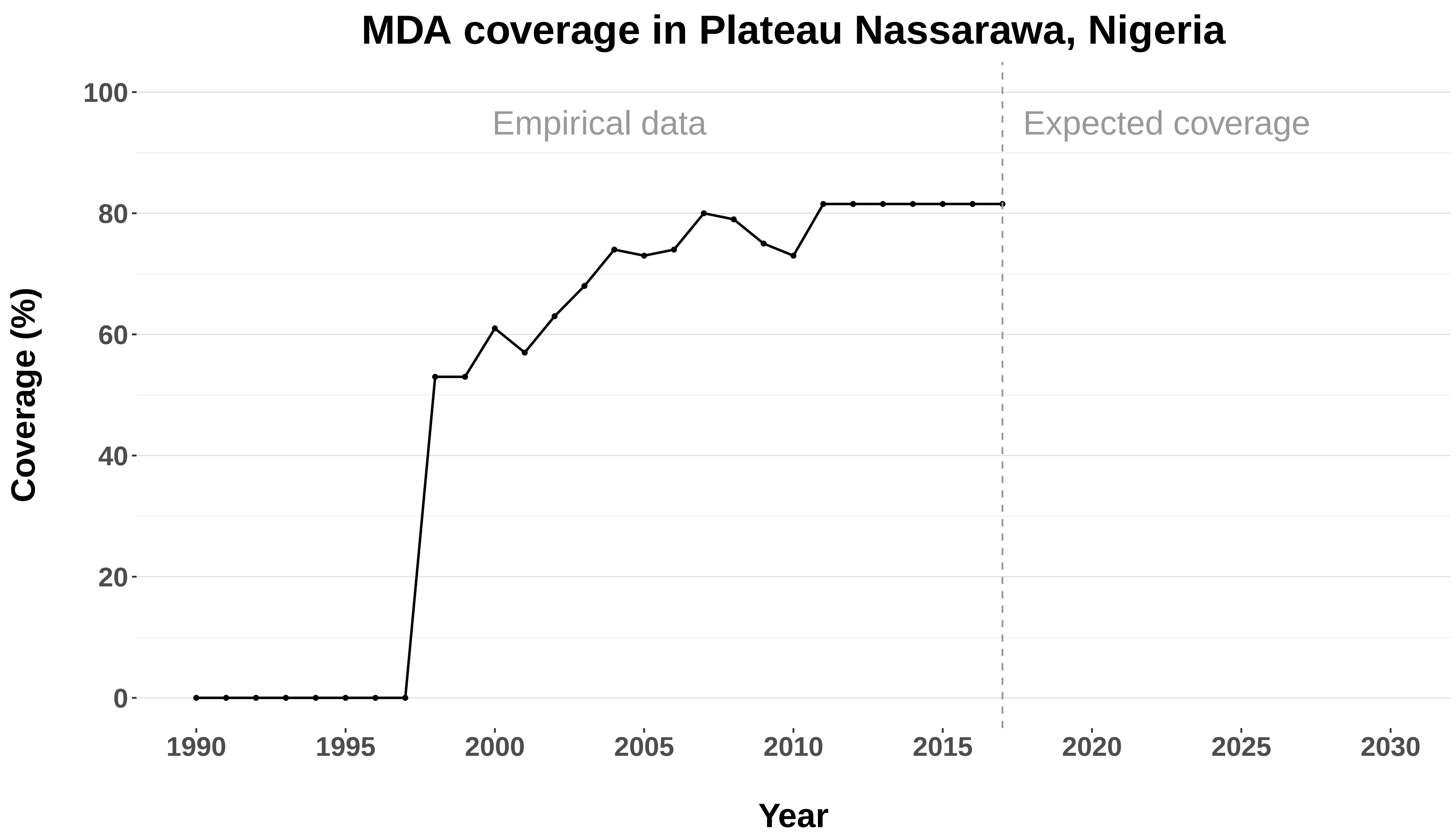

Note: We assume no ivermectin MDA for Plateau and Nassarawa since 2018 as onchocerciasis transmission has been interrupted.[4][‡, page 354].

**Project, country:**	**Plateau Nassarawa LF, Nigeria**
Bioclime:	
Endemicity level(s):	Hyperendemic
MDA start year:	2000
Mean MDA coverage per year:	81.5

	1990			2020			2030		
	N	%	DALYs	N	%	DALYs	N	%	DALYs
Total populaton at risk	922	100	_	2,080	100	_	2,729	100	_
**Infection:**									
Mf-positive	279	30.3	_	<0.5	<0.05	_	<0.5	<0.05	_
Mf-positive (age 5+)	279	30.3	_	<0.5	<0.05	_	<0.5	<0.05	_
Worm infected	367	39.8	_	14	0.7	_	<0.5	<0.05	_
**Clinical manifestations:**									
Palpable nodules	66	7.2	1	<0.5	<0.05	<0.5	<0.5	<0.05	<0.5
Reversible skin disease:									
Severe itch	37	4.0	7	<0.5	<0.05	<0.5	<0.5	<0.05	<0.5
Reactive skin disease	25	2.7	1	<0.5	<0.05	<0.5	<0.5	<0.05	<0.5
**Reversible skin disease**	**62**	**6.8**	**8**	**<0.5**	**<0.05**	**<0.5**	**<0.5**	**<0.05**	**<0.5**
Irreversible skin disease:									
Mild depigmentation	9	1.0	<0.5	7	0.3	<0.5	4	0.1	<0.5
Severe depigmentation	8	0.9	1	5	0.3	<0.5	3	0.1	<0.5
Atrophy	<0.5	<0.05	<0.5	<0.5	<0.05	<0.5	<0.5	<0.05	<0.5
Hanging groin	<0.5	<0.05	<0.5	<0.5	<0.05	<0.5	<0.5	<0.05	<0.5
**Irreversible skin disease**	**17**	**1.9**	**1**	**12**	**0.6**	**<0.5**	**7**	**0.2**	**<0.5**
**Total skin disease (OSD)**	**145**	**15.8**	**9**	**12**	**0.6**	**<0.5**	**7**	**0.2**	**<0.5**
Eye disease (OED):									
Visual impairment	16	1.7	<0.5	11	0.5	<0.5	6	0.2	<0.5
Blindness	5	0.6	39	2	0.1	12	<0.5	<0.05	3
**Eye disease (OED)**	**22**	**2.3**	**40**	**12**	**0.6**	**12**	**7**	**0.2**	**4**
**Total all manifestations**	**167**	**18.1**	**49**	**24**	**1.2**	**13**	**13**	**0.5**	**4**

Note 1: Population at risk, number of cases (N) and DALYs are presented in thousands.

Note 2: Total skin disease is defined as the sum of the number of cases with palpable nodules, reversible and irreversible skin disease.

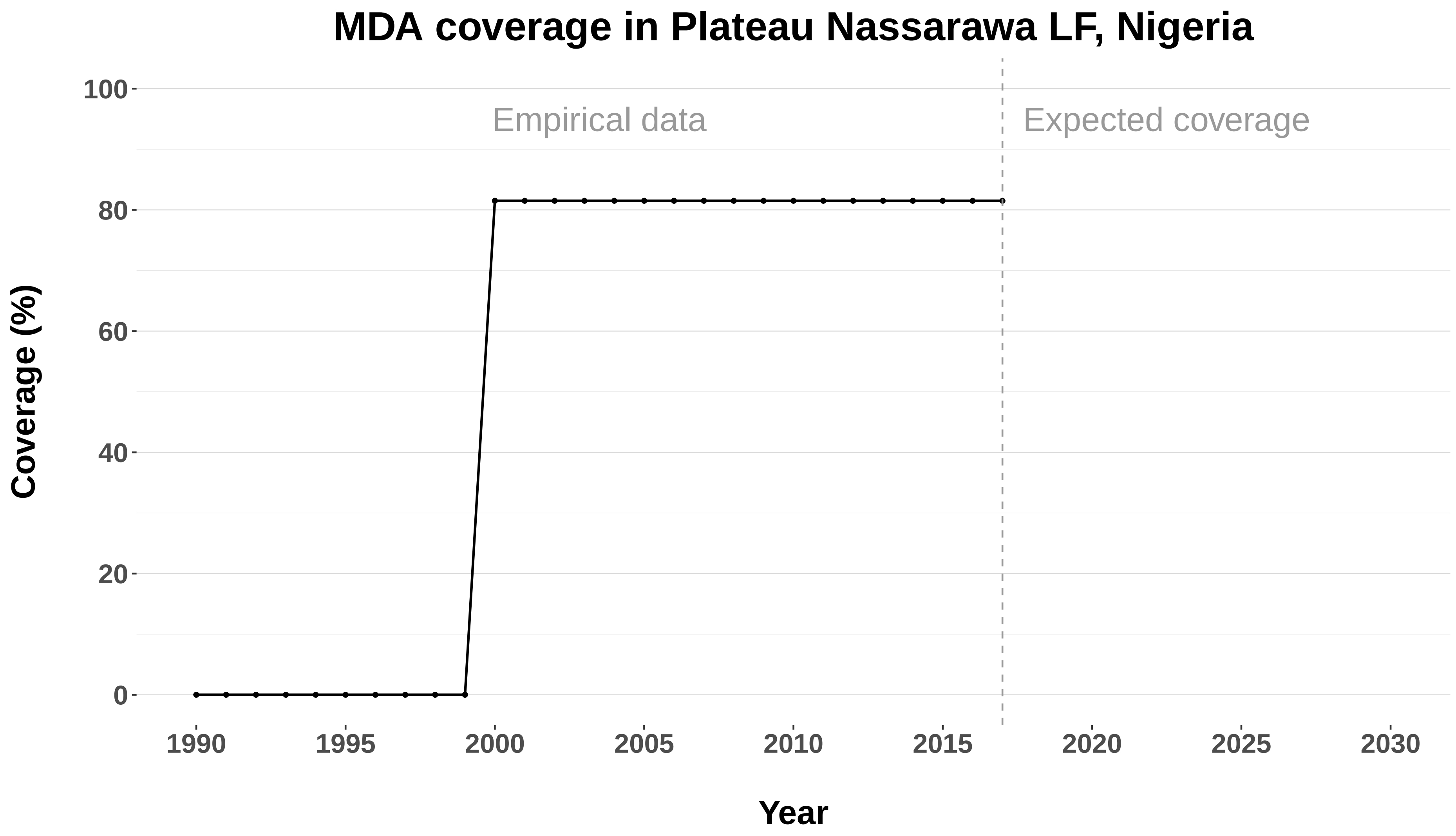

Note: We assume no ivermectin MDA for Plateau and Nassarawa since 2018 as onchocerciasis transmission has been interrupted.[4][‡, page 354].

**Project, country:**	**Taraba, Nigeria**
Bioclime:	
Endemicity level(s):	Hyperendemic
MDA start year:	1998
Mean MDA coverage per year:	69.1

	1990			2020			2030		
	N	%	DALYs	N	%	DALYs	N	%	DALYs
Total populaton at risk	991	100	_	2,236	100	_	2,932	100	_
**Infection:**									
Mf-positive	310	31.2	_	9	0.4	_	1	<0.05	_
Mf-positive (age 5+)	309	31.2	_	9	0.4	_	1	<0.05	_
Worm infected	397	40.1	_	75	3.4	_	6	0.2	_
**Clinical manifestations:**									
Palpable nodules	80	8.0	1	4	0.2	<0.5	<0.5	<0.05	<0.5
Reversible skin disease:									
Severe itch	45	4.5	8	1	<0.05	<0.5	<0.5	<0.05	<0.5
Reactive skin disease	32	3.3	2	<0.5	<0.05	<0.5	<0.5	<0.05	<0.5
**Reversible skin disease**	**77**	**7.8**	**10**	**1**	**<0.05**	**<0.5**	**<0.5**	**<0.05**	**<0.5**
Irreversible skin disease:									
Mild depigmentation	11	1.1	<0.5	8	0.4	<0.5	5	0.2	<0.5
Severe depigmentation	10	1.1	1	7	0.3	<0.5	4	0.1	<0.5
Atrophy	<0.5	<0.05	<0.5	<0.5	<0.05	<0.5	<0.5	<0.05	<0.5
Hanging groin	<0.5	<0.05	<0.5	<0.5	<0.05	<0.5	<0.5	<0.05	<0.5
**Irreversible skin disease**	**22**	**2.2**	**1**	**15**	**0.7**	**1**	**9**	**0.3**	**<0.5**
**Total skin disease (OSD)**	**178**	**18.0**	**11**	**20**	**0.9**	**1**	**10**	**0.3**	**<0.5**
Eye disease (OED):									
Visual impairment	19	2.0	1	14	0.6	<0.5	9	0.3	<0.5
Blindness	8	0.8	61	3	0.1	21	1	<0.05	8
**Eye disease (OED)**	**28**	**2.8**	**62**	**17**	**0.8**	**21**	**10**	**0.3**	**9**
**Total all manifestations**	**206**	**20.8**	**73**	**37**	**1.6**	**22**	**20**	**0.7**	**9**

Note 1: Population at risk, number of cases (N) and DALYs are presented in thousands.

Note 2: Total skin disease is defined as the sum of the number of cases with palpable nodules, reversible and irreversible skin disease.

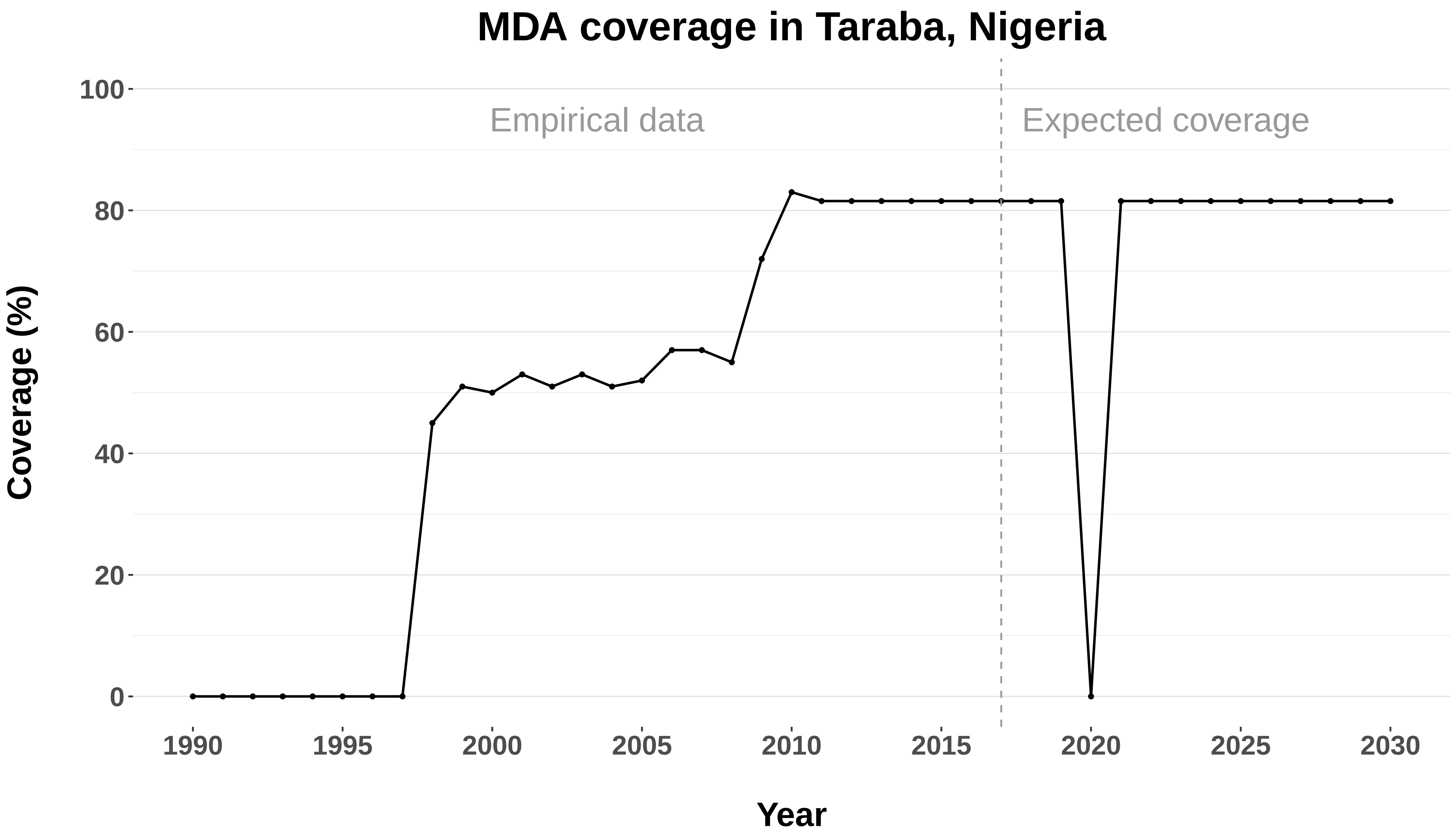
MDA coverage in Taraba, Nigeria
Empirical data
Expected coverage
Coverage (%)
0
20
40
60
80
100
1990
1995
2000
2005
2010
2015
2020
2025
2030
Year

**Project, country:**	**Yobe, Nigeria**
Bioclime:	
Endemicity level(s):	Mesoendemic
MDA start year:	1999
Mean MDA coverage per year:	71.4

	1990			2020			2030		
	N	%	DALYs	N	%	DALYs	N	%	DALYs
Total populaton at risk	366	100	_	827	100	_	1,085	100	_
**Infection:**									
Mf-positive	70	19.1	_	<0.5	<0.05	_	<0.5	<0.05	_
Mf-positive (age 5+)	70	19.1	_	<0.5	<0.05	_	<0.5	<0.05	_
Worm infected	99	27.0	_	5	0.6	_	<0.5	<0.05	_
**Clinical manifestations:**									
Palpable nodules	7	1.9	<0.5	<0.5	<0.05	<0.5	<0.5	<0.05	<0.5
Reversible skin disease:									
Severe itch	8	2.2	1	<0.5	<0.05	<0.5	<0.5	<0.05	<0.5
Reactive skin disease	5	1.4	<0.5	<0.5	<0.05	<0.5	<0.5	<0.05	<0.5
**Reversible skin disease**	**13**	**3.6**	**2**	**<0.5**	**<0.05**	**<0.5**	**<0.5**	**<0.05**	**<0.5**
Irreversible skin disease:									
Mild depigmentation	2	0.6	<0.5	1	0.2	<0.5	1	0.1	<0.5
Severe depigmentation	2	0.5	<0.5	1	0.1	<0.5	<0.5	<0.05	<0.5
Atrophy	<0.5	<0.05	<0.5	<0.5	<0.05	<0.5	<0.5	<0.05	<0.5
Hanging groin	<0.5	<0.05	<0.5	<0.5	<0.05	<0.5	<0.5	<0.05	<0.5
**Irreversible skin disease**	**4**	**1.0**	**<0.5**	**2**	**0.3**	**<0.5**	**1**	**0.1**	**<0.5**
**Total skin disease (OSD)**	**24**	**6.5**	**2**	**2**	**0.3**	**<0.5**	**1**	**0.1**	**<0.5**
Eye disease (OED):									
Visual impairment	4	1.0	<0.5	2	0.3	<0.5	1	0.1	<0.5
Blindness	1	0.3	7	<0.5	<0.05	2	<0.5	<0.05	1
**Eye disease (OED)**	**5**	**1.2**	**7**	**2**	**0.3**	**2**	**1**	**0.1**	**1**
**Total all manifestations**	**28**	**7.7**	**9**	**5**	**0.6**	**2**	**3**	**0.2**	**1**

Note 1: Population at risk, number of cases (N) and DALYs are presented in thousands.

Note 2: Total skin disease is defined as the sum of the number of cases with palpable nodules, reversible and irreversible skin disease.

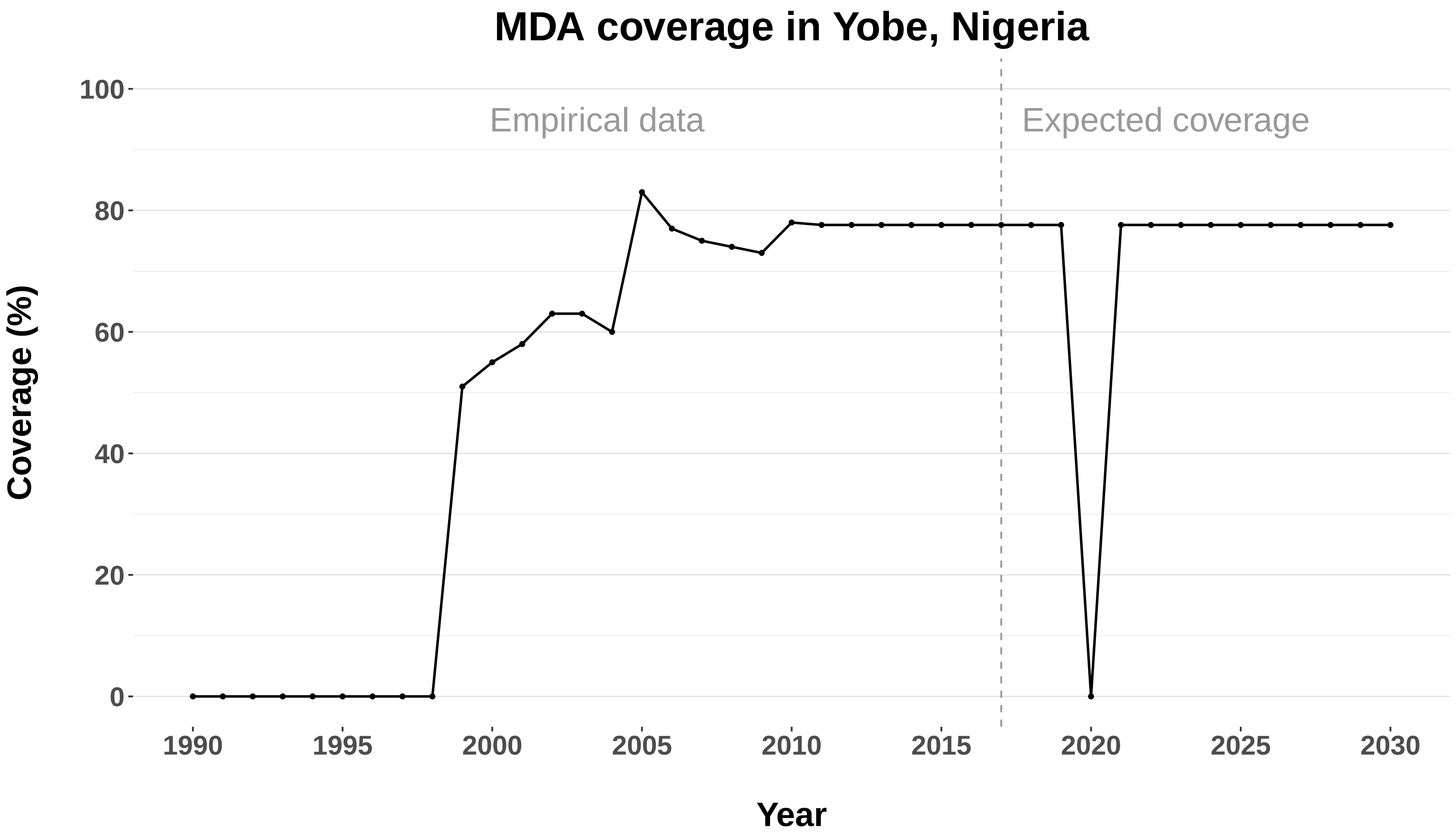

MDA coverage in Yobe, Nigeria
Empirical data
Expected coverage
Coverage (%)
Year
0
20
40
60
80
100
1990
1995
2000
2005
2010
2015
2020
2025
2030

**Project, country:**	**Zamfara, Nigeria**
Bioclime:	
Endemicity level(s):	Hypoendemic
MDA start year:	1999
Mean MDA coverage per year:	73.8

	1990			2020			2030		
	N	%	DALYs	N	%	DALYs	N	%	DALYs
Total populaton at risk	170	100	_	384	100	_	503	100	_
**Infection:**									
Mf-positive	36	21.1	_	<0.5	<0.05	_	<0.5	<0.05	_
Mf-positive (age 5+)	36	21.1	_	<0.5	<0.05	_	<0.5	<0.05	_
Worm infected	49	28.6	_	2	0.5	_	<0.5	<0.05	_
**Clinical manifestations:**									
Palpable nodules	4	2.2	<0.5	<0.5	<0.05	<0.5	<0.5	<0.05	<0.5
Reversible skin disease:									
Severe itch	4	2.5	1	<0.5	<0.05	<0.5	<0.5	<0.05	<0.5
Reactive skin disease	3	1.6	<0.5	<0.5	<0.05	<0.5	<0.5	<0.05	<0.5
**Reversible skin disease**	**7**	**4.2**	**1**	**<0.5**	**<0.05**	**<0.5**	**<0.5**	**<0.05**	**<0.5**
Irreversible skin disease:									
Mild depigmentation	1	0.7	<0.5	1	0.2	<0.5	1	0.1	<0.5
Severe depigmentation	1	0.6	<0.5	1	0.1	<0.5	<0.5	0.1	<0.5
Atrophy	<0.5	<0.05	<0.5	<0.5	<0.05	<0.5	<0.5	<0.05	<0.5
Hanging groin	<0.5	<0.05	<0.5	<0.5	<0.05	<0.5	<0.5	<0.05	<0.5
**Irreversible skin disease**	**2**	**1.2**	**<0.5**	**1**	**0.4**	**<0.5**	**1**	**0.2**	**<0.5**
**Total skin disease (OSD)**	**13**	**7.6**	**1**	**1**	**0.4**	**<0.5**	**1**	**0.2**	**<0.5**
Eye disease (OED):									
Visual impairment	2	1.2	<0.5	1	0.3	<0.5	1	0.1	<0.5
Blindness	1	0.3	4	<0.5	<0.05	1	<0.5	<0.05	<0.5
**Eye disease (OED)**	**3**	**1.5**	**4**	**1**	**0.4**	**1**	**1**	**0.1**	**<0.5**
**Total all manifestations**	**15**	**9.1**	**5**	**3**	**0.7**	**1**	**2**	**0.3**	**<0.5**

Note 1: Population at risk, number of cases (N) and DALYs are presented in thousands.

Note 2: Total skin disease is defined as the sum of the number of cases with palpable nodules, reversible and irreversible skin disease.

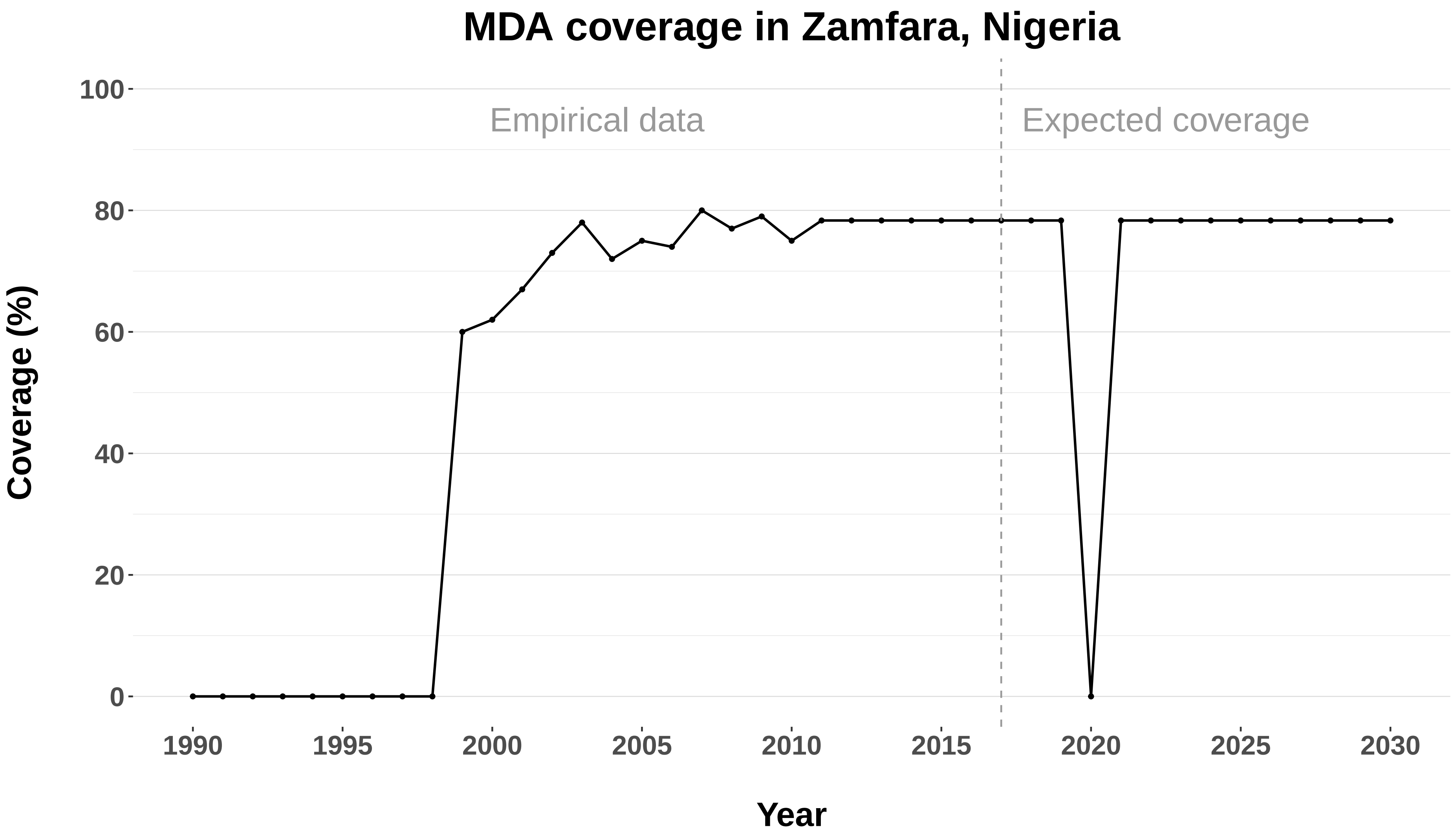

MDA coverage in Zamfara, Nigeria
Empirical data
Expected coverage
Coverage (%)
Year
0
20
40
60
80
100
1990
1995
2000
2005
2010
2015
2020
2025
2030

**Project, country:**	**East Bahr El Ghazal, South Sudan**
Bioclime:	
Endemicity level(s):	Hyperendemic
MDA start year:	2005
Mean MDA coverage per year:	51.3

	1990			2020			2030		
	N	%	DALYs	N	%	DALYs	N	%	DALYs
Total populaton at risk	326	100	_	736	100	_	966	100	_
**Infection:**									
Mf-positive	105	32.1	_	46	6.2	_	4	0.4	_
Mf-positive (age 5+)	105	32.1	_	46	6.2	_	4	0.4	_
Worm infected	138	42.3	_	125	17.0	_	30	3.1	_
**Clinical manifestations:**									
Palpable nodules	27	8.3	<0.5	10	1.3	<0.5	1	0.1	<0.5
Reversible skin disease:									
Severe itch	14	4.3	3	3	0.4	1	<0.5	<0.05	<0.5
Reactive skin disease	10	2.9	<0.5	1	0.1	<0.5	<0.5	<0.05	<0.5
**Reversible skin disease**	**24**	**7.2**	**3**	**4**	**0.5**	**1**	**<0.5**	**<0.05**	**<0.5**
Irreversible skin disease:									
Mild depigmentation	3	1.0	<0.5	4	0.5	<0.5	3	0.3	<0.5
Severe depigmentation	3	0.9	<0.5	3	0.4	<0.5	2	0.2	<0.5
Atrophy	<0.5	<0.05	<0.5	<0.5	<0.05	<0.5	<0.5	<0.05	<0.5
Hanging groin	<0.5	<0.05	<0.5	<0.5	<0.05	<0.5	<0.5	<0.05	<0.5
**Irreversible skin disease**	**6**	**2.0**	**<0.5**	**7**	**0.9**	**<0.5**	**5**	**0.5**	**<0.5**
**Total skin disease (OSD)**	**57**	**17.5**	**4**	**20**	**2.8**	**1**	**6**	**0.6**	**<0.5**
Eye disease (OED):									
Visual impairment	6	1.8	<0.5	7	0.9	<0.5	4	0.5	<0.5
Blindness	2	0.6	15	2	0.2	11	1	0.1	5
**Eye disease (OED)**	**8**	**2.4**	**15**	**8**	**1.1**	**11**	**5**	**0.5**	**5**
**Total all manifestations**	**65**	**19.9**	**19**	**29**	**3.9**	**12**	**11**	**1.2**	**5**

Note 1: Population at risk, number of cases (N) and DALYs are presented in thousands.

Note 2: Total skin disease is defined as the sum of the number of cases with palpable nodules, reversible and irreversible skin disease.

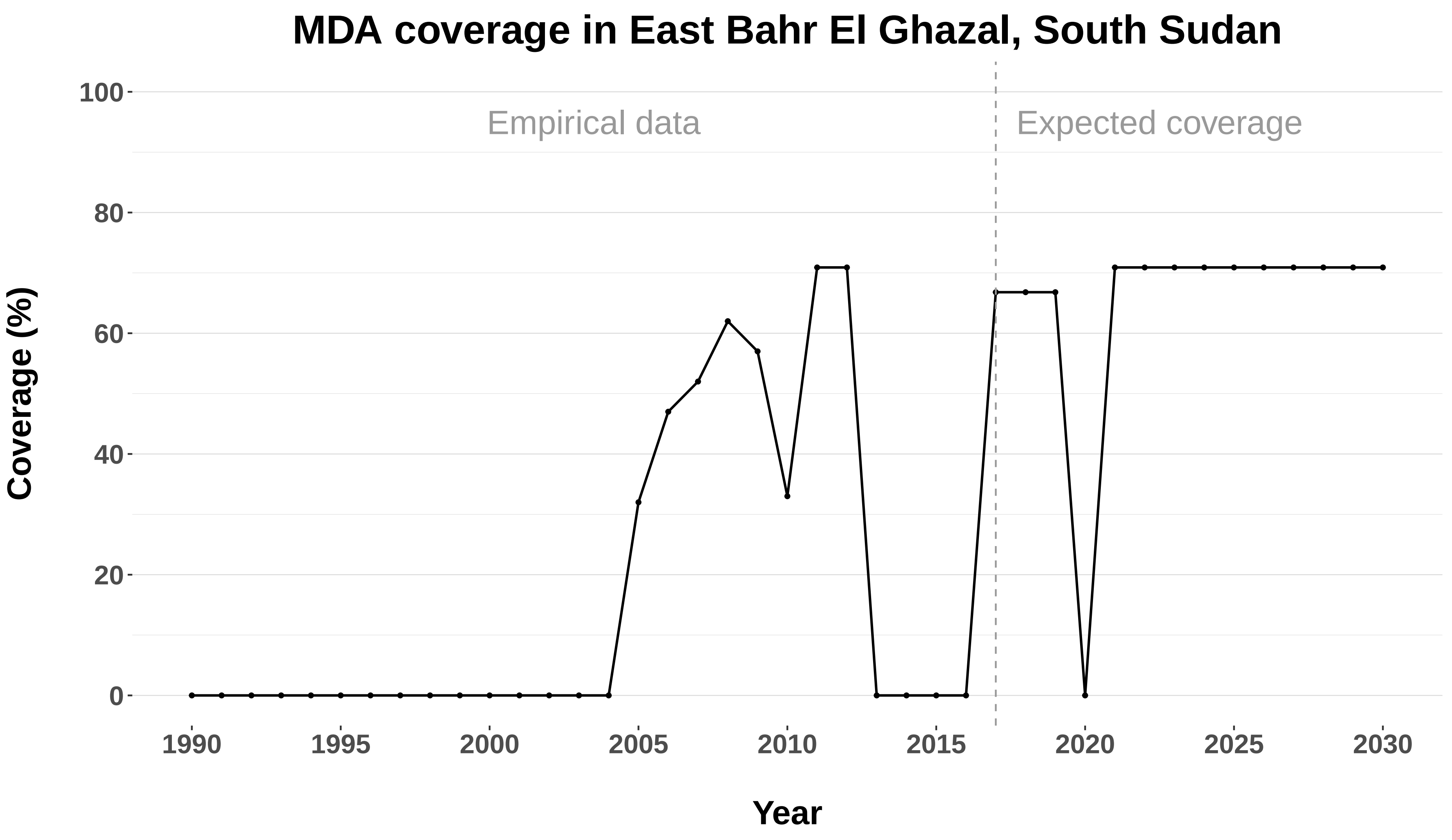
MDA coverage in East Bahr El Ghazal, South Sudan
Empirical data
Expected coverage
Coverage (%)
100
80
60
40
20
0
1990
1995
2000
2005
2010
2015
2020
2025
2030
Year

**Project, country:**	**East Equatoria, South Sudan**
Bioclime:	
Endemicity level(s):	Hyperendemic
MDA start year:	2006
Mean MDA coverage per year:	41.3

	1990			2020			2030		
	N	%	DALYs	N	%	DALYs	N	%	DALYs
Total populaton at risk	580	100	_	1,309	100	_	1,716	100	_
**Infection:**									
Mf-positive	180	31.0	_	143	10.9	_	26	1.5	_
Mf-positive (age 5+)	180	31.0	_	143	10.9	_	26	1.5	_
Worm infected	236	40.8	_	275	21.0	_	133	7.7	_
**Clinical manifestations:**									
Palpable nodules	44	7.6	<0.5	24	1.8	<0.5	7	0.4	<0.5
Reversible skin disease:									
Severe itch	24	4.1	4	10	0.8	2	1	0.1	<0.5
Reactive skin disease	16	2.8	1	5	0.4	<0.5	<0.5	<0.05	<0.5
**Reversible skin disease**	**40**	**6.9**	**5**	**15**	**1.2**	**2**	**1**	**0.1**	**<0.5**
Irreversible skin disease:									
Mild depigmentation	6	1.0	<0.5	8	0.6	<0.5	6	0.3	<0.5
Severe depigmentation	5	0.9	<0.5	6	0.5	<0.5	4	0.2	<0.5
Atrophy	<0.5	<0.05	<0.5	<0.5	<0.05	<0.5	<0.5	<0.05	<0.5
Hanging groin	<0.5	<0.05	<0.5	<0.5	<0.05	<0.5	<0.5	<0.05	<0.5
**Irreversible skin disease**	**11**	**1.9**	**<0.5**	**14**	**1.1**	**<0.5**	**10**	**0.6**	**<0.5**
**Total skin disease (OSD)**	**95**	**16.4**	**6**	**53**	**4.0**	**3**	**18**	**1.1**	**1**
Eye disease (OED):									
Visual impairment	10	1.8	<0.5	13	1.0	<0.5	9	0.5	<0.5
Blindness	4	0.6	26	3	0.2	23	1	0.1	10
**Eye disease (OED)**	**14**	**2.4**	**26**	**16**	**1.2**	**23**	**11**	**0.6**	**11**
**Total all manifestations**	**109**	**18.8**	**32**	**69**	**5.2**	**26**	**29**	**1.7**	**11**

Note 1: Population at risk, number of cases (N) and DALYs are presented in thousands.

Note 2: Total skin disease is defined as the sum of the number of cases with palpable nodules, reversible and irreversible skin disease.

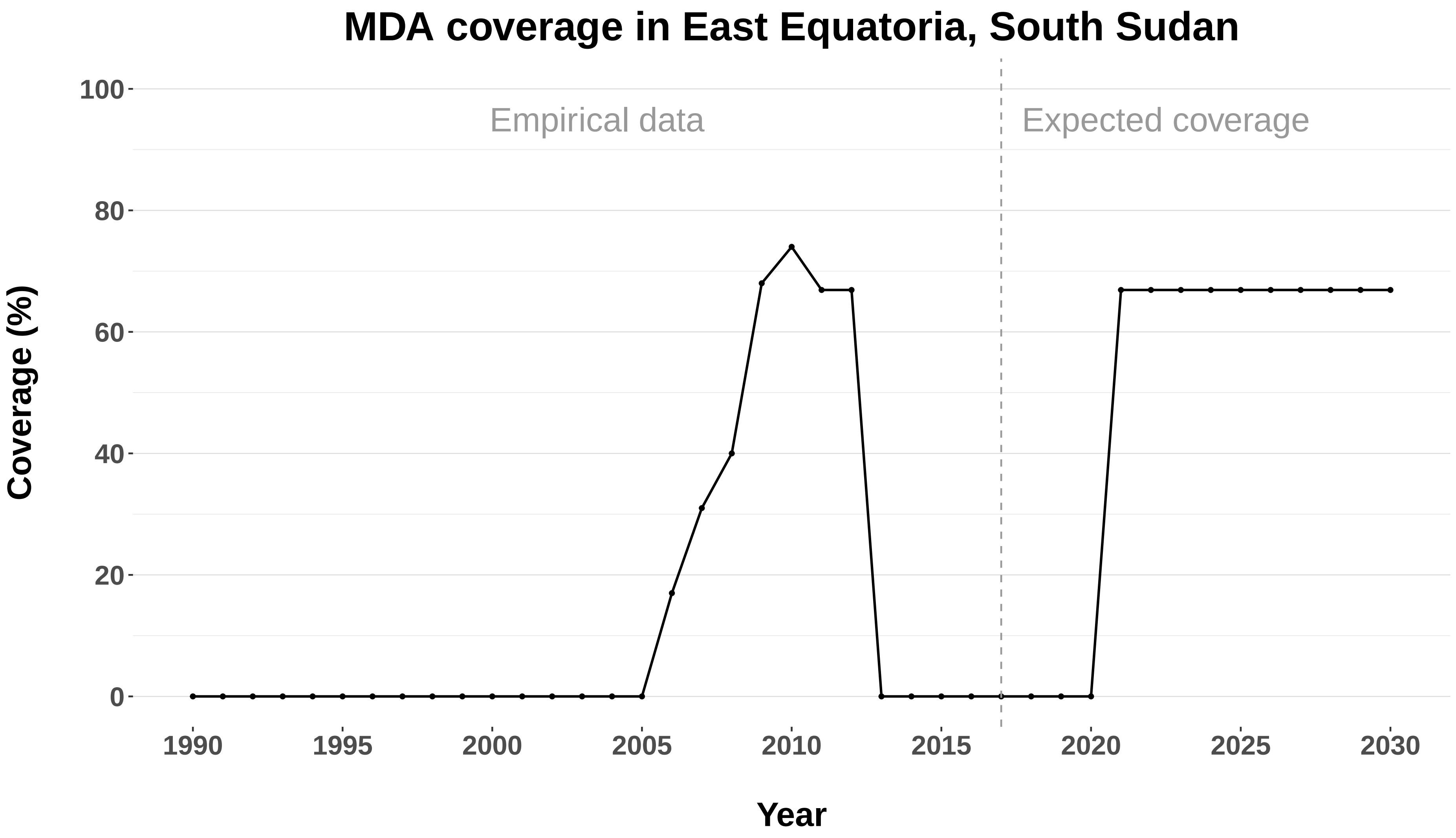
MDA coverage in East Equatoria, South Sudan
Empirical data
Expected coverage
Coverage (%)
100
80
60
40
20
0
1990
1995
2000
2005
2010
2015
2020
2025
2030
Year

**Project, country:**	**P20SouthSudan, South Sudan**
Bioclime:	
Endemicity level(s):	Hyperendemic
MDA start year:	2020
Mean MDA coverage per year:	54.5

	1990			2020			2030		
	N	%	DALYs	N	%	DALYs	N	%	DALYs
Total populaton at risk	16	100	_	37	100	_	48	100	_
**Infection:**									
Mf-positive	6	37.7	_	13	36.5	_	7	13.6	_
Mf-positive (age 5+)	6	37.6	_	13	36.5	_	7	13.6	_
Worm infected	8	48.1	_	17	46.9	_	15	30.0	_
**Clinical manifestations:**									
Palpable nodules	2	10.8	<0.5	4	10.6	<0.5	2	3.9	<0.5
Reversible skin disease:									
Severe itch	1	5.2	<0.5	2	5.0	<0.5	1	1.1	<0.5
Reactive skin disease	1	3.6	<0.5	1	3.5	<0.5	<0.5	0.3	<0.5
**Reversible skin disease**	**1**	**8.8**	**<0.5**	**3**	**8.5**	**<0.5**	**1**	**1.3**	**<0.5**
Irreversible skin disease:									
Mild depigmentation	<0.5	1.2	<0.5	<0.5	1.2	<0.5	<0.5	0.8	<0.5
Severe depigmentation	<0.5	1.1	<0.5	<0.5	1.1	<0.5	<0.5	0.7	<0.5
Atrophy	<0.5	<0.05	<0.5	<0.5	<0.05	<0.5	<0.5	<0.05	<0.5
Hanging groin	<0.5	<0.05	<0.5	<0.5	<0.05	<0.5	<0.5	<0.05	<0.5
**Irreversible skin disease**	**<0.5**	**2.3**	**<0.5**	**1**	**2.2**	**<0.5**	**1**	**1.5**	**<0.5**
**Total skin disease (OSD)**	**4**	**22.0**	**<0.5**	**8**	**21.3**	**<0.5**	**3**	**6.7**	**<0.5**
Eye disease (OED):									
Visual impairment	<0.5	2.2	<0.5	1	2.1	<0.5	1	1.4	<0.5
Blindness	<0.5	0.8	1	<0.5	0.8	2	<0.5	0.4	1
**Eye disease (OED)**	**<0.5**	**3.0**	**1**	**1**	**2.9**	**2**	**1**	**1.8**	**1**
**Total all manifestations**	**4**	**25.0**	**1**	**9**	**24.2**	**3**	**4**	**8.5**	**2**

Note 1: Population at risk, number of cases (N) and DALYs are presented in thousands.

Note 2: Total skin disease is defined as the sum of the number of cases with palpable nodules, reversible and irreversible skin disease.

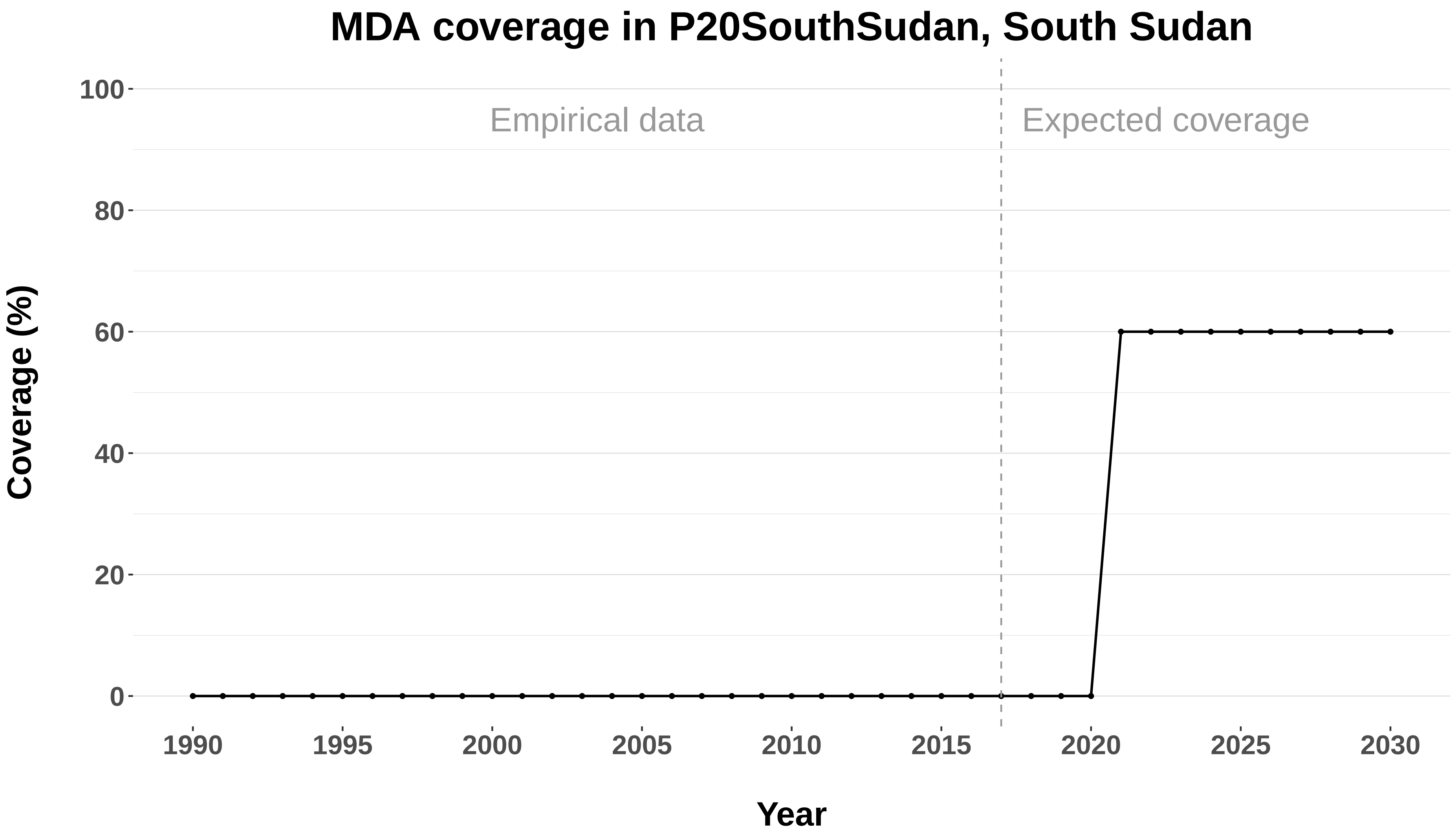
MDA coverage in P20SouthSudan, South Sudan
Empirical data
Expected coverage
Coverage (%)
100
80
60
40
20
0
1990
1995
2000
2005
2010
2015
2020
2025
2030
Year

**Project, country:**	**P5SouthSudan, South Sudan**
Bioclime:	
Endemicity level(s):	Hypoendemic
MDA start year:	2025
Mean MDA coverage per year:	65.0

	1990			2020			2030		
	N	%	DALYs	N	%	DALYs	N	%	DALYs
Total populaton at risk	450	100	_	1,015	100	_	1,331	100	_
**Infection:**									
Mf-positive	91	20.2	_	183	18.0	_	141	10.6	_
Mf-positive (age 5+)	91	20.2	_	182	18.0	_	141	10.6	_
Worm infected	127	28.2	_	262	25.8	_	272	20.4	_
**Clinical manifestations:**									
Palpable nodules	10	2.2	<0.5	20	1.9	<0.5	17	1.3	<0.5
Reversible skin disease:									
Severe itch	11	2.4	2	21	2.1	4	11	0.8	2
Reactive skin disease	7	1.5	<0.5	13	1.3	1	3	0.2	<0.5
**Reversible skin disease**	**17**	**3.9**	**2**	**34**	**3.4**	**4**	**14**	**1.1**	**2**
Irreversible skin disease:									
Mild depigmentation	3	0.6	<0.5	6	0.6	<0.5	6	0.5	<0.5
Severe depigmentation	2	0.5	<0.5	5	0.5	<0.5	5	0.3	<0.5
Atrophy	<0.5	<0.05	<0.5	<0.5	<0.05	<0.5	<0.5	<0.05	<0.5
Hanging groin	<0.5	<0.05	<0.5	<0.5	<0.05	<0.5	<0.5	<0.05	<0.5
**Irreversible skin disease**	**5**	**1.1**	**<0.5**	**11**	**1.1**	**<0.5**	**11**	**0.8**	**<0.5**
**Total skin disease (OSD)**	**33**	**7.3**	**3**	**65**	**6.4**	**5**	**42**	**3.1**	**3**
Eye disease (OED):									
Visual impairment	5	1.1	<0.5	10	0.9	<0.5	9	0.7	<0.5
Blindness	1	0.3	9	3	0.3	19	2	0.2	16
**Eye disease (OED)**	**6**	**1.4**	**9**	**12**	**1.2**	**19**	**12**	**0.9**	**16**
**Total all manifestations**	**39**	**8.7**	**11**	**77**	**7.6**	**24**	**54**	**4.0**	**19**

Note 1: Population at risk, number of cases (N) and DALYs are presented in thousands.

Note 2: Total skin disease is defined as the sum of the number of cases with palpable nodules, reversible and irreversible skin disease.

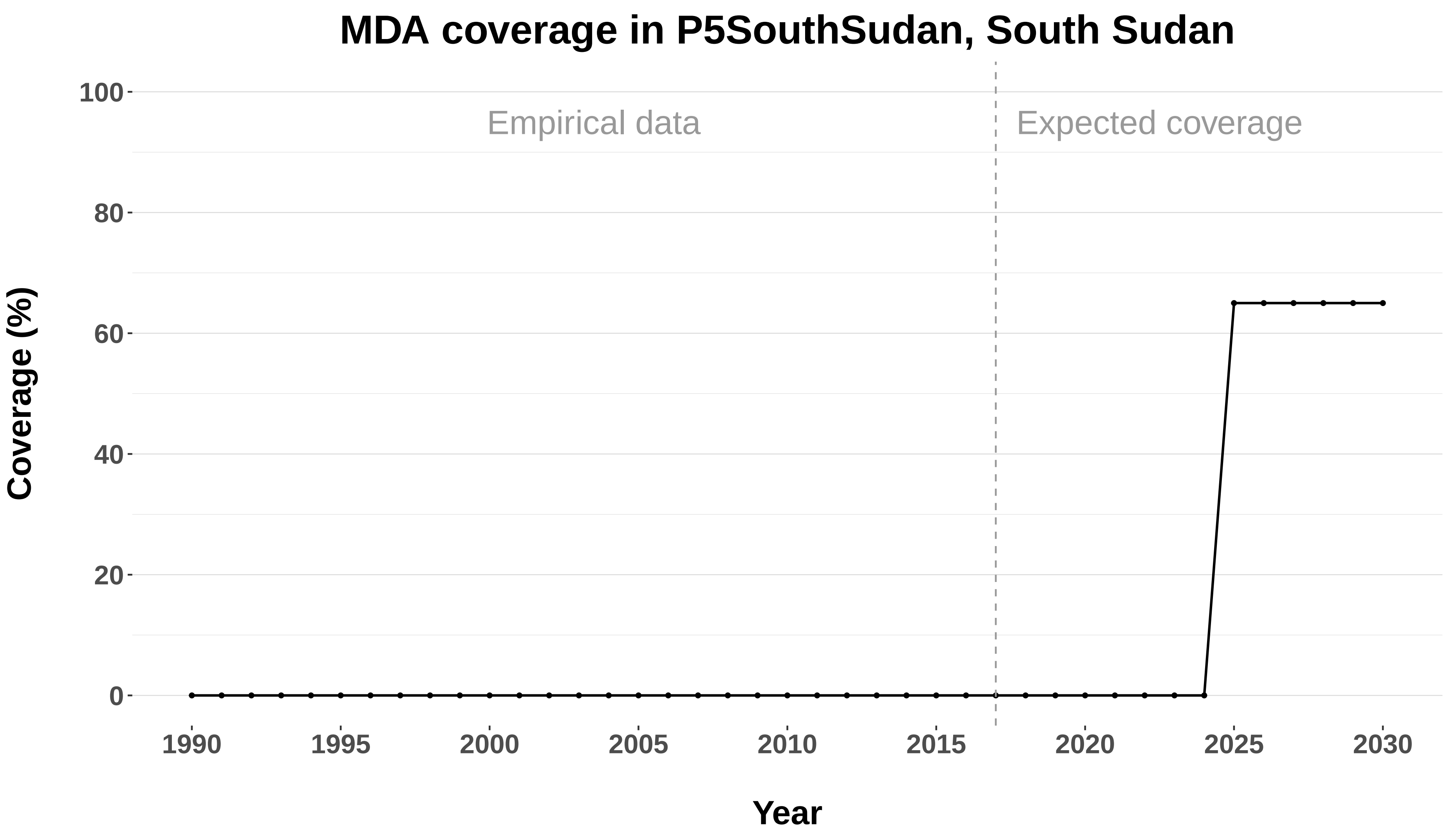
MDA coverage in P5SouthSudan, South Sudan
Empirical data
Expected coverage
Coverage (%)
100
80
60
40
20
0
1990
1995
2000
2005
2010
2015
2020
2025
2030
Year

**Project, country:**	**Upper Nile, South Sudan**
Bioclime:	
Endemicity level(s):	Mesoendemic
MDA start year:	2006
Mean MDA coverage per year:	39.5

	1990			2020			2030		
	N	%	DALYs	N	%	DALYs	N	%	DALYs
Total populaton at risk	304	100	_	686	100	_	899	100	_
**Infection:**									
Mf-positive	75	24.6	_	60	8.7	_	9	1.0	_
Mf-positive (age 5+)	75	24.5	_	60	8.7	_	9	1.0	_
Worm infected	100	32.9	_	119	17.3	_	53	5.9	_
**Clinical manifestations:**									
Palpable nodules	12	3.9	<0.5	7	1.0	<0.5	2	0.2	<0.5
Reversible skin disease:									
Severe itch	9	3.1	2	4	0.6	1	<0.5	0.1	<0.5
Reactive skin disease	6	2.1	<0.5	2	0.3	<0.5	<0.5	<0.05	<0.5
**Reversible skin disease**	**16**	**5.1**	**2**	**6**	**0.9**	**1**	**1**	**0.1**	**<0.5**
Irreversible skin disease:									
Mild depigmentation	2	0.8	<0.5	3	0.5	<0.5	2	0.3	<0.5
Severe depigmentation	2	0.7	<0.5	3	0.4	<0.5	2	0.2	<0.5
Atrophy	<0.5	<0.05	<0.5	<0.5	<0.05	<0.5	<0.5	<0.05	<0.5
Hanging groin	<0.5	<0.05	<0.5	<0.5	<0.05	<0.5	<0.5	<0.05	<0.5
**Irreversible skin disease**	**5**	**1.5**	**<0.5**	**6**	**0.8**	**<0.5**	**4**	**0.4**	**<0.5**
**Total skin disease (OSD)**	**32**	**10.5**	**2**	**19**	**2.7**	**1**	**6**	**0.7**	**<0.5**
Eye disease (OED):									
Visual impairment	4	1.4	<0.5	5	0.8	<0.5	4	0.4	<0.5
Blindness	1	0.4	9	1	0.2	8	1	0.1	4
**Eye disease (OED)**	**6**	**1.8**	**10**	**6**	**0.9**	**8**	**4**	**0.5**	**4**
**Total all manifestations**	**38**	**12.4**	**12**	**25**	**3.7**	**9**	**10**	**1.2**	**4**

Note 1: Population at risk, number of cases (N) and DALYs are presented in thousands.

Note 2: Total skin disease is defined as the sum of the number of cases with palpable nodules, reversible and irreversible skin disease.

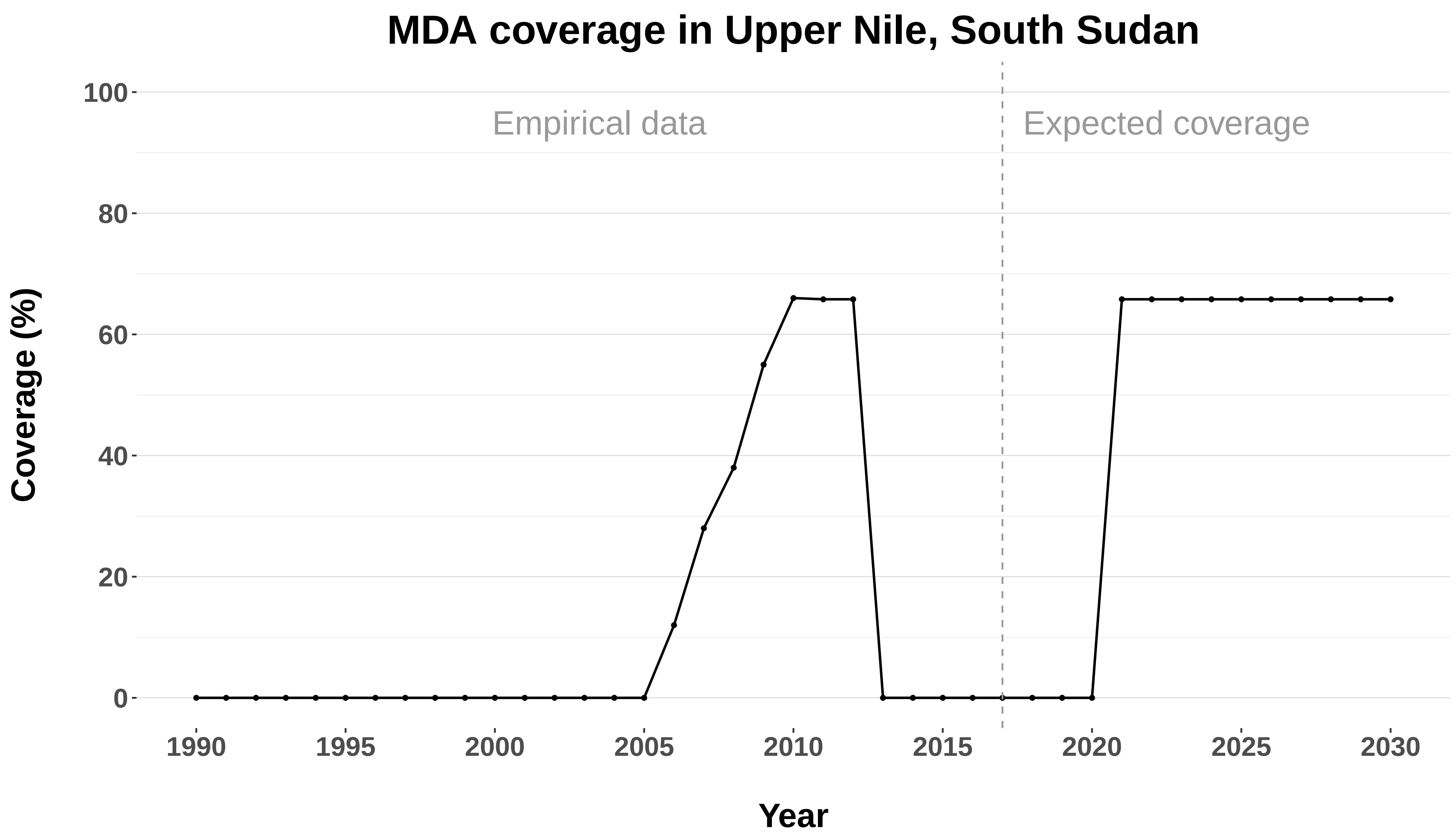
MDA coverage in Upper Nile, South Sudan
Empirical data
Expected coverage
Coverage (%)
Year
0
20
40
60
80
100
1990
1995
2000
2005
2010
2015
2020
2025
2030

**Project, country:**	**West Bahr El Ghazal, South Sudan**
Bioclime:	
Endemicity level(s):	Hyperendemic
MDA start year:	2006
Mean MDA coverage per year:	44.3

	1990			2020			2030		
	N	%	DALYs	N	%	DALYs	N	%	DALYs
Total populaton at risk	1,758	100	_	3,968	100	_	5,205	100	_
**Infection:**									
Mf-positive	525	29.8	_	509	12.8	_	137	2.6	_
Mf-positive (age 5+)	524	29.8	_	509	12.8	_	137	2.6	_
Worm infected	687	39.1	_	978	24.7	_	429	8.2	_
**Clinical manifestations:**									
Palpable nodules	137	7.8	1	141	3.5	1	53	1.0	1
Reversible skin disease:									
Severe itch	76	4.3	14	46	1.2	8	9	0.2	2
Reactive skin disease	56	3.2	3	17	0.4	1	2	<0.05	<0.5
**Reversible skin disease**	**132**	**7.5**	**16**	**63**	**1.6**	**9**	**11**	**0.2**	**2**
Irreversible skin disease:									
Mild depigmentation	18	1.0	<0.5	25	0.6	<0.5	20	0.4	<0.5
Severe depigmentation	18	1.0	1	24	0.6	2	18	0.3	1
Atrophy	<0.5	<0.05	<0.5	<0.5	<0.05	<0.5	<0.5	<0.05	<0.5
Hanging groin	<0.5	<0.05	<0.5	1	<0.05	<0.5	<0.5	<0.05	<0.5
**Irreversible skin disease**	**36**	**2.1**	**2**	**51**	**1.3**	**2**	**38**	**0.7**	**1**
**Total skin disease (OSD)**	**305**	**17.4**	**19**	**255**	**6.4**	**13**	**101**	**1.9**	**4**
Eye disease (OED):									
Visual impairment	32	1.8	1	46	1.2	1	35	0.7	1
Blindness	15	0.9	119	16	0.4	131	9	0.2	74
**Eye disease (OED)**	**47**	**2.7**	**120**	**63**	**1.6**	**133**	**44**	**0.8**	**75**
**Total all manifestations**	**353**	**20.1**	**139**	**317**	**8.0**	**145**	**145**	**2.8**	**79**

Note 1: Population at risk, number of cases (N) and DALYs are presented in thousands.

Note 2: Total skin disease is defined as the sum of the number of cases with palpable nodules, reversible and irreversible skin disease.

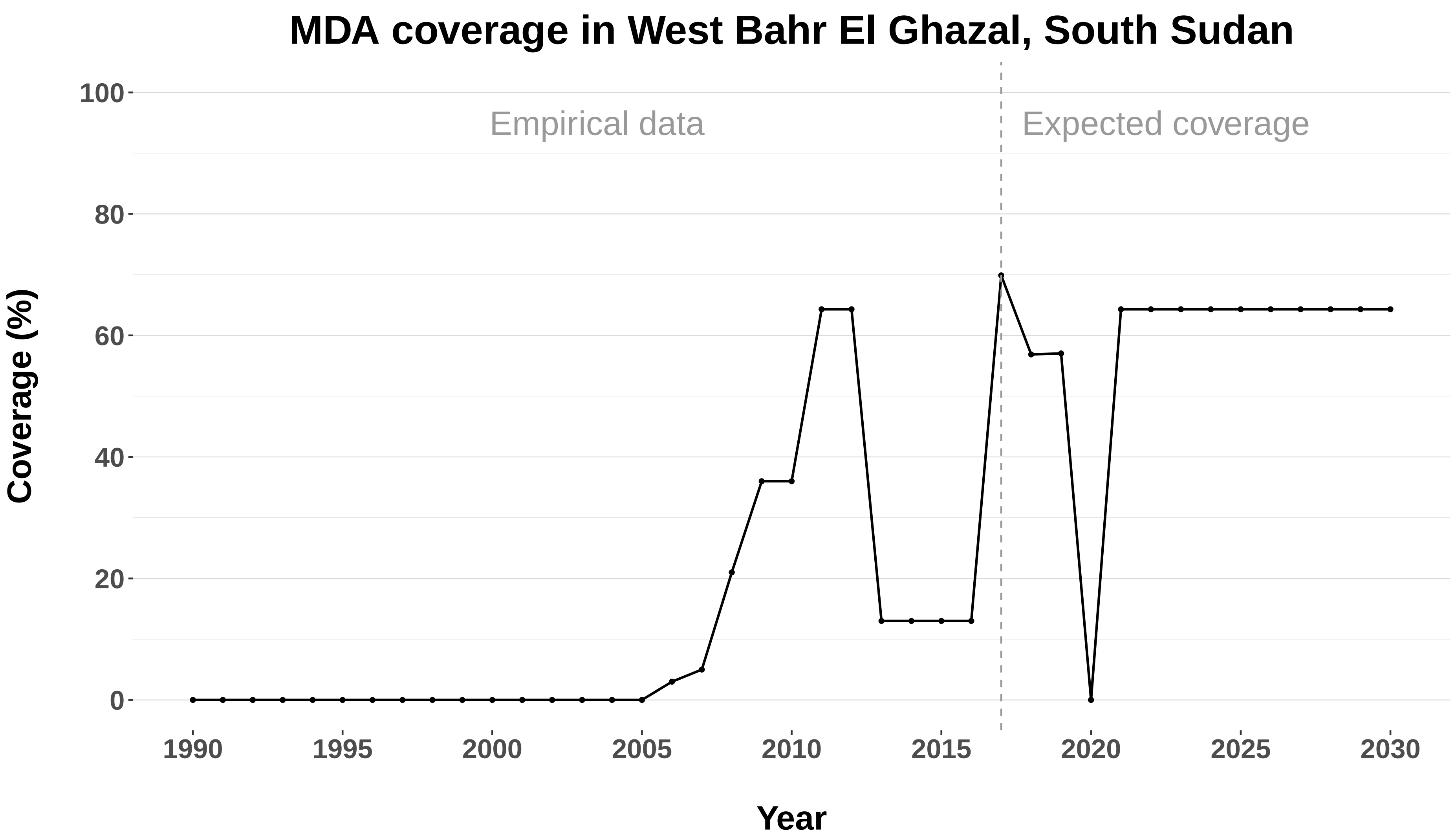
MDA coverage in West Bahr El Ghazal, South Sudan
Empirical data
Expected coverage
Coverage (%)
100
80
60
40
20
0
1990
1995
2000
2005
2010
2015
2020
2025
2030
Year

**Project, country:**	**West Equatoria, South Sudan**
Bioclime:	
Endemicity level(s):	Hyperendemic
MDA start year:	2005
Mean MDA coverage per year:	62.8

	1990			2020			2030		
	N	%	DALYs	N	%	DALYs	N	%	DALYs
Total populaton at risk	415	100	_	936	100	_	1,228	100	_
**Infection:**									
Mf-positive	141	33.9	_	26	2.7	_	1	<0.05	_
Mf-positive (age 5+)	140	33.9	_	26	2.7	_	1	<0.05	_
Worm infected	182	43.9	_	121	13.0	_	12	1.0	_
**Clinical manifestations:**									
Palpable nodules	38	9.2	<0.5	7	0.8	<0.5	<0.5	<0.05	<0.5
Reversible skin disease:									
Severe itch	19	4.6	3	2	0.2	<0.5	<0.5	<0.05	<0.5
Reactive skin disease	13	3.2	1	<0.5	<0.05	<0.5	<0.5	<0.05	<0.5
**Reversible skin disease**	**33**	**7.8**	**4**	**2**	**0.2**	**<0.5**	**<0.5**	**<0.05**	**<0.5**
Irreversible skin disease:									
Mild depigmentation	5	1.1	<0.5	5	0.6	<0.5	4	0.3	<0.5
Severe depigmentation	4	1.0	<0.5	4	0.5	<0.5	3	0.2	<0.5
Atrophy	<0.5	<0.05	<0.5	<0.5	<0.05	<0.5	<0.5	<0.05	<0.5
Hanging groin	<0.5	<0.05	<0.5	<0.5	<0.05	<0.5	<0.5	<0.05	<0.5
**Irreversible skin disease**	**9**	**2.2**	**<0.5**	**10**	**1.0**	**<0.5**	**6**	**0.5**	**<0.5**
**Total skin disease (OSD)**	**80**	**19.2**	**5**	**19**	**2.0**	**1**	**7**	**0.5**	**<0.5**
Eye disease (OED):									
Visual impairment	8	2.0	<0.5	9	1.0	<0.5	6	0.5	<0.5
Blindness	3	0.7	22	2	0.2	14	1	0.1	7
**Eye disease (OED)**	**11**	**2.7**	**22**	**11**	**1.2**	**14**	**7**	**0.6**	**7**
**Total all manifestations**	**91**	**21.9**	**27**	**30**	**3.2**	**15**	**14**	**1.1**	**7**

Note 1: Population at risk, number of cases (N) and DALYs are presented in thousands.

Note 2: Total skin disease is defined as the sum of the number of cases with palpable nodules, reversible and irreversible skin disease.

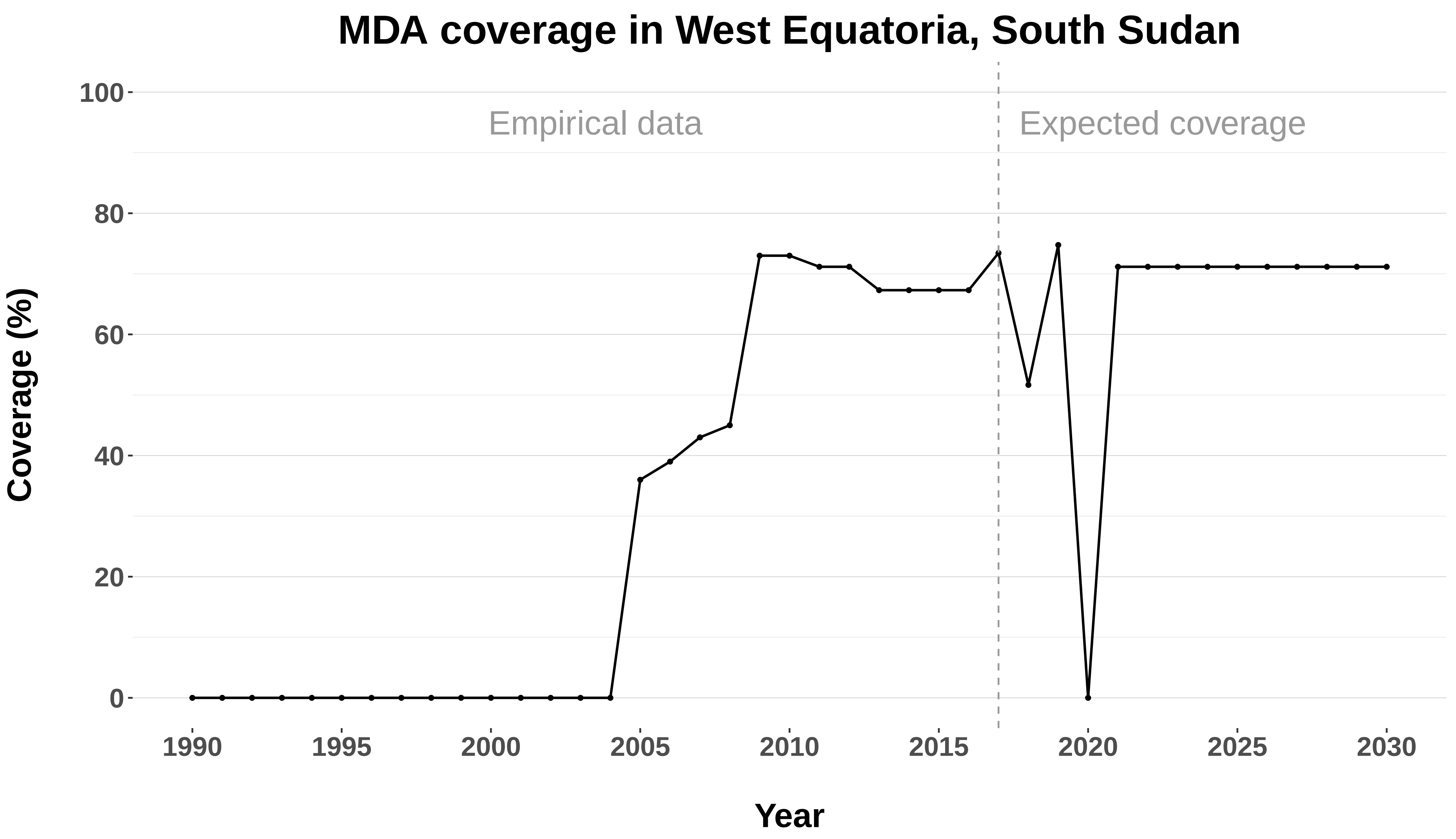
MDA coverage in West Equatoria, South Sudan
Empirical data
Expected coverage
Coverage (%)
100
80
60
40
20
0
1990
1995
2000
2005
2010
2015
2020
2025
2030
Year

**Project, country:**	**P5Sudan, Sudan**
Bioclime:	
Endemicity level(s):	Hypoendemic
MDA start year:	2025
Mean MDA coverage per year:	65.0

	1990			2020			2030		
	N	%	DALYs	N	%	DALYs	N	%	DALYs
Total populaton at risk	127	100	_	286	100	_	375	100	_
**Infection:**									
Mf-positive	29	22.6	_	61	21.2	_	48	12.9	_
Mf-positive (age 5+)	29	22.6	_	61	21.2	_	48	12.9	_
Worm infected	38	30.3	_	82	28.8	_	88	23.4	_
**Clinical manifestations:**									
Palpable nodules	4	2.9	<0.5	8	2.7	<0.5	7	1.8	<0.5
Reversible skin disease:									
Severe itch	4	2.8	1	8	2.7	1	4	1.1	1
Reactive skin disease	2	1.9	<0.5	5	1.7	<0.5	1	0.3	<0.5
**Reversible skin disease**	**6**	**4.7**	**1**	**13**	**4.4**	**2**	**5**	**1.4**	**1**
Irreversible skin disease:									
Mild depigmentation	1	0.8	<0.5	2	0.7	<0.5	2	0.5	<0.5
Severe depigmentation	1	0.6	<0.5	2	0.6	<0.5	2	0.4	<0.5
Atrophy	<0.5	<0.05	<0.5	<0.5	<0.05	<0.5	<0.5	<0.05	<0.5
Hanging groin	<0.5	<0.05	<0.5	<0.5	<0.05	<0.5	<0.5	<0.05	<0.5
**Irreversible skin disease**	**2**	**1.4**	**<0.5**	**4**	**1.3**	**<0.5**	**4**	**1.0**	**<0.5**
**Total skin disease (OSD)**	**11**	**8.9**	**1**	**24**	**8.4**	**2**	**15**	**4.1**	**1**
Eye disease (OED):									
Visual impairment	2	1.3	<0.5	3	1.2	<0.5	4	0.9	<0.5
Blindness	<0.5	0.4	3	1	0.4	7	1	0.2	6
**Eye disease (OED)**	**2**	**1.7**	**3**	**5**	**1.6**	**8**	**4**	**1.2**	**6**
**Total all manifestations**	**13**	**10.6**	**4**	**28**	**10.0**	**9**	**20**	**5.3**	**7**

Note 1: Population at risk, number of cases (N) and DALYs are presented in thousands.

Note 2: Total skin disease is defined as the sum of the number of cases with palpable nodules, reversible and irreversible skin disease.

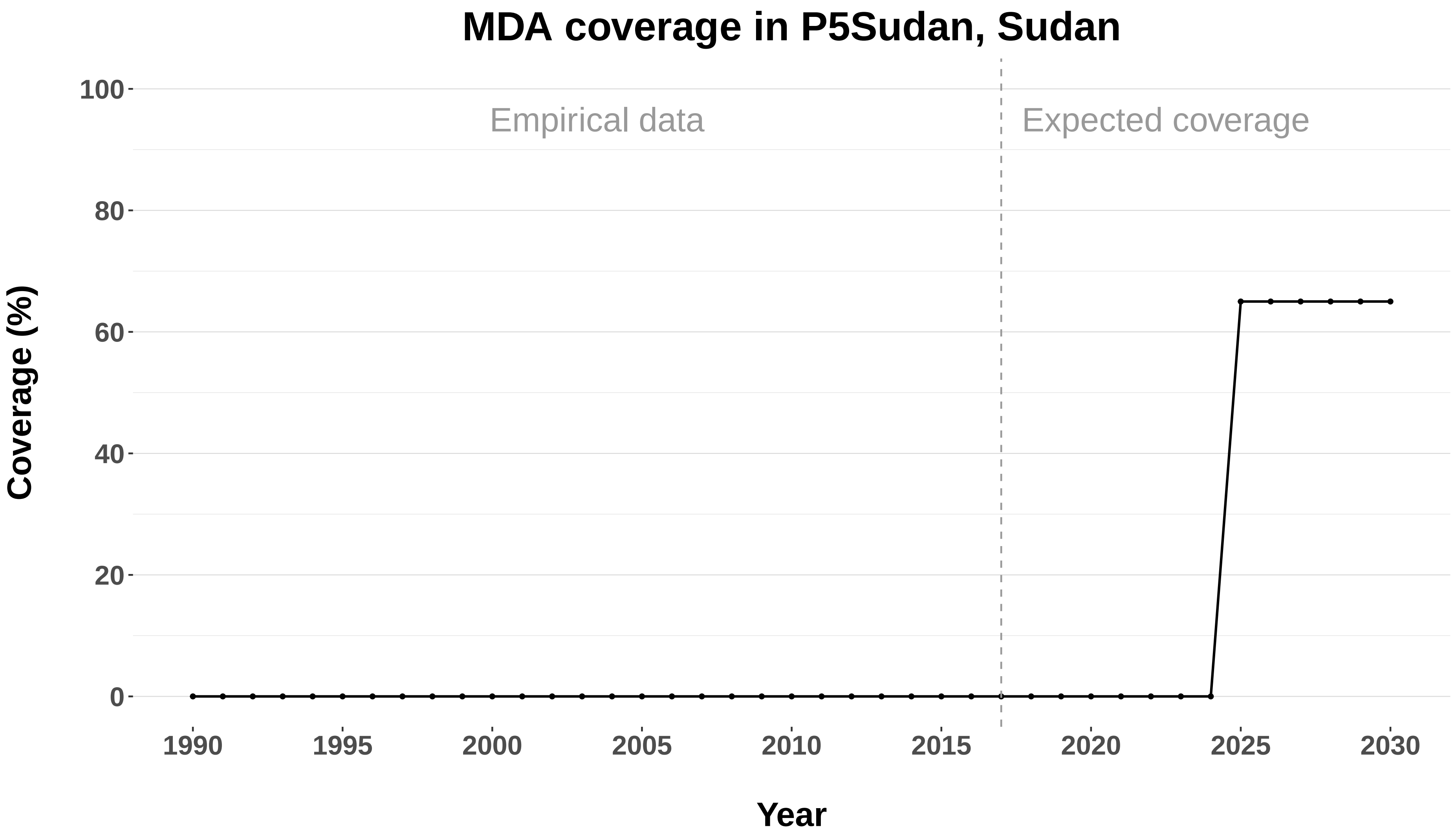
MDA coverage in P5Sudan, Sudan
Empirical data
Expected coverage
Coverage (%)
100
80
60
40
20
0
1990
1995
2000
2005
2010
2015
2020
2025
2030
Year

**Project, country:**	**Sudan, Sudan**
Bioclime:	
Endemicity level(s):	Hypoendemic
MDA start year:	2008
Mean MDA coverage per year:	78.0

	1990			2020			2030		
	N	%	DALYs	N	%	DALYs	N	%	DALYs
Total populaton at risk	120	100	_	270	100	_	354	100	_
**Infection:**									
Mf-positive	25	21.1	_	2	0.7	_	<0.5	<0.05	_
Mf-positive (age 5+)	25	21.1	_	2	0.7	_	<0.5	<0.05	_
Worm infected	34	28.6	_	17	6.4	_	1	0.2	_
**Clinical manifestations:**									
Palpable nodules	3	2.2	<0.5	<0.5	0.1	<0.5	<0.5	<0.05	<0.5
Reversible skin disease:									
Severe itch	3	2.5	1	<0.5	0.1	<0.5	<0.5	<0.05	<0.5
Reactive skin disease	2	1.6	<0.5	<0.5	<0.05	<0.5	<0.5	<0.05	<0.5
**Reversible skin disease**	**5**	**4.2**	**1**	**<0.5**	**0.1**	**<0.5**	**<0.5**	**<0.05**	**<0.5**
Irreversible skin disease:									
Mild depigmentation	1	0.7	<0.5	1	0.4	<0.5	1	0.2	<0.5
Severe depigmentation	1	0.6	<0.5	1	0.3	<0.5	<0.5	0.1	<0.5
Atrophy	<0.5	<0.05	<0.5	<0.5	<0.05	<0.5	<0.5	<0.05	<0.5
Hanging groin	<0.5	<0.05	<0.5	<0.5	<0.05	<0.5	<0.5	<0.05	<0.5
**Irreversible skin disease**	**1**	**1.2**	**<0.5**	**2**	**0.6**	**<0.5**	**1**	**0.3**	**<0.5**
**Total skin disease (OSD)**	**9**	**7.6**	**1**	**2**	**0.8**	**<0.5**	**1**	**0.3**	**<0.5**
Eye disease (OED):									
Visual impairment	1	1.2	<0.5	1	0.5	<0.5	1	0.3	<0.5
Blindness	<0.5	0.3	3	<0.5	0.1	2	<0.5	<0.05	1
**Eye disease (OED)**	**2**	**1.5**	**3**	**2**	**0.6**	**2**	**1**	**0.3**	**1**
**Total all manifestations**	**11**	**9.1**	**4**	**4**	**1.4**	**2**	**2**	**0.6**	**1**

Note 1: Population at risk, number of cases (N) and DALYs are presented in thousands.

Note 2: Total skin disease is defined as the sum of the number of cases with palpable nodules, reversible and irreversible skin disease.

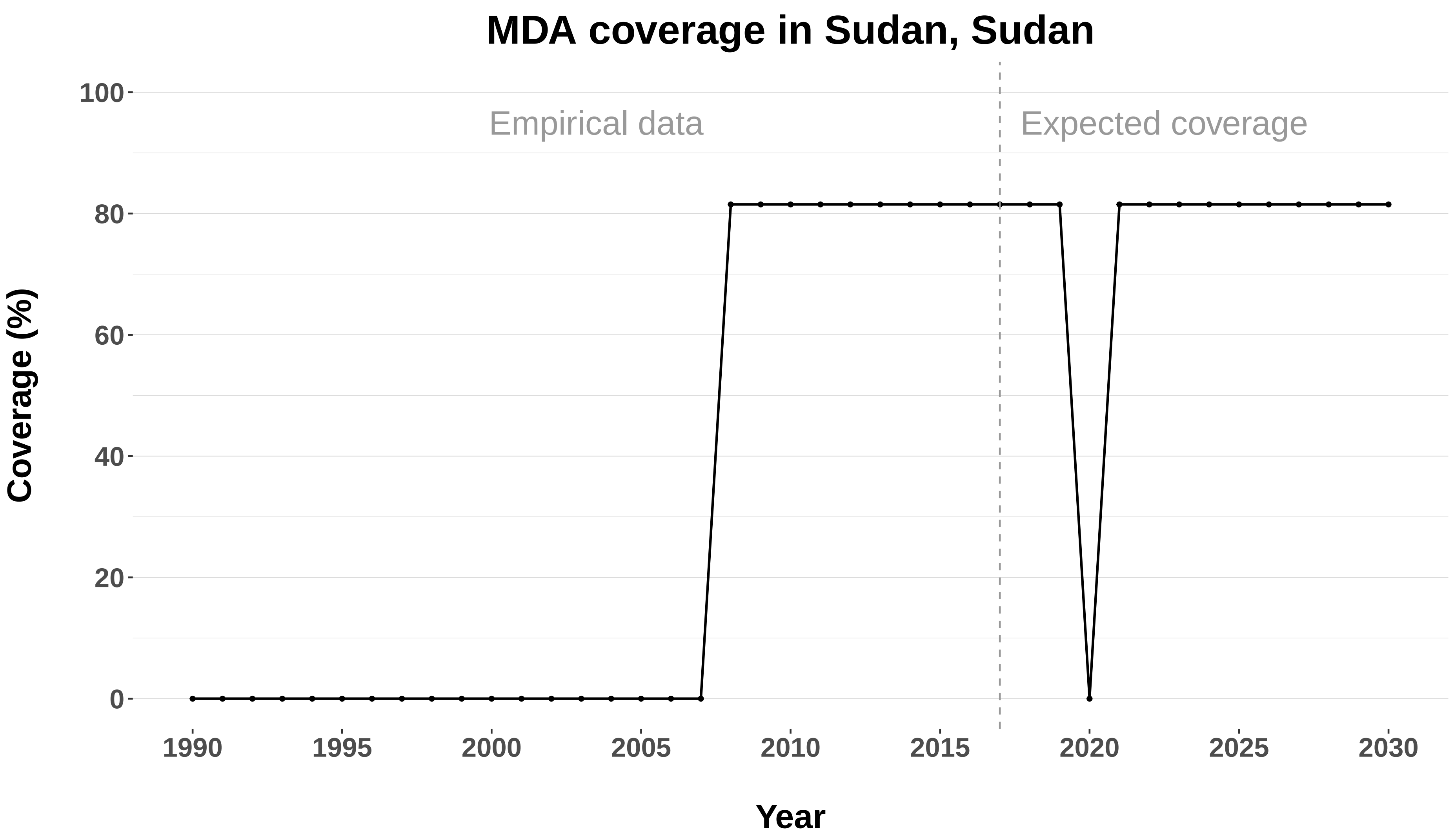

MDA coverage in Sudan, Sudan
Empirical data
Expected coverage
Coverage (%)
100
80
60
40
20
0
1990
1995
2000
2005
2010
2015
2020
2025
2030
Year

**Project, country:**	**Sudan Abu Hamed, Sudan**
Bioclime:	
Endemicity level(s):	Hypoendemic
MDA start year:	2008
Mean MDA coverage per year:	78.0

	1990			2020			2030		
	N	%	DALYs	N	%	DALYs	N	%	DALYs
Total populaton at risk	120	100	_	270	100	_	354	100	_
**Infection:**									
Mf-positive	25	21.1	_	2	0.7	_	<0.5	<0.05	_
Mf-positive (age 5+)	25	21.1	_	2	0.7	_	<0.5	<0.05	_
Worm infected	34	28.6	_	17	6.4	_	1	0.2	_
**Clinical manifestations:**									
Palpable nodules	3	2.2	<0.5	<0.5	0.1	<0.5	<0.5	<0.05	<0.5
Reversible skin disease:									
Severe itch	3	2.5	1	<0.5	0.1	<0.5	<0.5	<0.05	<0.5
Reactive skin disease	2	1.6	<0.5	<0.5	<0.05	<0.5	<0.5	<0.05	<0.5
**Reversible skin disease**	**5**	**4.2**	**1**	**<0.5**	**0.1**	**<0.5**	**<0.5**	**<0.05**	**<0.5**
Irreversible skin disease:									
Mild depigmentation	1	0.7	<0.5	1	0.4	<0.5	1	0.2	<0.5
Severe depigmentation	1	0.6	<0.5	1	0.3	<0.5	<0.5	0.1	<0.5
Atrophy	<0.5	<0.05	<0.5	<0.5	<0.05	<0.5	<0.5	<0.05	<0.5
Hanging groin	<0.5	<0.05	<0.5	<0.5	<0.05	<0.5	<0.5	<0.05	<0.5
**Irreversible skin disease**	**1**	**1.2**	**<0.5**	**2**	**0.6**	**<0.5**	**1**	**0.3**	**<0.5**
**Total skin disease (OSD)**	**9**	**7.6**	**1**	**2**	**0.8**	**<0.5**	**1**	**0.3**	**<0.5**
Eye disease (OED):									
Visual impairment	1	1.2	<0.5	1	0.5	<0.5	1	0.3	<0.5
Blindness	<0.5	0.3	3	<0.5	0.1	2	<0.5	<0.05	1
**Eye disease (OED)**	**2**	**1.5**	**3**	**2**	**0.6**	**2**	**1**	**0.3**	**1**
**Total all manifestations**	**11**	**9.1**	**4**	**4**	**1.4**	**2**	**2**	**0.6**	**1**

Note 1: Population at risk, number of cases (N) and DALYs are presented in thousands.

Note 2: Total skin disease is defined as the sum of the number of cases with palpable nodules, reversible and irreversible skin disease.

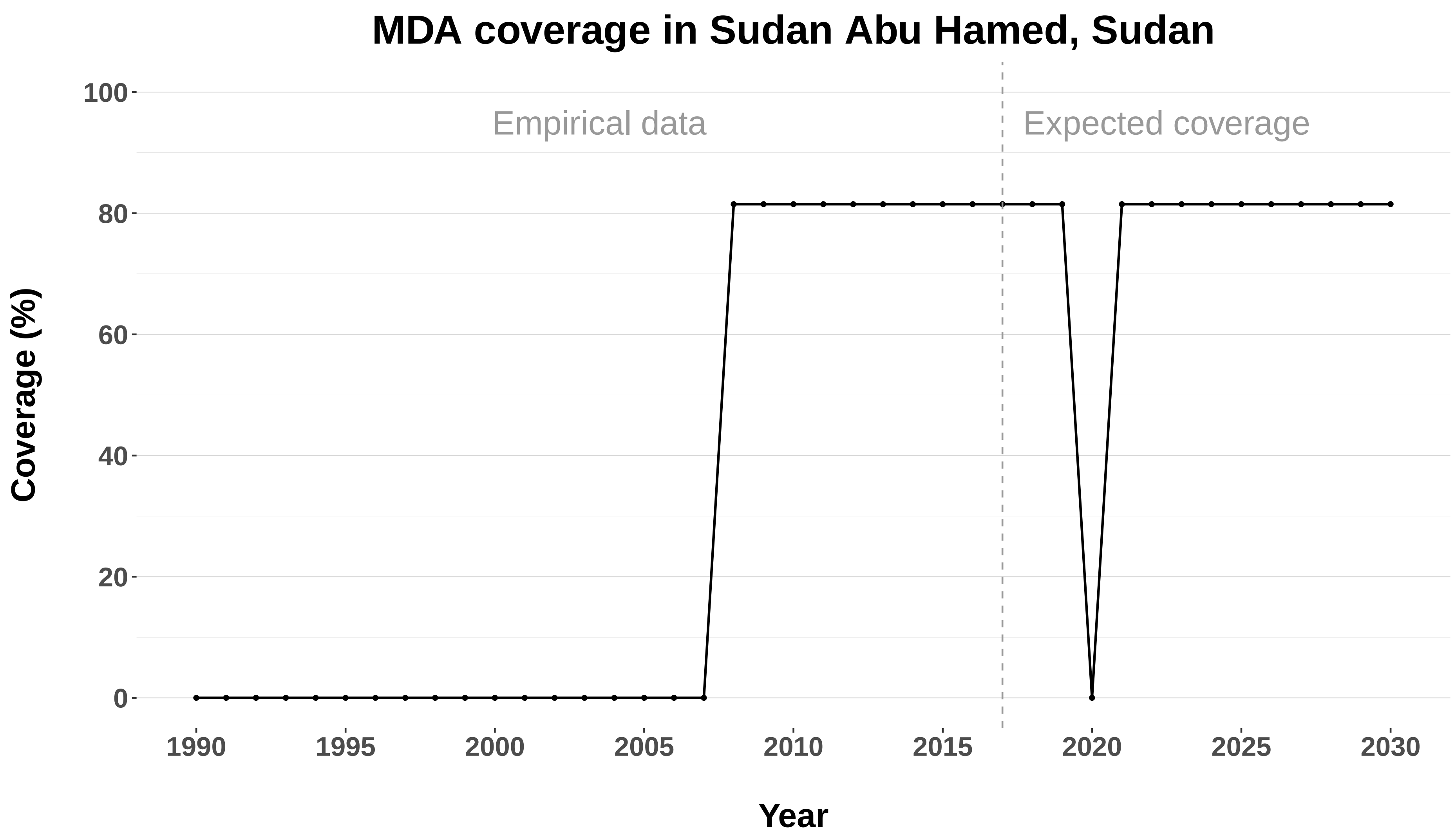

MDA coverage in Sudan Abu Hamed, Sudan
Empirical data
Expected coverage
Coverage (%)
100
80
60
40
20
0
1990
1995
2000
2005
2010
2015
2020
2025
2030
Year

**Project, country:**	**Kilosa, Tanzania**
Bioclime:	
Endemicity level(s):	Mesoendemic
MDA start year:	2002
Mean MDA coverage per year:	74.6

	1990			2020			2030		
	N	%	DALYs	N	%	DALYs	N	%	DALYs
Total populaton at risk	287	100	_	647	100	_	849	100	_
**Infection:**									
Mf-positive	80	27.8	_	1	0.1	_	<0.5	<0.05	_
Mf-positive (age 5+)	80	27.8	_	1	0.1	_	<0.5	<0.05	_
Worm infected	102	35.6	_	16	2.5	_	<0.5	<0.05	_
**Clinical manifestations:**									
Palpable nodules	14	4.8	<0.5	<0.5	<0.05	<0.5	<0.5	<0.05	<0.5
Reversible skin disease:									
Severe itch	11	3.8	2	<0.5	<0.05	<0.5	<0.5	<0.05	<0.5
Reactive skin disease	8	2.7	<0.5	<0.5	<0.05	<0.5	<0.5	<0.05	<0.5
**Reversible skin disease**	**19**	**6.5**	**2**	**<0.5**	**<0.05**	**<0.5**	**<0.5**	**<0.05**	**<0.5**
Irreversible skin disease:									
Mild depigmentation	3	1.0	<0.5	3	0.4	<0.5	2	0.2	<0.5
Severe depigmentation	3	0.9	<0.5	2	0.3	<0.5	1	0.1	<0.5
Atrophy	<0.5	<0.05	<0.5	<0.5	<0.05	<0.5	<0.5	<0.05	<0.5
Hanging groin	<0.5	<0.05	<0.5	<0.5	<0.05	<0.5	<0.5	<0.05	<0.5
**Irreversible skin disease**	**5**	**1.9**	**<0.5**	**5**	**0.7**	**<0.5**	**3**	**0.3**	**<0.5**
**Total skin disease (OSD)**	**38**	**13.2**	**3**	**5**	**0.8**	**<0.5**	**3**	**0.3**	**<0.5**
Eye disease (OED):									
Visual impairment	<0.5	<0.05	<0.5	<0.5	<0.05	<0.5	<0.5	<0.05	<0.5
Blindness	<0.5	<0.05	<0.5	<0.5	<0.05	<0.5	<0.5	<0.05	<0.5
**Eye disease (OED)**	**<0.5**	**<0.05**	**<0.5**	**<0.5**	**<0.05**	**<0.5**	**<0.5**	**<0.05**	**<0.5**
**Total all manifestations**	**38**	**13.2**	**3**	**5**	**0.8**	**<0.5**	**3**	**0.3**	**<0.5**

Note 1: Population at risk, number of cases (N) and DALYs are presented in thousands.

Note 2: Total skin disease is defined as the sum of the number of cases with palpable nodules, reversible and irreversible skin disease.

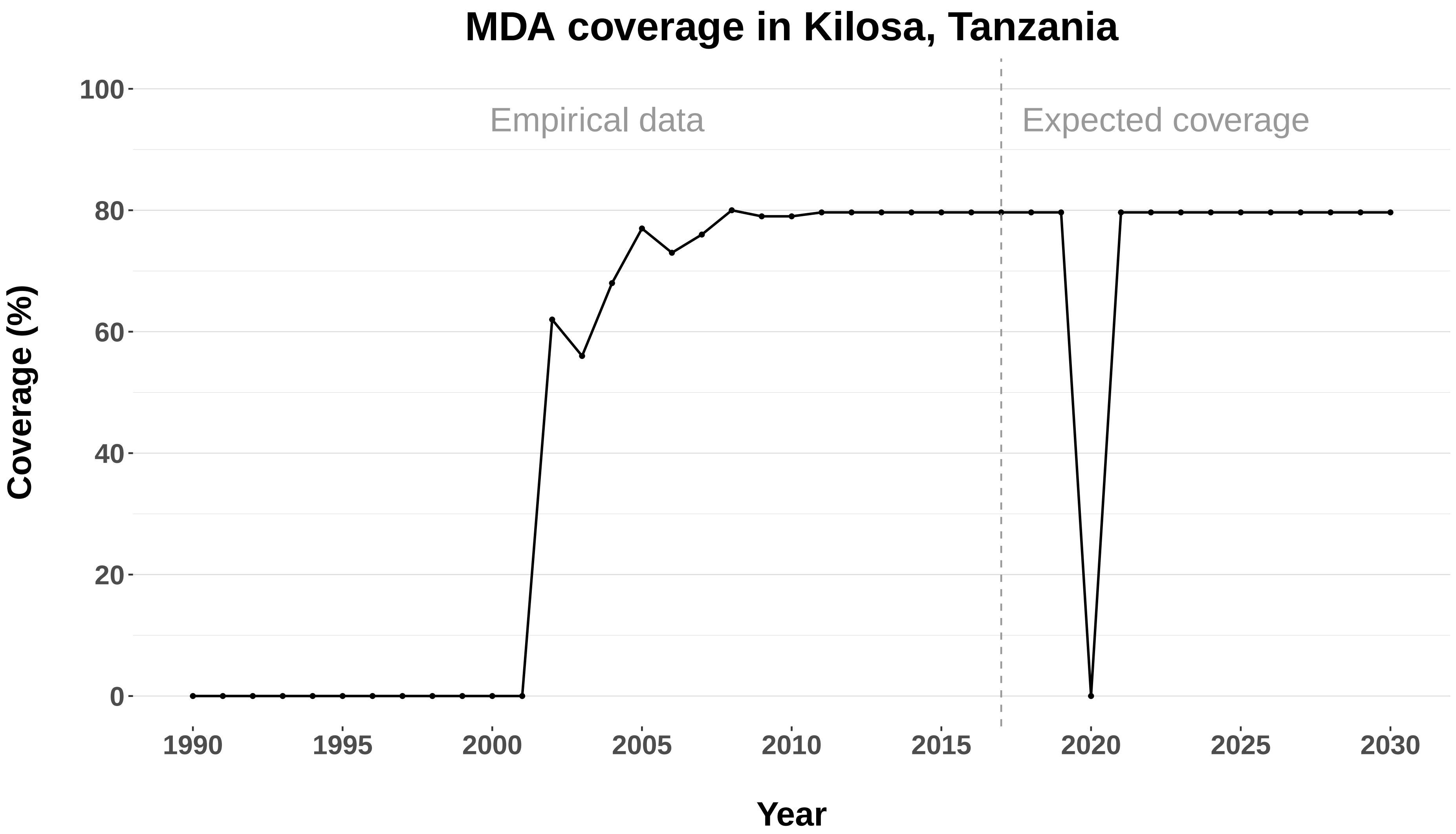
MDA coverage in Kilosa, Tanzania
Empirical data
Expected coverage
Coverage (%)
100
80
60
40
20
0
1990
1995
2000
2005
2010
2015
2020
2025
2030
Year

**Project, country:**	**Mahenge, Tanzania**
Bioclime:	
Endemicity level(s):	Hyperendemic
MDA start year:	1998
Mean MDA coverage per year:	68.4

	1990			2020			2030		
	N	%	DALYs	N	%	DALYs	N	%	DALYs
Total populaton at risk	287	100	_	647	100	_	849	100	_
**Infection:**									
Mf-positive	127	44.2	_	8	1.3	_	1	0.1	_
Mf-positive (age 5+)	126	44.1	_	8	1.3	_	1	0.1	_
Worm infected	154	53.8	_	42	6.5	_	6	0.7	_
**Clinical manifestations:**									
Palpable nodules	48	16.8	1	3	0.5	<0.5	<0.5	<0.05	<0.5
Reversible skin disease:									
Severe itch	22	7.7	4	<0.5	0.1	<0.5	<0.5	<0.05	<0.5
Reactive skin disease	17	6.0	1	<0.5	<0.05	<0.5	<0.5	<0.05	<0.5
**Reversible skin disease**	**39**	**13.7**	**5**	**1**	**0.1**	**<0.5**	**<0.5**	**<0.05**	**<0.5**
Irreversible skin disease:									
Mild depigmentation	5	1.6	<0.5	4	0.6	<0.5	3	0.3	<0.5
Severe depigmentation	6	2.1	<0.5	4	0.7	<0.5	3	0.3	<0.5
Atrophy	<0.5	<0.05	<0.5	<0.5	<0.05	<0.5	<0.5	<0.05	<0.5
Hanging groin	<0.5	0.1	<0.5	<0.5	<0.05	<0.5	<0.5	<0.05	<0.5
**Irreversible skin disease**	**11**	**3.8**	**1**	**8**	**1.3**	**<0.5**	**5**	**0.6**	**<0.5**
**Total skin disease (OSD)**	**99**	**34.4**	**6**	**12**	**1.9**	**<0.5**	**6**	**0.7**	**<0.5**
Eye disease (OED):									
Visual impairment	<0.5	0.1	<0.5	<0.5	<0.05	<0.5	<0.5	<0.05	<0.5
Blindness	<0.5	0.2	3	<0.5	<0.05	1	<0.5	<0.05	<0.5
**Eye disease (OED)**	**1**	**0.3**	**3**	**<0.5**	**0.1**	**1**	**<0.5**	**<0.05**	**<0.5**
**Total all manifestations**	**99**	**34.6**	**9**	**13**	**1.9**	**1**	**6**	**0.7**	**1**

Note 1: Population at risk, number of cases (N) and DALYs are presented in thousands.

Note 2: Total skin disease is defined as the sum of the number of cases with palpable nodules, reversible and irreversible skin disease.

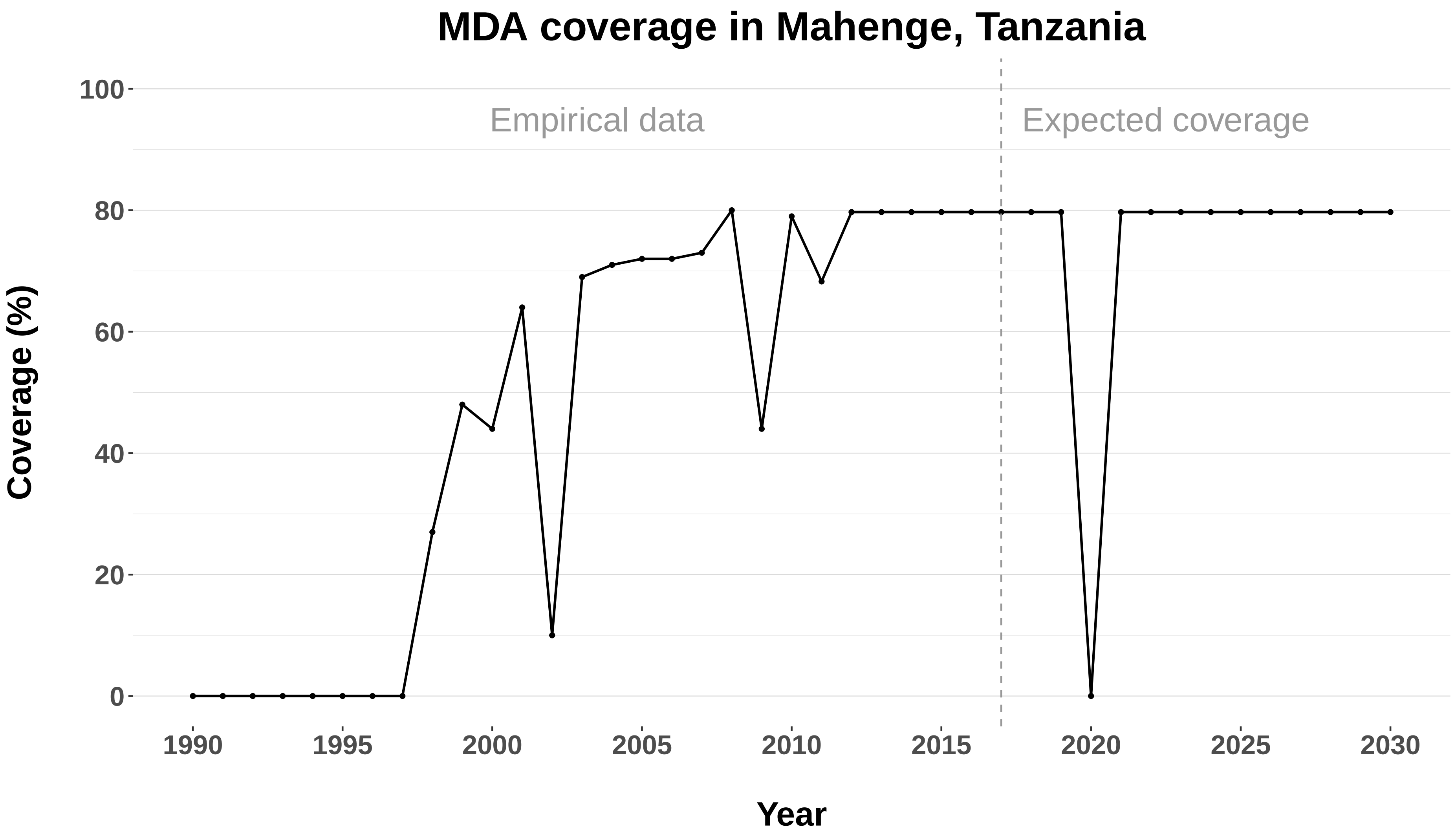
MDA coverage in Mahenge, Tanzania
Empirical data
Expected coverage
Coverage (%)
100
80
60
40
20
0
1990
1995
2000
2005
2010
2015
2020
2025
2030
Year

**Project, country:**	**Morogoro, Tanzania**
Bioclime:	
Endemicity level(s):	Mesoendemic
MDA start year:	2004
Mean MDA coverage per year:	73.1

	1990			2020			2030		
	N	%	DALYs	N	%	DALYs	N	%	DALYs
Total populaton at risk	205	100	_	463	100	_	608	100	_
**Infection:**									
Mf-positive	58	28.1	_	2	0.3	_	<0.5	<0.05	_
Mf-positive (age 5+)	58	28.1	_	2	0.3	_	<0.5	<0.05	_
Worm infected	75	36.8	_	22	4.7	_	1	0.1	_
**Clinical manifestations:**									
Palpable nodules	11	5.6	<0.5	1	0.1	<0.5	<0.5	<0.05	<0.5
Reversible skin disease:									
Severe itch	8	3.7	1	<0.5	<0.05	<0.5	<0.5	<0.05	<0.5
Reactive skin disease	5	2.5	<0.5	<0.5	<0.05	<0.5	<0.5	<0.05	<0.5
**Reversible skin disease**	**13**	**6.2**	**2**	**<0.5**	**<0.05**	**<0.5**	**<0.5**	**<0.05**	**<0.5**
Irreversible skin disease:									
Mild depigmentation	2	0.9	<0.5	2	0.4	<0.5	1	0.2	<0.5
Severe depigmentation	2	0.8	<0.5	1	0.3	<0.5	1	0.2	<0.5
Atrophy	<0.5	<0.05	<0.5	<0.5	<0.05	<0.5	<0.5	<0.05	<0.5
Hanging groin	<0.5	<0.05	<0.5	<0.5	<0.05	<0.5	<0.5	<0.05	<0.5
**Irreversible skin disease**	**4**	**1.8**	**<0.5**	**3**	**0.7**	**<0.5**	**2**	**0.4**	**<0.5**
**Total skin disease (OSD)**	**28**	**13.5**	**2**	**4**	**0.9**	**<0.5**	**2**	**0.4**	**<0.5**
Eye disease (OED):									
Visual impairment	<0.5	<0.05	<0.5	<0.5	<0.05	<0.5	<0.5	<0.05	<0.5
Blindness	<0.5	<0.05	<0.5	<0.5	<0.05	<0.5	<0.5	<0.05	<0.5
**Eye disease (OED)**	**<0.5**	**<0.05**	**<0.5**	**<0.5**	**<0.05**	**<0.5**	**<0.5**	**<0.05**	**<0.5**
**Total all manifestations**	**28**	**13.5**	**2**	**4**	**0.9**	**<0.5**	**2**	**0.4**	**<0.5**

Note 1: Population at risk, number of cases (N) and DALYs are presented in thousands.

Note 2: Total skin disease is defined as the sum of the number of cases with palpable nodules, reversible and irreversible skin disease.

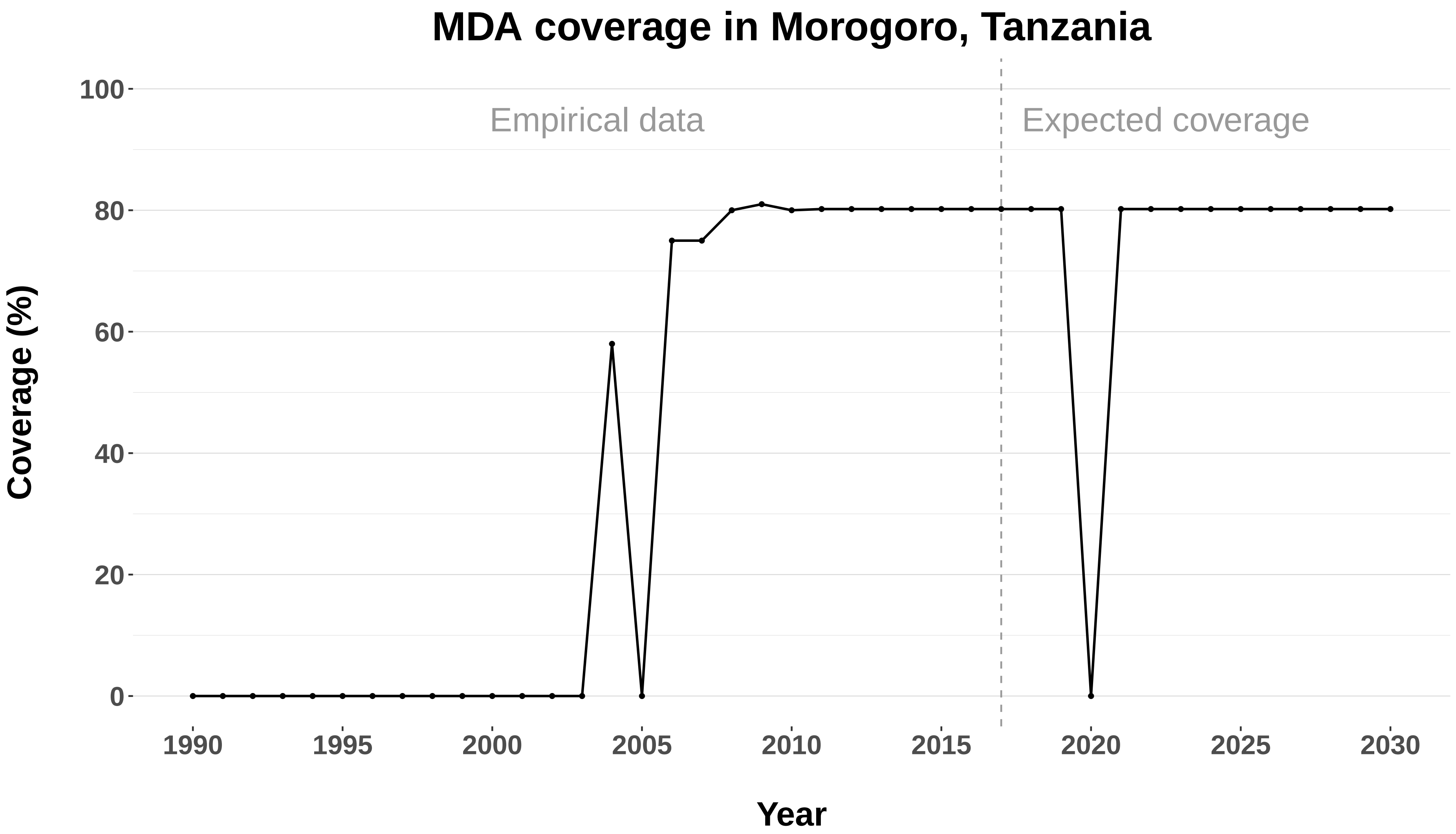
MDA coverage in Morogoro, Tanzania
Empirical data
Expected coverage
Coverage (%)
100
80
60
40
20
0
1990
1995
2000
2005
2010
2015
2020
2025
2030
Year

**Project, country:**	**P5Tanzania, Tanzania**
Bioclime:	
Endemicity level(s):	Hypoendemic
MDA start year:	2023
Mean MDA coverage per year:	65.0

	1990			2020			2030		
	N	%	DALYs	N	%	DALYs	N	%	DALYs
Total populaton at risk	520	100	_	1,174	100	_	1,540	100	_
**Infection:**									
Mf-positive	135	25.9	_	296	25.2	_	182	11.8	_
Mf-positive (age 5+)	135	25.9	_	296	25.2	_	182	11.8	_
Worm infected	176	33.7	_	389	33.1	_	371	24.1	_
**Clinical manifestations:**									
Palpable nodules	23	4.4	<0.5	50	4.3	1	33	2.2	<0.5
Reversible skin disease:									
Severe itch	18	3.4	3	39	3.3	7	15	1.0	3
Reactive skin disease	12	2.3	1	27	2.3	1	3	0.2	<0.5
**Reversible skin disease**	**30**	**5.7**	**4**	**66**	**5.6**	**8**	**19**	**1.2**	**3**
Irreversible skin disease:									
Mild depigmentation	5	0.9	<0.5	10	0.9	<0.5	10	0.7	<0.5
Severe depigmentation	4	0.8	<0.5	9	0.8	1	9	0.6	1
Atrophy	<0.5	<0.05	<0.5	<0.5	<0.05	<0.5	<0.5	<0.05	<0.5
Hanging groin	<0.5	<0.05	<0.5	<0.5	<0.05	<0.5	<0.5	<0.05	<0.5
**Irreversible skin disease**	**9**	**1.7**	**<0.5**	**19**	**1.7**	**1**	**19**	**1.2**	**1**
**Total skin disease (OSD)**	**61**	**11.7**	**4**	**136**	**11.6**	**10**	**71**	**4.6**	**4**
Eye disease (OED):									
Visual impairment	<0.5	<0.05	<0.5	<0.5	<0.05	<0.5	<0.5	<0.05	<0.5
Blindness	<0.5	<0.05	<0.5	<0.5	<0.05	1	<0.5	<0.05	<0.5
**Eye disease (OED)**	**<0.5**	**<0.05**	**<0.5**	**<0.5**	**<0.05**	**1**	**<0.5**	**<0.05**	**<0.5**
**Total all manifestations**	**61**	**11.8**	**5**	**136**	**11.6**	**10**	**71**	**4.6**	**4**

Note 1: Population at risk, number of cases (N) and DALYs are presented in thousands.

Note 2: Total skin disease is defined as the sum of the number of cases with palpable nodules, reversible and irreversible skin disease.

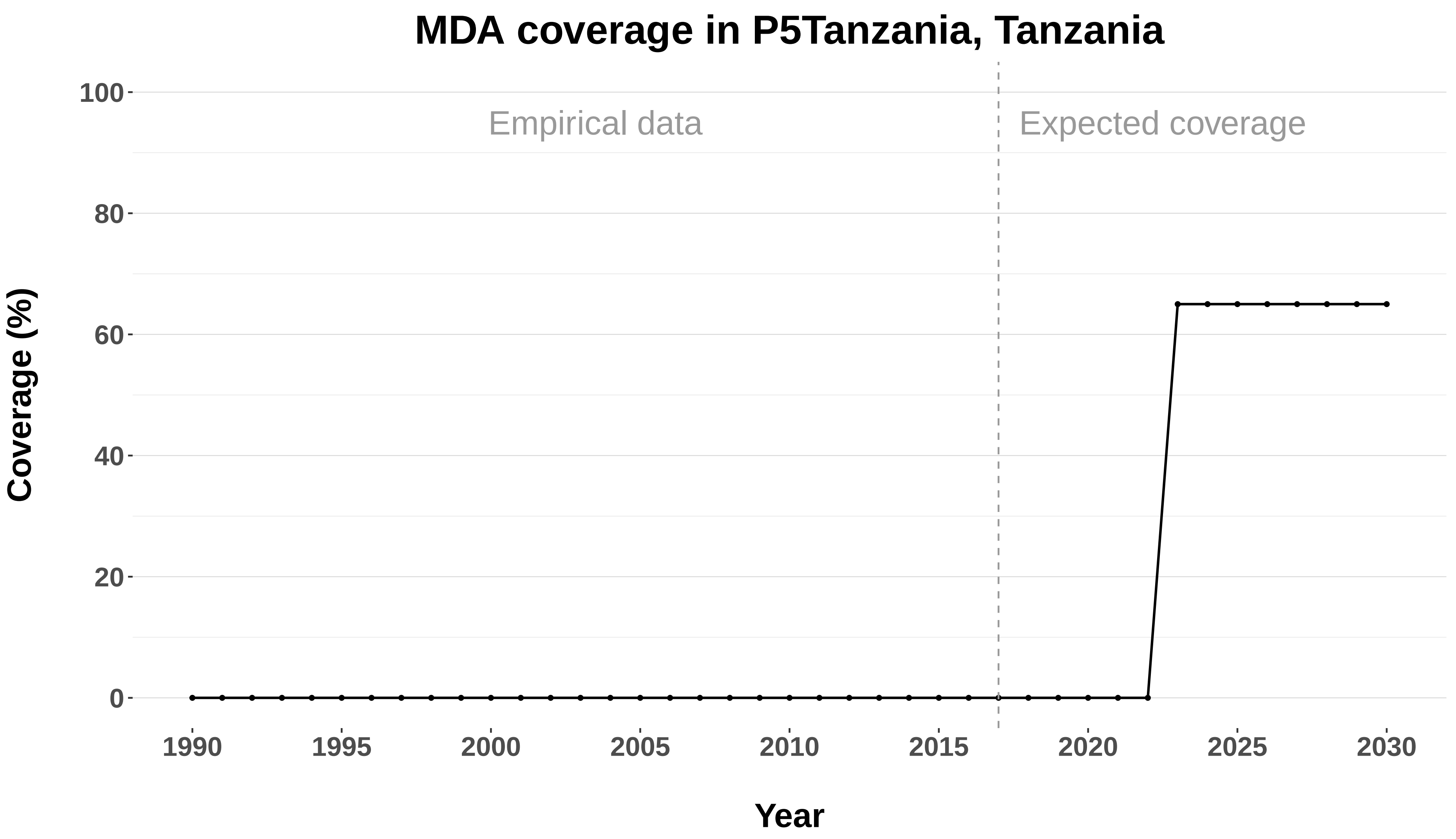
MDA coverage in P5Tanzania, Tanzania
Empirical data
Expected coverage
Coverage (%)
100
80
60
40
20
0
1990
1995
2000
2005
2010
2015
2020
2025
2030
Year

**Project, country:**	**Ruvuma, Tanzania**
Bioclime:	
Endemicity level(s):	Hyperendemic
MDA start year:	1999
Mean MDA coverage per year:	68.2

	1990			2020			2030		
	N	%	DALYs	N	%	DALYs	N	%	DALYs
Total populaton at risk	227	100	_	511	100	_	671	100	_
**Infection:**									
Mf-positive	127	56.2	_	28	5.4	_	3	0.5	_
Mf-positive (age 5+)	127	56.0	_	28	5.4	_	3	0.5	_
Worm infected	146	64.6	_	93	18.2	_	23	3.4	_
**Clinical manifestations:**									
Palpable nodules	64	28.3	1	12	2.4	<0.5	1	0.2	<0.5
Reversible skin disease:									
Severe itch	28	12.5	5	2	0.3	<0.5	<0.5	<0.05	<0.5
Reactive skin disease	24	10.5	1	<0.5	<0.05	<0.5	<0.5	<0.05	<0.5
**Reversible skin disease**	**52**	**23.0**	**6**	**2**	**0.3**	**<0.5**	**<0.5**	**<0.05**	**<0.5**
Irreversible skin disease:									
Mild depigmentation	6	2.4	<0.5	5	1.0	<0.5	4	0.6	<0.5
Severe depigmentation	8	3.7	1	7	1.4	<0.5	5	0.7	<0.5
Atrophy	<0.5	0.1	<0.5	<0.5	<0.05	<0.5	<0.5	<0.05	<0.5
Hanging groin	1	0.2	<0.5	<0.5	0.1	<0.5	<0.5	<0.05	<0.5
**Irreversible skin disease**	**15**	**6.5**	**1**	**13**	**2.5**	**1**	**9**	**1.3**	**<0.5**
**Total skin disease (OSD)**	**131**	**57.8**	**8**	**26**	**5.2**	**1**	**10**	**1.5**	**<0.5**
Eye disease (OED):									
Visual impairment	1	0.3	<0.5	<0.5	0.1	<0.5	<0.5	<0.05	<0.5
Blindness	1	0.4	7	<0.5	0.1	2	<0.5	<0.05	1
**Eye disease (OED)**	**2**	**0.7**	**7**	**1**	**0.2**	**3**	**<0.5**	**0.1**	**1**
**Total all manifestations**	**133**	**58.5**	**15**	**27**	**5.3**	**4**	**10**	**1.5**	**1**

Note 1: Population at risk, number of cases (N) and DALYs are presented in thousands.

Note 2: Total skin disease is defined as the sum of the number of cases with palpable nodules, reversible and irreversible skin disease.

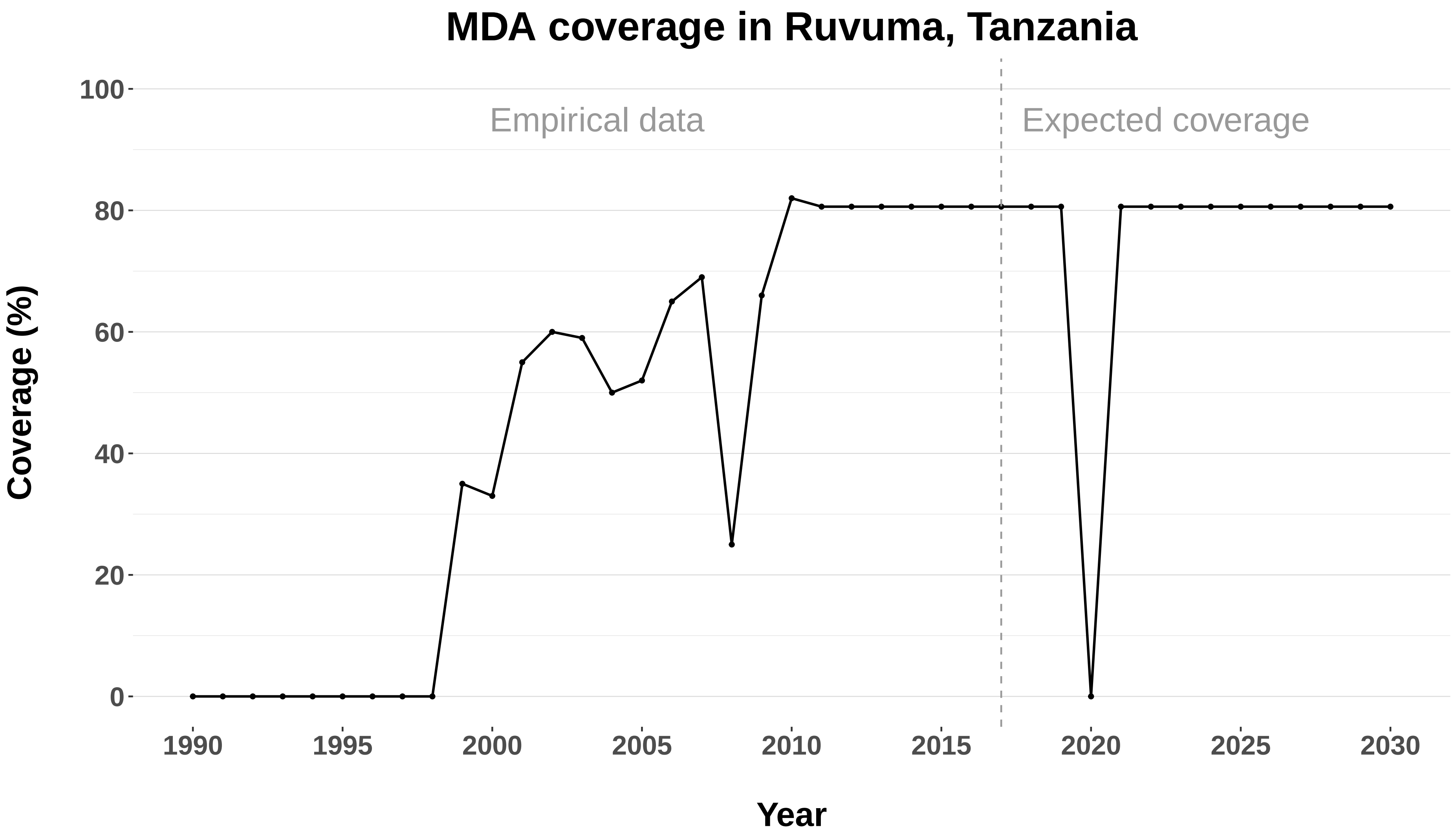
MDA coverage in Ruvuma, Tanzania
Empirical data
Expected coverage
Coverage (%)
100
80
60
40
20
0
1990
1995
2000
2005
2010
2015
2020
2025
2030
Year

**Project, country:**	**Tanga, Tanzania**
Bioclime:	
Endemicity level(s):	Mesoendemic
MDA start year:	2001
Mean MDA coverage per year:	73.1

	1990			2020			2030		
	N	%	DALYs	N	%	DALYs	N	%	DALYs
Total populaton at risk	178	100	_	401	100	_	526	100	_
**Infection:**									
Mf-positive	47	26.3	_	<0.5	0.1	_	<0.5	<0.05	_
Mf-positive (age 5+)	47	26.3	_	<0.5	0.1	_	<0.5	<0.05	_
Worm infected	62	34.9	_	8	2.1	_	<0.5	<0.05	_
**Clinical manifestations:**									
Palpable nodules	8	4.5	<0.5	<0.5	<0.05	<0.5	<0.5	<0.05	<0.5
Reversible skin disease:									
Severe itch	6	3.4	1	<0.5	<0.05	<0.5	<0.5	<0.05	<0.5
Reactive skin disease	4	2.3	<0.5	<0.5	<0.05	<0.5	<0.5	<0.05	<0.5
**Reversible skin disease**	**10**	**5.7**	**1**	**<0.5**	**<0.05**	**<0.5**	**<0.5**	**<0.05**	**<0.5**
Irreversible skin disease:									
Mild depigmentation	2	0.9	<0.5	1	0.3	<0.5	1	0.2	<0.5
Severe depigmentation	1	0.7	<0.5	1	0.2	<0.5	1	0.1	<0.5
Atrophy	<0.5	<0.05	<0.5	<0.5	<0.05	<0.5	<0.5	<0.05	<0.5
Hanging groin	<0.5	<0.05	<0.5	<0.5	<0.05	<0.5	<0.5	<0.05	<0.5
**Irreversible skin disease**	**3**	**1.6**	**<0.5**	**2**	**0.6**	**<0.5**	**1**	**0.3**	**<0.5**
**Total skin disease (OSD)**	**21**	**11.8**	**1**	**2**	**0.6**	**<0.5**	**1**	**0.3**	**<0.5**
Eye disease (OED):									
Visual impairment	<0.5	<0.05	<0.5	<0.5	<0.05	<0.5	<0.5	<0.05	<0.5
Blindness	<0.5	<0.05	<0.5	<0.5	<0.05	<0.5	<0.5	<0.05	<0.5
**Eye disease (OED)**	**<0.5**	**<0.05**	**<0.5**	**<0.5**	**<0.05**	**<0.5**	**<0.5**	**<0.05**	**<0.5**
**Total all manifestations**	**21**	**11.8**	**2**	**2**	**0.6**	**<0.5**	**1**	**0.3**	**<0.5**

Note 1: Population at risk, number of cases (N) and DALYs are presented in thousands.

Note 2: Total skin disease is defined as the sum of the number of cases with palpable nodules, reversible and irreversible skin disease.

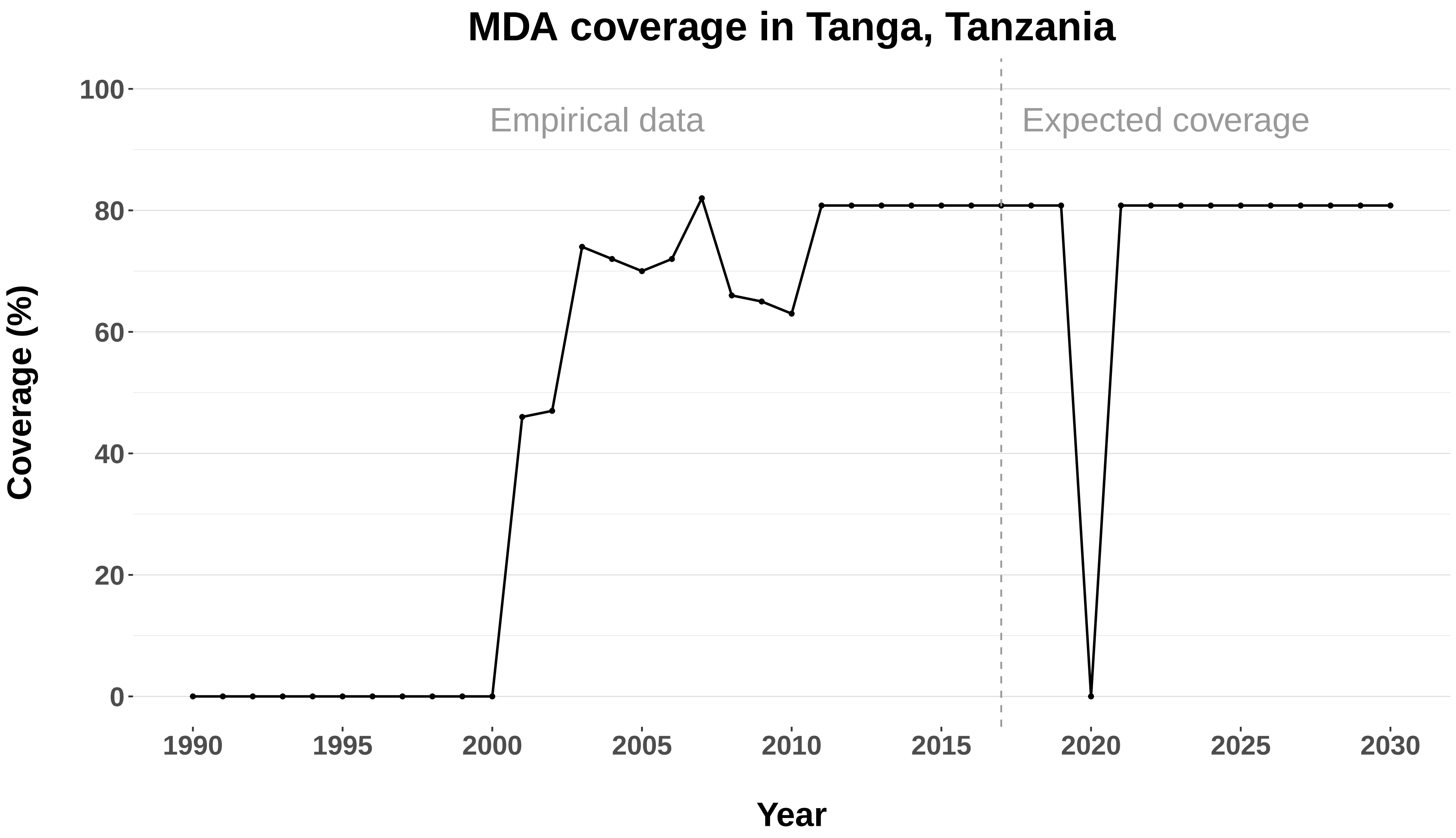
MDA coverage in Tanga, Tanzania
Empirical data
Expected coverage
Coverage (%)
100
80
60
40
20
0
1990
1995
2000
2005
2010
2015
2020
2025
2030
Year

**Project, country:**	**Tukuyu, Tanzania**
Bioclime:	
Endemicity level(s):	Mesoendemic
MDA start year:	2001
Mean MDA coverage per year:	75.0

	1990			2020			2030		
	N	%	DALYs	N	%	DALYs	N	%	DALYs
Total populaton at risk	65	100	_	147	100	_	193	100	_
**Infection:**									
Mf-positive	25	38.9	_	<0.5	0.3	_	<0.5	<0.05	_
Mf-positive (age 5+)	25	38.8	_	<0.5	0.3	_	<0.5	<0.05	_
Worm infected	31	48.0	_	5	3.7	_	<0.5	0.1	_
**Clinical manifestations:**									
Palpable nodules	8	13.0	<0.5	<0.5	0.1	<0.5	<0.5	<0.05	<0.5
Reversible skin disease:									
Severe itch	4	6.3	1	<0.5	<0.05	<0.5	<0.5	<0.05	<0.5
Reactive skin disease	3	4.7	<0.5	<0.5	<0.05	<0.5	<0.5	<0.05	<0.5
**Reversible skin disease**	**7**	**11.0**	**1**	**<0.5**	**<0.05**	**<0.5**	**<0.5**	**<0.05**	**<0.5**
Irreversible skin disease:									
Mild depigmentation	1	1.4	<0.5	1	0.5	<0.5	1	0.3	<0.5
Severe depigmentation	1	1.6	<0.5	1	0.5	<0.5	<0.5	0.2	<0.5
Atrophy	<0.5	<0.05	<0.5	<0.5	<0.05	<0.5	<0.5	<0.05	<0.5
Hanging groin	<0.5	<0.05	<0.5	<0.5	<0.05	<0.5	<0.5	<0.05	<0.5
**Irreversible skin disease**	**2**	**3.0**	**<0.5**	**2**	**1.1**	**<0.5**	**1**	**0.5**	**<0.5**
**Total skin disease (OSD)**	**18**	**27.1**	**1**	**2**	**1.2**	**<0.5**	**1**	**0.5**	**<0.5**
Eye disease (OED):									
Visual impairment	<0.5	0.1	<0.5	<0.5	<0.05	<0.5	<0.5	<0.05	<0.5
Blindness	<0.5	0.1	<0.5	<0.5	<0.05	<0.5	<0.5	<0.05	<0.5
**Eye disease (OED)**	**<0.5**	**0.1**	**<0.5**	**<0.5**	**<0.05**	**<0.5**	**<0.5**	**<0.05**	**<0.5**
**Total all manifestations**	**18**	**27.2**	**1**	**2**	**1.2**	**<0.5**	**1**	**0.5**	**<0.5**

Note 1: Population at risk, number of cases (N) and DALYs are presented in thousands.

Note 2: Total skin disease is defined as the sum of the number of cases with palpable nodules, reversible and irreversible skin disease.

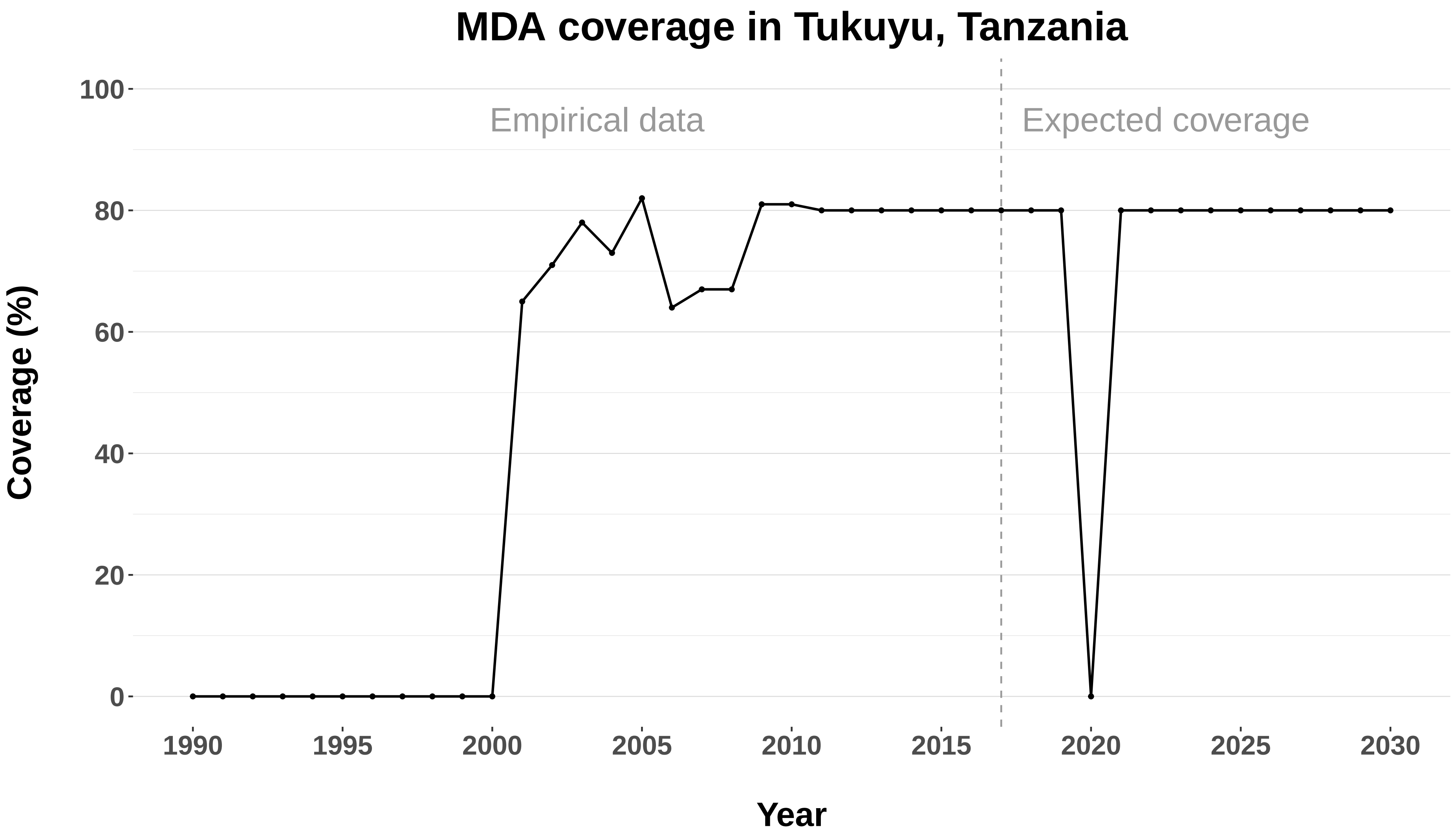
MDA coverage in Tukuyu, Tanzania
Empirical data
Expected coverage
Coverage (%)
100
80
60
40
20
0
1990
1995
2000
2005
2010
2015
2020
2025
2030
Year

**Project, country:**	**Tunduru, Tanzania**
Bioclime:	
Endemicity level(s):	Hyperendemic
MDA start year:	2005
Mean MDA coverage per year:	77.3

	1990			2020			2030		
	N	%	DALYs	N	%	DALYs	N	%	DALYs
Total populaton at risk	71	100	_	159	100	_	209	100	_
**Infection:**									
Mf-positive	33	46.7	_	3	1.9	_	<0.5	0.1	_
Mf-positive (age 5+)	33	46.6	_	3	1.9	_	<0.5	0.1	_
Worm infected	40	56.4	_	19	11.7	_	2	0.8	_
**Clinical manifestations:**									
Palpable nodules	13	18.0	<0.5	1	0.9	<0.5	<0.5	<0.05	<0.5
Reversible skin disease:									
Severe itch	6	8.1	1	<0.5	0.2	<0.5	<0.5	<0.05	<0.5
Reactive skin disease	4	6.2	<0.5	<0.5	<0.05	<0.5	<0.5	<0.05	<0.5
**Reversible skin disease**	**10**	**14.3**	**1**	**<0.5**	**0.2**	**<0.5**	**<0.5**	**<0.05**	**<0.5**
Irreversible skin disease:									
Mild depigmentation	1	1.7	<0.5	1	0.8	<0.5	1	0.4	<0.5
Severe depigmentation	1	2.1	<0.5	1	0.9	<0.5	1	0.5	<0.5
Atrophy	<0.5	<0.05	<0.5	<0.5	<0.05	<0.5	<0.5	<0.05	<0.5
Hanging groin	<0.5	0.1	<0.5	<0.5	<0.05	<0.5	<0.5	<0.05	<0.5
**Irreversible skin disease**	**3**	**3.9**	**<0.5**	**3**	**1.7**	**<0.5**	**2**	**0.9**	**<0.5**
**Total skin disease (OSD)**	**25**	**36.1**	**1**	**4**	**2.8**	**<0.5**	**2**	**0.9**	**<0.5**
Eye disease (OED):									
Visual impairment	<0.5	0.1	<0.5	<0.5	<0.05	<0.5	<0.5	<0.05	<0.5
Blindness	<0.5	0.1	1	<0.5	<0.05	<0.5	<0.5	<0.05	<0.5
**Eye disease (OED)**	**<0.5**	**0.2**	**1**	**<0.5**	**0.1**	**<0.5**	**<0.5**	**<0.05**	**<0.5**
**Total all manifestations**	**26**	**36.4**	**2**	**5**	**2.9**	**<0.5**	**2**	**1.0**	**<0.5**

Note 1: Population at risk, number of cases (N) and DALYs are presented in thousands.

Note 2: Total skin disease is defined as the sum of the number of cases with palpable nodules, reversible and irreversible skin disease.

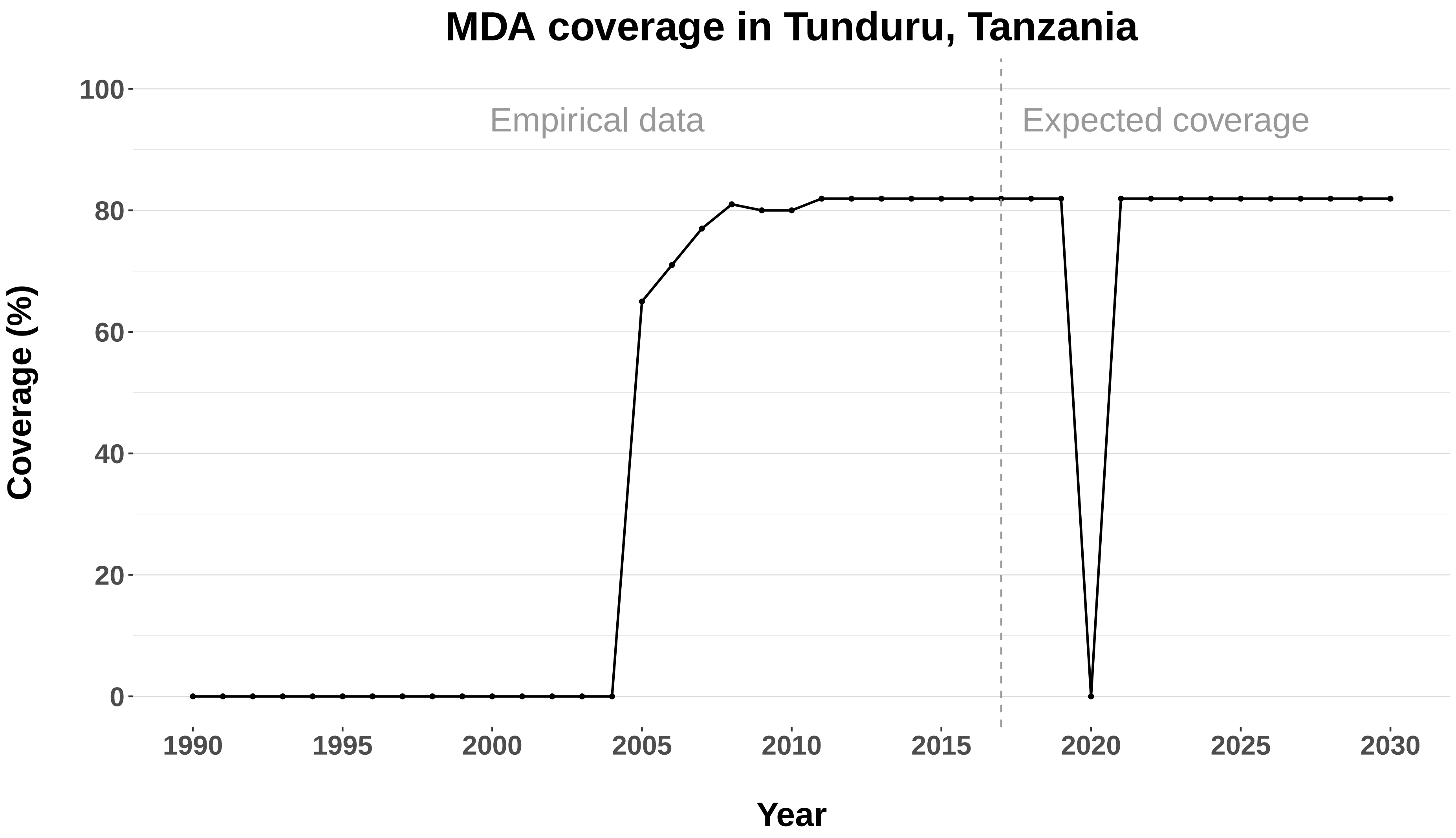
MDA coverage in Tunduru, Tanzania
Empirical data
Expected coverage
Coverage (%)
100
80
60
40
20
0
1990
1995
2000
2005
2010
2015
2020
2025
2030
Year

**Project, country:**	**P5Uganda, Uganda**
Bioclime:	
Endemicity level(s):	Hypoendemic
MDA start year:	1997
Mean MDA coverage per year:	72.8

	1990			2020			2030		
	N	%	DALYs	N	%	DALYs	N	%	DALYs
Total populaton at risk	131	100	_	295	100	_	387	100	_
**Infection:**									
Mf-positive	31	23.4	_	<0.5	<0.05	_	<0.5	<0.05	_
Mf-positive (age 5+)	31	23.3	_	<0.5	<0.05	_	<0.5	<0.05	_
Worm infected	41	31.3	_	<0.5	0.1	_	<0.5	<0.05	_
**Clinical manifestations:**									
Palpable nodules	4	3.2	<0.5	<0.5	<0.05	<0.5	<0.5	<0.05	<0.5
Reversible skin disease:									
Severe itch	4	3.0	1	<0.5	<0.05	<0.5	<0.5	<0.05	<0.5
Reactive skin disease	3	1.9	<0.5	<0.5	<0.05	<0.5	<0.5	<0.05	<0.5
**Reversible skin disease**	**6**	**4.9**	**1**	**<0.5**	**<0.05**	**<0.5**	**<0.5**	**<0.05**	**<0.5**
Irreversible skin disease:									
Mild depigmentation	1	0.8	<0.5	1	0.2	<0.5	<0.5	0.1	<0.5
Severe depigmentation	1	0.7	<0.5	<0.5	0.2	<0.5	<0.5	0.1	<0.5
Atrophy	<0.5	<0.05	<0.5	<0.5	<0.05	<0.5	<0.5	<0.05	<0.5
Hanging groin	<0.5	<0.05	<0.5	<0.5	<0.05	<0.5	<0.5	<0.05	<0.5
**Irreversible skin disease**	**2**	**1.5**	**<0.5**	**1**	**0.4**	**<0.5**	**1**	**0.2**	**<0.5**
**Total skin disease (OSD)**	**13**	**9.6**	**1**	**1**	**0.4**	**<0.5**	**1**	**0.2**	**<0.5**
Eye disease (OED):									
Visual impairment	<0.5	<0.05	<0.5	<0.5	<0.05	<0.5	<0.5	<0.05	<0.5
Blindness	<0.5	<0.05	<0.5	<0.5	<0.05	<0.5	<0.5	<0.05	<0.5
**Eye disease (OED)**	**<0.5**	**<0.05**	**<0.5**	**<0.5**	**<0.05**	**<0.5**	**<0.5**	**<0.05**	**<0.5**
**Total all manifestations**	**13**	**9.6**	**1**	**1**	**0.4**	**<0.5**	**1**	**0.2**	**<0.5**

Note 1: Population at risk, number of cases (N) and DALYs are presented in thousands.

Note 2: Total skin disease is defined as the sum of the number of cases with palpable nodules, reversible and irreversible skin disease.

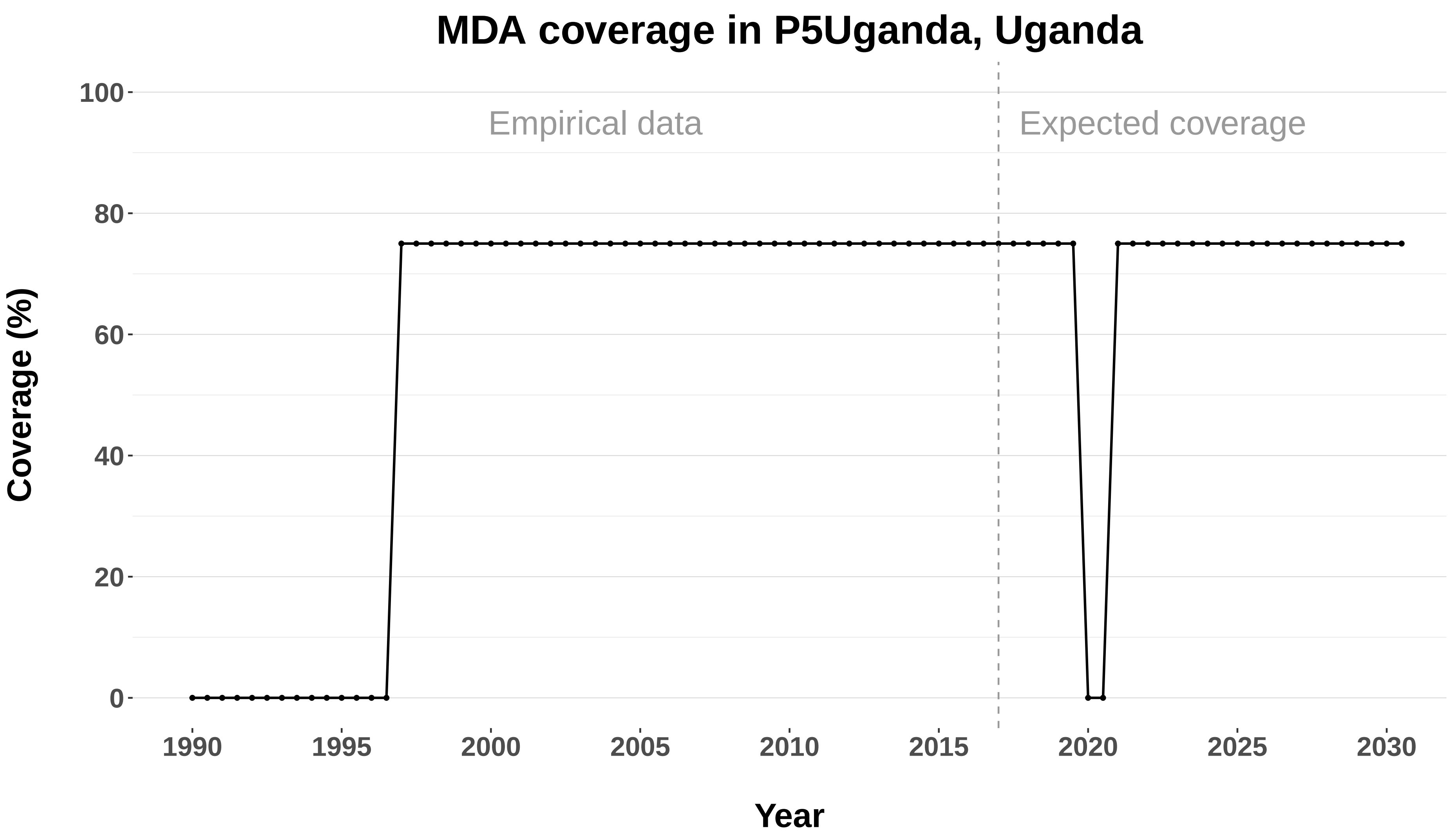

MDA coverage in P5Uganda, Uganda
Empirical data
Expected coverage
Coverage (%)
100
80
60
40
20
0
1990
1995
2000
2005
2010
2015
2020
2025
2030
Year

**Project, country:**	**Phase 1, Uganda**
Bioclime:	
Endemicity level(s):	Hyperendemic
MDA start year:	1999
Mean MDA coverage per year:	73.2

	1990			2020			2030		
	N	%	DALYs	N	%	DALYs	N	%	DALYs
Total populaton at risk	205	100	_	463	100	_	607	100	_
**Infection:**									
Mf-positive	58	28.3	_	<0.5	<0.05	_	<0.5	<0.05	_
Mf-positive (age 5+)	58	28.2	_	<0.5	<0.05	_	<0.5	<0.05	_
Worm infected	75	36.5	_	1	0.3	_	<0.5	<0.05	_
**Clinical manifestations:**									
Palpable nodules	12	5.6	<0.5	<0.5	<0.05	<0.5	<0.5	<0.05	<0.5
Reversible skin disease:									
Severe itch	8	3.9	1	<0.5	<0.05	<0.5	<0.5	<0.05	<0.5
Reactive skin disease	5	2.6	<0.5	<0.5	<0.05	<0.5	<0.5	<0.05	<0.5
**Reversible skin disease**	**13**	**6.5**	**2**	**<0.5**	**<0.05**	**<0.5**	**<0.5**	**<0.05**	**<0.5**
Irreversible skin disease:									
Mild depigmentation	2	1.0	<0.5	1	0.3	<0.5	1	0.1	<0.5
Severe depigmentation	2	0.9	<0.5	1	0.2	<0.5	1	0.1	<0.5
Atrophy	<0.5	<0.05	<0.5	<0.5	<0.05	<0.5	<0.5	<0.05	<0.5
Hanging groin	<0.5	<0.05	<0.5	<0.5	<0.05	<0.5	<0.5	<0.05	<0.5
**Irreversible skin disease**	**4**	**1.9**	**<0.5**	**2**	**0.5**	**<0.5**	**1**	**0.2**	**<0.5**
**Total skin disease (OSD)**	**29**	**14.0**	**2**	**2**	**0.5**	**<0.5**	**1**	**0.2**	**<0.5**
Eye disease (OED):									
Visual impairment	<0.5	<0.05	<0.5	<0.5	<0.05	<0.5	<0.5	<0.05	<0.5
Blindness	<0.5	<0.05	<0.5	<0.5	<0.05	<0.5	<0.5	<0.05	<0.5
**Eye disease (OED)**	**<0.5**	**<0.05**	**<0.5**	**<0.5**	**<0.05**	**<0.5**	**<0.5**	**<0.05**	**<0.5**
**Total all manifestations**	**29**	**14.0**	**2**	**2**	**0.5**	**<0.5**	**1**	**0.2**	**<0.5**

Note 1: Population at risk, number of cases (N) and DALYs are presented in thousands.

Note 2: Total skin disease is defined as the sum of the number of cases with palpable nodules, reversible and irreversible skin disease.

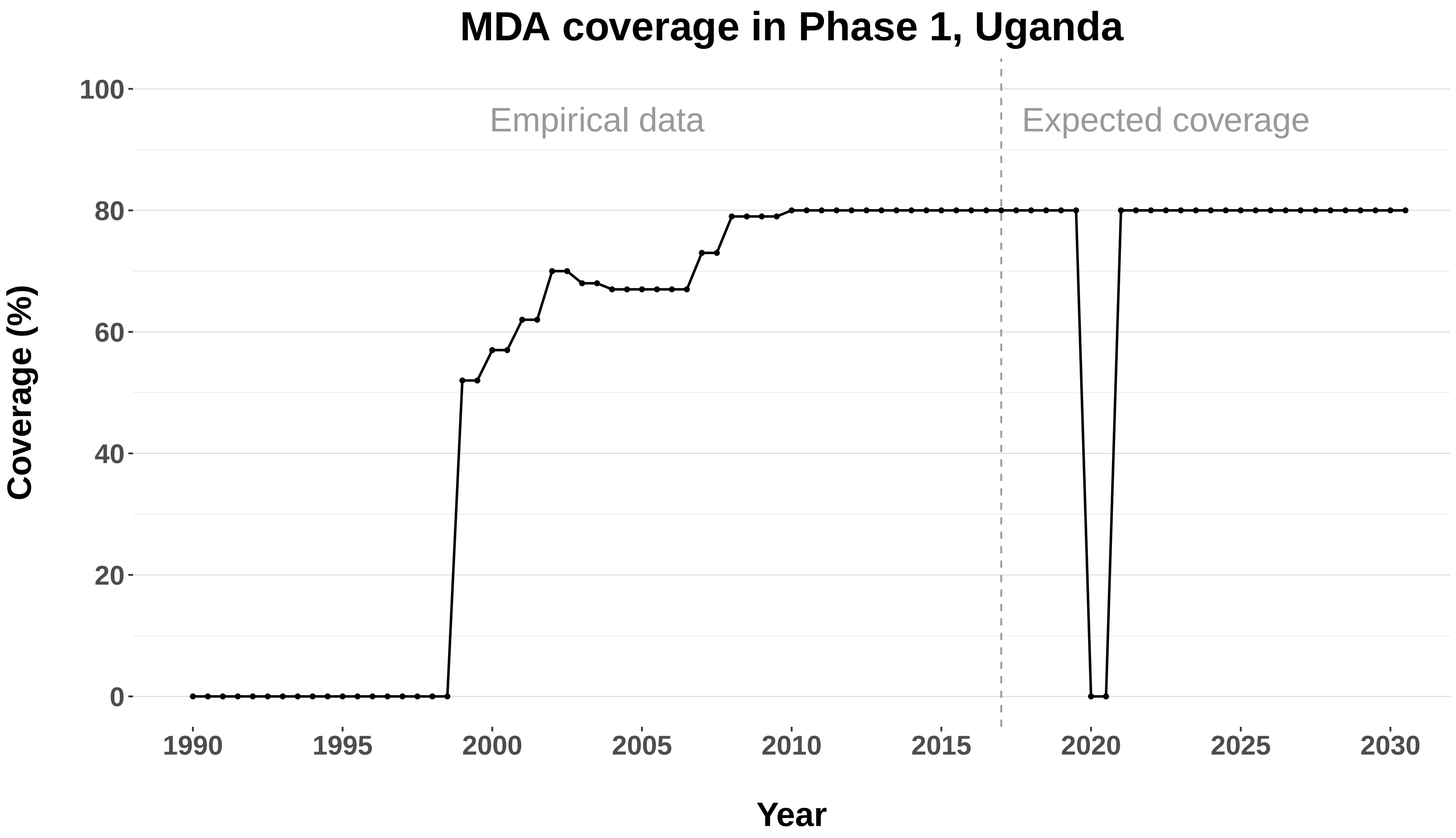
MDA coverage in Phase 1, Uganda
Empirical data
Expected coverage
Coverage (%)
0
20
40
60
80
100
1990
1995
2000
2005
2010
2015
2020
2025
2030
Year

**Project, country:**	**Phase 2, Uganda**
Bioclime:	
Endemicity level(s):	Hypoendemic
MDA start year:	1999
Mean MDA coverage per year:	72.4

	1990			2020			2030		
	N	%	DALYs	N	%	DALYs	N	%	DALYs
Total populaton at risk	399	100	_	900	100	_	1,181	100	_
**Infection:**									
Mf-positive	90	22.6	_	<0.5	<0.05	_	<0.5	<0.05	_
Mf-positive (age 5+)	90	22.6	_	<0.5	<0.05	_	<0.5	<0.05	_
Worm infected	119	29.8	_	1	0.1	_	<0.5	<0.05	_
**Clinical manifestations:**									
Palpable nodules	10	2.4	<0.5	<0.5	<0.05	<0.5	<0.5	<0.05	<0.5
Reversible skin disease:									
Severe itch	11	2.9	2	<0.5	<0.05	<0.5	<0.5	<0.05	<0.5
Reactive skin disease	8	1.9	<0.5	<0.5	<0.05	<0.5	<0.5	<0.05	<0.5
**Reversible skin disease**	**19**	**4.8**	**2**	**<0.5**	**<0.05**	**<0.5**	**<0.5**	**<0.05**	**<0.5**
Irreversible skin disease:									
Mild depigmentation	3	0.8	<0.5	2	0.2	<0.5	1	0.1	<0.5
Severe depigmentation	3	0.7	<0.5	1	0.2	<0.5	1	0.1	<0.5
Atrophy	<0.5	<0.05	<0.5	<0.5	<0.05	<0.5	<0.5	<0.05	<0.5
Hanging groin	<0.5	<0.05	<0.5	<0.5	<0.05	<0.5	<0.5	<0.05	<0.5
**Irreversible skin disease**	**6**	**1.5**	**<0.5**	**3**	**0.4**	**<0.5**	**2**	**0.2**	**<0.5**
**Total skin disease (OSD)**	**35**	**8.7**	**3**	**3**	**0.4**	**<0.5**	**2**	**0.2**	**<0.5**
Eye disease (OED):									
Visual impairment	<0.5	<0.05	<0.5	<0.5	<0.05	<0.5	<0.5	<0.05	<0.5
Blindness	<0.5	<0.05	<0.5	<0.5	<0.05	<0.5	<0.5	<0.05	<0.5
**Eye disease (OED)**	**<0.5**	**<0.05**	**<0.5**	**<0.5**	**<0.05**	**<0.5**	**<0.5**	**<0.05**	**<0.5**
**Total all manifestations**	**35**	**8.7**	**3**	**3**	**0.4**	**<0.5**	**2**	**0.2**	**<0.5**

Note 1: Population at risk, number of cases (N) and DALYs are presented in thousands.

Note 2: Total skin disease is defined as the sum of the number of cases with palpable nodules, reversible and irreversible skin disease.

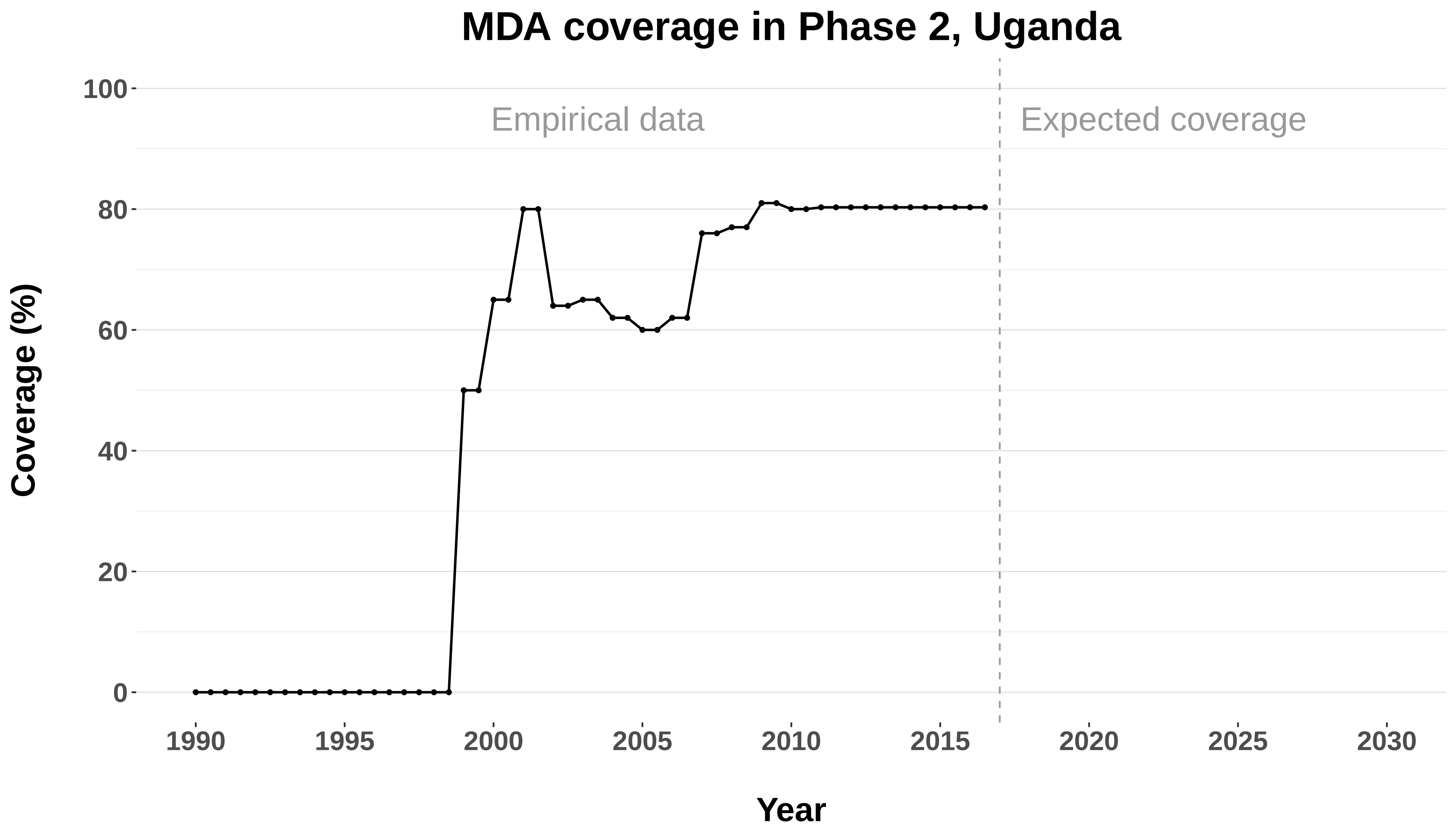

Note: We assume no ivermectin MDA for Phase 2 and Phase 4 as the foci in these APOC-projects are currently under post-treatment surveillance (PTS), or completed the PTS successfully [5-10][‡, page 354].

**Project, country:**	**Phase 3, Uganda**
Bioclime:	
Endemicity level(s):	Hyperendemic
MDA start year:	1999
Mean MDA coverage per year:	63.8

	1990			2020			2030		
	N	%	DALYs	N	%	DALYs	N	%	DALYs
Total populaton at risk	736	100	_	1,660	100	_	2,178	100	_
**Infection:**									
Mf-positive	336	45.7	_	3	0.2	_	<0.5	<0.05	_
Mf-positive (age 5+)	335	45.5	_	3	0.2	_	<0.5	<0.05	_
Worm infected	397	54.0	_	67	4.0	_	3	0.1	_
**Clinical manifestations:**									
Palpable nodules	145	19.7	2	4	0.2	<0.5	<0.5	<0.05	<0.5
Reversible skin disease:									
Severe itch	67	9.2	12	1	0.1	<0.5	<0.5	<0.05	<0.5
Reactive skin disease	55	7.5	3	<0.5	<0.05	<0.5	<0.5	<0.05	<0.5
**Reversible skin disease**	**123**	**16.7**	**15**	**1**	**0.1**	**<0.5**	**<0.5**	**<0.05**	**<0.5**
Irreversible skin disease:									
Mild depigmentation	14	1.9	<0.5	11	0.7	<0.5	8	0.3	<0.5
Severe depigmentation	20	2.7	1	14	0.8	1	9	0.4	1
Atrophy	<0.5	0.1	<0.5	<0.5	<0.05	<0.5	<0.5	<0.05	<0.5
Hanging groin	1	0.1	<0.5	<0.5	<0.05	<0.5	<0.5	<0.05	<0.5
**Irreversible skin disease**	**35**	**4.7**	**2**	**25**	**1.5**	**1**	**16**	**0.7**	**1**
**Total skin disease (OSD)**	**302**	**41.1**	**18**	**30**	**1.8**	**1**	**16**	**0.8**	**1**
Eye disease (OED):									
Visual impairment	1	0.2	<0.5	1	0.1	<0.5	<0.5	<0.05	<0.5
Blindness	2	0.3	14	<0.5	<0.05	3	<0.5	<0.05	1
**Eye disease (OED)**	**3**	**0.5**	**14**	**1**	**0.1**	**3**	**1**	**<0.05**	**1**
**Total all manifestations**	**306**	**41.6**	**32**	**31**	**1.9**	**5**	**17**	**0.8**	**2**

Note 1: Population at risk, number of cases (N) and DALYs are presented in thousands.

Note 2: Total skin disease is defined as the sum of the number of cases with palpable nodules, reversible and irreversible skin disease.

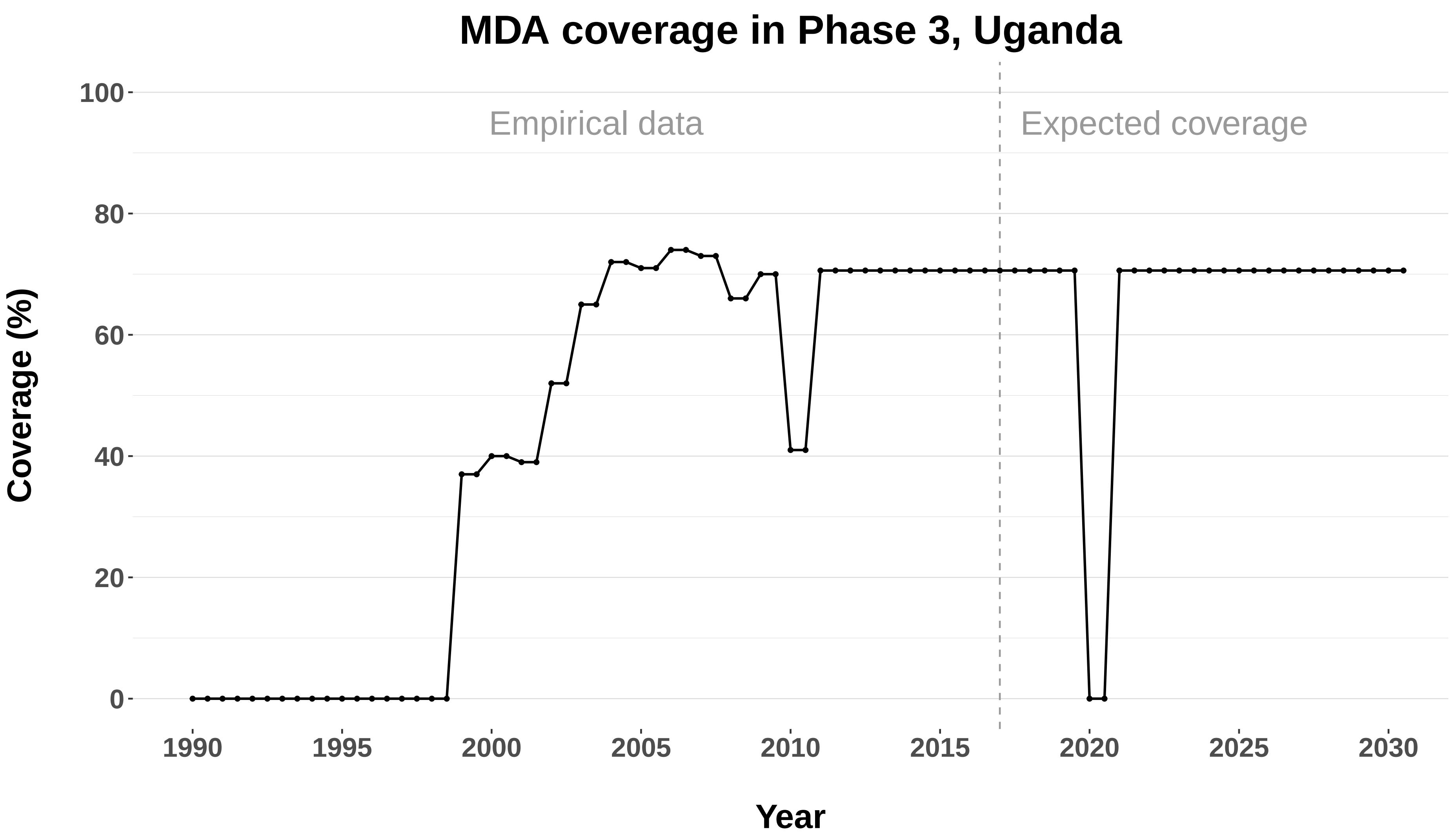
MDA coverage in Phase 3, Uganda
Empirical data
Expected coverage
Coverage (%)
100
80
60
40
20
0
1990
1995
2000
2005
2010
2015
2020
2025
2030
Year

**Project, country:**	**Phase 4, Uganda**
Bioclime:	
Endemicity level(s):	Hyperendemic
MDA start year:	1999
Mean MDA coverage per year:	77.2

	1990			2020			2030		
	N	%	DALYs	N	%	DALYs	N	%	DALYs
Total populaton at risk	416	100	_	940	100	_	1,233	100	_
**Infection:**									
Mf-positive	114	27.4	_	<0.5	<0.05	_	<0.5	<0.05	_
Mf-positive (age 5+)	114	27.4	_	<0.5	<0.05	_	<0.5	<0.05	_
Worm infected	148	35.5	_	1	0.1	_	<0.5	<0.05	_
**Clinical manifestations:**									
Palpable nodules	21	5.1	<0.5	<0.5	<0.05	<0.5	<0.5	<0.05	<0.5
Reversible skin disease:									
Severe itch	15	3.7	3	<0.5	<0.05	<0.5	<0.5	<0.05	<0.5
Reactive skin disease	11	2.6	<0.5	<0.5	<0.05	<0.5	<0.5	<0.05	<0.5
**Reversible skin disease**	**26**	**6.2**	**3**	**<0.5**	**<0.05**	**<0.5**	**<0.5**	**<0.05**	**<0.5**
Irreversible skin disease:									
Mild depigmentation	4	0.9	<0.5	2	0.3	<0.5	2	0.1	<0.5
Severe depigmentation	4	0.8	<0.5	2	0.2	<0.5	1	0.1	<0.5
Atrophy	<0.5	<0.05	<0.5	<0.5	<0.05	<0.5	<0.5	<0.05	<0.5
Hanging groin	<0.5	<0.05	<0.5	<0.5	<0.05	<0.5	<0.5	<0.05	<0.5
**Irreversible skin disease**	**7**	**1.8**	**<0.5**	**4**	**0.5**	**<0.5**	**3**	**0.2**	**<0.5**
**Total skin disease (OSD)**	**54**	**13.1**	**4**	**4**	**0.5**	**<0.5**	**3**	**0.2**	**<0.5**
Eye disease (OED):									
Visual impairment	<0.5	<0.05	<0.5	<0.5	<0.05	<0.5	<0.5	<0.05	<0.5
Blindness	<0.5	<0.05	<0.5	<0.5	<0.05	<0.5	<0.5	<0.05	<0.5
**Eye disease (OED)**	**<0.5**	**<0.05**	**<0.5**	**<0.5**	**<0.05**	**<0.5**	**<0.5**	**<0.05**	**<0.5**
**Total all manifestations**	**55**	**13.1**	**4**	**4**	**0.5**	**<0.5**	**3**	**0.2**	**<0.5**

Note 1: Population at risk, number of cases (N) and DALYs are presented in thousands.

Note 2: Total skin disease is defined as the sum of the number of cases with palpable nodules, reversible and irreversible skin disease.

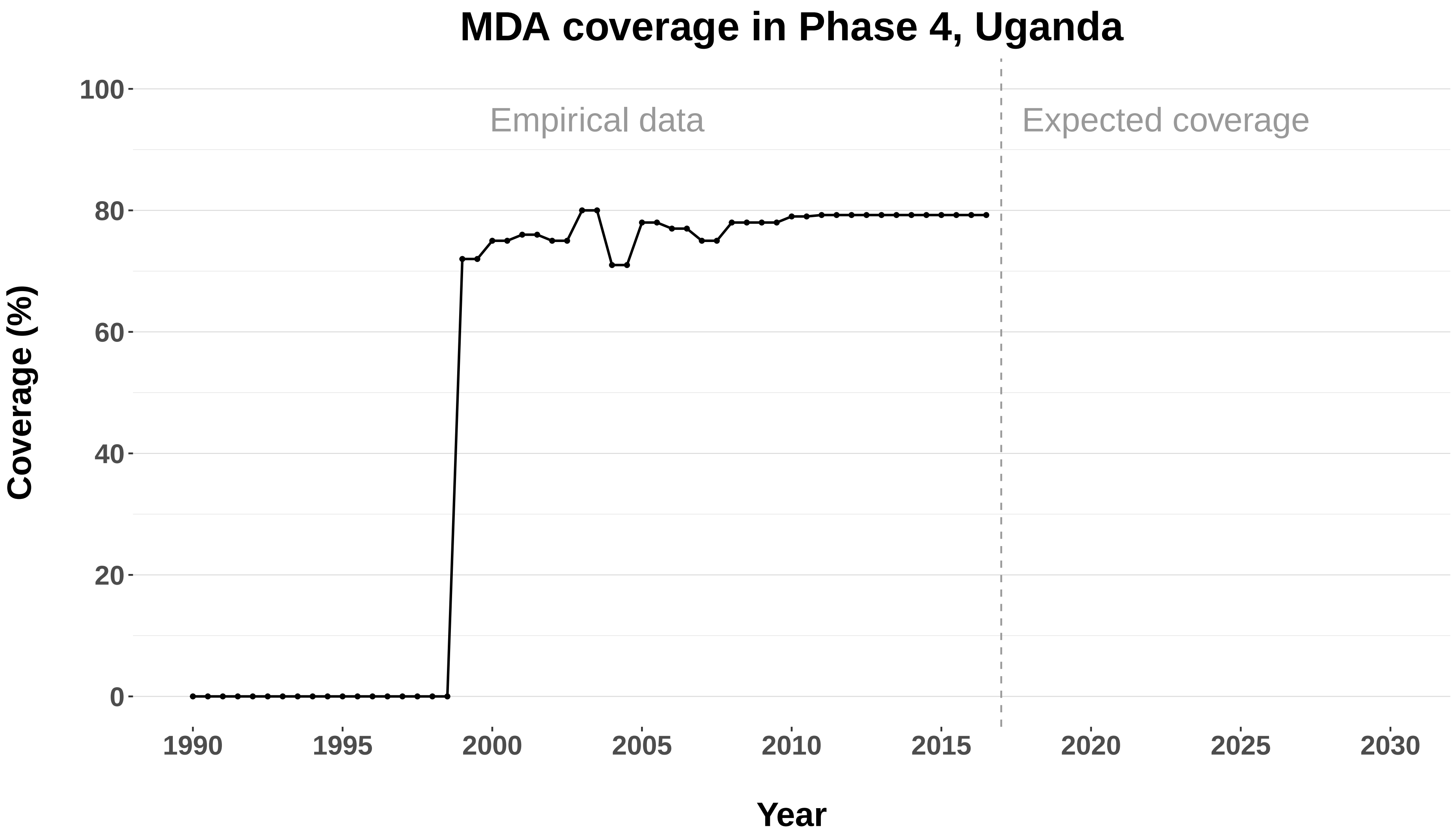

Note: We assume no ivermectin MDA for Phase 2 and Phase 4 as the foci in these APOC-projects are currently under post-treatment surveillance (PTS), or completed the PTS successfully [5-10][‡, page 354].

**Project, country:**	**Phase 5, Uganda**
Bioclime:	
Endemicity level(s):	Hyperendemic
MDA start year:	2012
Mean MDA coverage per year:	71.1

	1990			2020			2030		
	N	%	DALYs	N	%	DALYs	N	%	DALYs
Total populaton at risk	268	100	_	605	100	_	794	100	_
**Infection:**									
Mf-positive	79	29.6	_	5	0.8	_	<0.5	<0.05	_
Mf-positive (age 5+)	79	29.6	_	5	0.8	_	<0.5	<0.05	_
Worm infected	101	37.6	_	79	13.1	_	2	0.2	_
**Clinical manifestations:**									
Palpable nodules	18	6.8	<0.5	6	1.0	<0.5	<0.5	<0.05	<0.5
Reversible skin disease:									
Severe itch	12	4.3	2	2	0.3	<0.5	<0.5	<0.05	<0.5
Reactive skin disease	8	3.0	<0.5	<0.5	<0.05	<0.5	<0.5	<0.05	<0.5
**Reversible skin disease**	**20**	**7.3**	**2**	**2**	**0.3**	**<0.5**	**<0.5**	**<0.05**	**<0.5**
Irreversible skin disease:									
Mild depigmentation	3	1.1	<0.5	3	0.5	<0.5	2	0.2	<0.5
Severe depigmentation	3	1.0	<0.5	3	0.4	<0.5	2	0.2	<0.5
Atrophy	<0.5	<0.05	<0.5	<0.5	<0.05	<0.5	<0.5	<0.05	<0.5
Hanging groin	<0.5	<0.05	<0.5	<0.5	<0.05	<0.5	<0.5	<0.05	<0.5
**Irreversible skin disease**	**6**	**2.1**	**<0.5**	**5**	**0.9**	**<0.5**	**4**	**0.5**	**<0.5**
**Total skin disease (OSD)**	**43**	**16.2**	**3**	**13**	**2.2**	**1**	**4**	**0.5**	**<0.5**
Eye disease (OED):									
Visual impairment	<0.5	<0.05	<0.5	<0.5	<0.05	<0.5	<0.5	<0.05	<0.5
Blindness	<0.5	<0.05	<0.5	<0.5	<0.05	<0.5	<0.5	<0.05	<0.5
**Eye disease (OED)**	**<0.5**	**0.1**	**<0.5**	**<0.5**	**<0.05**	**<0.5**	**<0.5**	**<0.05**	**<0.5**
**Total all manifestations**	**44**	**16.2**	**3**	**14**	**2.2**	**1**	**4**	**0.5**	**<0.5**

Note 1: Population at risk, number of cases (N) and DALYs are presented in thousands.

Note 2: Total skin disease is defined as the sum of the number of cases with palpable nodules, reversible and irreversible skin disease.

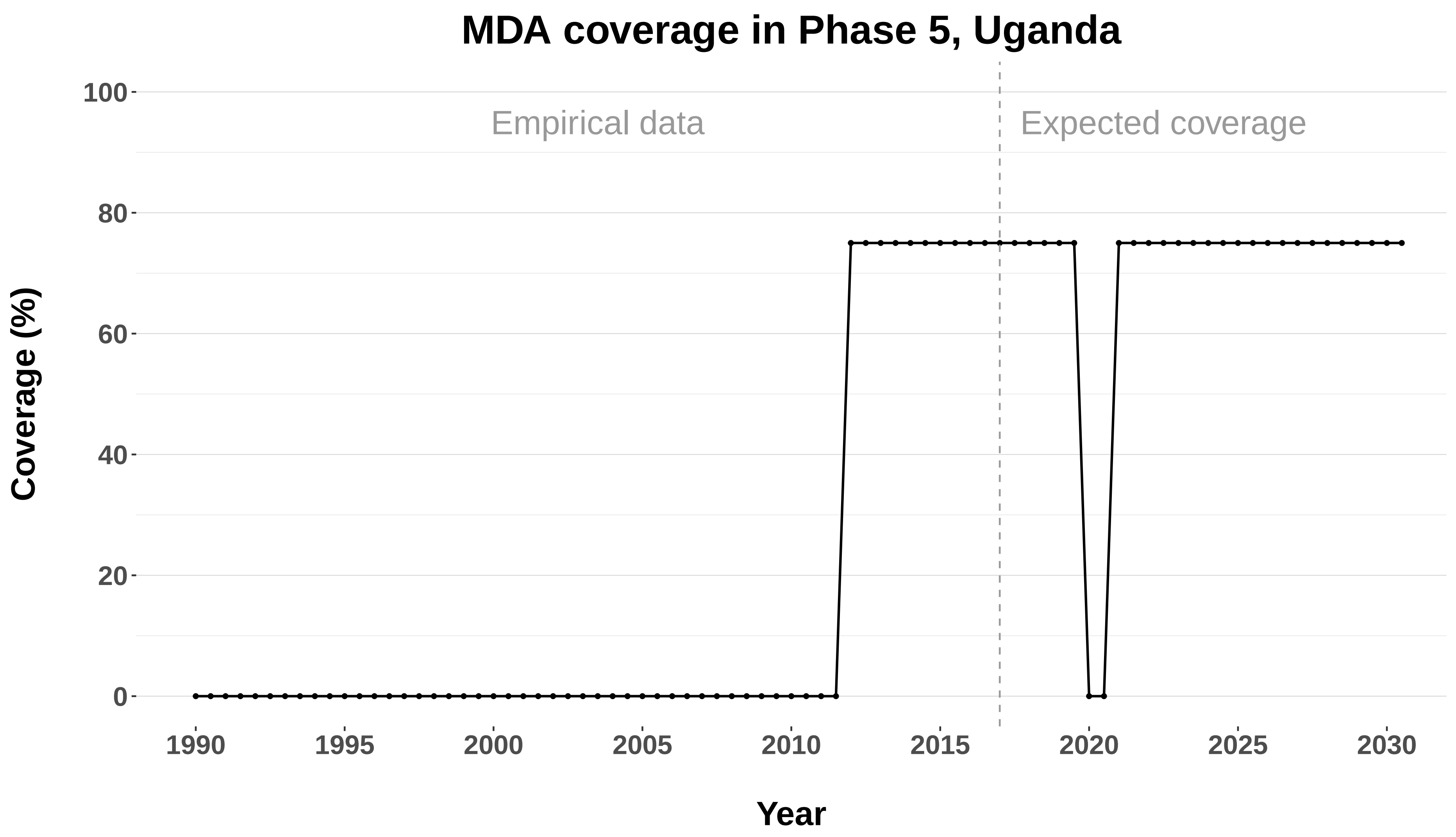

MDA coverage in Phase 5, Uganda
Empirical data
Expected coverage
Coverage (%)
100
80
60
40
20
0
1990
1995
2000
2005
2010
2015
2020
2025
2030
Year

‡ References:

1. ESPEN Portal. Onchocerciasis maps. Expand Spec Proj Elimin Neglected Trop Dis (ESPEN) World Health Organization Regional Office for Africa. Available at: http://espen.afro.who.int/diseases/onchocerciasis. Accessed on: 10 May 2021.
2. Herrador Z, Garcia B, Ncogo P, Perteguer MJ, Rubio JM, Rivas E, et al. Interruption of onchocerciasis transmission in Bioko Island: Accelerating the movement from control to elimination in Equatorial Guinea. Wanji S. PLoS Negl Trop Dis. Public Library of Science; 2018;12: e0006471.
3. Traoré S, Wilson MD, Sima A, Barro T, Diallo A, Ak? A, et al. The elimination of the onchocerciasis vector from the island of Bioko as a result of larviciding by the WHO African Programme for Onchocerciasis Control. Acta Trop. 2009;111: 211-218.
4. Richards FO, Eigege A, Umaru J, Kahansim B, Adelamo S, et al. (2020) The interruption of transmission of human onchocerciasis by an annual mass drug administration program in Plateau and Nasarawa States, Nigeria. Am J Trop Med Hyg 102: 582–592.
5. Katabarwa MN, Lakwo T, Habomugisha P, Unnasch TR, Garms R, et al. (2018) After 70 years of fighting an age-old scourge, onchocerciasis in Uganda, the end is in sight. Int Health10: i79–i88.
6. Katabarwa MN, Lakwo T, Habomugisha P, Agunyo S, Byamukama E, et al. (2014) Transmission of Onchocerca volvulus by Simulium neavei in Mount Elgon focus of eastern Uganda has been interrupted. Am J Trop Med Hyg 90: 1159–1166.
7. Lakwo TL, Garms R, Rubaale T, Katabarwa M, Walsh F, et al. (2013) The disappearance of onchocerciasis from the Itwara focus, western Uganda after elimination of the vector Simulium neavei and 19 years of annual ivermectin treatments. Acta Trop 126: 218–221.
8. Katabarwa MN, Walsh F, Habomugisha P, Lakwo TL, Agunyo S, et al. (2012) Transmission of onchocerciasis in Wadelai focus of northwestern Uganda has been interrupted and the disease eliminated. J Parasitol Res 2012.
9. Katabarwa MN, Katamanywa J, Lakwo T, Habomugisha P, Byamukama E, et al. (2016) The imaramagambo onchocerciasis focus in southwestern Uganda: Interruption of transmission after disappearance of the vector simulium neavei and its associated freshwater crabs. Am J Trop Med Hyg 95: 417–425.
10. Katabarwa MN, Habomugisha P, Khainza A, Oguttu DW, Byamukama E, et al. (2020) Historical elimination of onchocerciasis from victoria nile focus in Central Uganda verified using WHO criteria. Am J Trop Med Hyg 102: 1411–1416.
