# Supplementary material for: The burden of skin disease and eye disease due to onchocerciasis in countries formerly under the African Programme for Onchocerciasis Control mandate for 1990, 2020, and 2030
Source: PLoS Negl Trop Dis. 2021 Jul 26;15(7):e0009604. doi: 10.1371/journal.pntd.0009604 (PMC8312930; doi:10.1371/journal.pntd.0009604)
Supplement: Attachment — Submitted filename: Reaction to the reviewers_2021.06.15.pdf [file pntd.0009604.s005.pdf]

Reply to the reviewers

Manuscript: PNTD-D-20-01989

The burden of skin disease and eye disease due to onchocerciasis in Africa for 1990, 2020, and 2030

Natalie V.S. Vinkeles Melchers, Wilma A. Stolk, Welmoed van Loon, Belén Pedrique, Roel Bakker, Michele E. Murdoch, Sake J. de Vlas, Luc E. Coffeng

We would like to thank all reviewers for their comments and suggestions for improvement. We numbered the comments of the reviewers consecutively for ease of reference. The line numbers in our answers correspond to the PDF version of the track changed manuscript.

Reviewer's Responses to Questions

Methods

Reviewer #1

Are the objectives of the study clearly articulated with a clear testable hypothesis stated? Yes
Is the study design appropriate to address the stated objectives? Yes
Were correct statistical analysis used to support conclusions? Yes

Reviewer #2

1. **The objectives are clearly elucidated but the process is very theoretical and although well explained it is unrelated to the reality in the field in East and Central Africa.**

Response: There is little information available about current number of people infected and with onchocercal morbidity in Africa. The aim of this modelling study is to provide an indication of these numbers and the burden of disease due to onchocerciasis now and in the past. We also aim to understand how these numbers are likely to change over time thanks to mass drug administration and vector control, and henceforth what we can expect in 2030; the target year of the NTD Roadmap 2021-2030. A strength of this study is that we use existing data as input in our models, giving the most realistic possible representation of the actual epidemiological situation in onchocerciasis-endemic countries of east and central Africa. We acknowledge that this study has its limitations, as the work very much depends on assumptions made. Note that we have refined some uncertain assumptions guided by specific review comments, e.g. concerning history of control in conflict areas. We may not have been able to capture all uncertainties, but we think we made a significant improvement of our work through updated analyses. Please see also our responses to comments #2, #4, #8-#11.

Reviewer #4

2. **This is a robust analysis using a well-established method. The objectives of the study are clearly stipulated. The design of the analysis is appropriate to answer the objectives of the study. One thing the authors should clarify is that the countries supported by APOC are less than the currently endemic countries. So this should be clarified so that the figures here not to look like for Africa.**

Response: We made this distinction clearer in the title and in our objectives where we added "*for countries formerly under the APOC mandate*" [line 100]. In addition, we explained in the Methods section the following: "*APOC covered 20 countries in Africa. In Rwanda and Kenya, the prevalence of palpable nodules was virtually zero during REMO surveys* [1–4]. *As a result, these countries were considered non-endemic for onchocerciasis. We therefore did not include these two countries in our analysis.*" [lines 128-131]

Results

Reviewer #1
Does the analysis presented match the analysis plan? Yes
Are the results clearly and completely presented? Yes
Are the figures (Tables, Images) of sufficient quality for clarity? Sufficient

Reviewer #2

3. **The analysis presented do match the plan but there are flaws in the data sources and therefore the conclusions. The figures are clear.**

Response: We elaborated on the concerns the reviewer described with responses to his/her comments in #15-#33.

Reviewer #3
No Response

Reviewer #4
The results are clear.

Conclusions

Reviewer #1
Are the conclusions supported by the data presented? Yes
Are the limitations of analysis clearly described? yes
Do the authors discuss how these data can be helpful to advance our understanding of the topic under study? yes
Is public health relevance addressed? Yes

Reviewer #2

4. **Conclusions are supported by the data presented but these are so general that this study does not really add to what is already known.**

Response: As mentioned in our response #1, this study adds to the general knowledge of the past, current, and future number of cases and burden of disease due to onchocerciasis in Africa, and where we can expect most cases. As very little evidence is available for national onchocerciasis control programmes, we think our results are useful to help guide countries on where to invest in intensified interventions. We agree with the reviewer that our estimates should be interpreted with caution, and we added a sentence on this in our Discussion: *"The presented number of cases with infection and morbidity should be considered as indicative only, as the estimates depend on uncertain assumptions regarding baseline endemicity levels, local transmission conditions and programmatic factors."* [lines 353-355]

Editorial and Data Presentation Modifications

Reviewer #1
See comments below.

Reviewer #2
See comments below.

Reviewer #3

5. **The title of the paper seems to imply that this paper will provide results for the whole of Africa, and should be changed to make it clear that it only covers (some) ex-APOC countries.**

Response: We modified the title to make clear that this analysis focusses on APOC countries. In addition, we have performed additional analysis to include Liberia, such that this analysis now covers all ex-APOC countries.

6. **The authors might like to explain why they did not include all the ex-APOC countries (i.e. Liberia and Kenya), and make it clear that they did not cover all ex-APOC countries in the abstract.**

Response: We have now included Liberia in our analysis. As APOC did not find onchocerciasis in Kenya and Rwanda, they excluded the country from active interventions and therefore we have omitted these countries from our analysis. This analysis now includes all ex-APOC countries with active interventions.

7. **The authors seem to put more emphasis on macrofilaricides as a solution to annual MDA effectiveness issues, as opposed to other interventions. They do mention other possibilities, but it seems that the authors are suggesting that macrofilaricides would be the solution of choice, or possibly the only solution in some cases (e.g. lines 293 and 284). Please can the authors explain this, or make some small revisions so that the solutions are less one-sided.**

Response: We indeed think that macrofilaricidal drugs will be needed to mop up hotspots and to treat clinical cases that may remain even after the achievement elimination of transmission. Reviewer 2 seems to agree with us (see comment #24). Nonetheless, we now also mention other options to mop up residual cases. We have rephrased our wording in the Background section: “*In areas where the targets are met in time and MDA is scaled down, some form of clinical treatment may be needed to address residual cases of infection and disease (e.g. macrofilaricidal drugs or drugs inducing permanent sterilization of adult female worms). For areas where MDA was implemented relatively recently or less successfully, additional interventions may be needed to speed up progress towards elimination, e.g. intensified MDA efforts, use of moxidectin (longer suppression of skin mf), additional vector control, or new macrofilaricidal or worm-sterilizing drug treatments.*” [lines 82-89]. We have also rewritten the conclusion: “*In the few countries where we predict continued transmission between now and 2030, intensified MDA could be combined with local vector control efforts, or the introduction of new drugs for mopping up residual cases of infection and morbidity.*” [lines 470-473]

Summary and General Comments

Reviewer #1

8. **Excellently written paper, very extensive, well documented modelling work. However as with all modelling work results depend on the available data and these are relatively poor. First the model relies on REMO data, collected in 1993. Since 1993 a lot of changes other than onchocerciasis elimination efforts may have changed the onchocerciasis prevalence in sub-Saharan Africa.**

Response: We have added the following to the discussion: *“The REMO data are the most comprehensive and accurate data available, and the basis of the current map of onchocerciasis in the 20 APOC countries. The oldest REMO surveys possibly date from 1993, but many were done more recently. These REMO data provide a good indication of pre-control prevalence in treated areas. The extent of hypo-endemic areas is more uncertain; unfortunately, data from recent and ongoing mapping efforts in hypoendemic areas with more sensitive diagnostic tools are not yet published.”* [lines 390-396

We further agree that multiple factors can influence prevalence over time, including reported history of control and spatiotemporal environmental changes, as we acknowledge in the

discussion section: *"We further note that we did not capture the impact of potential secular developments, such as climate change, deforestation, economic development, demographic transition, and urbanisation, which may lead or may have led to changes in transmission intensity and lower population densities in endemic areas than assumed here".* [lines 396-401]

9. **Second, the model relies on ivermectin coverage as reported by the onchocerciasis control/elimination programs. These coverage rates underestimate in many areas, mainly in those with most onchocerciasis related morbidity the true coverage.**

Response: We agree with the reviewer about uncertainty of reported therapeutic (and geographic) coverage. For this reason, we had already included two sensitivity analysis to cover this uncertainty where we assessed the impact of 10% and 20%-point overreporting of MDA coverage (see Figure 3). We are not sure whether the reviewer implies that the coverage data we used in our modelling study are an underestimation of what occurs in the field. We have not performed additional analysis of underreporting of MDA coverage, as so far, overreporting by countries of MDA activities occurs more often than underreporting.

10. **Bi-annual CDTI and vector control was not taking into account because as the authors state "it was considered this would not make a big difference given the already large effect of annual MDA with ivermectin on prevalence of acute clinical manifestations and incidence of chronic conditions." I can see it is difficult to take this into account. However we have seen that implementing bi-annual CDTI in combination with vector control made a major difference concerning onchocerciasis related morbidity (epilepsy) in northern Uganda (1). We hope that a similar approach would be implemented in other areas of high O volvulus transmission and very high onchocerciasis morbidity for example in South Sudan.**

(1.) N. Gumisiriza et al., Prevalence and incidence of nodding syndrome and other forms of epilepsy in onchocerciasis-endemic areas in northern Uganda after the implementation of onchocerciasis control measures. Infect Dis Poverty 9, 12 (2020).

Response: We agree that bi-annual MDA and vector control contribute to a more rapid decline in infection and reversible clinical manifestations, although to a much lesser extent to the prevention of new chronic morbidity (annual MDA is already very effective). We had already accounted for bi-annual MDA for several projects in Ethiopia and for one APOC project in Uganda (Phase V by 2015). We have now further updated our assumptions about MDA history to reflect that Uganda launched an elimination policy in 2007 with the objective to eliminate onchocerciasis through bi-annual treatment with ivermectin using community-directed treatment strategy and/or vector control/elimination. We performed new analyses using the updated history of control. See S1 Text (section 2.3).

11. **The authors mention they did not capture *"the impact of potential secular developments such as deforestation, economic development, demographic transition, and urbanisation, which may have led to changes in transmission intensity and a lower (future) population at risk living in endemic areas than assumed here."* They do not mention however security problems, occurring frequently in certain African countries, who may seriously complicate the distribution of ivermectin. The current COVID-19 epidemic is another example that has interrupted onchocerciasis elimination efforts.**

Response: We agree with the reviewer. We adapted the MDA database to account for security problems due to massive conflicts in for South Sudan, the CAR, and Liberia (newly added), and updated our analyses for these countries. We added a paragraph on the assumptions applied to account for security issues in the S1 Text, section 2.3. The main analysis now captures lower or missed MDA rounds during the years of the civil war, in alignment with available evidence. We have now also introduced disruptions in MDA distribution due to the COVID-19 pandemic by introducing one or two missed rounds in 2020. We added a sentence on the MDA disruptions we

accounted for in the main manuscript: "*We accounted for MDA disruptions due to security issues in countries in civil war (CAR, Liberia, South Sudan) and due to the COVID-19 pandemic.*" [lines 151-153]. Assumptions related to these security problems are now described in S1 Text, section 2.3.

12. **It is good the authors mention the association between high intensity O. volvulus infection and the development of epilepsy during childhood [28–31]. However they mention this important public health problem in onchocerciasis endemic regions only in the discussion. I propose to mention this already in the introduction. Indeed epilepsy is an onchocerciasis associated condition with very high disability, much higher than skin lesions and blindness.**

Response: We have not added OAE in our introduction as we did not include OAE in our analysis, and mentioning it in the introduction is misleading for the reader to understand what clinical manifestations we do take account of.

13. **The authors predict that the highest country-level mf prevalence in 2025 is expected in Gabon (19.4%), where currently no MDA programme is in place due to Loa loa co-endemicity, Mozambique (11.7%), Republic of Congo (9.5%), and the Democratic Republic of Congo (8.2%) (Table 3). I'm surprised that countries that experience war with complete disruption of CDTI programs such as South Sudan and CAR are not mentioned.**

Response: In response to another comment, we have now updated the analyses, accounting for MDA interruptions due to security issues, and the country-specific mf prevalences for South Sudan ended up being quite similar with our recent estimates. We have added a the following the Discussion: "*We predict that by 2030 the highest remaining prevalence of infection and morbidity is likely to be found in endemic areas of Gabon, Democratic Republic of Congo, and the Republic of Congo. However, the highest burden will be found in countries with the highest number of population at risk or those with the blinding savanna type of onchocerciasis (i.e. Nigeria, DRC, South Sudan, Cameroon).*" [lines 343-347]

14. **The authors state "Disability weights are based on a previously published multi-country study by Salomon et al.[31]. This study by Soloman analysed data from new web-based surveys of participants aged 18-65 years, completed in four European countries. These data are clearly not relevant for the O. volvulus infected person. For example a score is given for "Disfigurement: level 2. Person has a visible physical deformity that causes others to stare and comment. As a result, the person is worried and has trouble sleeping and concentrating." I have difficulties to believe that chronic irreversible skin problems in O. volvulus infected persons are associated with more disability than O. volvulus related serious vision problems.**

Response: There is much discussion about the blindness disability weights as used by the GBD, and published disability weights varied widely [*Braithwaite et al. 2017*]. Therefore, we added the following text to our Discussion: "*We used the latest GBD disability weights* [5]*, mapping the clinical manifestations of onchocerciasis to the lay descriptions with corresponding disability weights to estimate DALYs lost due to onchocerciasis. The disability weight for blindness was estimated at 0.187 by the GBD. Several previous estimates of the blindness disability weight were considerably higher* [6]*. The difference between earlier and current estimates may be primarily due to the dimensions covered by the disability weight: the GBD disability weights focus only on health loss due to blindness, not capturing other socio-economic consequences of blindness that may occur in Africa (e.g., related to livelihood, employment, accessibility of public resources and infrastructure). Such socio-economic consequences are therefore also not captured in our burden estimates. Quantifying this socio-economic burden was beyond the scope of this work, but it is important to acknowledge that this burden will be high, especially for blindness in the African context* [7–9]."[lines 439-449]

Reviewer #2

15. **I congratulate the authors on the enormous and detailed work that you have done on this paper, but with all the assumptions (that you have well explained), I wonder if it would not have been better to look at what is happening on the ground and to look at more impact data. There is too much emphasis on coverage data much of which is unrealistic. I feel for all the work done your conclusions are in fact very weak.**

Response: Collecting impact data would be useful if this could take place at a large enough scale. However, collecting impact data is very costly, and may not necessarily provide generalisable results without taking account of the pre-control infection levels and history of control (coverage levels and/or vector control). In our study, we do take account of these epidemiological and programmatic variables, and with that, we can reasonably well predict the impact over time thanks to intervention efforts for various endemicity settings. We have, however, now used published impact data to verify our results for several countries. Please also see points #1 and #9.

Specific comments to authors

16. **Line 76. Agreed; 217.5 million people needing PC but to be more realistic you should add how many are nearing the end of treatment.**

Response: We updated the number of people needing PC to the most recent WHO progress report, 2019-2020, and added the following sentence in the background: "*Thanks to long-term and large-scale onchocerciasis interventions in Africa, 1.2 million people no longer require MDA today* [10] *and several other regions may be close to elimination [refs.*" [lines 73-75]

17. **Line 86: You mention people with disease but you need to make clearer the difference between transmission of the disease and manifestations of disease (morbidity) in the text.**

Response: This was changed and checked throughout the remaining text (main manuscript and supplements), and adapted where necessary.

18. **Line 173. Some hypoendemic foci may be driven by spill over from hyperendemic foci but by no means all of them. Look at Gabon!**

Response: We agree with the reviewer, and adapted the text to better capture the mechanisms in ONCHOSIM about low transmission areas: "*Because ONCHOSIM simulates a single closed community of humans and does not consider mobility of infected humans and flies between multiple communities, it cannot simulate stable hypoendemic infection levels ~~(which are probably driven by spill-over from adjacent more highly endemic areas)~~. It is yet unclear which mechanisms contribute the stabilisation of infection across hypoendemic areas, but several hypotheses have been raised, which may not be mutually exclusive: spill-over from adjacent more highly endemic areas, more efficient transmission at low levels through additional density-dependent processes in the blackfly* [11]*, high levels of local individual variation in exposure to blackflies, and/or assortative mixing of small high-risk sub-populations* [12]*. Rather than explicitly modelling hypoendemic areas making assumptions, ...*" [lines 177-185]

19. **Line 189. You explain the modifications to GBD for the skin manifestations of onchocerciasis. A similar approach must be made to visual impairment and blindness as once again the GBD does not really take into account the impact of blindness in an onchocerciasis environment.**

Response: We did not modify the GBD approach, but only mapped the various onchocercal skin sequelae to the lay descriptions of the latest GBD. This was not necessary for vision loss (which GBD provided very clear definitions for). Please see also our response to comment #14, and the added text in the Discussion about disability weights for blindness.

20. **Line 228. OED was hardly measured in the APOC region. Most of the surveys done by APOC in the early days remained unpublished and were not followed up.**

Response: There are various publications on OED prior to the initiation of MDA interventions, but indeed most of these studies were performed in West-Africa. Our model for OED was calibrated using data from the savanna bioclimate [13] and from the forest and mixed savanna-forest bioclimates, including some data from the DRC [14–23]. Details of the calibration and data used can be found in the recently accepted accompanying paper (Vinkeles-Melchers 2021 PLoS NTDs). We slightly changed the sentence to make clear this was a result of our study: "*The total pre-control prevalence of OED was predicted to be 0.8% (615.5 thousand cases), mostly due to visual impairment in savanna areas (82.2% of all OED cases in 1990; Table S3.1 in S3 Text).*" [line 243-254]

21. **Line 246. I think you may need to add South Sudan to your list. Surveys in the APOC era showed the results reported were not realistic, and there has been virtually no treatment for a period of about 10 years until 2017 when treatment has started to scale up again. There are undoubtedly new cases of blindness and there is a lot of epilepsy in the south which you have mentioned but will be a serious problem for morbidity management later.**

Response: We received various documents on the history of control in South Sudan from an onchocerciasis expert working in South Sudan (Prof. R Colebunders, collaborates with MoH South Sudan, and local experts from mainly Maridi county), and we were able to adapt the MDA database on the basis of this information. We adapted the history of control for South Sudan, also in line with the comments by reviewer 1 (#11 and #13) and performed new analyses. We modelled onchocerciasis for seven project areas in South Sudan, and for two of them we only assumed very late start of MDA (>2021). For three APOC-projects, we have now indeed modelled a complete disruption of MDA during the war of five to seven years. Two APOC-projects (in western South Sudan) did not experience war, and we found in documents that MDA was continued in these regions. Please see S1 Text, section 2.3 for a detailed description of our assumptions on the therapeutic coverage in South Sudan and the literature used.

22. **Line 298. I agree that excess mortality due to OED needs to be taken into account but there is definitely a difference between blindness trends due to vector control and those due to ivermectin. With ivermectin use more early cases are reversible where these are not impacted early enough with vector control.**

Response: This point is indeed valid for areas where vector control would have been implemented without mass distribution of ivermectin (e.g. in areas previously under the OCP mandate). However, if MDA with ivermectin is implemented, this is the main driving force behind a reduction in new cases of blindness (because of its direct effect on infection in humans, which vector control doesn't have). In APOC areas, there has only been very little vector control applied and – to our knowledge – never without ivermectin MDA as main strategy. So, we think we have been able to capture the trends in morbidity prevalence realistically, and that the different impact of interventions on case estimates have been captured well as in APOC areas ivermectin MDA was the main strategy for onchocerciasis control.

23. **Line 350. In these days of integration of PC it is unrealistic to not take into account treatments for LF, particularly in hypoendemic areas.**

Response: We used the MDA database earlier compiled by Kim *et al* [24], using MDA history as shared by the individual countries up to 2013. After that, we completed the MDA database with information published on the ESPEN portal. ESPEN reports combined therapeutic coverages of ivermectin distribution for LF and onchocerciasis in onchocerciasis-endemic areas. PC for LF was thus taken into account for most onchocerciasis-endemic areas in our study. The only exception are the P5-projects, for which we do not have data on history of control. Of the 17 P5-projects we model, 13 projects are co-endemic for loiasis, and henceforth ivermectin is contraindicated as a drug regimen in LF programmes. The effect of not taking account of PC for LF is therefore assumed

to be minimal in our estimates. We added a section on the uncertainty of these P5 projects and adapted the text on PC for LF:
The presented number of cases with infection and morbidity should be considered as indicative only, as the estimates depend on uncertain assumptions regarding baseline endemicity levels, local transmission conditions and programmatic factors. Numbers presented for any hypo-endemic areas left untreated by APOC should be considered with particular caution. Our P5 areas include all areas with an estimated nodule prevalence between 5% and 20%, based on a geostatistical analysis of REMO survey data. However, in most areas hypo-endemicity remains to be confirmed in the field through onchocerciasis elimination mapping, although the ESPEN website suggest that elimination mapping may not, or no longer be required in seven former APOC-countries (i.e., Burundi, Chad, Equatorial Guinea, Liberia, Malawi, Tanzania and Uganda). In some of the P5 areas, onchocerciasis infection prevalences may have been reduced thanks to MDA programmes against lymphatic filariasis in loiasis-free areas or the collateral benefits of other drug programmes (e.g., ivermectin against scabies in Ethiopia [25]*) may have led to minor overestimations of the aforementioned burden estimates for these areas."* [lines 353-365]

24. **Line 384. I agree with your conclusion that macrofilaricidal drugs will be needed to mop up hot spots and treating clinical cases and the sequelae of onchocerciasis may well continue after elimination of transmission will have been achieved.**

Response: Thank you. We adapted the sentence slightly to also accommodate the requests by reviewer 1. See comment #7.

25. **Figure 1 DALYs lost due to skin nodules???**

Response: Palpable nodules are considered a skin morbidity, as its manifestation can lead to stigmatisation and shame. It was therefore recommended through expert opinion (Dr. Michele Murdoch) to include palpable nodules as a clinical manifestation of onchocerciasis in the total burden estimates. Palpable nodules were also included in the onchocerciasis disease estimates for GBD 2013 Study. Palpable nodules have a low scoring of disability weight, but as many people are affected by nodules, the contribution to the total disease burden is non-negligible. We added a sentence on this in the Methods section: "*..., palpable nodules (which may lead to stigmatisation and shame [REF]), ...*" [line 166].

26. **Figure 1 and 3. Clinical signs of irreversible skin disease remain, I agree. I am not sure how much these really impact daily life once under treatment with ivermectin. There are some social consequences but I am not sure how you arrive at these DALYs**

Response: Treatment with ivermectin will unfortunately not impact the occurrence of irreversible disease manifestations. There is evidence that (irreversible) onchocercal morbidity leads to stigmatisation, shame, and lack of sexual intimacy, but there is indeed uncertainty of the socio-economic burden of these manifestations on people's lives. Irreversible skin diseases (depigmentation, atrophy, hanging groin) are not expected to resolve upon ivermectin intake, and the impact on a person's daily life will remain the same pre- as post-treatment.

We follow the methods of the GBD to estimate the burden of disease due to onchocerciasis. See also our response to comment #14 on the socio-economic burden of onchocerciasis.

Supplementary material.

27. **S2 No mention of Loa loa in DRC. It is the worst affected country and will play a major role in the treatment of hypoendemic areas.**

Response: Thank you for spotting this oversight. We corrected this in S2 Text.

28. **S2 and S4. I know assumtions have to be made but having straight line coverages between 75 and 80% are not at all realistic, especially in unstable countries which as you say is where most of the burden will be in the future.**

Response: Obviously there is no data on what the MDA therapeutic coverage will be across onchocerciasis-endemic countries in the coming years. We therefore used the most recent reported therapeutic coverage as reported by the countries to ESPEN. This data shows that even for unstable countries, such as for example the DRC, Liberia, Mozambique, and South Sudan, the achieved coverage has successfully increased to 70% to 80%. We acknowledge that there are many uncertainties of future MDA delivery, including events similar to the COVID-19 outbreak that massively influenced MDA delivery in 2020. However, we somewhat accounted for these uncertainties by performing several sensitivity analyses around MDA delivery, i.e. overreporting, earlier and later start of MDA programmes in hypoendemic areas. See also our response to comment #9.

Just a few specific points on the country profiles.

29. **Malawi is only working on cross border issues with Mozambique**

Response: We have modelled onchocerciasis in two foci in Malawi, Thyolo Mwanza and an extended area. The area of Thyolo Mwanza is located at the border area of Mozambique, and the extended area was later identified as onchocerciasis-endemic and henceforth received MDA. In our analysis, we use these project areas as originally defined by APOC. For both areas, we predict that infection levels were already ≤0.1% by 2020, which fits well with the reviewer's comment about Malawi only working on cross-border issues at the moment.

30. **3 States in Nigeria have already stopped treatment yet you graph shows treatment continuing**

Response: The Ministry of Health of Nigeria together with their partners have indeed interrupted transmission of river blindness in two states which are now conducting post-treatment surveillance, Plateau and Nasarawa. This is now reflected by the graphs. In an additional two states, Delta and Ebonyi, interruption of transmission likely has taken place, but we were not able to locate official documentation about this. Worth noting is that these assumptions of treatment continuing (or not) will not affect case and burden estimates for areas where transmission has been interrupted.

31. **South Sudan. There was virtually no treatment for up to 10 years and even an APOC survey found the reported coverage to be inadequate. Treatment started again in 207 but is still only just scaling up to 100% coverage. There is a lot of morbidity as already mentioned.**

    MDA was initiated in Maridi in 1997, but it was interrupted after a few rounds due to violent conflicts [33]. It was only resumed in 2017, when 40 .8% of the population took ivermectin [7]. In November 2019, biting rates up to 202 flies/man/hour were noted close to the Maridi dam and an onchocerciasis seroprevalence of 66.7% was documented using the OV16 rapid test among children 7-9 years old [32]). These figures suggest that currently, there is high ongoing onchocerciasis transmission in the Maridi area.

Response: We agree with the reviewer, and we have therefore adapted the MDA database and updated our analyses. Please see our answer to comments #11 and #13.

32. **Uganda. Treatment has already stopped in all foci apart from 1 in the North where the LRA was active.**

Response: According to reports, onchocerciasis has been eliminated or transmission has been interrupted in 15 out of 17 foci in 2020 (Habomugisha 2021; Katabarwa et al. 2018). In one focus, transmission interruption is suspected and in one foci transmission is still ongoing (Madi-Mid

North). The areas where MDA has stopped correspond to two APOC projects (Phases II and IV), and we have adapted S2 Text and S4 Text to show this halt in MDA. It is unclear if Uganda still has unknown hypoendemic areas (in our study represented by a P5-project, but we have added a section on the uncertainty of P5-projects; see our response to comment #23). To highlight the successes of the onchocerciasis efforts in, amongst others, Uganda, we have added in the Discussion: "*In other countries, the long-standing control interventions in Africa will have reduced infection and morbidity to negligible levels, such as across large areas in Burundi, Equatorial Guinea, and Uganda. For example, For Uganda, these predictions are in line with empirical evidence: 15 out of 17 onchocerciasis-endemic foci in Uganda are currently under post-treatment surveillance or have eliminated onchocerciasis* [26,27]." [lines 347-351]

33. Burundi. Most surveys have been negative. There is only the problem of security before doing impact surveys.

Response: WHO-ESPEN identified five regions in Burundi that are still endemic for onchocerciasis and requiring MDA in Burundi by 2019 (ESPEN Portal). These are likely regions that await formal status for post-treatment surveillance. We agree with the reviewer that most of the country has been mapped and surveyed, and that as a result there are no more areas that require onchocerciasis elimination mapping. Our results for 2020 are in line with the reviewer's comment, and we estimate very little cases with infection remaining for most APOC-projects in Burundi. The only exception is the P5 APOC-project, and we added a general statement about the uncertainty of the P5-projects in the Discussion section (see our response to comment #23).

Reviewer #3

34. This is a good and useful paper which deserves publication. It is well written, easy to read and needs very little revision. However, please note that if the other (accompanying) paper submitted by the authors (which I have not been asked to review) is rejected or requires major revision, that may have consequences for this paper.

Response: Thank you. The accompanying paper has been accepted by the Journal.

Reviewer #4

The analysis by Vinkeles Melchers and colleagues on the burden of skin disease and eye disease due to onchocerciasis in Africa for 1990, 2015, and 2025 is very important. The estimates provide the burden of skin and eye diseases due to onchocerciasis what progress has been achieved and what efforts are required to achieve the 2025 targets and the 2030 NTD Roadmap targets. I have the following comments though.

35. The title says Africa in fact the analysis was focusing only in the APOC countries. This should come out in the title as well.

Response: This has been adapted. Please see our response to comment #2.

36. The authors should clarify on the list of APOC supported countries and what is the difference between the current scope of the onchocerciasis programme compared with the APOC.

Response: We have now also added Liberia to our analysis to complete the list of endemic countries formerly covered by APOC. Please also see our responses to comments #2, #5 and #6 with regard to Kenya and Gabon being categorised as "non-endemic".

37. The authors have the liberty to analyse the data for APOC supported countries, but they should make clear distinction that there are additional countries which were not covered by APOC now in the elimination programme in Africa.

Response: Good suggestion. We added the following sentence in the background to clarify this point: "*Another 7.6 million people were estimated to be infected in areas previously under the*

mandate of the Onchocerciasis Control Programme (OCP, 1974-2002) in West-Africa, prior to the initiation of interventions [29].” [lines 70-72]

References

1. Zouré H, Noma M, Tekle AH, Amazigo UV, Diggle PJ, et al. (2014) The geographic distribution of onchocerciasis in the 20 participating countries of the African Programme for Onchocerciasis Control: (2) pre-control endemicity levels and estimated number infected. *Parasites & vectors* **7**.

2. Noma M, Zouré HG, Tekle AH, Enyong PA, Nwoke BE, et al. (2014) The geographic distribution of onchocerciasis in the 20 participating countries of the African Programme for Onchocerciasis Control: (1) priority areas for ivermectin treatment. *Parasites & Vectors* **7**: 325.

3. Roberts JM, Neumann E, Göckel CW, Highton RB (1967) Onchocerciasis in Kenya 9, 11 and 18 years after elimination of the vector. *Bulletin of the World Health Organization* **37**: 195–212.

4. Newell ED (1994) Onchocerciasis in Rwanda? *Transactions of the Royal Society of Tropical Medicine and Hygiene* **88**: 493.

5. Salomon JA, Haagsma JA, Davis A, de Noordhout CM, Polinder S, et al. (2015) Disability weights for the Global Burden of Disease 2013 study. *Lancet Glob Health* **3**: e712–e723.

6. Braithwaite T, Taylor H, Bourne R, Keeffe J, Pesudovs K (2017) Does blindness count? Disability weights for vision loss. *Clinical and Experimental Ophthalmology* **45**: 217–220.

7. Wagbatsoma VA, Okojie OH (2004) Psychosocial effects of river blindness in a rural community in Nigeria. *Journal of The Royal Society for the Promotion of Health* **124**: 134–136.

8. Alonso L, Murdoch M, Jofre-Bonet M (2009) Psycho-social and economical evaluation of onchocerciasis: a literature review. *Soc Med* **4**: 8–31.

9. Evans TG (1995) Socioeconomic consequences of blinding onchocerciasis in West Africa. *BULL WHO* **73**: 495–506.

10. WHO (2020) Elimination of human onchocerciasis: progress report, 2019-2020. *Weekly epidemiological record* **45**: 545–556.

11. Basáñez MG, Remme JHF, Alley ES, Bain O, Shelley AJ, et al. (1995) Density-dependent processes in the transmission of human onchocerciasis: Relationship between the numbers of microfilariae ingested and successful larval development in the simuliid vector. *Parasitology* **110**: 409–427.

12. de Vos AS, Stolk WA, de Vlas SJ, Coffeng LE (2018) The effect of assortative mixing on stability of low helminth transmission levels and on the impact of mass drug administration: Model explorations for onchocerciasis. *PLoS Neglected Tropical Diseases* **12**: e0006624.

13. Remme J, Dadzie KY, Rolland A, Thylefors B (1989) Ocular onchocerciasis and intensity of infection in the community. I. West African savanna. *Tropical medicine and parasitology : official organ of Deutsche Tropenmedizinische Gesellschaft and of Deutsche Gesellschaft fur Technische Zusammenarbeit (GTZ)* **40**: 340–347.

14. Remme JHF (2004) The Global Burden of Onchocerciasis in 1990. *Geneva: WHO.*

15. Brown R, Shannon R (1989) Prevalence, intensity and ocular manifestations of Onchocerca volvulus infection in Dimbelenge, Zaire. *Annales de la Societe belge de medecine tropicale* **69**: 137–142.

16. Henry MC, Maertens K (1990) The onchocerciasis focus at Kinsuka/Kinshasa (Republic of Zaire) in 1985. II. Parasitological and clinical aspects. *Annals of tropical medicine and parasitology* **84**: 493–502.

17. Whitworth JA, Gilbert CE, Mabey DM, Maude GH, Morgan D, et al. (1991) Effects of repeated doses of ivermectin on ocular onchocerciasis: community-based trial in Sierra Leone. *Lancet (London, England)* **338**: 1100–1103.

18. Whitworth JA, Gilbert CE, Mabey DM, Morgan D, Foster A (1993) Visual loss in an onchocerciasis endemic community in Sierra Leone. *The British journal of ophthalmology* **77**: 30–32.

19. Kayembe DL, Kasonga DL, Kayembe PK, Mwanza J-CK, Boussinesq M (2003) Profile of eye lesions and vision loss: a cross-sectional study in Lusambo, a forest-savanna area hyperendemic for onchocerciasis in the Democratic Republic of Congo. *Tropical medicine & international health : TM & IH* **8**: 83–89.

20. Resnikoff S, Pascolini D, Etya’ale D, Kocur I, Pararajasegaram R, et al. (2004) Global data on visual impairment in the year 2002. *Bulletin of the World Health Organization* **82**: 844–851.

21. Dadzie KY, Remme J, Baker RH, Rolland A, Thylefors B (1990) Ocular onchocerciasis and intensity of infection in the community. III. West African rainforest foci of the vector Simulium sanctipauli. *Tropical medicine and parasitology : official organ of Deutsche Tropenmedizinische Gesellschaft and of Deutsche Gesellschaft fur Technische Zusammenarbeit (GTZ)* **41**: 376–382.

22. Dadzie KY, De Sole G, Remme J (1992) Ocular onchocerciasis and the intensity of infection in the community. IV. The degraded forest of Sierra Leone. *Tropical medicine and parasitology : official organ of Deutsche Tropenmedizinische Gesellschaft and of Deutsche Gesellschaft fur Technische Zusammenarbeit (GTZ)* **43**: 75–79.

23. Dadzie KY, Remme J, Rolland A, Thylefors B (1989) Ocular onchocerciasis and intensity of infection in the community. II. West African rainforest foci of the vector Simulium yahense. *Tropical medicine and parasitology : official organ of Deutsche Tropenmedizinische Gesellschaft and of Deutsche Gesellschaft fur Technische Zusammenarbeit (GTZ)* **40**: 348–354.

24. Kim YE, Remme JHF, Steinmann P, Stolk WA, Roungou JB, et al. (2015) Control, elimination, and eradication of river blindness: scenarios, timelines, and ivermectin treatment needs in Africa. *PLoS Negl Trop Dis* **9**.

25. Enbiale W, Baynie TB, Ayalew A, Gebrehiwot T, Getanew T, et al. (2020) “Stopping the itch”: Mass drug administration for scabies outbreak control covered for over nine million people in Ethiopia. *Journal of Infection in Developing Countries* **14**: 28S-35S.

26. P. Habomugisha (2021) Uganda treatments and impact. The Carter Center; River blindness elimination program.

27. Katabarwa MN, Lakwo T, Habomugisha P, Unnasch TR, Garms R, et al. (2018) After 70 years of fighting an age-old scourge, onchocerciasis in Uganda, the end is in sight. *International Health* **10**: i79–i88.

28. ESPEN Portal (n.d.) Onchocerciasis maps. *Expanded Special Project for Elimination of Neglected Tropical Diseases (ESPEN) World Health Organization Regional Office for Africa Available at: https://espen.afro.who.int/ Assessed on: 11 May 2021*.

29. O'Hanlon SJ, Slater HC, Cheke RA, Boatin BA, Coffeng LE, et al. (2016) Model-based geostatistical mapping of the prevalence of Onchocerca volvulus in West Africa. *PLoS neglected tropical diseases* **10**: e0004328.
